# Enantioselective Aminosilylation of Alkenes by Palladium/Ming‐Phos‐Catalyzed Tandem Narasaka–Heck/Silylation Reaction

**DOI:** 10.1002/advs.202403470

**Published:** 2024-07-05

**Authors:** Kangning Cao, Jie Han, Wenshao Ye, Dejun Hu, Zihao Ye, Junfeng Yang, Junliang Zhang, Fener Chen

**Affiliations:** ^1^ Engineering Center of Catalysis and Synthesis for Chiral Molecules Department of Chemistry Fudan University Shanghai Shanghai 200433 China; ^2^ School of Chemical & Environmental Science Shaanxi University of Technology Hanzhong 723001 China; ^3^ Department of Chemistry Fudan University 2005 Songhu Road Shanghai 200438 China; ^4^ School of Chemistry and Chemical Engineering Henan Normal University Xinxiang Henan 453007 China; ^5^ Zhuhai Fudan Innovation Institute Zhuhai 519000 China

**Keywords:** asymmetric catalysis, Aza‐Heck, DFT, palladium, pyrrolines

## Abstract

A Pd‐catalyzed enantioselective aminosilylation of alkenes via tandem Aza‐Heck/silylation reaction under Pd/Sadphos catalysis is disclosed. A wide array of oxime esters and silicon reagents are tolerated, furnishing the chiral pyrrolines bearing one quaternary or two contiguous stereocenters in good yield with high enantioselectivity. Not only terminal alkenes but also tri‐substituented internal alkenes successfully participate in the reaction, delivering vicinal stereocenters in complete diastereoselectivity and high enantioselectivity. DFT study is conducted to probe the reaction pathway and the origin of the enantioselectivity, which revealed that the stereoinduction arises from the weak interaction between the aromatic ring of the substrate fragment and naphthyl group in the ligand.

## Graphical abstract

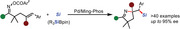

## 1 Introduction

**Figure 1:**
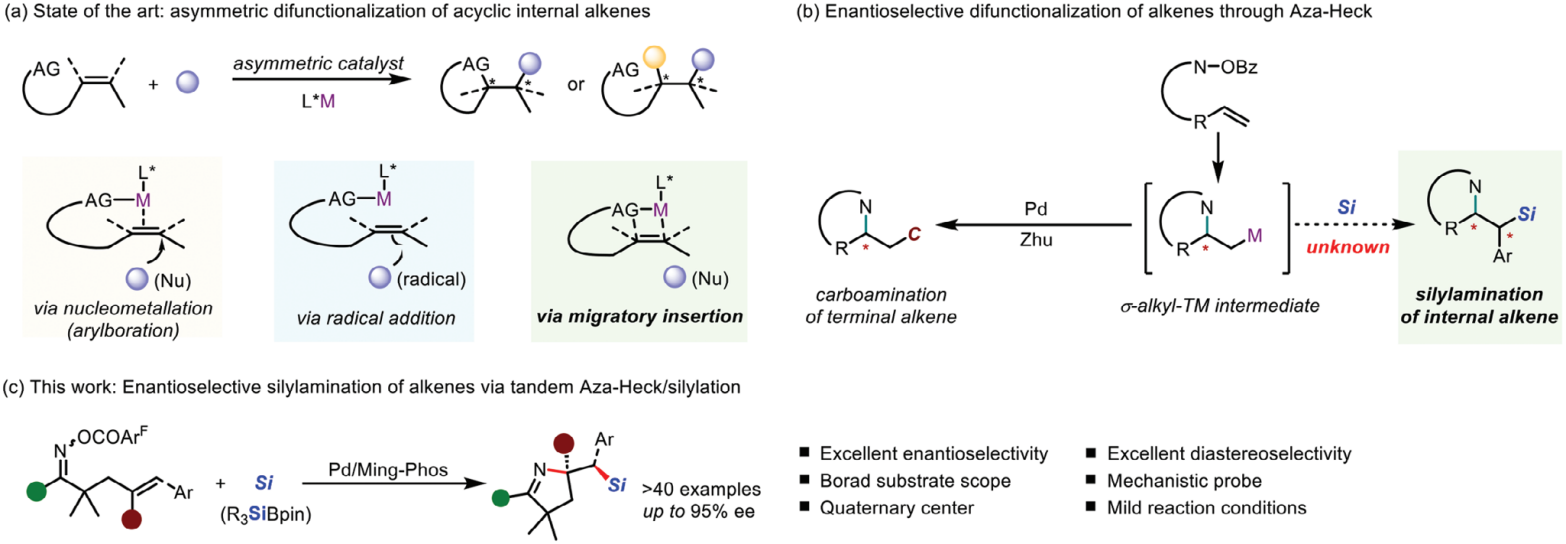
Background and reaction design.

Transition‐metal‐catalyzed asymmetric difunctionalizations of alkenes have emerged as potent techniques for the sustainable synthesis of intricate chiral organic molecules, which allow the rapid construction of two adjacent carbon–carbon and/or carbon–heteroatom chemical bonds, along with the formation of stereogenic centers. For selected reviews on asymmetric difunctionalization of alkenes, see ref. [1] Despite notable advancements in the asymmetric functionalizations of terminal and cyclic alkenes, achieving enantioselective transformations of internal acyclic alkenes has met with formidable challenges. For selected reviews and examples on asymmetric difunctionalization of internal alkenes, see ref. [2] The lagged development in this area mainly arises from the inherent difficulty in simultaneously and precisely controlling the regio‐, diastereo‐, and enantioselectivity of the two newly formed adjacent stereocenters. Another challenge lies in the low binding affinity of internal alkene with transition‐metal (TM) species. To address the limitation, the chelating strategy has been commonly employed in the difunctionalization of internal alkenes to stabilize key intermediates and guide regioselectivity.^[^ ^1h,i,l^ ^]^ Several approaches have been developed via the chelating strategy, including the nucleometallation^[^ ^3^ ^]^ and single‐electron transfer via a radical relay process,^[^ ^4^ ^]^ especially the migratory insertion via transition‐metal (TM), exampled by dicarbofunctionalization,^[^ ^2^, ^5^ ^]^ arylfluorination,^[^ ^6^ ^]^ and arylborylation (**Figure** **1**a).^[^ ^7^ ^]^ The Narasaka–Heck process, which typically involves the transition‐metal (TM)‐catalyzed oxidative addition of oxime esters, followed by intramolecular aminometallation of alkenes and β‐hydride elimination, has emerged as a potent tool for alkenes functionalization via the chelating strategy.^[^ ^8^ ^]^ The enantioselective variants, originally pioneered by Bower and co‐workers, enable enantioselective Narasaka–Heck cyclization to construct chiral dihydropyrroles containing a quaternary stereocenter.^[^ ^9^ ^]^ Subsequently, significant progress has been achieved by Zhu,^[^ ^10^ ^]^ Shu,^[^ ^11^ ^]^ and Wang^[^ ^12^ ^]^ through the combination of asymmetric aza‐Heck cyclizations with sequential TM‐catalyzed cross‐coupling reactions, which substantially advanced the existing carboamination approaches. Notwithstanding this elegant progress achieved, most developments were limited to the terminal alkenes‐tethered oxime esters (Figure 1b).

Recognizing the significance of chiral organosilanes as valuable molecular scaffolds in medicinal chemistry and materials science,^[^ ^13^ ^]^ we propose that the chiral alkyl‐Pd(II) intermediates generated by asymmetric migratory insertion could be intercepted by silyl‐Cu species, resulting in the optically active aminosilylation product.^[^ ^14^ ^]^ This aminosilylation product could also serve as a potential precursor for chiral amino alcohols. In this scenario, the primary challenge lies in the potential simultaneous occurrence of the TM‐mediated migratory insertion pathway and a radical pathway, which could compromise enantioselective control significantly.^[^ ^15^ ^]^ Additionally, the asymmetric environment created by the chiral ligand might be influenced by the presence of nucleophilic reagents. Beyond the difficulties mentioned above, the low binding affinity of TM to internal alkenes poses an additional challenge, given that existing tandem approaches only accommodate terminal alkenes. Following our continuous interest in alkene difunctionalization,^[^ ^16^ ^]^ herein, we present the first example of the asymmetric aminosilylation of alkenes via Aza‐Heck cyclization. Utilizing homemade chiral sulfinamide phosphine ligands (Sadphos) and Pd catalysis, a series of chiral dihydropyrroles containing one quaternary or two contiguous stereocenters can be readily obtained, which significantly expands the domain of asymmetric Aza‐Heck process (Figure 1c).

## 2 Results and Discussion

### 2.1 Reaction Optimizations

To test our hypothesis, we commenced catalytic asymmetric aminosilylation studies by employing terminal alkenes‐tethered oxime ester **1a** as substrate and commercially available Me_2_PhSiBpin **2** as the silicon source, using Pd(dba)_2_, CuBr and CsOAc in cyclopentane (**Table** **1**). At first, a batch of commercially available chiral ligands was examined. However, none of them delivered the desired product. The oxime ester remained intact in most cases. Given their excellent performance of Sadphos ligands in the Pd‐catalyzed asymmetric coupling reaction,^[^ ^17^ ^]^ subsequently, we explored the Sadphos ligands with different skeletons, Gratifyingly, Xanthene derived PC‐Phos and GF‐Phos afforded the desired product in excellent yield, albeit with poor enantioselectivity. Ligands with smaller bite angles, such as **M1**, and Xu‐Phos, gave the corresponding product in 50–60% yields and 40–50% ee, with superior performance for Ming‐Phos. The relatively poor enantio‐control of Xu‐Phos is probably ascribed to the stronger SET character of the oxidative addition via electron‐rich Pd(0) catalyst.^[^ ^15^ ^]^ If the nitrogen of Ming‐Phos was masked by the methyl group (**M2**), the enantioselectivity could be improved slightly (61% ee). Remarkably, modification of the substituents on the aromatic ring has a great influence on the enantioselectivity of the reaction. After substantial screening, **M7** with a deuterated methyl group on the N atom was identified to give the highest ee. The enhancing effect of the deuterium atom on the enantioselectivity has been proved previously.^[^ ^17e^ ^]^ Based on these results, **M7** was then selected as the optimal ligand and further examined under various conditions (See supporting information). The best performance was achieved in cyclopentane at 0 °C, affording the desired aminosilylation product **3a** in 87% yield with good enantioselectivity (91%) with 5 mol% catalyst loading. It is emphasized that activating acyl group plays an essential role in the reaction to occur. The parent *O*‐benzoyl oxime ester failed to deliver the desired product, with most of **1a** hydrolyzed to ketone. The *O*‐Perfluorobenzoyl oxime ester gave **3a** in 92% yield and 58% ee, and the rest was cyclized and protonated. These results indicate that the leaving group is involved in the asymmetric cyclization and the following silylation process. After extensive screening of a series of polyfluoro benzoyl substituents, we found that 2,3,4‐trifluoro benzoyl substituted **1a** gives the best performance in terms of both yield and ee of the product.

**Table 1:** Optimization of reaction conditions.^a)^Reaction condition: **1a** (0.1 mmol), **2a** (0.25 mmol), Pd(dba)_2_ (5 mol %), ligand (10 mol%), CsOAc (4 equiv), CuBr (10 mol%), Cyclopentane (1 mL), Ar = 2,3,4‐F_3_C_6_H_2_, 0 °C, 40 h; the yields were determined by GC analysis with *n*‐dodecane as internal standard.

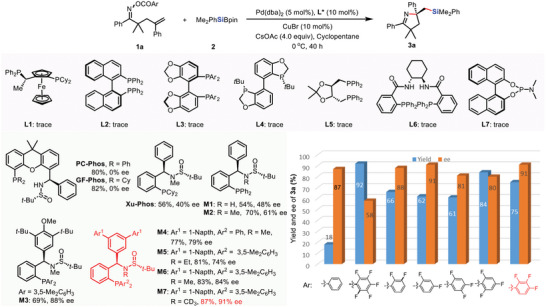

### 2.2 Substrate Scope

**Figure 2:**
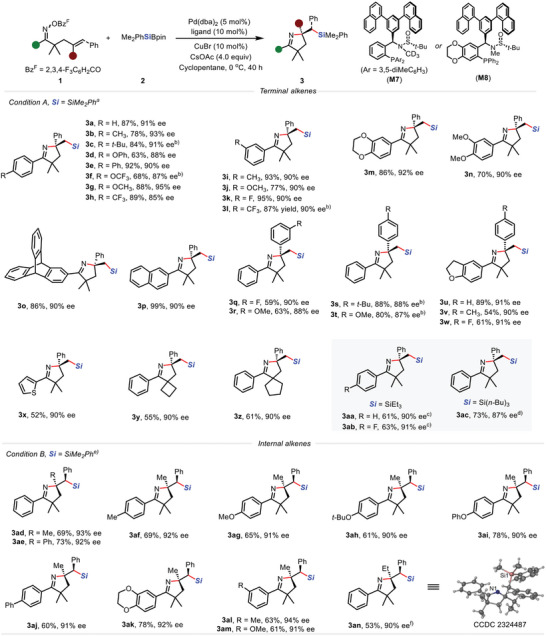
Exploration of the Substrate Scope. Unless otherwise noted, reaction conditions are as follows: a) Condition A: **1** (0.3 mmol), **2** (0.75 mmol), Pd(dba)_2_ (5 mol%), **M7** (10 mol%), CsOAc (4 equiv), CuBr (10 mol%), Cyclopentane (3 mL), 0 °C, 40 h. b) **M3** (10 mol%), at ‐10 °C. c) **M3** (10 mol%), at 0 °C. d) At 15 °C. e) Condition B: **1** (0.3 mmol), **2** (0.75 mmol), Pd(OAc)_2_ (5 mol%), **M8** (10 mol%), Rb_2_CO_3_ (2 equiv), CuBr (10 mol%), Cyclopentane (3 mL), 25 °C, 30 h. f) CsOAc (4.0 equiv).

With the established reaction conditions in hand, we started to examine the substrate scope. As summarized in **Figure** **2**, both electron‐withdrawing and electron‐donating substituents at the para and meta positions of the aromatic ring with respect to both the oxime ester and alkene could be well compatible (**3a** to **3w**), giving rise to structurally diverse dihydropyrole products **3** in high yields and enantioselectivities (**3a** to **3w**, 85 to 94% ee). Electron‐rich, ‐neutral, and ‐poor substituents, such as alkoxy (**3d**, **3g**, **3j**, **3m** and **3n**), fluorine (**3k**), trifluoromethyl (**3h**, **3l**), trifluoromethoxy (**3f**) were all compatible. Heterocycle, such as thiophene, also performed well to deliver the corresponding product **3x**. Of note, the *gem*‐dialkyl group is essential for the reaction to occur due to the Thorp‐Ingold effect (**3y** and **3z**). Replacement of the phenyl group on the alkene to alkyl group significantly decrease the yield (see Figure S1, Supporting Information for details). Remarkably, the silicon reagent could also be extended to the simple trialkylsilylborane reagent, such as triethylsilyborane and tributylsilborane (**3aa** to **3ac**).

Optically pure amino alcohols are important structural motifs in pharmaceutical and bioactive natural products.^[^ ^18^ ^]^ Considering silyl groups could serve as the precursor of alcohols,^[^ ^19^ ^]^ vicinal amino secondary alcohols could be readily accessed if the asymmetric aminosilylation reaction could be extended to the challenging internal alkenes. Subsequently, we turned our attention to the tri‐substituted alkenes. The optimal reaction conditions could be readily identified by modification of the ligand, and the catalytic system worked well for a range of oxime esters with different substituents at the para and meta positions of the aromatic ring, affording the corresponding aminosilylation product in high yields with excellent enantioselectivities (**3ad** to **3an**, 91 to 97% ee) with complete diastereocontrol. Yield and enantioselectivity were slightly decreased if the methyl group on the alkene was replaced with the ethyl group. The absolute configuration of the product **3a** was determined by X‐ray analysis of its derivatives (see Supporting Information).

### 2.3 Proposed Mechanism and Product Derivation

**Figure 3:**
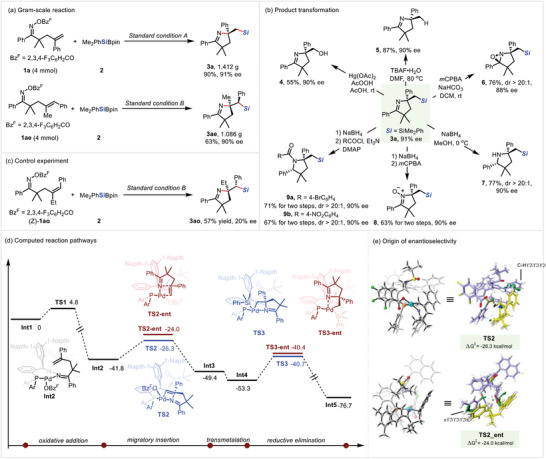
Proposed Mechanism and Product Derivation.

The gram‐scale syntheses of **3a** and **3ae** were successful, with no erosion of efficiency in yield, enantioselectivity, and diastereoselectivity under the standard conditions (**Figure** **3**a). **3a** could be converted to the corresponding chiral alcohol **4** using Fleming‐Tamao oxidation.^[^ ^20^ ^]^ The silyl group of **3a** could also be cleanly removed in the presence of TBAF to afford **5** in 87% yield with 90% ee.^[^ ^21^ ^]^ Direct *m*CPBA‐mediated oxidation of compound **3a** gave the oxaziridine **6** as a single diastereomer in 76% yield with 88% ee.^[^ ^12^ ^]^ Reduction of the imine moiety of **3a** by NaBH_4_ gave pyrrolidine **7**, followed by oxidation afforded the product **8**.^[^ ^22^ ^]^ The N atom on the pyrrolidine **7** could be simply protected by treating with 4‐bromobenzenesulfonyl chloride or 4‐nitrobenzenesulfonyl chloride in the presence of triethylamine, furnishing **9a** or **9b** (Figure 3b).^[^ ^23^ ^]^

To gain insight into the details of this asymmetric modulation, a series of control experiments were carried out (Figure 3c). Notably, if the (*Z*)−**1ao** was subjected to the standard condition, the product was obtained in 57% yield with 20% ee, as well as excellent diastereoselectivity. The result suggests that the geometry of the alkene affects the enantioselectivity significantly. However, in contrast to Bower's approach, no protodecarboxylation process was observed under the standard condition and the control experiment of the cesium 2,3,4‐trifluorobenzoate, probably owning to the relatively mild reaction condition (see Figure S4, Supporting Information for details).

### 2.4 Mechanistic Investigation

To illuminate the origin of the enantioselectivity of the aminosilylation pathway, the reaction pathway was investigated through the DFT calculation using Gaussian 09 (Figure 3d). As established in the literature,^[^ ^24^, ^25^ ^]^ the reaction is initiated by the oxidative addition of the Pd(0) into the N─O bond of the oxime ester (**TS1**), generating the imino‐Pd(II) intermediate **Int2** with an energy barrier of 4.8 kcal mol^−1^. Then, the imino‐Pd(II) intermediate **Int2** coordinates with the alkene, followed by the intramolecular migratory insertion, generating the intermediate **Int3** with a transition state of **TS2** (−26.3 kcal mol^−1^) and **TS2‐ent** (−24.0 kcal mol^−1^), respectively. Subsequently, the transmetallation of the alkyl‐Pd(II) species with Cu–Si species proceeds to give the Pd(II) intermediates **Int4**. Finally, reductive elimination of the resultant alkyl‐Pd(II) intermediates **Int4** and **Int4‐ent** leads to the (*R*)‐product and (*S*)‐product, with the transition states **TS3** (−40.7 kcal mol^−1^) and **TS3‐ent** (−40.2 kcal mol^−1^), respectively. Based on the calculation result, the enantio‐determining step proves to be the migratory insertion of the imino‐Pd(II) intermediate to the alkene. The two transition states differ in free energy of activation by 2.3 kcal mol^−1^ favoring the (*S*)‐product, aligning well with the experiments.

Further investigation of the calculated transition‐state structures **TS2** and **TS2‐ent** for migratory insertion was conducted via an independent gradient model (IGM) (Figure 2e).^[^ ^26^ ^]^ It revealed that the weak CH─*π* hydrogen bond exits in **TS2** between the aromatic ring of the substrate fragment and naphthyl group in the ligand, which may account for the stereoselectivity. This observation is consistent with the poor performance of the substrate bearing a methyl group (see Figure S1, Supporting Information for details). Taken together, these analyses suggest that the weak interactions between the substrate and ligand, particularly the CH···*π* interaction, probably dominate the stereoselectivity.^[^ ^27^ ^]^

## 3 Conclusion

In summary, we developed an efficient asymmetric aminosilylation reaction of alkenes via the tandem Narasaka–Heck process. It enables the construction of a broad array of pyrrolines bearing a quaternary or vicinal stereocenters in high yields with excellent enantioselectivities and diastereoselectivities. The success of this transformation relies on the development of a highly sophisticated ligand, which could effectively suppress the potentially SET pathway, but also ensure a high level of enantiocontrol. Mechanistic investigation and DFT study explain the origin of the enantioselectivity and reveal the weak interaction between the ligand and the substrate account for the stereoselectivity. Further studies toward the practical synthesis of other valuable chiral nitrogen‐containing heterocycle compounds are currently underway.

## 4 Experimental Section

### General Procedure for Synthesis of 3

Under nitrogen atmosphere, to an oven‐dried 10 mL Schlenk tube equipped with a magnetic stir was added Pd(dba)_2_ (8.5 mg, 0.015 mmol, 5 mol%), ligand **M7** (23.9 mg, 0.03 mmol, 10 mol%) and cyclopentane (3 mL). The catalyst/ligand solution was stirred for 1.0 h at 25 °C, CsOAc (230.3 mg, 1.20 mmol, 4.0 equiv), oxime esters **1** (0.30 mmol, 1.0 equiv), silylboronic ester **2** (0.75 mmol, 2.5 equiv) were added successively. The resulting mixture was then stirred vigorously at 0 °C for ≈40 h. After completion of the reaction (monitored by TLC), the reaction mixture was concentrated to dryness and the residue was purified by column chromatography (petroleum ether/ethyl acetate) to afford the desired product **3**.

The data that support the findings of this study are available within the paper and its Supplementary Information files. Crystallographic parameters for compounds **3an** is available free of charge from the Cambridge Crystallographic Data Centre under CCDC 2 324 487 (www.ccdc.cam.ac.uk/data_request/cif). All data are also available from authors upon request.

## Conflict of Interest

The authors declare no conflict of interest.

## Supporting information

Supporting Information

## Data Availability

The data that support the findings of this study are available in the supplementary material of this article.
