# Supplementary material for: Enantioselective Aminosilylation of Alkenes by Palladium/Ming‐Phos‐Catalyzed Tandem Narasaka–Heck/Silylation Reaction
Source: Adv Sci (Weinh). 2024 Jul 5;11(34):2403470. doi: 10.1002/advs.202403470 (PMC11425962; doi:10.1002/advs.202403470)
Supplement: Supplementary file 1 — Supporting Information [file ADVS-11-2403470-s001.pdf]

ADVANCED SCIENCE
Open Access

Supporting Information

for *Adv. Sci.*, DOI 10.1002/advs.202403470

Enantioselective Aminosilylation of Alkenes by Palladium/Ming-Phos-Catalyzed Tandem Narasaka–Heck/Silylation Reaction

Kangning Cao, Jie Han, Wenshao Ye, Dejun Hu, Zihao Ye, Junfeng Yang, Junliang Zhang* and Fener Chen**

Supporting Information

Enantioselective Aminosilylation of Alkenes by Palladium/Ming-Phos-Catalyzed Tandem Narasaka-Heck/Silylation Reaction

Kangning Cao, Jie Han, Wenshao Ye, Dejun Hu, Zihao Ye, Junfeng Yang,* Junliang Zhang, * Fener Chen*

Table of Contents

1. **General information……………………………………………………………S1-S2**
2. **Synthesis of the Ming-Phos and starting materials……………………………S3-S27**
3. **Optimization of the reaction conditions………………………………………..S28-S31**
4. **General experimental procedure and characterization of products…………S32-S59**
5. **Synthetic applications of the products and characterization of products……S60-S68**
6. **Determination of the absolute configurations …………………………………S69-S96**
7. **Mechanism studies…………………………………………………………………..S97-S98**
8. **DFT studies……………………………………………………………………………S99-S177**
9. **Copies of NMR spectra……………………………………………………………S178-S310**
10. **References…………………………………………………………..............S311-S312**

1. General information

General procedures and methods

Experiments involving moisture and/or air sensitive components were performed under a positive pressure of argon in oven-dried glassware equipped with a rubber septum inlet. Dried solvents and liquid reagents were transferred by oven-dried syringes or hypodermic syringe cooled to ambient temperature in a desiccator. Reactions mixtures were stirred in 10 mL sample vial with Teflon-coated magnetic stirring bars unless otherwise stated. Moisture in non-volatile reagents/compounds was removed in high *vacuo* by means of an oil pump and subsequent purging with nitrogen. Solvents were removed *in vacuo* under ~30 mmHg and heated with a water bath at 30–35 °C using rotary evaporator with aspirator. The condenser was cooled with running water at 0 °C.

All experiments were monitored by analytical thin layer chromatography (TLC). TLC was performed on pre-coated plates, 60 F_{254}. After elution, plate was visualized under UV illumination at 254 nm for UV active material. Further visualization was achieved by staining $Ce(SO_4)_2$ and phosphomolybdic acid solution. For those using the aqueous stains, the TLC plates were heated on a hot plate.

Columns for flash chromatography (FC) contained *silica gel* 200–300 mesh. Columns were packed as slurry of *silica gel* in petroleum ether and equilibrated solution using the appropriate solvent system. The elution was assisted by applying pressure of about 2 atm with an air pump.

Instrumentations

Proton nuclear magnetic resonance (^{1}H NMR) and carbon NMR (^{13}C NMR) were recorded in $CDCl_3$ otherwise stated. Chemical shifts are reported in parts per million (ppm), using the residual solvent signal as an internal standard: $CDCl_3$ (^{1}H NMR: δ 7.26, singlet; ^{13}C NMR: δ 77.0, triplet). Multiplicities were given as: *s* (singlet), *d* (doublet), *t* (triplet), *q* (quartet), *quintet*, *m* (multiplets), *dd* (doublet of doublets), *dt* (doublet of triplets), and *br* (broad). Coupling constants (*J*) were recorded in hertz (Hz). The number of proton atoms (*n*) for a given resonance was indicated by *n*H. The number of carbon atoms (*n*) for a given resonance was indicated by *n*C. HRMS (Analyzer: TOF) was reported in units of mass of charge ratio (m/z). Optical rotations were recorded on a polarimeter with a sodium lamp of wavelength 589 nm and

reported as follows; $[\alpha]_{\lambda}^{T°C}$ (c = g/100 mL, solvent). Melting points were determined on a melting point apparatus.

Enantiomeric excesses were determined by chiral High Performance Liquid Chromatography (HPLC) analysis. UV detection was monitored at 254 nm and 210 nm at the same time. HPLC samples were dissolved in HPLC grade isopropanol (IPA) unless otherwise stated.

Materials

All commercial reagents were purchased with the highest purity grade. They were used without further purification unless specified. All solvents used, mainly petroleum ether (PE) and ethyl acetate (EtOAc) were distilled. Anhydrous dichloromethane (DCM), CH_3CN were freshly distilled from CaH_2 and stored under N_2 atmosphere. THF, Et_2O, MTBE, 1,2-dimethoxyethane, *t*BuPh, mesitylene, CPME, and toluene were freshly distilled from sodium/benzophenone before use. All compounds synthesized were stored in a −20 ºC freezer and light-sensitive compounds were protected with aluminium foil. Unless otherwise noted, commercial reagents were purchased from Energy chemical, Bidepharm, Aesar, Leyan, Laajoo, and other commercial suppliers and were used as received.

2. Synthesis of the Ming-Phos and starting materials

2.1 Typical Procedure A: Synthesis of Ming-Phos M3-M8, M11-M14

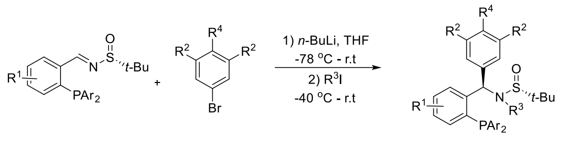

M3: R^1 = H, R^2 = *t*-Bu, R^3 = Me, Ar = 3,5-$(Me)_2C_6H_3$, R^4 = OMe
M4: R^1 = H, R^2 = 1-naphthyl, R^3 = Me, Ar = Ph, R^4 = H
M5: R^1 = H, R^2 = 1-naphthyl, R^3 = Et, Ar = 3,5-$(Me)_2C_6H_3$, R^4 = H
M6: R^1 = H, R^2 = 1-naphthyl, R^3 = Me, Ar = 3,5-$(Me)_2C_6H_3$, R^4 = H
M7: R^1 = H, R^2 = 1-naphthyl, R^3 = CD_3, Ar = 3,5-$(Me)_2C_6H_3$, R^4 = H
M8: R^1 = 3,4-benzodioxan, R^2 = 1-naphthyl, R^3 = Me, Ar = Ph, R^4 = H
M11: R^1 = 4-CF_3, R^2 = 1-naphthyl, R^3 = Me, Ar = Ph, R^4 = H
M12: R^1 = 3,4-dioxole, R^2 = 1-naphthyl, R^3 = Me, Ar = Ph, R^4 = H
M13: R^1 = H, R^2 = 1-naphthyl, R^3 = Me, Ar = 3,5-$(CF_3)_2C_6H_3$, R^4 = H
M14: R^1 = H, R^2 = 3,5-$(t\text{-}Bu)_2C_6H_3$, R^3 = Me, Ar = 3,5-$(Me)_2C_6H_3$, R^4 = H

Under nitrogen atmosphere, to an oven-dried 50 mL three-necked flask equipped with a magnetic stir bar was charged with aryl bromide (3.9 mmol, 1.3 equiv) and dry THF (5 mL). The mixture was cooled to -78 °C and *n*-BuLi (3.9 mmol, 1.3 equiv, 2.5 M in hexane) was added dropwise. After stirred at -78 °C for 1 h, the (*Rs*)-sulfinyl imine (3 mmol, 1.0 equiv), which was prepared according to the literature procedure,[1-2] in dry THF (5 mL) was added. The reaction mixture was kept at -78 °C and then slowly warmed to room temperature. Upon the (*Rs*)-sulfinyl imine disappeared, the system was re-cooled to -40 °C, and R^3I (3.9 mmol, 1.3 equiv) was added. Then, the refrigeration program was turned off to allow the system to warm up. Once reaction completed, the reaction mixture was quenched by the addition of saturated NH_4Cl solution (10 mL) and extracted by EtOAc (10 mL×3). The combined organic layers were washed by brine, dried over Na_2SO_4 and concentrated to dryness. The residue was purified by column chromatography (petroleum ether/ethyl acetate = 15:1 to 5:1) to afford **M3-M8, M11-M14**.

(*R*)-*N*-((*R*)-(2-(Bis(3,5-dimethylphenyl)phosphaneyl)phenyl)(3,5-di-*tert*-butyl-4-methoxyphenyl)methyl)-*N*,2-dimethylpropane-2-sulfinamide (**M3**)

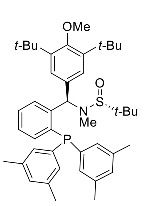

Following **Typical Procedure A**, isolated **M3** 868.8 mg, 33% yield for two steps, as white solid, m.p. 137.3-137.9 °C, **^{1}H NMR** (400 MHz, $CDCl_3$) δ 7.50 (m, *J* = 7.8, 4.5, 1.4 Hz, 1H), 7.35 (m, *J* = 7.6, 1.4 Hz, 1H), 7.17 (m, *J* = 7.5, 1.4 Hz, 1H), 7.07 – 6.97 (m, 3H), 6.93 (d, *J* = 3.4 Hz, 1H), 6.85 – 6.75 (m, 3H), 6.70 – 6.63 (m, 2H), 3.54 (s, 3H), 2.67 (s, 3H), 2.24 (s, 6H), 2.14 (s, 6H), 1.27 (s, 18H), 1.12 (s, 9H); **^{13}C NMR** (100 MHz, $CDCl_3$, This compound contains many sp^2-carbons, it's difficult to recognize the C-P couplings. So we list all the signals here.) δ 158.2, 146.7, 146.4, 142.8, 137.7, 137.7, 137.5, 137.4, 137.3, 134.8, 133.1, 131.6, 131.4, 131.2, 130.4, 130.1, 128.7, 128.5, 128.2, 127.3,

64.0, 58.5, 35.6, 32.0, 24.0, 21.3, 21.3; **^{31}P NMR** (162 MHz, $CDCl_3$) δ -17.71. HRMS (ESI) m/z calcd. for $C_{43}H_{58}NNaO_2PS$ $[M+Na]^+$ = 706.3818, found = 706.3805.

(*R*)-*N*-((*R*)-(3,5-Di(naphthalen-1-yl)phenyl)(2-(diphenylphosphanyl)phenyl)methyl)-*N*,2-dimethylpropane-2-sulfinamide (M4)

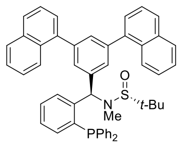

Following **Typical Procedure A**, isolated **M4** 1.087 g, 49% yield for two steps, as white solid, m.p. 94.1-94.7 °C, $[\alpha]_D^{20}$ = +21.7 (*c* = 0.40, $CHCl_3$). **^{1}H NMR** (400 MHz, $CDCl_3$) δ 7.92 – 7.82 (m, 6H), 7.74 (dd, *J* = 7.3, 3.9 Hz, 1H), 7.53 – 7.46 (m, 4H), 7.40 (s, 6H), 7.37 – 7.28 (m, 5H), 7.21 (t, *J* = 5.4 Hz, 3H), 7.14 (d, *J* = 3.9 Hz, 1H), 7.12 – 7.03 (m, 5H), 6.81 (s, 1H), 2.81 (s, 3H), 1.19 (s, 9H); **^{13}C NMR** (100 MHz, $CDCl_3$, This compound contains many sp^2-carbons, it's difficult to recognize the C-P couplings. So we list all the signals here.) δ 146.4, 146.1, 140.4, 139.7, 139.5, 137.5, 137.4, 136.2, 136.0, 135.8, 135.7, 135.2, 133.8, 133.7, 133.6, 133.5, 131.4, 131.0, 130.6, 129.3, 128.5, 128.4, 128.3, 128.2, 128.1, 127.6, 127.1, 126.0, 125.9, 125.7, 125.3, 58.7, 24.0; **^{31}P NMR** (162 MHz, $CDCl_3$) δ -18.02. HRMS (ESI) m/z calcd. for $C_{50}H_{44}NONaPS$ $[M+Na]^+$ = 760.2773, found = 760.2794.

(*R*)-*N*-((*R*)-(2-(Bis(3,5-dimethylphenyl)phosphanyl)phenyl)(3,5-di(naphthalen-1-yl)phenyl)methyl)-N-ethyl-2-methylpropane-2-sulfinamide (**M5**)

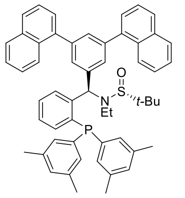

Following **Typical Procedure A**, isolated **M5** 387.5 mg, 16% yield for two steps, as white solid, m.p. 115-116 °C. $[\alpha]_D^{20}$ = -4.7 (*c* = 0.50, $CHCl_3$). **^{1}H NMR** (400 MHz, $CDCl_3$) δ 7.85 (dd, *J* = 21.5, 8.3 Hz, 6H), 7.59 (dd, *J* = 7.0, 4.9 Hz, 1H), 7.55 – 7.29 (m, 12H), 7.22 (t, *J* = 7.4 Hz, 1H), 7.10 (s, 1H), 6.93 (s, 2H), 6.75 (dd, *J* = 14.6, 8.0 Hz, 4H), 6.62 (s, 1H), 3.57 – 3.48 (m, 1H), 3.23 – 3.07 (m, 1H), 2.24 (s, 6H), 1.88 (s, 6H), 1.28 (t, *J* = 7.1 Hz, 3H), 1.12 (s, 9H); **^{13}C NMR** (100 MHz, $CDCl_3$, This compound contains many sp^2-carbons, it's difficult to recognize the C-P couplings. So we list all the signals here.) δ 140.6, 140.3, 139.8, 137.7, 137.6, 137.5, 137.4, 135.4, 135.3, 134.9, 133.8, 131.8, 131.6, 131.5, 131.3, 131.1, 130.7, 130.4, 130.1, 128.5, 128.2, 127.5, 127.4, 127.1, 125.9, 125.6, 125.3, 58.2, 23.6, 21.3, 21.0, 14.8; **^{31}P NMR** (162 MHz, $CDCl_3$) δ -17.21. HRMS (ESI) m/z calcd. for $C_{55}H_{54}NONaPS$ $[M+Na]^+$ = 830.3556, found = 830.3580.

(*R*)-*N*-((*R*)-(2-(Bis(3,5-dimethylphenyl)phosphanyl)phenyl)(3,5-di(naphthalen-1-yl)phenyl)methyl)-*N*,2-dimethylpropane-2-sulfinamide (M6)

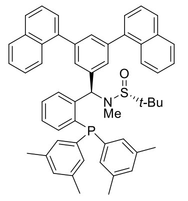

Following **Typical Procedure A**, isolated **M6** 1.238 g, 52% yield for two steps, as white solid, m.p. 110-111 ºC. $[\alpha]_D^{20}$ = +28.9 (*c* = 0.50, $CHCl_3$). **^{1}H NMR** (400 MHz, $CDCl_3$) δ 8.02 (d, *J* = 8.3 Hz, 2H), 7.91 (dd, *J* = 19.2, 8.1 Hz, 4H), 7.78 (dd, *J* = 7.1, 4.3 Hz, 1H), 7.57 – 7.43 (m, 12H), 7.29 (d, *J* = 7.5 Hz, 1H), 7.13 (dd, *J* = 6.5, 3.9 Hz, 1H), 7.00 (s, 1H), 6.93 (d, *J* = 7.8 Hz, 2H), 6.80 – 6.71 (m, 4H), 2.87 (s, 3H), 2.30 (s, 6H), 2.00 (s, 6H), 1.24 (s, 9H); **^{13}C NMR** (100 MHz, $CDCl_3$, This compound contains many sp^2-carbons, it's difficult to recognize the C-P couplings. So we list all the signals here.) δ 145.7, 145.8, 145.6, 141.8, 141.6, 140.3, 139.7, 137.8, 137.7, 137.6, 137.5, 137.0, 136.8, 135.5, 135.4, 134.8, 133.8, 131.7, 131.6, 131.5, 131.4, 130.7, 130.6, 130.5, 130.3, 128.8, 128.5, 128.4, 128.2, 127.6, 127.5, 127.1, 126.0, 125.7, 125.3, 58.7, 24.0, 21.3, 21.1; **^{31}P NMR** (162 MHz, $CDCl_3$) δ -16.61. HRMS (ESI) m/z calcd. for $C_{54}H_{52}NNaOPS$ $[M+Na]^+$ = 816.3399, found = 816.3417.

(*R*)-*N*-((*R*)-(2-(Bis(3,5-dimethylphenyl)phosphanyl)phenyl)(3,5-di(naphthalen-1-yl)phenyl)methyl)-2-methyl-N-(methyl-d3)propane-2-sulfinamide (**M7**)

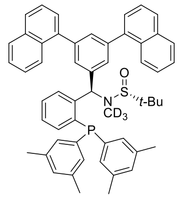

Following **Typical Procedure A**, isolated **M7** 1.218 g, 51% yield for two steps, as white solid, m.p. 104.1-104.9 ºC. $[\alpha]_D^{20}$ = +30.8 (*c* = 0.50, $CHCl_3$). **^{1}H NMR** (400 MHz, $CDCl_3$) δ 7.91 (dd, *J* = 16.3, 8.3 Hz, 4H), 7.84 (d, *J* = 8.2 Hz, 2H), 7.70 (dd, *J* = 7.5, 4.4 Hz, 1H), 7.49 (dd, *J* = 16.1, 8.6 Hz, 5H), 7.38 (dd, *J* = 16.2, 8.7 Hz, 7H), 7.22 (t, *J* = 7.4 Hz, 1H), 7.05 (dd, *J* = 7.2, 4.0 Hz, 1H), 6.95 (s, 1H), 6.84 (d, *J* = 7.9 Hz, 2H), 6.76 – 6.67 (m, 3H), 6.63 (d, *J* = 6.8 Hz, 1H), 2.24 (s, 6H), 1.94 (s, 6H), 1.17 (s, 9H); **^{13}C NMR** (100 MHz, $CDCl_3$, This compound contains many sp^2-carbons, it's difficult to recognize the C-P couplings. So we list all the signals here.) δ 145.8, 145.6, 140.3, 139.7, 137.8, 137.7, 137.6, 137.5, 137.0, 136.9, 136.8, 135.5, 135.4, 134.8, 133.8, 131.7, 131.6, 131.5, 131.4, 130.7, 130.6, 130.5, 130.3, 128.8, 128.5, 128.4, 128.2, 127.6, 127.5, 127.1, 126.0, 125.7, 125.3, 58.6, 24.0, 21.3, 21.0; **^{31}P NMR** (162 MHz, $CDCl_3$) δ -16.70. HRMS (ESI) m/z calcd. for $C_{54}H_{49}D_3NONaPS$ $[M+Na]^+$ = 819.3588, found = 819.3609.

(*R*)-*N*-((*R*)-(3,5-Di(naphthalen-1-yl)phenyl)(7-(diphenylphosphanyl)-2,3-dihydrobenzo[*b*][1,4]dioxin-6-yl)methyl)-*N*,2-dimethylpropane-2-sulfinamide (**M8**)

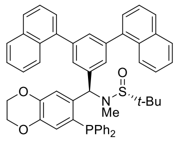

Following **Typical Procedure A**, isolated **M8** 1.026 g, 43% yield for two steps, as white solid, m.p. 130-131 ºC. $[\alpha]_D^{20}$ = -21.1 (*c* = 0.50, $CHCl_3$). **^{1}H NMR** (400 MHz, $CDCl_3$) δ 7.89 (d, *J* = 8.6 Hz, 4H), 7.85 (d, *J* = 8.2 Hz, 2H), 7.53 – 7.46 (m, 5H), 7.43 – 7.39 (m, 5H), 7.34 (d, *J* = 7.0 Hz, 2H), 7.30 (d, *J* = 5.0 Hz, 3H), 7.26 – 7.20 (m, 2H), 7.15 (d, *J* = 6.9 Hz, 1H), 7.12 – 7.02 (m, 4H), 6.71 (d, *J* = 7.3 Hz, 1H), 6.56 (d, *J* = 3.6 Hz, 1H), 4.24 – 4.18 (m, 4H), 2.79 (s, 3H), 1.21 (s, 9H); **^{13}C NMR** (100 MHz, $CDCl_3$, This compound contains many sp^2-carbons, it's difficult to recognize the C-P couplings. So we list all the signals here.) δ 144.6, 142.8, 140.3, 140.0, 139.8, 139.7, 137.8, 137.7, 136.3, 136.2, 133.7, 133.6, 133.5, 133.4, 133.3, 131.5, 130.9, 130.6, 128.5, 128.4, 128.3, 128.2, 128.1, 127.6, 127.1, 126.1, 126.0, 125.7, 125.3, 123.9, 117.1, 117.0, 64.4, 64.1, 58.6, 24.0; **^{31}P NMR** (162 MHz, $CDCl_3$) δ -19.46. HRMS (ESI) m/z calcd. for $C_{52}H_{46}NO_3NaPS$ $[M+Na]^+$ = 818.2828, found = 818.2849.

(*R*)-N-((*R*)-(3,5-Di(naphthalen-1-yl)phenyl)(2-(diphenylphosphanyl)-5-(trifluoromethyl)phenyl)methyl)-*N*,2-dimethylpropane-2-sulfinamide (**M11**)

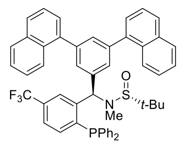

Following **Typical Procedure A**, isolated **M11** 700.6 mg, 29% yield for two steps, as white solid, m.p. 106-107 ºC. $[\alpha]_D^{20}$ = +8.7 (*c* = 0.50, $CHCl_3$). **^{1}H NMR** (400 MHz, $CDCl_3$) δ 8.01 (d, *J* = 1.9 Hz, 1H), 7.91 – 7.85 (m, 6H), 7.55 – 7.41 (m, 8H), 7.35 – 7.33 (m, 7H), 7.21 – 7.15 (m, 4H), 7.14 – 7.05 (m, 4H), 6.82 (d, *J* = 6.3 Hz, 1H), 2.82 (s, 3H), 1.18 (s, 9H); **^{13}C NMR** (100 MHz, $CDCl_3$, This compound contains many sp^2-carbons, it's difficult to recognize the C-P or C-F couplings. So we list all the signals here.) δ 147.3, 147.1, 141.6, 141.5, 140.7, 139.4, 138.6, 136.3, 136.2, 135.6, 134.6, 134.5, 133.9, 133.8, 133.7, 133.6, 133.5, 131.4, 131.0, 130.9, 129.0, 128.9, 128.7, 128.6, 128.5, 128.4, 128.3, 127.8, 127.1, 126.1, 125.8, 125.7, 125.3, 124.8, 124.7, 124.6, 124.5, 124.2, 124.1, 124.0, 123.9, 58.8, 23.8; **^{31}P NMR** (162 MHz, $CDCl_3$) δ -18.03; **^{19}F NMR** (376 MHz, $CDCl_3$) δ -62.78. HRMS (ESI) m/z calcd. for $C_{51}H_{43}F_3NONaPS$ $[M+Na]^+$ = 828.2647, found = 828.2668.

(*R*)-*N*-((*R*)-(3,5-Di(naphthalen-1-yl)phenyl)(6-(diphenylphosphanyl)benzo[d][1,3]dioxol-5-yl)methyl)-*N*,2-dimethylpropane-2-sulfinamide (**M12**)

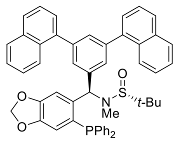

Following **Typical Procedure A**, isolated **M12** 937.5 mg, 40% yield for two steps, as white solid, m.p. 135.4-135.9 ºC. $[\alpha]_D^{20}$ = -25.3 (*c* = 0.50, $CHCl_3$). **^{1}H NMR** (400 MHz, $CDCl_3$) δ 7.94 – 7.82 (m, 6H), 7.53 – 7.47 (m, 4H), 7.42 –

7.40 (m, 5H), 7.37 – 7.29 (m, 5H), 7.26 – 7.00 (m, 8H), 6.88 (d, *J* = 8.5 Hz, 1H), 6.54 (s, 1H), 5.94 (d, *J* = 13.3 Hz, 2H), 2.81 (s, 3H), 1.23 (s, 9H); ^{13}**C NMR** (100 MHz, $CDCl_3$, This compound contains many sp^2-carbons, it's difficult to recognize the C-P couplings. So we list all the signals here.) δ 149.0, 147.0, 141.7, 141.5, 140.4, 139.8, 139.7, 137.8, 137.7, 136.2, 136.1, 133.7, 133.4, 133.3, 133.2, 133.1, 131.4, 130.7, 130.6, 128.5, 128.4, 128.3, 128.2, 128.1, 127.7, 127.1, 126.0, 125.7, 125.3, 114.3, 108.5, 108.4, 101.2, 58.6, 24.0; ^{31}**P NMR** (162 MHz, $CDCl_3$) δ -18.01. HRMS (ESI) m/z calcd. for $C_{51}H_{44}NO_3NaPS$ $[M+Na]^+$ = 804.2672, found = 804.2691.

(*R*)-*N*-((*R*)-(2-(Bis(3,5-bis(trifluoromethyl)phenyl)phosphanyl)phenyl)(3,5-di(naphthalen-1-yl)phenyl)methyl)-*N*,2-dimethylpropane-2-sulfinamide (**M13**)

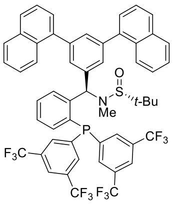

Following **Typical Procedure A**, isolated **M13** 696.4 mg, 23% yield for two steps, as white solid, m.p. 99-100 ºC. $[\alpha]^{20}_{D}$ = -12.4 (*c* = 0.50, $CHCl_3$). ^{1}**H NMR** (400 MHz, $CDCl_3$) δ 7.91 – 7.80 (m, 8H), 7.71 (s, 1H), 7.62 – 7.44 (m, 11H), 7.43 – 7.34 (m, 5H), 7.24 (s, 1H), 7.04 (dd, *J* = 7.1, 4.4 Hz, 1H), 6.83 (d, *J* = 7.1 Hz, 1H), 2.82 (s, 3H), 1.20 (s, 9H); ^{13}**C NMR** (100 MHz, $CDCl_3$, This compound contains many sp^2-carbons, it's difficult to recognize the C-P or C-F couplings. So we list all the signals here.) δ 147.8, 147.6, 140.9, 140.4, 140.2, 139.3, 139.0, 138.6, 138.4, 135.4, 135.3, 133.8, 133.24 – 131.62 (m), 131.5, 131.3, 131.2, 130.9, 128.8, 128.7, 128.6, 128.4, 128.0, 127.0, 126.1, 125.8, 125.4, 125.3, 124.2, 124.0, 123.70 – 123.25 (m), 123.21 – 122.80 (m), 121.5, 121.3, 58.8, 23.7; ^{31}**P NMR** (162 MHz, $CDCl_3$) δ -17.29; ^{19}**F NMR** (376 MHz, $CDCl_3$) δ -63.04, -63.09. HRMS (ESI) m/z calcd. for $C_{54}H_{40}F_{12}NONaPS$ $[M+Na]^+$ = 1032.2269, found = 1032.2283.

(*R*)-*N*-((*R*)-(2-(Bis(3,5-dimethylphenyl)phosphanyl)phenyl)(3,3'',5,5''-tetra-*tert*-butyl-[1,1':3',1''-terphenyl]-5'-yl)methyl)-*N*,2-dimethylpropane-2-sulfinamide (**M14**)

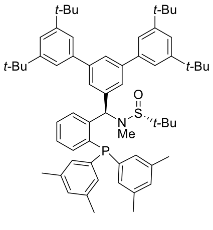

Following **Typical Procedure A**, isolated **M14** 868.8 mg, 33% yield for two steps, as white solid, m.p. 100.5-100.9 ºC. $[\alpha]^{20}_{D}$ = +10.5 (*c* = 0.50, $CHCl_3$). ^{1}**H NMR** (400 MHz, $CDCl_3$) δ 7.80 (dd, *J* = 7.6, 4.3 Hz, 1H), 7.58 (s, 1H), 7.45 – 7.34 (m, 9H), 7.20 (t, *J* = 7.5 Hz, 1H), 7.04 (dd, *J* = 7.4, 4.0 Hz, 1H), 6.96 (s, 1H), 6.90 (d, *J* = 8.0 Hz, 2H), 6.69 (s, 1H), 6.57 – 6.54 (m, 3H), 2.66 (s, 3H), 2.26 (s, 6H), 1.95 (s, 6H), 1.38 (s, 36H), 1.22 (s, 9H); ^{13}**C NMR** (100 MHz, $CDCl_3$, This compound contains many sp^2-carbons, it's difficult to recognize the C-P couplings. So we list

all the signals here.) δ 150.9, 146.1, 145.9, 142.3, 140.5, 140.0, 137.8, 137.7, 137.3, 137.2, 136.9, 136.7, 134.8, 131.7, 131.5, 131.4, 131.2, 130.3, 130.2, 128.9, 128.3, 127.6, 127.5, 127.4, 125.3, 121.6, 121.4, 58.7, 34.9, 31.5, 24.1, 21.3, 21.1; **^{31}P NMR** (162 MHz, $CDCl_3$) δ -16.90. HRMS (ESI) m/z calcd. for $C_{59}H_{76}NONaPS$ $[M+Na]^+$ = 940.5591, found = 940.5623.

2.2 Typical Procedure B: Synthesis of starting materials

The substrates **1a-1-1a-7**, **1a-1z** were synthesized according to **Step 1**.

The substrates **1ad-1an** were synthesized according to **Step 2-Step 4**.

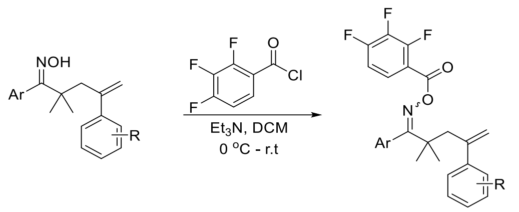

Step 1: Synthesis of SI-2. The substrates **SI-2** were synthesized according to published procedures.[3] In a nitrogen-filled glove box, to a solution of oxime (1.0 equiv.) in anhydrous DCM (0.5 M) at 0 °C was added Et_3N (2.0 equiv.) and stirred for 10 min. 2,3,4-trifluorobenzoyl chloride (1.2 equiv.) was dropwise added via a syringe at 0 ºC. After stirring at room temperature for 2-12 h, the reaction was quenched with saturated aqueous solution of $NaHCO_3$ and diluted with CH_2Cl_2. The mixture solution was washed with water, dried over anhydrous Na_2SO_4, filtered, and concentrated in vacuum. The residue was purified by column chromatography on silica gel to afford the corresponding oxime esters.

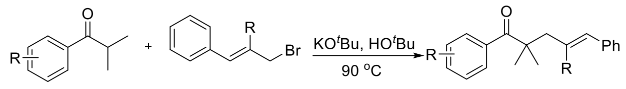

Step 2: Synthesis of SI-5. The spectral data of the substrates **SI-4** were consisted with that reported in the literature.[4] The substrates **SI-3** were synthesized according to published procedures.[3] Under air conditions, to a solution of **SI-3** (1.0 equiv.) in anhydrous HO^{t}Bu (3.0 mL/mmol) was added KO^{t}Bu (5.0 equiv.) and the mixture was stirred at room temperature for 5 minutes. Then, **SI-4** (1.5 equiv.) was added via syringe and the mixture was heated at 90 ºC for 12 hours. The mixture was cooled to room temperature and H_2O was added. The mixture was extracted with EtOAc. The organic extracts were combined, washed with brine, dried with Na_2SO_4 and concentrated in vacuum. The residue was used for the next step without purification.

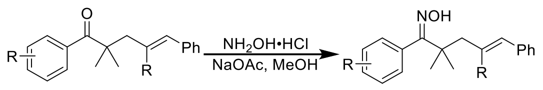

SI-6 **SI-7**

Step 3: Synthesis of SI-7. The substrates **SI-7** were synthesized according to published procedures.[3] Under air conditions, $H_2NOH{\cdot}HCl$ (4 equiv.) and NaOAc (7 equiv.) were added to a solution of **SI-6** (1.0 equiv.) in MeOH (2.0 mL/mmol) in a round-bottomed flask which was fitted with a reflux condenser. The mixture was heated at 80 ºC until consumption of starting material was observed by TLC. After cooling to room temperature, the mixture was diluted with EtOAc, washed with brine, dried with Na_2SO_4 and concentrated in vacuum to give **SI-7**, which was used for the next step without further purification.

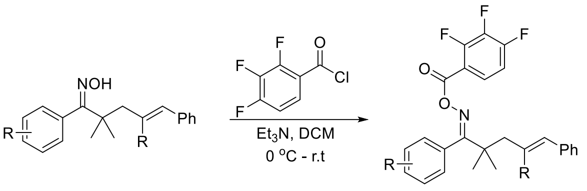

SI-8 **SI-9**

Step 4: Synthesis of SI-9. The substrates **SI-8** were synthesized according to published procedures.[3] In a nitrogen-filled glove box, to a solution of **SI-8** (1.0 equiv.) in anhydrous DCM (2.0 mL/mmol) at 0 ºC was added Et_3N (2.0 equiv.) followed by $C_6F_3H_2COCl$ (1.2 equiv.) dropwise via syringe. The mixture was then warmed to room temperature and stirred until the reaction was complete as observed by TLC. The mixture was quenched with water, diluted with DCM, washed with brine, dried with Na_2SO_4 and concentrated in vacuum. The residue was purified by column chromatograph to afford substrate **SI-9**.

2,2-Dimethyl-1,4-diphenylpent-4-en-1-one *O*-benzoyl oxime (1a-1)

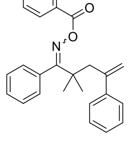

The reaction was performed following **Typical Procedure B** with **SI-1 (**1 mmol**)** to give the product **1a-1** (333.3 mg, 87% yield) as white solid, m.p. 77.1-77.9 ºC, **^{1}H NMR** (400 MHz, $CDCl_3$) δ 7.59 (d, *J* = 7.6 Hz, 2H), 7.53 – 7.41 (m, 6H), 7.37 – 7.27 (m, 5H), 7.17 – 7.07 (m, 2H), 5.49 – 5.23 (m, 2H), 3.04 (s, 2H), 1.22 (s, 6H); **^{13}C NMR** (100 MHz) δ 175.1, 163.6, 145.7, 143.2, 133.4, 132.9, 129.3, 129.1, 128.3, 128.2, 128.0, 127.2, 126.7, 126.6, 118.1, 44.4, 41.9, 26.2. HRMS (ESI) m/z calcd. for $C_{26}H_{25}NNaO_2$ $[M+Na]^+$ = 406.1777, found = 406.1781.

2,2-Dimethyl-1,4-diphenylpent-4-en-1-one *O*-(2,3-difluorobenzoyl) oxime (1a-3)

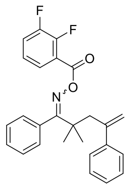

The reaction was performed following **Typical Procedure B** with **SI-1** (1 mmol) to give the product **1a-3** (335.3 mg, 80% yield) as white solid, m.p. 58.3-58.9 °C, **^{1}H NMR** (400 MHz, $CDCl_3$) δ 7.46 – 7.40 (m, 5H), 7.38 – 7.26 (m, 5H), 7.14 – 7.08 (m, 2H), 7.06 – 7.01 (m, 1H), 5.50 – 5.17 (m, 2H), 3.02 (s, 2H), 1.21 (s, 6H); **^{13}C NMR** (100 MHz, $CDCl_3$, This compound contains many sp^2-carbons, it's difficult to recognize the C-F couplings. So we list all the signals here.) δ 178.0, 160.9, 152.3 – 152.1 (m), 149.8 – 149.6 (m), 148.9 – 148.6 (m), 145.6, 143.2, 133.0, 128.3, 128.2, 127.9, 127.2, 126.6, 126.5, 126.4, 126.3, 123.8 – 123.6 (m), 122.1, 121.4, 121.2, 118.1, 44.4, 42.1, 26.2; **^{19}F NMR** (376 MHz, $CDCl_3$) δ -134.1 – -134.3 (m, 1F), -136.5 – -136.6 (m, 1F). HRMS (ESI) m/z calcd. for $C_{26}H_{23}F_2NNaO_2$ [M+Na]$^+$ = 442.1589, found = 442.1596.

2,2-Dimethyl-1,4-diphenylpent-4-en-1-one *O*-(2,4-difluorobenzoyl) oxime (1a-4)

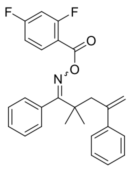

The reaction was performed following **Typical Procedure B** with **SI-1** (1 mmol) to give the product **1a-4** (347.9 mg, 83% yield) as white solid, m.p. 51.1-51.8 °C, **^{1}H NMR** (400 MHz, $CDCl_3$) δ 7.60 – 7.54 (m, 1H), 7.48 – 7.40 (m, 5H), 7.37 – 7.27 (m, 3H), 7.13 – 7.07 (m, 2H), 6.87 – 6.73 (m, 2H), 5.49 – 5.26 (m, 2H), 3.01 (s, 2H), 1.21 (s, 6H); **^{13}C NMR** (100 MHz, $CDCl_3$, This compound contains many sp^2-carbons, it's difficult to recognize the C-F couplings. So we list all the signals here.) δ 175.7, 145.6, 143.2, 135.0, 134.9, 135.0 – 134.8 (m), 133.6 – 133.5 (m), 133.3, 128.2, 127.9, 127.2, 126.6, 118.1, 112.6 – 112.3 (m), 111.6 - 111.4 (m), 106. 0 – 104.9 (m), 105.7, 105.4, 105.1, 104.9, 44.4, 42.1, 26.2; **^{19}F NMR** (376 MHz, $CDCl_3$) δ -197.9 – -198.0 (m, 1F), -101.7 – -102.0 (m, 1F), -102.8 – -102.9 (m, 1F). HRMS (ESI) m/z calcd. for $C_{26}H_{23}F_2NNaO_2$ [M+Na]$^+$ = 442.1589, found = 442.1597.

2,2-Dimethyl-1,4-diphenylpent-4-en-1-one *O*-(2,4,5-trifluorobenzoyl) oxime (1a-6)

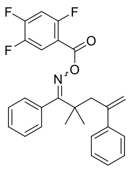

The reaction was performed following **Typical Procedure B** with **SI-1 (**1 mmol**)** to give the product **1a-6** (371.6 mg, 85% yield) as white solid, m.p. 67.1-67.9 °C, **^{1}H NMR** (400 MHz, $CDCl_3$) δ 7.51 – 7.39 (m, 5H), 7.36 – 7.28 (m, 4H), 7.09 – 7.07 (m, 2H), 6.92 – 6.86 (m, 1H), 5.50 – 5.24 (m, 2H), 3.00 (s, 2H), 1.21 (s, 6H); **^{13}C NMR** (100 MHz, $CDCl_3$, This compound contains many sp^2-carbons, it's difficult to recognize the C-F couplings. So we list all the signals here.) δ 176.1, 145.6, 143.1, 133.1, 128.4, 128.2, 128.0, 127.3, 126.6, 126.5, 119.7, 119.5, 118.1, 107.2, 107.0, 106.9, 106.7, 44.4, 42.1,

26.2; **^{19}F NMR** (376 MHz, $CDCl_3$) δ -108.4 – -108.5 (m, 1F), -124.9 – -125.0 (m, 1F), -141.2 – -141.4 (m, 1F). HRMS (ESI) m/z calcd. for $C_{26}H_{22}F_3NNaO_2$ $[M+Na]^+$ = 460.1495, found = 460.1503.

2,2-Dimethyl-1,4-diphenylpent-4-en-1-one *O*-(2,4,6-trifluorobenzoyl) oxime (1a-7)

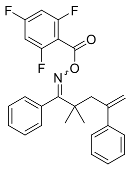

The reaction was performed following **Typical Procedure B** with **SI-1 (**1 mmol**)** to give the product **1a-7** (341.0 mg, 78% yield) as white solid, m.p. 69.9-70.2 ºC, **^{1}H NMR** (400 MHz, $CDCl_3$) δ 7.45 – 7.37 (m, 5H), 7.36 – 7.27 (m, 3H), 7.09 – 7.02 (m, 2H), 6.69 – 6.55 (m, 2H), 5.48 – 5.17 (m, 2H), 2.98 (s, 2H), 1.18 (s, 6H); **^{13}C NMR** (100 MHz, $CDCl_3$, This compound contains many sp^2-carbons, it's difficult to recognize the C-F couplings. So we list all the signals here.) δ 176.0, 165.9 – 165.2 (m), 163.3 – 162.9 (m), 160.4 – 160.0 (m), 158.4 – 158.3 (m), 145.6, 143.1, 132.8, 128.3, 128.2, 127.8, 127.2, 126.6, 126.5, 118.1, 101.2 – 100.6 (m), 44.4, 42.2, 26.2; **^{19}F NMR** (376 MHz, $CDCl_3$) δ -101.7 – -101.8 (m, 1F), -105.3 – -105.4 (m, 1F). HRMS (ESI) m/z calcd. for $C_{26}H_{22}F_3NNaO_2$ $[M+Na]^+$ = 460.1495, found = 460.1502.

2,2-Dimethyl-1,4-diphenylpent-4-en-1-one *O*-(2,3,4-trifluorobenzoyl) oxime (1a)

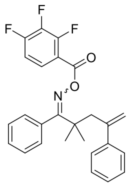

The reaction was performed following **Typical Procedure B** with **SI-1 (**10 mmol**)** to give the product **1a** (3.76 g, 86% yield) as white solid, m.p. 49-50 ºC, **^{1}H NMR** (400 MHz, $CDCl_3$) δ 7.47 – 7.40 (m, 5H), 7.38 – 7.27 (m, 4H), 7.09 – 7.07 (m, 2H), 6.97 – 6.90 (m, 1H), 5.51 – 5.24 (m, 2H), 3.01 (s, 2H), 1.21 (s, 6H); **^{13}C NMR** (100 MHz, $CDCl_3$, This compound contains many sp^2-carbons, it's difficult to recognize the C-F couplings. So we list all the signals here.) δ 176.1, 145.6, 143.2, 133.1, 128.4, 128.2, 128.0, 127.2, 126.6, 126.5, 126.0 – 125.9 (m), 118.1, 112.1, 112.1, 112.0, 112.0, 44.4, 42.1, 26.2; **^{19}F NMR** (376 MHz, $CDCl_3$) δ -126.0 – -126.1 (m, 1F), -128.6 – -128.7 (m, 1F), -158.5 – -158.7 (m, 1F). HRMS (ESI) m/z calcd. for $C_{26}H_{22}F_3NNaO_2$ $[M+Na]^+$ = 460.1495, found = 460.1508.

2,2-Dimethyl-4-phenyl-1-(*p*-tolyl)pent-4-en-1-one *O*-(2,3,4-trifluorobenzoyl) oxime (1b)

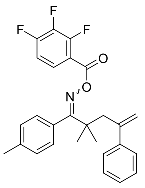

The reaction was performed following **Typical Procedure B** with **SI-1 (**1.6 mmol**)** to give the product **1b** (685.8 mg, 95% yield) as white solid, m.p. 63.3-63.9 ºC, **^{1}H NMR** (400 MHz, $CDCl_3$) δ 7.41 – 7.33 (m, 3H), 7.32 – 7.27 (m, 2H), 7.26 – 7.22 (m, 1H), 7.20 (d, J = 7.8 Hz, 2H), 6.97 – 6.86 (m, 3H), 5.47 – 5.19 (m, 2H),

3.02 – 2.89 (m, 2H), 2.38 (s, 3H), 1.16 (s, 6H); **^{13}C NMR** (100 MHz, $CDCl_3$, This compound contains many sp^2-carbons, it's difficult to recognize the C-F couplings. So we list all the signals here.) δ 176.3, 160.3 – 160.2 (m), 155.4 – 155.2 (m), 152.7 – 152.6 (m), 145.6, 143.2, 141.5 – 141.4 (m), 138.2, 130.0, 128.6, 128.2, 127.2, 126.6, 126.4, 126.0 – 125.9 (m), 118.0, 112.1, 111.9, 44.4, 42.2, 26.2, 21.2; **^{19}F NMR** (376 MHz, $CDCl_3$) δ -126.1 – -126.2 (m, 1F), -128.6 – -128.7 (m, 1F), -158.6 – -158.7 (m, 1F). HRMS (ESI) m/z calcd. for $C_{27}H_{24}F_3NNaO_2$ $[M+Na]^+$ = 474.1651, found = 474.1657.

1-(4-(*tert*-Butyl)phenyl)-2,2-dimethyl-4-phenylpent-4-en-1-one *O*-(2,3,4-trifluorobenzoyl) oxime (1c)

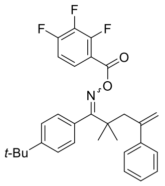

The reaction was performed following **Typical Procedure B** with **SI-1 (**1.9 mmol**)** to give the product **1c** (805.9 mg, 86% yield) as white solid, m.p. 52.2-52.8 ºC, **^{1}H NMR** (400 MHz, $CDCl_3$) δ 7.45 – 7.42 (m, 4H), 7.37 – 7.27 (m, 4H), 7.01 (d, J = 8.1 Hz, 2H), 6.89 (m, 1H), 5.48 – 5.24 (m, 2H), 3.01 (s, 2H), 1.39 (s, 9H), 1.22 (s, 6H); **^{13}C NMR** (100 MHz, $CDCl_3$, This compound contains many sp^2-carbons, it's difficult to recognize the C-F couplings. So we list all the signals here.) δ 176.5, 160.2 – 160.0 (m), 153.0 – 152.6 (m), 151.4, 145.6, 141.6, 139.0, 130.1, 128.2, 127.2, 126.6, 126.3, 126.0, 125.9, 124.8, 118.0, 112.0, 111.8, 44.3, 42.1, 34.7, 31.2, 26.1; **^{19}F NMR** (376 MHz, $CDCl_3$) δ -126.2 – -126.3 (m, 1F), -128.5 – -128.6 (m, 1F), -158.9 – -159.0 (m, 1F). HRMS (ESI) m/z calcd. for $C_{30}H_{30}F_3NNaO_2$ $[M+Na]^+$ = 516.2121, found = 516.2134.

2,2-Dimethyl-1-(4-phenoxyphenyl)-4-phenylpent-4-en-1-one *O*-(2,3,4-trifluorobenzoyl) oxime (1d)

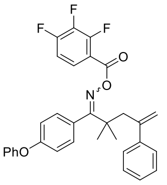

The reaction was performed following **Typical Procedure B** with **SI-1 (**1.4 mmol**)** to give the product **1d** (696.4 mg, 94% yield) as white solid, m.p. 98-99 ºC, **^{1}H NMR** (400 MHz, $CDCl_3$) δ 7.60 – 7.52 (m, 1H), 7.46 – 7.38 (m, 4H), 7.37 – 7.27 (m, 3H), 7.22 – 7.15 (m, 1H), 7.12 – 6.96 (m, 7H), 5.52 – 5.23 (m, 2H), 3.00 (s, 2H), 1.22 (s, 6H); **^{13}C NMR** (100 MHz, $CDCl_3$, This compound contains many sp^2-carbons, it's difficult to recognize the C-F couplings. So we list all the signals here.) δ 175.7, 157.3, 157.0, 145.6, 143.1, 129.8, 128.2, 128.2, 128.0, 127.3, 126.7, 126.3 - 126.2 (m), 123.6, 118.8, 118.6, 118.1, 112.3, 112.1, 44.5, 42.2, 26.3; **^{19}F NMR** (376 MHz, $CDCl_3$) δ -125.8 – -

125.9 (m, 1F), -128.0 – -128.1 (m, 1F), -158.7 – -158.8 (m, 1F). HRMS (ESI) m/z calcd. for $C_{32}H_{26}F_3NNaO_3$ [M+Na]$^+$ = 552.1757, found = 552.1771.

1-([1,1'-Biphenyl]-4-yl)-2,2-dimethyl-4-phenylpent-4-en-1-one *O*-(2,3,4-trifluorobenzoyl) oxime（1e）

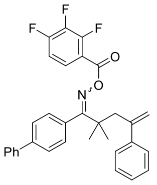

The reaction was performed following **Typical Procedure B** with **SI-1 (**1.7 mmol**)** to give the product **1e** (636.9 mg, 73% yield) as white solid, m.p. 84-85 °C, **¹H NMR** (400 MHz, $CDCl_3$) δ 7.67 – 7.61 (m, 4H), 7.50 (t, *J* = 7.7 Hz, 2H), 7.43 (m, 3H), 7.40 – 7.27 (m, 4H), 7.15 (d, *J* = 8.2 Hz, 2H), 6.91 (m, 1H), 5.49 – 5.25 (m, 2H), 3.03 (s, 2H), 1.24 (s, 6H); **¹³C NMR** (100 MHz, $CDCl_3$, This compound contains many sp^2-carbons, it's difficult to recognize the C-F couplings. So we list all the signals here.) δ 176.0, 145.6, 143.2, 141.2, 140.2, 131.9, 128.9, 128.3, 127.7, 127.3, 127.0, 126.7, 126.6, 126.0 – 125.9 (m), 118.1, 112.2 – 112.1 (m), 112.0, 111.9, 44.5, 42.3, 26.3; **¹⁹F NMR** (376 MHz, $CDCl_3$) δ -125.9 – -126.0 (m, 1F), -128.5 – -128.6 (m, 1F), -158.4 – -158.5 (m, 1F). HRMS (ESI) m/z calcd. for $C_{32}H_{26}F_3NNaO_2$ [M+Na]$^+$ = 536.1808, found = 536.1809.

2,2-Dimethyl-4-phenyl-1-(4-(trifluoromethoxy)phenyl)pent-4-en-1-one *O*-(2,3,4-trifluorobenzoyl) oxime（1f）

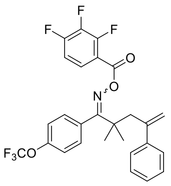

The reaction was performed following **Typical Procedure B** with **SI-1 (**1.5 mmol**)** to give the product **1f** (641.0 mg, 82% yield) as white solid, m.p. 54.1-54.9 °C, **¹H NMR** (400 MHz, $CDCl_3$) δ 7.47 – 7.38 (m, 3H), 7.37 – 7.27 (m, 5H), 7.12 – 7.05 (m, 2H), 6.95 (m, 1H), 5.46 – 5.25 (m, 2H), 2.99 (s, 2H), 1.21 (s, 6H); **¹³C NMR** (100 MHz, $CDCl_3$, This compound contains many sp^2-carbons, it's difficult to recognize the C-F couplings. So we list all the signals here.) δ 174.8, 149.2, 145.5, 143.0, 131.7, 128.3, 128.2, 127.4, 126.7, 126.1 – 126.0 (m), 120.7, 119.1, 118.2, 112.2 (d, *J* = 4.0 Hz), 112.1 (d, *J* = 4.1 Hz), 44.5, 42.1, 26.3; **¹⁹F NMR** (376 MHz, $CDCl_3$) δ -57.9, -125.5 – -125.6 (m, 1F), -128.5 – -128.6 (m, 1F), -158.4 – -158.5 (m, 1F). HRMS (ESI) m/z calcd. for $C_{27}H_{21}F_6NNaO_3$ [M+Na]$^+$ = 544.1318, found = 544.1326.

1-(4-Methoxyphenyl)-2,2-dimethyl-4-phenylpent-4-en-1-one *O*-(2,3,4-trifluorobenzoyl) oxime (1g)

The reaction was performed following **Typical Procedure B** with **SI-1 (**2.3 mmol**)** to give the

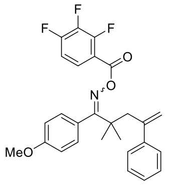

product **1g** (977.8 mg, 91% yield) as white solid, m.p. 55-56 °C, **^{1}H NMR** (400 MHz, $CDCl_3$) δ 7.47 – 7.39 (m, 3H), 7.37 – 7.27 (m, 3H), 7.06 – 6.99 (m, 2H), 6.99 – 6.90 (m, 3H), 5.51 – 5.23 (m, 2H), 3.88 (s, 3H), 3.05 – 2.93 (m, 2H), 1.20 (s, 6H); **^{13}C NMR** (100 MHz, $CDCl_3$, This compound contains many sp^2-carbons, it's difficult to recognize the C-F couplings. So we list all the signals here.) δ 176.0, 160.3, 159.5, 153.0 – 152.7 (m), 145.6, 143.2, 141.5, 128.2, 127.9, 127.2, 126.6, 126.0 – 125.9 (m), 125.1, 118.0, 115.4, 113.4, 112.2 – 112.1 (m), 112.0, 55.2, 44.5, 42.3, 26.3; **^{19}F NMR** (376 MHz, $CDCl_3$) δ -126.1 – -126.2 (m, 1F), -128.5 – -128.6 (m, 1F), -158.5 – -158.6 (m, 1F). HRMS (ESI) m/z calcd. for $C_{27}H_{24}F_3NNaO_3$ [M+Na]$^+$ = 490.1600, found = 490.1613.

2,2-Dimethyl-4-phenyl-1-(4-(trifluoromethyl)phenyl)pent-4-en-1-one *O*-(2,3,4-trifluorobenzoyl) oxime（1h）

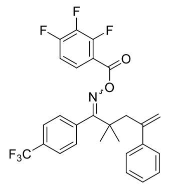

The reaction was performed following **Typical Procedure B** with **SI-1 (**1 mmol**)** to give the product **1h** (383.9 mg, 76% yield) as white solid, m.p. 43.3-43.9 °C, **^{1}H NMR** (400 MHz, $CDCl_3$) δ 7.66 (d, *J* = 8.1 Hz, 2H), 7.42 – 7.35 (m, 3H), 7.33 – 7.27 (m, 2H), 7.24 (d, *J* = 7.3 Hz, 1H), 7.12 (d, *J* = 8.0 Hz, 2H), 6.96 – 6.89 (m, 1H), 5.42 – 5.22 (m, 2H), 2.96 (s, 2H), 1.17 (s, 6H); **^{13}C NMR** (100 MHz, $CDCl_3$, This compound contains many sp^2-carbons, it's difficult to recognize the C-F couplings. So we list all the signals here.) δ 174.6, 160.0, 145.4, 142.9, 136.8, 130.9, 128.3, 127.4, 127.0, 126.7, 126.1, 126.0, 125.1, 125.0, 125.0, 118.3, 112.3, 112.3, 112.1, 112.1, 44.6, 42.0, 26.4; **^{19}F NMR** (376 MHz, $CDCl_3$) δ -62.8, -125.4 – -125.5 (m, 1F), -128.5 – -128.6 (m, 1F), -158.2 – -158.4 (m, 1F). HRMS (ESI) m/z calcd. for $C_{27}H_{21}F_6NNaO_2$ [M+Na]$^+$ = 528.1369, found = 528.1358.

2,2-Dimethyl-4-phenyl-1-(*m*-tolyl)pent-4-en-1-one *O*-(2,3,4-trifluorobenzoyl) oxime (1i)

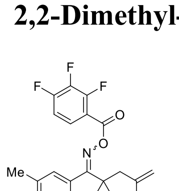

The reaction was performed following **Typical Procedure B** with **SI-1 (**3 mmol**)** to give the product **1i** (1.22 g, 90% yield) as white solid, m.p. 60-61 °C, **^{1}H NMR** (400 MHz, $CDCl_3$) δ 7.46 – 7.40 (m, 2H), 7.39 – 7.27 (m, 5H), 7.22 (d, *J* = 7.8 Hz, 1H), 6.98 – 6.91 (m, 1H), 6.90 – 6.83 (m, 2H), 5.47 – 5.24 (m, 2H), 3.07 – 2.95 (m, 2H), 2.39 (s, 3H), 1.20 (s, 6H); **^{13}C NMR** (100 MHz, $CDCl_3$, This compound contains many sp^2-carbons, it's difficult to recognize the C-F couplings. So we list all the signals here.) δ 176.2, 145.7, 143.2, 137.6, 132.9, 129.1, 128.2, 127.8, 127.2, 127.0, 126.7, 126.0, 126.0 - 125.9 (m), 125.9, 123.5, 118.1, 112.1, 112.0, 44.5, 42.1, 26.3, 21.4; **^{19}F NMR** (376 MHz, $CDCl_3$)

δ -126.1 – -126.2 (m, 1F), -128.7 – -128.8 (m, 1F), -158.6 – -158.7 (m, 1F). HRMS (ESI) m/z calcd. for $C_{27}H_{24}F_3NNaO_2$ $[M+Na]^+$ = 474.1651, found = 474.1664.

1-(3-Methoxyphenyl)-2,2-dimethyl-4-phenylpent-4-en-1-one *O*-(2,3,4-trifluorobenzoyl) oxime（1j）

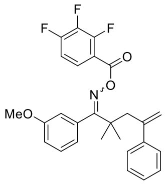

The reaction was performed following **Typical Procedure B** with **SI-1 (**2.1 mmol**)** to give the product **1j** (804.5 mg, 82% yield) as colorless oil, **^{1}H NMR** (400 MHz, $CDCl_3$) δ 7.47 – 7.39 (m, 3H), 7.38 – 7.27 (m, 4H), 7.01 – 6.91 (m, 2H), 6.68 (m, 1H), 6.62 (m, 1H), 5.48 – 5.25 (m, 2H), 3.83 (s, 3H), 3.02 (s, 2H), 1.22 (s, 6H); **^{13}C NMR** (100 MHz, $CDCl_3$, This compound contains many sp^2-carbons, it's difficult to recognize the C-F couplings. So we list all the signals here.) δ 175.7, 160.2, 159.1, 155.4 – 155.2 (m), 152.8 – 152.6 (m), 145.6, 143.1, 134.2, 129.1, 128.2, 127.2, 126.6, 126.0, 118.9, 118.1, 115.4 – 115.2 (m), 113.8, 112.3, 112.1, 112.0, 55.3, 44.5, 42.1, 26.3; **^{19}F NMR** (376 MHz, $CDCl_3$) δ -126.0 – -126.1 (m, 1F), -128.6 – -128.7 (m, 1F), -158.5 – -158.6 (m, 1F). HRMS (ESI) m/z calcd. for $C_{27}H_{24}F_3NNaO_3$ $[M+Na]^+$ = 490.1600, found = 490.1614.

1-(3-Fluorophenyl)-2,2-dimethyl-4-phenylpent-4-en-1-one *O*-(2,3,4-trifluorobenzoyl) oxime（1k）

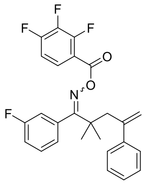

The reaction was performed following **Typical Procedure B** with **SI-1 (**1 mmol**)** to give the product **1k** (400.5 mg, 88% yield) as colorless oil, **^{1}H NMR** (400 MHz, $CDCl_3$) δ 7.49 – 7.37 (m, 4H), 7.35 – 7.27 (m, 3H), 7.11 (m, 1H), 6.95 (m, 1H), 6.83 (m, 1H), 6.75 (m, 1H), 5.43 – 5.24 (m, 2H), 2.98 (s, 2H), 1.19 (s, 6H); **^{13}C NMR** (100 MHz, $CDCl_3$, This compound contains many sp^2-carbons, it's difficult to recognize the C-F couplings. So we list all the signals here.) δ 174.5, 163.5, 161.0, 160.1, 155.5 – 155.4 (m), 152.9 – 152.7 (m), 145.5, 143.0, 134.9, 130.8, 128.3, 127.3, 126.7, 126.1 – 126.0 (m), 122.3, 118.2, 115.4, 113.9, 112.3 – 112.2 (m), 112.1 – 112.0 (m), 44.6, 42.1, 26.3; **^{19}F NMR** (376 MHz, $CDCl_3$) δ -112.2, -125.6 – -125.8 (m, 1F), -128.4 – -128.5 (m, 1F), -158.3 – -158.5 (m, 1F). HRMS (ESI) m/z calcd. for $C_{26}H_{21}F_4NNaO_2$ $[M+Na]^+$ = 478.1401, found = 478.1413.

2,2-Dimethyl-4-phenyl-1-(3-(trifluoromethyl)phenyl)pent-4-en-1-one *O*-(2,3,4-trifluorobenzoyl) oxime（1l）

The reaction was performed following **Typical Procedure B** with **SI-1 (**1.3 mmol**)** to give the

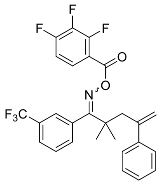

product **1l** (512.2 mg, 78% yield) as colorless oil, **^{1}H NMR** (400 MHz, $CDCl_3$) δ 7.66 (d, *J* = 7.9 Hz, 1H), 7.52 (t, *J* = 7.8 Hz, 1H), 7.45 – 7.36 (m, 3H), 7.31 – 7.27 (m, 2H), 7.26 – 7.17 (m, 3H), 6.96 – 6.89 (m, 1H), 5.46 – 5.19 (m, 2H), 2.97 (s, 2H), 1.18 (s, 6H); **^{13}C NMR** (100 MHz, $CDCl_3$, This compound contains many sp^2-carbons, it's difficult to recognize the C-F couplings. So we list all the signals here.) δ 174.1, 156.0, 155.5, 145.6, 142.8, 133.9, 130.8, 130.5, 130.1, 129.9, 128.6, 128.3, 127.4, 126.8, 126.1, 126.1, 126.1, 126.0, 125.3, 125.3, 123.6, 123.6, 123.5, 118.3, 112.3, 112.2, 112.1, 112.1, 44.9, 42.0, 26.6; **^{19}F NMR** (376 MHz, $CDCl_3$) δ -62.8, -125.5 – -125.6 (m, 1F), -128.5 – -128.6 (m, 1F), -158.3 – -158.5 (m, 1F). HRMS (ESI) m/z calcd. for $C_{27}H_{21}F_6NNaO_2$ $[M+Na]^+$ = 528.1369, found = 528.1367.

1-(2,3-Dihydrobenzo[*b*][1,4]dioxin-6-yl)-2,2-dimethyl-4-phenylpent-4-en-1-one *O*-(2,3,4-trifluorobenzoyl) oxime（1m）

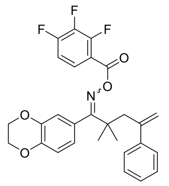

The reaction was performed following **Typical Procedure B** with **SI-1 (**3 mmol**)** to give the product **1m** (512.2 mg, 78% yield) as colorless oil, **^{1}H NMR** (400 MHz, $CDCl_3$) δ 7.50 – 7.38 (m, 3H), 7.35 – 7.26 (m, 3H), 6.96 (m, 1H), 6.90 (d, *J* = 8.3 Hz, 1H), 6.60 (d, *J* = 2.0 Hz, 1H), 6.55 – 6.53 (m, 1H), 5.43 – 5.22 (m, 2H), 4.36 – 4.27 (m, 4H), 2.96 (s, 2H), 1.18 (s, 6H); **^{13}C NMR** (100 MHz, $CDCl_3$, This compound contains many sp^2-carbons, it's difficult to recognize the C-F couplings. So we list all the signals here.) δ 175.5, 160.3, 152.5 – 152.7 (m), 145.7, 143.6, 143.2, 143.1, 141.6, 139.0, 128.2, 127.2, 126.6, 126.1 – 126.0 (m), 125.9, 119.9, 118.0, 116.9, 115.9, 112.1, 112.0, 64.4, 64.3, 44.5, 42.3, 26.3; **^{19}F NMR** (376 MHz, $CDCl_3$) δ -126.1 – -126.2 (m, 1F), -128.4 – -128.5 (m, 1F), -158.6 – -158.7 (m, 1F). HRMS (ESI) m/z calcd. for $C_{28}H_{24}F_3NNaO_4$ $[M+Na]^+$ = 518.1550, found = 518.1563.

1-(3,4-Dimethoxyphenyl)-2,2-dimethyl-4-phenylpent-4-en-1-one *O*-(2,3,4-trifluorobenzoyl) oxime（1n）

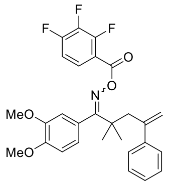

The reaction was performed following **Typical Procedure B** with **SI-1** (2 mmol) to give the product **1n** (835.3 mg, 84% yield) as colorless oil, **^{1}H NMR** (400 MHz, $CDCl_3$) δ 7.47 – 7.39 (m, 3H), 7.35 – 7.27 (m, 3H), 7.00 – 6.88 (m, 2H), 6.64 (dd, *J* = 8.2, 1.9 Hz, 1H), 6.56 (d, *J* = 1.9 Hz, 1H), 5.46 – 5.24 (m, 2H), 3.93 (s, 3H), 3.84 (s, 3H), 2.99 (s, 2H), 1.21 (s, 6H); **^{13}C NMR** (100 MHz, $CDCl_3$, This

compound contains many sp^2-carbons, it's difficult to recognize the C-F couplings. So we list all the signals here.) δ 175.8, 160.3 – 160.2 (m), 155.4 – 155.2 (m), 152.8 – 155.6 (m), 149.0, 148.5, 145.7, 143.0, 128.2, 127.2, 126.6, 126.0 – 125.9 (m), 125.3, 119.1, 117.9, 115.4 – 115.3 (m), 112.2, 112.0, 110.6, 110.2, 56.0, 55.8, 44.6, 42.3, 26.4; **^{19}F NMR** (376 MHz, $CDCl_3$) δ -125.9 – -126.0 (m, 1F), -128.5 – -128.6 (m, 1F), -158.3 – -158.5 (m, 1F). HRMS (ESI) m/z calcd. for $C_{28}H_{26}F_3NNaO_4$ $[M+Na]^+$ = 520.1706, found = 520.1718.

1-((9*r*,10*r*)-9,10-Dihydro-9,10-[1,2]benzenoanthracen-2-yl)-2,2-dimethyl-4-phenylpent-4-en-1-one *O*-(2,3,4-trifluorobenzoyl) oxime（1o）

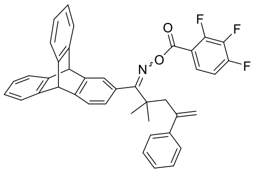

The reaction was performed following **Typical Procedure B** with **SI-1** (1 mmol) to give the product **1o** (551.9 mg, 90% yield) as white solid, m.p. 51-52 °C, **^{1}H NMR** (400 MHz, $CDCl_3$) δ 7.47 – 7.35 (m, 7H), 7.33 – 7.27 (m, 3H), 7.11 – 7.01 (m, 5H), 6.70 – 6.68 (m, 1H), 6.52 – 6.41 (m, 1H), 6.14 (m, 1H), 5.48 (s, 1H), 5.43 – 5.35 (m, 2H), 5.24 (d, J = 1.6 Hz, 1H), 2.92 (s, 2H), 1.17 (s, 6H); **^{13}C NMR** (100 MHz, $CDCl_3$, This compound contains many sp^2-carbons, it's difficult to recognize the C-F couplings. So we list all the signals here.) δ 176.7, 145.6, 145.5, 145.3, 144.9, 144.8, 143.2, 130.0, 128.2, 127.2, 126.7, 125.3, 125.2 – 125.1 (m), 123.7, 123.6, 123.1, 123.0, 122.3, 118.0, 111.7, 111.6, 53.9, 53.8, 44.4, 42.1, 26.1; **^{19}F NMR** (376 MHz, $CDCl_3$) δ -126.4 – -126.5 (m, 1F), -130.0 – -130.2 (m, 1F), -158.5 – -158.6 (m, 1F). HRMS (ESI) m/z calcd. for $C_{40}H_{30}F_3NNaO_2$ $[M+Na]^+$ = 636.2122, found = 636.2141.

2,2-Dimethyl-1-(naphthalen-2-yl)-4-phenylpent-4-en-1-one *O*-(2,3,4-trifluorobenzoyl) oxime（1p）

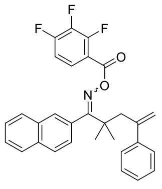

The reaction was performed following **Typical Procedure B** with **SI-1** (1.7 mmol) to give the product **1p** (662.6 mg, 80% yield) as white solid, m.p. 94.1-94.9 °C, **^{1}H NMR** (400 MHz, $CDCl_3$) δ 7.93 – 7.82 (m, 3H), 7.61 – 7.53 (m, 2H), 7.49 (d, J = 1.6 Hz, 1H), 7.45 – 7.40 (m, 2H), 7.36 – 7.27 (m, 4H), 7.20 – 7.17 (m, 1H), 6.82 (m, 1H), 5.50 – 5.25 (m, 2H), 3.06 (s, 2H), 1.26 (s, 6H); **^{13}C NMR** (100 MHz, $CDCl_3$, This compound contains many sp^2-carbons, it's difficult to recognize the C-F couplings. So we list all the signals here.) δ 175.9, 152.8 – 152.6 (m), 145.7, 143.1, 132.8, 132.5, 130.5, 128.2, 128.1, 127.8, 127.6, 127.3, 126.8, 126.7, 126.6, 125.9 – 125.5 (m), 124.6, 118.2, 112.1, 111.9, 44.7, 42.4, 26.4; **^{19}F NMR** (376 MHz, $CDCl_3$) δ -126.0 – -126.1 (m, 1F), -128.6 – -128.7 (m,

1F), -158.4 – -158.6 (m, 1F). HRMS (ESI) m/z calcd. for $C_{30}H_{24}F_3NNaO_2$ $[M+Na]^+$ = 510.1651, found = 510.1664.

4-(3-Fluorophenyl)-2,2-dimethyl-1-phenylpent-4-en-1-one *O*-(2,3,4-trifluorobenzoyl) oxime (1q)

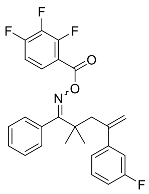

The reaction was performed following **Typical Procedure B** with **SI-1** (2.1 mmol) to give the product **1q** (755.1 mg, 79% yield) as colorless oil, **^{1}H NMR** (400 MHz, $CDCl_3$) δ 7.43 – 7.30 (m, 4H), 7.26 – 7.22 (m, 1H), 7.17 – 7.15 (m, 1H), 7.10 – 7.01 (m, 3H), 6.97 – 6.86 (m, 2H), 5.46 – 5.25 (m, 2H), 2.94 (s, 2H), 1.16 (s, 6H); **^{13}C NMR** (100 MHz, $CDCl_3$, This compound contains many sp^2-carbons, it's difficult to recognize the C-F couplings. So we list all the signals here.) δ 175.8, 164.0, 161.5, 152.7, 145.6, 145.5, 144.5, 132.9, 129.7, 129.6, 128.5, 128.0, 126.4, 126.0, 126.0, 125.9, 125.9, 122.3, 122.3, 119.1, 114.1, 113.9, 113.6, 113.4, 112.1, 112.1, 112.0, 111.9, 44.3, 42.1, 26.2; **^{19}F NMR** (376 MHz, $CDCl_3$) δ -113.4 – -113.5 (m, 1F), -126.0 – -126.1 (m, 1F), -128.6 – -128.7 (m, 1F), -158.2 – -158.6 (m, 1F). HRMS (ESI) m/z calcd. for $C_{26}H_{21}F_4NNaO_2$ $[M+Na]^+$ = 478.1401, found = 478.1406.

4-(3-Methoxyphenyl)-2,2-dimethyl-1-phenylpent-4-en-1-one *O*-(2,3,4-trifluorobenzoyl) oxime (1r)

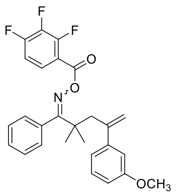

The reaction was performed following **Typical Procedure B** with **SI-1** (1.4 mmol) to give the product **1r** (582.1 mg, 89% yield) as colorless oil, **^{1}H NMR** (400 MHz, $CDCl_3$) δ 7.42 – 7.29 (m, 4H), 7.21 (t, *J* = 7.9 Hz, 1H), 7.08 – 7.03 (m, 2H), 6.96 (dd, *J* = 7.7, 1.4 Hz, 1H), 6.93 – 6.85 (m, 2H), 6.79 (dd, *J* = 8.2, 2.6 Hz, 1H), 5.47 – 5.20 (m, 2H), 3.79 (s, 3H), 2.94 (s, 2H), 1.17 (s, 6H); **^{13}C NMR** (100 MHz, $CDCl_3$, This compound contains many sp^2-carbons, it's difficult to recognize the C-F couplings. So we list all the signals here.) δ 176.0, 160.1, 159.5, 152.8, 145.4, 144.7, 141.5, 133.0, 129.2, 128.4, 128.0, 126.5, 126.0, 126.0, 125.9, 125.9, 119.1, 118.2, 112.5, 112.1, 112.1, 111.9, 111.9, 55.2, 44.4, 42.2, 26.1; **^{19}F NMR** (376 MHz, $CDCl_3$) δ -126.0 – -126.1 (m, 1F), -128.6 – -128.7 (m, 1F), -158.6 – -158.7 (m, 1F). HRMS (ESI) m/z calcd. for $C_{27}H_{24}F_3NNaO_3$ $[M+Na]^+$ = 490.1600, found = 490.1602.

4-(4-(*Tert*-butyl)phenyl)-2,2-dimethyl-1-phenylpent-4-en-1-one *O*-(2,3,4-trifluorobenzoyl) oxime（1s）

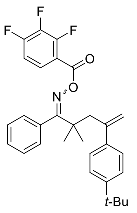

The reaction was performed following **Typical Procedure B** with **SI-1** (1.1 mmol) to give the product **1s** (444.9 mg, 82% yield) as white solid, m.p. 53-54 ^{o}C, **^{1}H NMR** (400 MHz, $CDCl_3$) δ 7.38 (q, *J* = 2.8 Hz, 3H), 7.31 (s, 5H), 7.01 – 6.98 (m, 2H), 6.89 (m, 1H), 5.45 – 5.16 (m, 2H), 2.94 (s, 2H), 1.30 (s, 9H), 1.19 (s, 6H); **^{13}C NMR** (100 MHz, $CDCl_3$, This compound contains many sp^2-carbons, it's difficult to recognize the C-F couplings. So we list all the signals here.) δ 176.1, 160.2 – 160.0 (m), 152.9 – 152.7 (m), 150.3, 145.3, 140.0, 133.1, 128.4, 127.9, 126.5, 126.4, 126.0 – 125.9 (m), 125.1, 117.3, 115.4 – 115.3 (m), 112.1, 111.9, 44.5, 42.0, 34.4, 31.3, 26.4; **^{19}F NMR** (376 MHz, $CDCl_3$) δ -115.5, -126.0 – -126.1 (m, 1F), -128.5 – -128.7 (m, 1F), -158.6 – -158.7 (m, 1F). HRMS (ESI) m/z calcd. for $C_{30}H_{30}F_3NNaO_2$ $[M+Na]^+$ = 516.2121, found = 516.2134.

4-(4-Methoxyphenyl)-2,2-dimethyl-1-phenylpent-4-en-1-one *O*-(2,3,4-trifluorobenzoyl) oxime（1t）

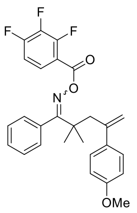

The reaction was performed following **Typical Procedure B** with **SI-1** (1.5 mmol) to give the product **1t** (610.0 mg, 87% yield) as colorless oil, **^{1}H NMR** (400 MHz, $CDCl_3$) δ 7.39 (dd, *J* = 5.1, 1.9 Hz, 3H), 7.32 (dd, *J* = 9.2, 2.6 Hz, 3H), 7.09 – 7.02 (m, 2H), 6.95 – 6.87 (m, 1H), 6.86 – 6.80 (m, 2H), 5.33 (d, *J* = 1.7 Hz, 1H), 5.15 (d, *J* = 1.6 Hz, 1H), 3.78 (s, 3H), 2.93 (s, 2H), 1.17 (s, 6H); **^{13}C NMR** (100 MHz, $CDCl_3$, This compound contains many sp^2-carbons, it's difficult to recognize the C-F couplings. So we list all the signals here.) δ 176.2, 158.9, 144.8, 135.5, 133.1, 128.4, 127.9, 127.7, 126.5, 126.0, 126.0, 125.9, 125.9, 116.7, 113.5, 112.1, 112.1, 111.9, 111.9, 55.2, 44.4, 42.1, 26.2; **^{19}F NMR** (376 MHz, $CDCl_3$) δ -126.0 – -126.1 (m, 1F), -128.6 – -128.7 (m, 1F), -158.6 – -158.7 (m, 1F). HRMS (ESI) m/z calcd. for $C_{27}H_{24}F_3NNaO_3$ $[M+Na]^+$ = 490.1600, found = 490.1613.

1-(2,3-Dihydrobenzofuran-5-yl)-2,2-dimethyl-4-phenylpent-4-en-1-one O-(2,3,4-trifluorobenzoyl) oxime（1u）

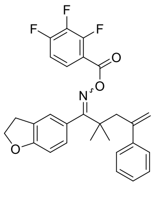

The reaction was performed following **Typical Procedure B** with **SI-1** (1.3 mmol) to give the product **1u** (523.3 mg, 84% yield) as white solid, m.p. 98-99 ^{o}C, **^{1}H NMR** (400 MHz, $CDCl_3$) δ 7.50 – 7.40 (m, 3H), 7.37 – 7.27 (m, 3H), 6.97 (m, 1H), 6.88 (d, *J* = 1.5 Hz, 1H), 6.83 (d, *J* = 1.2 Hz, 2H), 5.48 – 5.24 (m, 2H), 4.65 (t, *J* = 8.7 Hz, 2H), 3.32 – 3.20 (m, 2H), 2.98 (s, 2H), 1.21 (s, 6H); **^{13}C NMR** (100 MHz, $CDCl_3$, This compound contains many sp^2-carbons, it's difficult to recognize the C-F

couplings. So we list all the signals here.) δ 176.3, 160.4, 160.1, 155.3 – 155.2 (m), 152.8 – 152.6 (m), 150.2 – 149.9 (m), 145.7, 143.2, 128.2, 127.2, 126.8, 126.6, 126.6, 126.1 – 125.9 (m), 124.8, 123.4, 118.0, 112.2 – 112.1 (m), 112.0, 108.7, 71.3, 44.6, 42.3, 29.5, 26.4; ^{19}**F NMR** (376 MHz, $CDCl_3$) δ -126.1 – -126.2 (m, 1F), -128.6 – -128.7 (m, 1F), -158.5 – -158.6 (m, 1F). HRMS (ESI) m/z calcd. for $C_{28}H_{24}F_3NNaO_3$ $[M+Na]^+$ = 502.1600, found = 502.1613.

1-(2,3-Dihydrobenzofuran-5-yl)-2,2-dimethyl-4-(*p*-tolyl)pent-4-en-1-one *O*-(2,3,4-trifluorobenzoyl) oxime（1v）

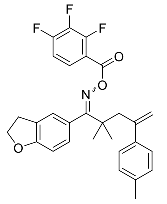

The reaction was performed following **Typical Procedure B** with **SI-1** (1 mmol) to give the product **1v** (424.1 mg, 86% yield) as white solid, m.p. 60.0-60.7 °C, ^{1}**H NMR** (400 MHz, $CDCl_3$) δ 7.45 – 7.39 (m, 1H), 7.29 – 7.26 (m, 2H), 7.10 (d, *J* = 7.9 Hz, 2H), 6.93 (m, 1H), 6.85 (s, 1H), 6.79 (d, *J* = 2.1 Hz, 2H), 5.39 – 5.15 (m, 2H), 4.61 (t, *J* = 8.7 Hz, 2H), 3.22 (t, *J* = 8.7 Hz, 2H), 2.91 (s, 2H), 2.33 (s, 3H), 1.16 (s, 6H); ^{13}**C NMR** (100 MHz, $CDCl_3$, This compound contains many sp^2-carbons, it's difficult to recognize the C-F couplings. So we list all the signals here.) δ 176.4, 160.1, 145.4, 140.3, 136.9, 128.9, 126.7, 126.6, 126.5, 126.1 – 126.0 (m), 124.9, 123.5, 117.3, 112.2 – 112.1 (m), 112.0, 108.7, 71.4, 44.5, 42.4, 29.6, 26.3, 21.0; ^{19}**F NMR** (376 MHz, $CDCl_3$) δ -126.1 – -126.2 (m, 1F), -128.6 – -128.7 (m, 1F), -158.5 – -158.7 (m, 1F). HRMS (ESI) m/z calcd. for $C_{29}H_{26}F_3NNaO_3$ $[M+Na]^+$ = 516.1757, found = 516.1769.

1-(2,3-Dihydrobenzofuran-5-yl)-4-(4-fluorophenyl)-2,2-dimethylpent-4-en-1-one *O*-(2,3,4-trifluorobenzoyl) oxime（1w）

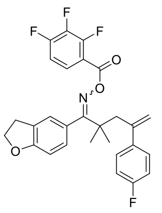

The reaction was performed following **Typical Procedure B** with **SI-1** (1.3 mmol) to give the product **1w** (568.8 mg, 88% yield) as white solid, m.p. 74-75 °C, ^{1}**H NMR** (400 MHz, $CDCl_3$) δ 7.46 – 7.37 (m, 1H), 7.37 – 7.30 (m, 2H), 7.02 – 6.89 (m, 3H), 6.83 (t, *J* = 1.2 Hz, 1H), 6.77 (t, *J* = 1.4 Hz, 2H), 5.40 – 5.19 (m, 2H), 4.61 (t, *J* = 8.7 Hz, 2H), 3.21 (t, *J* = 8.7 Hz, 2H), 2.91 (s, 2H), 1.15 (s, 6H); ^{13}**C NMR** (100 MHz, $CDCl_3$, This compound contains many sp^2-carbons, it's difficult to recognize the C-F couplings. So we list all the signals here.) δ 176.1, 163.4, 161.0, 160.1, 144.7, 139.3, 128.2, 126.8, 126.6, 126.1 – 125.9 (m), 124.8, 123.4, 118.1, 115.1, 114.9, 112.2, 112.0, 108.8, 71.4, 44.8, 42.3, 29.5, 26.4; ^{19}**F NMR** (376 MHz, $CDCl_3$) δ -115.5, -126.0 – -126.1 (m, 1F), -

128.6 – -128.7 (m, 1F), -158.5 – -158.6 (m, 1F). HRMS (ESI) m/z calcd. for $C_{28}H_{23}F_4NNaO_3$ $[M+Na]^+$ = 520.1506, found = 520.1519.

2,2-Dimethyl-4-phenyl-1-(thiophen-2-yl)pent-4-en-1-one *O*-(2,3,4-trifluorobenzoyl) oxime (1x)

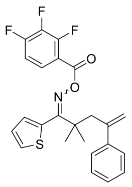

The reaction was performed following **Typical Procedure B** with **SI-1** (1.5 mmol) to give the product **1x** (478.6 mg, 72% yield) as white solid, m.p. 38.9-39.1 °C, **^{1}H NMR** (400 MHz, $CDCl_3$) δ 7.60 – 7.48 (m, 2H), 7.45 – 7.39 (m, 2H), 7.37 – 7.28 (m, 3H), 7.14 (dd, *J* = 5.1, 3.6 Hz, 1H), 7.08 – 6.97 (m, 2H), 5.41 (d, *J* = 1.6 Hz, 1H), 5.26 (d, *J* = 1.3 Hz, 1H), 3.00 (s, 2H), 1.30 (s, 6H); **^{13}C NMR** (100 MHz, $CDCl_3$, This compound contains many sp^2-carbons, it's difficult to recognize the C-F couplings. So we list all the signals here.) δ 169.5, 145.5, 143.1, 130.0, 128.2, 128.1, 127.2, 127.0, 126.7, 126.5, 126.2, 126.1, 126.1, 117.9, 112.3, 112.2, 112.1, 112.0, 44.8, 42.7, 26.4; **^{19}F NMR** (376 MHz, $CDCl_3$) δ -125.7 – -158.8 (m, 1F), -128.4 – -128.5 (m, 1F), -158.4 – -158.5 (m, 1F). HRMS (ESI) m/z calcd. for $C_{24}H_{20}F_3NNaO_2S$ $[M+Na]^+$ = 466.1059, found = 466.1066.

Phenyl(1-(2-phenylallyl)cyclobutyl)methanone *O*-(2,3,4-trifluorobenzoyl) oxime（1y）

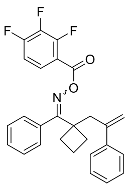

The reaction was performed following **Typical Procedure B** with **SI-1** (1 mmol) to give the product **1y** (386.3 mg, 86% yield) as colorless oil, **^{1}H NMR** (400 MHz, $CDCl_3$) δ 7.48 – 7.29 (m, 5H), 7.26 – 7.19 (m, 4H), 7.18 – 7.11 (m, 2H), 6.94 – 6.87 (m, 1H), 5.42 (s, 1H), 5.30 (s, 1H), 2.98 – 2.87 (m, 2H), 2.81 (d, *J* = 1.4 Hz, 2H), 2.21 – 2.11 (m, 2H), 2.10 – 1.99 (m, 2H); **^{13}C NMR** (100 MHz, $CDCl_3$, This compound contains many sp^2-carbons, it's difficult to recognize the C-F couplings. So we list all the signals here.) δ 173.2, 160.3, 152.7, 144.8, 142.6, 141.5, 132.5, 128.8, 128.2, 128.2, 128.0, 127.9, 127.3, 126.8, 126.4, 126.1, 126.0, 126.0, 125.6, 114.9, 112.2, 112.1, 112.0, 111.9, 47.4, 41.3, 30.9, 15.7; **^{19}F NMR** (376 MHz, $CDCl_3$) δ -126.0 – -126.1 (m, 1F), -128.4 – -128.5 (m, 1F), -158.5 – -158.7 (m, 1F). HRMS (ESI) m/z calcd. for $C_{27}H_{22}F_3NNaO_2$ $[M+Na]^+$ = 472.1495, found = 472.1508.

Phenyl(1-(2-phenylallyl)cyclopentyl)methanone *O*-(2,3,4-trifluorobenzoyl) oxime（1z）

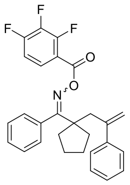

The reaction was performed following **Typical Procedure B** with **SI-1** (3.5 mmol) to give the product **1z** (1.394 g, 86% yield) as colorless oil, **^{1}H NMR** (400 MHz, $CDCl_3$) δ 7.45 – 7.27 (m, 9H), 7.17 – 7.10 (m, 2H), 6.96 – 6.90 (m, 1H), 5.49 (s, 2H), 2.87 (s, 2H), 2.37 – 2.20 (m, 2H), 1.93 – 1.68 (m, 6H); **^{13}C NMR** (100 MHz, $CDCl_3$, This compound contains many sp^2-carbons, it's difficult to recognize the C-F couplings. So we list all the signals here.) δ 174.4, 145.9, 143.0, 133.3, 128.5, 128.2, 127.9, 127.3, 126.5, 126.3, 126.0 – 125.9 (m), 116.5, 112.1, 111.9, 53.5, 42.2, 36.2, 23.8; ^{19}F NMR (376 MHz, $CDCl_3$) δ -126.1 – -126.2 (m, 1F), -128.6 – -128.7 (m, 1F), -158.6 – -158.7 (m, 1F). HRMS (ESI) m/z calcd. for $C_{28}H_{24}F_3NNaO_2$ $[M+Na]^+$ = 486.1651, found = 486.1665.

(4*E*)-2,2,4-Trimethyl-1,5-diphenylpent-4-en-1-one *O*-(2,3,4-trifluorobenzoyl) oxime （1ad）

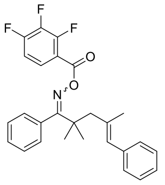

The reaction was performed following **Typical Procedure B** with **SI-3** (10 mmol) to give the product **1ad** (1.35 g, 30% overall yield in 3 steps) as white solid, m.p. 69.1-69.4 °C, **^{1}H NMR** (400 MHz, $CDCl_3$) δ 7.55 – 7.47 (m, 3H), 7.41 (t, *J* = 7.6 Hz, 3H), 7.37 – 7.27 (m, 3H), 7.26 – 7.20 (m, 2H), 7.00 – 6.93 (m, 1H), 6.46 (s, 1H), 2.67 (s, 2H), 2.06 (s, 3H), 1.44 (s, 6H); **^{13}C NMR** (100 MHz, $CDCl_3$, This compound contains many sp^2-carbons, it's difficult to recognize the C-F couplings. So we list all the signals here.) δ 176.3, 138.2, 135.1, 133.2, 130.2, 128.9, 128.4, 128.1, 128.0, 126.6, 126.2, 126.0 – 125.9 (m), 112.1, 112.1, 112.0, 111.9, 50.2, 42.4, 26.3, 20.9; **^{19}F NMR** (376 MHz, $CDCl_3$) δ -126.0 – -126.1 (m, 1F), -128.5 – -128.6 (m, 1F), -158.5 – -158.7 (m, 1F). HRMS (ESI) m/z calcd. for $C_{27}H_{24}F_3NNaO_2$ $[M+Na]^+$ = 474.1651, found = 474.1658.

(4Z)-2,2-Dimethyl-1,4,5-triphenylpent-4-en-1-one *O*-(2,3,4-trifluorobenzoyl) oxime （1ae）

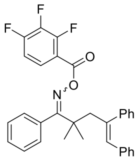

The reaction was performed following **Typical Procedure B** with **SI-3** (13 mmol) to give the product **1ae** (1.33 g, 20% overall yield in 3 steps) as white solid, m.p. 84.1-84.4 °C, **^{1}H NMR** (400 MHz, $CDCl_3$) δ 7.47 – 7.43 (m, 3H), 7.43 – 7.36 (m, 1H), 7.35 – 7.27 (m, 5H), 7.21 – 7.12 (m, 3H), 7.08 – 6.93 (m, 5H), 6.70 (s, 1H), 3.06 (s, 2H), 1.31 (s, 6H); **^{13}C NMR** (100 MHz, $CDCl_3$, This compound contains many sp^2-carbons, it's difficult to recognize the C-F couplings. So we list all the signals here.) δ 175.8, 160.2, 159.3, 152.7, 141.6, 139.1, 137.2, 133.0, 131.3, 129.3, 129.1, 128.4, 128.4, 127.9, 127.8, 127.0, 126.6, 126.3, 126.0, 112.1, 112.1, 111.9, 111.9, 49.4, 42.6, 26.4; **^{19}F NMR** (376 MHz,

$CDCl_3$) δ -126.0 – -126.2 (m, 1F), -128.6 – -128.7 (m, 1F), -158.5 – -158.7 (m, 1F). HRMS (ESI) m/z calcd. for $C_{32}H_{26}F_3NNaO_2$ $[M+Na]^+$ = 536.1808, found = 536.1818.

(4*E*)-2,2,4-Trimethyl-5-phenyl-1-(*p*-tolyl)pent-4-en-1-one *O*-(2,3,4-trifluorobenzoyl) oxime (1af)

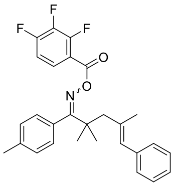

The reaction was performed following **Typical Procedure B** with **SI-3** (10 mmol) to give the product **1af** (1.26 g, 27% overall yield in 3 steps) as colorless oil, **^{1}H NMR** (400 MHz, $CDCl_3$) δ 7.44 – 7.27 (m, 5H), 7.25 – 7.16 (m, 3H), 7.06 (d, *J* = 7.9 Hz, 2H), 6.94 – 6.87 (m, 1H), 6.38 (s, 1H), 2.58 (s, 2H), 2.41 (s, 3H), 1.99 (s, 3H), 1.36 (s, 6H); **^{13}C NMR** (100 MHz, $CDCl_3$, This compound contains many sp^2-carbons, it's difficult to recognize the C-F couplings. So we list all the signals here.) δ 176.5, 160.3, 138.2, 138.2, 135.2, 130.1, 130.1, 128.9, 128.8, 128.6, 128.5, 128.0, 126.6, 126.5, 126.1, 126.0, 126.0, 126.0, 125.9, 112.1, 112.1, 111.9, 111.9, 50.2, 42.5, 26.3, 21.3, 20.9; **^{19}F NMR** (376 MHz, $CDCl_3$) δ -126.2, -128.6, -158.6. HRMS (ESI) m/z calcd. for $C_{28}H_{26}F_3NNaO_2$ $[M+Na]^+$ = 488.1808, found = 488.1820.

(4*E*)-1-(4-Methoxyphenyl)-2,2,4-trimethyl-5-phenylpent-4-en-1-one *O*-(2,3,4-trifluorobenzoyl) oxime (1ag)

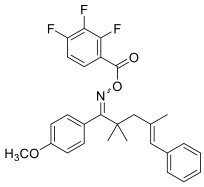

The reaction was performed following **Typical Procedure B** with **SI-3** (15 mmol) to give the product **1ag** (2.4 g, 33% overall yield in 3 steps) as colorless oil, **^{1}H NMR** (400 MHz, $CDCl_3$) δ 7.41 (t, *J* = 7.5 Hz, 2H), 7.36 – 7.27 (m, 3H), 7.18 (d, *J* = 8.7 Hz, 2H), 7.06 – 6.94 (m, 3H), 6.46 (s, 1H), 3.93 (s, 3H), 2.65 (s, 2H), 2.06 (s, 3H), 1.44 (s, 6H); **^{13}C NMR** (100 MHz, $CDCl_3$, This compound contains many sp^2-carbons, it's difficult to recognize the C-F couplings. So we list all the signals here.) δ 176.2, 159.5, 138.2, 135.2, 130.0, 128.9, 128.8, 128.1, 128.0, 126.1, 126.1, 126.0, 126.0, 125.9, 125.2, 113.4, 112.1, 112.1, 112.0, 111.9, 55.2, 50.3, 42.6, 26.4, 20.8; **^{19}F NMR** (376 MHz, $CDCl_3$) δ -126.1 – -126.2 (m, 1F), -128.5 – -128.6 (m, 1F), -158.1 – -158.6 (m, 1F). HRMS (ESI) m/z calcd. for $C_{28}H_{26}F_3NNaO_3$ $[M+Na]^+$ = 504.1757, found = 504.1762.

(4*E*)-2,2,4-Trimethyl-1-(4-phenoxyphenyl)-5-phenylpent-4-en-1-one *O*-(2,3,4-trifluorobenzoyl) oxime (1ai)

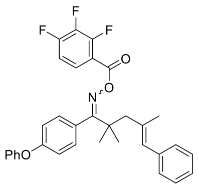

The reaction was performed following **Typical Procedure B** with **SI-3** (6 mmol) to give the product **1ai** (814.8 mg, 25% overall yield in 3 steps) as white solid, m.p. 79.4-79.9 °C, **^{1}H NMR** (400 MHz, $CDCl_3$) δ 7.51 – 7.43 (m, 1H), 7.33 – 7.27 (m, 3H), 7.22 (dd, *J* = 5.4, 3.4 Hz, 2H), 7.18 – 7.13 (m, 1H), 7.11 – 7.07 (m, 3H), 7.06 – 6.96 (m, 5H), 6.94 – 6.87 (m, 1H), 6.35 (s, 1H), 2.55 (s, 2H), 1.94 (s, 3H), 1.34 (s, 6H); **^{13}C NMR** (100 MHz, $CDCl_3$, This compound contains many sp^2-carbons, it's difficult to recognize the C-F couplings. So we list all the signals here.) δ 175.9, 160.3, 157.3, 156.9, 152.7, 141.5, 138.1, 135.0, 130.1, 129.8, 129.8, 128.9, 128.8, 128.3, 128.3, 128.1, 128.0, 128.0, 126.2, 126.0, 123.6, 123.5, 118.9, 118.8, 118.5, 112.2, 112.2, 112.1, 112.0, 50.2, 42.5, 26.3, 20.9; **^{19}F NMR** (376 MHz, $CDCl_3$) δ -125.7 – -125.9 (m, 1F), -127.9 – -128.1 (m, 1F), -158.7 – -158.8 (m, 1F). HRMS (ESI) m/z calcd. for $C_{33}H_{28}F_3NNaO_3$ $[M+Na]^+$ = 566.1913, found = 566.1902.

(4*E*)-1-([1,1'-Biphenyl]-4-yl)-2,2,4-trimethyl-5-phenylpent-4-en-1-one *O*-(2,3,4-trifluorobenzoyl) oxime (1aj)

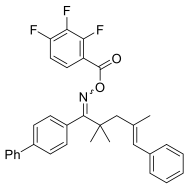

The reaction was performed following **Typical Procedure B** with **SI-3** (7 mmol) to give the product 1aj (1.18 g, 32% overall yield in 3 steps) as white solid, m.p. 105.5-105.9 °C, **^{1}H NMR** (400 MHz, $CDCl_3$) δ 7.56 – 7.37 (m, 5H), 7.29 (d, *J* = 7.5 Hz, 2H), 7.22 – 7.13 (m, 4H), 7.10 – 7.07 (m, 2H), 7.06 – 7.02 (m, 2H), 6.71 – 6.65 (m, 1H), 6.21 (s, 1H), 2.43 (s, 2H), 1.81 (d, *J* = 1.3 Hz, 3H), 1.21 (s, 6H); **^{13}C NMR** (100 MHz, $CDCl_3$, This compound contains many sp^2-carbons, it's difficult to recognize the C-F couplings. So we list all the signals here.) δ 176.2, 141.3, 140.2, 138.2, 135.1, 132.1, 130.2, 129.8, 128.9, 128.9, 128.1, 128.1, 127.7, 127.2, 127.2, 127.0, 126.7, 126.5, 126.2, 126.0, 125.9, 112.1, 112.0, 111.9, 50.3, 42.5, 26.4, 20.9; **^{19}F NMR** (376 MHz, $CDCl_3$) δ -125.9 – -126.0 (m, 1F), -128.4 – -128.5 (m, 1F), -158.4 – -158.5 (m, 1F). HRMS (ESI) m/z calcd. for $C_{33}H_{28}F_3NNaO_2$ $[M+Na]^+$ = 550.1964, found = 550.1964.

(4*E*)-1-(2,3-Dihydrobenzo[*b*][1,4]dioxin-6-yl)-2,2,4-trimethyl-5-phenylpent-4-en-1-one *O*-(2,3,4-trifluorobenzoyl) oxime (1ak)

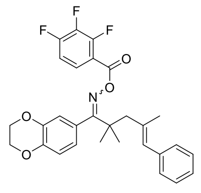

The reaction was performed following **Typical Procedure B** with **SI-3** (4 mmol) to give the product **1ak** (631.4 mg, 31% overall yield in 3 steps) as colorless oil, **^{1}H NMR** (400 MHz, $CDCl_3$) δ 7.51 – 7.42 (m, 1H), 7.33 (t, J = 7.6 Hz, 2H), 7.29 – 7.27 (m, 2H), 7.23 – 7.14 (m, 1H), 6.99 – 6.91 (m, 2H), 6.71 (d, J = 2.0 Hz, 1H), 6.64 (dd, J = 8.3, 2.0 Hz, 1H), 6.38 (s, 1H), 4.30 (s, 4H), 2.58 (s, 2H), 1.98 (s, 3H), 1.37 (s, 6H); **^{13}C NMR** (100 MHz, $CDCl_3$, This compound contains many sp^2-carbons, it's difficult to recognize the C-F couplings. So we list all the signals here.) δ 175.7, 160.3, 152.8, 143.6, 143.1, 138.2, 135.2, 130.0, 129.7, 128.9, 128.8, 128.1, 128.0, 126.2, 126.1, 126.1, 126.0, 120.1, 116.9, 116.8, 115.9, 112.1, 112.1, 112.0, 111.9, 64.4, 64.3, 50.2, 42.5, 26.4, 20.8; **^{19}F NMR** (376 MHz, $CDCl_3$) δ -126.1 – -126.2 (m, 1F), -128.3 – -128.5 (m, 1F), -158.6 – -158.7 (m, 1F). HRMS (ESI) m/z calcd. for $C_{29}H_{26}F_3NNaO_4$ [M+Na]$^+$ = 532.1706, found = 532.1709.

(4*E*)-2,2,4-Trimethyl-5-phenyl-1-(*m*-tolyl)pent-4-en-1-one *O*-(2,3,4-trifluorobenzoyl) oxime (1al)

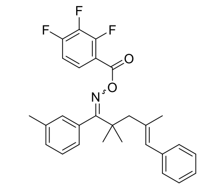

The reaction was performed following **Typical Procedure B** with **SI-3** (8 mmol) to give the product **1al** (1.04 g, 28% overall yield in 3 steps) as colorless oil, **^{1}H NMR** (400 MHz, $CDCl_3$) δ 7.69 – 7.60 (m, 4H), 7.59 – 7.53 (m, 3H), 7.52 – 7.47 (m, 2H), 7.25 – 7.13 (m, 2H), 6.68 (s, 1H), 2.88 (s, 2H), 2.68 (s, 3H), 2.28 (s, 3H), 1.66 (s, 6H); **^{13}C NMR** (100 MHz, $CDCl_3$, This compound contains many sp^2-carbons, it's difficult to recognize the C-F couplings. So we list all the signals here.) δ 176.4, 160.2, 152.6, 139.0, 138.2, 137.7, 135.1, 133.1, 130.1, 129.7, 129.1, 129.0, 128.9, 128.8, 128.3, 128.1, 128.0, 127.8, 127.8, 127.1, 126.1, 126.0, 126.0, 125.9, 125.9, 123.7, 112.1, 112.1, 111.9, 111.9, 111.9, 50.2, 42.4, 26.4, 21.4, 20.9; **^{19}F NMR** (376 MHz, $CDCl_3$) δ -126.1 – -126.2 (m, 1F), -128.6 – -128.8 (m, 1F), -158.6 – -158.8 (m, 1F). HRMS (ESI) m/z calcd. for $C_{28}H_{26}F_3NNaO_2$ [M+Na]$^+$ = 488.1808, found = 488.1815.

(4*E*)-1-(3-Methoxyphenyl)-2,2,4-trimethyl-5-phenylpent-4-en-1-one *O*-(2,3,4-trifluorobenzoyl) oxime (1am)

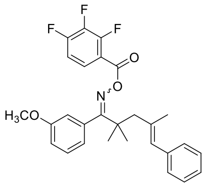

The reaction was performed following **Typical Procedure B** with **SI-3** (5 mmol) to give the product 1am (794.0 mg, 33% overall yield in 3 steps) as colorless oil, **^{1}H NMR** (400 MHz, $CDCl_3$) δ 7.41 – 7.31 (m, 4H), 7.30 – 7.26 (m, 2H), 7.22 – 7.15 (m, 1H), 6.97 – 6.87 (m, 2H), 6.79 – 6.67 (m, 2H), 6.39 (s, 1H), 3.80 (s, 3H), 2.59 (s, 2H), 1.99 (s, 3H), 1.37 (s, 6H); **^{13}C NMR** (100 MHz, $CDCl_3$, This compound contains many sp^2-carbons, it's difficult to recognize the C-F couplings. So we list all the signals here.) δ 176.1, 159.2, 138.2, 135.1, 134.3, 130.2, 129.8, 129.2, 128.9, 128.8, 128.1, 128.0, 126.2, 126.1, 126.0, 119.0, 113.7, 112.6, 112.2, 112.1, 112.0, 111.9, 55.3, 50.2, 42.4, 26.4, 20.9; **^{19}F NMR** (376 MHz, $CDCl_3$) δ -126.0 – -126.1 (m, 1F), -128.5 – -128.6 (m, 1F), -158.5 – -158.6 (m, 1F). HRMS (ESI) m/z calcd. for $C_{28}H_{26}F_3NNaO_3$ $[M+Na]^+$ = 504.1757, found = 504.1765.

4-Benzylidene-2,2-dimethyl-1-phenylhexan-1-one *O*-(2,3,4-trifluorobenzoyl) oxime (1an)

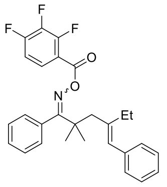

The reaction was performed following **Typical Procedure B** with **SI-3** (10 mmol) to give the product 1am (565.2 mg, 15% overall yield in 3 steps) as white solid, m.p. 66.0-66.9 °C, **^{1}H NMR** (400 MHz, $CDCl_3$) δ 7.52 – 7.47 (m, 3H), 7.44 – 7.37 (m, 3H), 7.33 – 7.27 (m, 3H), 7.26 – 7.21 (m, 2H), 6.97 (ddd, *J* = 9.0, 6.7, 2.2 Hz, 1H), 6.48 (s, 1H), 2.62 (s, 2H), 2.40 (q, *J* = 7.5 Hz, 2H), 1.45 (s, 6H), 1.13 (t, *J* = 7.5 Hz, 3H); **^{13}C NMR** (100 MHz, $CDCl_3$, This compound contains many sp^2-carbons, it's difficult to recognize the C-F couplings. So we list all the signals here.) δ 176.4, 141.1, 138.2, 133.2, 129.4, 128.6, 128.4, 128.1, 128.0, 127.9, 126.6, 126.4, 126.2, 126.0, 125.9, 112.1, 112.1, 111.9, 111.9, 45.0, 42.4, 26.4, 25.6, 13.3; **^{19}F NMR** (376 MHz, $CDCl_3$) δ -126.0 – -126.2 (m, 1F), -128.5 – -128.3 (m, 1F), -158.6 – -158.7 (m, 1F). HRMS (ESI) m/z calcd. for $C_{28}H_{26}F_3NNaO_2$ $[M+Na]^+$ = 488.1808, found = 488.1817.

4-Benzylidene-2,2-dimethyl-1-phenylhexan-1-one *O*-(2,3,4-trifluorobenzoyl) oxime (1ao)

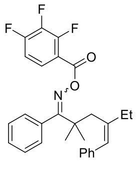

The reaction was performed following **Typical Procedure B** with **SI-3** (20 mmol) to give the product 1am (930.0 mg, 10% overall yield in 3 steps) as yellow oil, **^{1}H NMR** (400 MHz, $CDCl_3$) δ 7.39 – 7.31 (m, 4H), 7.26 – 7.20 (m, 2H), 7.17 – 7.12 (m, 3H), 7.06 – 7.00 (m, 2H), 6.91 – 6.84 (m, 1H), 6.49 (s, 1H), 2.78 (s,

2H), 2.35 – 2.29 (m, 2H), 1.20 – 1.12 (m, 9H); **^{13}C NMR** (100 MHz, $CDCl_3$, This compound contains many sp^2-carbons, it's difficult to recognize the C-F couplings. So we list all the signals here.) δ 176.5, 152.9, 141.5, 139.0, 133.1, 128.9, 128.3, 128.1, 127.9, 127.9, 126.6, 126.0, 125.9, 112.1, 112.1, 111.9, 111.9, 42.7, 38.3, 31.6, 26.5, 13.4; **^{19}F NMR** (376 MHz, $CDCl_3$) δ -126.0 – -126.1 (m, 1F), -128.6 – -128.7 (m, 1F), -158.6 – -158.7 (m, 1F). HRMS (ESI) m/z calcd. for $C_{28}H_{26}F_3NNaO_2$ $[M+Na]^+$ = 488.1808, found = 488.1819.

3. Optimization of the reaction conditions

3.1 Table S1. Optimization of chiral catalyst[a]

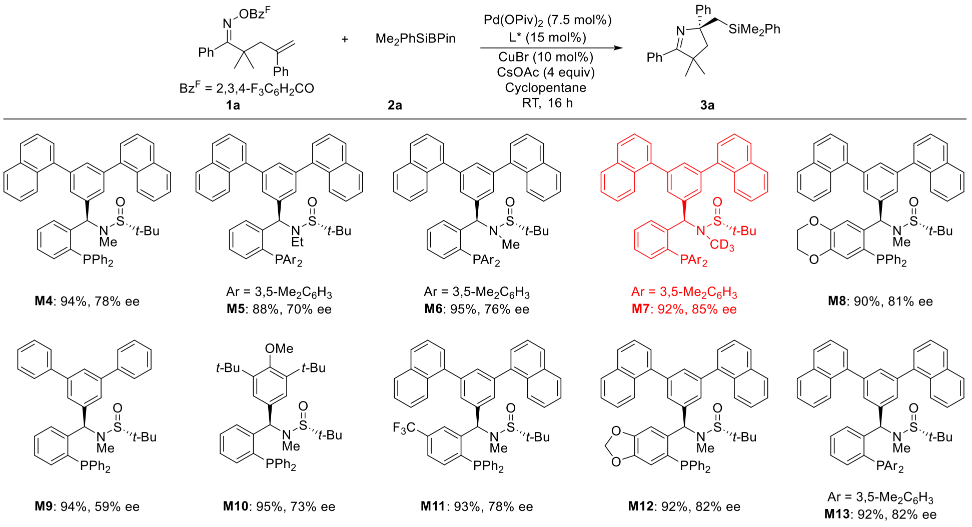

[a]Unless otherwise noted, all reactions were carried out with 0.35 mmol of **2a**, 0.1 mmol of **1a**, and 7.5 mol% of Pd(OPiv)$_2$, **L*** (15 mol%) in 1.0 mL cyclopentane at RT for 16 h; yield was determined by GC, ee was determined by HPLC analysis.

3.2 Table S2. Optimization of Palladium Salts[a]

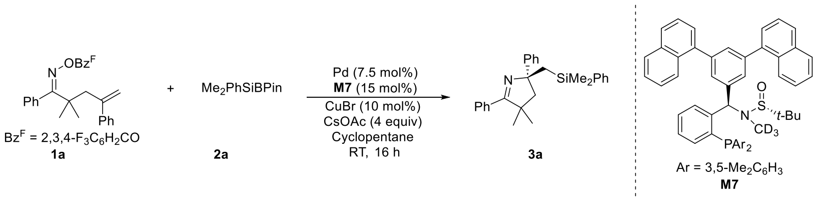

Entry	Pd (mol%)	Yield (%)	*Ee* (%)
1	$Pd_2(dba)_3$	77	85
2	$Pd(dba)_2$	77	86
3	$Pd(OPiv)_2$	81	84
4	$Pd(TFA)_2$	76	84
5	$Pd_2(dba)_3{\cdot}CHCl_3$	79	86
6	$[Pd(allyl)Cl_2]$	70	82
7	$Pd(OAc)_2$	77	83
8	$PdCl_2$	N.R	N.A

[a]Unless otherwise noted, all reactions were carried out with 0.35 mmol of **2a**, 0.1 mmol of **1a**, and 7.5 mol% of Pd, **M7** (15 mol%) in 1.0 mL cyclopentane at RT for 16 h; yield was determined by GC, ee was determined by HPLC analysis. N.R. = no reaction. N.A. = not available.

3.3 Table S3. Optimization of Copper Salts[a]

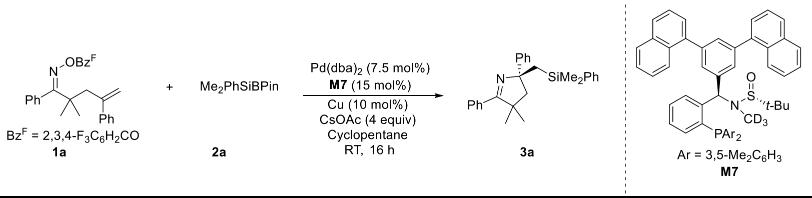

Entry	Cu (mol%)	Yield (%)	*Ee* (%)
1	$Cu(acac)_2$	68	77
2	$CuSO_4$	69	84
3	CuCl	80	83
4	$CuBr_2$	83	83

5	$Cu(OAc)_2$	74	82
6	CuTc	41	82
7	$CuBr{\cdot}SMe_2$	78	86
8	CuOAc	64	82
9	CuI	76	83

[a]Unless otherwise noted, all reactions were carried out with 0.35 mmol of **2a**, 0.1 mmol of **1a**, and 7.5 mol% of $Pd(dba)_2$, **M7** (15 mol%) in 1.0 mL cyclopentane at RT for 16 h; yield was determined by GC, ee was determined by HPLC analysis.

3.4 Table S4. Optimization of Palladium Salts and equivalents and temperature[a]

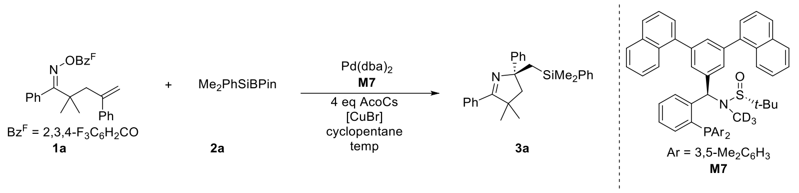

Entry	a:b	CuBr (mol%)	$Pd(dba)_2$ (mol%)	L*(mol%)	T/°C	Yield (%)	*Ee* (%)
1	1:3.5	10	7.5	15	15	82	76
2	1:3.5	10	7.5	15	0	77	88
3	1:3.5	10	7.5	15	-20	63	69
4	1:3.5	10	7.5	15	-40	/	/
5	1:3.5	No	7.5	15	0	27	85
6	1:3.5	5	7.5	15	0	32	85
7	1:3.5	15	7.5	15	0	69	88
8	1:3.5	20	7.5	15	0	57	87
9	1:3.5	30	5	7.5	0	27	87
10	1:1.5	10	7.5	15	0	37	88
11	1:2	10	7.5	15	0	48	88
12	1:2.5	10	7.5	15	0	70	90
13	1:3	10	7.5	15	0	78	88
14	1:2.5	10	3.75	7.5	0	78	90
15	1:2.5	10	5	10	0	75	91
16	1:2.5	10	10	20	0	83	90

[a]Unless otherwise noted, all reactions were carried out with 0.1 mmol of **1a**, yield was determined by GC, ee was determined by HPLC analysis.

3.5 Table S5. Optimization of chiral catalyst[a]

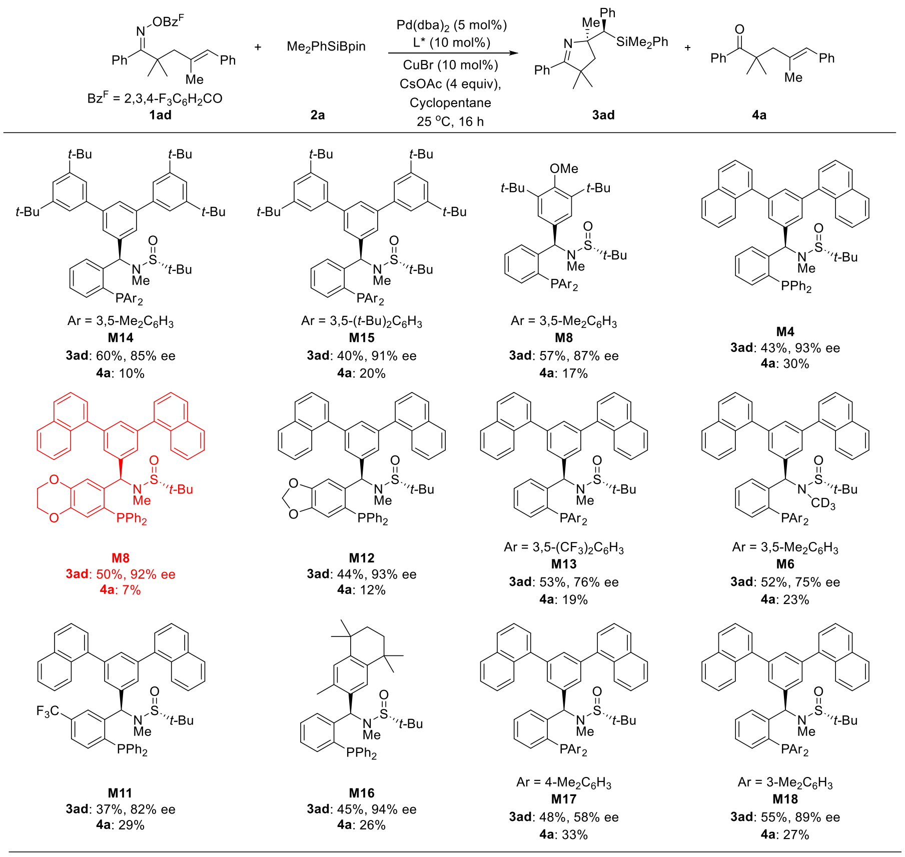

[a]Unless otherwise noted, all reactions were carried out with 0.25 mmol of **2a**, 0.1 mmol of **1ad**, and 5 mol% of $Pd(dba)_2$, **L*** (10 mol%) in 1.0 mL cyclopentane at 25 °C for 16 h; yield was determined by GC, ee was determined by HPLC analysis.

3.6 Table S6. Optimization of Palladium Salts[a]

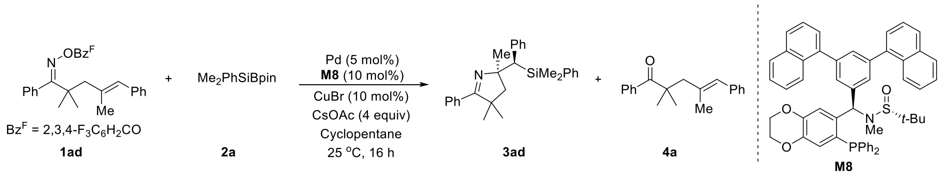

Entry	Pd (mol%)	**3ad**/Yield (%)	**3ad**/*Ee* (%)	**4a**/Yield (%)
1	$Pd_2(dba)_3$	42	93	13
2	$Pd(dba)_2$	77	86	9
3	$Pd(TFA)_2$	48	92	14
4	$Pd_2(dba)_3{\cdot}CHCl_3$	51	94	17
5	$[Pd(allyl)Cl_2]$	66	94	5
6	$Pd(OAc)_2$	67	93	7
7	$PdCl_2$	N.R	N.A	N.A
8	$Pd(OPiv)_2$	51	92	14

[a]Unless otherwise noted, all reactions were carried out with 0.25 mmol of **2a**, 0.1 mmol of **1ad**, and 5 mol% of Pd, **M8** (10 mol%) in 1.0 mL cyclopentane at 25 °C for 16 h; yield was determined by GC, ee was determined by HPLC analysis.

3.7 Table S7. Optimization of Base[a]

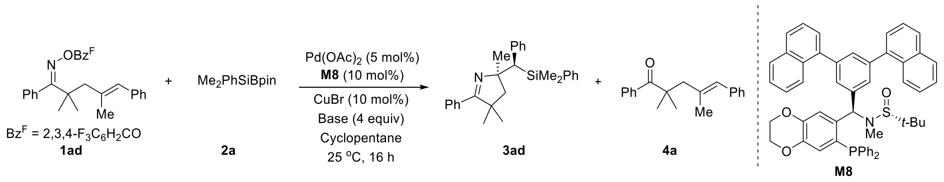

Entry	Base (equiv)	**3ad**/Yield (%)	**3ad**/*Ee* (%)	**4a**/Yield (%)
1	Cs_2CO_3	61	92	8
2	K_3PO_4	60	92	9
3	Na_2CO_3	63	90	8
4	Rb_2CO_3	70	90	6
5	DIPEA	/	/	/
6	NaOH	/	/	/
7	DABCO	36	91	7
8	$NaHCO_3$	67	89	11
9	TMSOK	/	/	/
10	*t*BuOLi	/	/	/
11	AcONa	11	80	5

[a]Unless otherwise noted, all reactions were carried out with 0.25 mmol of **2a**, 0.1 mmol of **1ad**, and 5 mol% of Pd, **M8** (10 mol%) in 1.0 mL cyclopentane at 25 °C for 16 h; yield was determined by GC, ee was determined by HPLC analysis.

3.8 Table S8. Optimization of Base equivalents [a]

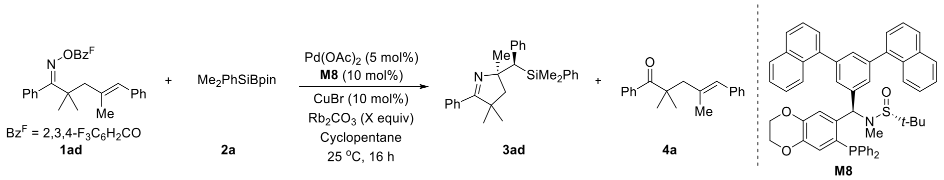

Entry	Rb_2CO_3 (equiv)	**3ad**/Yield (%)	**3ad**/*Ee* (%)	**4a**/Yield (%)
1	1.5	68	90	10
2	2	75(69)	93	7
3	2.5	71	90	11
4	3	73	91	11
5	3.5	70	92	6

[a]Unless otherwise noted, all reactions were carried out with 0.25 mmol of **2a**, 0.1 mmol of **1ad**, and 5 mol% of Pd, **M8** (10 mol%) in 1.0 mL cyclopentane at 25 °C for 16 h; yield was determined by GC, ee was determined by HPLC analysis.

3.9 Figure S1. Failed substrates (No reaction/<70% ee):

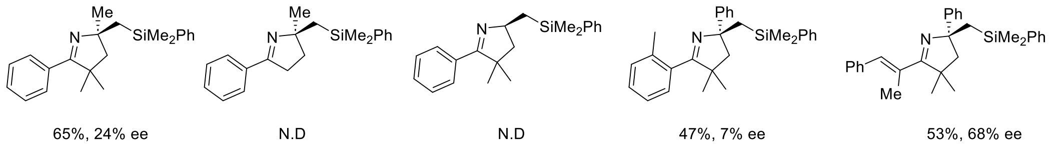

4. General experimental procedures and characterization of products

4.1 General procedure for enantioselective Aza-Heck Cyclization/Silylation Reactions

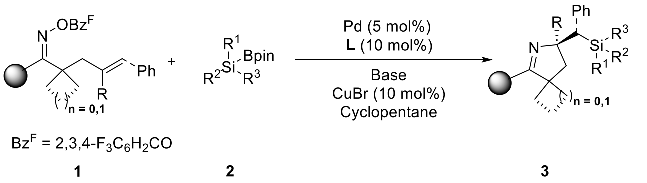

General procedure A: Under nitrogen atmosphere, to an oven-dried 10 mL Schlenk tube equipped with a magnetic stir was added $Pd(dba)_2$ (8.5 mg, 0.015 mmol, 5 mol%), ligand **M6** (23.9 mg, 0.03 mmol, 10 mol%) and cyclopentane (3 mL). The catalyst/ligand solution was stirred for 1.0 h at 25 °C, CsOAc (230.3 mg, 1.20 mmol, 4.0 equiv), oxime esters **1** (0.30 mmol, 1.0 equiv), silylboronic ester **2** (0.75 mmol, 2.5 equiv) were added successively. The resulting mixture was then stirred vigorously at 0 °C for about 40 h. After completion of the reaction (monitored by TLC), the reaction mixture was concentrated to dryness and the residue was purified by column chromatography (petroleum ether/ethyl acetate) to afford desired product **3a-3ac**.

General procedure B: Under nitrogen atmosphere, to an oven-dried 10 mL Schlenk tube equipped with a magnetic stir was added $Pd(OAc)_2$ (3.3 mg, 0.015 mmol, 5 mol%), ligand **M7** (24 mg, 0.03 mmol, 10 mol%) and cyclopentane (3 mL). The catalyst/ligand solution was stirred for 1.0 h at 25 °C, Rb_2CO_3 (90.0 mg, 0.6 mmol, 2.0 equiv) or CsOAc (230.3 mg, 1.20 mmol, 4.0 equiv), oxime esters **1** (0.30 mmol, 1.0 equiv), silylboronic ester **2a** (0.75 mmol, 2.5 equiv) and CuBr (4.2 mg, 0.03 mmol, 10 mol%) were added successively. The resulting mixture was then stirred vigorously at 25 °C for about 30 h. After completion of the reaction (monitored by TLC), the reaction mixture was concentrated to dryness and the residue was purified by column chromatography (petroleum ether/ethyl acetate) to afford desired product **3ad-3an**.

Note*: we tried the Phosphine ligands (such as PPh_3, PCy_3, XPhos, SPhos), but it is weird that the system was messy and we couldn't even get the product. So, we used a pair of enantiomers of Ming-Phos (**M6**) with close to 1:1 ratio to get the product **3** with low ee, which was used to determine the two peaks of two enantiomers of **3** in HPLC. In addition, we also measured the ee value of the mixed Ming-Phos (**M6**) enantiomers mentioned above with 31% ee by HPLC.*

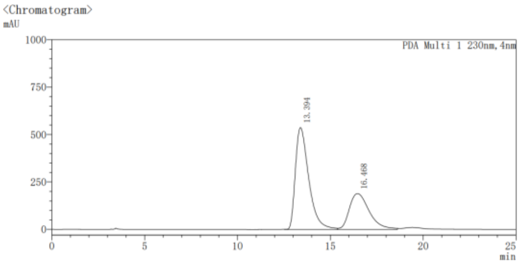

<Peak Table>
PDA Ch1 230nm

No.	Ret. Time (min)	Height (mAU)	Height%	Area (mAU*min)	Area%
1	13.394	537461	73.950	26961299	65.625
2	16.468	189332	26.050	14122544	34.375
Total		726793	100.000	41083843	100.000

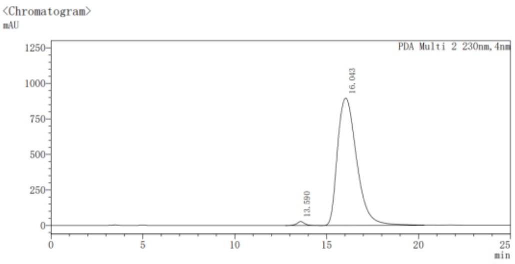

<Peak Table>
PDA Ch2 230nm

No.	Ret. Time (min)	Height (mAU)	Height%	Area (mAU*min)	Area%
1	13.590	27972	3.025	879087	1.326
2	16.043	896597	96.975	65397723	98.674
Total		924569	100.000	66276810	100.000

4.2 Characterization of products

(*S*)-2-((Dimethyl(phenyl)silyl)methyl)-4,4-dimethyl-2,5-diphenyl-3,4-dihydro-2*H*-pyrrole (3a)

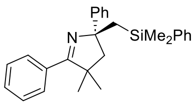

Following **General procedure A**, isolated **3a** 103.7 mg, 87% yield, 91% ee, as colorless oil, $[\alpha]_D^{20}$ = -147.9 (c = 2.6, $CHCl_3$). **^{1}H NMR** (400 MHz, $CDCl_3$) δ 7.82 – 7.72 (m, 2H), 7.40 – 7.31 (m, 7H), 7.26 – 7.16 (m, 5H), 7.15 – 7.06 (m, 1H), 2.30 (d, *J* = 12.8 Hz, 1H), 2.17 (d, *J* = 12.7 Hz, 1H), 1.76 (d, *J* = 14.5 Hz, 1H), 1.54 (d, *J* = 14.5 Hz, 1H), 1.30 (s, 3H), 1.05 (s, 3H), 0.18 (s, 3H), -0.06 (s, 3H); **^{13}C NMR** (100 MHz, $CDCl_3$) δ 175.9, 150.5, 140.8, 134.8, 133.5, 129.3, 128.4, 128.3, 128.1, 127.9, 127.5, 125.9, 125.8, 76.1, 58.3, 50.7, 35.4, 28.0, 27.4, -1.2, -1.8. HRMS (ESI) m/z calcd. for $C_{27}H_{31}NNaSi$ $[M+Na]^+$ = 420.2118, found = 420.2121.

HPLC conditions: Daicel Chiralpak OZ-3 column; hexane/2-propanol = 100/0, 0.7 mL/min, 25 ^oC. Retention times: 9.44 min (major), 13.08 min (minor).

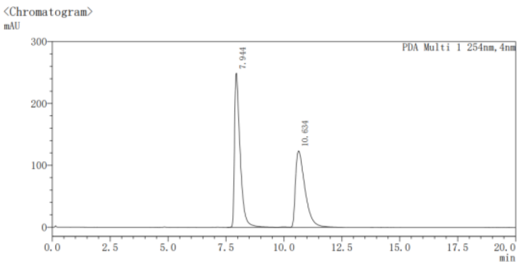

<Peak Table>
PDA Ch1 254nm

No.	Ret. Time (min)	Height (mAU)	Height%	Area (mAU*min)	Area%
1	7.944	249126	66.841	4212497	54.428
2	10.634	123590	33.159	3527024	45.572
Total		372716	100.000	7739520	100.000

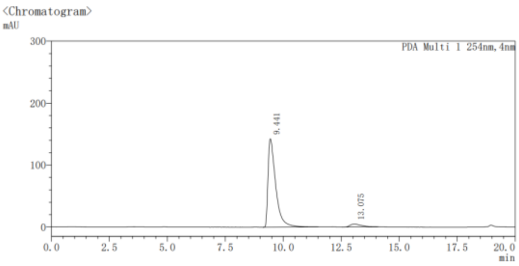

<Peak Table>
PDA Ch1 254nm

No.	Ret. Time (min)	Height (mAU)	Height%	Area (mAU*min)	Area%
1	9.441	142713	96.727	3296276	95.311
2	13.075	4829	3.273	162157	4.689
Total		147541	100.000	3458433	100.000

(*S*)-2-((Dimethyl(phenyl)silyl)methyl)-4,4-dimethyl-2-phenyl-5-(*p*-tolyl)-3,4-dihydro-2*H*-pyrrole (3b)

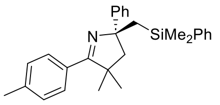

Following **General procedure A**, isolated **3b** 96.2 mg, 78% yield, 93% ee, as colorless oil, $[\alpha]_D^{20}$ = -183.1 (c = 2.4, $CHCl_3$). **^{1}H NMR** (400 MHz, $CDCl_3$) δ 7.88 (d, *J* = 7.6 Hz, 2H), 7.66 – 7.51 (m, 4H), 7.50 – 7.27 (m, 8H), 2.53 (s, 3H), 2.47 (d, *J* =

12.8 Hz, 1H), 2.35 (d, *J* = 12.8 Hz, 1H), 1.94 (d, *J* = 14.9 Hz, 1H), 1.71 (d, *J* = 14.9 Hz, 1H), 1.49 (s, 3H), 1.23 (s, 3H), 0.37 (s, 3H), 0.12 (s, 3H); **^{13}C NMR** (100 MHz, $CDCl_3$) δ 175.5, 150.7, 140.9, 139.4, 133.5, 131.9, 128.8, 128.4, 128.3, 127.9, 127.5, 125.8, 75.9, 58.6, 50.6, 35.4, 28.1, 27.4, 21.3, -1.2, -1.7. HRMS (ESI) m/z calcd. for $C_{28}H_{33}NNaSi$ $[M+H]^+$ = 434.2274, found = 434.2279.

HPLC conditions: Daicel Chiralpak OZ-3 column; hexane/2-propanol = 100/0, 0.5 mL/min, 25 °C. Retention times: 12.18 min (major), 13.73 min (minor).

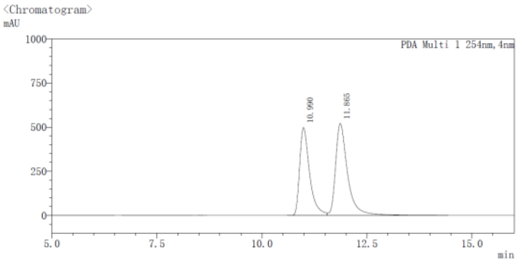

<Peak Table>
PDA Ch1 254nm

No.	Ret.Time(min)	Height(mAU)	Height%	Area(mAU*min)	Area%
1	10.990	497817	48.866	8202034	44.292
2	11.865	520916	51.134	10316092	55.708
Total		1018733	100.000	18518126	100.000

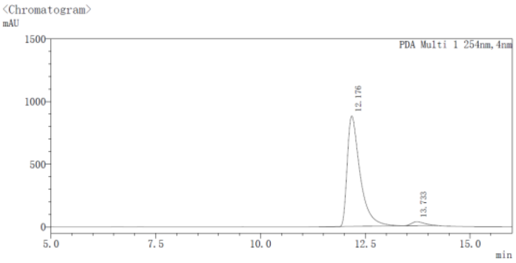

<Peak Table>
PDA Ch1 254nm

No.	Ret.Time(min)	Height(mAU)	Height%	Area(mAU*min)	Area%
1	12.176	880739	96.569	18829725	96.431
2	13.733	31295	3.431	696915	3.569
Total		912034	100.000	19526640	100.000

(*S*)-5-(4-(*tert*-Butyl)phenyl)-2-((dimethyl(phenyl)silyl)methyl)-4,4-dimethyl-2-phenyl-3,4-dihydro-2*H*-pyrrole (3c)

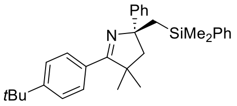

Following **General procedure A**, use **M8**, at -10 °C, isolated **3c** 114.2 mg, 84% yield, 91% ee, as colorless oil, $[\alpha]_D^{20}$ = -213.5 (c = 2.9, $CHCl_3$). **^{1}H NMR** (400 MHz, $CDCl_3$) δ 7.87 (d, J = 8.2 Hz, 2H), 7.52 – 7.43 (m, 6H), 7.37 – 7.26 (m, 5H), 7.18 (t, *J* = 7.3 Hz, 1H), 2.39 (d, *J* = 12.8 Hz, 1H), 2.27 (d, *J* = 12.8 Hz, 1H), 1.86 (d, *J* = 14.6 Hz, 1H), 1.61 (d, *J* = 14.6 Hz, 1H), 1.41 (d, *J* = 4.4 Hz, 12H), 1.16 (s, 3H), 0.30 (s, 3H), 0.03 (s, 3H); **^{13}C NMR** (100 MHz, $CDCl_3$) δ 175.4, 152.5, 150.7, 141.0, 133.5, 131.8, 128.3, 128.1, 127.8, 127.5, 125.8, 125.0, 75.9, 58.8, 50.6, 35.5, 31.2, 28.2, 27.5, -1.1, -1.8. HRMS (ESI) m/z calcd. for $C_{31}H_{39}NNaSi$ $[M+Na]^+$ = 476.2744, found = 476.2747.

HPLC conditions: Daicel Chiralpak OZ-3-OZ-3 column; hexane/2-propanol = 100/0, 0.7 mL/min, 25 °C. Retention times: 14.88 min (major), 16.45 min (minor).

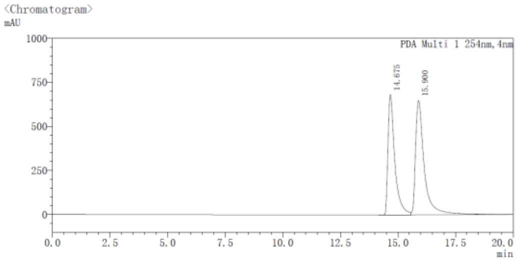

<Peak Table>
PDA Ch1 254nm

No.	Ret. Time(min)	Height(mAU)	Height%	Area(mAU*min)	Area%
1	14.675	681217	51.237	13976563	44.827
2	15.900	648322	48.763	17202056	55.173
Total		1329538	100.000	31178618	100.000

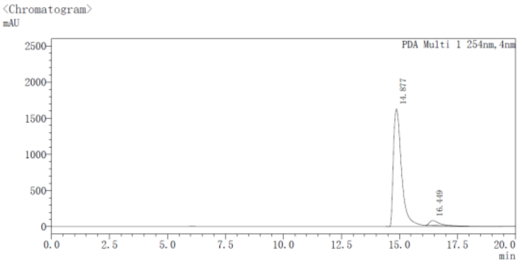

<Peak Table>
PDA Ch1 254nm

No.	Ret. Time(min)	Height(mAU)	Height%	Area(mAU*min)	Area%
1	14.877	1627865	95.956	41613023	95.373
2	16.449	68600	4.044	2018786	4.627
Total		1696466	100.000	43631809	100.000

(*S*)-2-((Dimethyl(phenyl)silyl)methyl)-4,4-dimethyl-5-(4-phenoxyphenyl)-2-phenyl-3,4-dihydro-2*H*-pyrrole (3d)

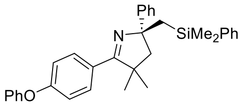

Following **General procedure A**, isolated **3d** 92.5 mg, 63% yield, 88% ee, as colorless oil, $[\alpha]_D^{20}$ = -57.8 (c = 2.3, $CHCl_3$). **^{1}H NMR** (400 MHz, $CDCl_3$) δ 7.87 (d, *J* = 8.7 Hz, 2H), 7.51 – 7.37 (m, 6H), 7.34 – 7.26 (m, 5H), 7.19 (d, *J* = 7.3 Hz, 2H), 7.11 (d, *J* = 8.6 Hz, 2H), 7.03 (d, *J* = 7.6 Hz, 2H), 2.38 (d, *J* = 12.8 Hz, 1H), 2.26 (d, *J* = 12.7 Hz, 1H), 1.84 (d, *J* = 14.6 Hz, 1H), 1.60 (d, *J* = 14.9 Hz, 1H), 1.40 (s, 3H), 1.14 (s, 3H), 0.25 (s, 3H), 0.02 (s, 3H); **^{13}C NMR** (100 MHz, $CDCl_3$) δ 174.8, 158.6, 156.6, 150.6, 140.9, 133.5, 130.0, 129.8, 129.5, 128.4, 127.9, 127.5, 125.8, 125.8, 123.7, 119.5, 117.8, 75.8, 58.7, 50.5, 35.4, 28.1, 27.5, -1.2, -1.7. HRMS (ESI) m/z calcd. for $C_{33}H_{36}NOSi$ $[M+H]^+$ = 490.2561, found = 490.2571.

HPLC conditions: Daicel Chiralpak ADH-ADH column; hexane/2-propanol = 99.5/0.5, 0.3 mL/min, 25 ^oC. Retention times: 34.06 min (major), 36.05 min (minor).

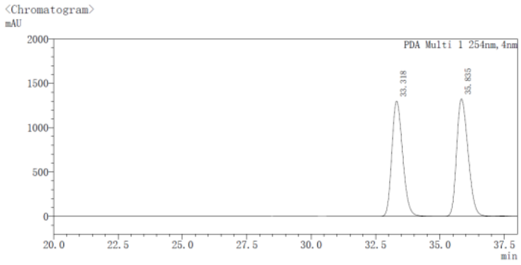

<Peak Table>
PDA Ch1 254nm

No.	Ret. Time(min)	Height(mAU)	Height%	Area(mAU*min)	Area%
1	33.318	1300728	49.547	38094389	48.180
2	35.835	1324519	50.453	40972687	51.820
Total		2625247	100.000	79067076	100.000

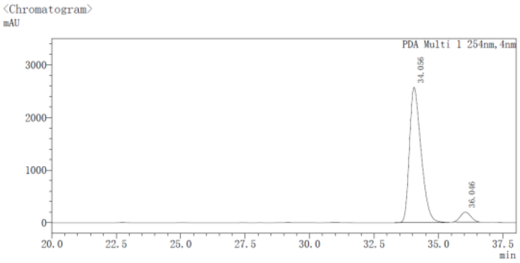

<Peak Table>
PDA Ch1 254nm

No.	Ret. Time(min)	Height(mAU)	Height%	Area(mAU*min)	Area%
1	34.056	2573379	93.175	80934899	93.807
2	36.046	188503	6.825	5343488	6.193
Total		2761882	100.000	86278388	100.000

(*S*)-5-([1,1'-Biphenyl]-4-yl)-2-((dimethyl(phenyl)silyl)methyl)-4,4-dimethyl-2-phenyl-3,4-dihydro-2*H*-pyrrole (3e)

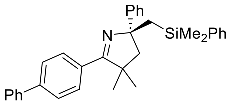

Following **General procedure A**, isolated **3e** 130.6 mg, 92% yield, 90% ee, as colorless oil, $[\alpha]_D^{20}$ = -262.3 (c = 3.3, $CHCl_3$). **^{1}H NMR** (400 MHz, $CDCl_3$) δ 7.96 (d, *J* = 8.3 Hz, 2H), 7.71 – 7.63 (m, 4H), 7.54 – 7.44 (m, 6H), 7.43 – 7.38 (m,

1H), 7.36 – 7.26 (m, 5H), 7.23 – 7.15 (m, 1H), 2.41 (d, *J* = 12.8 Hz, 1H), 2.28 (d, *J* = 12.8 Hz, 1H), 1.86 (d, *J* = 14.6 Hz, 1H), 1.64 (d, *J* = 14.6 Hz, 1H), 1.43 (s, 3H), 1.18 (s, 3H), 0.28 (s, 3H), 0.04 (s, 3H); **^{13}C NMR** (100 MHz, $CDCl_3$) δ 175.4, 150.6, 142.1, 140.9, 140.6, 133.6, 128.8, 128.4, 127.9, 127.6, 127.5, 127.1, 126.8, 125.9, 125.8, 76.1, 58.6, 50.7, 35.4, 28.1, 27.5, -1.2, -1.7. HRMS (ESI) m/z calcd. for $C_{33}H_{35}NNaSi$ $[M+Na]^+$ = 496.2431, found = 496.2426.

HPLC conditions: Daicel Chiralpak ADH column; hexane/2-propanol = 99/1, 0.5 mL/min, 25 °C. Retention times: 9.09 min (major), 12.10 min (minor).

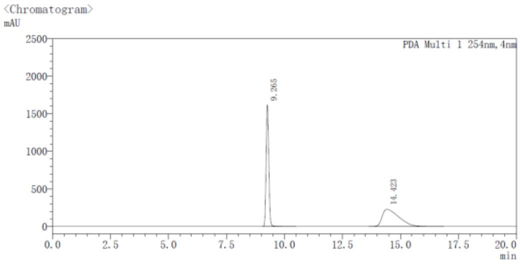

<Peak Table>
PDA Ch1 254nm

No.	Ret.Time(min)	Height(mAU)	Height%	Area(mAU*min)	Area%
1	9.265	1616804	87.626	12932794	53.259
2	14.423	228319	12.374	11350175	46.741
Total		1845123	100.000	24282969	100.000

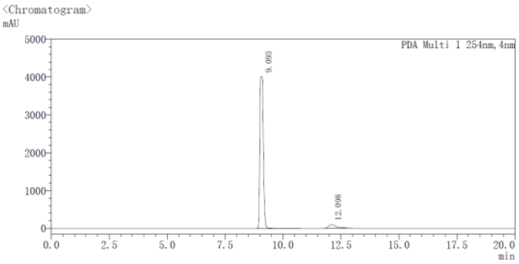

<Peak Table>
PDA Ch1 254nm

No.	Ret.Time(min)	Height(mAU)	Height%	Area(mAU*min)	Area%
1	9.093	3999621	97.654	41919018	95.024
2	12.098	96102	2.346	2195260	4.976
Total		4095723	100.000	44114278	100.000

(*S*)-2-((Dimethyl(phenyl)silyl)methyl)-4,4-dimethyl-2-phenyl-5-(4-(trifluoromethoxy)phenyl)-3,4-dihydro-2*H*-pyrrole (3f)

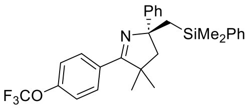

Following **General procedure A**, use **M8**, at -10 °C, isolated **3f** 98.2 mg, 68% yield, 87% ee, as colorless oil, $[\alpha]_D^{20}$ = -9.4 (c = 0.5, $CHCl_3$). **^{1}H NMR** (400 MHz, $CDCl_3$) δ 7.88 (d, *J* = 8.8 Hz, 2H), 7.52 – 7.41 (m, 4H), 7.37 – 7.26 (m, 7H), 7.24 – 7.19 (m, 1H), 2.41 (d, *J* = 12.9 Hz, 1H), 2.29 (d, *J* = 12.8 Hz, 1H), 1.86 (d, *J* = 14.6 Hz, 1H), 1.63 (d, *J* = 14.6 Hz, 1H), 1.40 (s, 3H), 1.15 (s, 3H), 0.25 (s, 3H), 0.04 (s, 3H); **^{13}C NMR** (100 MHz, $CDCl_3$) δ 174.5, 150.2, 140.7, 133.5, 133.3, 129.9, 128.4, 128.0, 127.5, 126.0, 125.7, 123.1 (q, $^1J_{C\text{-}F}$ = 262 Hz), 120.4, 76.2, 58.5, 50.6, 35.4, 28.0, 27.3, -1.3, -1.7; **^{19}F NMR** (376 MHz, $CDCl_3$) δ -57.7. HRMS (ESI) m/z calcd. for $C_{28}H_{31}F_3NOSi$ $[M+H]^+$ = 482.2122, found = 482.2119.

HPLC conditions: Daicel Chiralpak OZ-3-OZ-3 column; hexane/2-propanol = 100/0, 0.6 mL/min, 25 °C. Retention times: 8.29 min (major), 8.91 min (minor).

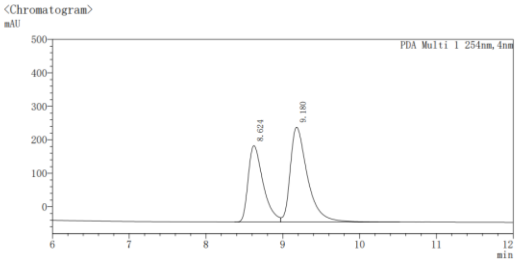

<Peak Table>
PDA Ch1 254nm

No.	Ret.Time(min)	Height(mAU)	Height%	Area(mAU*min)	Area%
1	8.624	228021	44.576	3093495	41.044
2	9.180	283510	55.424	4443575	58.956
Total		511531	100.000	7537070	100.000

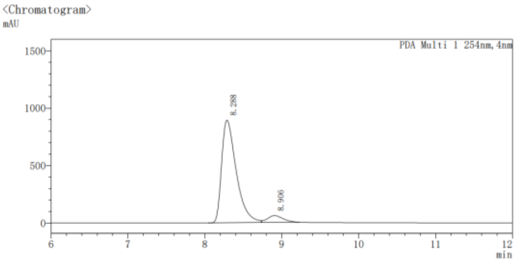

<Peak Table>
PDA Ch1 254nm

No.	Ret.Time(min)	Height(mAU)	Height%	Area(mAU*min)	Area%
1	8.288	894504	93.688	11804625	93.364
2	8.906	60267	6.312	839066	6.636
Total		954771	100.000	12643691	100.000

(*S*)-2-((Dimethyl(phenyl)silyl)methyl)-5-(4-methoxyphenyl)-4,4-dimethyl-2-phenyl-3,4-dihydro-2*H*-pyrrole (3g)

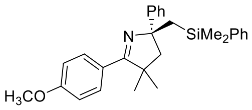

Following **General procedure A**, isolated **3g** 112.8 mg, 88% yield, 95% ee, as colorless oil, $[\alpha]_D^{20}$ = -86.5 (c = 1.0, $CHCl_3$). **^{1}H NMR** (400 MHz, $CDCl_3$) δ 7.80 (d, *J* = 8.8 Hz, 2H), 7.40 – 7.35 (m, 4H), 7.26 – 7.16 (m, 5H), 7.09 (t, *J* = 7.3 Hz, 1H), 6.87 (d, *J* = 8.7 Hz, 2H), 3.80 (s, 3H), 2.28 (d, *J* = 12.8 Hz, 1H), 2.17 (d, *J* = 12.8 Hz, 1H), 1.75 (d, *J* = 14.6 Hz, 1H), 1.52 (d, *J* = 14.6 Hz, 1H), 1.32 (s, 3H), 1.06 (s, 3H), 0.19 (s, 3H), 0.06 (s, 3H); **^{13}C NMR** (100 MHz, $CDCl_3$) δ 174.8, 160.6, 150.8, 141.0, 133.5, 129.9, 128.3, 127.8, 127.5, 127.2, 125.8, 113.4, 75.6, 58.9, 55.3, 50.4, 35.4, 28.2, 27.5, -1.2, -1.7. HRMS (ESI) m/z calcd. for $C_{28}H_{34}NOSi$ $[M+H]^+$ = 428.2404, found = 428.2406.

HPLC conditions: Daicel Chiralpak IA-IA column; hexane/2-propanol = 99.5/0.5, 0.3 mL/min, 25 ^{o}C. Retention times: 29.64 min (major), 28.87 min (minor).

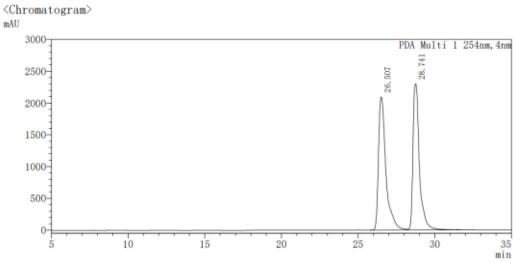

<Peak Table>
PDA Ch1 254nm

No.	Ret.Time(min)	Height(mAU)	Height%	Area(mAU*min)	Area%
1	26.507	2096495	47.679	70811094	51.211
2	28.741	2300603	52.321	67461254	48.789
Total		4397098	100.000	138272348	100.000

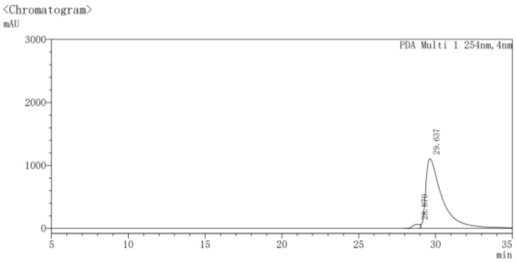

<Peak Table>
PDA Ch1 254nm

No.	Ret.Time(min)	Height(mAU)	Height%	Area(mAU*min)	Area%
1	28.870	66133	5.640	2159869	2.506
2	29.637	1106367	94.360	84013595	97.494
Total		1172500	100.000	86173464	100.000

(*S*)-2-((Dimethyl(phenyl)silyl)methyl)-4,4-dimethyl-2-phenyl-5-(4-(trifluoromethyl)phenyl)-3,4-dihydro-2*H*-pyrrole (3h)

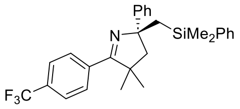

Following **General procedure A**, isolated **3h** 124.2 mg, 89% yield, 85% ee, as colorless oil, $[\alpha]_D^{20}$ = -188.9 (c = 3.1, $CHCl_3$). **^{1}H NMR** (400 MHz, $CDCl_3$) δ 7.95 – 7.86 (m, 2H), 7.72 – 7.62 (m, 2H), 7.49 – 7.41 (m, 4H), 7.38 – 7.27 (m, 5H),

7.22 – 7.18 (m, 1H), 2.41 (d, *J* = 12.7 Hz, 1H), 2.28 (d, *J* = 12.3 Hz, 1H), 1.86 (d, *J* = 14.3 Hz, 1H), 1.62 (d, *J* = 14.3 Hz, 1H), 1.38 (s, 3H), 1.13 (s, 3H), 0.23 (s, 3H), 0.03 (s, 3H); **^{13}C NMR** (100 MHz, $CDCl_3$) δ 174.9, 150.1, 140.6, 138.2, 133.5, 131.1 (q, $^{2}J_{C-F}$ = 32.2 Hz), 128.6, 128.5, 128.0, 127.6, 126.1, 125.7, 125.0 (q, $^{3}J_{C-F}$ = 3.8 Hz), 124.1 (q, $^{1}J_{C-F}$ = 269 Hz), 76.5, 58.4, 50.8, 35.4, 27.9, 27.3, -1.4, -1.7; **^{19}F NMR** (376 MHz, $CDCl_3$) δ -62.7. HRMS (ESI) m/z calcd. for $C_{28}H_{30}F_3NNaSi$ $[M+Na]^+$ = 488.1992, found = 488.1990.

HPLC conditions: Daicel Chiralpak ADH-ADH column; hexane/2-propanol = 99.5/0.5, 0.5 mL/min, 25 °C. Retention times: 15.79 min (major), 17.16 min (minor).

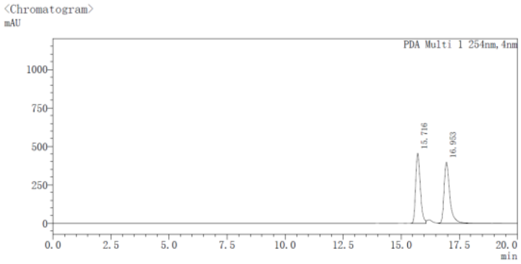

<Peak Table>
PDA Ch1 254nm

No.	Ret.Time(min)	Height(mAU)	Height%	Area(mAU*min)	Area%
1	15.716	455360	53.419	6351760	48.256
2	16.953	397069	46.581	6810997	51.744
Total		852429	100.000	13162757	100.000

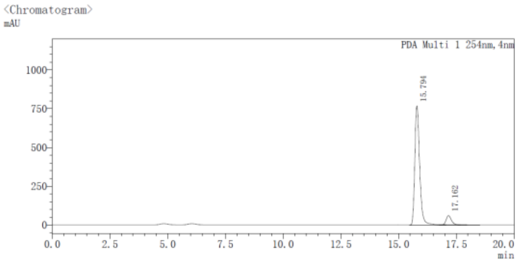

<Peak Table>
PDA Ch1 254nm

No.	Ret.Time(min)	Height(mAU)	Height%	Area(mAU*min)	Area%
1	15.794	768564	92.890	11275852	92.410
2	17.162	58827	7.110	926194	7.590
Total		827391	100.000	12202045	100.000

(*S*)-2-((Dimethyl(phenyl)silyl)methyl)-4,4-dimethyl-2-phenyl-5-(m-tolyl)-3,4-dihydro-2*H*-pyrrole (3i)

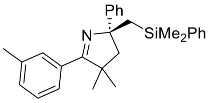

Following **General procedure A**, isolated **3i** 114.7 mg, 93% yield, 90% ee, as colorless oil, $[\alpha]_D^{20}$ = -220.7 (c = 2.9, $CHCl_3$). **^{1}H NMR** (400 MHz, $CDCl_3$) δ 7.72 – 7.60 (m, 2H), 7.52 – 7.48 (m, 4H), 7.41 – 7.26 (m, 7H), 7.23 – 7.19 (m, 1H), 2.46 (s, 3H), 2.41 (d, *J* = 12.9 Hz, 1H), 2.28 (d, *J* = 12.7 Hz, 1H), 1.86 (d, *J* = 14.5 Hz, 1H), 1.67 (d, *J* = 14.8 Hz, 1H), 1.41 (s, 3H), 1.16 (s, 3H), 0.28 (s, 3H), 0.07 (s, 3H); **^{13}C NMR** (100 MHz, $CDCl_3$) δ 176.1, 150.5, 140.8, 137.7, 134.8, 133.5, 130.1, 129.0, 128.4, 127.9, 127.5, 125.9, 125.8, 125.3, 76.0, 58.0, 50.8, 35.4, 28.1, 27.4, 21.6, -1.2, -1.7. HRMS (ESI) m/z calcd. for $C_{28}H_{34}NSi$ $[M+H]^+$ = 412.2455, found = 412.2462.

HPLC conditions: Daicel Chiralpak OZ-3 column; hexane/2-propanol = 100/0, 0.7 mL/min, 25 °C. Retention times: 10.71 min (major), 15.95 min (minor).

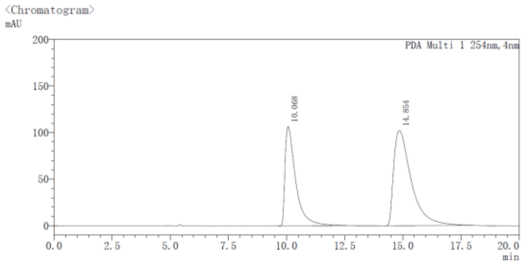

<Peak Table>
PDA Ch1 254nm

No.	Ret. Time(min)	Height(mAU)	Height%	Area(mAU*min)	Area%
1	10.068	106660	51.069	3389273	38.612
2	14.854	102193	48.931	5388447	61.388
Total		208853	100.000	8777720	100.000

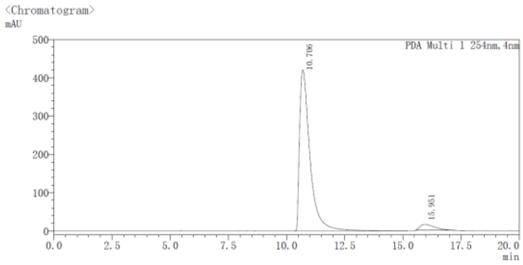

<Peak Table>
PDA Ch1 254nm

No.	Ret. Time(min)	Height(mAU)	Height%	Area(mAU*min)	Area%
1	10.706	420693	96.421	13115872	94.937
2	15.951	15616	3.579	699497	5.063
Total		436309	100.000	13815369	100.000

(*S*)-2-((Dimethyl(phenyl)silyl)methyl)-5-(3-methoxyphenyl)-4,4-dimethyl-2-phenyl-3,4-dihydro-2*H*-pyrrole (3j)

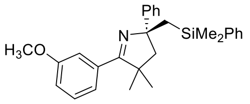

Following **General procedure A**, isolated **3j** 98.7 mg, 77% yield, 90% ee, as colorless oil, $[\alpha]_D^{20}$ = -199.4 (c = 2.5, $CHCl_3$). **^{1}H NMR** (400 MHz, $CDCl_3$) δ 7.57 – 7.40 (m, 6H), 7.38 – 7.27 (m, 6H), 7.20 (d, *J* = 7.8 Hz, 1H), 7.05 – 6.98 (m, 1H), 3.90 (s, 3H), 2.40 (d, *J* = 12.9 Hz, 1H), 2.27 (d, *J* = 11.6 Hz, 1H), 1.85 (d, *J* = 14.3 Hz, 1H), 1.63 (d, *J* = 15.6 Hz, 1H), 1.40 (s, 3H), 1.15 (s, 3H), 0.30 (s, 3H), 0.02 (s, 3H); **^{13}C NMR** (100 MHz, $CDCl_3$) δ 175.7, 159.3, 150.5, 140.8, 136.1, 133.5, 129.0, 128.4, 127.9, 127.5, 125.9, 125.8, 120.7, 115.2, 113.8, 76.0, 58.5, 55.3, 50.7, 35.4, 28.1, 27.4, -1.1, -1.8. HRMS (ESI) m/z calcd. for $C_{28}H_{34}NOSi$ $[M+H]^+$ = 428.2404, found = 428.2407.

HPLC conditions: Daicel Chiralpak OZ-3 column; hexane/2-propanol = 100/0, 0.7 mL/min, 25 ^oC. Retention times: 21.90 min (major), 31.01 min (minor).

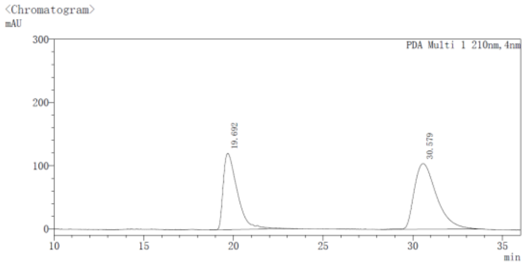

<Peak Table>
PDA Ch1 210nm

No.	Ret. Time(min)	Height(mAU)	Height%	Area(mAU*min)	Area%
1	19.692	120570	53.693	6618689	42.410
2	30.579	103985	46.307	8987750	57.590
Total		224555	100.000	15606438	100.000

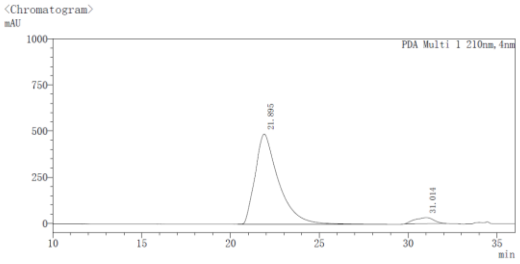

<Peak Table>
PDA Ch1 210nm

No.	Ret. Time(min)	Height(mAU)	Height%	Area(mAU*min)	Area%
1	21.895	488297	93.674	44636919	94.779
2	31.014	32975	6.326	2458758	5.221
Total		521272	100.000	47095677	100.000

(*S*)-2-((Dimethyl(phenyl)silyl)methyl)-5-(3-fluorophenyl)-4,4-dimethyl-2-phenyl-3,4-dihydro-2*H*-pyrrole (3k)

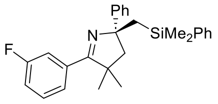

Following **General procedure A**, isolated **3k** 118.3 mg, 95% yield, 90% ee, as colorless oil, $[\alpha]_D^{20}$ = -176.8 (c = 2.9, $CHCl_3$). **^{1}H NMR** (400 MHz, $CDCl_3$) δ 7.63 – 7.53 (m, 2H), 7.51 – 7.35 (m, 5H), 7.29 (t, *J* = 6.2 Hz, 5H), 7.22 – 7.09 (m, 2H), 2.38 (d, *J* = 12.7 Hz, 1H), 2.25 (d, *J* = 12.8 Hz, 1H), 1.82 (d, *J* = 14.5 Hz, 1H), 1.60 (d, *J*

= 14.6 Hz, 1H), 1.37 (s, 3H), 1.11 (s, 3H), 0.23 (s, 3H), 0.01 (s, 3H); **^{13}C NMR** (100 MHz, $CDCl_3$) δ 174.7, 162.6 (d, $^{1}J_{C\text{-}F}$ = 245.2 Hz), 150.2, 136.9 (d, $^{3}J_{C\text{-}F}$ = 9.1 Hz), 133.5, 129.6 (d, $^{3}J_{C\text{-}F}$ = 8.0 Hz), 128.5, 128.0, 127.5, 126.0, 125.7, 123.9 (d, $^{4}J_{C\text{-}F}$ = 2.8 Hz), 116.3 (d, $^{2}J_{C\text{-}F}$ = 21.2 Hz), 115.3 (d, $^{2}J_{C\text{-}F}$ = 22.5 Hz), 76.2, 58.4, 50.7, 35.4, 28.0, 27.3, -1.3, -1.8; **^{19}F NMR** (376 MHz, $CDCl_3$) δ -112.9. HRMS (ESI) m/z calcd. for $C_{27}H_{31}FNSi$ $[M+H]^+$ = 416.2204, found = 416.2212.

HPLC conditions: Daicel Chiralpak OZ-3 column; hexane/2-propanol = 100/0, 0.7 mL/min, 25 °C. Retention times: 9.92 min (major), 12.20 min (minor).

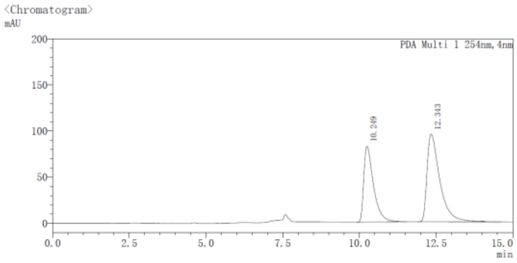

<Peak Table>
PDA Ch1 254nm

No.	Ret. Time(min)	Height(mAU)	Height%	Area(mAU*min)	Area%
1	10.249	82298	46.385	1817131	39.689
2	12.343	95125	53.615	2761312	60.311
Total		177422	100.000	4578443	100.000

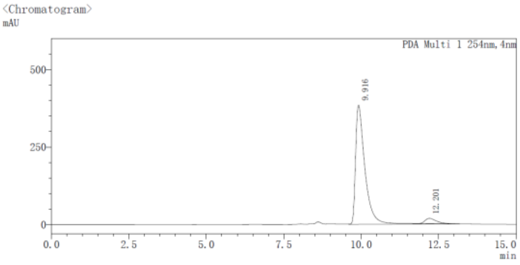

<Peak Table>
PDA Ch1 254nm

No.	Ret. Time(min)	Height(mAU)	Height%	Area(mAU*min)	Area%
1	9.916	384276	95.747	8091122	94.853
2	12.201	17070	4.253	439013	5.147
Total		401346	100.000	8530135	100.000

(*S*)-2-((Dimethyl(phenyl)silyl)methyl)-4,4-dimethyl-2-phenyl-5-(3-(trifluoromethyl)phenyl)-3,4-dihydro-2*H*-pyrrole (3l)

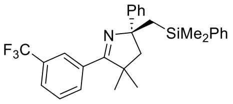

Following **General procedure A**, use **M8**, at -10 °C, isolated **3l** 121.4 mg, 87% yield, 90% ee, as colorless oil, $[\alpha]_D^{20}$ = -131.1 (c = 2.0, $CHCl_3$). **^{1}H NMR** (400 MHz, $CDCl_3$) δ 8.14 (s, 1H), 7.98 (d, *J* = 7.6 Hz, 1H), 7.69 (d, *J* = 7.6 Hz, 1H), 7.53 (t, *J* = 7.8 Hz, 1H), 7.48 – 7.41 (m, 4H), 7.36 – 7.26 (m, 5H), 7.24 – 7.15 (m, 1H), 2.41 (d, *J* = 12.7 Hz, 1H), 2.28 (d, *J* = 12.7 Hz, 1H), 1.84 (d, *J* = 14.6 Hz, 1H), 1.62 (d, *J* = 13.5 Hz, 1H), 1.38 (s, 3H), 1.13 (s, 3H), 0.23 (s, 3H), 0.01 (s, 3H); **^{13}C NMR** (100 MHz, $CDCl_3$) δ 174.6, 150.1, 140.5, 135.5, 133.5, 131.5, 130.6 (d, $^{2}J_{C\text{-}F}$ = 32 Hz), 130.7, 130.4, 128.6, 128.5, 128.0, 127.5, 126.0, 126.0 (q, $^{3}J_{C\text{-}F}$ = 3.9 Hz), 125.7, 125.1 (q, $^{3}J_{C\text{-}F}$ = 3.9 Hz), 124.0 (q, $^{1}J_{C\text{-}F}$ = 270 Hz), 76.4, 58.4, 50.7, 35.5, 27.9, 27.3, -1.3, -1.8; **^{19}F NMR** (376 MHz, $CDCl_3$) δ -62.6. HRMS (ESI) m/z calcd. for $C_{28}H_{31}F_3NSi$ $[M+H]^+$ = 466.2172, found = 466.2175.

HPLC conditions: Daicel Chiralpak OZ-3-OZ-3 column; hexane/2-propanol = 100/0, 0.7 mL/min, 25 °C. Retention times: 14.22 min (major), 15.12 min (minor).

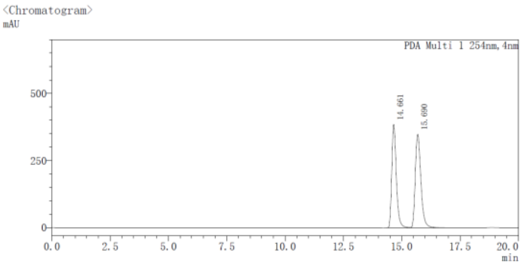

<Peak Table>
PDA Ch1 254nm

No.	Ret.Time(min)	Height(mAU)	Height%	Area(mAU*min)	Area%
1	14.661	385161	52.509	5246041	47.361
2	15.690	348349	47.491	5830774	52.639
Total		733510	100.000	11076815	100.000

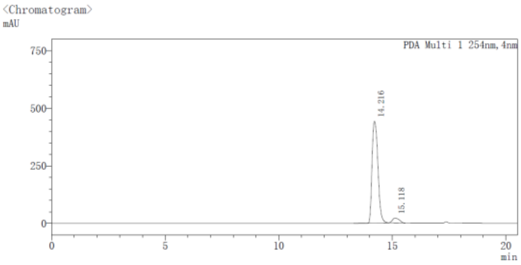

<Peak Table>
PDA Ch1 254nm

No.	Ret.Time(min)	Height(mAU)	Height%	Area(mAU*min)	Area%
1	14.216	441515	95.536	7886886	94.865
2	15.118	20629	4.464	426900	5.135
Total		462144	100.000	8313786	100.000

(*S*)-5-(2,3-Dihydrobenzo[*b*][1,4]dioxin-6-yl)-2-((dimethyl(phenyl)silyl)methyl)-4,4-dimethyl-2-phenyl-3,4-dihydro-2*H*-pyrrole (3m)

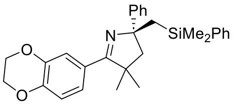

Following **General procedure A**, isolated **3m** 117.4 mg, 86% yield, 92% ee, as colorless oil, $[\alpha]_D^{20}$ = -232.3 (c = 2.9, $CHCl_3$). **^{1}H NMR** (400 MHz, $CDCl_3$) δ 7.69 – 7.54 (m, 6H), 7.50 – 7.38 (m, 5H), 7.36 – 7.29 (m, 1H), 7.06 (d, *J* = 8.5 Hz, 1H), 4.46 (s, 4H), 2.50 (dd, *J* = 13.0, 1.3 Hz, 1H), 2.39 (d, *J* = 12.8 Hz, 1H), 1.97 (dd, *J* = 14.5, 1.4 Hz, 1H), 1.73 (d, *J* = 14.6 Hz, 1H), 1.54 (s, 3H), 1.27 (s, 3H), 0.42 (s, 3H), 0.16 (s, 3H); **^{13}C NMR** (100 MHz, $CDCl_3$) δ 174.6, 150.7, 144.8, 143.0, 140.9, 133.5, 128.3, 128.1, 127.8, 127.5, 125.8, 125.7, 122.0, 117.5, 116.8, 75.6, 64.5, 64.3, 58.8, 50.4, 35.4, 28.2, 27.5, -1.1, -1.8. HRMS (ESI) m/z calcd. for $C_{29}H_{34}NO_2Si$ $[M+H]^+$ = 456.2353, found = 456.2363.

HPLC conditions: Daicel Chiralpak ODH column; hexane/2-propanol = 99.5/0.5, 0.5 mL/min, 25 ^{o}C. Retention times: 14.20 min (major), 13.67 min (minor).

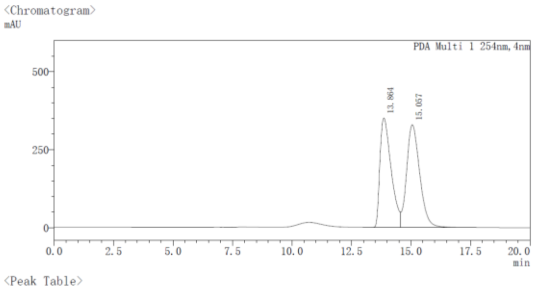

<Peak Table>
PDA Ch1 254nm

No.	Ret.Time(min)	Height(mAU)	Height%	Area(mAU*min)	Area%
1	13.864	349890	51.547	11484624	47.842
2	15.057	328887	48.453	12520570	52.158
Total		678777	100.000	24005194	100.000

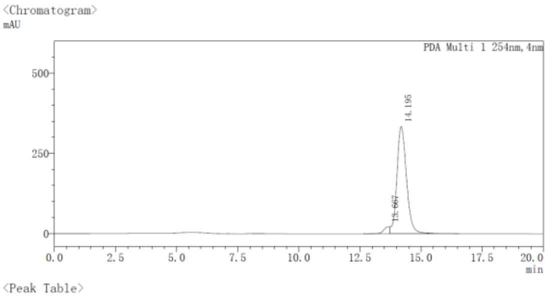

<Peak Table>
PDA Ch1 254nm

No.	Ret.Time(min)	Height(mAU)	Height%	Area(mAU*min)	Area%
1	13.667	21772	6.113	390105	4.090
2	14.195	334356	93.887	9147501	95.910
Total		356128	100.000	9537606	100.000

(*S*)-5-(3,4-Dimethoxyphenyl)-2-((dimethyl(phenyl)silyl)methyl)-4,4-dimethyl-2-phenyl-3,4-dihydro-2*H*-pyrrole (3n)

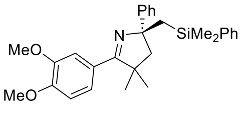

Following **General procedure A**, isolated **3n** 96.0 mg, 70% yield, 90% ee, as colorless oil, $[\alpha]_D^{20}$ = -222.0 (c = 2.4, $CHCl_3$). **^{1}H NMR** (400 MHz, $CDCl_3$) δ 7.75 (s, 1H), 7.51 – 7.40 (m, 5H), 7.35 – 7.25 (m, 5H), 7.23 – 7.15 (m, 1H), 6.89 (d,

J = 8.3 Hz, 1H), 3.96 (s, 6H), 2.39 (d, *J* = 12.8 Hz, 1H), 2.27 (d, *J* = 12.8 Hz, 1H), 1.86 (d, *J* = 14.6 Hz, 1H), 1.60 (d, *J* = 14.7 Hz, 1H), 1.42 (s, 3H), 1.17 (s, 3H), 0.32 (s, 3H), 0.01 (s, 3H); **^{13}C NMR** (100 MHz, $CDCl_3$) δ 174.7, 150.7, 150.3, 148.7, 140.9, 133.5, 128.4, 127.9, 127.5, 127.3, 125.9, 125.7, 121.0, 111.9, 110.0, 75.5, 59.2, 55.9, 55.8, 50.3, 35.3, 28.3, 27.6, -0.9, -1.8. HRMS (ESI) m/z calcd. for $C_{29}H_{36}NO_2Si$ $[M+H]^+$ = 458.2510, found = 458.2509.

HPLC conditions: Daicel Chiralpak ADH column; hexane/2-propanol = 99/1, 1.0 mL/min, 25 °C. Retention times: 7.53 min (major), 5.84 min (minor).

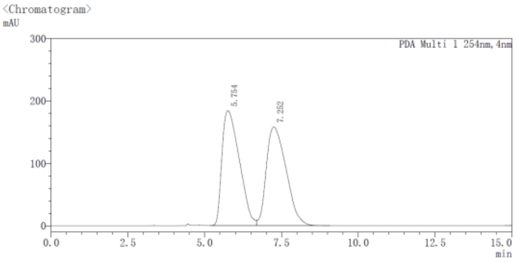

<Peak Table>
PDA Ch1 254nm

No.	Ret.Time(min)	Height(mAU)	Height%	Area(mAU*min)	Area%
1	5.754	184216	53.815	7257368	50.494
2	7.252	158100	46.185	7115379	49.506
Total		342317	100.000	14372747	100.000

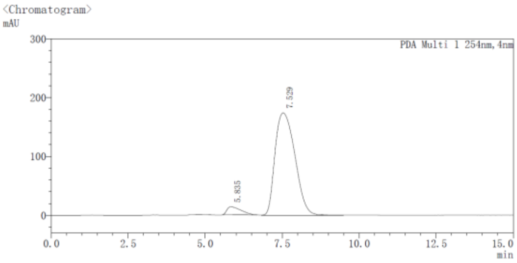

<Peak Table>
PDA Ch1 254nm

No.	Ret.Time(min)	Height(mAU)	Height%	Area(mAU*min)	Area%
1	5.835	13524	7.222	425150	5.179
2	7.529	173731	92.778	7783767	94.821
Total		187255	100.000	8208917	100.000

(*S*)-5-((9*S*,10*S*)-9,10-Dihydro-9,10-[1,2]benzenoanthracen-2-yl)-2-((dimethyl(phenyl)silyl)methyl)-4,4-dimethyl-2-phenyl-3,4-dihydro-2*H*-pyrrole (3o)

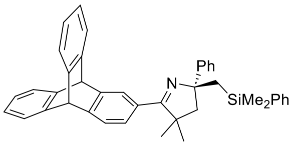

Following **General procedure A**, isolated **3o** 147.9 mg, 86% yield, 90% ee, as colorless oil, $[\alpha]_D^{20}$= -49.7 (c = 0.5, $CHCl_3$). **^{1}H NMR** (400 MHz, $CDCl_3$) δ 7.93 (s, 1H), 7.55 – 7.40 (m, 10H), 7.39 – 7.31 (m, 3H), 7.28 (d, *J* = 2.7 Hz, 2H), 7.22 – 7.16 (m, 1H), 7.10 – 7.04 (m, 4H), 5.51 (d, *J* = 6.2 Hz, 2H), 2.37 (d, *J* = 12.8 Hz, 1H), 2.23 (d, *J* = 12.8 Hz, 1H), 1.83 (d, *J* = 14.6 Hz, 1H), 1.63 (d, *J* = 10.0 Hz, 1H), 1.35 (s, 3H), 1.10 (s, 3H), 0.24 (s, 3H), 0.05 (s, 3H); **^{13}C NMR** (100 MHz, $CDCl_3$) δ 175.5, 150.5, 146.4, 145.1, 144.9, 140.9, 133.6, 131.7, 128.4, 127.8, 127.5, 125.8, 125.8, 125.2, 123.8, 123.8, 123.7, 123.6, 123.0, 75.8, 58.4, 54.1, 53.9, 50.6, 35.4, 28.1, 27.4, -1.3, -1.6. HRMS (ESI) m/z calcd. for $C_{41}H_{40}NSi$ $[M+H]^+$ = 574.2925, found = 574.2915.

HPLC conditions: Daicel Chiralpak ADH column; hexane/2-propanol = 97/3, 0.5 mL/min, 25 °C. Retention times: 8.14 min (major), 8.86 min (minor).

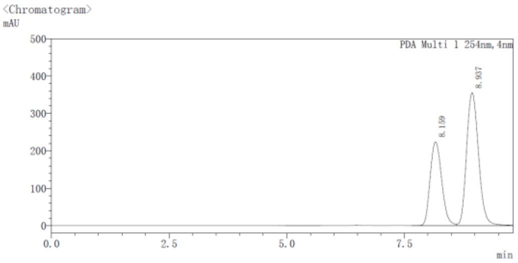

<Peak Table>
PDA Ch1 254nm

No.	Ret.Time(min)	Height(mAU)	Height%	Area(mAU*min)	Area%
1	8.159	224049	38.654	3814089	37.807
2	8.937	355574	61.346	6274261	62.193
Total		579624	100.000	10088350	100.000

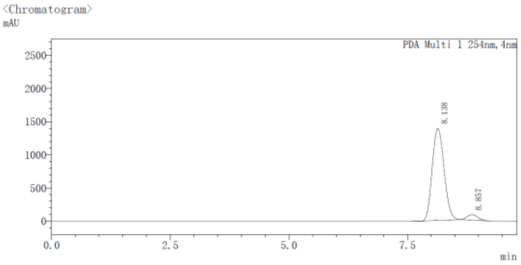

<Peak Table>
PDA Ch1 254nm

No.	Ret.Time(min)	Height(mAU)	Height%	Area(mAU*min)	Area%
1	8.138	1387088	94.448	24123123	94.844
2	8.857	81538	5.552	1311329	5.156
Total		1468625	100.000	25434452	100.000

(*S*)-2-((Dimethyl(phenyl)silyl)methyl)-4,4-dimethyl-5-(naphthalen-2-yl)-2-phenyl-3,4-dihydro-2*H*-pyrrole (3p)

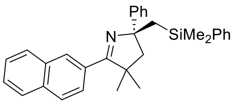

Following **General procedure A**, isolated **3p** 132.8 mg, 99% yield, 90% ee, as colorless oil, $[\alpha]_D^{20}$ = -214.7 (c = 3.3, $CHCl_3$). **^{1}H NMR** (400 MHz, $CDCl_3$) δ 8.23 (s, 1H), 8.08 (d, *J* = 8.6 Hz, 1H), 7.88 (d, *J* = 8.2 Hz, 3H), 7.58 – 7.43 (m, 6H), 7.36 – 7.26 (m, 5H), 7.19 (m, 1H), 2.42 (d, *J* = 12.8 Hz, 1H), 2.30 (d, *J* = 12.7 Hz, 1H), 1.87 (d, *J* = 13.5 Hz, 1H), 1.65 (d, *J* = 15.3 Hz, 1H), 1.47 (s, 3H), 1.21 (s, 3H), 0.27 (s, 3H), 0.04 (s, 3H); **^{13}C NMR** (100 MHz, $CDCl_3$) δ 175.7, 150.6, 140.8, 133.8, 133.6, 132.8, 132.2, 128.7, 128.4, 127.9, 127.9, 127.7, 127.6, 127.5, 127.5, 126.7, 126.3, 126.1, 125.9, 125.8, 76.1, 58.7, 50.8, 35.4, 28.3, 27.6, -1.1, -1.7. HRMS (ESI) m/z calcd. for $C_{31}H_{34}NSi$ $[M+H]^+$ = 448.2455, found = 448.2463.

HPLC conditions: Daicel Chiralpak ODH-ODH-ODH column; hexane/2-propanol = 99/1, 0.5 mL/min, 25 ^{o}C. Retention times: 22.20 min (major), 21.64 min (minor).

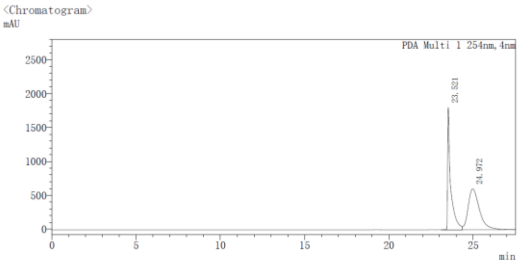

<Peak Table>
PDA Ch1 254nm

No.	Ret.Time(min)	Height(mAU)	Height%	Area(mAU*min)	Area%
1	23.521	1807841	74.950	22919202	44.890
2	24.972	604209	25.050	28137500	55.110
Total		2412050	100.000	51056703	100.000

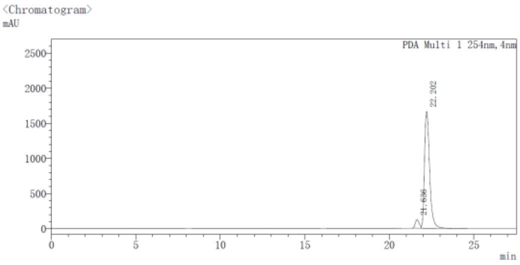

<Peak Table>
PDA Ch1 254nm

No.	Ret.Time(min)	Height(mAU)	Height%	Area(mAU*min)	Area%
1	21.636	120657	6.769	1906704	5.169
2	22.202	1661868	93.231	34983972	94.831
Total		1782524	100.000	36890676	100.000

(*S*)-2-((Dimethyl(phenyl)silyl)methyl)-2-(3-fluorophenyl)-4,4-dimethyl-5-phenyl-3,4-dihydro-2*H*-pyrrole (3q)

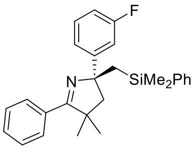

Following **General procedure A**, isolated **3q** 73.5 mg, 59% yield, 90% ee, as colorless oil, $[\alpha]_D^{20}$ = -120.9 (c = 1.1, $CHCl_3$). **^{1}H NMR** (400 MHz, $CDCl_3$) δ 7.87 – 7.84 (m, 2H), 7.52 – 7.39 (m, 5H), 7.33 – 7.30 (m, 3H), 7.25 –

7.09 (m, 3H), 6.89 – 6.83 (m, 1H), 2.39 – 2.21 (m, 2H), 1.82 (dd, J = 14.7, 2.2 Hz, 1H), 1.60 (dd, J = 14.6, 2.1 Hz, 1H), 1.39 (s, 3H), 1.15 (s, 3H), 0.29 (s, 3H), 0.08 (s, 3H); **^{13}C NMR** (100 MHz, $CDCl_3$) δ 176.3, 162.7 (d, $^1J_{C\text{-}F}$ = 244.5 Hz), 153.4 (d, $^3J_{C\text{-}F}$ = 6.7 Hz), 140.4, 134.5, 133.5, 129.5, 129.3 (d, $^3J_{C\text{-}F}$ = 8.1 Hz), 128.5, 128.3, 128.1, 127.5, 121.4 (d, $^4J_{C\text{-}F}$ = 2.7 Hz), 112.9 (d, $^2J_{C\text{-}F}$ = 22.1 Hz), 112.6 (d, $^2J_{C\text{-}F}$ = 21.4 Hz), 75.8, 58.5, 50.8, 35.3, 28.0, 27.4, -1.2, -1.7; **^{19}F NMR** (376 MHz, $CDCl_3$) δ -113.5. HRMS (ESI) m/z calcd. for $C_{27}H_{31}FNSi$ $[M+H]^+$ = 416.2204, found = 416.2201.

HPLC conditions: Daicel Chiralpak OZ-3 column; hexane/2-propanol = 100/0, 0.7 mL/min, 25 ^{o}C. Retention times: 10.05 min (major), 11.48 min (minor).

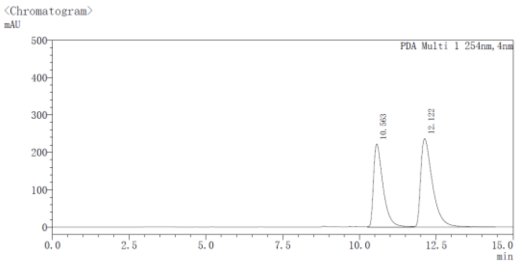

<Peak Table>
PDA Ch1 254nm

No.	Ret.Time(min)	Height(mAU)	Height%	Area(mAU*min)	Area%
1	10.563	222550	48.488	4779520	43.210
2	12.122	236431	51.512	6281618	56.790
Total		458981	100.000	11061138	100.000

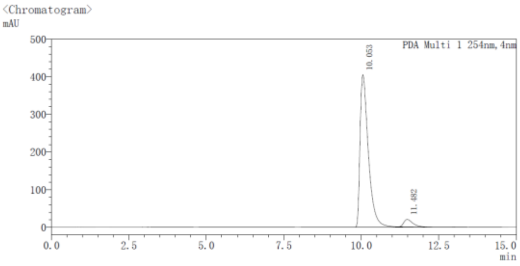

<Peak Table>
PDA Ch1 254nm

No.	Ret.Time(min)	Height(mAU)	Height%	Area(mAU*min)	Area%
1	10.053	405343	95.275	7654352	94.901
2	11.482	20103	4.725	411283	5.099
Total		425446	100.000	8065635	100.000

(*S*)-2-((Dimethyl(phenyl)silyl)methyl)-2-(3-methoxyphenyl)-4,4-dimethyl-5-phenyl-3,4-dihydro-2*H*-pyrrole (3r)

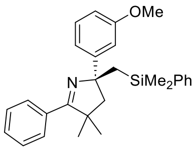

Following **General procedure A**, isolated **3r** 80.7 mg, 63% yield, 88% ee, as colorless oil, $[\alpha]_D^{20}$ = -66.3 (c = 1.3, $CHCl_3$). **^{1}H NMR** (400 MHz, $CDCl_3$) δ 7.90 – 7.78 (m, 2H), 7.51 – 7.38 (m, 5H), 7.33 – 7.26 (m, 3H), 7.20 (t, J = 8.0 Hz, 1H), 7.06 – 7.01 (m, 2H), 6.77 – 6.67 (m, 1H), 3.80 (s, 3H), 2.37 (d, J = 12.8 Hz, 1H), 2.24 (d, J = 12.8 Hz, 1H), 1.82 (d, J = 14.6 Hz, 1H), 1.60 (d, J = 14.6 Hz, 1H), 1.38 (s, 3H), 1.14 (s, 3H), 0.27 (s, 3H), 0.06 (s, 3H); **^{13}C NMR** (100 MHz, $CDCl_3$) δ 175.9, 159.2, 152.3, 140.8, 134.8, 133.5, 129.3, 128.8, 128.3, 128.3, 128.1, 127.5, 118.3, 112.1, 110.8, 76.0, 58.3, 55.1, 50.7, 35.3, 28.0, 27.4, -1.2, -1.7. HRMS (ESI) m/z calcd. for $C_{28}H_{34}NOSi$ $[M+H]^+$ = 428.2404, found = 428.2401.

HPLC conditions: Daicel Chiralpak OZ-3 column; hexane/2-propanol = 99.9/0.1, 0.7 mL/min, 25 ^{o}C. Retention times: 7.93 min (major), 8.64 min (minor).

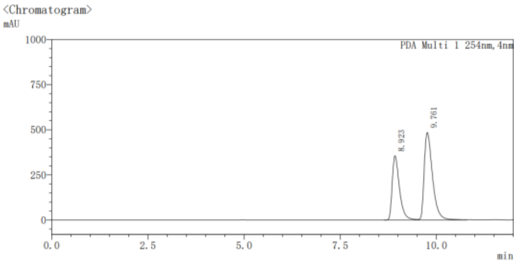

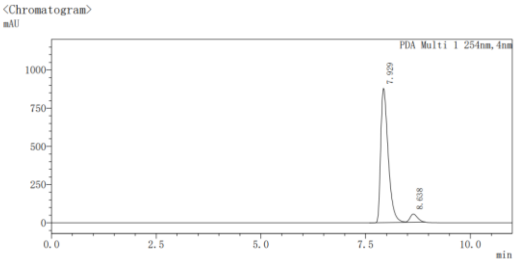

<Peak Table>
PDA Ch1 254nm

No.	Ret.Time(min)	Height(mAU)	Height%	Area(mAU*min)	Area%
1	8.923	355281	42.277	4683822	38.987
2	9.761	485087	57.723	7330016	61.013
Total		840368	100.000	12013838	100.000

<Peak Table>
PDA Ch1 254nm

No.	Ret.Time(min)	Height(mAU)	Height%	Area(mAU*min)	Area%
1	7.929	878887	94.224	10873168	94.183
2	8.638	53874	5.776	671522	5.817
Total		932761	100.000	11544690	100.000

(*S*)-2-(4-(*tert*-Butyl)phenyl)-2-((dimethyl(phenyl)silyl)methyl)-4,4-dimethyl-5-phenyl-3,4-dihydro-2*H*-pyrrole (3s)

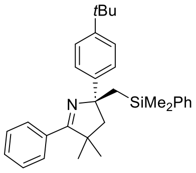

Following **General procedure A**, use **M8**, at -10 °C, isolated **3s** 112.0 mg, 88% yield, 88% ee, as colorless oil, $[\alpha]_D^{20}$ = -183.2 (c = 3.0, $CHCl_3$). **^{1}H NMR** (400 MHz, $CDCl_3$) δ 7.90 – 7.82 (m, 2H), 7.51 – 7.36 (m, 7H), 7.35 – 7.27 (m, 5H), 2.42 (d, *J* = 12.6 Hz, 1H), 2.25 (d, *J* = 12.5 Hz, 1H), 1.83 (d, *J* = 14.9 Hz, 1H), 1.66 (d, *J* = 14.7 Hz, 1H), 1.40 (s, 3H), 1.35 (s, 9H), 1.16 (s, 3H), 0.26 (s, 3H), 0.06 (s, 3H); **^{13}C NMR** (100 MHz, $CDCl_3$) δ 175.5, 148.6, 147.2, 140.8, 135.0, 133.5, 129.2, 128.3, 128.3, 128.0, 127.5, 125.3, 124.6, 75.9, 57.7, 50.8, 35.5, 34.3, 31.4, 28.1, 27.4, -1.2, -1.9. HRMS (ESI) m/z calcd. for $C_{31}H_{40}NSi$ $[M+H]^+$ = 454.2925, found = 454.2933.

HPLC conditions: Daicel Chiralpak ODH column; hexane/2-propanol = 100/0, 0.7 mL/min, 25 °C. Retention times: 9.04 min (major), 8.30 min (minor).

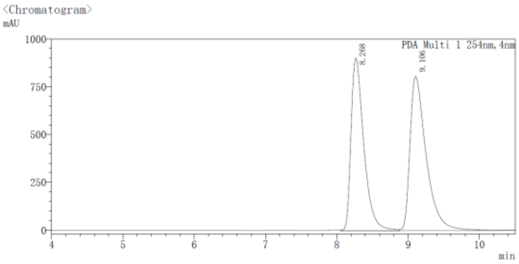

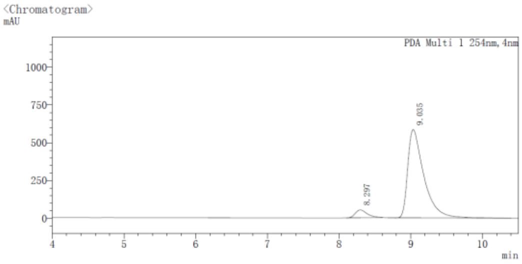

<Peak Table>
PDA Ch1 254nm

No.	Ret.Time(min)	Height(mAU)	Height%	Area(mAU*min)	Area%
1	8.268	902224	52.805	11195923	46.768
2	9.106	806373	47.195	12743499	53.232
Total		1708596	100.000	23939422	100.000

<Peak Table>
PDA Ch1 254nm

No.	Ret.Time(min)	Height(mAU)	Height%	Area(mAU*min)	Area%
1	8.297	52094	8.177	598114	6.134
2	9.035	584954	91.823	9153285	93.866
Total		637048	100.000	9751399	100.000

(*S*)-2-((Dimethyl(phenyl)silyl)methyl)-2-(4-methoxyphenyl)-4,4-dimethyl-5-phenyl-3,4-dihydro-2*H*-pyrrole (3t)

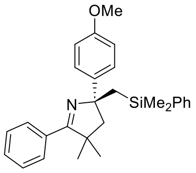

Following **General procedure A**, use **M8**, at -10 °C, isolated **3t** 102.5 mg, 80% yield, 87% ee, as colorless oil, $[\alpha]_D^{20}$ = -129.2 (c = 1.7, $CHCl_3$). **^{1}H NMR** (400 MHz, $CDCl_3$) δ 7.90 – 7.77 (m, 2H), 7.53 – 7.38 (m, 5H), 7.38 – 7.26 (m, 5H), 6.80 (d, *J* = 7.9 Hz, 2H), 3.80 (s, 3H), 2.35 (d, *J* = 12.8 Hz, 1H),

2.21 (d, J = 12.3 Hz, 1H), 1.79 (d, J = 14.6 Hz, 1H), 1.61 (d, J = 14.6 Hz, 1H), 1.37 (s, 3H), 1.13 (s, 3H), 0.25 (s, 3H), 0.05 (s, 3H); ^{13}**C NMR** (100 MHz, $CDCl_3$) δ 175.6, 157.7, 142.6, 140.8, 134.9, 133.5, 129.2, 128.3, 128.3, 128.1, 127.5, 126.8, 113.1, 75.7, 58.3, 55.2, 50.8, 35.6, 28.1, 27.3, -1.2, -1.7. HRMS (ESI) m/z calcd. for $C_{28}H_{34}NOSi$ $[M+H]^+$ = 428.2404, found = 428.2413.

HPLC conditions: Daicel Chiralpak IBN-5 column; hexane/2-propanol = 100/0, 0.7 mL/min, 25 ^{o}C. Retention times: 18.46 min (major), 16.00 min (minor).

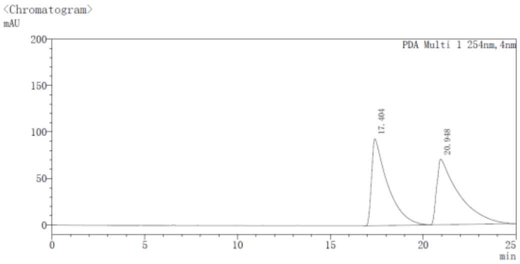

<Peak Table>
PDA Ch1 254nm

No.	Ret. Time(min)	Height(mAU)	Height%	Area(mAU*min)	Area%
1	17.404	93309	56.926	5602783	49.075
2	20.948	70604	43.074	5814001	50.925
Total		163913	100.000	11416785	100.000

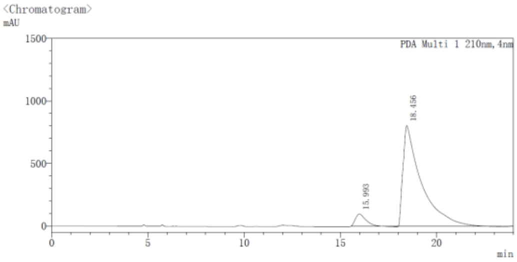

<Peak Table>
PDA Ch1 210nm

No.	Ret. Time(min)	Height(mAU)	Height%	Area(mAU*min)	Area%
1	15.993	97001	10.753	3599031	6.340
2	18.456	805067	89.247	53164962	93.660
Total		902068	100.000	56763992	100.000

(*S*)-5-(2,3-Dihydrobenzofuran-5-yl)-2-((dimethyl(phenyl)silyl)methyl)-4,4-dimethyl-2-phenyl-3,4-dihydro-2*H*-pyrrole (3u)

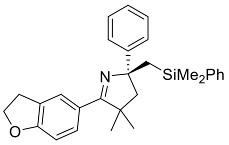

Following **General procedure A**, isolated **3u** 117.3 mg, 89% yield, 91% ee, as colorless oil, $[\alpha]_D^{20}$ = -228.6 (c = 2.9, $CHCl_3$). ^{1}**H NMR** (400 MHz, $CDCl_3$) δ 7.78 (s, 1H), 7.66 (d, J = 7.6 Hz, 1H), 7.54 – 7.37 (m, 5H), 7.34 – 7.27 (m, 4H), 7.21 – 7.15 (m, 1H), 6.81 (d, J = 8.4 Hz, 1H), 4.65 (t, J = 8.7 Hz, 2H), 3.28 (t, J = 8.7 Hz, 2H), 2.37 (d, J = 12.8 Hz, 1H), 2.25 (d, J = 12.8 Hz, 1H), 1.83 (d, J = 14.6 Hz, 1H), 1.62 (d, J = 14.6 Hz, 1H), 1.40 (s, 3H), 1.13 (s, 3H), 0.24 (s, 3H), 0.04 (s, 3H); ^{13}**C NMR** (100 MHz, $CDCl_3$) δ 175.2, 161.3, 150.8, 142.2, 141.0, 134.3, 133.6, 128.9, 128.7, 128.4, 127.9, 127.5, 125.9, 125.8, 125.6, 108.6, 75.5, 71.6, 58.8, 50.5, 35.4, 29.7, 28.3, 27.6, -1.2, -1.6. HRMS (ESI) m/z calcd. for $C_{29}H_{34}NOSi$ $[M+H]^+$ = 440.2404, found = 440.2414.

HPLC conditions: Daicel Chiralpak ADH column; hexane/2-propanol = 100/0, 0.7 mL/min, 25 ^{o}C. Retention times: 16.87 min (major), 14.50 min (minor).

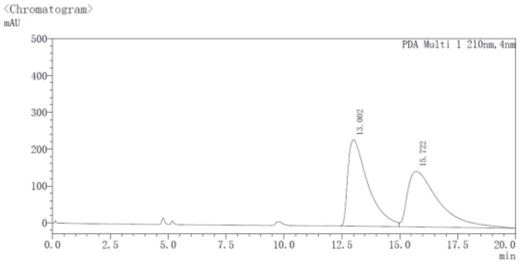

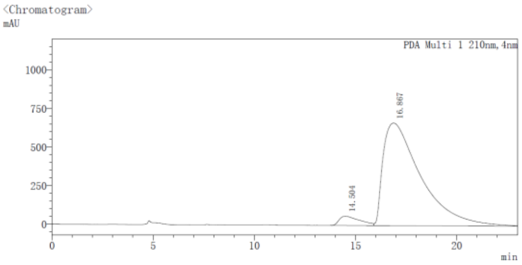

<Peak Table>
PDA Ch1 210nm

No.	Ret. Time (min)	Height (mAU)	Height%	Area (mAU*min)	Area%
1	13.002	234228	60.798	14100083	49.967
2	15.722	151030	39.202	14118701	50.033
Total		385258	100.000	28218784	100.000

<Peak Table>
PDA Ch1 210nm

No.	Ret. Time (min)	Height (mAU)	Height%	Area (mAU*min)	Area%
1	14.504	59753	8.239	4164690	4.588
2	16.867	665537	91.761	86616565	95.412
Total		725290	100.000	90781256	100.000

(*S*)-5-(2,3-Dihydrobenzofuran-5-yl)-2-((dimethyl(phenyl)silyl)methyl)-4,4-dimethyl-2-(p-tolyl)-3,4-dihydro-2*H*-pyrrole (3v)

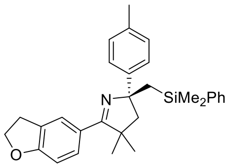

Following **General procedure A**, isolated **3v** 73.4 mg, 54% yield, 90% ee, as colorless oil, $[\alpha]_D^{20}$ = -88.7 (c = 1.8, $CHCl_3$). **^{1}H NMR** (400 MHz, $CDCl_3$) δ 7.78 (s, 1H), 7.66 (d, *J* = 8.4 Hz, 1H), 7.50 (dd, *J* = 5.5, 3.1 Hz, 2H), 7.34 (s, 5H), 7.10 (d, *J* = 7.7 Hz, 2H), 6.83 (dd, *J* = 8.4, 2.9 Hz, 1H), 4.67 (ddd, *J* = 11.5, 6.6, 2.5 Hz, 2H), 3.30 (t, *J* = 8.9 Hz, 2H), 2.36 (s, 3H), 2.24 (d, *J* = 12.8 Hz, 1H), 1.86 – 1.78 (m, 1H), 1.64 (d, *J* = 14.3 Hz, 1H), 1.41 (s, 3H), 1.35 (s, 1H), 1.16 (s, 3H), 0.26 (s, 3H), 0.08 (s, 3H); **^{13}C NMR** (100 MHz, $CDCl_3$) δ 175.0, 147.8, 141.0, 135.1, 133.6, 128.6, 128.5, 128.3, 127.5, 127.1, 125.7, 125.5, 108.6, 75.3, 71.6, 58.5, 50.5, 35.4, 29.6, 28.2, 27.5, 21.0, -1.2, -1.6. HRMS (ESI) m/z calcd. for $C_{30}H_{36}NOSi$ $[M+H]^+$ = 454.2561, found = 454.2559.

HPLC conditions: Daicel Chiralpak ADH-ADH column; hexane/2-propanol = 100/0, 0.6 mL/min, 25 ^{o}C. Retention times: 32.99 min (major), 29.58 min (minor).

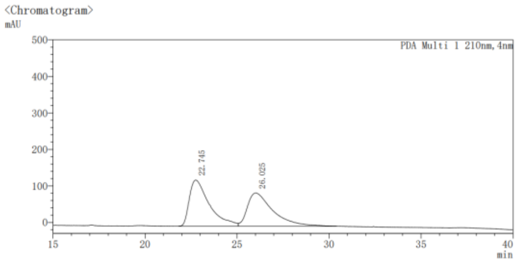

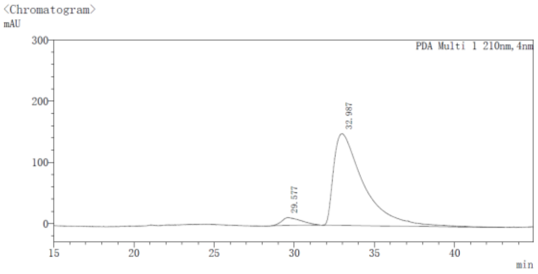

<Peak Table>
PDA Ch1 210nm

No.	Ret. Time (min)	Height (mAU)	Height%	Area (mAU*min)	Area%
1	22.745	126090	58.119	9531120	52.136
2	26.025	90863	41.881	8750299	47.864
Total		216953	100.000	18281419	100.000

<Peak Table>
PDA Ch1 210nm

No.	Ret. Time (min)	Height (mAU)	Height%	Area (mAU*min)	Area%
1	29.577	13022	7.988	1084589	5.232
2	32.987	150001	92.012	19646407	94.768
Total		163023	100.000	20730996	100.000

(*S*)-5-(2,3-Dihydrobenzofuran-5-yl)-2-((dimethyl(phenyl)silyl)methyl)-2-(4-fluorophenyl)-4,4-dimethyl-3,4-dihydro-2*H*-pyrrole (3w)

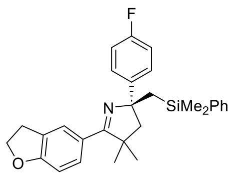

Following **General procedure A**, isolated **3w** 83.7 mg, 61% yield, 91% ee, as colorless oil, $[\alpha]_D^{20}$ = -113.7 (c = 2.1, $CHCl_3$). **^{1}H NMR** (400 MHz, $CDCl_3$) δ 7.75 (s, 1H), 7.63 (d, *J* = 7.9 Hz, 1H), 7.47 – 7.40 (m, 2H), 7.39 – 7.27 (m, 5H), 6.96 – 6.87 (m, 2H), 6.80 (d, *J* = 8.4 Hz, 1H), 4.63 (t, *J* = 8.7 Hz, 2H), 3.26 (t, *J* = 8.7 Hz, 2H), 2.35 – 2.17 (m, 2H), 1.77 (d, *J* = 14.6 Hz, 1H), 1.58 (d, *J* = 14.6 Hz, 1H), 1.39 (s, 3H), 1.12 (s, 3H), 0.23 (s, 3H), 0.05 (s, 3H); **^{13}C NMR** (100 MHz, $CDCl_3$) δ 175.2, 161.3, 161.1 (d, $^1J_{C-F}$ = 242 Hz), 159.9, 146.5, 140.6, 133.5, 128.6, 128.4, 127.5, 127.3 (d, $^3J_{C-F}$ = 7.8 Hz), 127.1 (d, $^4J_{C-F}$ = 5.0 Hz), 125.5, 114.4 (d, $^2J_{C-F}$ = 20.8 Hz), 108.6, 75.1, 71.6, 58.9, 50.5, 35.6, 29.6, 28.2, 27.5, -1.1, -1.6; **^{19}F NMR** (376 MHz, $CDCl_3$) δ -117.9. HRMS (ESI) m/z calcd. for $C_{29}H_{33}FNOSi$ $[M+H]^+$ = 458.2310, found = 458.2318.

HPLC conditions: Daicel Chiralpak IA column; hexane/2-propanol = 100/0, 0.7 mL/min, 25 ^{o}C. Retention times: 20.27 min (major), 18.03 min (minor).

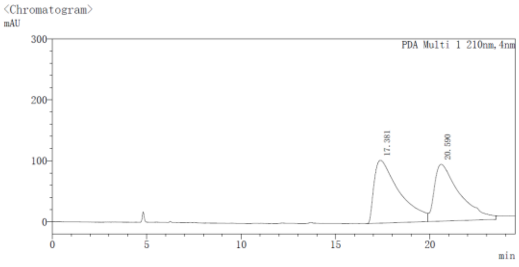

<Peak Table>
PDA Ch1 210nm

No.	Ret. Time(min)	Height(mAU)	Height%	Area(mAU*min)	Area%
1	17.381	103113	52.494	9579184	52.422
2	20.590	93315	47.506	8694111	47.578
Total		196428	100.000	18273295	100.000

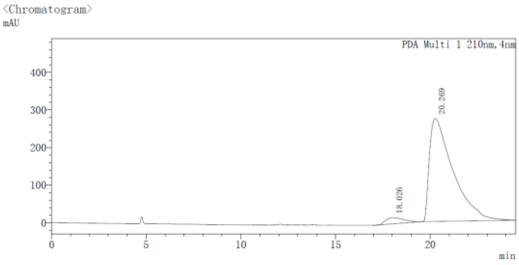

<Peak Table>
PDA Ch1 210nm

No.	Ret. Time(min)	Height(mAU)	Height%	Area(mAU*min)	Area%
1	18.026	16248	5.600	1066960	4.291
2	20.269	273893	94.400	23797972	95.709
Total		290141	100.000	24864932	100.000

(*S*)-2-((Dimethyl(phenyl)silyl)methyl)-4,4-dimethyl-2-phenyl-5-(thiophen-2-yl)-3,4-dihydro-2*H*-pyrrole (3x)

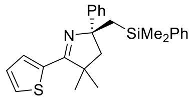

Following **General procedure A**, isolated **3x** 62.9 mg, 52% yield, 90% ee, as colorless oil, $[\alpha]_D^{20}$ = -141.6 (c = 1.6, $CHCl_3$). **^{1}H NMR** (400 MHz, $CDCl_3$) δ 7.53 – 7.40 (m, 6H), 7.36 – 7.26 (m, 5H), 7.22 – 7.16 (m, 1H), 7.13 (t, *J* = 4.4 Hz, 1H), 2.37 (d, *J* = 12.6 Hz, 1H), 2.28 (d, *J* = 12.3 Hz, 1H), 1.86 (d, *J* = 15.5 Hz, 1H), 1.58 (d, *J* = 14.8 Hz, 1H), 1.46 (s, 3H), 1.18 (s, 3H), 0.35 (s, 3H), 0.02 (s, 3H); **^{13}C NMR** (100 MHz, $CDCl_3$) δ 170.5, 150.7, 140.9, 139.1, 133.5, 128.5, 128.4, 127.9, 127.5, 127.5, 127.3, 125.8, 125.6, 76.6, 58.8, 50.5, 35.3, 28.1, 27.4, -1.0, -1.9. HRMS (ESI) m/z calcd. for $C_{25}H_{30}NSSi$ $[M+H]^+$ = 404.1863, found = 404.1862.

HPLC conditions: Daicel Chiralpak OZ-3 column; hexane/2-propanol = 100/0, 0.7 mL/min, 25 ^{o}C. Retention times: 13.17 min (major), 17.70 min (minor).

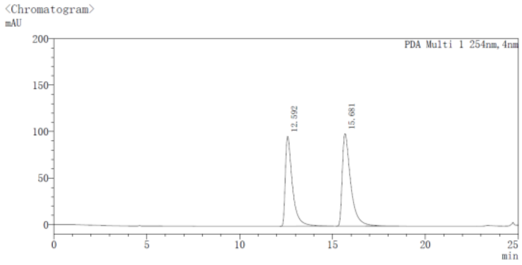

<Peak Table>
PDA Ch1 254nm

No.	Ret. Time(min)	Height(mAU)	Height%	Area(mAU*min)	Area%
1	12.592	96564	49.278	2566743	44.947
2	15.681	99394	50.722	3143813	55.053
Total		195958	100.000	5710556	100.000

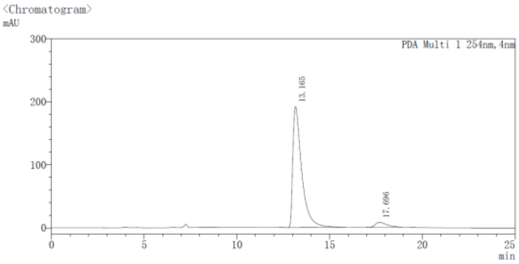

<Peak Table>
PDA Ch1 254nm

No.	Ret. Time(min)	Height(mAU)	Height%	Area(mAU*min)	Area%
1	13.165	191932	96.005	6421017	94.859
2	17.696	7988	3.995	347974	5.141
Total		199920	100.000	6768991	100.000

(*S*)-7-((Dimethyl(phenyl)silyl)methyl)-5,7-diphenyl-6-azaspiro[3.4]oct-5-ene (3y)

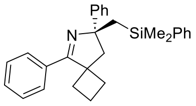

Following **General procedure A**, isolated **3y** 67.5 mg, 55% yield, 90% ee, as colorless oil, $[\alpha]_D^{20}$ = -58.6 (c = 1.7, $CHCl_3$). **^{1}H NMR** (400 MHz, $CDCl_3$) δ 7.99 (s, 2H), 7.57 – 7.53 (m, 7H), 7.44 – 7.34 (m, 5H), 7.32 – 7.26 (m, 1H), 2.85 (q, *J* = 9.7 Hz, 1H), 2.69 – 2.58 (m, 3H), 2.28 – 2.18 (m, 1H), 2.12 – 2.03 (m, 1H), 1.85 (d, *J* = 14.1 Hz, 2H), 1.61 (d, *J* = 14.5 Hz, 1H), 1.40 (d, *J* = 10.7 Hz, 1H), 0.28 (s, 3H), 0.10 (s, 3H); **^{13}C NMR** (100 MHz, $CDCl_3$) δ 174.5, 149.6, 140.8, 135.1, 133.5, 129.3, 128.4, 128.2, 128.0, 127.9, 127.5, 126.0, 125.8, 76.6, 57.7, 55.0, 33.3, 33.0, 32.3, 16.4, -1.2, -1.8. HRMS (ESI) m/z calcd. for $C_{28}H_{32}NSi$ $[M+H]^+$ = 410.2299, found = 410.2300.

HPLC conditions: Daicel Chiralpak OZ-3 column; hexane/2-propanol = 100/0, 0.7 mL/min, 25 ^{o}C. Retention times: 13.59 min (major), 21.52 min (minor).

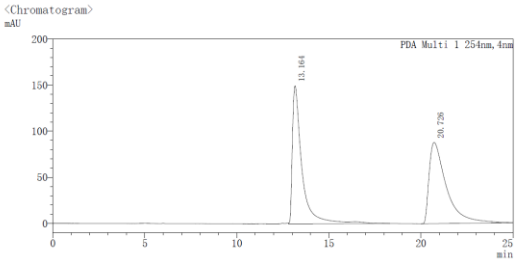

<Peak Table>
PDA Ch1 254nm

No.	Ret. Time(min)	Height(mAU)	Height%	Area(mAU*min)	Area%
1	13.164	149718	62.954	5430898	48.578
2	20.726	88101	37.046	5748743	51.422
Total		237819	100.000	11179641	100.000

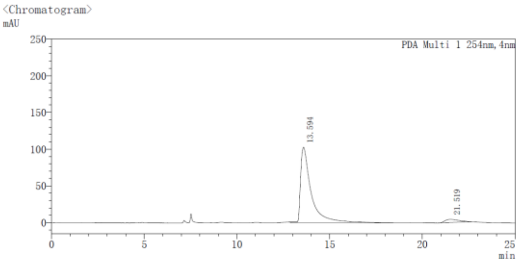

<Peak Table>
PDA Ch1 254nm

No.	Ret. Time(min)	Height(mAU)	Height%	Area(mAU*min)	Area%
1	13.594	102789	96.056	4085227	94.963
2	21.519	4220	3.944	216707	5.037
Total		107010	100.000	4301934	100.000

(*S*)-3-((Dimethyl(phenyl)silyl)methyl)-1,3-diphenyl-2-azaspiro[4.4]non-1-ene (3z)

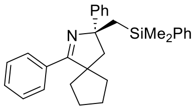

Following **General procedure A**, isolated **3z** 77.5 mg, 61% yield, 90% ee, as colorless oil, $[\alpha]_D^{20}$ = -99.3 (c = 1.9, $CHCl_3$). **^{1}H NMR** (400 MHz, $CDCl_3$) δ 7.87 – 7.77 (m, 2H), 7.54 – 7.39 (m, 7H), 7.37 – 7.27 (m, 5H), 7.20 (t, *J* = 7.3 Hz, 1H), 2.37 (d, *J* = 12.5 Hz, 1H), 2.24 (d, *J* = 12.7 Hz, 2H), 1.92 (dt, *J* = 15.1, 8.5 Hz, 1H), 1.85 (d, *J* = 14.5 Hz, 1H), 1.80 – 1.65 (m, 4H), 1.55 (dd, *J* = 13.8, 8.0 Hz, 1H), 1.39 – 1.23 (m, 2H), 0.27 (s, 3H), 0.04 (s, 3H); **^{13}C NMR** (100 MHz, $CDCl_3$) δ 175.5, 150.6, 140.9, 134.7, 133.6, 129.2, 128.4,

128.3, 128.1, 127.8, 127.5, 125.8, 76.8, 60.8, 58.9, 38.5, 38.0, 34.7, 25.9, 25.6, -1.1, -1.7. HRMS (ESI) m/z calcd. for $C_{29}H_{34}NSi$ $[M+H]^+$ = 424.2455, found = 424.2459.

HPLC conditions: Daicel Chiralpak OZ-3 column; hexane/2-propanol = 100/0, 0.7 mL/min, 25 °C. Retention times: 11.01 min (major), 17.53 min (minor).

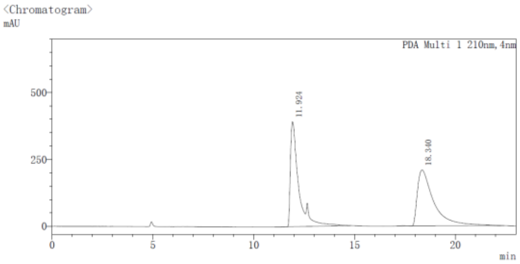

<Peak Table>
PDA Ch1 210nm

No.	Ret. Time (min)	Height (mAU)	Height%	Area (mAU*min)	Area%
1	11.924	392323	65.251	11224077	48.631
2	18.340	208931	34.749	11856138	51.369
Total		601254	100.000	23080214	100.000

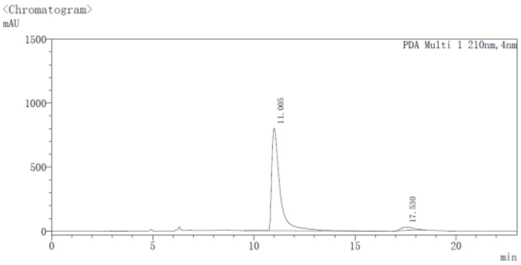

<Peak Table>
PDA Ch1 210nm

No.	Ret. Time (min)	Height (mAU)	Height%	Area (mAU*min)	Area%
1	11.005	800727	96.644	22460102	94.994
2	17.530	27810	3.356	1183639	5.006
Total		828537	100.000	23643741	100.000

(*S*)-4,4-Dimethyl-2,5-diphenyl-2-((triethylsilyl)methyl)-3,4-dihydro-2*H*-pyrrole (3aa)

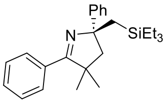

Following **General procedure A**, use **M8**, isolated **3aa** 69.0 mg, 61% yield, 90% ee, as colorless oil, $[\alpha]_D^{20}$ = -126.8 (c = 1.5, $CHCl_3$). **^{1}H NMR** (400 MHz, $CDCl_3$) δ 7.93 – 7.89 (m, 2H), 7.54 – 7.37 (m, 5H), 7.32 – 7.26 (m, 2H), 7.22 – 7.15 (m, 1H), 2.44 (d, *J* = 12.7 Hz, 1H), 2.29 (d, *J* = 12.8 Hz, 1H), 1.56 (d, *J* = 14.6 Hz, 1H), 1.47 (s, 3H), 1.37 (d, *J* = 14.6 Hz, 1H), 1.14 (s, 3H), 0.87 – 0.75 (m, 9H), 0.51 – 0.29 (m, 6H); **^{13}C NMR** (100 MHz, $CDCl_3$) δ 175.5, 150.9, 135.0, 129.2, 128.3, 128.1, 127.8, 125.8, 125.8, 76.1, 58.8, 50.7, 31.0, 28.3, 27.4, 7.4, 4.3. HRMS (ESI) m/z calcd. for $C_{25}H_{36}NSi$ $[M+H]^+$ = 378.2612, found = 378.2604.

HPLC conditions: Daicel Chiralpak OZ-3 column; hexane/2-propanol = 100/0, 0.7 mL/min, 25 °C. Retention times: 5.68 min (major), 6.40 min (minor).

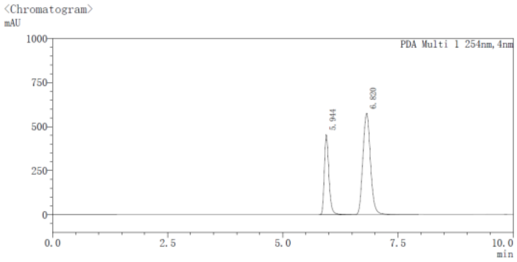

<Peak Table>
PDA Ch1 254nm

No.	Ret. Time (min)	Height (mAU)	Height%	Area (mAU*min)	Area%
1	5.944	453812	44.103	3109832	32.028
2	6.820	575168	55.897	6599877	67.972
Total		1028980	100.000	9709709	100.000

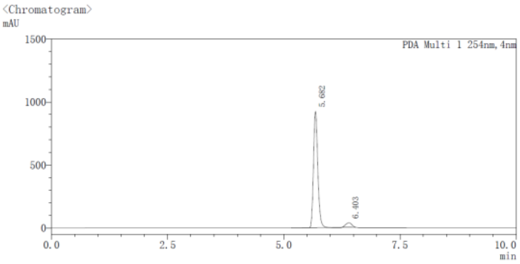

<Peak Table>
PDA Ch1 254nm

No.	Ret. Time (min)	Height (mAU)	Height%	Area (mAU*min)	Area%
1	5.682	921265	96.275	5906508	94.790
2	6.403	35640	3.725	324642	5.210
Total		956905	100.000	6231150	100.000

(*S*)-5-(4-Fluorophenyl)-4,4-dimethyl-2-phenyl-2-((triethylsilyl)methyl)-3,4-dihydro-2*H*-pyrrole (3ab)

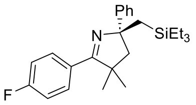

Following **General procedure A**, use **M8**, isolated **3ab** 74.7 mg, 63% yield, 91% ee, as colorless oil, $[\alpha]_D^{20}$ = -80.7 (c = 1.2, $CHCl_3$). **^{1}H NMR** (400 MHz, $CDCl_3$) δ 8.09 – 7.97 (m, 2H), 7.61 – 7.50 (m, 2H), 7.43 – 7.34 (m, 2H), 7.32 – 7.26 (m, 1H), 7.20 (t, *J* = 8.7 Hz, 2H), 2.53 (d, *J* = 12.8 Hz, 1H), 2.38 (d, *J* = 12.8 Hz, 1H), 1.65 (d, *J* = 14.5 Hz, 1H), 1.57 (s, 3H), 1.46 (s, 1H), 1.23 (s, 3H), 0.89 (t, *J* = 7.9 Hz, 9H), 0.55 – 0.39 (m, 6H); **^{13}C NMR** (100 MHz, $CDCl_3$) δ 174.2, 163.5 (d, $^1J_{C-F}$ = 249.2 Hz), 162.3, 150.8, 131.0 (d, $^4J_{C-F}$ = 3.4 Hz), 130.3 (d, $^3J_{C-F}$ = 8.1 Hz), 127.8, 125.8, 125.7, 115.1 (d, $^2J_{C-F}$ = 21.6 Hz), 76.0, 59.0, 50.6, 30.9, 28.3, 27.4, 7.4, 4.3; **^{19}F NMR** (376 MHz, $CDCl_3$) δ -111.9. HRMS (ESI) m/z calcd. for $C_{25}H_{35}FNSi$ $[M+H]^+$ = 396.2517, found = 396.2506.

HPLC conditions: Daicel Chiralpak ODH-ODH-ODH column; hexane/2-propanol = 100/0, 0.7 mL/min, 25 °C. Retention times: 20.68 min (major), 20.21 min (minor).

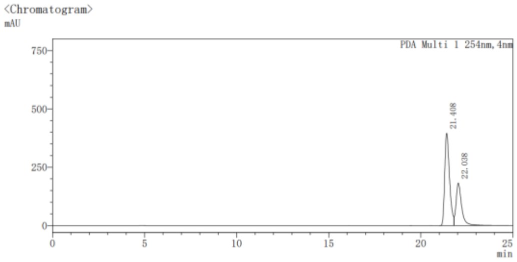

<Peak Table>
PDA Ch1 254nm

No.	Ret.Time(min)	Height(mAU)	Height%	Area(mAU*min)	Area%
1	21.408	395446	68.452	7150019	65.009
2	22.038	182252	31.548	3848406	34.991
Total		577698	100.000	10998426	100.000

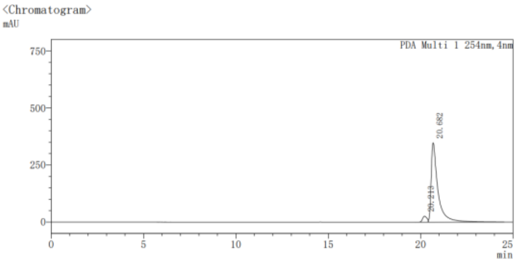

<Peak Table>
PDA Ch1 254nm

No.	Ret.Time(min)	Height(mAU)	Height%	Area(mAU*min)	Area%
1	20.213	25997	6.962	430251	4.633
2	20.682	347415	93.038	8856393	95.367
Total		373413	100.000	9286644	100.000

(*S*)-4,4-Dimethyl-2,5-diphenyl-2-((tributylsilyl)methyl)-3,4-dihydro-2*H*-pyrrole (3ac)

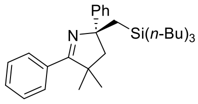

Following **General procedure A**, at 15 °C, isolated **3ac** 101.0 mg, 73% yield, 87% ee, as colorless oil, $[\alpha]_D^{20}$ = -81.1 (c = 1.5, $CHCl_3$). **^{1}H NMR** (400 MHz, $CDCl_3$) δ 7.93 – 7.90 (m, 2H), 7.48 (d, *J* = 7.3 Hz, 2H), 7.46 – 7.37 (m, 3H), 7.30 – 7.26 (m, 2H), 7.18 (t, *J* = 7.1 Hz, 1H), 2.42 (d, *J* = 12.6 Hz, 1H), 2.29 (d, *J* = 12.3 Hz, 1H), 1.55 (d, *J* = 14.7 Hz, 1H), 1.49 (s, 3H), 1.35 (d, *J* = 14.6 Hz, 1H), 1.24 – 1.02 (m, 15H), 0.83 – 0.78 (m, 9H), 0.47 – 0.27 (m, 6H); **^{13}C NMR** (100 MHz, $CDCl_3$) δ 175.3, 150.9, 135.0, 129.2, 128.3, 128.0, 127.8, 125.8, 125.8, 76.2, 59.0, 50.7, 31.9, 28.4, 27.5, 26.9, 26.1, 13.7, 13.3. HRMS (ESI) m/z calcd. for $C_{31}H_{47}NSi$ $[M+H]^+$ = 461.3478, found = 462.3550.

HPLC conditions: Daicel Chiralpak OZ-3 column; hexane/2-propanol = 100/0, 0.7 mL/min. Retention times: 9.98 min (major), 10.73 min (minor).

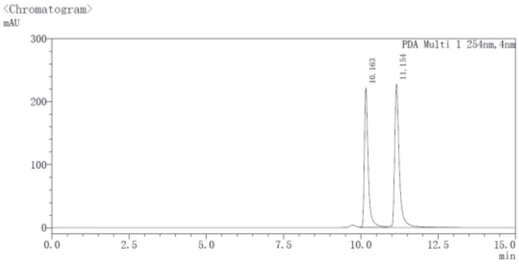

<Peak Table>
PDA Ch1 254nm

No.	Ret.Time(min)	Height(mAU)	Height%	Area(mAU*min)	Area%
1	10.163	220914	49.280	1908340	44.435
2	11.154	227365	50.720	2386352	55.565
Total		448279	100.000	4294692	100.000

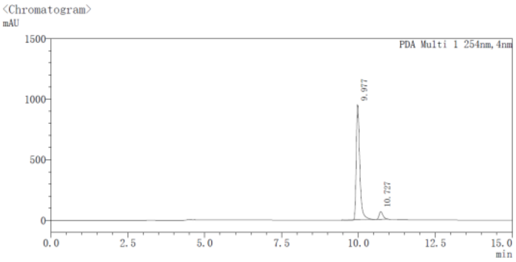

<Peak Table>
PDA Ch1 254nm

No.	Ret.Time(min)	Height(mAU)	Height%	Area(mAU*min)	Area%
1	9.977	949571	93.679	7604830	93.271
2	10.727	64073	6.321	548671	6.729
Total		1013645	100.000	8153502	100.000

(*R*)-2-((*R*)-(Dimethyl(phenyl)silyl)(phenyl)methyl)-2,4,4-trimethyl-5-phenyl-3,4-dihydro-2*H*-pyrrole (3ad)

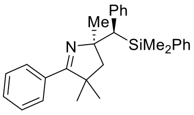

Following **General procedure B**, isolated **3ad** 85.1 mg, 69% yield, 93% ee, as colorless oil, $[\alpha]_D^{20}$ = +81.0 (c = 1.0, $CHCl_3$). **^{1}H NMR** (400 MHz, $CDCl_3$) δ 7.66 – 7.54 (m, 4H), 7.45 – 7.26 (m, 10H), 7.23 – 7.16 (m, 1H), 2.77 (d, *J* = 1.4 Hz, 1H), 2.20 (d, *J* = 13.2 Hz, 1H), 1.66 (d, *J* = 13.2 Hz, 1H), 1.41 (s, 3H), 1.35 (s, 3H), 1.13 (s, 3H), 0.50 (s, 3H), 0.27 (s, 3H); **^{13}C NMR** (100 MHz, $CDCl_3$) δ 141.7, 140.1, 134.3, 131.3, 129.0, 128.5, 128.1, 128.0, 127.6, 127.5, 125.2, 75.4, 53.5, 50.6, 30.1, 29.4, 28.0, -0.2, -1.4. HRMS (ESI) m/z calcd. for $C_{28}H_{34}NSi$ $[M+H]^+$ = 412.2455, found = 412.2463.

HPLC conditions: Daicel Chiralpak IC column; hexane/2-propanol = 99/1, 0.5 mL/min, 25 °C. Retention times: 8.68 min (major), 8.02 min (minor).

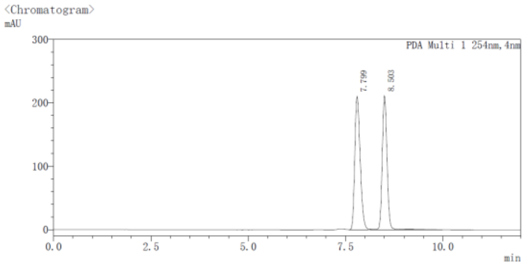

<Peak Table>
PDA Ch1 254nm

No.	Ret.Time(min)	Height(mAU)	Height%	Area(mAU*min)	Area%
1	7.799	210368	49.895	2021774	52.649
2	8.503	211250	50.105	1818337	47.351
Total		421619	100.000	3840111	100.000

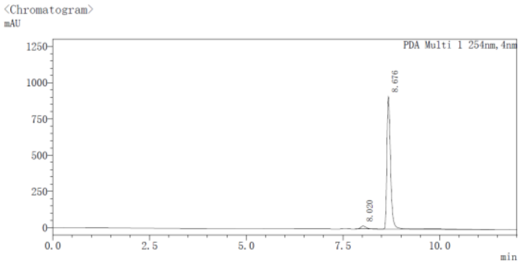

<Peak Table>
PDA Ch1 254nm

No.	Ret.Time(min)	Height(mAU)	Height%	Area(mAU*min)	Area%
1	8.020	20992	2.247	216887	3.403
2	8.676	913016	97.753	6157129	96.597
Total		934008	100.000	6374016	100.000

(*S*)-2-((*R*)-(Dimethyl(phenyl)silyl)(phenyl)methyl)-4,4-dimethyl-2,5-diphenyl-3,4-dihydro-2*H*-pyrrole (3ae)

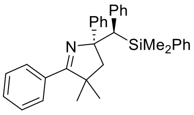

Following **General procedure B**, isolated **3ae** 103.6 mg, 73% yield, 92% ee, as colorless oil, $[\alpha]_D^{20}$ = -185.1 (c = 1.0, $CHCl_3$). **^{1}H NMR** (400 MHz, $CDCl_3$) δ 8.11 – 8.04 (m, 2H), 7.62 – 7.57 (m, 3H), 7.56 – 7.53 (m, 2H), 7.47 – 7.26 (m, 6H), 7.14 – 7.09 (m, 4H), 7.06 – 6.99 (m, 2H), 3.20 (s, 1H), 2.50 (d, *J* = 12.6 Hz, 1H), 2.38 (d, *J* = 12.6 Hz, 1H), 1.13 (s, 3H), 0.99 (s, 3H), 0.55 (s, 3H), 0.22 (s, 3H); **^{13}C NMR** (100 MHz, $CDCl_3$) δ 176.7,

149.3, 141.3, 140.1, 134.7, 134.3, 131.2, 129.5, 128.6, 128.4, 128.2, 127.5, 127.0, 126.9, 126.8, 125.2, 124.4, 79.4, 56.4, 50.8, 49.1, 26.6, 26.4, -1.8. HRMS (ESI) m/z calcd. for $C_{33}H_{36}NSi$ $[M+H]^+$ = 474.2612, found = 474.2625.

HPLC conditions: Daicel Chiralpak IG-OZH column; hexane/2-propanol = 99.7/0.3, 0.5 mL/min, 25 °C. Retention times: 15.66 min (major), 15.01 min (minor).

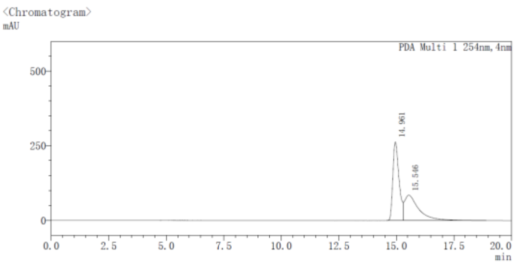

<Peak Table>
PDA Ch1 254nm

No.	Ret. Time (min)	Height (mAU)	Height%	Area (mAU*min)	Area%
1	14.961	263349	75.597	4949367	57.198
2	15.546	85009	24.403	3703686	42.802
Total		348358	100.000	8653053	100.000

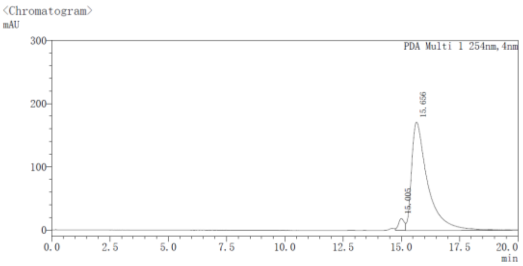

<Peak Table>
PDA Ch1 254nm

No.	Ret. Time (min)	Height (mAU)	Height%	Area (mAU*min)	Area%
1	15.005	18712	9.860	326237	3.811
2	15.656	171067	90.140	8233945	96.189
Total		189779	100.000	8560181	100.000

(*R*)-2-((*R*)-(Dimethyl(phenyl)silyl)(phenyl)methyl)-2,4,4-trimethyl-5-(*p*-tolyl)-3,4-dihydro-2*H*-pyrrole (3af)

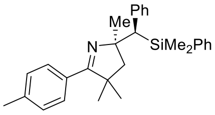

Following **General procedure B**, isolated **3af** 88.0 mg, 69% yield, 92% ee, as colorless oil, $[\alpha]_D^{20}$ = +8.4 (c = 0.5, $CHCl_3$). **^{1}H NMR** (400 MHz, $CDCl_3$) δ 7.64 – 7.55 (m, 4H), 7.40 – 7.27 (m, 7H), 7.20 (d, *J* = 7.8 Hz, 3H), 2.75 (s, 1H), 2.43 (s, 3H), 2.20 (d, *J* = 13.2 Hz, 1H), 1.65 (d, *J* = 13.2 Hz, 1H), 1.42 (s, 3H), 1.34 (s, 3H), 1.16 (s, 3H), 0.48 (s, 3H), 0.29 (s, 3H); **^{13}C NMR** (100 MHz, $CDCl_3$) δ 174.4, 141.8, 140.2, 139.0, 134.3, 132.0, 131.3, 128.7, 128.5, 128.1, 127.6, 127.4, 125.1, 75.2, 53.9, 50.4, 30.0, 29.4, 28.1, 21.3, -0.1, -1.4. HRMS (ESI) m/z calcd. for $C_{29}H_{36}NSi$ $[M+H]^+$ = 426.2612, found = 426.2614.

HPLC conditions: Daicel Chiralpak ODH column; hexane/2-propanol = 99.7/0.3, 0.7 mL/min, 25 °C. Retention times: 7.49 min (major), 8.71 min (minor).

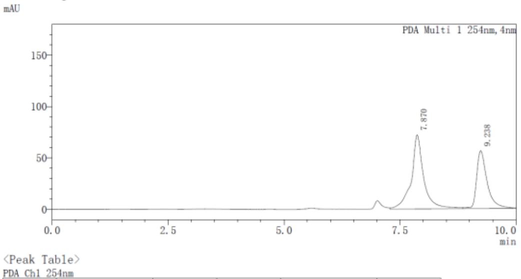

<Peak Table>
PDA Ch1 254nm

No.	Ret. Time (min)	Height (mAU)	Height%	Area (mAU*min)	Area%
1	7.870	72022	56.122	1346796	60.021
2	9.238	56308	43.878	897076	39.979
Total		128330	100.000	2243872	100.000

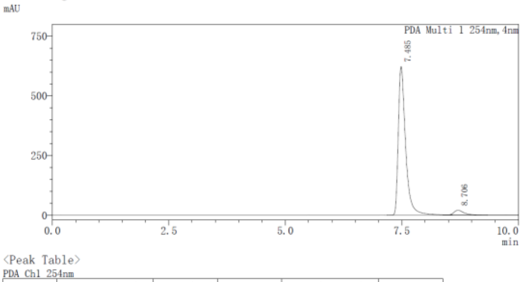

<Peak Table>
PDA Ch1 254nm

No.	Ret. Time (min)	Height (mAU)	Height%	Area (mAU*min)	Area%
1	7.485	622922	96.868	6876462	95.879
2	8.706	20141	3.132	295522	4.121
Total		643062	100.000	7171984	100.000

(*R*)-2-((*R*)-(Dimethyl(phenyl)silyl)(phenyl)methyl)-5-(4-methoxyphenyl)-2,4,4-trimethyl-

3,4-dihydro-2*H*-pyrrole (3ag)

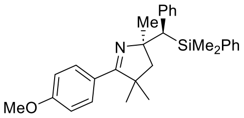

Following **General procedure B**, isolated **3ag** 86.0 mg, 65% yield, 91% ee, as colorless oil, $[\alpha]_D^{20}$ = +29.8 (c = 0.7, $CHCl_3$). **^{1}H NMR** (400 MHz, $CDCl_3$) δ 7.74 (d, *J* = 8.5 Hz, 2H), 7.70 – 7.59 (m, 2H), 7.49 – 7.24 (m, 8H), 6.98 (d, *J* = 8.4 Hz, 2H), 3.96 (s, 3H), 2.79 (s, 1H), 2.25 (d, *J* = 13.2 Hz, 1H), 1.71 (d, *J* = 13.2 Hz, 1H), 1.50 (s, 3H), 1.40 (s, 3H), 1.25 (s, 3H), 0.55 (s, 3H), 0.34 (s, 3H); **^{13}C NMR** (100 MHz, $CDCl_3$) δ 173.6, 160.3, 141.8, 140.2, 134.3, 131.3, 129.7, 128.4, 127.6, 127.4, 127.3, 125.1, 113.3, 74.9, 55.2, 54.2, 50.5, 50.2, 30.0, 29.5, 28.2, -0.1, -1.3. HRMS (ESI) m/z calcd. for $C_{29}H_{36}NOSi$ $[M+H]^+$ = 442.2561, found = 442.2571.

HPLC conditions: Daicel Chiralpak IG column; hexane/2-propanol = 99.7/0.3, 0.7 mL/min, 25 °C. Retention times: 19.11 min (major), 14.68 min (minor).

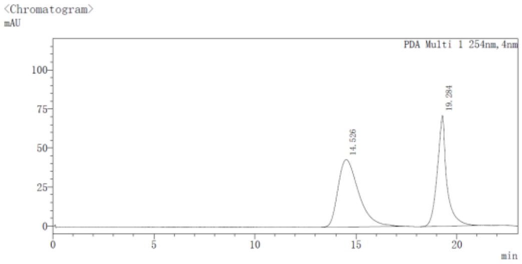

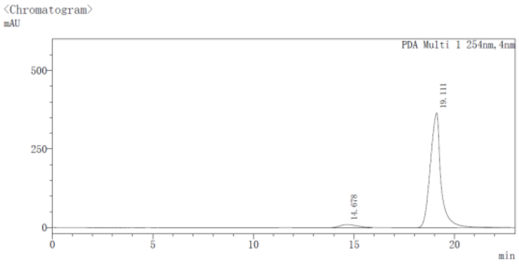

<Peak Table>
PDA Ch1 254nm

No.	Ret.Time(min)	Height(mAU)	Height%	Area(mAU*min)	Area%
1	14.526	43136	37.846	3078048	57.029
2	19.284	70840	62.154	2319324	42.971
Total		113976	100.000	5397372	100.000

<Peak Table>
PDA Ch1 254nm

No.	Ret.Time(min)	Height(mAU)	Height%	Area(mAU*min)	Area%
1	14.678	10395	2.763	666072	4.676
2	19.111	365822	97.237	13578358	95.324
Total		376217	100.000	14244430	100.000

(*R*)-5-(4-(*tert*-Butoxy)phenyl)-2-((*R*)-(dimethyl(phenyl)silyl)(phenyl)methyl)-2,4,4-trimethyl-3,4-dihydro-2*H*-pyrrole (3ah)

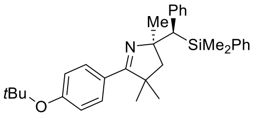

Following **General procedure**, isolated **3ah** 88.4 mg, 61% yield, 90% ee, as colorless oil, $[\alpha]_D^{20}$ = +40.3 (c = 1.3, $CHCl_3$). **^{1}H NMR** (400 MHz, $CDCl_3$) δ 7.63 – 7.54 (m, 4H), 7.36 – 7.28 (m, 7H), 7.23 – 7.18 (m, 1H), 7.03 – 6.96 (m, 2H), 2.73 (s, 1H), 2.18 (d, *J* = 13.2 Hz, 1H), 1.65 (d, *J* = 13.2 Hz, 1H), 1.44 (s, 12H), 1.35 (s, 3H), 1.15 (s, 3H), 0.51 (s, 3H), 0.27 (s, 3H); **^{13}C NMR** (100 MHz, $CDCl_3$) δ 174.1, 156.4, 141.8, 140.2, 134.2, 131.3, 129.6, 129.0, 128.4, 127.6, 127.4, 125.2, 123.2, 78.8, 75.1, 54.1, 50.5, 50.3, 30.0, 29.7, 29.5, 28.9, 28.2, -0.2, -1.4. HRMS (ESI) m/z calcd. for $C_{32}H_{42}NOSi$ $[M+H]^+$ = 484.3030, found = 484.3040.

HPLC conditions: Daicel Chiralpak OZ-3 column; hexane/2-propanol = 99.7/0.3, 0.7 mL/min, 25 °C. Retention times: 9.72 min (major), 7.88 min (minor).

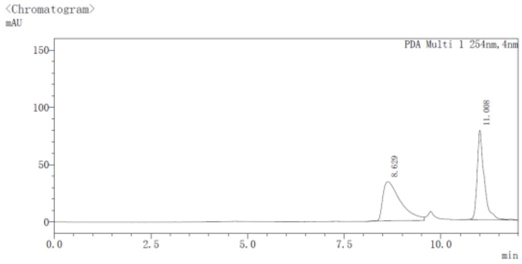

<Peak Table>
PDA Ch1 254nm

No.	Ret. Time(min)	Height(mAU)	Height%	Area(mAU*min)	Area%
1	8.629	34194	30.495	1092026	52.600
2	11.008	77935	69.505	984053	47.400
Total		112129	100.000	2076078	100.000

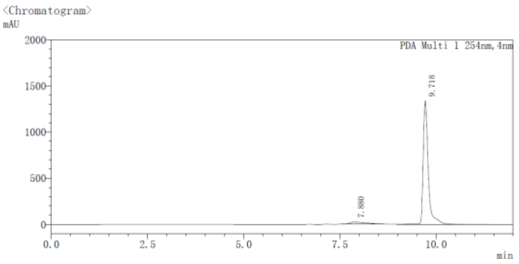

<Peak Table>
PDA Ch1 254nm

No.	Ret. Time(min)	Height(mAU)	Height%	Area(mAU*min)	Area%
1	7.880	21904	1.612	627986	4.852
2	9.718	1336652	98.388	12315442	95.148
Total		1358557	100.000	12943428	100.000

(*R*)-2-((*R*)-(Dimethyl(phenyl)silyl)(phenyl)methyl)-2,4,4-trimethyl-5-(4-phenoxyphenyl)-3,4-dihydro-2*H*-pyrrole (3ai)

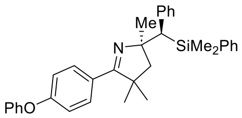

Following **General procedure B**, isolated **3ai** 117.8 mg, 78% yield, 90% ee, as colorless oil, $[\alpha]_D^{20}$ = +118.9 (c = 2.0, $CHCl_3$). **^{1}H NMR** (400 MHz, $CDCl_3$) δ 7.77 – 7.73 (m, 2H), 7.71 – 7.65 (m, 2H), 7.53 – 7.48 (m, 2H), 7.44 – 7.35 (m, 7H), 7.33 – 7.26 (m, 2H), 7.23 – 7.16 (m, 2H), 7.12 – 7.06 (m, 2H), 2.83 (s, 1H), 2.29 (d, *J* = 13.3 Hz, 1H), 1.75 (d, *J* = 13.2 Hz, 1H), 1.52 (s, 3H), 1.44 (s, 3H), 1.27 (s, 3H), 0.60 (s, 3H), 0.36 (s, 3H); **^{13}C NMR** (100 MHz, $CDCl_3$) δ 173.6, 158.2, 156.7, 141.7, 140.2, 134.2, 131.2, 129.8, 129.8, 129.6, 128.4, 127.6, 127.4, 125.2, 123.6, 119.3, 117.9, 75.1, 54.1, 50.5, 50.3, 30.0, 29.4, 28.2, -1.4. HRMS (ESI) m/z calcd. for $C_{34}H_{38}NOSi$ $[M+H]^+$ = 504.2717, found = 504.2713. HPLC conditions: Daicel Chiralpak OZ-3 column; hexane/2-propanol = 99.5/0.5, 0.7 mL/min, 25 ^{o}C. Retention times: 6.12 min (major), 17.56 min (minor).

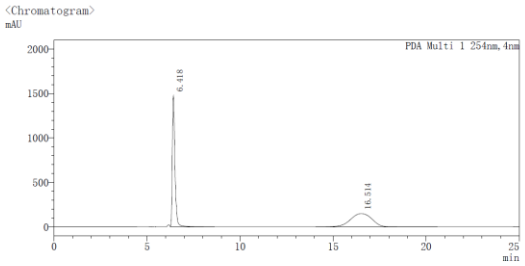

<Peak Table>
PDA Ch1 254nm

No.	Ret. Time(min)	Height(mAU)	Height%	Area(mAU*min)	Area%
1	6.418	1473767	90.790	14681766	54.914
2	16.514	149508	9.210	12054048	45.086
Total		1623275	100.000	26735814	100.000

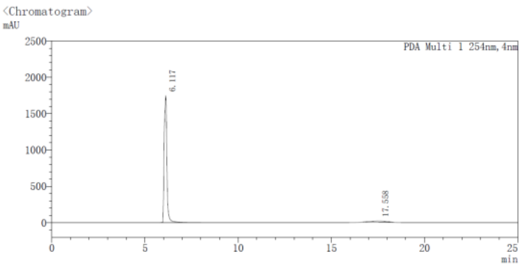

<Peak Table>
PDA Ch1 254nm

No.	Ret. Time(min)	Height(mAU)	Height%	Area(mAU*min)	Area%
1	6.117	1744199	99.096	17871541	95.084
2	17.558	15913	0.904	924004	4.916
Total		1760112	100.000	18795545	100.000

(*R*)-5-([1,1'-Biphenyl]-4-yl)-2-((*R*)-(dimethyl(phenyl)silyl)(phenyl)methyl)-2,4,4-trimethyl-3,4-dihydro-2*H*-pyrrole (3aj)

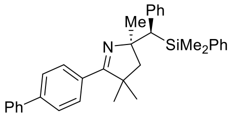

Following **General procedure B**, isolated **3aj** 87.7 mg, 60% yield, 91% ee, as colorless oil, $[\alpha]_D^{20}$ = +93.1 (c = 1.4, $CHCl_3$). **^{1}H NMR** (400 MHz, $CDCl_3$) δ 7.64 – 7.57 (m, 2H), 7.55 – 7.52 (m, 2H), 7.49 – 7.44 (m, 4H), 7.39 – 7.35 (m, 2H), 7.30 – 7.27(m, 1H), 7.22 – 7.13 (m, 7H), 7.09 – 7.05 (m, 1H), 2.64 (s, 1H), 2.09 (d, *J* = 13.3

Hz, 1H), 1.54 (d, *J* = 13.2 Hz, 1H), 1.32 (s, 3H), 1.23 (s, 3H), 1.06 (s, 3H), 0.38 (s, 3H), 0.15 (s, 3H); **^{13}C NMR** (100 MHz, $CDCl_3$) δ 174.2, 141.7, 140.6, 140.2, 134.3, 133.8, 131.3, 128.8, 128.6, 128.5, 127.6, 127.5, 127.1, 126.7, 125.2, 75.4, 53.9, 50.5, 50.4, 30.0, 29.4, 28.2, -0.1, -1.4. HRMS (ESI) m/z calcd. for $C_{34}H_{38}NSi$ $[M+H]^+$ = 488.2768, found = 488.2780.

HPLC conditions: Daicel Chiralpak ADH-ADH column; hexane/2-propanol = 99.5/0.5, 0.7 mL/min, 25 ^oC. Retention times: 22.26 min (major), 19.58 min (minor).

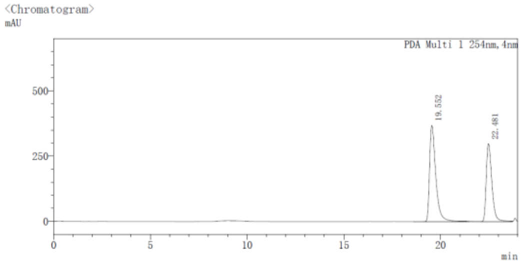

<Peak Table>
PDA Ch1 254nm

No.	Ret. Time (min)	Height (mAU)	Height%	Area (mAU*min)	Area%
1	19.552	369148	55.269	8338201	57.914
2	22.481	298759	44.731	6059367	42.086
Total		667907	100.000	14397568	100.000

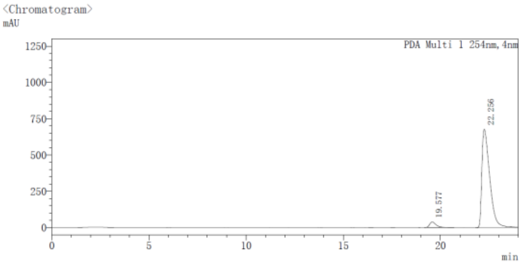

<Peak Table>
PDA Ch1 254nm

No.	Ret. Time (min)	Height (mAU)	Height%	Area (mAU*min)	Area%
1	19.577	38385	5.361	923439	4.600
2	22.256	677557	94.639	19149367	95.400
Total		715942	100.000	20072806	100.000

(*R*)-5-(2,3-Dihydrobenzo[*b*][1,4]dioxin-6-yl)-2-((*R*)-(dimethyl(phenyl)silyl)(phenyl)methyl)-2,4,4-trimethyl-3,4-dihydro-2*H*-pyrrole (3ak)

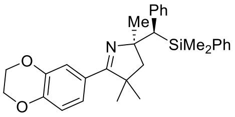

Following **General procedure**, isolated **3ak** 109.8 mg, 78% yield, 92% ee, as colorless oil, $[\alpha]_D^{20}$ = +25.9 (c = 1.1, $CHCl_3$). **^{1}H NMR** (400 MHz, $CDCl_3$) δ 7.68 – 7.62 (m, 2H), 7.46 – 7.26 (m, 10H), 6.95 (d, *J* = 8.5 Hz, 1H), 4.40 (s, 4H), 2.78 (s, 1H), 2.24 (d, *J* = 13.2 Hz, 1H), 1.71 (d, *J* = 13.2 Hz, 1H), 1.49 (s, 3H), 1.38 (s, 3H), 1.24 (s, 3H), 0.54 (s, 3H), 0.35 (s, 3H); **^{13}C NMR** (100 MHz, $CDCl_3$) δ 173.5, 144.6, 142.9, 141.8, 140.2, 134.3, 131.3, 128.5, 127.6, 127.5, 125.2, 121.9, 117.4, 116.7, 75.0, 64.6, 64.3, 54.2, 50.5, 50.2, 30.0, 29.5, 28.3, -0.1, -1.3. HRMS (ESI) m/z calcd. for $C_{30}H_{36}NO_2Si$ $[M+H]^+$ = 470.2510, found = 470.2513.

HPLC conditions: Daicel Chiralpak OZ-3 column; hexane/2-propanol = 99.9/0.1, 0.7 mL/min, 25 ^oC. Retention times: 9.47 min (major), 8.12 min (minor).

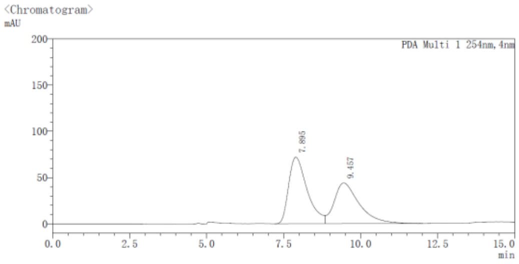

<Peak Table>
PDA Ch1 254nm

No.	Ret. Time(min)	Height(mAU)	Height%	Area(mAU*min)	Area%
1	7.895	71974	62.075	3014881	54.887
2	9.457	43973	37.925	2478053	45.113
Total		115947	100.000	5492934	100.000

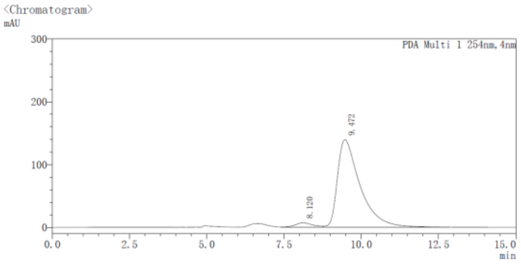

<Peak Table>
PDA Ch1 254nm

No.	Ret. Time(min)	Height(mAU)	Height%	Area(mAU*min)	Area%
1	8.120	6941	4.746	277471	3.754
2	9.472	139306	95.254	7114582	96.246
Total		146247	100.000	7392053	100.000

(*R*)-2-((*R*)-(dimethyl(phenyl)silyl)(phenyl)methyl)-2,4,4-trimethyl-5-(*m*-tolyl)-3,4-dihydro-2*H*-pyrrole (3al)

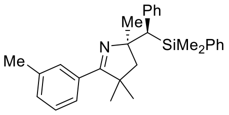

Following **General procedure B**, isolated **3al** 80.4 mg, 63% yield, 94% ee, as colorless oil, $[\alpha]_D^{20}$ = +25.0 (c = 1.3, $CHCl_3$). **^{1}H NMR** (400 MHz, $CDCl_3$) δ 7.63 – 7.59 (m, 2H), 7.48 – 7.27 (m, 10H), 7.23 (d, *J* = 8.6 Hz, 2H), 2.82 (s, 1H), 2.43 (s, 3H), 2.23 (d, *J* = 13.3 Hz, 1H), 1.68 (d, *J* = 13.2 Hz, 1H), 1.42 (s, 3H), 1.36 (s, 3H), 1.12 (s, 3H), 0.51 (s, 3H), 0.30 (s, 3H); **^{13}C NMR** (100 MHz, $CDCl_3$) δ 174.9, 141.8, 140.1, 137.5, 135.0, 134.3, 131.4, 129.7, 128.9, 128.5, 127.9, 127.7, 127.6, 127.4, 125.2, 125.0, 75.3, 53.3, 50.7, 50.2, 30.3, 29.5, 28.0, 21.5, -0.1, -1.4. HRMS (ESI) m/z calcd. for $C_{29}H_{36}NSi$ $[M+H]^+$ = 426.2612, found = 426.2605.

HPLC conditions: Daicel Chiralpak IBN-5-IBN-5 column; hexane/2-propanol = 99.8/0.2, 0.6 mL/min, 25 °C. Retention times: 19.60 min (major), 18.33 min (minor).

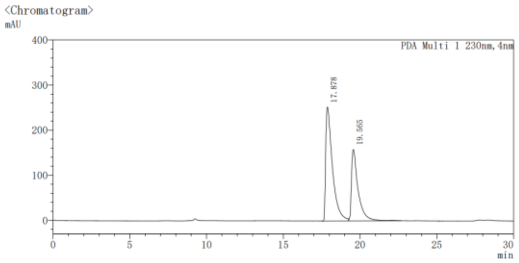

<Peak Table>
PDA Ch1 230nm

No.	Ret. Time(min)	Height(mAU)	Height%	Area(mAU*min)	Area%
1	17.878	252917	61.498	7758301	61.981
2	19.565	158344	38.502	4758942	38.019
Total		411261	100.000	12517243	100.000

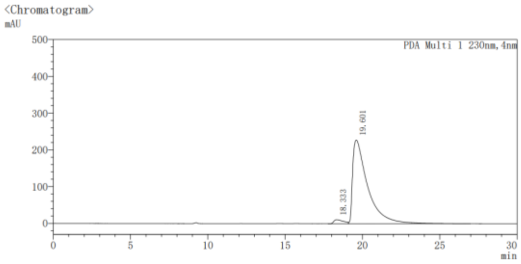

<Peak Table>
PDA Ch1 230nm

No.	Ret. Time(min)	Height(mAU)	Height%	Area(mAU*min)	Area%
1	18.333	10988	4.602	475932	2.996
2	19.601	227766	95.398	15411315	97.004
Total		238754	100.000	15887247	100.000

(*R*)-2-((*R*)-(Dimethyl(phenyl)silyl)(phenyl)methyl)-5-(3-methoxyphenyl)-2,4,4-trimethyl-3,4-dihydro-2*H*-pyrrole (3am)

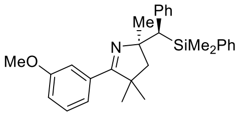

Following **General procedure B**, isolated **3am** 80.7 mg, 61% yield, 91% ee, as colorless oil, $[\alpha]_D^{20}$ = +19.3 (c = 1.0, $CHCl_3$). **^{1}H NMR** (400 MHz, $CDCl_3$) δ 7.69 – 7.62 (m, 2H), 7.55 – 7.27 (m, 11H), 7.04 (d, *J* = 8.2 Hz, 1H), 3.95 (s, 3H), 2.86 (s, 1H), 2.29 (d, *J* = 12.8 Hz, 1H), 1.75 (d, *J* = 13.4 Hz, 1H), 1.49 (s, 3H), 1.41 (s, 3H), 1.19 (s,

3H), 0.55 (s, 3H), 0.39 (s, 3H); **^{13}C NMR** (100 MHz, $CDCl_3$) δ 174.6, 159.2, 141.7, 140.0, 134.3, 131.3, 128.9, 128.5, 127.6, 127.4, 125.2, 120.4, 115.0, 113.5, 75.3, 55.2, 53.5, 50.6, 50.3, 30.2, 29.4, 28.0, -0.1, -1.4. HRMS (ESI) m/z calcd. for $C_{29}H_{36}NOSi$ $[M+H]^+$ = 442.2561, found = 442.2567.

HPLC conditions: Daicel Chiralpak ADH-ADH column; hexane/2-propanol = 99.5/0.5, 0.7 mL/min, 25 ^{o}C. Retention times: 15.03 min (major), 15.73 min (minor).

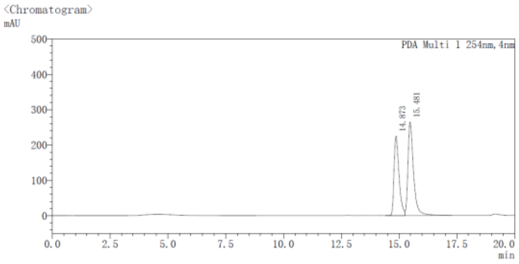

<Peak Table>
PDA Ch1 254nm

No.	Ret.Time(min)	Height(mAU)	Height%	Area(mAU*min)	Area%
1	14.873	224645	45.945	3363684	43.242
2	15.481	264300	54.055	4415001	56.758
Total		488945	100.000	7778684	100.000

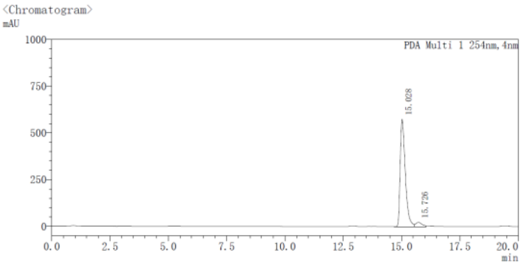

<Peak Table>
PDA Ch1 254nm

No.	Ret.Time(min)	Height(mAU)	Height%	Area(mAU*min)	Area%
1	15.028	573381	96.161	8701989	95.382
2	15.726	22892	3.839	421323	4.618
Total		596273	100.000	9123312	100.000

(*R*)-2-((*R*)-(Dimethyl(phenyl)silyl)(phenyl)methyl)-2-ethyl-4,4-dimethyl-5-phenyl-3,4-dihydro-2*H*-pyrrole (3an)

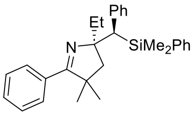

Following **General procedure B**, CsOAc instead of Rb_2CO_3, isolated **3an** 67.6 mg, 53% yield, 90% ee, as colorless oil, $[\alpha]_D^{20}$ = -40.6 (c = 1.1, $CHCl_3$). **^{1}H NMR** (400 MHz, $CDCl_3$) δ 7.67 – 7.65 (m, 2H), 7.59 – 7.56 (m, 2H), 7.45 – 7.38 (m, 3H), 7.36 – 7.27 (m, 7H), 7.23 – 7.17 (m, 1H), 2.83 (s, 1H), 2.05 (d, *J* = 13.6 Hz, 1H), 1.79 (s, 1H), 1.66 – 1.57 (m, 2H), 1.34 (s, 3H), 1.12 (s, 3H), 0.81 – 0.71 (m, 3H), 0.45 (s, 3H), 0.22 (s, 3H); **^{13}C NMR** (100 MHz, $CDCl_3$) δ 175.2, 141.5, 140.4, 135.1, 134.2, 131.7, 128.9, 128.4, 128.1, 127.9, 127.5, 127.4, 125.2, 78.7, 50.5, 48.8, 48.1, 32.7, 28.4, 28.3, 8.8, -0.2, -1.4. HRMS (ESI) m/z calcd. for $C_{29}H_{36}NSi$ $[M+H]^+$ = 426.2612, found = 426.2616.

HPLC conditions: Daicel Chiralpak OZ-3 column; hexane/2-propanol = 100/0, 0.7 mL/min, 25 ^{o}C. Retention times: 28.52 min (major), 16.47 min (minor).

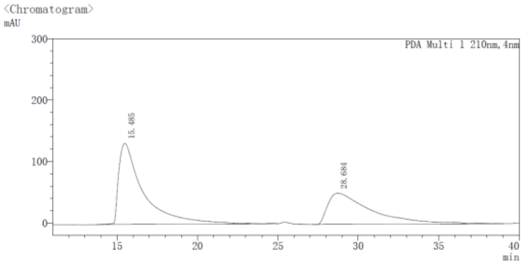

<Peak Table>
PDA Ch1 210nm

No.	Ret. Time (min)	Height (mAU)	Height%	Area (mAU*min)	Area%
1	15.485	132251	72.227	14217347	59.709
2	28.684	50854	27.773	9593695	40.291
Total		183105	100.000	23811043	100.000

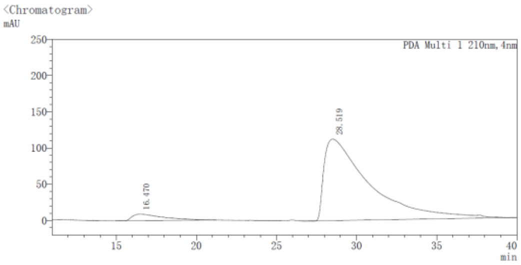

<Peak Table>
PDA Ch1 210nm

No.	Ret. Time (min)	Height (mAU)	Height%	Area (mAU*min)	Area%
1	16.470	8890	7.330	1197520	5.101
2	28.519	112391	92.670	22279275	94.899
Total		121282	100.000	23476795	100.000

(*R*)-2-((*S*)-(Dimethyl(phenyl)silyl)(phenyl)methyl)-2-ethyl-4,4-dimethyl-5-phenyl-3,4-dihydro-2H-pyrrole (3ao)

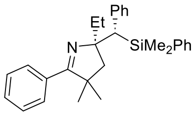

Following **General procedure B**, CsOAc instead of Rb_2CO_3, isolated **3ao** 72.7 mg, 57% yield, 20% ee, as colorless oil, $[\alpha]_D^{20}$ = -56.3 (c = 1.0, $CHCl_3$). **^{1}H NMR** (400 MHz, $CDCl_3$) δ 7.85 – 7.76 (m, 2H), 7.53 – 7.50 (m, 2H), 7.44 – 7.37 (m, 3H), 7.35 – 7.27 (m, 3H), 7.17 – 6.97 (m, 5H), 2.78 (d, *J* = 1.7 Hz, 1H), 1.90 – 1.69 (m, 3H), 1.65 – 1.54 (m, 1H), 1.34 (d, *J* = 1.5 Hz, 3H), 0.75 (ddd, *J* = 15.2, 7.5, 1.6 Hz, 6H), 0.60 (t, *J* = 1.4 Hz, 3H), 0.24 – 0.16 (m, 3H); **^{13}C NMR** (100 MHz, $CDCl_3$) δ 175.1, 142.1, 140.7, 134.2, 131.2, 129.0, 128.4, 128.3, 128.1, 127.6, 127.3, 125.0, 79.6, 50.5, 49.7, 46.9, 35.2, 29.4, 26.8, 9.1, -0.5, -1.3. HRMS (ESI) m/z calcd. for $C_{29}H_{36}NSi$ $[M+H]^+$ = 426.2612, found = 426.2604.

HPLC conditions: Daicel Chiralpak OZ-3 column; hexane/2-propanol = 100/0, 0.7 mL/min, 25 °C. Retention times: 7.62 min (major), 8.57 min (minor).

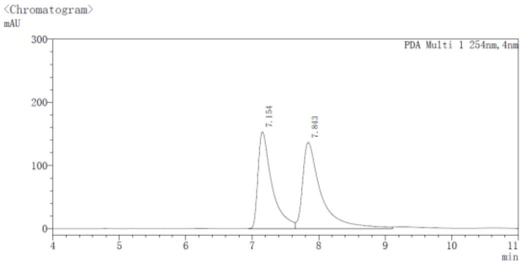

<Peak Table>
PDA Ch1 254nm

No.	Ret. Time (min)	Height (mAU)	Height%	Area (mAU*min)	Area%
1	7.154	153308	52.847	2251932	45.895
2	7.843	136788	47.153	2654786	54.105
Total		290095	100.000	4906718	100.000

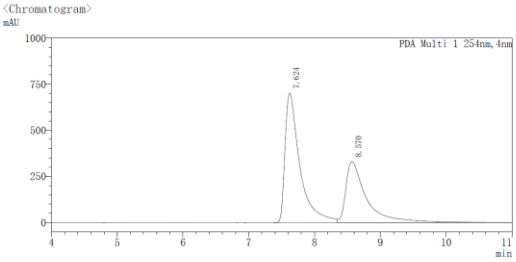

<Peak Table>
PDA Ch1 254nm

No.	Ret. Time (min)	Height (mAU)	Height%	Area (mAU*min)	Area%
1	7.624	703060	67.966	11029982	60.053
2	8.570	331376	32.034	7337208	39.947
Total		1034436	100.000	18367190	100.000

5. Synthetic applications of the products and characterization of products

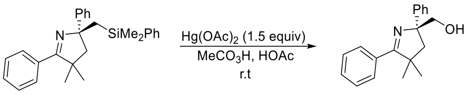

3a **4**, 55% yield, 90% ee

The reaction was performed according to a literature procedure.[5] Mercuric acetate (95.6 mg, 0.3 mmol) as added to a stirred solution of the **3a** (79.5 mg, 0.2 mmol) in peracetic acid (8 mmol) , and the mixture kept for 12 h at room temperature. Ether (10 ml) was added and the solution washed with sodium thiosulphate solution, water, sodium hydrogen carbonate solution, and brine, dried (Na_2S0_4), and evaporated in vacua. The resulting oil was purified by preparative thin layer chromatography eluting with hexan:elethyl acetate (1:l) to give the **4** in 55% yield, 90% ee.

(*S*)-(4,4-Dimethyl-2,5-diphenyl-3,4-dihydro-2*H*-pyrrol-2-yl)methanol (4)

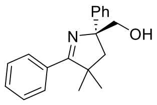

As colorless oil, $[\alpha]_D^{20}$ = -13.9 (c = 1.0, $CHCl_3$). **^{1}H NMR** (400 MHz, $CDCl_3$) δ 7.80 – 7.73 (m, 2H), 7.47 – 7.39 (m, 5H), 7.36 – 7.31 (m, 2H), 7.26 – 7.20 (m, 1H), 3.95 (d, *J* = 11.3 Hz, 1H), 3.65 (dd, *J* = 11.3, 5.6 Hz, 1H), 2.49 (dd, *J* = 12.7, 2.5 Hz, 1H), 2.23 (dd, *J* = 12.7, 4.4 Hz, 1H), 1.41 (d, *J* = 11.5 Hz, 3H), 1.10 (d, *J* = 8.1 Hz, 3H); **^{13}C NMR** (100 MHz, $CDCl_3$) δ 181.7, 145.9, 134.5, 129.8, 128.3, 128.2, 126.7, 126.0, 78.6, 70.9, 51.7, 49.5, 27.6, 27.5. HRMS (ESI) m/z calcd. for $C_{19}H_{21}NNaO$ $[M+Na]^+$ = 302.1515, found = 302.1523.

HPLC conditions: Daicel Chiralpak IG column; hexane/2-propanol = 98/2, 1.0 mL/min, 25 ^oC. Retention times: 18.10 min (major), 27.39 min (minor).

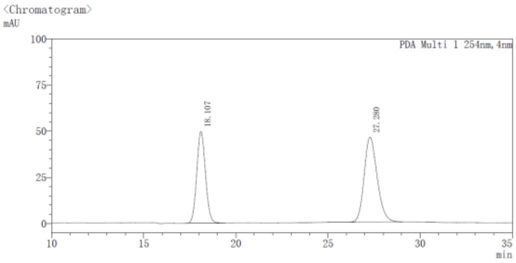

<Peak Table>
PDA Ch1 254nm

No.	Ret.Time(min)	Height(mAU)	Height%	Area(mAU*min)	Area%
1	18.107	49671	51.922	1628921	41.911
2	27.280	45993	48.078	2257720	58.089
Total		95663	100.000	3886641	100.000

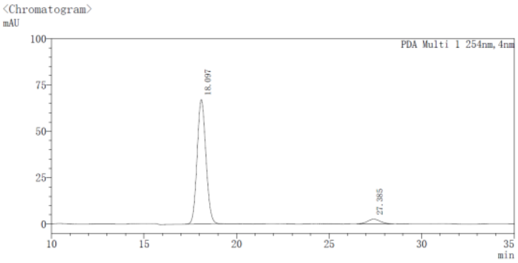

<Peak Table>
PDA Ch1 254nm

No.	Ret.Time(min)	Height(mAU)	Height%	Area(mAU*min)	Area%
1	18.097	67156	96.381	2223831	94.851
2	27.385	2522	3.619	120717	5.149
Total		69678	100.000	2344548	100.000

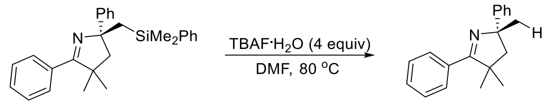

3a **5**, 87% yield, 90% ee

The reaction was performed according to a literature procedure.[6] To a solution of **3a** (79.5 mg, 0.2 mmol) in anhydrous DMF (2.0 mL) was added solid TBAF·H_2O (1.12 g, 4 mmol), and the reaction mixture was heated at 80 ℃ for 16 h. After the addition of sat. aq NH_4Cl, the reaction mixture was processed with EtOAc. The resulting crude material was purified by silica gel chromatography to afford **5** in 87% yield, 90% ee.

(*S*)-2,4,4-Trimethyl-2,5-diphenyl-3,4-dihydro-2*H*-pyrrole (5)

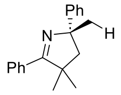

As colorless oil, $[\alpha]_D^{20}$ = -150.8 (c = 1.0, $CHCl_3$). **^{1}H NMR** (400 MHz, $CDCl_3$) δ 7.87 – 7.76 (m, 2H), 7.52 – 7.47 (m, 2H), 7.45 – 7.38 (m, 3H), 7.37 – 7.30 (m, 2H), 7.24 – 7.18 (m, 1H), 2.36 (d, *J* = 12.8 Hz, 1H), 2.25 (d, *J* = 12.8 Hz, 1H), 1.70 (s, 3H), 1.47 (s, 3H), 1.17 (s, 3H); **^{13}C NMR** (100 MHz, $CDCl_3$) δ 177.7, 150.4, 135.0, 129.4, 128.2, 128.1, 126.0, 125.2, 73.9, 55.9, 51.4, 32.4, 28.3, 27.5. HRMS (ESI) m/z calcd. for $C_{19}H_{21}NNa$ $[M+Na]^+$ = 286.1566, found = 286.1566.

HPLC conditions: Daicel Chiralpak IBN-5 column; hexane/2-propanol = 99/1, 0.7 mL/min, 25 °C. Retention times: 7.73 min (major), 5.12 min (minor).

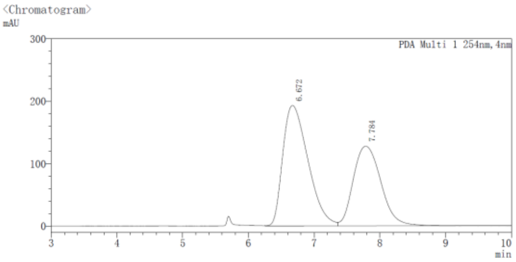

<Peak Table>
PDA Ch1 254nm

No.	Ret.Time(min)	Height(mAU)	Height%	Area(mAU*min)	Area%
1	6.672	192900	60.202	5136032	57.964
2	7.784	127522	39.798	3724749	42.036
Total		320422	100.000	8860781	100.000

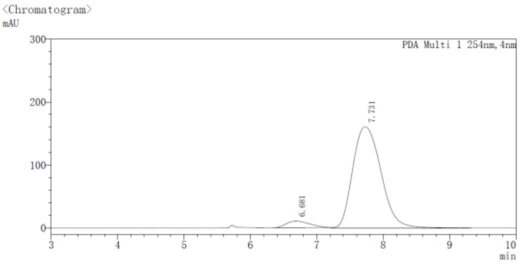

<Peak Table>
PDA Ch1 254nm

No.	Ret.Time(min)	Height(mAU)	Height%	Area(mAU*min)	Area%
1	6.681	10394	6.072	255899	5.118
2	7.731	160772	93.928	4744386	94.882
Total		171166	100.000	5000285	100.000

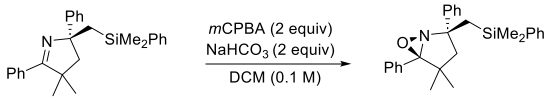

3a **6**, 76% yield, 88% ee

The reaction was performed according to a literature procedure.[7] A solution of **3a** (79.4 mg, 0.2 mmol, 1 equiv), *m*CPBA (69.0 mg, 0.4 mmol, 2 equiv) and $NaHCO_3$ (33.6 mg, 0.4 mmol, 2equiv) in DCM (2 mL) were stirred at room temperature for 12 h. The resulting mixture was concentrated under reduced pressure. The residue was purified through column chromatography on silica gel

(EtOAc/petroleum ether = 1:6) to give **6** as a colorless oil in 76% yield (62.8 mg, >20:1 dr) and 88% ee.

(2*S*,5*S*)-2-((Dimethyl(phenyl)silyl)methyl)-4,4-dimethyl-2,5-diphenyl-6-oxa-1-azabicyclo[3.1.0]hexane (6)

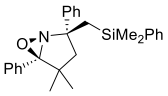

As colorless oil, $[\alpha]_D^{20}$ = -45.6 (c = 0.5, $CHCl_3$). **^{1}H NMR** (400 MHz, $CDCl_3$) δ 7.73 – 7.64 (m, 2H), 7.50 – 7.27 (m, 13H), 2.27 (d, *J* = 13.1 Hz, 1H), 1.91 – 1.76 (m, 2H), 1.69 (d, *J* = 14.5 Hz, 1H), 1.27 (s, 3H), 0.63 (s, 3H), 0.15 (s, 3H), -0.02 (s, 3H)； **^{13}C NMR** (100 MHz, $CDCl_3$) δ 144.3, 139.7, 134.2, 133.5, 128.6, 128.4, 128.2, 127.8, 127.5, 127.5, 127.0, 126.8, 94.2, 72.7, 49.1, 43.2, 32.5, 25.1, 24.1, -1.7, -2.5. HRMS (ESI) m/z calcd. for $C_{27}H_{31}NNaOSi$ $[M+Na]^+$ = 436.2067, found = 436.2062.

HPLC conditions: Daicel Chiralpak ID column; hexane/2-propanol = 99/1, 0.3 mL/min, 25 °C. Retention times: 19.38 min (major), 14.47 min (minor).

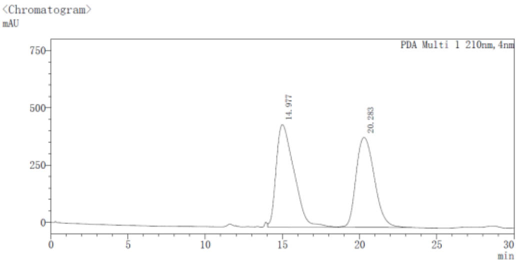

<Peak Table>
PDA Ch1 210nm

No.	Ret. Time (min)	Height (mAU)	Height%	Area (mAU*min)	Area%
1	14.977	446249	53.239	35959162	52.581
2	20.283	391943	46.761	32429551	47.419
Total		838192	100.000	68388713	100.000

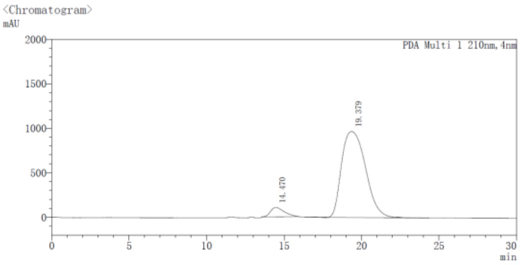

<Peak Table>
PDA Ch1 210nm

No.	Ret. Time (min)	Height (mAU)	Height%	Area (mAU*min)	Area%
1	14.470	105901	9.870	6569286	5.924
2	19.379	967036	90.130	104316599	94.076
Total		1072937	100.000	110885885	100.000

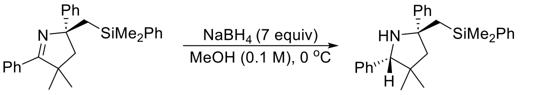

3a **7**, 77% yield, 90% ee

The reaction was performed according to a literature procedure.[8] Prepare a solution of **3a** (0.2 mmol, 89.9 mg) in MeOH (2.0 mL), add $NaBH_4$ (52.9 mg,1.4 mmol) and stir the mixture for 2 hours. After completion of the reaction, water was added. The organic phase was extracted with ethyl acetate. The combined organic layers were washed with water and brine, and dried over anhydrous Na_2SO_4. The resulting solution was concentrated under vacuum and the residue was purified by column chromatography on silica gel (eluent: petroleum ether: ethyl acetate = 20:1) to afford the title product **7** in 77% yield, 90% ee.

(2*S*,5*R*)-2-((Dimethyl(phenyl)silyl)methyl)-4,4-dimethyl-2,5-diphenylpyrrolidine (7)

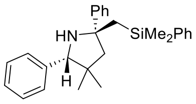

As colorless oil, $[\alpha]_D^{20}$ = +36.7 (c = 0.5, $CHCl_3$). **^{1}H NMR** (400 MHz, $CDCl_3$) δ 7.68 (d, *J* = 7.6 Hz, 2H), 7.48 (d, *J* = 7.3 Hz, 2H), 7.41 (dt, *J* = 4.0, 2.2 Hz, 2H), 7.38 – 7.27 (m, 8H), 7.22 (t, *J* = 7.2 Hz, 1H), 4.26 (s, 1H), 2.34 (d, *J* = 12.6 Hz, 1H), 2.21 (d, *J* = 12.5 Hz, 1H), 1.74 (dd, *J* = 15.0, 4.0 Hz, 3H), 1.04 (s, 3H), 0.41 (s, 3H), 0.02 (s, 3H), -0.09 (s, 3H); **^{13}C NMR** (100 MHz, $CDCl_3$) δ 151.7, 141.2, 139.6, 133.3, 128.8, 127.8, 127.7, 127.5, 126.7, 126.1, 125.5, 70.4, 62.4, 60.4, 42.1, 36.2, 27.0, 23.1, -2.0, -2.3. HRMS (ESI) m/z calcd. for $C_{27}H_{34}NSi$ $[M+H]^+$ = 400.2455, found = 400.2444. HPLC conditions: Daicel Chiralpak OZ-3 column; hexane/2-propanol = 100/0, 0.7 mL/min, 25 °C. Retention times: 25.72 min (major), 14.53 min (minor).

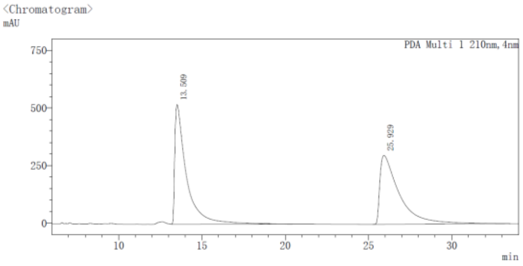

<Peak Table>
PDA Ch1 210nm

No.	Ret.Time(min)	Height(mAU)	Height%	Area(mAU*min)	Area%
1	13.509	518704	63.438	25251051	50.924
2	25.929	298947	36.562	24334408	49.076
Total		817651	100.000	49585459	100.000

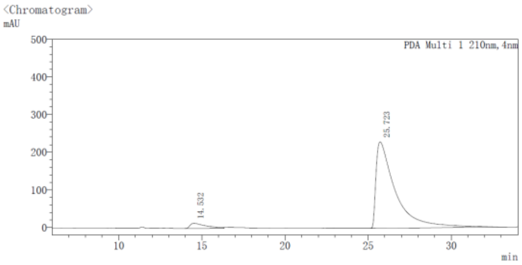

<Peak Table>
PDA Ch1 210nm

No.	Ret.Time(min)	Height(mAU)	Height%	Area(mAU*min)	Area%
1	14.532	13929	5.711	958342	4.837
2	25.723	229947	94.289	18855930	95.163
Total		243875	100.000	19814273	100.000

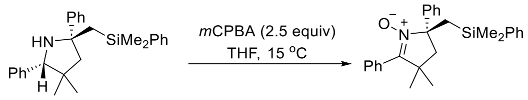

7, 77% yield, 90% ee **8**, 63% yield, 90% ee

The reaction was performed according to a literature procedure.[9] In a reaction tube, **7** (0.2 mmol) was added into THF (2 mL) at 15 °C. Then *m*CPBA (0.5 mmol) was added at the same temperature, and the reaction solution was stirred for 30 min. The solvent was removed under reduced pressure, and the residue was purified by column chromatography on silica gel (eluent: petroleum ether: ethyl acetate = 3:1) to afford the title product **8** in 63% yield, 90% ee.

(*S*)-2-((Dimethyl(phenyl)silyl)methyl)-4,4-dimethyl-2,5-diphenyl-3,4-dihydro-2*H*-pyrrole 1-oxide (8)

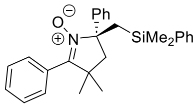

As colorless oil, $[\alpha]_D^{20}$ = -8.59 (c = 0.5, $CHCl_3$). **^{1}H NMR** (400 MHz, $CDCl_3$) δ 7.95 (d, J = 8.2 Hz, 2H), 7.58 – 7.50 (m, 4H), 7.48 – 7.37 (m, 3H), 7.36 – 7.27 (m, 5H), 7.25 – 7.19 (m, 1H), 2.56 (d, *J* = 13.0 Hz, 1H), 2.23 (d, *J* = 13.0 Hz, 1H), 2.03 (d, *J* = 14.8 Hz, 1H), 1.89 (d, *J* = 14.9 Hz, 1H), 1.18 (s, 3H), 1.06 (s, 3H), 0.31 (s, 3H), 0.18 (s, 3H); **^{13}C NMR** (100 MHz, $CDCl_3$) δ 146.6, 144.7, 140.0, 133.6, 129.6,

129.3, 128.7, 128.3, 128.2, 127.7, 127.1, 125.6, 81.3, 50.8, 41.8, 30.9, 28.9, 26.8, -1.6, -1.8.

HRMS (ESI) m/z calcd. for $C_{27}H_{31}NNaOSi$ $[M+Na]^+$ = 436.2067, found = 436.2064.

HPLC conditions: Daicel Chiralpak IBN-5 column; hexane/2-propanol = 97/3, 1.0 mL/min, 25 °C. Retention times: 8.75 min (major) , 7.74 min (minor).

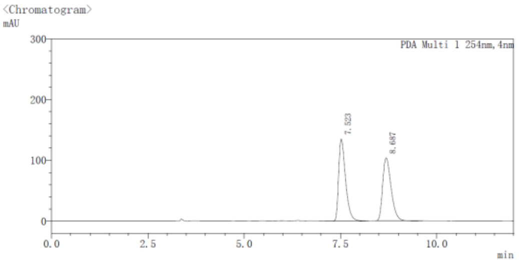

<Peak Table>
PDA Ch1 254nm

No.	Ret. Time(min)	Height(mAU)	Height%	Area(mAU*min)	Area%
1	7.523	134883	56.565	1685447	51.734
2	8.687	103574	43.435	1572481	48.266
Total		238457	100.000	3257928	100.000

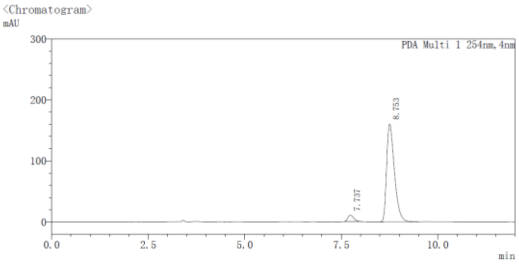

<Peak Table>
PDA Ch1 254nm

No.	Ret. Time(min)	Height(mAU)	Height%	Area(mAU*min)	Area%
1	7.737	10871	6.340	120101	5.056
2	8.753	160591	93.660	2255436	94.944
Total		171462	100.000	2375537	100.000

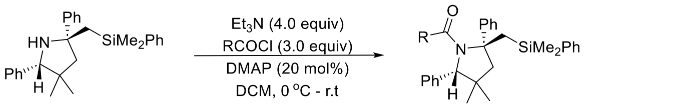

7, 77% yield, 90% ee

9a, R = 4-BrC_6H_4, 71% yield, 90% ee
9b, R = 4-$NO_2C_6H_4$, 67% yield, 90% ee

The reaction was performed according to a literature procedure.[10] To the mixture of **7** (79.4 mg, 0.2 mmol), 4-bromobenzoyl chloride (87.8 mg, 0.4 mmol) or 4-nitrobenzoyl chloride (74.2 mg, 0.4 mmol) and DMAP (5.0 mg, 0.04 mmol) was added newly distilled dichloromethane (2 mL). In an ice-water bath, trimethylamine (56 μl, 0.4 mmol) was added to the reaction mixture. Then, the reaction mixture was stirred at room temperature for 12 h and was monitored by TLC, which indicated the incompletion of the reaction. After the completion of the reaction indicated by TLC, the reaction mixture was quenched by the saturated aqueous solution of $NaHCO_3$, which was extracted by dichloromethane. The organic layer was washed with brine and combined together, which was dried by anhydrous sodium sulfate. Finally, the organic layer was evaporated under the reduced pressure to give a residue, which was further purified through preparative thin layer chromatography on silica gel to give pure product **9a** in 71% yield, 90% ee or **9b** in 67% yield, 90% ee.

(4-Bromophenyl)((2*S*,5*R*)-2-((dimethyl(phenyl)silyl)methyl)-4,4-dimethyl-2,5-diphenylpyrrolidin-1-yl)methanone (9a)

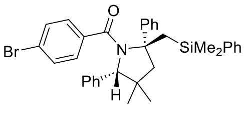

As white solid, $[\alpha]_D^{20}$ = +6.2 (c = 1.0, $CHCl_3$). **^{1}H NMR** (400 MHz, $CDCl_3$) δ 7.72 (d, *J* = 7.9 Hz, 2H), 7.52 – 7.29 (m, 2H), 7.38 – 7.29 (m, 5H), 7.23 (t, *J* = 7.3 Hz, 1H), 7.18 (d, *J* = 8.3 Hz, 2H), 7.04 – 6.96 (m, 3H),

6.80 (d, *J* = 8.3 Hz, 2H), 6.65 (d, *J* = 7.2 Hz, 2H), 4.52 (s, 1H), 2.97 (dd, *J* = 14.7, 2.2 Hz, 1H), 2.66 (d, *J* = 13.2 Hz, 1H), 2.24 (dd, *J* = 13.3, 2.1 Hz, 1H), 1.87 (dd, *J* = 14.7, 2.6 Hz, 1H), 1.06 (s, 3H), 0.26 – 0.13 (m, 9H); **^{13}C NMR** (100 MHz, $CDCl_3$) δ 171.2, 146.6, 139.9, 139.7, 138.3, 133.7, 130.7, 128.7, 127.9, 127.7, 127.7, 127.5, 127.3, 126.7, 76.9, 73.6, 54.0, 40.6, 32.5, 29.4, 25.3, -1.0, -1.3. HRMS (ESI) m/z calcd. for $C_{34}H_{36}BrNNaOSi$ $[M+Na]^+$ = 604.1642, found = 604.1636.

HPLC conditions: Daicel Chiralpak IBN-5 column; hexane/2-propanol = 95/5, 1.0 mL/min, 25 °C. Retention times: 6.19 min (major), 5.30 min (minor).

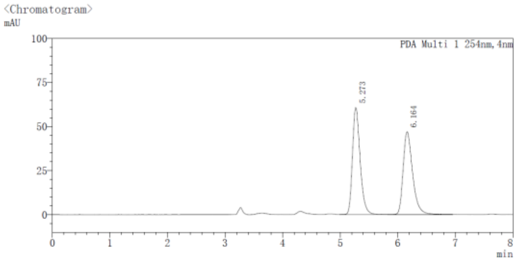

<Peak Table>
PDA Ch1 254nm

No.	Ret.Time(min)	Height(mAU)	Height%	Area(mAU*min)	Area%
1	5.273	60691	56.363	562918	51.323
2	6.164	46987	43.637	533893	48.677
Total		107679	100.000	1096811	100.000

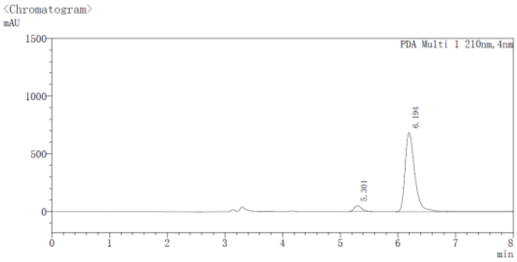

<Peak Table>
PDA Ch1 210nm

No.	Ret.Time(min)	Height(mAU)	Height%	Area(mAU*min)	Area%
1	5.301	48825	6.641	423786	5.042
2	6.194	686418	93.359	7981196	94.958
Total		735242	100.000	8404982	100.000

((2*S*,5*R*)-2-((Dimethyl(phenyl)silyl)methyl)-4,4-dimethyl-2,5-diphenylpyrrolidin-1-yl)(4-nitrophenyl)methanone (9b)

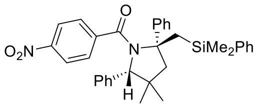

As white solid, $[\alpha]_D^{20}$ = +12.9 (c = 1.0, $CHCl_3$). **^{1}H NMR** (400 MHz, $CDCl_3$) δ 7.91 (d, *J* = 8.8 Hz, 2H), 7.74 (d, *J* = 7.3 Hz, 2H), 7.57 – 7.51 (m, 2H), 7.43 – 7.25 (m, 7H), 7.09 – 7.01 (m, 3H), 6.97 (d, *J* = 6.7 Hz, 1H), 6.65 (s, 2H), 4.49 (s, 1H), 2.98 (d, *J* = 14.7 Hz, 1H), 2.73 (d, *J* = 13.2 Hz, 1H), 2.29 (d, *J* = 13.3 Hz, 1H), 1.93 (d, *J* = 14.6 Hz, 1H), 1.09 (s, 3H), 0.27 – 0.19 (m, 9H); **^{13}C NMR** (100 MHz, $CDCl_3$) δ 170.0, 146.9, 146.2, 145.2, 139.6, 139.2, 133.8, 128.9, 128.1, 127.9, 127.8, 127.7, 127.2, 127.0, 126.9, 126.8, 122.9, 76.8, 74.1, 53.9, 40.7, 32.7, 29.4, 25.2, -1.1, -1.3. HRMS (ESI) m/z calcd. for $C_{34}H_{36}N_2NaO_3Si$ $[M+Na]^+$ = 571.2387, found = 571.2377.

HPLC conditions: Daicel Chiralpak ODH column; hexane/2-propanol = 95/5, 1.0 mL/min, 25 °C. Retention times: 7.75 min (major), 8.89 min (minor).

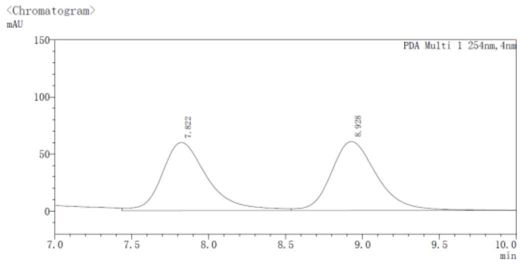

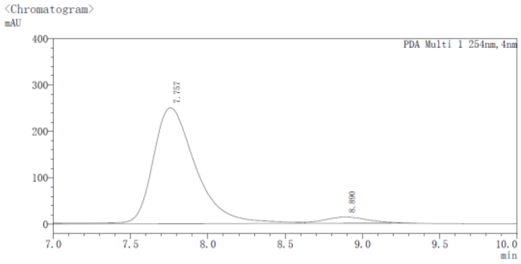

<Peak Table>
PDA Ch1 254nm

No.	Ret.Time(min)	Height(mAU)	Height%	Area(mAU*min)	Area%
1	7.822	59684	49.673	1222963	49.277
2	8.928	60470	50.327	1258834	50.723
Total		120154	100.000	2481797	100.000

<Peak Table>
PDA Ch1 254nm

No.	Ret.Time(min)	Height(mAU)	Height%	Area(mAU*min)	Area%
1	7.757	250156	94.868	4827560	94.751
2	8.890	13534	5.132	267451	5.249
Total		263690	100.000	5095011	100.000

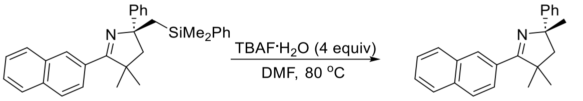

3p **10**, 87% yield, 90% ee

The reaction was performed according to a literature procedure.[6] To a solution of **3p** (89.4 mg, 0.2 mmol) in anhydrous DMF (2.0 mL) was added solid TBAF·H_2O (1.12 g, 4 mmol), and the reaction mixture was heated at 80 °C for 16 h. After the addition of sat. aq NH_4Cl, the reaction mixture was processed with EtOAc. The resulting crude material was purified by silica gel chromatography to afford **10** in 87% yield, 90% ee.

(*S*)-2,4,4-Trimethyl-5-(naphthalen-2-yl)-2-phenyl-3,4-dihydro-2*H*-pyrrole (10)

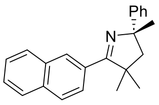

As colorless oil, $[\alpha]_D^{20}$ = +153.1 (c = 1.0, $CHCl_3$). **^{1}H NMR** (400 MHz, $CDCl_3$) δ 8.27 (s, 1H), 8.03 (dd, *J* = 8.5, 1.7 Hz, 1H), 7.94 – 7.84 (m, 3H), 7.56 – 7.49 (m, 4H), 7.39 – 7.32 (m, 2H), 7.25 – 7.18 (m, 1H), 2.41 (d, *J* = 12.8 Hz, 1H), 2.30 (d, *J* = 12.8 Hz, 1H), 1.74 (s, 3H), 1.57 (s, 3H), 1.27 (s, 3H); **^{13}C NMR** (100 MHz, $CDCl_3$) δ 177.5, 150.3, 133.7, 132.9, 132.3, 128.6, 128.2, 127.8, 127.7, 127.6, 126.7, 126.2, 126.0, 126.0, 125.3, 74.0, 56.2, 51.5, 32.4, 28.5, 27.8. HRMS (ESI) m/z calcd. for $C_{23}H_{24}N$ $[M+H]^+$ = 314.1903, found = 314.1896.

HPLC conditions: Daicel Chiralpak IG column; hexane/2-propanol = 99.7/0.3, 0.7 mL/min, 25 °C. Retention times: 10.25 min (major), 8.39 min (minor).

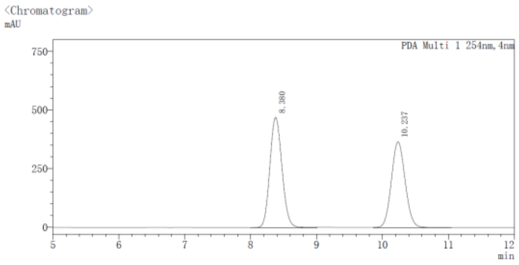

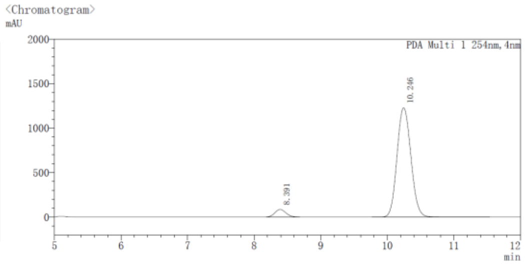

<Peak Table>
PDA Ch1 254nm

No.	Ret.Time(min)	Height(mAU)	Height%	Area(mAU*min)	Area%
1	8.380	470286	56.235	6002331	53.131
2	10.237	365999	43.765	5294792	46.869
Total		836285	100.000	11297123	100.000

<Peak Table>
PDA Ch1 254nm

No.	Ret.Time(min)	Height(mAU)	Height%	Area(mAU*min)	Area%
1	8.391	85176	6.486	968882	5.167
2	10.246	1227996	93.514	17783085	94.833
Total		1313172	100.000	18751966	100.000

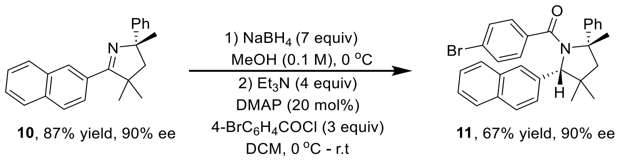

10, 87% yield, 90% ee **11**, 67% yield, 90% ee

The reaction was performed according to a literature procedure.[8,10] Prepare a solution of **10** (0.17 mmol, 54.5 mg) in MeOH (1.7 mL), add $NaBH_4$ (45.0 mg,1.19 mmol) and stir the mixture for 2 hours. After completion of the reaction, water was added. The organic phase was extracted with ethyl acetate. The combined organic layers were washed with water and brine, and dried over anhydrous Na_2SO_4. The resulting solution was concentrated and to be used directly in the next step. To the mixture of 4-bromobenzoyl chloride (111.9 mg, 0.51 mmol) and DMAP (4.15 mg, 0.034 mmol) was added newly distilled dichloromethane (1.7 mL). In an ice-water bath, trimethylamine (94.5 μl, 0.68 mmol) was added to the reaction mixture. Then, the reaction mixture was stirred at room temperature for 12 h and was monitored by TLC, which indicated the incompletion of the reaction. After the completion of the reaction indicated by TLC, the reaction mixture was quenched by the saturated aqueous solution of $NaHCO_3$, which was extracted by dichloromethane. The organic layer was washed with brine and combined together, which was dried by anhydrous sodium sulfate. Finally, the organic layer was evaporated under the reduced pressure to give a residue, which was further purified through preparative thin layer chromatography on silica gel to give pure product **11** in 67% yield, 90% ee.

(4-Bromophenyl)((2*S*,5*R*)-2,4,4-trimethyl-5-(naphthalen-2-yl)-2-phenylpyrrolidin-1-yl)methanone (11)

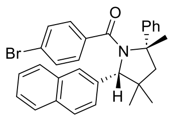

As White solid, $[\alpha]_D^{20}$ = +37.1 (c = 0.5, $CHCl_3$). **^{1}H NMR** (400 MHz, $CDCl_3$) δ 7.79 – 7.71 (m, 1H), 7.65 – 7.55 (m, 3H), 7.52 – 7.47 (m, 1H), 7.45 – 7.40 (m, 4H), 7.37 – 7.29 (m, 1H), 7.20 – 7.12 (m, 3H), 6.97 (s, 1H), 6.87 (d, J = 8.1 Hz, 2H), 4.66 (s, 1H), 2.68 (d, J = 13.1 Hz, 1H), 2.39 (d, J = 13.1 Hz, 1H), 2.17 (s, 3H), 1.29 (s, 3H), 0.44 (s, 3H); **^{13}C NMR** (100 MHz, $CDCl_3$) δ 171.4, 147.5, 137.9, 137.6, 132.8, 132.3, 130.9, 128.0, 127.8, 127.5, 127.4, 127.2, 126.3, 126.3, 126.1, 126.0, 125.9, 122.6, 77.7, 69.8, 56.1, 41.0, 30.8, 29.8, 25.3. HRMS (ESI) m/z calcd. for $C_{30}H_{28}BrNNaO$ $[M+Na]^+$ = 520.1246, found = 520.1244.

HPLC conditions: Daicel Chiralpak IBN-5 column; hexane/2-propanol = 90/10, 1.0 mL/min, 25 °C. Retention times: 8.19 min (major), 10.03 min (minor).

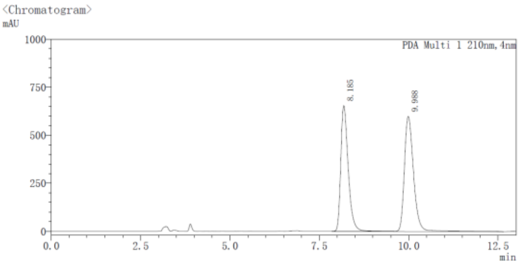

<Peak Table>
PDA Ch1 210nm

No.	Ret.Time(min)	Height(mAU)	Height%	Area(mAU*min)	Area%
1	8.185	656618	52.236	9623760	46.882
2	9.988	600411	47.764	10903807	53.118
Total		1257029	100.000	20527567	100.000

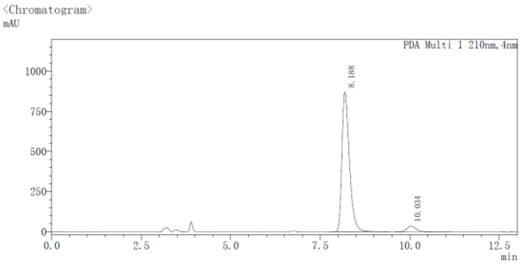

<Peak Table>
PDA Ch1 210nm

No.	Ret.Time(min)	Height(mAU)	Height%	Area(mAU*min)	Area%
1	8.188	870875	96.054	12607227	94.985
2	10.034	35774	3.946	665575	5.015
Total		906649	100.000	13272802	100.000

6. Determination of the absolute configurations

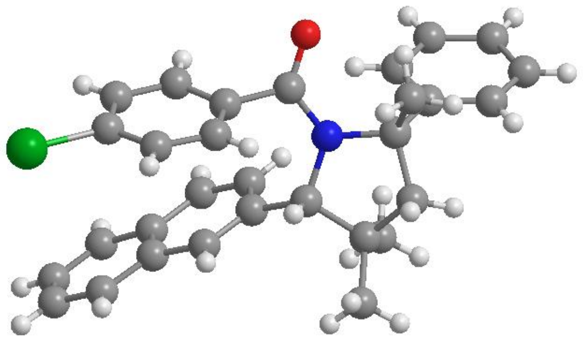

Figure S2. Absolute configuration of **11** (CCDC 2312890)

(Solvent: Chloroform/*n*-Hexane)

Table S9. Crystal data and structure refinement for mo_231207b_0m_sx.

Identification code	mo_231207b_0m_sx
Empirical formula	$C_{33}H_{35}BrNO$
Formula weight	541.53
Temperature/K	273.15
Crystal system	triclinic
Space group	P1
a/Å	10.6388(14)
b/Å	11.2744(13)
c/Å	13.1949(19)
α/°	66.452(6)
β/°	78.039(6)
γ/°	87.919(5)
Volume/Å3	1417.5(3)
Z	2
$\rho_{calc}g/cm3$	1.269
μ/mm^{-1}	1.475
F(000)	566.0
Crystal size/mm^3	0.12 × 0.11 × 0.09
Radiation	MoKα (λ = 0.71073)
2Θ range for data collection/°	3.918 to 52.78
Index ranges	-13 ≤ h ≤ 13, -13 ≤ k ≤ 14, -16 ≤ l ≤ 16

Reflections collected	30395
Independent reflections	11475 [R_{int} = 0.1059, R_{sigma} = 0.1461]
Data/restraints/parameters	11475/3/658
Goodness-of-fit on F^2	0.925
Final R indexes [I>=2σ (I)]	R_1 = 0.0561, wR_2 = 0.1123
Final R indexes [all data]	R_1 = 0.1167, wR_2 = 0.1378
Largest diff. peak/hole / e Å^{-3}	0.40/-0.28
Flack parameter	0.040(10)

Table S10. Fractional Atomic Coordinates (×10^4) and Equivalent Isotropic Displacement Parameters (Å^2×10^3) for mo_231207b_0m_sx. Ueq is defined as 1/3 of the trace of the orthogonalised U_{IJ} tensor.

Atom	*x*	*y*	*z*	U(eq)
Br01	8205.6(7)	26.9(7)	5637.1(8)	68.3(4)
Br02	2788.1(8)	9673.9(8)	7581.1(9)	76.1(4)
O003	11028(5)	4372(5)	7068(5)	44.3(14)
O004	6238(5)	5340(5)	5853(5)	52.0(15)
N005	4554(6)	5453(6)	5000(6)	40.9(16)
N006	8918(6)	4385(5)	7917(6)	36.0(15)
C007	8369(8)	2500(8)	5697(8)	40(2)
C008	8722(7)	3384(7)	6100(7)	40.7(19)
C009	8807(7)	1264(7)	6095(8)	42(2)
C00A	6630(7)	1667(7)	8668(7)	39.8(19)
C00B	2656(7)	8311(7)	4302(7)	38.4(18)
C00C	3862(9)	10288(8)	2891(8)	44(2)
C00D	5220(7)	5798(7)	5629(7)	40.5(19)
C00E	3584(8)	7581(8)	4007(8)	40(2)
C00F	3476(7)	6436(8)	6860(7)	43(2)
C00G	9895(7)	3984(7)	7290(7)	38.1(19)
C00H	9751(7)	5628(7)	8891(8)	43(2)
C00I	10534(7)	4664(8)	9357(7)	47(2)
C00J	7476(7)	2368(8)	8897(7)	35(2)
C00K	7575(8)	3821(8)	8284(8)	38(2)
C00L	2782(7)	9689(7)	3760(8)	40.0(19)
C00M	7564(8)	5907(8)	8307(8)	42(2)
C00N	9508(7)	3022(7)	6863(7)	39.5(19)
C00O	3392(8)	6107(8)	4612(8)	39(2)

C00P	3934(11)	11665(9)	2304(9)	62(3)
C00Q	6497(7)	296(7)	9207(8)	42(2)
C00R	5990(9)	3003(8)	3810(8)	55(2)
C00S	6858(8)	5014(8)	3600(8)	48(2)
C00T	4621(7)	6771(7)	6066(7)	40.1(19)
C00U	2944(8)	7285(8)	7341(8)	42(2)
C00V	3764(9)	5906(9)	2687(8)	48(2)
C00W	5858(8)	4082(8)	4085(7)	43(2)
C00X	5441(8)	4621(9)	8956(9)	56(2)
C00Y	9017(7)	5672(7)	7995(7)	39.3(19)
C00Z	3564(7)	8472(8)	6991(8)	48(2)
C010	3016(7)	5396(7)	3907(7)	42(2)
C011	6881(7)	4585(7)	8984(7)	39.5(19)
C012	5652(8)	-426(9)	8961(9)	61(3)
C013	4686(8)	8188(8)	3166(8)	51(2)
C014	9690(8)	6693(8)	9220(8)	53(2)
C015	4821(8)	9507(8)	2610(8)	51(2)
C016	11229(9)	4681(10)	10130(9)	62(3)
C017	4652(8)	4158(7)	4929(8)	46(2)
C018	9998(7)	1801(8)	7193(8)	49(2)
C019	7952(9)	4863(10)	2879(9)	61(2)
C01A	10386(9)	6716(10)	9992(9)	64(3)
C01B	7053(10)	2834(10)	3104(9)	65(3)
C01C	8108(8)	378(8)	10230(8)	54(2)
C01D	9653(8)	6718(7)	6847(7)	47(2)
C01E	3376(9)	4013(8)	4616(9)	47(2)
C01F	1834(8)	10484(8)	4055(9)	54(2)
C01G	5262(7)	7940(8)	5784(8)	51(2)
C01H	8073(10)	3788(11)	2623(9)	71(3)
C01I	9618(7)	904(8)	6826(8)	48(2)
C01J	1586(8)	5447(9)	3935(9)	60(3)
C01K	8226(8)	1694(8)	9694(8)	46(2)
C01L	4707(8)	8816(8)	6227(9)	57(3)
C01M	7008(9)	4034(9)	10213(8)	52(2)
C01N	1957(10)	11799(9)	3495(10)	67(3)
C01O	5520(10)	-1742(10)	9497(11)	74(3)

C01P	4681(9)	3097(8)	6091(8)	57(2)
C01Q	11140(10)	5715(12)	10448(10)	74(3)
C01R	12812(11)	1716(10)	9420(11)	77(3)
C01S	6242(11)	-2403(10)	10289(10)	71(3)
C01T	2998(11)	12388(9)	2614(10)	71(3)
C01U	7242(9)	-370(9)	10014(9)	50(2)
C01V	11230(11)	522(12)	11201(11)	80(3)
C01W	7088(10)	-1749(9)	10551(9)	58(3)
C01X	10593(11)	-796(12)	12032(11)	84(4)
C01Y	12296(13)	423(13)	10296(12)	96(4)
C01Z	9475(13)	-731(17)	12920(13)	113(5)
C020	8745(13)	-2043(15)	13642(15)	118(5)

Table S11. Anisotropic Displacement Parameters (Å^2×10^3) for mo_231207b_0m_sx. The Anisotropic displacement factor exponent takes the form: -2π^2[h^{2}a^{*2}U$_{11}$+2hka*b*U12+…].

Atom	U_{11}	U_{22}	U_{33}	U_{23}	U_{13}	U_{12}
Br01	54.3(7)	63.8(6)	110.8(11)	-53.4(7)	-31.5(7)	9.5(5)
Br02	46.3(6)	81.2(8)	129.8(12)	-74.3(8)	-15.3(7)	10.0(5)
O003	29(3)	54(3)	50(4)	-25(3)	1(3)	-2(2)
O004	39(3)	71(4)	53(4)	-31(3)	-12(3)	15(3)
N005	40(4)	42(4)	40(5)	-16(3)	-10(3)	4(3)
N006	35(3)	33(3)	40(4)	-15(3)	-6(3)	0(3)
C007	34(5)	44(5)	42(6)	-19(4)	-9(4)	8(4)
C008	39(5)	39(4)	38(6)	-14(4)	-1(4)	4(3)
C009	27(4)	49(5)	56(6)	-29(5)	-4(4)	3(3)
C00A	32(4)	49(5)	38(5)	-19(4)	-5(4)	4(3)
C00B	23(4)	46(4)	40(6)	-13(4)	0(4)	-5(3)
C00C	45(5)	46(5)	40(6)	-14(5)	-11(5)	1(4)
C00D	33(4)	50(5)	32(5)	-12(4)	-3(4)	2(4)
C00E	35(5)	42(5)	41(6)	-12(4)	-10(4)	-7(4)
C00F	35(5)	47(5)	44(6)	-17(4)	-6(4)	-1(4)
C00G	36(5)	40(4)	36(5)	-11(4)	-11(4)	6(3)
C00H	34(4)	47(5)	44(6)	-19(4)	2(4)	-6(4)
C00I	39(5)	57(5)	43(6)	-21(5)	-4(4)	-5(4)
C00J	24(4)	41(4)	37(5)	-14(4)	-5(4)	4(3)
C00K	30(4)	45(5)	36(6)	-12(4)	-8(4)	-3(4)
C00L	32(4)	46(5)	45(6)	-20(4)	-12(4)	1(3)

C00M	40(5)	41(5)	41(6)	-15(4)	-7(4)	7(4)
C00N	29(4)	41(4)	43(6)	-15(4)	1(4)	2(3)
C00O	26(4)	49(5)	38(6)	-16(4)	-3(4)	5(4)
C00P	69(7)	49(6)	58(8)	-12(5)	-12(6)	-17(5)
C00Q	30(4)	47(5)	49(6)	-25(4)	5(4)	-3(3)
C00R	63(6)	59(5)	47(7)	-23(5)	-21(5)	14(4)
C00S	46(5)	50(5)	40(6)	-12(4)	-5(4)	14(4)
C00T	33(4)	51(5)	40(6)	-21(4)	-11(4)	4(4)
C00U	32(5)	54(5)	43(6)	-21(5)	-10(4)	1(4)
C00V	54(6)	52(5)	36(6)	-16(5)	-5(5)	-2(4)
C00W	50(5)	50(5)	26(5)	-14(4)	-9(4)	9(4)
C00X	36(5)	72(6)	61(7)	-27(5)	-11(5)	11(4)
C00Y	30(4)	39(4)	44(6)	-16(4)	2(4)	-1(3)
C00Z	33(4)	59(5)	63(7)	-35(5)	-15(5)	12(4)
C010	35(4)	44(4)	41(6)	-12(4)	-4(4)	-4(3)
C011	28(4)	54(5)	30(5)	-14(4)	1(4)	3(3)
C012	52(6)	57(6)	78(8)	-35(6)	-2(5)	0(4)
C013	36(5)	55(5)	46(6)	-10(5)	3(4)	-1(4)
C014	43(5)	55(5)	61(7)	-32(5)	7(5)	-4(4)
C015	38(5)	55(5)	48(6)	-14(5)	9(4)	-18(4)
C016	55(6)	81(7)	54(7)	-29(6)	-11(5)	-1(5)
C017	48(5)	47(5)	40(6)	-16(4)	-10(4)	5(4)
C018	36(4)	49(5)	69(7)	-25(5)	-22(5)	10(4)
C019	50(6)	77(6)	45(7)	-17(5)	-6(5)	16(5)
C01A	50(6)	89(7)	66(8)	-51(6)	5(5)	-16(5)
C01B	75(7)	80(7)	60(8)	-47(6)	-26(6)	31(6)
C01C	50(5)	52(5)	46(6)	-6(5)	-13(5)	6(4)
C01D	52(5)	37(4)	36(6)	-3(4)	2(4)	-3(4)
C01E	47(5)	47(5)	40(6)	-12(5)	-5(4)	-2(4)
C01F	52(5)	54(5)	61(7)	-27(5)	-17(5)	5(4)
C01G	26(4)	63(5)	56(7)	-21(5)	3(4)	-4(4)
C01H	59(6)	112(9)	40(7)	-35(7)	-7(5)	34(6)
C01I	40(5)	43(4)	68(7)	-27(5)	-20(5)	10(4)
C01J	44(5)	65(6)	65(7)	-20(5)	-13(5)	-8(4)
C01K	41(5)	52(5)	41(6)	-13(4)	-13(4)	3(4)
C01L	34(5)	54(5)	85(8)	-34(5)	-4(5)	-6(4)

C01M	49(5)	62(6)	43(6)	-21(5)	-5(5)	-4(4)
C01N	76(7)	48(5)	92(9)	-35(6)	-34(7)	7(5)
C01O	67(7)	68(7)	93(10)	-49(7)	7(7)	-11(5)
C01P	70(6)	51(5)	43(6)	-12(5)	-8(5)	0(4)
C01Q	60(7)	113(9)	59(8)	-46(7)	-9(6)	-10(6)
C01R	71(8)	67(7)	81(10)	-27(7)	9(7)	-5(6)
C01S	81(7)	49(5)	71(8)	-33(6)	30(6)	-16(5)
C01T	92(8)	38(5)	88(9)	-18(6)	-39(8)	-6(5)
C01U	42(6)	54(6)	47(7)	-21(5)	9(5)	-5(4)
C01V	58(7)	106(9)	80(10)	-34(8)	-27(7)	6(6)
C01W	72(7)	43(5)	41(7)	-11(5)	14(5)	9(5)
C01X	65(7)	110(9)	74(10)	-27(8)	-29(7)	18(7)
C01Y	84(9)	118(11)	94(11)	-59(9)	-4(8)	19(8)
C01Z	74(9)	191(15)	94(12)	-84(12)	-2(8)	-18(10)
C020	71(9)	149(14)	108(13)	-37(11)	4(9)	10(9)

Table S12. Bond Lengths for mo_231207b_0m_sx.

Atom	Atom	Length/Å	Atom	Atom	Length/Å
Br01	C009	1.903(7)	C00Q	C012	1.401(11)
Br02	C00Z	1.903(7)	C00Q	C01U	1.411(12)
O003	C00G	1.232(8)	C00R	C00W	1.397(10)
O004	C00D	1.223(8)	C00R	C01B	1.368(13)
N005	C00D	1.365(9)	C00S	C00W	1.381(11)
N005	C00O	1.493(10)	C00S	C019	1.395(12)
N005	C017	1.498(9)	C00T	C01G	1.381(10)
N006	C00G	1.371(10)	C00U	C00Z	1.373(11)
N006	C00K	1.487(10)	C00V	C010	1.522(12)
N006	C00Y	1.504(9)	C00W	C017	1.539(12)
C007	C008	1.397(11)	C00X	C011	1.538(10)
C007	C009	1.379(11)	C00Y	C01D	1.533(11)
C008	C00N	1.369(11)	C00Z	C01L	1.363(12)
C009	C01I	1.359(11)	C010	C01E	1.547(12)
C00A	C00J	1.372(10)	C010	C01J	1.514(11)
C00A	C00Q	1.418(11)	C011	C01M	1.521(12)
C00B	C00E	1.356(11)	C012	C01O	1.362(13)
C00B	C00L	1.425(10)	C013	C015	1.368(11)
C00C	C00L	1.408(12)	C014	C01A	1.386(12)

C00C	C00P	1.428(13)	C016	C01Q	1.383(13)
C00C	C015	1.411(12)	C017	C01E	1.531(11)
C00D	C00T	1.499(10)	C017	C01P	1.528(12)
C00E	C00O	1.529(11)	C018	C01I	1.387(10)
C00E	C013	1.410(12)	C019	C01H	1.378(13)
C00F	C00T	1.382(11)	C01A	C01Q	1.367(14)
C00F	C00U	1.392(11)	C01B	C01H	1.411(14)
C00G	C00N	1.510(10)	C01C	C01K	1.362(11)
C00H	C00I	1.366(11)	C01C	C01U	1.414(12)
C00H	C00Y	1.531(11)	C01F	C01N	1.364(12)
C00H	C014	1.426(10)	C01G	C01L	1.394(11)
C00I	C016	1.384(12)	C01N	C01T	1.386(15)
C00J	C00K	1.506(11)	C01O	C01S	1.380(15)
C00J	C01K	1.410(11)	C01R	C01Y	1.488(17)
C00K	C011	1.564(11)	C01S	C01W	1.362(14)
C00L	C01F	1.422(11)	C01U	C01W	1.427(13)
C00M	C00Y	1.556(11)	C01V	C01X	1.527(16)
C00M	C011	1.515(11)	C01V	C01Y	1.504(17)
C00N	C018	1.385(10)	C01X	C01Z	1.509(17)
C00O	C010	1.563(11)	C01Z	C020	1.532(19)
C00P	C01T	1.366(15)			

Table S13. Bond Angles for mo_231207b_0m_sx.

Atom	Atom	Atom	Angle/°	Atom	Atom	Atom	Angle/°
C00D	N005	C00O	123.8(6)	C00S	C00W	C017	123.4(7)
C00D	N005	C017	122.6(6)	N006	C00Y	C00H	113.0(6)
C00O	N005	C017	111.1(6)	N006	C00Y	C00M	100.1(5)
C00G	N006	C00K	123.6(6)	N006	C00Y	C01D	110.8(7)
C00G	N006	C00Y	121.4(6)	C00H	C00Y	C00M	112.7(7)
C00K	N006	C00Y	112.8(6)	C00H	C00Y	C01D	109.4(6)
C009	C007	C008	118.9(8)	C01D	C00Y	C00M	110.6(7)
C00N	C008	C007	119.8(7)	C00U	C00Z	Br02	118.7(7)
C007	C009	Br01	118.5(6)	C01L	C00Z	Br02	119.4(6)
C01I	C009	Br01	119.6(6)	C01L	C00Z	C00U	122.0(7)
C01I	C009	C007	121.9(7)	C00V	C010	C00O	114.1(6)
C00J	C00A	C00Q	121.9(7)	C00V	C010	C01E	112.2(7)
C00E	C00B	C00L	120.4(8)	C01E	C010	C00O	98.8(6)

C00L	C00C	C00P	118.8(9)
C00L	C00C	C015	119.0(8)
C015	C00C	C00P	122.2(9)
O004	C00D	N005	123.4(7)
O004	C00D	C00T	120.5(7)
N005	C00D	C00T	116.1(6)
C00B	C00E	C00O	118.1(8)
C00B	C00E	C013	119.9(8)
C013	C00E	C00O	122.0(8)
C00T	C00F	C00U	120.6(7)
O003	C00G	N006	123.5(6)
O003	C00G	C00N	120.6(7)
N006	C00G	C00N	115.9(6)
C00I	C00H	C00Y	124.3(7)
C00I	C00H	C014	116.8(7)
C014	C00H	C00Y	118.8(7)
C00H	C00I	C016	122.9(8)
C00A	C00J	C00K	119.0(7)
C00A	C00J	C01K	118.4(7)
C01K	C00J	C00K	122.6(7)
N006	C00K	C00J	113.7(6)
N006	C00K	C011	102.7(6)
C00J	C00K	C011	115.5(7)
C00C	C00L	C00B	119.4(7)
C00C	C00L	C01F	118.7(8)
C01F	C00L	C00B	121.9(8)
C011	C00M	C00Y	106.6(6)
C008	C00N	C00G	119.7(7)
C008	C00N	C018	119.9(7)
C018	C00N	C00G	120.3(7)
N005	C00O	C00E	113.8(6)
N005	C00O	C010	104.2(6)
C00E	C00O	C010	115.7(7)
C01T	C00P	C00C	120.4(10)
C012	C00Q	C00A	122.1(8)
C012	C00Q	C01U	118.6(8)
C01J	C010	C00O	110.5(7)
C01J	C010	C00V	109.8(7)
C01J	C010	C01E	111.1(7)
C00M	C011	C00K	100.3(6)
C00M	C011	C00X	111.7(7)
C00M	C011	C01M	112.4(7)
C00X	C011	C00K	109.4(6)
C01M	C011	C00K	114.6(7)
C01M	C011	C00X	108.3(7)
C01O	C012	C00Q	121.5(10)
C015	C013	C00E	121.1(8)
C01A	C014	C00H	120.6(9)
C013	C015	C00C	120.1(8)
C01Q	C016	C00I	119.2(10)
N005	C017	C00W	112.9(7)
N005	C017	C01E	101.9(6)
N005	C017	C01P	110.0(7)
C01E	C017	C00W	114.6(7)
C01P	C017	C00W	108.4(7)
C01P	C017	C01E	108.9(7)
C00N	C018	C01I	120.4(7)
C01H	C019	C00S	121.5(9)
C01Q	C01A	C014	120.3(9)
C00R	C01B	C01H	119.2(8)
C01K	C01C	C01U	122.1(8)
C017	C01E	C010	106.6(6)
C01N	C01F	C00L	120.7(10)
C00T	C01G	C01L	120.1(8)
C019	C01H	C01B	118.3(9)
C009	C01I	C018	118.9(7)
C01C	C01K	C00J	120.8(7)
C00Z	C01L	C01G	119.2(7)
C01F	C01N	C01T	120.8(9)
C012	C01O	C01S	120.4(9)
C01A	C01Q	C016	120.2(9)
C01W	C01S	C01O	120.5(9)

C01U	C00Q	C00A	119.3(7)	C00P	C01T	C01N	120.6(9)
C01B	C00R	C00W	123.2(9)	C00Q	C01U	C01C	117.5(8)
C00W	C00S	C019	120.8(8)	C00Q	C01U	C01W	118.4(9)
C00F	C00T	C00D	119.3(7)	C01C	C01U	C01W	124.1(10)
C01G	C00T	C00D	120.9(7)	C01Y	C01V	C01X	112.7(10)
C01G	C00T	C00F	119.4(7)	C01S	C01W	C01U	120.6(10)
C00Z	C00U	C00F	118.5(8)	C01Z	C01X	C01V	114.2(11)
C00R	C00W	C017	119.4(7)	C01R	C01Y	C01V	112.2(10)
C00S	C00W	C00R	117.1(8)	C01X	C01Z	C020	113.1(12)

Table S14. Torsion Angles for mo_231207b_0m_sx.

A	B	C	D	Angle/°	A	B	C	D	Angle/°
Br01	C009	C01I	C018	-177.0(7)	C00N	C018	C01I	C009	3.2(13)
Br02	C00Z	C01L	C01G	-179.0(7)	C00O	N005	C00D	O004	-172.9(8)
O003	C00G	C00N	C008	-114.7(9)	C00O	N005	C00D	C00T	8.3(11)
O003	C00G	C00N	C018	61.8(11)	C00O	N005	C017	C00W	116.5(7)
O004	C00D	C00T	C00F	-112.5(9)	C00O	N005	C017	C01E	-6.9(9)
O004	C00D	C00T	C01G	60.4(12)	C00O	N005	C017	C01P	-122.3(8)
N005	C00D	C00T	C00F	66.3(10)	C00O	C00E	C013	C015	177.9(8)
N005	C00D	C00T	C01G	-120.8(8)	C00O	C010	C01E	C017	-41.6(8)
N005	C00O	C010	C00V	-83.4(8)	C00P	C00C	C00L	C00B	175.8(7)
N005	C00O	C010	C01E	35.8(8)	C00P	C00C	C00L	C01F	-2.9(12)
N005	C00O	C010	C01J	152.3(7)	C00P	C00C	C015	C013	-177.4(8)
N005	C017	C01E	C010	30.9(9)	C00Q	C00A	C00J	C00K	179.4(8)
N006	C00G	C00N	C008	64.8(10)	C00Q	C00A	C00J	C01K	-0.1(12)
N006	C00G	C00N	C018	-118.7(8)	C00Q	C012	C01O	C01S	-1.1(15)
N006	C00K	C011	C00M	36.0(7)	C00Q	C01U	C01W	C01S	-0.1(14)
N006	C00K	C011	C00X	153.5(7)	C00R	C00W	C017	N005	-171.2(7)
N006	C00K	C011	C01M	-84.6(8)	C00R	C00W	C017	C01E	-55.1(10)
C007	C008	C00N	C00G	179.6(7)	C00R	C00W	C017	C01P	66.7(9)
C007	C008	C00N	C018	3.0(13)	C00R	C01B	C01H	C019	-0.6(14)
C007	C009	C01I	C018	1.0(13)	C00S	C00W	C017	N005	11.1(11)
C008	C007	C009	Br01	174.9(6)	C00S	C00W	C017	C01E	127.1(8)
C008	C007	C009	C01I	-3.1(13)	C00S	C00W	C017	C01P	-111.0(9)
C008	C00N	C018	C01I	-5.2(13)	C00S	C019	C01H	C01B	0.5(15)
C009	C007	C008	C00N	1.0(13)	C00T	C00F	C00U	C00Z	1.5(13)
C00A	C00J	C00K	N006	-138.8(8)	C00T	C01G	C01L	C00Z	3.9(14)
C00A	C00J	C00K	C011	102.8(9)	C00U	C00F	C00T	C00D	175.4(7)

C00A	C00J	C01K	C01C	-0.4(13)	C00U	C00F	C00T	C01G	2.5(12)
C00A	C00Q	C012	C01O	-179.1(9)	C00U	C00Z	C01L	C01G	0.2(14)
C00A	C00Q	C01U	C01C	-1.1(12)	C00V	C010	C01E	C017	78.9(9)
C00A	C00Q	C01U	C01W	179.7(8)	C00W	C00R	C01B	C01H	0.0(14)
C00B	C00E	C00O	N005	-133.9(8)	C00W	C00S	C019	C01H	0.1(14)
C00B	C00E	C00O	C010	105.5(9)	C00W	C017	C01E	C010	-91.3(9)
C00B	C00E	C013	C015	-2.2(13)	C00Y	N006	C00G	O003	24.9(11)
C00B	C00L	C01F	C01N	-177.6(8)	C00Y	N006	C00G	C00N	-154.6(7)
C00C	C00L	C01F	C01N	1.1(12)	C00Y	N006	C00K	C00J	-144.1(7)
C00C	C00P	C01T	C01N	-0.7(15)	C00Y	N006	C00K	C011	-18.5(8)
C00D	N005	C00O	C00E	51.5(11)	C00Y	C00H	C00I	C016	177.1(8)
C00D	N005	C00O	C010	178.4(7)	C00Y	C00H	C014	C01A	-177.2(8)
C00D	N005	C017	C00W	-80.7(9)	C00Y	C00M	C011	C00K	-42.2(8)
C00D	N005	C017	C01E	155.9(8)	C00Y	C00M	C011	C00X	-158.0(7)
C00D	N005	C017	C01P	40.5(10)	C00Y	C00M	C011	C01M	79.9(8)
C00D	C00T	C01G	C01L	-178.0(8)	C011	C00M	C00Y	N006	30.8(8)
C00E	C00B	C00L	C00C	1.9(11)	C011	C00M	C00Y	C00H	-89.4(7)
C00E	C00B	C00L	C01F	-179.4(8)	C011	C00M	C00Y	C01D	147.7(7)
C00E	C00O	C010	C00V	42.4(10)	C012	C00Q	C01U	C01C	178.7(8)
C00E	C00O	C010	C01E	161.5(7)	C012	C00Q	C01U	C01W	-0.6(13)
C00E	C00O	C010	C01J	-81.9(9)	C012	C01O	C01S	C01W	0.4(16)
C00E	C013	C015	C00C	1.4(13)	C013	C00E	C00O	N005	46.0(11)
C00F	C00T	C01G	C01L	-5.1(13)	C013	C00E	C00O	C010	-74.7(10)
C00F	C00U	C00Z	Br02	176.4(6)	C014	C00H	C00I	C016	1.1(13)
C00F	C00U	C00Z	C01L	-2.8(13)	C014	C00H	C00Y	N006	-167.1(7)
C00G	N006	C00K	C00J	53.0(10)	C014	C00H	C00Y	C00M	-54.6(9)
C00G	N006	C00K	C011	178.5(7)	C014	C00H	C00Y	C01D	68.9(9)
C00G	N006	C00Y	C00H	-83.3(9)	C014	C01A	C01Q	C016	1.0(16)
C00G	N006	C00Y	C00M	156.7(7)	C015	C00C	C00L	C00B	-2.7(12)
C00G	N006	C00Y	C01D	39.9(9)	C015	C00C	C00L	C01F	178.6(8)
C00G	C00N	C018	C01I	178.3(8)	C015	C00C	C00P	C01T	-178.8(9)
C00H	C00I	C016	C01Q	-0.3(14)	C017	N005	C00D	O004	26.4(12)
C00H	C014	C01A	C01Q	-0.1(14)	C017	N005	C00D	C00T	-152.3(7)
C00I	C00H	C00Y	N006	17.0(11)	C017	N005	C00O	C00E	-145.9(7)
C00I	C00H	C00Y	C00M	129.5(8)	C017	N005	C00O	C010	-19.0(8)
C00I	C00H	C00Y	C01D	-106.9(9)	C019	C00S	C00W	C00R	-0.6(12)
C00I	C00H	C014	C01A	-1.0(12)	C019	C00S	C00W	C017	177.1(8)
C00I	C016	C01Q	C01A	-0.9(15)	C01B	C00R	C00W	C00S	0.6(13)

C00J	C00A	C00Q	C012	-178.8(8)	C01B	C00R	C00W	C017	-177.3(8)
C00J	C00A	C00Q	C01U	0.9(12)	C01C	C01U	C01W	C01S	-179.3(9)
C00J	C00K	C011	C00M	160.4(7)	C01F	C01N	C01T	C00P	-1.3(15)
C00J	C00K	C011	C00X	-82.1(9)	C01J	C010	C01E	C017	-157.7(8)
C00J	C00K	C011	C01M	39.7(9)	C01K	C00J	C00K	N006	40.7(11)
C00K	N006	C00G	O003	-173.6(7)	C01K	C00J	C00K	C011	-77.7(10)
C00K	N006	C00G	C00N	6.9(11)	C01K	C01C	C01U	C00Q	0.5(14)
C00K	N006	C00Y	C00H	113.3(7)	C01K	C01C	C01U	C01W	179.7(9)
C00K	N006	C00Y	C00M	-6.7(8)	C01O	C01S	C01W	C01U	0.2(15)
C00K	N006	C00Y	C01D	-123.5(7)	C01P	C017	C01E	C010	147.1(7)
C00K	C00J	C01K	C01C	-179.9(8)	C01U	C00Q	C012	C01O	1.2(13)
C00L	C00B	C00E	C00O	-179.6(7)	C01U	C01C	C01K	C00J	0.2(14)
C00L	C00B	C00E	C013	0.5(12)	C01V	C01X	C01Z	C020	172.4(11)
C00L	C00C	C00P	C01T	2.7(14)	C01X	C01V	C01Y	C01R	173.7(11)
C00L	C00C	C015	C013	1.0(13)	C01Y	C01V	C01X	C01Z	-<br>177.2(10)
C00L	C01F	C01N	C01T	1.0(13)					

Table S15. Hydrogen Atom Coordinates (Å×10^4) and Isotropic Displacement Parameters (Å^2×10^3) for mo_231207b_0m_sx.

Atom	*x*	*y*	*z*	U(eq)
H007	7846.83	2739.55	5169.33	48
H008	8424.84	4217.21	5850.06	49
H00A	6128.93	2102.33	8144.68	48
H00G	1932.25	7908.71	4861.01	46
H00H	3058.37	5636	7073.88	51
H00I	10603.61	3966.11	9146	56
H00K	7210.29	4051.19	7610.12	46
H00B	7439.86	6425.25	8752.24	50
H00C	7232.01	6359.26	7627.48	50
H00O	2700.09	5911.84	5283.67	47
H00P	4623.34	12071.24	1706.12	74
H00R	5323.98	2367.9	4122.41	66
H00S	6801.66	5751.14	3756.96	58
H00U	2183.61	7052.79	7887.17	50
H00J	3545.9	6780.03	2289.42	72
H00L	3547.64	5371.55	2329.24	72
H00M	4669.89	5886.86	2674.4	72

H00D	5021.95	5125.63	9353.94	85
H00E	5061.39	3754.49	9311.79	85
H00F	5342.38	5004.95	8185.63	85
H012	5169.25	2.13	8420.18	73
H013	5332.64	7682.73	2986.87	61
H014	9179.7	7376.97	8915.03	63
H015	5546.05	9889.99	2046.13	62
H016	11749.91	4005.06	10431.36	75
H018	10586.18	1582.26	7663.62	59
H019	8613.93	5502.44	2563.45	73
H01A	10340.59	7414.99	10200.67	77
H01J	7101.59	2100.68	2942.55	78
H01C	8612.93	-41.43	10754.11	64
H01B	9232.02	6703.18	6274.34	71
H01D	10545.26	6552.6	6664.87	71
H01E	9580.6	7552.61	6881.22	71
H01L	2713.29	3603.72	5292.39	56
H01M	3470.25	3485.63	4179.03	56
H01N	1123.14	10102.85	4636.04	65
H01P	6065.84	8142.99	5296.53	61
H01R	8809.26	3692.68	2145.29	85
H01I	9912.3	70.01	7076.61	57
H01T	1119.73	5131.31	4705.7	89
H01U	1353.42	4916.06	3579.99	89
H01V	1378.69	6324.96	3537.01	89
H01K	8808.82	2152.76	9856.66	55
H01X	5110.32	9624.67	6004.65	68
H01F	7901.99	4004.21	10245.6	78
H01G	6612.18	3174.32	10594.4	78
H01H	6589.55	4574.93	10573.48	78
H01Y	1336.09	12305.49	3706.14	81
H01O	4940.89	-2197.02	9327.52	89
H01Z	5508.15	3131.95	6265.14	86
H	4527.26	2265.83	6086.67	86
HA	4025.79	3227.3	6652.82	86
H01Q	11593.62	5730.37	10974.44	88

H1	13461.58	1607.73	8843.03	116
HB	13180.5	2188.86	9761.54	116
HC	12127.23	2186.71	9088.87	116
H01S	6148.9	-3301.68	10645.75	85
H01	3058.9	13284.93	2230.96	86
H2	10584.12	1068.45	10847.33	96
HD	11573.53	933.05	11612.94	96
H01W	7570.15	-2205.75	11084.02	70
H3	10294.08	-1222.83	11610.2	101
HE	11234.94	-1324.46	12406.77	101
H4	12987.16	-40.24	10640.93	115
HF	11976.52	-72.52	9938.46	115
H5	8883.06	-114.96	12550.72	135
HG	9790.99	-416.06	13409.87	135
H02A	8290.3	-2282.93	13189.65	177
H02B	8143.41	-1979.52	14267.68	177
H02C	9344.84	-2688.45	13920.89	177

Experimental

Single crystals of $C_{33}H_{35}BrNO$ [mo_231207b_0m_sx] were []. A suitable crystal was selected and [] on a Bruker APEX-II CCD diffractometer. The crystal was kept at 273.15 K during data collection. Using Olex2 [1], the structure was solved with the olex2.solve [2] structure solution program using Charge Flipping and refined with the SHELXL [3] refinement package using Least Squares minimisation.

[1] Dolomanov, O.V., Bourhis, L.J., Gildea, R.J, Howard, J.A.K. & Puschmann, H. (2009), J. Appl. Cryst. 42, 339-341.

[2] Bourhis, L.J., Dolomanov, O.V., Gildea, R.J., Howard, J.A.K., Puschmann, H. (2015). Acta Cryst. A71, 59-75.

[3] Sheldrick, G.M. (2015). Acta Cryst. C71, 3-8.

Crystal structure determination of [mo_231207b_0m_sx]

Crystal Data for $C_{33}H_{35}BrNO$ (M =541.53 g/mol): triclinic, space group P1 (no. 1), a = 10.6388(14) Å, b = 11.2744(13) Å, c = 13.1949(19) Å, α = 66.452(6)°, β = 78.039(6)°, γ = 87.919(5)°, V = 1417.5(3) Å^3, Z = 2, T = 273.15 K, μ(MoKα) = 1.475 mm-1, Dcalc = 1.269 g/cm3, 30395 reflections

measured (3.918° ≤ 2Θ ≤ 52.78°), 11475 unique (Rint = 0.1059, Rsigma = 0.1461) which were used in all calculations. The final R_1 was 0.0561 (I > 2σ(I)) and wR_2 was 0.1378 (all data).

Refinement model description

Number of restraints - 3, number of constraints - unknown.

Details:

1. Fixed Uiso

At 1.2 times of:

All C(H) groups, All C(H,H) groups

At 1.5 times of:

All C(H,H,H) groups

2.a Ternary CH refined with riding coordinates:

C00K(H00K), C00O(H00O)

2.b Secondary CH2 refined with riding coordinates:

C00M(H00B,H00C), C01E(H01L,H01M), C01V(H2,HD), C01X(H3,HE), C01Y(H4,HF), C01Z(H5,HG)

2.c Aromatic/amide H refined with riding coordinates:

C007(H007), C008(H008), C00A(H00A), C00B(H00G), C00F(H00H), C00I(H00I), C00P(H00P), C00R(H00R), C00S(H00S), C00U(H00U), C012(H012), C013(H013), C014(H014), C015(H015), C016(H016), C018(H018), C019(H019), C01A(H01A), C01B(H01J), C01C(H01C), C01F(H01N), C01G(H01P), C01H(H01R), C01I(H01I), C01K(H01K), C01L(H01X), C01N(H01Y), C01O(H01O), C01Q(H01Q), C01S(H01S), C01T(H01), C01W(H01W)

2.d Idealised Me refined as rotating group:

C00V(H00J,H00L,H00M), C00X(H00D,H00E,H00F), C01D(H01B,H01D,H01E), C01J(H01T, H01U,H01V), C01M(H01F,H01G,H01H), C01P(H01Z,H,HA), C01R(H1,HB,HC), C020(H02A, H02B,H02C)

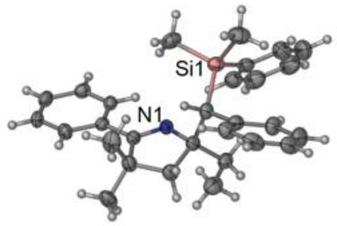

Figure S3. Absolute configuration of **3an** (CCDC 2324487)

(Solvent: Chloroform/*n*-Hexane)

Table S16. Crystal data and structure refinement for exp_3913_auto.

Identification code	exp_3913_auto
Empirical formula	$C_{29}H_{35}NSi$
Formula weight	425.67
Temperature/K	173.00(10)
Crystal system	monoclinic
Space group	$P2_1$
a/Å	15.4543(3)
b/Å	8.27200(10)
c/Å	21.0647(3)
α/°	90
β/°	110.261(2)
γ/°	90
Volume/Å^3	2526.25(7)
Z	4
ρ_{calc}g/cm^3	1.119
μ/mm^{-1}	0.914
F(000)	920.0
Crystal size/mm^3	0.32 × 0.26 × 0.22
Radiation	Cu Kα (λ = 1.54184)
2Θ range for data collection/°	6.096 to 134.152
Index ranges	$-18 \leq h \leq 18, -9 \leq k \leq 9, -25 \leq l \leq 25$
Reflections collected	64256
Independent reflections	8977 [R_{int} = 0.0671, R_{sigma} = 0.0381]
Data/restraints/parameters	8977/1/569
Goodness-of-fit on F^2	1.064

Final R indexes [I>=2σ (I)]	R_1 = 0.0345, wR_2 = 0.0812
Final R indexes [all data]	R_1 = 0.0379, wR_2 = 0.0832
Largest diff. peak/hole / e Å^{-3}	0.12/-0.20
Flack parameter	-0.001(10)

Table S17. Fractional Atomic Coordinates (×10^4) and Equivalent Isotropic Displacement Parameters (Å^2×10^3) for exp_3913_auto. U_{eq} is defined as 1/3 of the trace of the orthogonalised U_{IJ} tensor.

Atom	*x*	*y*	*z*	U(eq)
Si1	2913.2(5)	3344.5(10)	4684.0(4)	37.67(18)
N1	1969.0(14)	5313(3)	3385.9(9)	31.1(5)
C1	2311.2(17)	5309(3)	2360.4(12)	30.4(5)
C2	1939.9(16)	3767(3)	2197.8(11)	30.4(5)
C3	1944.2(18)	2997(3)	1617.7(12)	36.1(6)
C4	2321.7(19)	3755(4)	1184.9(13)	39.7(7)
C5	2694(2)	5276(4)	1339.5(13)	41.0(7)
C6	2695.8(19)	6043(4)	1923.2(13)	38.8(6)
C7	2262.1(17)	6113(3)	2982.5(12)	30.5(5)
C8	2562(2)	7849(3)	3196.9(13)	38.4(6)
C9	2193(2)	8037(3)	3787.0(13)	39.6(6)
C10	1985.4(18)	6313(3)	3970.9(12)	33.4(6)
C11	3624(2)	7946(4)	3444.5(16)	53.7(8)
C12	2130(3)	9135(4)	2661.2(15)	53.4(8)
C13	1039.8(18)	6207(4)	4062.4(12)	38.0(6)
C14	217(2)	6461(4)	3411.0(14)	51.1(8)
C15	2780.1(19)	5635(3)	4600.7(12)	34.5(6)
C16	3461(2)	2582(4)	4078.6(15)	49.0(8)
C17	3765(2)	2965(4)	5551.1(15)	57.6(9)
C18	2845.6(19)	6473(3)	5256.3(12)	35.5(6)
C19	3503(2)	7666(4)	5526.1(13)	43.6(7)
C20	3568(2)	8453(4)	6125.9(14)	51.1(8)
C21	2978(2)	8053(4)	6462.3(14)	53.9(9)
C22	2334(2)	6874(5)	6206.1(14)	52.7(8)
C23	2267(2)	6076(4)	5611.6(13)	43.5(7)
C24	1834.3(19)	2173(4)	4577.6(14)	40.0(6)

C25	1699(2)	1365(4)	5117.4(17)	56.3(9)
C26	915(3)	455(5)	5033(2)	66.5(10)
C27	233(3)	337(5)	4410(2)	64.8(10)
C28	344(2)	1123(5)	3867.0(18)	56.8(8)
C29	1126(2)	2014(4)	3947.0(14)	44.9(7)
Si2	1682.7(4)	7041.5(9)	8570.0(3)	28.73(15)
N2	3404.2(14)	5112(3)	8681.0(9)	27.4(4)
C30	4314.6(17)	5384(3)	7977.3(11)	28.6(5)
C31	4540(2)	4916(4)	7419.5(13)	39.7(7)
C32	5121(2)	5870(4)	7196.5(15)	47.5(7)
C33	5500.1(18)	7266(4)	7532.1(14)	42.6(7)
C34	5276.7(18)	7750(4)	8084.9(14)	39.7(6)
C35	4690.2(17)	6822(3)	8300.7(12)	33.0(6)
C36	3686.1(16)	4453(3)	8238.7(11)	27.2(5)
C37	2788.6(17)	4008(3)	8876.4(12)	28.7(5)
C38	2876.9(19)	2367(3)	8556.7(14)	36.4(6)
C39	3341.8(18)	2733(3)	8031.3(13)	33.6(6)
C40	2636(2)	2676(4)	7305.1(14)	45.3(7)
C41	4124(2)	1522(4)	8105.1(17)	48.7(8)
C42	3113.6(18)	3914(4)	9653.2(12)	36.8(6)
C43	4148(2)	3659(5)	9991.6(14)	56.6(9)
C44	2234.3(17)	8082(3)	9407.2(12)	31.1(5)
C45	3189.9(18)	8224(4)	9721.0(13)	39.4(6)
C46	3580(2)	9004(4)	10335.5(14)	43.3(7)
C47	3030(2)	9676(4)	10658.6(15)	46.0(7)
C48	2090(2)	9572(4)	10357.8(16)	54.2(9)
C49	1698(2)	8794(4)	9743.2(15)	44.7(7)
C50	414.9(18)	7432(4)	8260.9(14)	43.6(7)
C51	2123(2)	7951(4)	7930.1(13)	40.5(7)
C52	1799.6(16)	4744(3)	8578.6(11)	27.2(5)
C53	1102.2(16)	3899(3)	8827.0(12)	29.0(5)
C54	980.1(18)	4334(4)	9429.9(13)	36.5(6)
C55	335.5(19)	3570(4)	9646.1(15)	46.8(7)
C56	-211(2)	2361(4)	9264.7(17)	52.1(8)

C57	-105.0(19)	1917(4)	8670.2(18)	52.2(8)
C58	543.8(18)	2671(4)	8453.6(14)	39.8(6)

Table S18. Anisotropic Displacement Parameters (Å^2 ×10^3) for exp_3913_auto. The Anisotropic displacement factor exponent takes the form: -2π^2[h^{2}a*^{2}U$_{11}$+2hka*b*U$_{12}$+…].

Atom	U_{11}	U_{22}	U_{33}	U_{23}	U_{13}	U_{12}
Si1	41.5(4)	34.4(4)	33.8(4)	-3.7(3)	8.9(3)	3.2(3)
N1	38.4(12)	30.5(12)	25.4(10)	-2.9(9)	12.6(9)	0.3(10)
C1	30.7(13)	32.6(14)	27.8(12)	-0.5(10)	9.9(10)	3.8(11)
C2	29.9(12)	34.7(15)	26.9(12)	0.2(10)	10.3(10)	1.7(11)
C3	38.5(14)	34.4(16)	34.0(13)	-5.3(11)	10.8(11)	2.8(12)
C4	44.7(15)	48.2(19)	28.9(13)	-2.5(12)	16.0(11)	9.3(14)
C5	48.4(16)	47.7(19)	34.4(14)	5.3(13)	23.7(12)	6.1(14)
C6	45.8(16)	34.4(16)	40.5(14)	3.3(12)	20.3(12)	-2.6(13)
C7	34.0(13)	29.6(14)	28.3(12)	-1.9(11)	11.2(10)	0.3(11)
C8	53.9(17)	28.5(15)	35.6(13)	-3.6(11)	19.2(12)	-4.3(13)
C9	54.3(17)	32.7(16)	32.4(13)	-6.5(11)	15.9(12)	-0.5(13)
C10	42.9(15)	31.6(15)	26.7(12)	-3.9(10)	13.1(11)	3.0(12)
C11	58.2(19)	49(2)	56.9(18)	-13.0(15)	24.5(15)	-21.5(16)
C12	90(2)	30.2(17)	44.4(16)	1.7(13)	28.7(17)	1.1(16)
C13	41.6(15)	42.6(17)	30.8(13)	-3.3(12)	13.9(11)	5.0(13)
C14	45.2(17)	62(2)	41.1(16)	-3.3(14)	7.9(13)	7.2(15)
C15	38.3(14)	38.5(17)	27.5(12)	-4.8(11)	12.3(11)	-1.8(12)
C16	49.8(17)	43.3(18)	52.9(17)	-11.9(14)	16.6(14)	6.6(15)
C17	59(2)	52(2)	49.1(18)	0.3(15)	1.8(15)	8.7(17)
C18	40.5(15)	34.9(16)	28.4(12)	-4.1(11)	8.4(11)	2.0(12)
C19	53.6(17)	40.0(17)	33.2(14)	-2.4(12)	10.0(12)	-2.4(14)
C20	68(2)	37.1(18)	35.3(14)	-6.4(13)	1.1(14)	-4.2(16)
C21	80(2)	48(2)	28.3(14)	-7.2(13)	12.0(14)	11.8(18)
C22	66(2)	63(2)	30.9(14)	-4.0(15)	19.5(14)	4.5(18)
C23	48.4(17)	50.8(19)	31.1(13)	-6.8(13)	13.5(12)	-2.0(15)
C24	46.4(16)	32.2(16)	43.1(14)	-1.1(12)	17.8(12)	6.9(13)
C25	65(2)	51(2)	55.1(19)	5.7(16)	24.2(17)	5.0(17)
C26	72(2)	60(3)	82(3)	17(2)	45(2)	4(2)

C27	50(2)	55(2)	99(3)	5(2)	37(2)	-1.9(18)
C28	44.3(18)	54(2)	71(2)	-5.1(18)	18.5(16)	-1.4(16)
C29	46.9(16)	41.0(17)	48.5(16)	-2.2(14)	18.5(13)	0.1(15)
Si2	29.9(3)	28.3(4)	28.9(3)	-1.8(3)	11.3(3)	0.6(3)
N2	29.2(10)	30.4(12)	25.1(10)	-0.1(8)	12.6(8)	1.4(9)
C30	28.9(12)	31.0(14)	27.8(12)	2.6(10)	12.4(10)	5.7(11)
C31	50.4(17)	37.8(17)	40.5(15)	-2.3(12)	28.0(13)	3.5(13)
C32	56.3(18)	53(2)	48.5(16)	5.3(15)	37.0(14)	7.9(16)
C33	34.9(14)	47(2)	52.8(16)	13.3(14)	24.1(13)	4.2(13)
C34	34.5(14)	43.8(17)	42.1(14)	1.0(13)	14.8(11)	-4.2(13)
C35	31.4(13)	39.0(16)	30.5(12)	-1.1(11)	13.0(10)	-0.3(12)
C36	28.5(12)	26.9(13)	26.8(12)	-0.1(10)	10.4(10)	2.2(11)
C37	32.6(13)	27.3(14)	29.6(12)	3.2(10)	15.2(10)	1.6(11)
C38	43.6(15)	29.2(15)	43.7(14)	2.6(11)	24.5(12)	1.9(12)
C39	40.4(14)	26.0(13)	40.6(14)	-5.1(11)	22.0(11)	1.5(12)
C40	52.2(17)	45.4(18)	39.8(15)	-15.5(13)	17.8(13)	-11.0(15)
C41	57.2(19)	30.2(16)	72(2)	-1.2(14)	39.1(17)	8.5(14)
C42	35.8(14)	46.8(18)	31.5(13)	10.0(12)	16.4(11)	6.3(13)
C43	41.0(16)	89(3)	37.5(15)	18.5(16)	10.8(12)	11.4(17)
C44	33.8(13)	28.3(14)	35.5(12)	-3.4(11)	17.5(10)	-2.1(11)
C45	35.6(14)	46.9(17)	39.5(14)	-11.4(13)	18.1(11)	-2.7(13)
C46	37.3(15)	48.7(19)	42.8(15)	-11.3(13)	12.4(12)	-7.3(14)
C47	55.2(18)	43.4(18)	42.8(16)	-19.4(13)	21.2(14)	-14.7(15)
C48	52.3(19)	59(2)	62.4(19)	-33.1(17)	34.5(16)	-11.4(16)
C49	36.5(14)	49.1(19)	53.9(17)	-20.4(14)	22.3(13)	-7.5(13)
C50	36.1(15)	43.3(19)	46.4(16)	-2.1(13)	8.2(12)	5.7(13)
C51	50.6(16)	35.1(17)	37.8(14)	2.5(12)	17.9(12)	-0.3(13)
C52	31.1(13)	28.2(14)	24.0(11)	-3.3(9)	11.4(10)	0.1(10)
C53	26.1(12)	28.3(14)	31.2(12)	0.0(10)	8.2(10)	-0.4(10)
C54	32.1(13)	42.7(17)	36.4(13)	-4.9(12)	14.3(11)	-8.0(12)
C55	38.4(15)	56(2)	53.8(17)	1.2(15)	26.2(13)	-6.5(15)
C56	39.1(16)	45(2)	79(2)	9.7(16)	28.3(15)	-6.5(14)
C57	33.1(15)	38.3(18)	79(2)	-8.0(17)	12.1(14)	-11.6(14)
C58	33.5(14)	37.8(16)	43.7(15)	-8.3(12)	7.9(11)	-2.7(12)

Table S19. Bond Lengths for exp_3913_auto.

Atom	Atom	Length/Å	Atom	Atom	Length/Å
Si1	C15	1.907(3)	Si2	C44	1.881(3)
Si1	C16	1.868(3)	Si2	C50	1.866(3)
Si1	C17	1.871(3)	Si2	C51	1.866(3)
Si1	C24	1.874(3)	Si2	C52	1.908(3)
N1	C7	1.277(3)	N2	C36	1.279(3)
N1	C10	1.477(3)	N2	C37	1.476(3)
C1	C2	1.392(4)	C30	C31	1.391(3)
C1	C6	1.396(4)	C30	C35	1.394(4)
C1	C7	1.495(3)	C30	C36	1.487(3)
C2	C3	1.380(3)	C31	C32	1.393(4)
C3	C4	1.390(4)	C32	C33	1.375(5)
C4	C5	1.375(4)	C33	C34	1.383(4)
C5	C6	1.383(4)	C34	C35	1.379(4)
C7	C8	1.529(4)	C36	C39	1.529(4)
C8	C9	1.544(4)	C37	C38	1.542(4)
C8	C11	1.543(4)	C37	C42	1.538(3)
C8	C12	1.526(4)	C37	C52	1.561(3)
C9	C10	1.541(4)	C38	C39	1.544(3)
C10	C13	1.541(4)	C39	C40	1.542(4)
C10	C15	1.567(4)	C39	C41	1.537(4)
C13	C14	1.528(4)	C42	C43	1.523(4)
C15	C18	1.517(3)	C44	C45	1.398(4)
C18	C19	1.389(4)	C44	C49	1.393(3)
C18	C23	1.389(4)	C45	C46	1.384(4)
C19	C20	1.394(4)	C46	C47	1.377(4)
C20	C21	1.375(5)	C47	C48	1.372(4)
C21	C22	1.364(5)	C48	C49	1.383(4)
C22	C23	1.387(4)	C52	C53	1.521(3)
C24	C25	1.396(4)	C53	C54	1.394(3)
C24	C29	1.405(4)	C53	C58	1.388(4)
C25	C26	1.385(5)	C54	C55	1.383(4)
C26	C27	1.373(5)	C55	C56	1.374(4)

C27	C28	1.377(5)	C56	C57	1.368(4)
C28	C29	1.375(4)	C57	C58	1.385(4)

Table S20. Bond Angles for exp_3913_auto.

Atom	Atom	Atom	Angle/˚	Atom	Atom	Atom	Angle/˚
C16	Si1	C15	109.41(14)	C44	Si2	C52	115.86(11)
C16	Si1	C17	106.12(15)	C50	Si2	C44	109.15(12)
C16	Si1	C24	109.82(13)	C50	Si2	C52	105.08(13)
C17	Si1	C15	105.87(14)	C51	Si2	C44	109.42(12)
C17	Si1	C24	109.03(15)	C51	Si2	C50	106.24(13)
C24	Si1	C15	116.07(13)	C51	Si2	C52	110.56(12)
C7	N1	C10	110.9(2)	C36	N2	C37	110.8(2)
C2	C1	C6	118.0(2)	C31	C30	C35	117.8(2)
C2	C1	C7	118.8(2)	C31	C30	C36	123.8(2)
C6	C1	C7	123.2(2)	C35	C30	C36	118.4(2)
C3	C2	C1	120.9(2)	C30	C31	C32	120.4(3)
C2	C3	C4	120.3(3)	C33	C32	C31	120.8(3)
C5	C4	C3	119.6(2)	C32	C33	C34	119.3(3)
C4	C5	C6	120.1(3)	C35	C34	C33	120.0(3)
C5	C6	C1	121.1(3)	C34	C35	C30	121.6(2)
N1	C7	C1	119.3(2)	N2	C36	C30	118.7(2)
N1	C7	C8	115.5(2)	N2	C36	C39	115.6(2)
C1	C7	C8	125.1(2)	C30	C36	C39	125.7(2)
C7	C8	C9	100.0(2)	N2	C37	C38	105.04(18)
C7	C8	C11	109.1(2)	N2	C37	C42	108.9(2)
C11	C8	C9	111.6(2)	N2	C37	C52	106.41(19)
C12	C8	C7	115.2(2)	C38	C37	C52	113.0(2)
C12	C8	C9	109.7(2)	C42	C37	C38	112.0(2)
C12	C8	C11	110.8(3)	C42	C37	C52	111.15(19)
C10	C9	C8	106.1(2)	C37	C38	C39	105.9(2)
N1	C10	C9	104.69(19)	C36	C39	C38	100.4(2)
N1	C10	C13	109.2(2)	C36	C39	C40	111.6(2)
N1	C10	C15	106.7(2)	C36	C39	C41	113.2(2)
C9	C10	C13	111.9(2)	C40	C39	C38	111.1(2)
C9	C10	C15	112.0(2)	C41	C39	C38	110.2(2)

C13	C10	C15	111.9(2)	C41	C39	C40	110.0(2)
C14	C13	C10	114.2(2)	C43	C42	C37	114.1(2)
C10	C15	Si1	117.48(18)	C45	C44	Si2	122.91(19)
C18	C15	Si1	113.52(18)	C49	C44	Si2	120.9(2)
C18	C15	C10	113.1(2)	C49	C44	C45	116.2(2)
C19	C18	C15	120.4(2)	C46	C45	C44	121.9(2)
C23	C18	C15	122.0(2)	C47	C46	C45	120.5(3)
C23	C18	C19	117.6(2)	C48	C47	C46	118.9(3)
C18	C19	C20	121.0(3)	C47	C48	C49	120.8(3)
C21	C20	C19	120.2(3)	C48	C49	C44	121.9(3)
C22	C21	C20	119.4(3)	C37	C52	Si2	117.95(17)
C21	C22	C23	120.9(3)	C53	C52	Si2	112.63(17)
C22	C23	C18	120.9(3)	C53	C52	C37	113.2(2)
C25	C24	Si1	121.9(2)	C54	C53	C52	122.0(2)
C25	C24	C29	116.0(3)	C58	C53	C52	121.1(2)
C29	C24	Si1	122.1(2)	C58	C53	C54	116.8(2)
C26	C25	C24	121.8(3)	C55	C54	C53	121.5(3)
C27	C26	C25	120.6(3)	C56	C55	C54	120.5(3)
C26	C27	C28	119.1(3)	C57	C56	C55	119.1(3)
C29	C28	C27	120.5(3)	C56	C57	C58	120.7(3)
C28	C29	C24	122.0(3)	C57	C58	C53	121.5(3)

Table S21. Torsion Angles for exp_3913_auto.

A	B	C	D	Angle/°	A	B	C	D	Angle/°
Si1	C15	C18	C19	122.6(3)	Si2	C44	C45	C46	179.6(2)
Si1	C15	C18	C23	-56.7(3)	Si2	C44	C49	C48	-179.7(3)
Si1	C24	C25	C26	-177.8(3)	Si2	C52	C53	C54	-50.8(3)
Si1	C24	C29	C28	178.5(3)	Si2	C52	C53	C58	128.0(2)
N1	C7	C8	C9	-10.7(3)	N2	C36	C39	C38	-8.7(3)
N1	C7	C8	C11	106.5(3)	N2	C36	C39	C40	109.1(3)
N1	C7	C8	C12	-128.2(3)	N2	C36	C39	C41	-126.1(2)
N1	C10	C13	C14	-48.0(3)	N2	C37	C38	C39	-15.0(3)
N1	C10	C15	Si1	-40.9(3)	N2	C37	C42	C43	-47.0(3)
N1	C10	C15	C18	-176.2(2)	N2	C37	C52	Si2	-36.3(2)
C1	C2	C3	C4	0.0(4)	N2	C37	C52	C53	-170.86(19)

C1	C7	C8	C9	170.8(2)	C30	C31	C32	C33	1.7(5)
C1	C7	C8	C11	-71.9(3)	C30	C36	C39	C38	170.6(2)
C1	C7	C8	C12	53.4(4)	C30	C36	C39	C40	-71.6(3)
C2	C1	C6	C5	1.1(4)	C30	C36	C39	C41	53.2(3)
C2	C1	C7	N1	7.1(4)	C31	C30	C35	C34	-0.8(4)
C2	C1	C7	C8	-174.5(2)	C31	C30	C36	N2	-168.7(2)
C2	C3	C4	C5	0.2(4)	C31	C30	C36	C39	12.0(4)
C3	C4	C5	C6	0.2(4)	C31	C32	C33	C34	-2.0(5)
C4	C5	C6	C1	-0.9(4)	C32	C33	C34	C35	1.0(4)
C6	C1	C2	C3	-0.7(4)	C33	C34	C35	C30	0.5(4)
C6	C1	C7	N1	-174.3(2)	C35	C30	C31	C32	-0.2(4)
C6	C1	C7	C8	4.1(4)	C35	C30	C36	N2	10.3(3)
C7	N1	C10	C9	9.8(3)	C35	C30	C36	C39	-169.0(2)
C7	N1	C10	C13	129.8(2)	C36	N2	C37	C38	10.1(3)
C7	N1	C10	C15	-109.0(2)	C36	N2	C37	C42	130.2(2)
C7	C1	C2	C3	178.0(2)	C36	N2	C37	C52	-110.0(2)
C7	C1	C6	C5	-177.5(2)	C36	C30	C31	C32	178.7(3)
C7	C8	C9	C10	15.5(3)	C36	C30	C35	C34	-179.9(2)
C8	C9	C10	N1	-16.0(3)	C37	N2	C36	C30	179.9(2)
C8	C9	C10	C13	-134.2(2)	C37	N2	C36	C39	-0.8(3)
C8	C9	C10	C15	99.2(2)	C37	C38	C39	C36	13.8(3)
C9	C10	C13	C14	67.4(3)	C37	C38	C39	C40	-104.4(3)
C9	C10	C15	Si1	-154.88(18)	C37	C38	C39	C41	133.5(2)
C9	C10	C15	C18	69.8(3)	C37	C52	C53	C54	86.2(3)
C10	N1	C7	C1	179.3(2)	C37	C52	C53	C58	-95.0(3)
C10	N1	C7	C8	0.7(3)	C38	C37	C42	C43	68.7(3)
C10	C15	C18	C19	-100.2(3)	C38	C37	C52	Si2	-151.06(16)
C10	C15	C18	C23	80.4(3)	C38	C37	C52	C53	74.4(2)
C11	C8	C9	C10	-99.7(3)	C42	C37	C38	C39	-133.0(2)
C12	C8	C9	C10	137.1(2)	C42	C37	C52	Si2	82.1(2)
C13	C10	C15	Si1	78.6(2)	C42	C37	C52	C53	-52.5(3)
C13	C10	C15	C18	-56.8(3)	C44	C45	C46	C47	-0.2(5)
C15	Si1	C24	C25	-111.3(3)	C45	C44	C49	C48	-1.1(5)
C15	Si1	C24	C29	70.7(3)	C45	C46	C47	C48	-0.6(5)

C15	C10	C13	C14	-166.0(2)	C46	C47	C48	C49	0.5(5)
C15	C18	C19	C20	179.6(3)	C47	C48	C49	C44	0.3(5)
C15	C18	C23	C22	-179.2(3)	C49	C44	C45	C46	1.1(4)
C16	Si1	C24	C25	124.0(3)	C50	Si2	C44	C45	-170.1(2)
C16	Si1	C24	C29	-54.0(3)	C50	Si2	C44	C49	8.4(3)
C17	Si1	C24	C25	8.1(3)	C51	Si2	C44	C45	-54.2(3)
C17	Si1	C24	C29	-169.9(2)	C51	Si2	C44	C49	124.3(2)
C18	C19	C20	C21	0.1(5)	C52	Si2	C44	C45	71.6(3)
C19	C18	C23	C22	1.4(4)	C52	Si2	C44	C49	-109.9(2)
C19	C20	C21	C22	0.4(5)	C52	C37	C38	C39	100.6(2)
C20	C21	C22	C23	-0.1(5)	C52	C37	C42	C43	-163.9(3)
C21	C22	C23	C18	-0.9(5)	C52	C53	C54	C55	179.2(3)
C23	C18	C19	C20	-1.0(4)	C52	C53	C58	C57	-178.7(3)
C24	C25	C26	C27	-1.0(6)	C53	C54	C55	C56	-0.6(5)
C25	C24	C29	C28	0.4(5)	C54	C53	C58	C57	0.1(4)
C25	C26	C27	C28	0.8(6)	C54	C55	C56	C57	0.4(5)
C26	C27	C28	C29	-0.1(6)	C55	C56	C57	C58	0.1(5)
C27	C28	C29	C24	-0.5(5)	C56	C57	C58	C53	-0.3(5)
C29	C24	C25	C26	0.4(5)	C58	C53	C54	C55	0.3(4)

Table S22. Hydrogen Atom Coordinates (Å×10[4]) and Isotropic Displacement Parameters (Å[2]×10[3]) for exp_3913_auto.

Atom	*x*	*y*	*z*	U(eq)
H2	1680.11	3237.78	2489.92	36
H3	1688.25	1944.16	1513.66	43
H4	2322.12	3225.62	784.75	48
H5	2951.2	5800.74	1044.73	49
H6	2963.09	7086.79	2028.47	47
H9A	2660.78	8561.12	4179.63	48
H9B	1626.1	8703.62	3646.44	48
H11A	3887.17	7120.03	3791.31	81
H11B	3824.61	9019.04	3636.82	81
H11C	3836.23	7761.54	3062.48	81
H12A	2413.05	9085.87	2310.85	80
H12B	2235.24	10206.91	2872.63	80

H12C	1465.93	8938.89	2456.11	80
H13A	982.6	5131.76	4250.23	46
H13B	1016.12	7030.22	4397.25	46
H14A	202.03	5595.69	3089.65	77
H14B	276.75	7507.84	3212.06	77
H14C	-354.73	6442.91	3513.57	77
H15	3353.07	5989.86	4521.75	41
H16A	3010.97	2613.22	3617.48	73
H16B	3670.57	1467.71	4195.83	73
H16C	3990.47	3266.59	4104.8	73
H17A	4318.09	3620.18	5619.29	86
H17B	3933.12	1817.51	5597.98	86
H17C	3489.36	3258.15	5890.01	86
H19	3914.98	7950.4	5298.17	52
H20	4021	9267.95	6302.84	61
H21	3017.89	8593.22	6869.53	65
H22	1925.82	6594.66	6437.9	63
H23	1819.9	5247.66	5445.24	52
H25	2157.33	1442.19	5554.69	68
H26	848.95	-93.93	5409.96	80
H27	-307.81	-276.89	4354.64	78
H28	-124.06	1048.65	3433.63	68
H29	1188.79	2537.52	3564.01	54
H31	4296.81	3940.12	7189.27	48
H32	5256.61	5551.99	6807.79	57
H33	5911.28	7892.5	7385.96	51
H34	5527.35	8721.66	8316.08	48
H35	4538.57	7172.27	8678.83	40
H38A	2261.39	1876.83	8334.65	44
H38B	3258.99	1611.67	8906.21	44
H40A	2137.48	3446.12	7263.23	68
H40B	2380.04	1582.92	7206.66	68
H40C	2942.26	2962.68	6983.61	68
H41A	4397.04	1736.24	7757.05	73

H41B	3877.13	419.34	8051.22	73
H41C	4598.41	1636.7	8554.16	73
H42A	2938.29	4927.01	9827.57	44
H42B	2786.21	3013.52	9782.57	44
H43A	4334.63	2683.41	9808.31	85
H43B	4297.81	3535.14	10480.62	85
H43C	4478.58	4595.72	9903.46	85
H45	3582.5	7772.97	9506.17	47
H46	4231.85	9075.98	10536.02	52
H47	3296.59	10202.95	11082.59	55
H48	1703.32	10038.78	10574.15	65
H49	1045.46	8744.06	9544.62	54
H50A	156.67	7052.24	8598.54	65
H50B	120.5	6854.63	7834.01	65
H50C	301.71	8594.68	8187.81	65
H51A	1878.48	9048.89	7822.17	61
H51B	1923.8	7290.58	7518.34	61
H51C	2798.49	7990.08	8113.8	61
H52	1594.99	4451.75	8088.08	33
H54	1347.76	5174.08	9698.2	44
H55	270.07	3883.13	10061	56
H56	-656.05	1840.76	9411.82	63
H57	-480.3	1083.41	8403.19	63
H58	607.97	2339.08	8040.37	48

Experimental

Single crystals of $C_{29}H_{35}NSi$ **[exp_3913_auto]** were **[]**. A suitable crystal was selected and **[]** on a **XtaLAB AFC12 (RINC): Kappa dual home/near** diffractometer. The crystal was kept at 173.00(10) K during data collection. Using Olex2 [1], the structure was solved with the SHELXT [2] structure solution program using Intrinsic Phasing and refined with the SHELXL [3] refinement package using Least Squares minimisation.

1. Dolomanov, O.V., Bourhis, L.J., Gildea, R.J, Howard, J.A.K. & Puschmann, H. (2009), J. Appl. Cryst. 42, 339-341.

2. Sheldrick, G.M. (2015). Acta Cryst. A71, 3-8.
3. Sheldrick, G.M. (2015). Acta Cryst. C71, 3-8.

Crystal structure determination of [exp_3913_auto]

Crystal Data for $C_{29}H_{35}NSi$ (M =425.67 g/mol): monoclinic, space group $P2_1$ (no. 4), a = 15.4543(3) Å, b = 8.27200(10) Å, c = 21.0647(3) Å, β = 110.261(2) °, V = 2526.25(7) Å^3, Z = 4, T = 173.00(10) K, μ(Cu Kα) = 0.914 mm^{-1}, $Dcalc$ = 1.119 g/cm^3, 64256 reflections measured (6.096° ≤ 2Θ ≤ 134.152°), 8977 unique (R_{int} = 0.0671, R_{sigma} = 0.0381) which were used in all calculations. The final R_1 was 0.0345 (I > 2σ(I)) and wR_2 was 0.0832 (all data).

Refinement model description

Number of restraints - 1, number of constraints - unknown.

Details:

1. Fixed Uiso

At 1.2 times of:

All C(H) groups, All C(H,H) groups

At 1.5 times of:

All C(H,H,H) groups

2.a Ternary CH refined with riding coordinates:

C15(H15), C52(H52)

2.b Secondary CH2 refined with riding coordinates:

C9(H9A,H9B), C13(H13A,H13B), C38(H38A,H38B), C42(H42A,H42B)

2.c Aromatic/amide H refined with riding coordinates:

C2(H2), C3(H3), C4(H4), C5(H5), C6(H6), C19(H19), C20(H20), C21(H21), C22(H22), C23(H23), C25(H25), C26(H26), C27(H27), C28(H28), C29(H29), C31(H31), C32(H32), C33(H33), C34(H34), C35(H35), C45(H45), C46(H46), C47(H47), C48(H48), C49(H49), C54(H54), C55(H55), C56(H56), C57(H57), C58(H58)

2.d Idealised Me refined as rotating group:

C11(H11A,H11B,H11C), C12(H12A,H12B,H12C), C14(H14A,H14B,H14C),

C16(H16A,H16B,

H16C), C17(H17A,H17B,H17C), C40(H40A,H40B,H40C), C41(H41A,H41B,H41C),

C43(H43A,

H43B,H43C), C50(H50A,H50B,H50C), C51(H51A,H51B,H51C)

7. Mechanism studies

7.1 Analysis of the crude reaction mixture of 1e by ^{19}F NMR

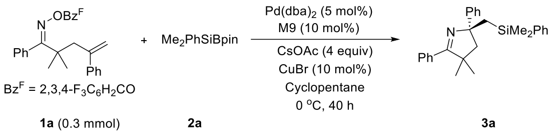

For the reaction of oxime ester **1a** and **2a**, the crude reaction mixture was analyzed by ^{19}F NMR to probe the fate of the OBz^F leaving group.

Under nitrogen atmosphere, to an oven-dried 10 mL Schlenk tube equipped with a magnetic stir was added Pd(dba)$_2$ (8.5 mg, 0.015 mmol, 5 mol%), ligand **M9** (23.9 mg, 0.03 mmol, 10 mol%) and cyclopentane (3 mL). The catalyst/ligand solution was stirred for 1.0 h at 25 °C, CsOAc (230.3 mg, 1.20 mmol, 4.0 equiv), oxime esters **1a** (0.30 mmol, 1.0 equiv), silylboronic ester **2a** (0.75 mmol, 2.5 equiv) were added successively. The resulting mixture was then stirred vigorously at 0 °C for about 40 h. After completion of the reaction (monitored by TLC), the reaction mixture was concentrated to dryness and the residue was purified by column chromatography (petroleum ether/ethyl acetate) to afford desired product **3a**.

1,2,3-trifluorobenzene, 2,3,4-trifluorobenzoic acid and the corresponding cesium 2,3,4-trifluorobenzoate were considered as possible products in the reaction. Their ^{19}F NMR spectra were compared with the ^{19}F NMR spectra of crude reaction mixture. No 1,2,3-trifluorobenzene was observed in the reaction mixture.

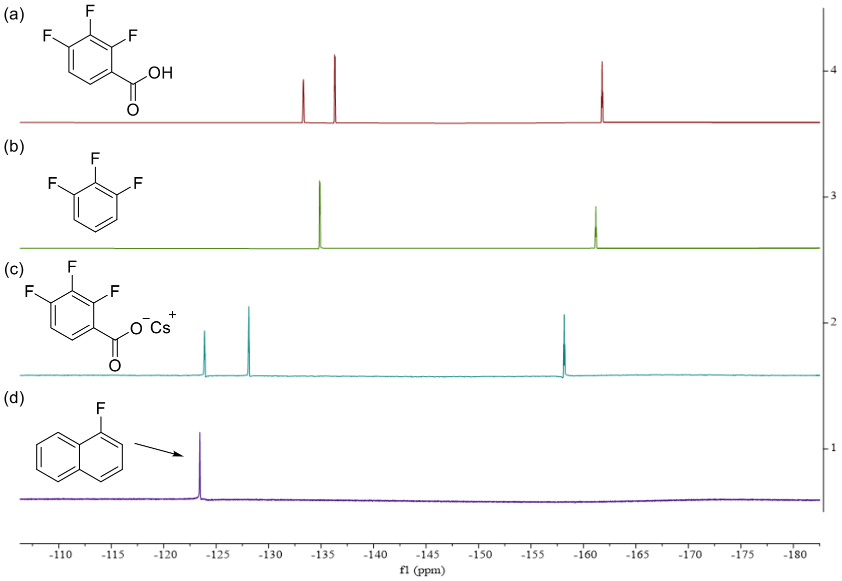

Figure S4. (a) ^{19}F NMR spectra of 2,3,4-trifluorobenzoic acid in cyclopentane; (b) ^{19}F NMR spectra of 1,2,3-trifluorobenzene in cyclopentane; (c) ^{19}F NMR spectra of cesium 2,3,4-trifluorobenzoate in ethanol; (d) ^{19}F NMR spectra of the reaction mixture.

8. DFT studies

All the density functional theory (DFT) calculations were performed using Gaussian 09 program.[11] Geometry optimizations were performed with the B3LYP5-GD3 functional[12] using a combined basis set (Lanl2DZ[5] for Palladium and 6-31G(d) basis[13] for the other atoms). Harmonic frequency calculations were performed for each stationary point to ensure that it is either an energy minimum (no imaginary frequency) or a transition state (only one imaginary frequency). For each transition state, intrinsic reaction coordinate (IRC) analysis was performed to ensure that it connects the correct reactant and product. The single-point energy calculations were further performed with the B3LYP-GD3 functional and a combined basis set (SDD[14] for Palladium and the 6-311+G(d,p) basis set[15] for all other atoms), using a self-consistent reaction field (SCRF) method called IEFPCM[16] in order to obtain energies in solution. The single-point energies corrected by the thermal correction to Gibbs free energies (TCG, obtained from frequency calculations) were used as the Gibbs free energies reported in this work, corresponding to the reference state of 1 mol/L, 298.15 K. The independent gradient model (IGM) was conducted with Multiwfn and visualized using VMD.[17] The 3-D images of the calculated structures were prepared using CYLview.[18]

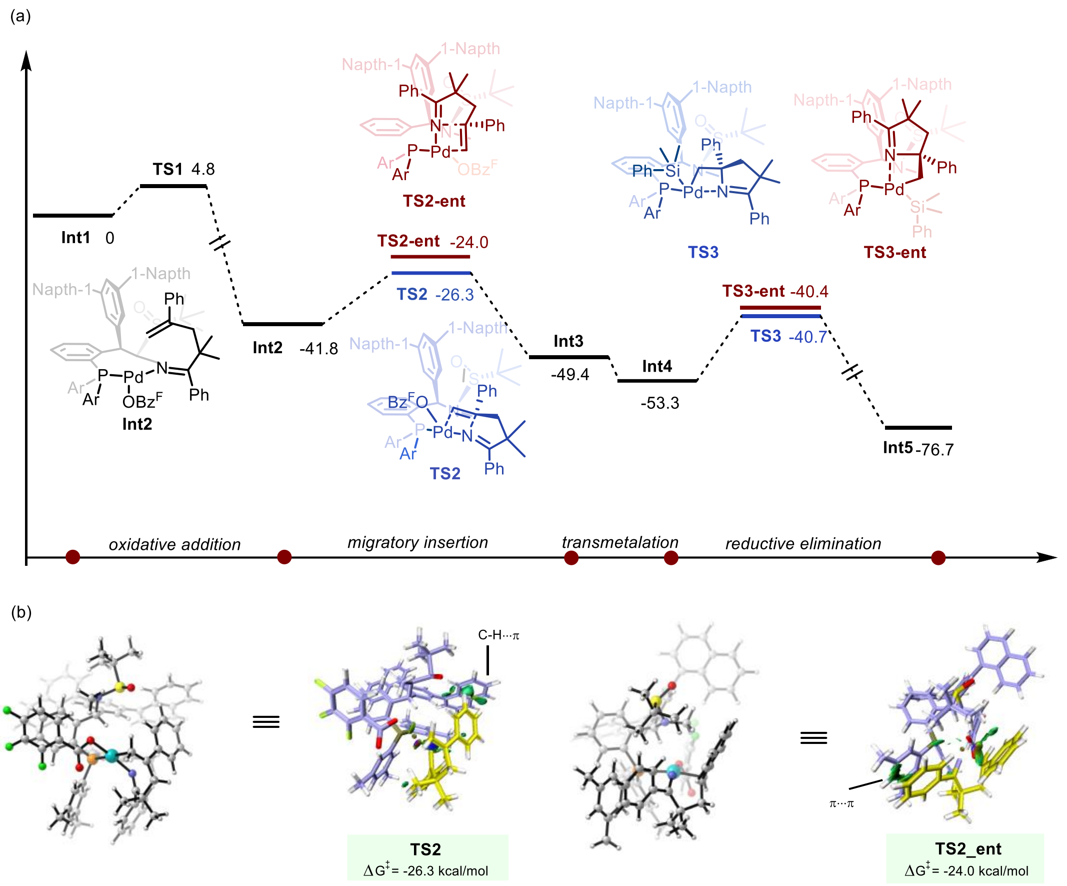

Figure S5. (a) Computed pathways for catalytic aza-Heck cyclization/sonogashira reaction. (b) Geometries for the transition-state structure of migratory insertion and IGM analysis of the transition states. Bz^F = 2,3,4-$F_3C_6H_2CO$.

3. Absolutely Localized Molecular Orbital Energy Decomposition Analysis

The interaction energy in a complex is decomposed into four energy subitems with distinct physical meanings by symmetry-adapted perturbation theory (SAPT), i.e. the electrostatic E_{ele}, the exchange E_{ex}, the induction E_{ind} and the dispersion E_{disp}, respectively.

Table S23. Details of energy decomposition analysis for **TS2** and **TS2-ent** (unit: kcal/mol).

	**TS2**	**TS2-ent**
E_{ele}	-175.60854012	-172.05219510
E_{ex}	137.79448687	134.25173455
E_{ind}	-150.08009431	-156.13018464
**E_{disp}**	**-72.27722854**	**-68.97262075**

Table S24. Energy data (hartrees).

Geometry	$E_{B3LYP/6\text{-}31G(d)}$	TCG	$E_{M06\text{-}L/6\text{-}311+G(d,p)}$	TCG+$E_{M06\text{-}L/6\text{-}311+G(d,p)}$	Imaginary Frequency (cm^{-1})
Int1	-4594.23521190	1.221787	-4595.769621	-4594.547834	
TS1	-4594.21954113	1.219060	-4595.759311	-4594.540251	212.29i
Int2	-4595.84060284	1.226121	-4595.8406028	-4594.614482	
TS2	-4594.27747754	1.222325	-4595.812048	-4594.589723	319.72i
TS2-ent	-4594.278186	1.227448	-4595.813542	-4594.586094	368.41i
Int3	-4595.852262	1.225626	-4594.626636	-4594.626636	
Int3-ent	-4594.29296264	1.223532	-4595.850970	-4594.627438	
Int4	-4476.110871	1.302389	-4478.853265	-4477.550876	
Int4-ent	-4475.470128	1.308245	-4478.86181	-4477.553565	
TS3	-4476.0847	1.31079	-4478.841646	-4477.530856	99.62i
TS3-ent	-4476.082677	1.311186	-4478.841562	-4477.530376	111.46i
Int5	-4475.505646	1.295705	-4478.883839	-4477.588134	
Int5-ent	-4475.487167	1.305647	-4478.873734	-4477.568087	
SM1 (Cu-SiPhMe2)	-797.036654	0.125475	-798.3953223	-798.2698473	
SM2 (CuO_2CAr^F; Ar^F = 2,3,4-$F_3C_6H_2$)	-914.032317	0.041808	-915.3937619	-915.3519539	

Int1

S	2.21656800	0.48867300	4.09288300
O	2.84343600	1.81968000	4.45837300
N	1.51694300	0.62081900	2.50594700
C	1.10993400	-4.76295000	0.43941800
H	1.79463600	-4.32873900	1.16478400
C	1.34395400	-6.05229600	-0.03836900
C	0.44768500	-6.59034800	-0.97689900
H	0.62337500	-7.59276100	-1.36249200
C	-0.66139300	-5.87037200	-1.42774600
C	-0.87300500	-4.57373200	-0.93233200
H	-1.72153100	-3.99950900	-1.29311100
C	-1.62986800	-1.58263200	-0.24393800
C	-1.63722000	-1.16699600	-1.58832200
H	-0.74180300	-1.28360600	-2.18860300
C	-2.77177500	-0.59034900	-2.16160800
C	-3.89793200	-0.38524000	-1.34876600
H	-4.76678000	0.11887800	-1.76629000
C	-3.92183800	-0.78626300	-0.01165700
C	-2.78364300	-1.40859700	0.52661100
H	-2.79581000	-1.71395200	1.56862500
C	-0.39211900	-2.23234500	2.28123700
C	-0.75278200	-3.41821400	2.93835400
H	-0.82034100	-4.34374400	2.37640300
C	-1.03304100	-3.42911400	4.30477300
H	-1.30636200	-4.36006000	4.79409300
C	-0.98391100	-2.23754600	5.02600700
C	-0.64060500	-1.05087100	4.37856900
H	-0.62673200	-0.12882100	4.94648200
C	-0.31906800	-1.02427000	3.01612200

C	1.91950000	1.82917200	1.76758100
H	1.43036000	2.73498600	2.14681100
H	1.65522300	1.69192300	0.71775900
H	2.99578400	1.96871700	1.84194500
C	3.65166700	-0.67717800	3.69002200
C	3.03751300	-2.02155000	3.29232700
H	3.84195400	-2.75416100	3.15502000
H	2.49721700	-1.93362000	2.34638200
H	2.34917800	-2.40267500	4.05597800
C	4.53272100	-0.11065500	2.57800300
H	4.00378900	-0.09510200	1.62099900
H	5.41481300	-0.74917700	2.45188500
H	4.86996800	0.90074700	2.82815700
C	4.42822300	-0.78157200	5.01004000
H	5.27068300	-1.47151300	4.87997400
H	3.79874000	-1.16885000	5.82098500
H	4.82038300	0.19432800	5.31173200
C	-0.89222400	1.43764100	2.59965400
C	-1.81213700	1.73276600	1.59240800
H	-1.82935900	1.13279000	0.68874200
C	-2.72160300	2.78904500	1.71911300
C	-2.67002700	3.58235000	2.87311500
H	-3.33065500	4.43971300	2.96214900
C	-1.74349200	3.31829400	3.89702300
C	-0.86919400	2.22821400	3.74972300
H	-0.15383200	2.01924400	4.53758400
C	-1.85613500	5.67695900	7.51646300
C	-0.58849100	5.41197700	6.93268800
C	-0.50849500	4.65855100	5.70993600
C	-1.72760900	4.15986000	5.12507300

C	-2.93702400	4.45516800	5.73642900
H	-3.85418900	4.06326000	5.30515600
C	-3.00834600	5.21615600	6.92418800
C	-3.65813500	3.03960800	0.58929200
C	-5.08277400	3.03254600	0.76009100
C	-5.91806700	3.19152200	-0.39840200
C	-5.32153900	3.35443800	-1.67804700
C	-3.95254500	3.34969500	-1.81736400
C	-3.12816100	3.19010400	-0.68139000
H	-2.05087500	3.17654300	-0.80723300
C	0.07814800	0.29085200	2.33889300
H	0.00667200	0.12188700	1.26147100
H	-1.21810100	-2.22431400	6.08674600
C	2.56718400	-6.82234000	0.40331700
H	3.43506900	-6.56199900	-0.21773600
H	2.83087000	-6.59300800	1.44164500
H	2.41688300	-7.90365900	0.31957800
C	-1.61020400	-6.44580600	-2.45253000
H	-2.65166200	-6.36905400	-2.11873900
H	-1.52920600	-5.89584700	-3.39849100
H	-1.39485800	-7.49938200	-2.65718300
C	-2.79161900	-0.18865500	-3.61795800
H	-3.50995200	-0.79569600	-4.18194000
H	-3.08359300	0.86039600	-3.73701200
H	-1.81474200	-0.32868000	-4.08982600
C	-5.12778300	-0.53630100	0.86305300
H	-4.84714900	0.03031900	1.75793300
H	-5.89395700	0.04231300	0.33904600
H	-5.57815000	-1.47883700	1.19969400
C	0.78049400	4.45912800	5.13907800

H	0.88307200	3.92627700	4.20339900
C	1.92080400	4.91952500	5.75955900
H	2.88667400	4.71433400	5.30836200
C	1.83762700	5.63726300	6.97536000
C	0.60677800	5.88554700	7.53966300
H	0.52790100	6.44984200	8.46624500
H	2.74351400	5.99595300	7.45681800
H	-3.97723700	5.41740100	7.37316700
H	-1.89776200	6.24916400	8.44036200
C	-5.71306300	2.81880400	2.01833200
C	-7.08550400	2.78510100	2.13312900
H	-7.54252400	2.61442100	3.10418000
C	-7.33128800	3.15684200	-0.24258500
C	-7.90632100	2.96123200	0.99281900
H	-8.98777900	2.93403200	1.09527300
H	-7.95351800	3.28217800	-1.12586200
H	-5.09594200	2.66187700	2.89623400
H	-5.96723300	3.47472500	-2.54489500
H	-3.49214400	3.45894500	-2.79420300
Pd	1.76509800	-1.09861500	-0.37848800
C	6.78401900	-1.58797700	-0.30336900
C	6.69886800	-2.64731900	0.60062200
C	5.51446500	-3.38739300	0.68420000
C	4.42993200	-3.07147800	-0.12904000
C	4.50451400	-2.00842300	-1.04534400
C	5.69626500	-1.27134200	-1.12096700
H	7.69683200	-1.00282200	-0.37468600
H	7.54405200	-2.89356700	1.23759500
H	5.43106900	-4.20797800	1.39200400
H	3.50726400	-3.63040800	-0.04253300

H	5.76161800	-0.44347400	-1.81911600
C	3.35203700	-1.62989600	-1.94612300
N	3.15497200	-0.30265800	-1.86331900
O	2.37659200	0.20900800	-2.99149300
C	1.56369200	1.23824600	-2.65851000
O	1.31626000	1.61016300	-1.53287400
C	0.96944900	1.84156700	-3.88418500
C	-0.35467200	2.29077900	-3.84513600
C	1.70311200	2.01809500	-5.06585800
C	-0.94500200	2.87298300	-4.96136700
C	1.13729900	2.63915600	-6.17697000
C	-0.18902500	3.05539700	-6.11758200
F	-0.77428500	3.62504800	-7.17889300
F	-2.23339200	3.24576300	-4.92290000
F	-1.11213700	2.13330000	-2.75371100
C	2.95714500	-2.57271000	-3.13000600
C	3.48538400	-4.00234300	-2.89600400
H	4.56158900	-4.00930000	-2.70665300
H	2.98075000	-4.49336200	-2.05821300
H	3.30346800	-4.59738100	-3.79413500
C	3.64746300	-2.04390000	-4.41116300
H	3.37021800	-2.67955100	-5.25946400
H	3.36856100	-1.01579100	-4.64135600
H	4.73714300	-2.08383900	-4.29836900
C	1.39842800	-2.61521900	-3.25617200
H	1.03424100	-1.59123200	-3.26323400
H	1.03368500	-3.06662000	-2.33068500
C	0.74624900	-3.36157800	-4.41482800
C	0.76124600	-4.70250200	-4.50834200
H	0.28463100	-5.22003600	-5.33634000

H	1.23126600	-5.32468000	-3.75522300
C	-0.06061200	-2.55657900	-5.37467700
C	-1.31508600	-3.02348900	-5.80971900
C	0.36780400	-1.30004500	-5.83770400
C	-2.09846400	-2.27963000	-6.69109500
H	-1.69085700	-3.96642100	-5.42348200
C	-0.41039800	-0.55986600	-6.72825200
H	1.31996000	-0.90005000	-5.50751600
C	-1.64723600	-1.04363200	-7.15924300
H	-3.06864400	-2.65976500	-7.00024900
H	-0.04767800	0.39869300	-7.08367400
H	-2.25712700	-0.45809500	-7.84169200
C	0.01079800	-4.00996200	-0.00961000
P	-0.04945900	-2.23434300	0.46411800
H	1.69740200	2.79781600	-7.09152700
H	2.73073200	1.67512500	-5.09951900

TS1

S	-6.29408100	-4.26739400	4.77955700
O	-5.72480400	-2.87476600	4.97220400
N	-7.09750500	-4.32954000	3.23218200
C	-7.50642000	-9.87737900	0.97017300
H	-6.57139300	-9.35306900	1.14262600
C	-7.48669400	-11.22866600	0.61851700
C	-8.71019900	-11.86972400	0.37475800
H	-8.70945300	-12.92148800	0.09350500
C	-9.93015800	-11.19364200	0.47757900
C	-9.92039700	-9.83850400	0.83915200
H	-10.86133500	-9.30109700	0.92018500
C	-10.23472700	-6.71442400	0.85431700
C	-10.28595100	-6.31406700	-0.49248400

H	-9.41210300	-6.43337200	-1.12416000
C	-11.43266600	-5.72402200	-1.02555700
C	-12.53122000	-5.51442500	-0.17667200
H	-13.41018700	-5.00505900	-0.56601100
C	-12.51473900	-5.91405300	1.16142000
C	-11.35871700	-6.53114700	1.66575300
H	-11.33003100	-6.82286900	2.71194700
C	-8.83859100	-7.28779200	3.32522400
C	-9.03484300	-8.46014400	4.06891800
H	-9.07561800	-9.41707100	3.55939400
C	-9.18901800	-8.41684600	5.45480100
H	-9.33670700	-9.33762500	6.01253100
C	-9.17496400	-7.18659100	6.10841500
C	-8.98494600	-6.01447300	5.37542900
H	-8.98976000	-5.06740600	5.89938300
C	-8.78838300	-6.03673100	3.98955900
C	-6.83529000	-3.14782300	2.39265000
H	-7.25858100	-2.22767400	2.81332500
H	-7.27671400	-3.33284300	1.41216600
H	-5.76242000	-3.00949300	2.27196700
C	-4.81683200	-5.38450500	4.39538600
C	-5.36811900	-6.78823900	4.13603600
H	-4.52789100	-7.48086000	4.00402000
H	-5.97129900	-6.80676700	3.22452800
H	-5.98303200	-7.14917200	4.96897300
C	-4.02415700	-4.85430100	3.20138600
H	-4.59122000	-4.94780000	2.27170300
H	-3.10474300	-5.44192900	3.08706200
H	-3.74761800	-3.80638000	3.35824100
C	-3.97528500	-5.33981400	5.67885400

H	-3.09973800	-5.98906000	5.55888800
H	-4.54225700	-5.69668200	6.54771000
H	-3.62858800	-4.32263100	5.88446700
C	-9.52766400	-3.61999900	3.46921000
C	-10.44389000	-3.34229500	2.45273600
H	-10.44590100	-3.94698600	1.55199100
C	-11.36634400	-2.29497800	2.56422200
C	-11.33034900	-1.48975800	3.70987900
H	-12.00024200	-0.63836100	3.78568400
C	-10.40736500	-1.73334500	4.74199100
C	-9.52181400	-2.81595900	4.61107800
H	-8.81373800	-3.00848000	5.40880300
C	-10.57153200	0.67786600	8.32488500
C	-9.29631400	0.41267600	7.75793400
C	-9.19817600	-0.36041900	6.54861600
C	-10.40814600	-0.87667600	5.95966000
C	-11.62554600	-0.58144600	6.55525000
H	-12.53568300	-0.98697300	6.12173000
C	-11.71436900	0.19800600	7.72967000
C	-12.29701200	-2.06206000	1.42618100
C	-13.72285000	-2.06995200	1.58581800
C	-14.54910400	-1.92474700	0.41879700
C	-13.94293300	-1.77187600	-0.85768000
C	-12.57273600	-1.77356600	-0.98578000
C	-11.75821000	-1.92270700	0.15835900
H	-10.68067900	-1.94021800	0.03999300
C	-8.53003700	-4.74380400	3.20999000
H	-8.67563900	-4.98439100	2.15640900
H	-9.31408200	-7.13143600	7.18443300
C	-6.18056800	-11.97496900	0.47120200

H	-5.95810900	-12.17579700	-0.58478100
H	-5.34244500	-11.40110500	0.87976000
H	-6.21270700	-12.94307100	0.98438400
C	-11.23691000	-11.89160000	0.17826200
H	-12.02528200	-11.57637900	0.87076600
H	-11.58370100	-11.65385600	-0.83586600
H	-11.13759800	-12.97985900	0.24656900
C	-11.49219500	-5.31237900	-2.47769700
H	-12.17991900	-5.95747600	-3.03915900
H	-11.85080600	-4.28229000	-2.57718500
H	-10.51253600	-5.37932900	-2.95865900
C	-13.69294600	-5.65957500	2.07185100
H	-13.38694300	-5.07809600	2.94914300
H	-14.48001200	-5.09409800	1.56510300
H	-14.12530500	-6.59984900	2.43736100
C	-7.90144700	-0.56171700	5.99566100
H	-7.77927400	-1.11081300	5.07183000
C	-6.77101600	-0.08421200	6.62183600
H	-5.80079400	-0.29520600	6.18334100
C	-6.87220400	0.65483000	7.82338400
C	-8.11093000	0.90496200	8.36946900
H	-8.20392500	1.48478100	9.28512500
H	-5.97420600	1.02832200	8.30848100
H	-12.68912500	0.39884700	8.16595900
H	-10.62643500	1.26524300	9.23852900
C	-14.36325400	-2.26761200	2.84149900
C	-15.73647300	-2.29995800	2.94599400
H	-16.20103300	-2.45850700	3.91552800
C	-15.96351900	-1.95855300	0.56389300
C	-16.54831000	-2.13861900	1.79702900

H	-17.63054800	-2.16485100	1.89123100
H	-16.57873400	-1.84430200	-0.32576300
H	-13.75314400	-2.41419900	3.72597900
H	-14.58209000	-1.65906100	-1.73031900
H	-12.10102800	-1.66188700	-1.95692500
Pd	-6.86043500	-6.25302100	0.49509900
C	-1.23953500	-7.23060900	0.14269600
C	-1.33549800	-8.03202300	1.28300400
C	-2.59153300	-8.44505600	1.73667600
C	-3.74328900	-8.06232600	1.05000300
C	-3.65147800	-7.27908300	-0.10597900
C	-2.39212000	-6.85057500	-0.54809300
H	-0.26786100	-6.89085700	-0.20509600
H	-0.43817900	-8.32112700	1.82297300
H	-2.67729900	-9.05075800	2.63482000
H	-4.72466100	-8.33584600	1.42255600
H	-2.31910100	-6.21318100	-1.42391300
C	-4.89274500	-6.86399100	-0.86891200
N	-5.21268200	-5.64293300	-0.50132500
O	-5.95986200	-4.72693000	-1.90342000
C	-7.03841100	-4.07931800	-1.57327700
O	-7.75259700	-4.30000000	-0.58343000
C	-7.41564800	-3.01705400	-2.56276600
C	-8.73645400	-2.55722700	-2.64941500
C	-6.47409800	-2.49171800	-3.46170300
C	-9.10535200	-1.61277700	-3.60358600
C	-6.82567700	-1.53453400	-4.40968000
C	-8.14671200	-1.10344800	-4.47611600
F	-8.52613600	-0.19056800	-5.38011600
F	-10.38060900	-1.20108400	-3.68337500

F	-9.70646200	-3.01309500	-1.84910300
C	-5.33752700	-7.70642300	-2.10293400
C	-4.83821300	-9.15436500	-1.92689600
H	-3.75267100	-9.19592500	-1.80397800
H	-5.30061500	-9.63338000	-1.05789900
H	-5.09359600	-9.73370600	-2.81754900
C	-4.64792000	-7.11133800	-3.35353900
H	-4.97334900	-7.66403100	-4.24171200
H	-4.88586000	-6.05606900	-3.48405800
H	-3.55994100	-7.21209700	-3.27420600
C	-6.90172400	-7.71270200	-2.22039400
H	-7.26269300	-6.70422700	-2.03903500
H	-7.26892300	-8.32528200	-1.39130700
C	-7.55165400	-8.22720500	-3.50096700
C	-7.58323100	-9.53880700	-3.79360000
H	-8.04143800	-9.91509700	-4.70371200
H	-7.16143200	-10.28113700	-3.12400200
C	-8.27383700	-7.23729200	-4.35141100
C	-9.47274300	-7.59082200	-5.00021200
C	-7.82424300	-5.91172900	-4.48975100
C	-10.17665300	-6.66801700	-5.77222100
H	-9.87534300	-8.59006300	-4.86517500
C	-8.52738400	-4.98666100	-5.26279300
H	-6.91994200	-5.59394700	-3.98733700
C	-9.70665100	-5.35919800	-5.90970300
H	-11.10578800	-6.96662800	-6.25055300
H	-8.15093700	-3.97163900	-5.35128800
H	-10.26121300	-4.63554100	-6.50080600
C	-8.71485800	-9.17309800	1.08359800
P	-8.63342300	-7.37958100	1.49111900

H	-6.10054000	-1.11859200	-5.09992200
H	-5.45421100	-2.85196000	-3.40435900

Int2

S	-2.49630900	-0.14788500	-3.24643600
O	-1.66710900	-0.50164200	-4.46998800
N	-1.78550900	1.21763200	-2.41903100
C	3.63181100	1.66328900	1.78454500
H	3.60028400	2.22010500	0.85587200
C	4.73269200	1.81862900	2.62567100
C	4.79673200	1.04817800	3.79806300
H	5.65675700	1.15731400	4.45694200
C	3.79046500	0.14033100	4.13664200
C	2.68460100	0.00924900	3.28248800
H	1.90896300	-0.70762100	3.52985800
C	0.13815000	-0.70487700	1.68555300
C	0.11969000	-2.06605100	1.37159600
H	0.75790900	-2.44355300	0.58395100
C	-0.67796300	-2.95082700	2.10720800
C	-1.47107300	-2.44057200	3.13787300
H	-2.08313100	-3.12459600	3.72131500
C	-1.51399800	-1.07123300	3.43476500
C	-0.68732800	-0.21084000	2.70808500
H	-0.68144200	0.84948900	2.94609800
C	0.32123600	2.00209500	0.62031000
C	0.68184800	3.15457700	1.33703100
H	1.49059600	3.10675300	2.05626600
C	0.04028700	4.37414400	1.12385300
H	0.36119700	5.25651800	1.66954200
C	-0.97786100	4.45484300	0.17770100
C	-1.35620500	3.31419500	-0.52913900

H	-2.15966800	3.37571400	-1.25503800
C	-0.73351000	2.07571600	-0.32733200
C	-0.83756100	1.98002300	-3.26026900
H	-1.13007700	3.03347000	-3.30392100
H	-0.82999500	1.56786700	-4.27249300
H	0.17178200	1.90593100	-2.85707700
C	-4.06284800	0.65665300	-3.91571600
C	-3.71511200	1.83465000	-4.82459200
H	-4.62419800	2.18763400	-5.32672700
H	-2.99497500	1.53114100	-5.59123000
H	-3.29694300	2.66784300	-4.25347800
C	-4.74750300	-0.46476700	-4.71308800
H	-4.11205400	-0.80287100	-5.53715500
H	-5.68814500	-0.08789800	-5.13145600
H	-4.98711500	-1.32754800	-4.07978500
C	-4.90663700	1.07796800	-2.71015000
H	-5.86914000	1.46929000	-3.06151200
H	-4.40361100	1.85609500	-2.13104900
H	-5.10907400	0.23479600	-2.03932600
C	-2.42068400	0.20246700	-0.24725300
C	-2.59641400	-1.18510800	-0.23426100
H	-1.88161700	-1.82125200	-0.74606400
C	-3.68530200	-1.77241500	0.43073400
C	-4.55916900	-0.94657400	1.15246100
H	-5.38234600	-1.39408100	1.70205800
C	-4.38385100	0.44255600	1.18410100
C	-3.33496100	0.99920200	0.44127000
H	-3.23454500	2.07579500	0.42021400
C	-7.14051900	2.97028900	3.34328700
C	-5.74774400	3.18760000	3.51929100

C	-4.80491100	2.34440900	2.83738600
C	-5.30303400	1.31404100	1.96250900
C	-6.67213800	1.13951000	1.83711900
H	-7.04464400	0.37112800	1.16520900
C	-7.59277500	1.95910500	2.52764700
C	-3.89331700	-3.24599900	0.42263100
C	-3.89183200	-4.00663600	-0.79765900
C	-4.00137600	-5.43738200	-0.72845400
C	-4.16023100	-6.06754300	0.53435500
C	-4.23722600	-5.31359400	1.68247400
C	-4.10105100	-3.90857400	1.62136000
H	-4.11811200	-3.33398100	2.54138200
C	-1.28042900	0.84262500	-1.05578000
H	-0.46746000	0.10733900	-1.15296000
H	-1.47949300	5.39916800	-0.01322300
C	5.84507600	2.77089900	2.25107200
H	6.70847100	2.22610400	1.84722800
H	5.51552900	3.47732200	1.48288100
H	6.19641400	3.33995500	3.11975200
C	3.87584300	-0.70693200	5.38487500
H	3.90933400	-1.77232400	5.12724200
H	4.77070400	-0.47229700	5.97083500
H	3.00123500	-0.55486900	6.02926200
C	-0.65112300	-4.42488200	1.78142200
H	-1.39964400	-4.98061400	2.35212300
H	-0.85042000	-4.59020400	0.71677900
H	0.33658700	-4.85030600	1.99936600
C	-2.46252400	-0.53753800	4.48140900
H	-3.44111800	-0.31985100	4.03418700
H	-2.61960100	-1.26194400	5.28819700

H	-2.09965800	0.39663400	4.92154700
C	-3.41862800	2.56194800	3.08520300
H	-2.68755400	1.91376400	2.61966100
C	-2.98724600	3.57457400	3.91323800
H	-1.92190400	3.72025400	4.07050300
C	-3.91975600	4.42271700	4.55679100
C	-5.26848300	4.22316100	4.36840500
H	-5.99449000	4.85774900	4.87138600
H	-3.56922300	5.22114800	5.20507500
H	-8.65864700	1.79534200	2.39478700
H	-7.84164900	3.61677400	3.86560400
C	-3.83981000	-3.40390400	-2.08495800
C	-3.84762200	-4.16249800	-3.23487000
H	-3.80510100	-3.67112500	-4.20306900
C	-3.98077700	-6.19378700	-1.93322500
C	-3.90090800	-5.57535400	-3.16111400
H	-3.89067100	-6.16605900	-4.07278000
H	-4.04662900	-7.27722600	-1.86602400
H	-3.79482700	-2.32567200	-2.15880300
H	-4.23972300	-7.15115000	0.57469400
H	-4.37996800	-5.79421900	2.64655600
Pd	2.42692800	-0.26978300	-1.04463000
C	2.59830700	0.77025700	2.11450100
P	1.29346600	0.45632200	0.86738300
C	5.18662900	-3.73382500	3.61359800
C	4.32726300	-4.82258900	3.78614800
C	3.37878700	-5.11561300	2.80268400
C	3.29388100	-4.32694000	1.65473900
C	4.16515500	-3.24583900	1.46515100
C	5.10648300	-2.94958800	2.46099000

H	5.91658800	-3.48858200	4.38067700
H	4.39238700	-5.43274700	4.68296200
H	2.69907500	-5.95412600	2.93259700
H	2.53792400	-4.53094400	0.90333700
H	5.75570900	-2.08609200	2.34856700
C	3.99919300	-2.34627700	0.26339800
N	2.87105000	-1.76769100	0.23516800
O	4.04430800	2.31300700	-1.41566300
C	2.88754400	2.49118700	-1.81427300
O	1.99536900	1.58401300	-2.03716900
C	2.37080400	3.89647000	-2.03096400
C	1.69015300	4.27337300	-3.19488600
C	2.51334400	4.84459500	-1.01896800
C	1.16444900	5.55584200	-3.34823100
C	1.96173900	6.11702400	-1.13586000
C	1.29667700	6.46738600	-2.30737200
F	3.12193000	4.54250800	0.14345100
F	2.04456600	6.98348000	-0.11243400
F	0.77094800	7.70233600	-2.40202900
C	5.13103700	-2.21930200	-0.78013100
C	6.28577000	-3.20870400	-0.52617200
H	6.84465700	-2.95859900	0.37942200
H	5.92213400	-4.23656100	-0.41989000
H	6.98704100	-3.17940700	-1.36863100
C	5.69885000	-0.78351800	-0.75529700
H	6.51703900	-0.68910900	-1.48084200
H	4.95025900	-0.01817600	-0.98153300
H	6.09852600	-0.55370000	0.23919700
C	4.55631900	-2.58510300	-2.19891200
H	4.52398900	-3.67456300	-2.27688900

H	5.30805900	-2.25685000	-2.92564600
C	3.21371200	-2.01699600	-2.63766700
C	3.20307200	-0.76474100	-3.20229300
H	2.33771300	-0.34238000	-3.69794200
H	4.12901100	-0.21128700	-3.32366700
C	1.98337100	-2.87032700	-2.57065200
C	0.76842600	-2.46184900	-3.16000000
C	1.98275200	-4.08350200	-1.85937100
C	-0.39140900	-3.21809500	-3.01852700
H	0.69492700	-1.53883700	-3.72414100
C	0.81823200	-4.83952000	-1.71374100
H	2.88885900	-4.43915200	-1.38458100
C	-0.37977800	-4.40879100	-2.28433400
H	-1.30148400	-2.86769600	-3.49108600
H	0.85166400	-5.76528100	-1.14524900
H	-1.28984900	-4.98948900	-2.17021400
H	1.56781800	3.54182300	-3.98561800
H	0.64414100	5.85834100	-4.25036500

TS2

S	-2.38367400	-0.10014800	-3.19563400
O	-1.50763000	-0.43955700	-4.39048200
N	-1.73528600	1.28291400	-2.34408900
C	3.79510700	1.53216800	1.68795900
H	3.85907200	1.92025700	0.67739900
C	4.86424700	1.75288300	2.55859700
C	4.79706700	1.21943700	3.85367100
H	5.62940400	1.37867100	4.53748600
C	3.68864300	0.48590000	4.28773900
C	2.61668000	0.29594800	3.40478800
H	1.75578600	-0.27395300	3.73845100

C	0.22156200	-0.67815700	1.67850900
C	0.26038600	-2.03000600	1.32070500
H	0.92322500	-2.35019300	0.52533200
C	-0.53324900	-2.96572300	1.99395500
C	-1.36973000	-2.51679000	3.02081100
H	-1.98404400	-3.23859900	3.55407700
C	-1.46151700	-1.16212600	3.36749600
C	-0.64854400	-0.24813900	2.69059500
H	-0.69081300	0.80544600	2.95441900
C	0.36649100	2.05030000	0.68622600
C	0.75839700	3.19931600	1.39062800
H	1.59565300	3.14455500	2.07716700
C	0.11809900	4.42299200	1.19652400
H	0.46397100	5.30480400	1.72839200
C	-0.93131800	4.51052700	0.28451500
C	-1.33560600	3.37408300	-0.41518500
H	-2.15350700	3.44396100	-1.12468200
C	-0.71063800	2.13317800	-0.23363000
C	-0.78828200	2.07995900	-3.15576300
H	-1.13471000	3.11527300	-3.23443500
H	-0.70912800	1.65080800	-4.15817900
H	0.20518600	2.06931700	-2.70947300
C	-3.94534600	0.67228500	-3.91337900
C	-3.59565300	1.86142000	-4.80714600
H	-4.49473900	2.19099900	-5.34218200
H	-2.83994200	1.57875700	-5.54713600
H	-3.22139000	2.70429700	-4.22056800
C	-4.58274700	-0.45729000	-4.73785900
H	-3.91185000	-0.78471500	-5.53790900
H	-5.51179600	-0.09249100	-5.19143400

H	-4.83558500	-1.32401400	-4.11573400
C	-4.83488900	1.06974900	-2.73254500
H	-5.79063500	1.45107700	-3.11214700
H	-4.36153400	1.84821100	-2.12942000
H	-5.04761900	0.21648000	-2.07769300
C	-2.40319800	0.25398500	-0.18616000
C	-2.57643600	-1.13471100	-0.18851400
H	-1.85186900	-1.76524400	-0.69294600
C	-3.67183800	-1.73083200	0.45740000
C	-4.55617700	-0.91329000	1.17604600
H	-5.38602100	-1.36688200	1.71042100
C	-4.38040300	0.47490600	1.22822700
C	-3.32438500	1.04165500	0.50369300
H	-3.22132500	2.11827400	0.50143400
C	-7.14382800	2.96071700	3.42573200
C	-5.75158100	3.16237900	3.62358800
C	-4.80699100	2.33406500	2.92610700
C	-5.30305100	1.33345200	2.01655700
C	-6.67162400	1.17182500	1.87137200
H	-7.04150400	0.42457300	1.17460000
C	-7.59421300	1.97782400	2.57518900
C	-3.88317000	-3.20449000	0.43792500
C	-3.88708600	-3.95980400	-0.78550900
C	-4.03241300	-5.38798400	-0.72347300
C	-4.20213700	-6.02185200	0.53577500
C	-4.26212000	-5.27278400	1.68854500
C	-4.10450100	-3.86974500	1.63324200
H	-4.12040500	-3.29846700	2.55548100
C	-1.25381200	0.90463600	-0.97246300
H	-0.44035600	0.16896600	-1.06119100

H	-1.43216200	5.45852100	0.11003700
C	6.07057500	2.52855800	2.08125600
H	6.80013000	1.86455700	1.59856700
H	5.77995700	3.27999600	1.34001000
H	6.58096700	3.03174400	2.90987000
C	3.63504400	-0.11680500	5.67248800
H	3.63059400	-1.21234800	5.61417800
H	4.49533700	0.18576700	6.27867900
H	2.72428100	0.18308800	6.20483400
C	-0.47331900	-4.42719800	1.61699600
H	-1.43972800	-4.91930200	1.76333000
H	-0.18643100	-4.55054600	0.56743500
H	0.26805900	-4.96028800	2.22773300
C	-2.45361400	-0.69582400	4.40658900
H	-3.42940000	-0.50175700	3.94270700
H	-2.60142200	-1.44860100	5.18896100
H	-2.13622500	0.23803000	4.88129900
C	-3.42174500	2.53469900	3.19393600
H	-2.68920100	1.89963600	2.71247300
C	-2.99296100	3.51735900	4.05874800
H	-1.92849200	3.65146700	4.23128300
C	-3.92684800	4.35070800	4.71949500
C	-5.27473900	4.16730000	4.51004000
H	-6.00188900	4.79116900	5.02469500
H	-3.57795100	5.12562500	5.39657100
H	-8.65969200	1.82593400	2.42595500
H	-7.84611000	3.59630100	3.95977500
C	-3.81345400	-3.35309800	-2.07007100
C	-3.85079000	-4.10588500	-3.22328500
H	-3.79553600	-3.61262500	-4.18980900

C	-4.04071100	-6.13877900	-1.93176800
C	-3.95198800	-5.51655800	-3.15698800
H	-3.96839900	-6.10190900	-4.07207900
H	-4.13877900	-7.22008600	-1.86939400
H	-3.73099600	-2.27656000	-2.13913000
H	-4.30586700	-7.10362300	0.57001600
H	-4.41382600	-5.75532300	2.65027200
Pd	2.38888500	-0.13522500	-1.26123500
C	2.66183700	0.81615300	2.10680900
P	1.35350500	0.50167400	0.85336500
C	5.03887700	-2.76831800	3.56362400
C	4.11173700	-3.71434700	4.00756600
C	3.18739200	-4.25394200	3.10814700
C	3.18637300	-3.84483200	1.77598700
C	4.12250000	-2.90362700	1.32259700
C	5.05181700	-2.36945700	2.22691400
H	5.74713500	-2.32893700	4.26028700
H	4.10681600	-4.02618400	5.04853100
H	2.46125000	-4.98795200	3.44735100
H	2.45990100	-4.24323500	1.07515700
H	5.75412800	-1.60943600	1.90089400
C	4.04685500	-2.40675000	-0.08637300
N	2.90608900	-2.00578200	-0.48319400
O	4.18982800	2.38463400	-1.44081400
C	3.02737200	2.62666000	-1.77929400
O	2.09257100	1.76993800	-2.03619800
C	2.53289400	4.05178900	-1.86605800
C	1.79784600	4.52334100	-2.96020700
C	2.73500100	4.91157700	-0.78755100
C	1.28931100	5.82138700	-2.98825500

C	2.20299400	6.19765900	-0.78093600
C	1.49028000	6.64756500	-1.88869900
F	3.37752600	4.50196100	0.32168100
F	2.34151600	6.97847300	0.30399500
F	0.98269200	7.89350600	-1.86199900
C	5.25988200	-2.43639900	-1.03303300
C	6.30194900	-3.50116200	-0.65376800
H	6.81137400	-3.25014600	0.28092500
H	5.83931000	-4.48688700	-0.52849900
H	7.06018000	-3.57685400	-1.44172000
C	5.93562600	-1.04527800	-1.07166400
H	6.73629400	-1.04138700	-1.82144100
H	5.23230300	-0.24106000	-1.30075800
H	6.38965700	-0.81183400	-0.10240400
C	4.59883600	-2.82032800	-2.39391700
H	4.55405900	-3.91035300	-2.46301100
H	5.23591700	-2.48027600	-3.21767700
C	3.20669500	-2.22780100	-2.56258100
C	3.14679200	-0.87398600	-3.06514800
H	2.35746800	-0.60087800	-3.76136700
H	4.09672100	-0.39452500	-3.29086000
C	2.00877600	-3.11971400	-2.67316100
C	0.79799500	-2.65380700	-3.22135400
C	2.00728600	-4.39844100	-2.08449600
C	-0.36225400	-3.42217700	-3.16501400
H	0.71853000	-1.67269500	-3.67391700
C	0.84708300	-5.17095200	-2.03341000
H	2.90726600	-4.78363100	-1.61658000
C	-0.35026000	-4.68262800	-2.56225000
H	-1.27401900	-3.01608000	-3.58849400

H	0.87679300	-6.14971400	-1.56151800
H	-1.26188000	-5.26914300	-2.50306300
H	1.62014000	3.85348500	-3.79404200
H	0.72805900	6.19966700	-3.83558100

TS2-ent

S	-6.73289100	-5.41836400	4.78798800
O	-5.87613100	-4.35061400	5.44258900
N	-7.20726800	-4.88044100	3.20963000
C	-8.32333300	-9.71164300	1.12169100
H	-8.01424200	-9.36156200	2.10043600
C	-8.20083600	-11.07125200	0.82481600
C	-8.59299100	-11.51890700	-0.44186100
H	-8.48909300	-12.57411200	-0.68763700
C	-9.11047600	-10.64031000	-1.39991400
C	-9.26771700	-9.29048100	-1.06090700
H	-9.71362400	-8.61709300	-1.78532900
C	-10.44883000	-6.37134600	-0.31800400
C	-11.66728300	-6.00530500	0.27567200
H	-11.83976300	-6.18135100	1.33292400
C	-12.67287000	-5.39070200	-0.47972300
C	-12.45464000	-5.17880900	-1.84750300
H	-13.23321100	-4.69824900	-2.43756400
C	-11.25368400	-5.53612200	-2.46484400
C	-10.25236300	-6.12065500	-1.68180900
H	-9.28909600	-6.32086300	-2.14020100
C	-9.66338800	-7.08490500	2.38138600
C	-10.48639600	-8.16654400	2.75259300
H	-10.71736100	-8.93749900	2.02630900
C	-11.01746500	-8.27186400	4.03483400
H	-11.64950700	-9.11773500	4.29082300

C	-10.73639600	-7.28173700	4.97783400
C	-9.95711500	-6.18907500	4.61100500
H	-9.77516000	-5.39764200	5.33002500
C	-9.41761700	-6.06084100	3.32037000
C	-6.38684600	-3.79270800	2.64429900
H	-6.07533100	-3.07168900	3.40759200
H	-6.97931000	-3.27951400	1.88858200
H	-5.49312300	-4.20428200	2.17336400
C	-5.53677100	-6.81340300	4.34394500
C	-6.32211200	-7.85397000	3.54666300
H	-5.70234700	-8.74649500	3.39981800
H	-6.58631900	-7.46632800	2.56056700
H	-7.23684700	-8.15852400	4.06993600
C	-4.33330900	-6.27715200	3.56971200
H	-4.58174700	-6.08695800	2.52415900
H	-3.52240200	-7.01652000	3.58632400
H	-3.95912000	-5.35613900	4.02798000
C	-5.10758100	-7.36004900	5.71419500
H	-4.42979200	-8.20972900	5.56813200
H	-5.96723500	-7.71254300	6.29755600
H	-4.58455000	-6.59384100	6.29455100
C	-9.39342300	-3.56241000	3.54571300
C	-10.71547400	-3.37120300	3.12440100
H	-11.14174100	-4.04798100	2.38900900
C	-11.50694700	-2.34505500	3.64446300
C	-10.91811900	-1.43028800	4.53284600
H	-11.50497700	-0.60161000	4.91834800
C	-9.58211100	-1.56577200	4.91535900
C	-8.83432100	-2.66253000	4.44844500
H	-7.81765300	-2.79084800	4.80397300

C	-7.68314600	1.26032100	7.56237300
C	-7.19096100	1.13020300	6.23560100
C	-7.83575400	0.22193300	5.33091400
C	-8.94105200	-0.56096500	5.80592300
C	-9.39857500	-0.38150700	7.09849000
H	-10.23803100	-0.97677300	7.44771300
C	-8.77327500	0.53175000	7.97965900
C	-12.93132900	-2.21299800	3.24152100
C	-13.84522200	-3.31862600	3.35126000
C	-15.19752800	-3.14512800	2.89873100
C	-15.60244500	-1.89311700	2.36199400
C	-14.71481900	-0.84471500	2.28845100
C	-13.38470200	-1.00610000	2.73778500
H	-12.68953000	-0.17954800	2.64705300
C	-8.66396100	-4.77307000	2.96546900
H	-8.73608100	-4.64039400	1.88601500
H	-11.13508900	-7.34893100	5.98627200
C	-7.68060600	-12.02718400	1.87189300
H	-6.82652200	-11.59838600	2.40521900
H	-8.45750400	-12.25399900	2.61390700
H	-7.35677300	-12.97305900	1.42724500
C	-9.48159900	-11.12541300	-2.78270100
H	-9.70028100	-12.19837300	-2.78879200
H	-10.35847600	-10.59597600	-3.17102300
H	-8.66069800	-10.95390500	-3.49249700
C	-13.94723500	-4.91123200	0.17043400
H	-13.89227400	-3.83500400	0.37628200
H	-14.81546400	-5.07416700	-0.47844300
H	-14.13288500	-5.40982300	1.12485200
C	-10.98217800	-5.23138900	-3.91810000

H	-10.67593400	-6.13084800	-4.46633800
H	-11.86355700	-4.81549200	-4.41736100
H	-10.16087100	-4.50832200	-3.99083300
C	-7.37317200	0.15968800	3.98544900
H	-7.89086900	-0.47333000	3.27439300
C	-6.30113400	0.92010800	3.57195300
H	-5.96728800	0.86268300	2.53918700
C	-5.63889800	1.78516500	4.47678800
C	-6.08196200	1.89196900	5.77592000
H	-5.59203300	2.56928600	6.47187800
H	-4.79048900	2.37431500	4.13860900
H	-9.15016500	0.64033700	8.99311600
H	-7.18672000	1.95134200	8.23976600
C	-13.48257100	-4.57964700	3.90573000
C	-14.38678100	-5.61736200	3.97633500
H	-14.07536500	-6.57002300	4.39649800
C	-16.10537100	-4.23590600	2.98872400
C	-15.71230200	-5.44885700	3.50829300
H	-16.41681700	-6.27421600	3.56564800
H	-17.12399500	-4.09257000	2.63554100
H	-12.47777700	-4.72756000	4.28310100
H	-16.62507600	-1.78073500	2.00956000
H	-15.02036000	0.10957100	1.86912600
Pd	-7.08281100	-5.67019000	-0.12439700
C	-8.88434900	-8.81554900	0.19982100
P	-9.01759800	-7.02695200	0.64161500
C	-5.02486700	-11.82767000	-0.27365000
C	-4.31992100	-11.86921800	0.93126900
C	-3.96396100	-10.67581900	1.56647500
C	-4.29574400	-9.45146900	0.99035700

C	-4.97724400	-9.40036000	-0.23937000
C	-5.35131100	-10.60409800	-0.85636500
H	-5.33370600	-12.75014500	-0.75714600
H	-4.06035200	-12.82494200	1.37815400
H	-3.42248200	-10.69834500	2.50847300
H	-4.01734300	-8.51939700	1.46862700
H	-5.93478700	-10.58865800	-1.76919200
C	-5.24643700	-8.06363800	-0.83934100
N	-5.52530600	-7.09600100	-0.04605500
O	-8.52115100	-3.63890100	-2.26176500
C	-8.77483500	-3.39431900	-1.07891400
O	-8.34031400	-4.02133600	-0.03581900
C	-9.73863000	-2.28298800	-0.72154500
C	-9.60277500	-1.50522400	0.43100600
C	-10.84368400	-2.04236200	-1.54759400
C	-10.53861900	-0.52819800	0.75850800
C	-11.80313200	-1.08363300	-1.22691300
C	-11.63896400	-0.33078500	-0.07101500
F	-12.53714200	0.61266900	0.26966800
F	-10.38529900	0.20965100	1.87142400
F	-8.54587800	-1.63452600	1.25522000
C	-5.04994600	-7.78987400	-2.34691100
C	-4.11396500	-8.76474600	-3.08262400
H	-4.56143900	-9.74987500	-3.22958900
H	-3.16967200	-8.90091700	-2.54371400
H	-3.88178800	-8.35825200	-4.07364700
C	-6.43977300	-7.78633900	-3.02420100
H	-6.33254700	-7.61327700	-4.10170400
H	-7.06721900	-6.99442200	-2.60316100
H	-6.96120100	-8.73832900	-2.87602000

C	-4.42268100	-6.35990500	-2.37076700
H	-3.35095300	-6.43660700	-2.57576500
H	-4.86095900	-5.78398600	-3.19082000
C	-4.62661200	-5.60662400	-1.05587100
C	-5.61790200	-4.55097100	-1.05636200
H	-5.48698800	-3.69568900	-0.39958100
H	-6.07829700	-4.29397200	-2.00798400
C	-3.44201700	-5.50665300	-0.13702300
C	-2.54616200	-6.58545000	-0.01148800
C	-3.18663700	-4.35949400	0.63271200
C	-1.46045300	-6.53159000	0.85818900
H	-2.70913800	-7.49428100	-0.58012100
C	-2.10033000	-4.30170400	1.50677200
H	-3.82647400	-3.48975900	0.55042200
C	-1.23589800	-5.38911400	1.63097300
H	-0.79478700	-7.38666400	0.93766800
H	-1.93487000	-3.40168800	2.09197600
H	-0.39486200	-5.34640300	2.31714000
H	-10.94250200	-2.63911200	-2.44580300
H	-12.67094300	-0.90637700	-1.85244400

Int3

S	-2.48033900	-0.02152000	-3.18979000
O	-1.78789300	-0.38969500	-4.48607800
N	-1.56230900	1.22056800	-2.36484900
C	3.75767100	1.89761400	1.98483800
H	3.94816900	2.06718700	0.93029200
C	4.65395200	2.42563100	2.92267600
C	4.41606200	2.19425900	4.28211700
H	5.10717000	2.59763200	5.02036900
C	3.30723000	1.45449000	4.71731500

C	2.40864000	0.96388500	3.76400400
H	1.54218800	0.40259800	4.09774400
C	0.44182500	-0.63005000	1.96906700
C	0.80252800	-1.97722100	1.87276300
H	1.62021900	-2.26669200	1.22365100
C	0.13790400	-2.95244300	2.62470100
C	-0.89231100	-2.54544800	3.47925600
H	-1.40134200	-3.29056600	4.08889200
C	-1.29389700	-1.20537000	3.57524500
C	-0.60910900	-0.25276600	2.81511000
H	-0.87995300	0.79494200	2.89624600
C	0.36863700	2.06383200	0.78140500
C	0.59506100	3.23397400	1.52665000
H	1.35544500	3.23790400	2.29744100
C	-0.10905800	4.41176800	1.27815900
H	0.11627000	5.30673200	1.85097500
C	-1.06210900	4.43657800	0.26425400
C	-1.30711100	3.28149900	-0.47629800
H	-2.05333000	3.29407100	-1.26288100
C	-0.61670700	2.08531700	-0.24031400
C	-0.58589800	1.91833600	-3.22399600
H	-0.79895100	2.99212800	-3.25366800
H	-0.63208000	1.51208300	-4.23785800
H	0.42436300	1.77950700	-2.83825700
C	-3.97787700	1.00573400	-3.72188100
C	-3.53636700	2.15725500	-4.62350500
H	-4.41812200	2.63015600	-5.07332300
H	-2.89389100	1.78843200	-5.42951300
H	-2.99464700	2.91964400	-4.05772700
C	-4.85484900	0.01234400	-4.49936500

H	-4.31846200	-0.39409300	-5.36196400
H	-5.75515800	0.52520700	-4.85820200
H	-5.17909800	-0.81968400	-3.86309700
C	-4.69169100	1.49311400	-2.46067900
H	-5.62222000	2.00097800	-2.74301600
H	-4.07033200	2.19650100	-1.90184500
H	-4.94954300	0.66471700	-1.79082900
C	-2.09327200	0.05317700	-0.23595300
C	-2.11136600	-1.34316400	-0.27717300
H	-1.35141200	-1.87769700	-0.83800600
C	-3.13489500	-2.06231600	0.35268400
C	-4.09623900	-1.37318100	1.10037200
H	-4.86565400	-1.93596100	1.62146700
C	-4.08674700	0.02882500	1.17473500
C	-3.09595500	0.72144100	0.46607600
H	-3.11895400	1.80312000	0.46005500
C	-7.25514200	2.10268300	3.26023300
C	-5.91984200	2.54119800	3.46479200
C	-4.83770900	1.86290200	2.80530200
C	-5.14067700	0.75882400	1.92989300
C	-6.46322800	0.37139000	1.77323100
H	-6.69478900	-0.44651700	1.09677400
C	-7.51970800	1.03347200	2.43666600
C	-3.23653200	-3.54045200	0.16755800
C	-3.72241500	-4.06938900	-1.07474800
C	-3.76049300	-5.49239300	-1.25773200
C	-3.33199500	-6.34277800	-0.20278300
C	-2.90068800	-5.81140500	0.99089600
C	-2.85924400	-4.40808800	1.17357200
H	-2.50038500	-3.99751800	2.10974900

C	-1.03534300	0.83249500	-1.02159100
H	-0.15224300	0.18582400	-1.14547600
H	-1.61101700	5.34768200	0.04314300
C	5.84152800	3.22655800	2.44195700
H	6.59341300	2.57584700	1.97770100
H	5.53110200	3.94894100	1.67929400
H	6.32489600	3.76728500	3.26228400
C	3.09893600	1.16598800	6.18648500
H	3.62059400	0.24593300	6.48325300
H	3.48528600	1.97610900	6.81465200
H	2.03825100	1.02716700	6.42148200
C	0.51228900	-4.40828500	2.47908000
H	0.21315500	-4.99250200	3.35607900
H	0.01200300	-4.84890900	1.60625600
H	1.59230800	-4.52727400	2.33990900
C	-2.45229500	-0.80875900	4.45908100
H	-3.39905800	-0.90065900	3.91255000
H	-2.51730900	-1.44795200	5.34644100
H	-2.37487900	0.23242700	4.78634200
C	-3.51417400	2.31455500	3.07517300
H	-2.67803200	1.80531500	2.61726700
C	-3.27146300	3.38490500	3.90744700
H	-2.24800800	3.70799600	4.07758000
C	-4.34324500	4.06321900	4.53477000
C	-5.63559400	3.64145300	4.32046000
H	-6.46714700	4.14642900	4.80655700
H	-4.14303900	4.90835300	5.18763500
H	-8.54187100	0.70111400	2.27777200
H	-8.06254800	2.62812200	3.76454000
C	-4.16271600	-3.23811300	-2.14176800

C	-4.61003500	-3.77997600	-3.32661400
H	-4.92751100	-3.12721400	-4.13436600
C	-4.22203900	-6.01848100	-2.49550100
C	-4.63951100	-5.18403200	-3.50866700
H	-4.98869600	-5.59937900	-4.44999200
H	-4.24115700	-7.09781400	-2.62708800
H	-4.12399400	-2.16253000	-2.01822200
H	-3.35644700	-7.41927400	-0.35409800
H	-2.58065800	-6.46404800	1.79868000
Pd	2.54273800	-0.14783000	-1.02413100
C	2.62491200	1.18544500	2.39645100
P	1.47121600	0.60083600	1.08237200
C	4.88332100	-1.35905600	2.95620700
C	4.93685400	-2.45636300	3.81795300
C	4.68660300	-3.74061700	3.32647300
C	4.38126000	-3.92879300	1.97908700
C	4.29895600	-2.82732600	1.10874500
C	4.56509800	-1.54244600	1.61210500
H	5.07558400	-0.35718700	3.32495800
H	5.17553100	-2.31261400	4.86823300
H	4.72697900	-4.59819500	3.99218200
H	4.17860500	-4.92882000	1.61417900
H	4.52608000	-0.68792200	0.94568100
C	3.91571500	-2.98305200	-0.30769000
N	3.13236100	-2.14027400	-0.88398900
O	4.23319500	2.52428800	-1.10285100
C	3.07995800	2.67404900	-1.51444800
O	2.21565200	1.75037500	-1.78153900
C	2.50257100	4.05935000	-1.69543200
C	1.83247800	4.44450900	-2.86239400

C	2.56610600	4.96939200	-0.64149100
C	1.24946300	5.70550600	-2.98290900
C	1.95728300	6.21786700	-0.72813000
C	1.30911200	6.58099000	-1.90512200
F	3.14850200	4.64527500	0.52794000
F	1.96442700	7.04608600	0.33017400
F	0.72565600	7.79227000	-1.96839800
C	4.45316800	-4.05852700	-1.27317100
C	3.73761400	-5.42011300	-1.12094800
H	3.93386200	-5.88318800	-0.14952800
H	2.65716700	-5.32682200	-1.25064500
H	4.11174400	-6.10502300	-1.89026900
C	5.96942500	-4.24278700	-1.08458700
H	6.36100500	-4.90445200	-1.86539100
H	6.49670900	-3.28468300	-1.15247100
H	6.20040000	-4.68763100	-0.11114000
C	4.11944200	-3.40575900	-2.64897800
H	3.83086000	-4.14492800	-3.40029200
H	4.99707800	-2.86699900	-3.02240700
C	2.98597700	-2.38704200	-2.34502500
C	3.16466700	-0.93649400	-2.82835400
H	2.50473500	-0.59737100	-3.62649100
H	4.20752600	-0.66544300	-3.01149400
C	1.59228000	-2.94626100	-2.64268300
C	1.08876600	-2.90355100	-3.94905500
C	0.81271200	-3.55034600	-1.64712200
C	-0.15781200	-3.45311400	-4.25192300
H	1.67036600	-2.43039000	-4.73542800
C	-0.41952300	-4.12956700	-1.95433600
H	1.15967300	-3.56336900	-0.62067500

C	-0.91335900	-4.07949100	-3.25846000
H	-0.54340100	-3.38078200	-5.26430700
H	-0.99883800	-4.60345100	-1.16877400
H	-1.88042700	-4.51165100	-3.49147100
H	1.76391000	3.73574000	-3.67974300
H	0.73722100	6.01668100	-3.88671300

Int3-ent

S	1.64009300	0.16843800	3.66197500
O	2.30277600	1.37771100	4.29705000
N	1.32405100	0.48007900	1.99969800
C	1.26878900	-4.31775500	-0.48182300
H	1.97259600	-3.67354000	0.03665000
C	1.56767100	-5.67009900	-0.64757000
C	0.63166900	-6.48350500	-1.30197200
H	0.84991200	-7.54158700	-1.43495800
C	-0.57040800	-5.96964400	-1.79825000
C	-0.84233400	-4.60424700	-1.62785400
H	-1.77351400	-4.19072900	-2.00506600
C	-1.78725600	-1.55244800	-1.48353500
C	-3.03077600	-1.48238200	-0.83396600
H	-3.11208200	-1.71260300	0.22368200
C	-4.18141800	-1.10178900	-1.53509600
C	-4.07224300	-0.80652000	-2.90080100
H	-4.96323400	-0.50029600	-3.44690000
C	-2.84920500	-0.87683300	-3.57204300
C	-1.71083100	-1.23946100	-2.84500700
H	-0.75020000	-1.22805000	-3.33903600
C	-0.73140000	-2.03224600	1.14682700
C	-1.27355100	-3.23404300	1.64222200
H	-1.27817600	-4.11904400	1.01686300

C	-1.83072700	-3.31221600	2.91613100
H	-2.24399700	-4.25354600	3.26796600
C	-1.87301300	-2.17213100	3.71857100
C	-1.33146800	-0.98173600	3.24284600
H	-1.36594000	-0.09460500	3.86581200
C	-0.73573700	-0.88975200	1.97418800
C	2.20456900	1.45113400	1.33004600
H	2.72420100	2.06281500	2.07457300
H	1.61140900	2.09934600	0.68893800
H	2.93938900	0.93316500	0.70755100
C	3.01923100	-1.11927900	3.51644500
C	2.53104400	-2.23404100	2.59147600
H	3.27939800	-3.03487100	2.56441200
H	2.39107300	-1.85917100	1.57411500
H	1.58154000	-2.66233100	2.93279500
C	4.30582000	-0.45707600	3.02479100
H	4.29401200	-0.29428800	1.94486200
H	5.15915500	-1.10594600	3.24581100
H	4.45654700	0.50165400	3.53171600
C	3.19037400	-1.62431000	4.95845100
H	3.96842900	-2.39676700	4.98311300
H	2.26384500	-2.06603900	5.34491200
H	3.49294600	-0.81060500	5.62564200
C	-0.96540200	1.62389800	1.97783900
C	-2.29191000	1.62823500	1.52920000
H	-2.64469500	0.82338200	0.88935000
C	-3.18331900	2.63356600	1.91268900
C	-2.70927100	3.69171800	2.70217300
H	-3.38601800	4.49172400	2.98771600
C	-1.37706500	3.73076800	3.12114600

C	-0.51492100	2.67553200	2.77353500
H	0.50267200	2.68737300	3.14847900
C	0.05791100	6.91671100	5.65316400
C	0.67523100	6.72691300	4.38760700
C	0.19184800	5.69581200	3.51371800
C	-0.88508600	4.85808100	3.95925600
C	-1.46841500	5.09845200	5.19090000
H	-2.28420500	4.46051100	5.52024600
C	-1.00146600	6.12857300	6.03993700
C	-4.60680200	2.58763700	1.48731400
C	-5.44525900	1.46772000	1.81540400
C	-6.80887000	1.46935900	1.36474900
C	-7.29306200	2.56750800	0.60324500
C	-6.47349500	3.63540900	0.31718900
C	-5.13484600	3.64701900	0.77089400
H	-4.49575400	4.48829900	0.52815900
C	-0.09095900	0.43924900	1.56644900
H	-0.03332100	0.47929300	0.47656500
H	-2.31867400	-2.20710300	4.70888200
C	2.89199500	-6.22294500	-0.17336100
H	3.23815700	-5.71051600	0.73133700
H	2.83121200	-7.29458600	0.04361000
H	3.66881900	-6.08695500	-0.93889300
C	-1.55815600	-6.85056400	-2.52478300
H	-1.30345700	-7.91083500	-2.42678400
H	-2.57635200	-6.70843900	-2.14437100
H	-1.57467400	-6.60605400	-3.59420300
C	-5.51920500	-0.99857000	-0.84406500
H	-5.91583100	0.02092300	-0.90431800
H	-6.25825600	-1.66250400	-1.31020300

H	-5.45057400	-1.25582700	0.21505400
C	-2.73471100	-0.56505600	-5.04603300
H	-2.88013600	-1.46755100	-5.65553300
H	-3.48988100	0.16225300	-5.36427400
H	-1.74407800	-0.16084900	-5.28036400
C	0.77841300	5.57086400	2.22300100
H	0.38177400	4.83937900	1.53047800
C	1.81614700	6.38991600	1.83527000
H	2.24677200	6.27942100	0.84351800
C	2.31895500	7.38051800	2.71338400
C	1.75331400	7.54793400	3.95732800
H	2.12026300	8.31931600	4.63078500
H	3.14221600	8.01587200	2.39735700
H	-1.47500700	6.28087200	7.00615200
H	0.43382900	7.69997900	6.30737800
C	-4.99767500	0.36451600	2.59749800
C	-5.83928600	-0.68349100	2.90137200
H	-5.46955300	-1.51330900	3.49799600
C	-7.64860300	0.36938300	1.69281300
C	-7.17886100	-0.68587600	2.44192000
H	-7.83331300	-1.51869200	2.68480100
H	-8.67779200	0.37960300	1.34115700
H	-3.97742000	0.35228000	2.96335100
H	-8.32354900	2.54999200	0.25612800
H	-6.84344600	4.47368200	-0.26666400
Pd	1.76567000	-0.75579900	-1.54854100
C	0.06890300	-3.77789200	-0.96788100
P	-0.21033300	-1.99134200	-0.64050000
C	-0.02815600	-3.50322600	-5.09223700
C	-0.03528100	-4.88558000	-5.28816600

C	1.00853700	-5.66549800	-4.78314100
C	2.06402500	-5.06783200	-4.09657600
C	2.07321300	-3.68108400	-3.89218000
C	1.02162800	-2.90150200	-4.39962200
H	-0.83561600	-2.88947200	-5.48001100
H	-0.85048000	-5.35488400	-5.83212600
H	0.99787600	-6.74321800	-4.91824400
H	2.86513200	-5.68123900	-3.69807600
H	1.05056400	-1.82385900	-4.26829200
C	3.22035500	-2.97074100	-3.29332700
N	3.11189700	-2.15083700	-2.30920900
O	0.30910900	0.96709700	-3.61184800
C	0.13098400	1.42303800	-2.47493800
O	0.71724000	1.04274900	-1.38458800
C	-0.92922100	2.48452200	-2.26770400
C	-0.94603800	3.37857200	-1.19417600
C	-1.99605900	2.54179400	-3.17598500
C	-1.99309000	4.28053100	-1.01821100
C	-3.05623300	3.42956900	-3.01547300
C	-3.04393600	4.29569600	-1.93003200
F	-4.04685400	5.17358900	-1.73704900
F	-1.99446900	5.12376400	0.02741700
F	0.05639500	3.43776900	-0.30011100
C	4.62127200	-2.97222800	-3.93027700
C	5.46678600	-4.15035100	-3.39853100
H	5.03045100	-5.11330000	-3.68488600
H	5.56305000	-4.12110700	-2.31023900
H	6.47295500	-4.10110200	-3.83025400
C	4.54671300	-3.03647300	-5.46485000
H	5.54862300	-2.90248500	-5.88791500

H	3.89841000	-2.24879100	-5.86411400
H	4.15617600	-4.00069500	-5.80563200
C	5.15491400	-1.59852700	-3.43880900
H	6.22960300	-1.61497400	-3.24189700
H	4.96144100	-0.83802600	-4.20285600
C	4.31366800	-1.27555700	-2.16793400
C	3.57027000	0.07594700	-2.13729200
H	3.90972600	0.79697300	-1.39244400
H	3.45406700	0.52943500	-3.12370100
C	5.09366900	-1.58513200	-0.89563300
C	4.87821800	-2.75483100	-0.15894400
C	6.13287100	-0.72803600	-0.50627500
C	5.70363000	-3.08199700	0.91959200
H	4.07094400	-3.42105100	-0.44016000
C	6.95581700	-1.05018200	0.57284200
H	6.30787300	0.18798400	-1.06506700
C	6.75159700	-2.23661200	1.28329200
H	5.52343600	-4.00009100	1.47306500
H	7.75913400	-0.37609300	0.85711500
H	7.39665500	-2.49043600	2.11983500
H	-1.97164400	1.86096900	-4.01628500
H	-3.88857200	3.46359100	-3.70962600

Int4

S	2.40072100	-0.80997400	2.95838800
O	2.67707300	-1.88877500	3.98960400
N	1.25405200	0.33782500	3.59232900
C	1.37947600	-4.06300300	1.58514200
H	1.54869600	-3.34793200	2.38356600
C	2.10125300	-5.25639200	1.60493500
C	1.87596800	-6.19235200	0.58504200

H	2.43710400	-7.12549900	0.58581200
C	0.94725600	-5.95600200	-0.43325300
C	0.22408000	-4.75217700	-0.42665000
H	-0.51207300	-4.57402400	-1.20360800
C	-1.88607400	-2.20014400	-0.38866300
C	-3.14432900	-1.90394400	0.14898200
H	-3.26682900	-1.76728500	1.21846400
C	-4.26091400	-1.74076200	-0.68064300
C	-4.10153300	-1.92096900	-2.05802400
H	-4.96309800	-1.79843100	-2.71255800
C	-2.85729900	-2.23529500	-2.62296800
C	-1.74725900	-2.34880600	-1.77921000
H	-0.76312400	-2.51097100	-2.21298600
C	-0.98701400	-2.13423100	2.36451600
C	-1.70002200	-3.27468700	2.78530100
H	-1.80320900	-4.11558900	2.10589300
C	-2.28655400	-3.33870000	4.04359700
H	-2.83562100	-4.22671400	4.34368200
C	-2.17716300	-2.24549900	4.90788900
C	-1.46592300	-1.11961100	4.50851400
H	-1.41379800	-0.26243200	5.17103600
C	-0.84126900	-1.04913600	3.24988200
C	1.21293800	0.42180200	5.05655600
H	0.99237200	-0.53967300	5.53389000
H	0.44729300	1.15245000	5.33122800
H	2.17279100	0.77919500	5.42877300
C	3.97745100	0.22468400	2.85330700
C	3.64515200	1.51845600	2.10857500
H	4.57801200	2.03684600	1.85407200
H	3.03389800	2.18395000	2.72324300

H	3.10049400	1.32156800	1.17957000
C	4.57836000	0.46692800	4.23859100
H	4.06474600	1.27328200	4.77010000
H	5.63039600	0.75842200	4.13275700
H	4.53223700	-0.44588400	4.84125600
C	4.89747900	-0.68344900	2.01975400
H	5.84463200	-0.16459400	1.83277100
H	4.45850200	-0.93338500	1.04957300
H	5.11726700	-1.61766700	2.54600800
C	-0.87903200	1.46821500	2.93119500
C	-2.23745200	1.38342900	2.61872800
H	-2.67736700	0.41280900	2.42508100
C	-3.03423200	2.52630500	2.47163200
C	-2.43250700	3.78044600	2.64206200
H	-3.02813300	4.68221400	2.53564500
C	-1.06655200	3.89191200	2.93797600
C	-0.30033500	2.72880100	3.09712700
H	0.76163600	2.80818700	3.30247400
C	0.81612900	7.75185100	3.35791900
C	0.22500200	7.41785300	2.10973000
C	-0.39562500	6.13387400	1.94264400
C	-0.41756800	5.22606100	3.05526200
C	0.19467300	5.58873600	4.24197600
H	0.17980100	4.89184000	5.07574200
C	0.81164800	6.85153800	4.39827800
C	-4.44688200	2.40273400	2.02473300
C	-5.35846500	1.44475800	2.59349000
C	-6.67148100	1.31661100	2.02373700
C	-7.05451100	2.15355000	0.94186500
C	-6.17660400	3.08263800	0.43378500

C	-4.87609300	3.19767100	0.97258200
H	-4.17943400	3.90089700	0.52513000
C	-0.04252000	0.19848800	2.87841700
H	0.24997400	0.10274000	1.82554300
H	-2.64744400	-2.26974800	5.88718500
C	3.09821700	-5.52050600	2.70941000
H	3.26972200	-4.61716900	3.30281800
H	2.74037200	-6.30846500	3.38476700
H	4.06156300	-5.85479300	2.30568200
C	0.73443700	-6.96759400	-1.53620900
H	1.06221700	-7.96734300	-1.23331600
H	-0.32179900	-7.02930700	-1.82210000
H	1.30009800	-6.69550200	-2.43687800
C	-5.59159200	-1.32448400	-0.09986200
H	-6.42515100	-1.84031700	-0.59046900
H	-5.64818000	-1.52511100	0.97452400
H	-5.75158400	-0.24712000	-0.23066100
C	-2.71974900	-2.43025000	-4.11439600
H	-1.67540500	-2.39530400	-4.43205700
H	-3.11602600	-3.40569000	-4.42277500
H	-3.27470500	-1.66245800	-4.66637800
C	-0.92548800	5.80335200	0.66266600
H	-1.35686600	4.82103000	0.50679100
C	-0.87433700	6.70019200	-0.38202000
H	-1.27814800	6.42199300	-1.35177100
C	-0.28978600	7.97769600	-0.20480900
C	0.25013300	8.32309000	1.01337400
H	0.71513600	9.29613200	1.15408600
H	-0.25917200	8.67789600	-1.03516500
H	1.27177000	7.10827400	5.34854200

H	1.27994000	8.72864300	3.47298400
C	-5.03448600	0.61546100	3.70556900
C	-5.92314000	-0.32287800	4.18369400
H	-5.64129600	-0.94876800	5.02607800
C	-7.56687600	0.34351300	2.54595800
C	-7.20056500	-0.47139300	3.59277800
H	-7.89218100	-1.21663400	3.97597000
H	-8.55261900	0.25267500	2.09579200
H	-4.07178600	0.72560900	4.19012800
H	-8.05057600	2.04226900	0.52028800
H	-6.46812200	3.71820100	-0.39768700
Pd	1.29866800	-0.70874500	-0.38335600
C	0.43649500	-3.80237000	0.57653600
P	-0.35460900	-2.13435400	0.62145000
C	-0.16312100	-5.01147800	-4.59301500
C	-0.69051800	-4.77614900	-5.86579100
C	-0.28649800	-3.64701200	-6.58006200
C	0.65163300	-2.76660400	-6.03608900
C	1.19950200	-3.00004700	-4.76469200
C	0.77051900	-4.13196900	-4.04788100
H	-0.47781900	-5.88248100	-4.02578100
H	-1.41665400	-5.46272000	-6.29261600
H	-0.70585000	-3.44386200	-7.56182900
H	0.92908800	-1.87884400	-6.58868800
H	1.18334900	-4.30807700	-3.06039100
C	2.21463800	-2.10853100	-4.14275200
N	2.16701000	-1.88471800	-2.88268800
C	3.47949600	-1.58595200	-4.84809500
C	4.44208500	-2.78537900	-5.01620300
H	4.00445700	-3.54220800	-5.67784200

H	4.66549900	-3.25694900	-4.05423100
H	5.38654700	-2.44897600	-5.46071100
C	3.29220400	-0.88960500	-6.20403000
H	4.23431300	-0.40639500	-6.49150100
H	2.51868700	-0.11666700	-6.15507800
H	3.03733700	-1.60010600	-6.99730100
C	3.97356300	-0.59105500	-3.77576000
H	5.06366100	-0.51480900	-3.75470500
H	3.57204200	0.40187400	-4.00350400
C	3.34423700	-1.08690900	-2.43684200
C	2.88769000	0.07445200	-1.51522700
H	3.66862600	0.32107500	-0.78646300
H	2.72127700	0.96855900	-2.11710500
C	4.28522700	-2.00829200	-1.64311100
C	3.84899500	-3.22546400	-1.10117600
C	5.60774200	-1.60862000	-1.39225400
C	4.71806100	-4.02666200	-0.35380700
H	2.82664900	-3.54848100	-1.26229700
C	6.47439300	-2.40466100	-0.64434900
H	5.96181000	-0.65580400	-1.77617800
C	6.03297100	-3.62398200	-0.12311300
H	4.35516700	-4.96823900	0.04415800
H	7.49380000	-2.07092400	-0.46694400
H	6.70587500	-4.24904500	0.45808700
Si	0.26260500	1.19686700	-1.22755900
C	1.01805200	2.77687900	-0.49017100
H	2.09652900	2.84316100	-0.67040600
H	0.54479800	3.67591600	-0.90617200
H	0.85936600	2.79319500	0.59348000
C	-1.59752600	1.21544400	-0.82820500

H	-2.00662200	2.19435400	-1.11615600
H	-2.14415400	0.44396600	-1.37746400
H	-1.79422600	1.07419600	0.23547000
C	0.32181600	1.33039600	-3.12362200
C	1.08250600	2.30283500	-3.79796700
C	-0.43166900	0.44067900	-3.91013000
C	1.09089100	2.38462600	-5.19398900
H	1.67596600	3.01418600	-3.22724600
C	-0.43434300	0.51563800	-5.30333400
H	-1.02671900	-0.32394700	-3.42236200
C	0.32912200	1.49138200	-5.95180100
H	1.68736000	3.14731700	-5.68941700
H	-1.02636900	-0.18991400	-5.88073300
H	0.33021900	1.55543900	-7.03732200

Int4-ent

S	1.44773800	1.72895900	4.71706600
O	2.01975000	3.11742100	4.58021900
N	1.63090600	0.85574100	3.24371700
C	0.66318600	-4.22192000	0.07345800
H	1.22302700	-3.95912100	0.97261000
C	0.87343100	-5.47124200	-0.51452900
C	0.15587700	-5.79321500	-1.66879600
H	0.30932000	-6.76847500	-2.13622700
C	-0.74779900	-4.89507600	-2.24609900
C	-0.93152900	-3.64910100	-1.64320200
H	-1.62518900	-2.93716200	-2.09116300
C	-2.02865500	-1.08369700	-0.23582100
C	-3.12613400	-1.86827900	0.13803500
H	-2.96875700	-2.82623100	0.63499200
C	-4.43151400	-1.43961800	-0.10478700

C	-4.62059500	-0.20850700	-0.73948300
H	-5.63856700	0.14611500	-0.91271700
C	-3.54757900	0.58998800	-1.13447400
C	-2.25181300	0.13780900	-0.86793800
H	-1.40419100	0.75112600	-1.15568600
C	-0.50319800	-1.84627500	2.00664100
C	-1.10003600	-3.02396100	2.49021200
H	-1.30174100	-3.83983600	1.79813200
C	-1.45116900	-3.17141500	3.82523800
H	-1.92346400	-4.09231500	4.16312800
C	-1.20537000	-2.13116100	4.71845400
C	-0.58651600	-0.97552100	4.26232100
H	-0.41091900	-0.14975400	4.95214000
C	-0.20951800	-0.81518600	2.92337300
C	2.79013300	1.21219600	2.42445700
H	3.31157500	2.06585800	2.87224000
H	2.47494400	1.50267200	1.41290700
H	3.48937200	0.36699600	2.32627600
C	2.71822600	0.86038300	5.82243200
C	2.41391100	-0.62236500	5.86224200
H	3.13868000	-1.12988000	6.51333500
H	2.48478700	-1.06960200	4.86483300
H	1.41279000	-0.82981400	6.26181200
C	4.12329200	1.14422200	5.32467000
H	4.37930700	0.53684600	4.44987600
H	4.84576400	0.90823200	6.11689700
H	4.23599700	2.20492100	5.06985900
C	2.49317300	1.51685600	7.18050900
H	3.16473600	1.06659900	7.92237100
H	1.46382000	1.37697500	7.53531900

H	2.70055900	2.59170000	7.14056400
C	-0.65502700	1.61621900	2.44580900
C	-2.00919000	1.29718100	2.33823200
H	-2.31681300	0.25490600	2.31109500
C	-2.99207100	2.29040600	2.23116200
C	-2.57690200	3.62529000	2.18610300
H	-3.32574800	4.41512500	2.13193400
C	-1.22112700	3.97260100	2.22700300
C	-0.27177400	2.95735100	2.37776600
H	0.78171300	3.22908900	2.41902400
C	0.03677700	8.08128400	2.11312300
C	-0.87079900	7.63051600	1.12582500
C	-1.27785200	6.25554500	1.11663200
C	-0.78198700	5.38010100	2.13685500
C	0.13764200	5.86119000	3.05469900
H	0.53460200	5.18055500	3.80862700
C	0.54692300	7.20802100	3.04429900
C	-4.42145500	1.94800900	2.07948100
C	-5.05428300	0.87904900	2.79919300
C	-6.41752200	0.55047500	2.49104100
C	-7.12173900	1.30270500	1.52114300
C	-6.51497700	2.35835100	0.88457700
C	-5.17289200	2.66901300	1.16358800
H	-4.69099000	3.47392400	0.60879800
C	0.39282600	0.51448100	2.49870000
H	0.76151700	0.38516400	1.46474800
H	-1.49566400	-2.21735600	5.76484500
C	1.89765300	-6.40664700	0.05346500
H	2.89284500	-6.19755300	-0.36873400
H	1.98812000	-6.29850900	1.14089700

H	1.66886900	-7.45437200	-0.17219800
C	-1.48711400	-5.25565900	-3.49985600
H	-0.80570900	-5.33170900	-4.35879600
H	-1.98857400	-6.22737400	-3.41181300
H	-2.24540900	-4.50588100	-3.75032700
C	-5.59915600	-2.27458700	0.32747100
H	-5.61177500	-3.24805800	-0.18012100
H	-5.56722800	-2.47618400	1.40598300
H	-6.55118800	-1.77462400	0.11570600
C	-3.76736300	1.89886800	-1.82882100
H	-3.58796500	1.81002900	-2.90874600
H	-4.79289200	2.26091300	-1.69091800
H	-3.07852700	2.67121700	-1.45973300
C	-2.11283500	5.81766300	0.05656300
H	-2.37670700	4.76394900	-0.00448900
C	-2.56553600	6.68945100	-0.90661800
H	-3.19766900	6.32177100	-1.71373600
C	-2.20589400	8.05234800	-0.86257300
C	-1.36890600	8.50545400	0.12863300
H	-1.06057200	9.55062600	0.15733900
H	-2.57639500	8.73727000	-1.62327600
H	1.25883000	7.55586200	3.79069300
H	0.33399900	9.12983800	2.11024300
C	-4.42016800	0.14343000	3.83432900
C	-5.06403200	-0.88592200	4.48324300
H	-4.54896300	-1.42617400	5.27594100
C	-7.04934500	-0.51627800	3.17590000
C	-6.38800500	-1.23178700	4.14486200
H	-6.88777100	-2.04853700	4.66273200
H	-8.08164700	-0.75448900	2.91818800

H	-3.41322900	0.41380200	4.14264600
H	-8.15436400	1.03323700	1.29853400
H	-7.05822500	2.94306200	0.14349400
Pd	1.78788500	-0.57215000	-0.53487100
C	-0.23347700	-3.30386200	-0.48086600
P	-0.31284500	-1.60039000	0.17518300
Si	1.22222800	1.23243000	-1.92017500
C	2.55126800	2.13913500	-2.92657600
H	3.06095000	1.47454400	-3.63514900
H	2.07220300	2.93629900	-3.51293600
H	3.31465200	2.60868500	-2.29321400
C	0.32848800	2.66837000	-1.04757800
H	-0.15203700	3.33284800	-1.78205000
H	-0.43101400	2.37480700	-0.31380700
H	1.06494500	3.28220700	-0.51040600
C	0.08859400	0.47091200	-3.24663000
C	0.20049300	-0.88666800	-3.58704600
C	-0.88138000	1.22429000	-3.92694300
C	-0.62640700	-1.47073800	-4.54424400
H	0.95041100	-1.50385100	-3.08335400
C	-1.71907100	0.64702500	-4.87926400
H	-1.00024500	2.28640000	-3.69786300
C	-1.59949700	-0.70751200	-5.18566400
H	-0.50899600	-2.52896000	-4.78283000
H	-2.47116100	1.25507500	-5.38302300
H	-2.25782200	-1.16169900	-5.92530900
C	2.16348000	-3.51205400	3.80491900
C	2.69422100	-4.69707600	4.31248800
C	3.78934400	-5.28732000	3.68574400
C	4.34468800	-4.70569600	2.55012700

C	3.80670000	-3.52566900	2.01606900
C	2.72103400	-2.92817600	2.67515600
H	1.31348100	-3.03438300	4.28983200
H	2.25865000	-5.15602300	5.19843600
H	4.21091300	-6.21124300	4.07736800
H	5.18315900	-5.19515500	2.05915000
H	2.31246600	-1.99723000	2.27801000
C	4.30542700	-2.90489100	0.78011300
N	3.51457400	-2.19912300	0.04950000
C	5.73722900	-3.00251200	0.24615600
C	5.92683800	-4.31166100	-0.53429800
H	5.82589000	-5.19889600	0.10254700
H	5.19908200	-4.39989100	-1.35046700
H	6.93092700	-4.33716800	-0.97643800
C	6.80242200	-2.86276600	1.32975200
H	7.79464200	-2.77091400	0.87052900
H	6.63343000	-1.96670700	1.94035900
H	6.83006900	-3.72611300	2.00450200
C	5.74179200	-1.79776800	-0.71447400
H	6.38151200	-1.96322400	-1.58982600
H	6.12278400	-0.91076300	-0.18860800
C	4.25521100	-1.56784600	-1.07380100
C	3.77533700	-0.11669400	-1.04350200
H	4.01936900	0.41853100	-1.96305000
H	4.21242500	0.42114900	-0.18828700
C	3.85509500	-2.19898300	-2.40378200
C	2.95954800	-3.26719100	-2.48775800
C	4.37253500	-1.67098000	-3.59223400
C	2.58022800	-3.78669400	-3.72463100
H	2.53883300	-3.67984200	-1.57347700

C	3.99124700	-2.18306200	-4.82835300
H	5.07645000	-0.83839300	-3.54727100
C	3.08842000	-3.24293500	-4.90049800
H	1.87531800	-4.61765000	-3.75956100
H	4.40003400	-1.75109800	-5.74062100
H	2.78448200	-3.63968500	-5.86790600

TS3

S	2.40647900	-0.87692800	3.42056900
O	2.63811200	-1.81318500	4.58875200
N	1.22520700	0.33860500	3.84691400
C	1.47706100	-4.35663700	1.59699500
H	1.74256100	-3.70918500	2.42985700
C	2.13448900	-5.57995900	1.45214400
C	1.78657800	-6.40686900	0.37405600
H	2.29500500	-7.36160500	0.25137400
C	0.80115400	-6.03493300	-0.54708400
C	0.14263400	-4.80931100	-0.37254700
H	-0.63842500	-4.52074500	-1.07000000
C	-1.79719800	-2.23357500	-0.06745900
C	-3.05717300	-2.17285500	0.53936800
H	-3.15346500	-2.25831200	1.61654600
C	-4.21788300	-1.98423500	-0.22345200
C	-4.09437600	-1.85985800	-1.60957200
H	-4.98801800	-1.69459200	-2.20970200
C	-2.84692500	-1.92051200	-2.24703000
C	-1.70221800	-2.10371000	-1.46535200
H	-0.72250100	-2.09593000	-1.93855000
C	-0.80648700	-2.31288200	2.64576200
C	-1.41853000	-3.48877000	3.11873000
H	-1.43876400	-4.36925000	2.48351600

C	-2.01725700	-3.53864600	4.37385700
H	-2.48732000	-4.45659200	4.71569700
C	-2.02983800	-2.39485700	5.17612900
C	-1.41037700	-1.23175000	4.72893600
H	-1.43821900	-0.34131500	5.34812900
C	-0.76583200	-1.17660000	3.48195800
C	1.10326200	0.60716100	5.28235500
H	0.92652400	-0.29785300	5.87557200
H	0.27600100	1.30782600	5.42586800
H	2.01746000	1.08311100	5.64219700
C	3.98811500	0.15749600	3.26489100
C	3.67264800	1.42766300	2.47570300
H	4.61099700	1.92486600	2.19884700
H	3.06939100	2.12342000	3.06372800
H	3.12312100	1.20708700	1.55528900
C	4.58439200	0.44611800	4.64275300
H	4.05588000	1.25771800	5.15169500
H	5.63310100	0.74859900	4.53328500
H	4.54280100	-0.44837200	5.27199700
C	4.90436900	-0.78295600	2.46080300
H	5.86109500	-0.28326700	2.26808300
H	4.46559600	-1.05403100	1.49442900
H	5.10803500	-1.70600000	3.01414000
C	-0.89495100	1.31293500	2.92067100
C	-2.22623000	1.14631300	2.52713100
H	-2.63769100	0.14751400	2.43912100
C	-3.02183000	2.24057900	2.16030800
C	-2.45185400	3.52022900	2.18407900
H	-3.05455100	4.37988200	1.90696500
C	-1.11132800	3.70834000	2.54930500

C	-0.34680600	2.59930300	2.93427800
H	0.69815400	2.73118100	3.19332500
C	0.71175300	7.62041400	2.53084800
C	0.15051200	7.13020200	1.32118100
C	-0.45494600	5.82822500	1.29786900
C	-0.48828300	5.05864400	2.51093100
C	0.09503300	5.57191400	3.65665400
H	0.07046700	4.98061500	4.56810400
C	0.69406400	6.85225000	3.67210700
C	-4.40760000	2.04611600	1.65625900
C	-5.38013300	1.24465000	2.34924700
C	-6.68341100	1.08094300	1.76629400
C	-6.98731200	1.71468200	0.53215200
C	-6.04362800	2.48347600	-0.10842900
C	-4.75888100	2.64400000	0.45565000
H	-4.01326200	3.22543100	-0.07910400
C	-0.01668200	0.08650300	3.07279100
H	0.35943600	-0.08044700	2.05053300
H	-2.51922900	-2.40968200	6.14624500
C	3.23346700	-5.96810500	2.41315600
H	3.00831200	-5.63579800	3.43202700
H	3.39408300	-7.05141700	2.42926500
H	4.18116800	-5.49476500	2.12308600
C	0.45861600	-6.92317900	-1.72151400
H	0.77829200	-7.95678100	-1.55315400
H	-0.62032100	-6.92912100	-1.91538600
H	0.95050500	-6.57173800	-2.63821000
C	-5.57206100	-1.92837300	0.44358900
H	-6.08908400	-2.89465800	0.36891900
H	-5.48874800	-1.67709700	1.50519000

H	-6.21326500	-1.17445100	-0.02359200
C	-2.74474400	-1.72427200	-3.74158400
H	-1.73984500	-1.94004500	-4.10835200
H	-3.44381200	-2.37420800	-4.28067400
H	-2.98776200	-0.68866400	-4.01445600
C	-0.95396200	5.34246700	0.05539100
H	-1.37116900	4.34309200	0.00759300
C	-0.88409300	6.10588400	-1.08964600
H	-1.25959100	5.70543900	-2.02745300
C	-0.31342900	7.40127700	-1.05574900
C	0.19393500	7.89661000	0.12394800
H	0.64799000	8.88437600	0.15652200
H	-0.26773200	7.99595500	-1.96403200
H	1.13050700	7.22769600	4.59364900
H	1.16311100	8.60972600	2.53562500
C	-5.12672700	0.61116000	3.59953200
C	-6.08080200	-0.17262900	4.21171500
H	-5.85505000	-0.65081300	5.16102000
C	-7.64718100	0.27332700	2.42932800
C	-7.35405000	-0.35162000	3.61968100
H	-8.09777000	-0.97305300	4.11085400
H	-8.62587000	0.15329800	1.97054200
H	-4.16582600	0.75010900	4.08085400
H	-7.97656900	1.57688200	0.10213300
H	-6.27417800	2.96086000	-1.05678700
Pd	1.32041100	-0.77799800	0.07007800
C	0.47777000	-3.96533800	0.69155400
P	-0.21918600	-2.27257400	0.88704300
C	-0.55799400	-4.60014100	-4.54302700
C	-1.11232900	-4.28949700	-5.78789500

C	-0.66745700	-3.16115300	-6.47929100
C	0.33481300	-2.35253800	-5.93881400
C	0.90407300	-2.65932700	-4.69239800
C	0.43890600	-3.79212900	-3.99992800
H	-0.90377900	-5.47047600	-3.99303500
H	-1.89103000	-4.91791400	-6.21143300
H	-1.10625000	-2.90186600	-7.43900900
H	0.64347100	-1.46108100	-6.46894100
H	0.86558800	-4.02429600	-3.03013600
C	1.96363600	-1.82995300	-4.06205500
N	1.92115600	-1.61698400	-2.79868100
C	3.24318800	-1.34033900	-4.76514500
C	4.16139600	-2.56823300	-4.97302200
H	3.69154200	-3.28686300	-5.65466500
H	4.37456000	-3.07843300	-4.02965700
H	5.11378600	-2.25186200	-5.41488200
C	3.07079200	-0.61046900	-6.10530700
H	4.03126800	-0.16619700	-6.39424700
H	2.33558100	0.19603800	-6.03664000
H	2.77625300	-1.29563500	-6.90702000
C	3.77410500	-0.38359300	-3.67481700
H	4.86630800	-0.34488500	-3.65045600
H	3.40174900	0.62480000	-3.87790800
C	3.14205900	-0.90247300	-2.35023200
C	2.80003300	0.24215400	-1.34403600
H	3.27383300	0.03083100	-0.36459200
H	3.33025000	1.15429500	-1.64224700
C	4.07271500	-1.89784500	-1.63010900
C	3.65561500	-3.18225100	-1.26152400
C	5.37246800	-1.49605800	-1.28247200

C	4.52202900	-4.04795900	-0.58726500
H	2.64655800	-3.50398400	-1.48806700
C	6.23496100	-2.35401700	-0.60071800
H	5.71181900	-0.49548700	-1.53897500
C	5.81352600	-3.64063800	-0.25310500
H	4.17221400	-5.04033000	-0.32332900
H	7.23586800	-2.01735400	-0.34259000
H	6.48438900	-4.31490400	0.27274800
Si	1.04126500	1.33775900	-1.34894800
C	1.66184600	2.90100100	-0.46360600
H	2.72776300	3.11148500	-0.60962300
H	1.09484300	3.77678800	-0.79818200
H	1.48600700	2.79962900	0.61346000
C	-0.81257900	1.26761900	-0.87546600
H	-1.26964000	2.16660100	-1.31687000
H	-1.34271300	0.39458700	-1.25910800
H	-0.96333100	1.30519900	0.20459900
C	0.91186400	1.68106100	-3.21609800
C	1.61073400	2.73029200	-3.83831300
C	0.02550000	0.93205400	-4.01236800
C	1.43350400	3.02362700	-5.19446600
H	2.29893300	3.33980000	-3.25513000
C	-0.16349500	1.22004400	-5.36492100
H	-0.52951100	0.11045200	-3.56939000
C	0.54082600	2.27090100	-5.96116300
H	1.98511700	3.84288700	-5.64954800
H	-0.85839600	0.62380900	-5.95149400
H	0.39541900	2.50038300	-7.01384500

TS3-ent

S	1.15411100	1.31983400	4.98853400

O	1.69524100	2.73398600	4.87866100
N	1.43816000	0.44669800	3.52979800
C	0.57774100	-4.23177300	-0.25190300
H	1.24562300	-3.95341500	0.55816300
C	0.82309900	-5.40792100	-0.95982600
C	-0.03199300	-5.74141800	-2.02253800
H	0.15175500	-6.65519700	-2.58558500
C	-1.11266200	-4.92737600	-2.37415900
C	-1.33280600	-3.74427100	-1.65200600
H	-2.15288200	-3.09205400	-1.93648200
C	-2.26513800	-1.10572100	-0.14975800
C	-3.42039000	-1.75489300	0.30792900
H	-3.34001000	-2.67528100	0.87914500
C	-4.68758200	-1.21616100	0.06529700
C	-4.77716100	-0.01927700	-0.65664500
H	-5.75689000	0.41917000	-0.82850500
C	-3.64058000	0.64828700	-1.12313700
C	-2.38414800	0.09247500	-0.85863300
H	-1.48783600	0.59435500	-1.19885100
C	-0.73323300	-2.13040600	2.01952400
C	-1.28186600	-3.36487100	2.41372200
H	-1.48315700	-4.12095500	1.66159000
C	-1.57991700	-3.63956200	3.74623800
H	-2.00660300	-4.60071800	4.01981700
C	-1.34950100	-2.66228600	4.71612100
C	-0.77599400	-1.44908300	4.34395300
H	-0.60282100	-0.69102100	5.09900500
C	-0.42362300	-1.17263900	3.01579300
C	2.68331600	0.76881500	2.81223200
H	3.16365800	1.63749000	3.26977800

H	2.46141400	1.02656300	1.77365700
H	3.37226100	-0.08315200	2.81539500
C	2.36464200	0.47555000	6.19398900
C	2.17603700	-1.03883800	6.14518800
H	2.86843300	-1.50627600	6.85673200
H	2.38558000	-1.43450600	5.14937300
H	1.16117800	-1.33723700	6.42607700
C	3.80634100	0.87998500	5.87738700
H	4.19850100	0.34125200	5.01115500
H	4.44317300	0.63864700	6.73727600
H	3.87173700	1.95615100	5.69085400
C	1.94025100	1.05094800	7.55601700
H	2.56977900	0.61995700	8.34343700
H	0.89651500	0.80875100	7.78957100
H	2.05780400	2.13891600	7.57793200
C	-0.72411500	1.32257000	2.53024400
C	-2.10542000	1.13490200	2.46260900
H	-2.50908100	0.13181500	2.50153900
C	-2.98291800	2.22314800	2.30329300
C	-2.43354100	3.50532500	2.16349300
H	-3.09073000	4.36431300	2.07008500
C	-1.04638700	3.70537300	2.15028500
C	-0.20523200	2.60904100	2.34942100
H	0.86464700	2.77379400	2.36893600
C	0.72637300	7.60712400	1.68429600
C	-0.16165600	7.14673500	0.67535000
C	-0.75524600	5.84497700	0.79815400
C	-0.44864000	5.05175000	1.95499900
C	0.45140900	5.52838800	2.89455800
H	0.70263400	4.90220400	3.74753100

C	1.03735400	6.80822100	2.76117400
C	-4.45442600	2.02990600	2.20453000
C	-5.18109900	1.09785400	3.02755700
C	-6.58063600	0.89040300	2.77058000
C	-7.23253800	1.64418800	1.75963100
C	-6.53322500	2.57617800	1.02895700
C	-5.15061700	2.75361500	1.24668300
H	-4.60657100	3.44155200	0.60730400
C	0.24985900	0.15551100	2.67799500
H	0.68963000	0.02151100	1.67659100
H	-1.60777600	-2.84361500	5.75605800
C	2.02274200	-6.26564800	-0.62660900
H	2.85067000	-6.06230000	-1.31961100
H	2.38911900	-6.06327700	0.38539400
H	1.79114700	-7.33412200	-0.70229200
C	-2.04790800	-5.31220800	-3.49753700
H	-1.56799800	-5.99641700	-4.20546000
H	-2.94435700	-5.81504700	-3.11082100
H	-2.38699400	-4.42916200	-4.05040300
C	-5.92119100	-1.89833000	0.61003900
H	-5.93622200	-2.96351000	0.34898600
H	-5.95663700	-1.82588400	1.70348900
H	-6.83626200	-1.43865700	0.22362100
C	-3.75113000	1.94148200	-1.89486400
H	-3.59131500	1.77120000	-2.96634000
H	-4.73496200	2.40355800	-1.76570800
H	-2.98956100	2.65960400	-1.56883400
C	-1.58239800	5.38148200	-0.26475000
H	-1.99902400	4.38212800	-0.20991500
C	-1.83867900	6.16872900	-1.36586000

H	-2.46731900	5.78808900	-2.16673700
C	-1.28105600	7.46663700	-1.46603000
C	-0.45832500	7.93845400	-0.46831000
H	-0.01076100	8.92683000	-0.54525000
H	-1.49407800	8.08141400	-2.33642900
H	1.72899200	7.15900500	3.52239200
H	1.16629200	8.59652600	1.58234500
C	-4.59630500	0.37908600	4.10972600
C	-5.32001400	-0.53942100	4.83824000
H	-4.84008800	-1.07714800	5.65145900
C	-7.29880300	-0.06485700	3.54146900
C	-6.68322800	-0.77941200	4.54350100
H	-7.24347500	-1.50996500	5.12072700
H	-8.35292500	-0.21898100	3.32256400
H	-3.56307300	0.56507700	4.37548900
H	-8.29167700	1.47608600	1.58002400
H	-7.03112700	3.15893300	0.25854500
Pd	1.36662400	-0.67145600	-0.39625400
C	-0.49249200	-3.38717100	-0.59456800
P	-0.56906600	-1.73163500	0.20413000
Si	1.49653400	1.06029500	-2.16592500
C	2.46955900	2.01132500	-3.51620300
H	3.01161300	1.32695600	-4.17953900
H	1.75732700	2.57263700	-4.13401400
H	3.18701900	2.72846600	-3.09556200
C	0.72350600	2.48072200	-1.15485900
H	0.29400400	3.24881400	-1.81169300
H	-0.04702600	2.14402700	-0.45687300
H	1.50428800	2.97138300	-0.55962800
C	0.21700500	0.17769400	-3.27700400

C	0.24740200	-1.19350100	-3.58243900
C	-0.79905500	0.95185700	-3.87044300
C	-0.69550900	-1.77011900	-4.43630300
H	1.00754000	-1.82687300	-3.14039500
C	-1.75657700	0.37802700	-4.71046800
H	-0.85837600	2.01950000	-3.66642800
C	-1.70820500	-0.98879800	-4.99664700
H	-0.63714700	-2.83334500	-4.65311300
H	-2.53663200	0.99843300	-5.14649000
H	-2.45021400	-1.43750200	-5.65272500
C	2.32768800	-3.62322400	3.56439000
C	3.16998100	-4.58104100	4.13792400
C	4.44903700	-4.77805500	3.61370700
C	4.88659600	-4.02993200	2.51871400
C	4.04733700	-3.07299200	1.92466900
C	2.76682400	-2.87451400	2.47656300
H	1.33175000	-3.45157600	3.96059100
H	2.83239600	-5.16637300	4.98890700
H	5.11038700	-5.51983600	4.05307500
H	5.87701300	-4.21362400	2.12085100
H	2.11716900	-2.12827800	2.03857100
C	4.42971800	-2.29785600	0.71929300
N	3.50777800	-1.79930500	-0.02087400
C	5.85960500	-2.02715100	0.21321600
C	6.42367700	-3.27096300	-0.51304400
H	6.55728700	-4.12121900	0.16230900
H	5.76105300	-3.58457800	-1.32558100
H	7.40237800	-3.03062200	-0.94449200
C	6.81945600	-1.55383200	1.31707600
H	7.76045700	-1.21432200	0.86861500

H	6.38904000	-0.71490100	1.87631700
H	7.05752700	-2.34676600	2.03172800
C	5.57192400	-0.89183600	-0.80666100
H	6.22479800	-0.94246000	-1.68236200
H	5.73027400	0.08089900	-0.32679900
C	4.05980500	-1.04343200	-1.15944100
C	3.32923200	0.33102700	-1.20288200
H	3.88099900	0.94621800	-1.91598100
H	3.47670300	0.82917300	-0.23519700
C	3.83051700	-1.82630900	-2.45856500
C	3.18380400	-3.06878900	-2.45979200
C	4.25135200	-1.29204400	-3.68430900
C	2.94230600	-3.75025900	-3.65475200
H	2.83300000	-3.48597700	-1.52498000
C	4.01223300	-1.97064100	-4.88020600
H	4.76592600	-0.33506100	-3.70805800
C	3.35134800	-3.20223900	-4.87119800
H	2.40902300	-4.69711200	-3.62630100
H	4.33828600	-1.53343000	-5.82021000
H	3.15635100	-3.72484600	-5.80367800

Int5

S	2.31051200	-0.88432700	3.21152600
O	2.46207300	-1.75196200	4.43169600
N	0.99052600	0.22337900	3.39876100
C	1.21814600	-5.18559900	0.35403000
H	2.08270500	-4.53703900	0.52654600
C	1.40911000	-6.52573900	0.00474900
C	0.28347800	-7.30939700	-0.25182300
H	0.41793700	-8.35408600	-0.54062300
C	-1.01233200	-6.78915500	-0.16001600

C	-1.16978400	-5.44915900	0.19984000
H	-2.17598700	-5.03329400	0.25920100
C	-1.78343700	-2.29402300	0.13016900
C	-3.00620100	-2.66868400	0.70249800
H	-3.02088500	-3.34801100	1.55765200
C	-4.21722600	-2.16120300	0.22643800
C	-4.18179800	-1.21413500	-0.80150900
H	-5.11983600	-0.77206400	-1.14476000
C	-2.97948100	-0.80230400	-1.37974100
C	-1.79023300	-1.37958500	-0.92551900
H	-0.83604400	-1.10725500	-1.38513600
C	-0.65743400	-2.76006100	2.59776600
C	-1.06627300	-3.91096400	3.28138600
H	-0.93580700	-4.88544100	2.81258200
C	-1.67611000	-3.83461500	4.53048000
H	-1.98790600	-4.74615100	5.03811200
C	-1.91849600	-2.59114700	5.10474500
C	-1.48249300	-1.44152800	4.45454300
H	-1.66496600	-0.46583500	4.90543400
C	-0.81558900	-1.50280900	3.22709700
C	0.76481700	0.66732900	4.76689400
H	0.68495400	-0.16266300	5.48425200
H	-0.15630300	1.26019600	4.79248200
H	1.59086700	1.31607500	5.07848500
C	3.77277600	0.33657700	3.28291800
C	3.37989700	1.62528900	2.58773900
H	4.27786500	2.21447400	2.35132400
H	2.71606800	2.24313700	3.20424600
H	2.85550900	1.42424500	1.64419400
C	4.23242500	0.53664400	4.71535600

H	3.62551300	1.27510200	5.25079300
H	5.27099300	0.89248400	4.72504300
H	4.18752700	-0.40812400	5.26955400
C	4.83415300	-0.41556100	2.48334600
H	5.74162700	0.19540400	2.39069700
H	4.48474700	-0.65946700	1.47003100
H	5.11090300	-1.35692300	2.97477100
C	-1.13852300	0.91058500	2.36892600
C	-2.48058200	0.68063200	2.07063200
H	-2.87597600	-0.33307700	2.13859300
C	-3.31594900	1.71651500	1.62582400
C	-2.76073900	2.98657400	1.43192300
H	-3.39517100	3.79807100	1.07389000
C	-1.41171900	3.23734200	1.70549600
C	-0.62594500	2.20173800	2.21516800
H	0.43265800	2.37860700	2.40496000
C	0.49160900	7.01911200	0.93281800
C	-0.15621600	6.35820600	-0.13781100
C	-0.80727700	5.10183000	0.09834700
C	-0.78230200	4.54529500	1.41871900
C	-0.09559200	5.21225600	2.41782900
H	-0.06288200	4.77147500	3.41437300
C	0.53272700	6.45028900	2.18276000
C	-4.73432500	1.46907900	1.29481600
C	-5.59344100	0.69723100	2.14488500
C	-6.93438800	0.42467700	1.71383100
C	-7.39000100	0.93952400	0.47699200
C	-6.56592100	1.71921000	-0.29972800
C	-5.24661900	1.97970000	0.11245100
H	-4.59174300	2.56322400	-0.53528200

C	-0.20144600	-0.24371200	2.64105600
H	0.21844900	-0.51396700	1.65285200
H	-2.43598900	-2.51407700	6.05967200
C	2.79503600	-7.08658500	-0.10607200
H	3.30575700	-7.09249800	0.86570200
H	2.78943100	-8.11483300	-0.48360900
H	3.41717500	-6.48514200	-0.78238300
C	-2.20226900	-7.64540000	-0.47548700
H	-2.11981200	-8.64017000	-0.02129300
H	-3.13352400	-7.18945700	-0.12213200
H	-2.30823400	-7.80195900	-1.55767200
C	-5.51466600	-2.59738400	0.83804400
H	-5.63183300	-3.68830000	0.79945200
H	-5.57720900	-2.30866400	1.89634600
H	-6.37369200	-2.14765200	0.32795300
C	-2.94380200	0.27270300	-2.42424100
H	-2.34779100	-0.02640300	-3.29764900
H	-3.94932400	0.54081700	-2.76980600
H	-2.47786700	1.18804800	-2.02688700
C	-1.38047600	4.43142000	-1.01376800
H	-1.85428500	3.46419200	-0.85955400
C	-1.31853800	4.96213700	-2.28154700
H	-1.74070300	4.40825000	-3.11898100
C	-0.69707100	6.20802900	-2.50476900
C	-0.13528300	6.88965500	-1.45135900
H	0.35919000	7.84766800	-1.61289100
H	-0.65375200	6.62125100	-3.51090400
H	1.04600100	6.95507800	2.99908800
H	0.97376500	7.97761800	0.74111700
C	-5.19725400	0.21307300	3.41883500

C	-6.04784700	-0.53750300	4.19774200
H	-5.71048300	-0.89649900	5.16887700
C	-7.78327900	-0.35432900	2.53866600
C	-7.35118000	-0.83675400	3.75103000
H	-8.01497600	-1.43580100	4.37177900
H	-8.79556600	-0.55987700	2.18978800
H	-4.20576800	0.45361500	3.79396700
H	-8.40844400	0.71500500	0.15992600
H	-6.92173500	2.12215100	-1.24657300
Pd	1.56553600	-1.73909700	-0.16606000
C	-0.06138200	-4.63315200	0.45660200
P	-0.14880500	-2.83223000	0.81465400
C	-0.30505600	-3.93720000	-3.06017700
C	-1.24233300	-3.42714500	-3.95638000
C	-0.98845600	-2.23015600	-4.62263800
C	0.20016300	-1.54003400	-4.39422800
C	1.13574500	-2.02846500	-3.47463300
C	0.87253100	-3.23770000	-2.81108600
H	-0.49348900	-4.87306100	-2.53528100
H	-2.17163100	-3.96479500	-4.13778500
H	-1.71907000	-1.82744600	-5.32266500
H	0.38783500	-0.59508400	-4.90231600
H	1.60514400	-3.62876300	-2.10423400
C	2.37756200	-1.29299700	-3.17634000
N	2.68043000	-0.95625400	-1.96636400
C	3.46713600	-1.03090400	-4.20714000
C	4.16026700	-2.37649300	-4.47430500
H	3.46191500	-3.10088100	-4.91238800
H	4.56840800	-2.81048200	-3.55241400
H	4.98973700	-2.23782600	-5.17925800

C	2.99532100	-0.43218800	-5.52755000
H	3.86504800	-0.20369500	-6.15693900
H	2.43843900	0.50192300	-5.37892600
H	2.35989100	-1.13034600	-6.08618900
C	4.36758200	-0.06989900	-3.42052400
H	5.43350800	-0.23514100	-3.61865200
H	4.13501300	0.96390000	-3.70783400
C	4.00353700	-0.28726900	-1.93240600
C	3.86943200	1.05197600	-1.18317600
H	3.90337400	0.85706000	-0.09957400
H	4.76591600	1.65646500	-1.39120000
C	5.01002000	-1.19199300	-1.22246200
C	4.68979100	-2.47982100	-0.77746100
C	6.30745600	-0.71844000	-0.99326900
C	5.63081800	-3.25667100	-0.10203500
H	3.68506300	-2.87721100	-0.93964500
C	7.25058200	-1.49554900	-0.32995000
H	6.58054500	0.28026300	-1.33697800
C	6.91288300	-2.76923800	0.12658300
H	5.35040600	-4.24943500	0.24889200
H	8.25277500	-1.10293100	-0.16419100
H	7.64710500	-3.37574800	0.65418400
Si	2.32788900	2.14644400	-1.43982300
C	2.72874200	3.80298200	-0.63677800
H	3.68406700	4.22226200	-0.97684600
H	1.94680600	4.53919200	-0.86162700
H	2.77884300	3.71245200	0.45702800
C	0.82222900	1.42023900	-0.60516500
H	0.00326500	2.15046800	-0.56275400
H	0.45988700	0.51395200	-1.10447700

H	1.05269200	1.12378100	0.42651000
C	1.87723600	2.45643700	-3.24812800
C	2.67880600	3.23547900	-4.09930000
C	0.66583400	1.97535600	-3.76891100
C	2.29238800	3.52144500	-5.40598600
H	3.62742200	3.63351800	-3.73100200
C	0.26757800	2.25850400	-5.07491900
H	0.01277200	1.36749200	-3.14036300
C	1.08123200	3.03447600	-5.89701400
H	2.93263100	4.12987800	-6.04320600
H	-0.68177300	1.87195600	-5.44819700
H	0.77490200	3.26017400	-6.91729300

Int5-ent

S	1.15718700	1.18379400	4.69298700
O	1.77890300	2.54962400	4.54751200
N	1.29130900	0.30669900	3.19038600
C	0.05555000	-4.74699400	-0.23331100
H	0.90422500	-4.50878500	0.41459100
C	-0.01894200	-5.99931200	-0.83347100
C	-1.09172800	-6.26080700	-1.69996200
H	-1.15277500	-7.23289900	-2.19398900
C	-2.07607800	-5.30945900	-1.95264600
C	-1.98912800	-4.06331200	-1.31453500
H	-2.75562000	-3.31220800	-1.50893400
C	-2.13668600	-1.15051900	-0.18044000
C	-3.38634900	-1.47310800	0.36380500
H	-3.47930800	-2.32924200	1.03636900
C	-4.51161500	-0.69831800	0.08734500
C	-4.35734000	0.41772500	-0.74253400
H	-5.22766800	1.04650300	-0.94277100

C	-3.12644100	0.76406400	-1.30026100
C	-2.01574700	-0.03766800	-1.01152200
H	-1.03837700	0.20785700	-1.43201700
C	-0.92001500	-2.32856800	2.05570600
C	-1.48196800	-3.51428700	2.55283700
H	-1.70211400	-4.32804200	1.86290300
C	-1.77939500	-3.66916900	3.90280300
H	-2.21417800	-4.60197200	4.25860500
C	-1.54389400	-2.61896000	4.78514100
C	-0.98517100	-1.43953500	4.30600900
H	-0.81832900	-0.60564800	4.98870300
C	-0.63594300	-1.28034000	2.96293700
C	2.36005600	0.80389500	2.31518900
H	2.75585200	1.75553800	2.69663300
H	1.96685500	0.97380800	1.30635300
H	3.18566800	0.08300100	2.23041400
C	2.41341100	0.23111300	5.73310300
C	1.88032700	-1.17024200	5.95845000
H	2.60637900	-1.74909000	6.54484800
H	1.72386600	-1.68994900	5.00623400
H	0.93341500	-1.16739700	6.51338800
C	3.76651400	0.20745900	5.04612800
H	3.77922300	-0.49968400	4.20872300
H	4.53288200	-0.11443100	5.76386400
H	4.04619600	1.20527400	4.68587900
C	2.47969100	1.02261600	7.03331600
H	3.15232200	0.51607300	7.73709700
H	1.49550400	1.10217700	7.51315800
H	2.85924000	2.03490500	6.86064700
C	-0.96776700	1.19943200	2.46136900

C	-2.35271100	1.03780000	2.52276000
H	-2.76974700	0.03943100	2.63961400
C	-3.22148000	2.13278800	2.38974300
C	-2.66753900	3.39466800	2.14322300
H	-3.32576900	4.25919900	2.05994900
C	-1.28473800	3.57800000	2.04040900
C	-0.45023400	2.47660700	2.22729300
H	0.62557400	2.62951900	2.17361700
C	0.49662200	7.43448900	1.38666700
C	-0.50258800	6.99633500	0.48533900
C	-1.10083800	5.70676100	0.66978800
C	-0.68533900	4.90277500	1.77841700
C	0.32528700	5.35910400	2.60807200
H	0.65307600	4.72635600	3.43402400
C	0.91300900	6.62437400	2.41628400
C	-4.69276400	1.99041100	2.44172400
C	-5.37002400	1.16360600	3.39925100
C	-6.80093000	1.05669300	3.33344400
C	-7.51914000	1.79909600	2.36674700
C	-6.85451300	2.60973600	1.47832500
C	-5.45162000	2.69134300	1.51562700
H	-4.93491500	3.29537900	0.76889300
C	-0.00922800	0.02426200	2.50617800
H	0.31912900	-0.15402000	1.46340100
H	-1.79879300	-2.71240200	5.84000400
C	1.03201500	-7.03786200	-0.57844600
H	1.52859800	-7.34773500	-1.50717800
H	1.80456700	-6.66515500	0.10392700
H	0.60551500	-7.94692500	-0.13443500
C	-3.20530200	-5.59234000	-2.89748200

H	-3.16688600	-6.61769500	-3.28117400
H	-4.18164500	-5.45205800	-2.41646200
H	-3.18471300	-4.91416100	-3.76115900
C	-5.84953800	-1.05170200	0.66202500
H	-6.35978500	-1.81055300	0.05230400
H	-5.76069400	-1.46248600	1.67568700
H	-6.50772400	-0.17657500	0.71160200
C	-3.00019300	1.95757400	-2.19751100
H	-3.00185800	1.66427100	-3.25635800
H	-3.82941200	2.66152800	-2.05227300
H	-2.06121300	2.49846000	-2.02508300
C	-2.03852600	5.26102100	-0.29643200
H	-2.45722600	4.26086400	-0.20117000
C	-2.39959600	6.05455900	-1.36106800
H	-3.11390200	5.68363500	-2.09478200
C	-1.84096400	7.34038900	-1.51538200
C	-0.90833600	7.79389800	-0.61299400
H	-0.45269500	8.77669300	-0.73534200
H	-2.13677700	7.96338900	-2.35767300
H	1.69218100	6.96057800	3.09827300
H	0.93822000	8.42006600	1.23910300
C	-4.70512200	0.45454500	4.43321100
C	-5.39161500	-0.36982100	5.29497400
H	-4.84994100	-0.90409400	6.07379900
C	-7.47632500	0.20029300	4.23698900
C	-6.78960600	-0.51572200	5.18755400
H	-7.32246500	-1.17601800	5.86957400
H	-8.56084100	0.12207400	4.16038100
H	-3.63250400	0.57716400	4.55658900
H	-8.60473700	1.70706400	2.33647900

H	-7.40532400	3.17550500	0.72868800
Pd	1.47454100	-1.45936600	-0.19068000
C	-0.92837900	-3.77003500	-0.46009300
P	-0.63230400	-2.10785600	0.24087700
Si	2.10609500	1.28463000	-2.28734200
C	2.69008500	2.57773900	-3.53582600
H	3.35418500	2.14546100	-4.29511500
H	1.84951900	3.03846100	-4.06900300
H	3.23763600	3.38855200	-3.03805800
C	0.96866100	2.16606300	-1.06386800
H	0.13083000	2.66950500	-1.56656400
H	0.53836000	1.45574000	-0.34165200
H	1.51069700	2.93277600	-0.49234600
C	1.04821100	0.04731900	-3.24784500
C	1.13126000	-1.35277200	-3.20733900
C	-0.00824500	0.60129700	-3.99384200
C	0.19748800	-2.16182200	-3.85291700
H	1.93250900	-1.82975800	-2.64744200
C	-0.94667400	-0.19821800	-4.64349200
H	-0.11983600	1.68696000	-4.05381900
C	-0.85092200	-1.58628700	-4.56544100
H	0.28334100	-3.24628400	-3.77754000
H	-1.75766300	0.26402800	-5.20589100
H	-1.58991500	-2.21628000	-5.06005400
C	2.29849600	-3.71247500	3.15276500
C	3.02225400	-4.87107100	3.42037000
C	4.25328100	-5.07162900	2.79776900
C	4.74833400	-4.12798800	1.90311300
C	4.01689800	-2.97008300	1.59488900
C	2.79060500	-2.76857100	2.25543300

H	1.33925200	-3.53331500	3.63974200
H	2.63608000	-5.61017100	4.12021800
H	4.83209200	-5.96982400	3.00491800
H	5.70075400	-4.31626100	1.41452300
H	2.21677700	-1.86129300	2.06839800
C	4.49279000	-2.01115800	0.58327000
N	3.66470000	-1.41363800	-0.21712600
C	5.95801500	-1.59819200	0.40399500
C	6.82372500	-2.63738500	-0.32511500
H	7.05665000	-3.50665700	0.29836400
H	6.35792000	-2.99209100	-1.25064900
H	7.78192600	-2.17483000	-0.59319100
C	6.60007700	-1.25449200	1.74806500
H	7.60136500	-0.83308500	1.59348200
H	6.00640200	-0.51318700	2.29939800
H	6.70385600	-2.14130000	2.38550700
C	5.76275800	-0.34939200	-0.47230500
H	6.58026900	-0.19191900	-1.18629000
H	5.71041300	0.54190900	0.16989300
C	4.39020600	-0.53119000	-1.16153700
C	3.67402800	0.82387400	-1.27753900
H	4.42274000	1.54150300	-1.65100100
H	3.47491900	1.15753300	-0.24823700
C	4.51376000	-1.22420800	-2.51763800
C	4.28704500	-2.59461400	-2.68203300
C	4.86487900	-0.47464300	-3.64584000
C	4.39120900	-3.19180500	-3.93642900
H	3.98625100	-3.19773500	-1.82619800
C	4.96779500	-1.06767200	-4.90085100
H	5.05919200	0.59345100	-3.54452900

C	4.72592500	-2.43068000	-5.05214300
H	4.19588100	-4.25804400	-4.04014500
H	5.23350500	-0.45915100	-5.76360300
H	4.79791900	-2.89502900	-6.03388600

SM1

Cu	1.09069000	1.04765700	0.26659200
Si	3.32407800	0.67797400	0.02704100
C	4.25563100	2.32832400	-0.14459700
H	4.11301600	2.95617500	0.74183400
H	5.33228200	2.15038500	-0.26664300
H	3.89726700	2.88988800	-1.01490400
C	3.71566900	-0.39922300	-1.48892100
H	4.79554000	-0.58317600	-1.56749500
H	3.20583400	-1.36770500	-1.44141600
H	3.38996300	0.10430300	-2.40611600
C	3.96179600	-0.19449400	1.58464800
C	4.52855300	-1.48109400	1.54604300
C	3.88033200	0.44366700	2.83735000
C	4.99889700	-2.10301300	2.70638900
H	4.60900200	-2.00976500	0.59901300
C	4.35123200	-0.16824000	3.99920900
H	3.43623900	1.43588600	2.90631900
C	4.91183900	-1.44788700	3.93622100
H	5.43368600	-3.09813500	2.64947400
H	4.27907200	0.34800500	4.95354600
H	5.27599700	-1.93003200	4.83986500

SM2

C	-1.32489500	2.90278800	-5.23255900
C	-0.78058200	3.48228600	-6.38808500
C	-0.92944500	3.42040600	-3.98782100

C	0.12603800	4.53823500	-6.29698900
C	-0.02543200	4.47230100	-3.88303000
H	-1.35342600	2.96897700	-3.09903700
C	0.49805000	5.02458000	-5.04683600
H	0.28256700	4.87224300	-2.92352600
F	-1.09181900	3.06749400	-7.61915600
F	0.63665100	5.08080200	-7.41198900
F	1.37352400	6.03922200	-4.99198800
C	-2.29037100	1.76095300	-5.27568500
O	-2.74900300	1.31818200	-4.17401300
O	-2.62365700	1.25619100	-6.39622300
Cu	-3.79641400	-0.09425800	-5.35867900

9. Copies of NMR spectra

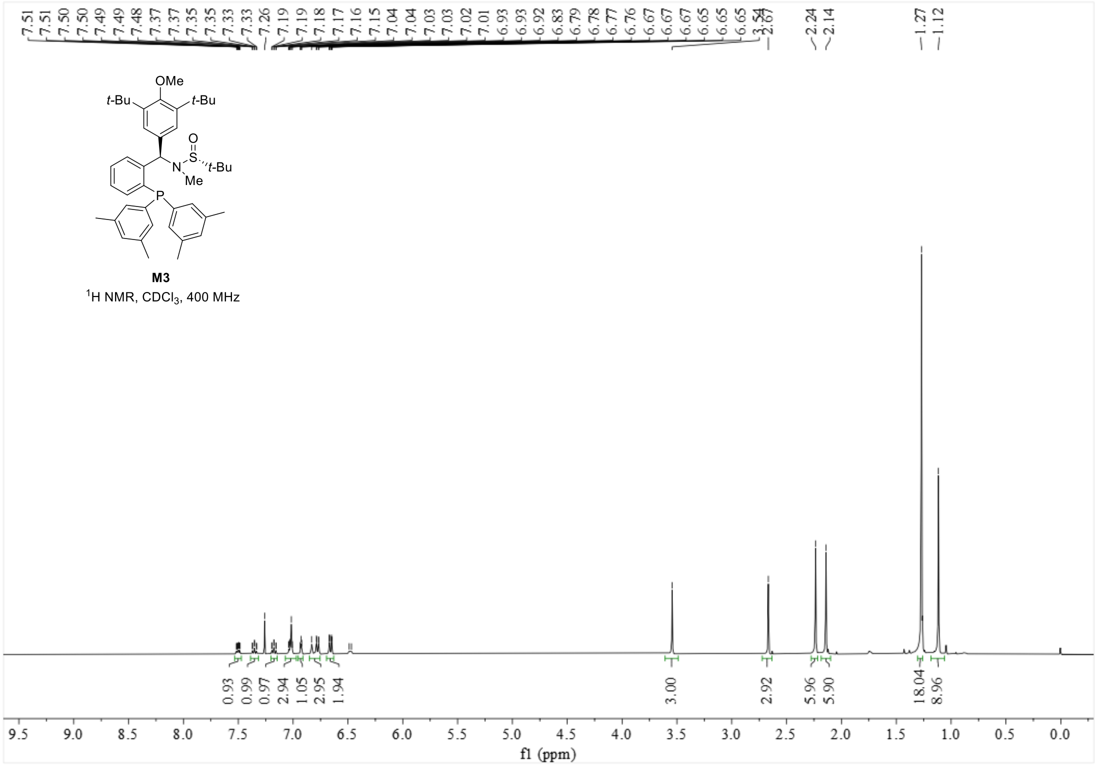

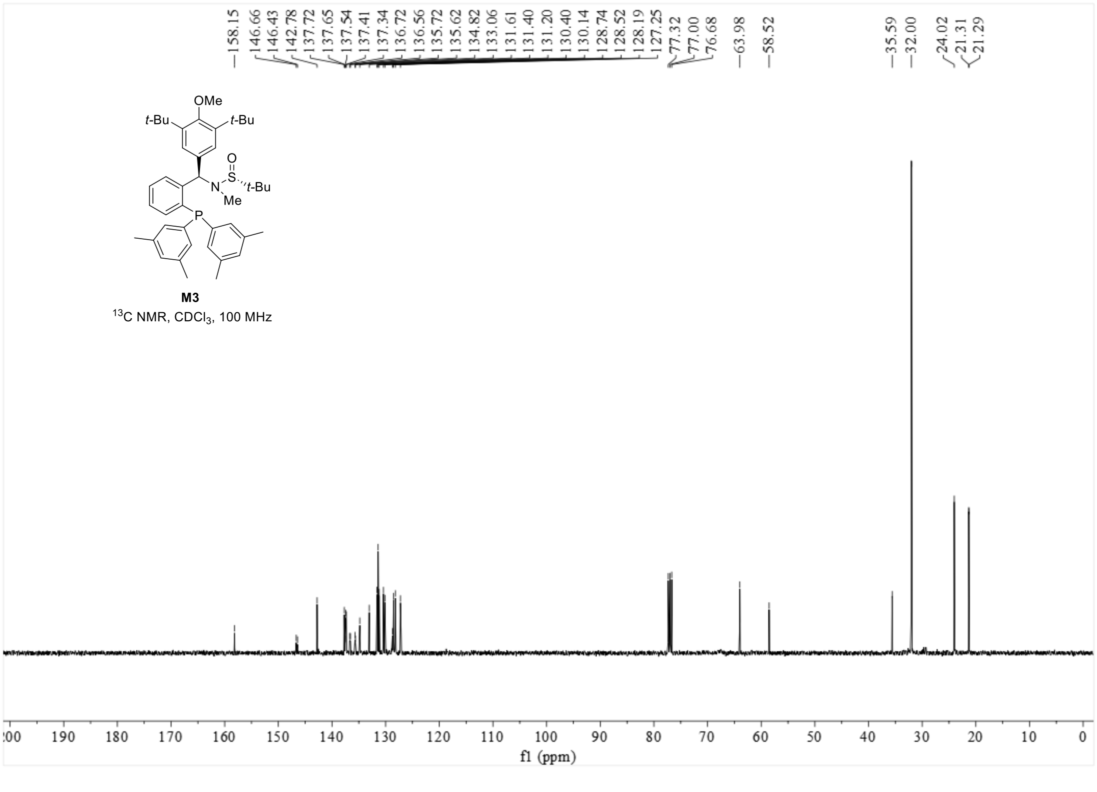

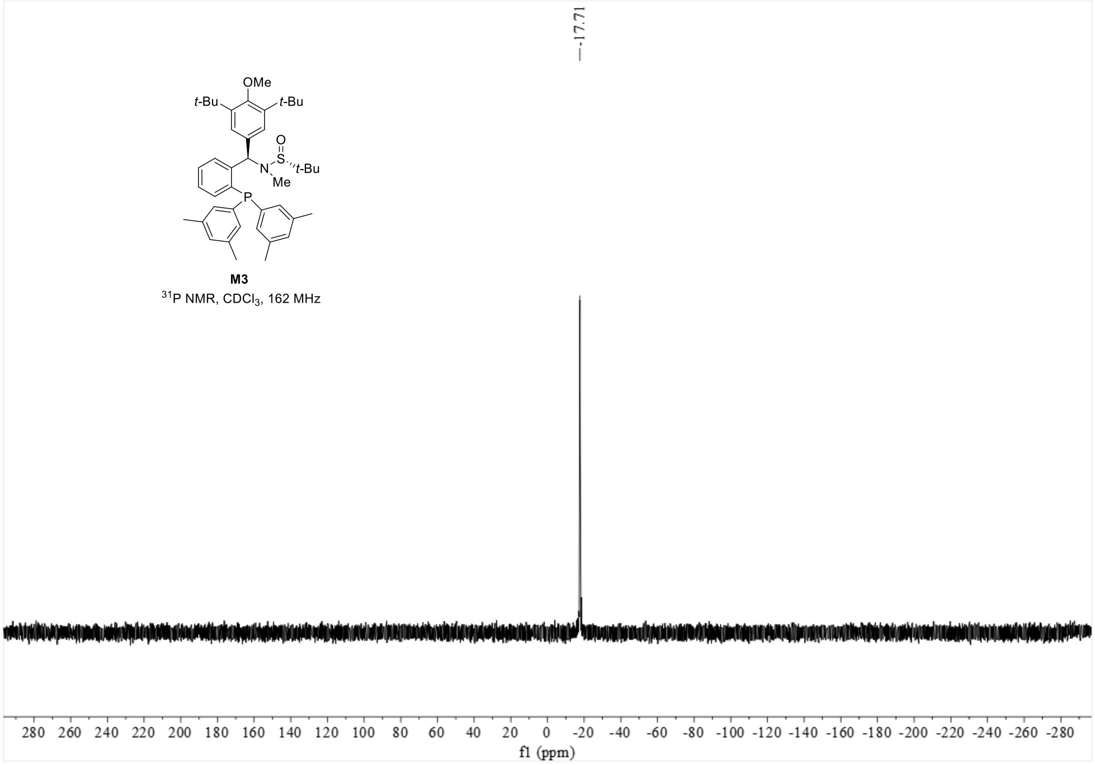

-17.71
OMe
t-Bu
t-Bu
O
S
N
t-Bu
Me
P
M3
31P NMR, CDCl3, 162 MHz
280 260 240 220 200 180 160 140 120 100 80 60 40 20 0 -20 -40 -60 -80 -100 -120 -140 -160 -180 -200 -220 -240 -260 -280
f1 (ppm)

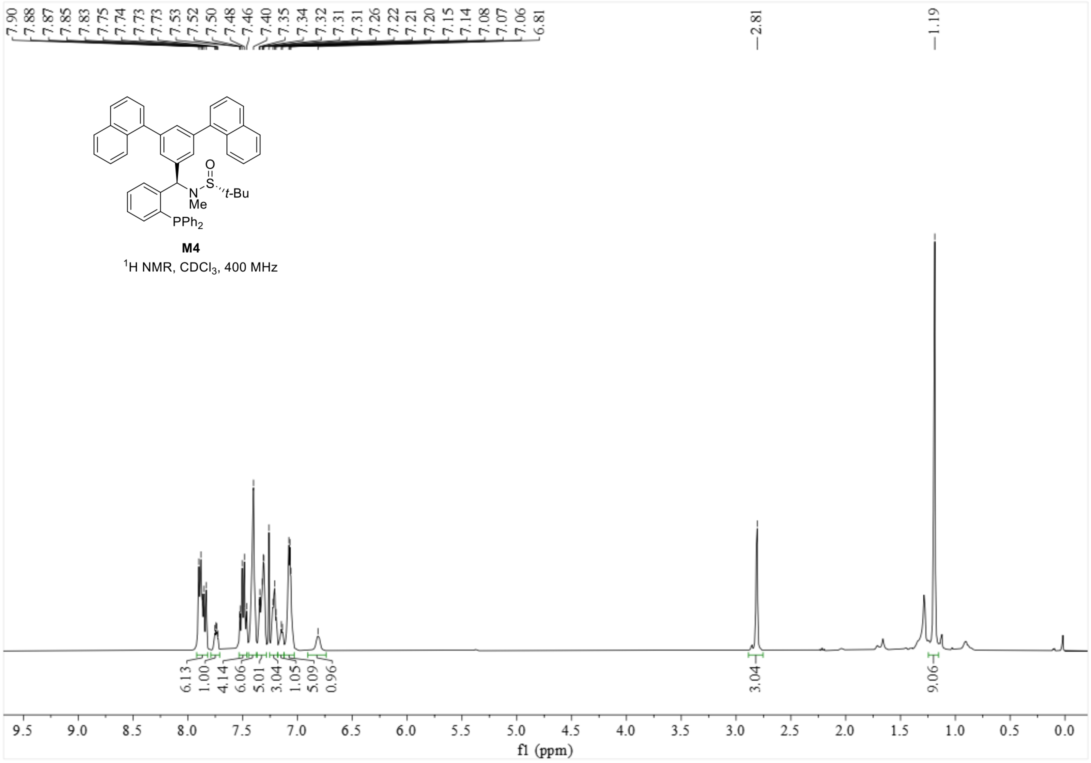

7.90 7.88 7.87 7.85 7.83 7.75 7.74 7.73 7.73 7.53 7.52 7.50 7.48 7.46 7.40 7.35 7.34 7.32 7.31 7.31 7.26 7.22 7.21 7.20 7.15 7.14 7.08 7.07 7.06 6.81
2.81
1.19
O
S
N
t-Bu
Me
PPh2
M4
1H NMR, CDCl3, 400 MHz
6.13 1.00 4.14 6.06 5.01 3.04 1.05 5.09 0.96
3.04
9.06
9.5 9.0 8.5 8.0 7.5 7.0 6.5 6.0 5.5 5.0 4.5 4.0 3.5 3.0 2.5 2.0 1.5 1.0 0.5 0.0
f1 (ppm)

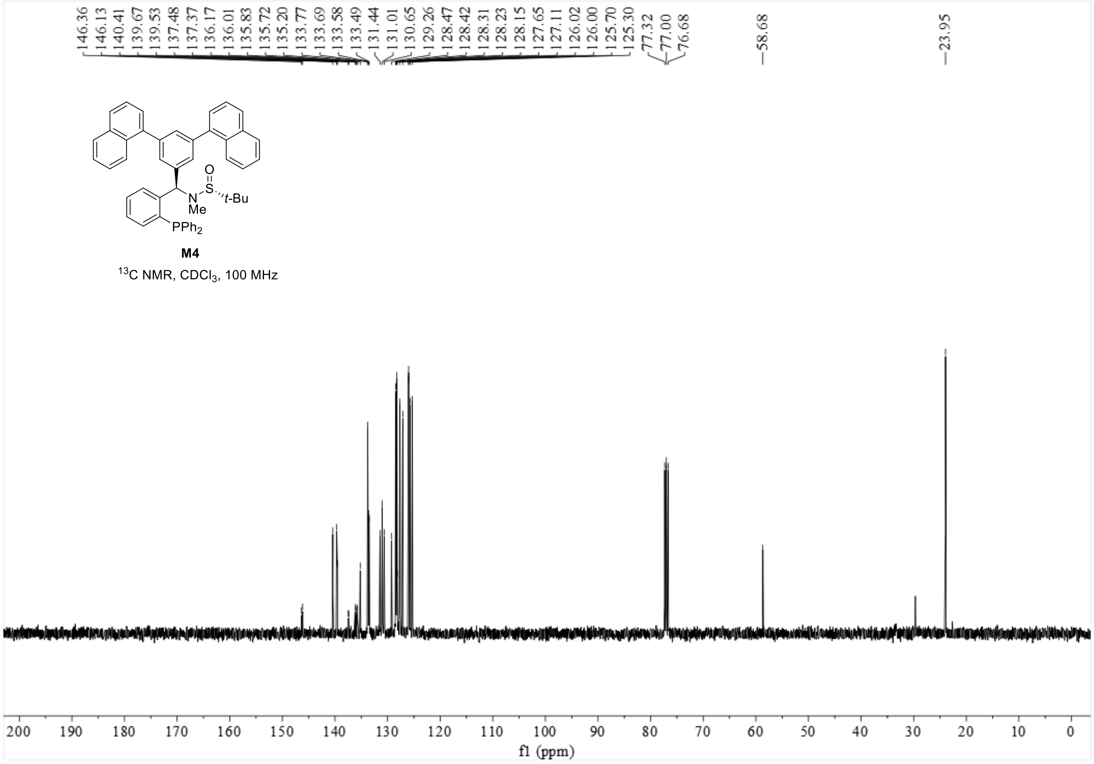
146.36
146.13
140.41
139.67
139.53
137.48
137.37
136.17
136.01
135.83
135.72
135.20
133.77
133.69
133.58
133.49
131.44
131.01
130.65
129.26
128.47
128.42
128.31
128.23
128.15
127.65
127.11
126.02
126.00
125.70
125.30
77.32
77.00
76.68
58.68
23.95
M4
13C NMR, CDCl3, 100 MHz
f1 (ppm)

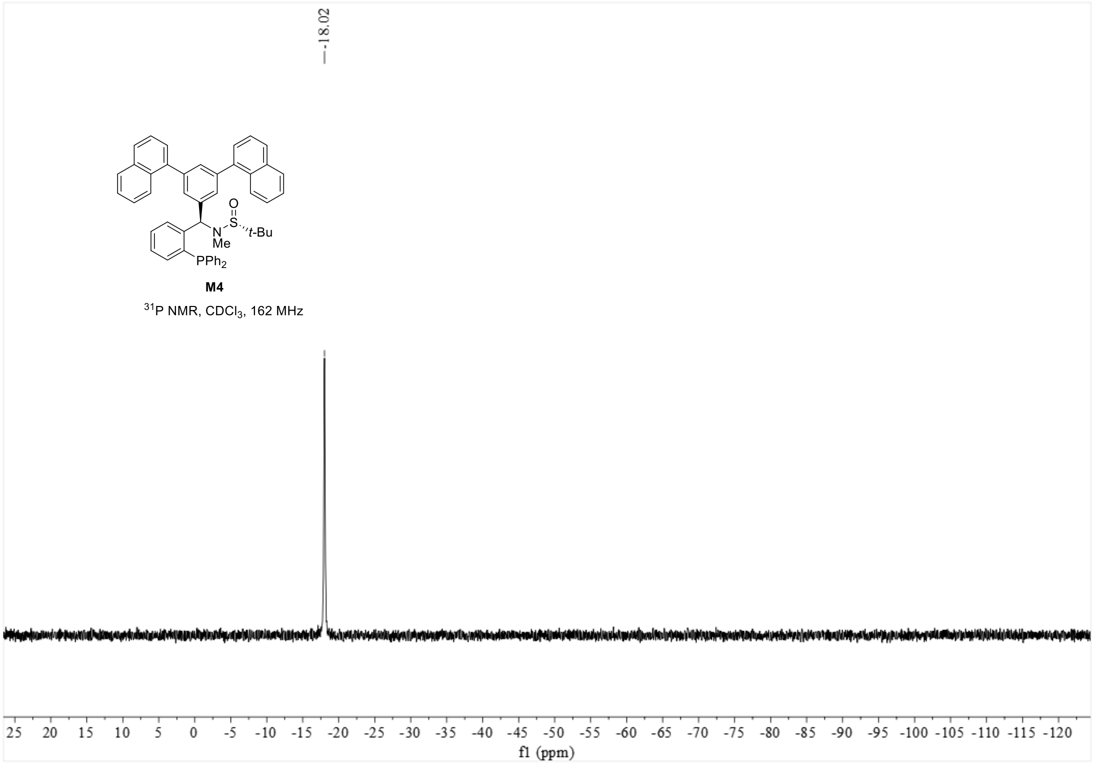
-18.02
M4
31P NMR, CDCl3, 162 MHz
f1 (ppm)

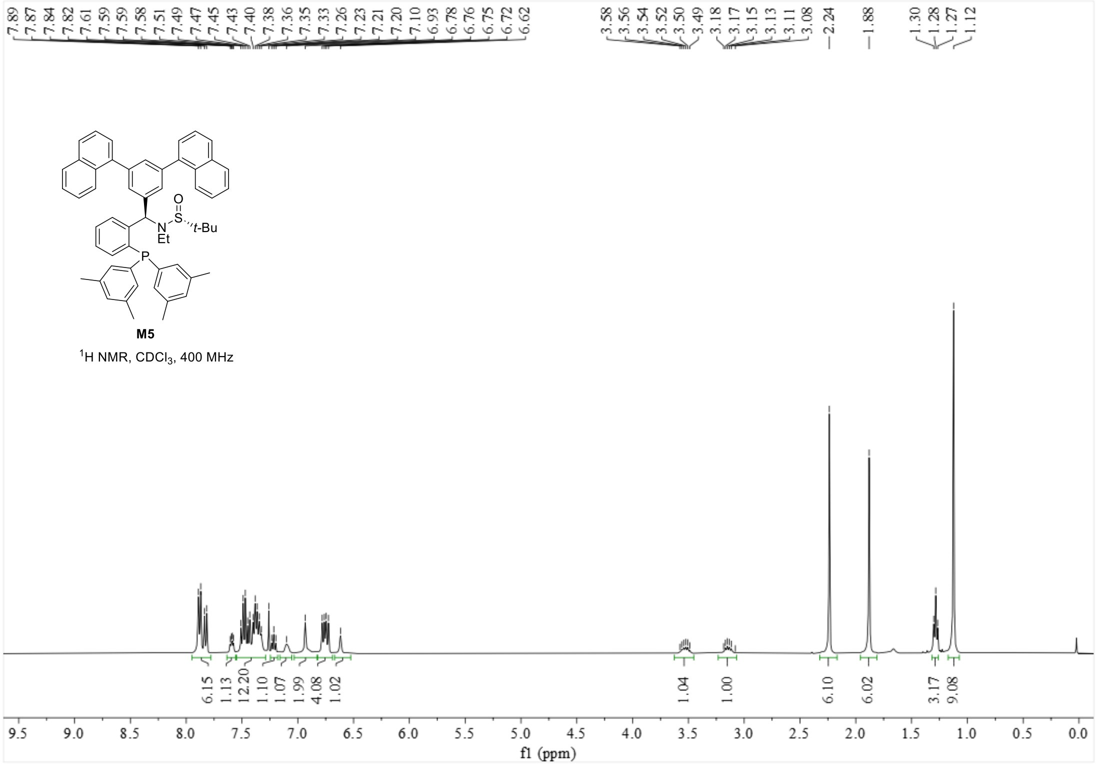
M5
¹H NMR, CDCl₃, 400 MHz
f1 (ppm)

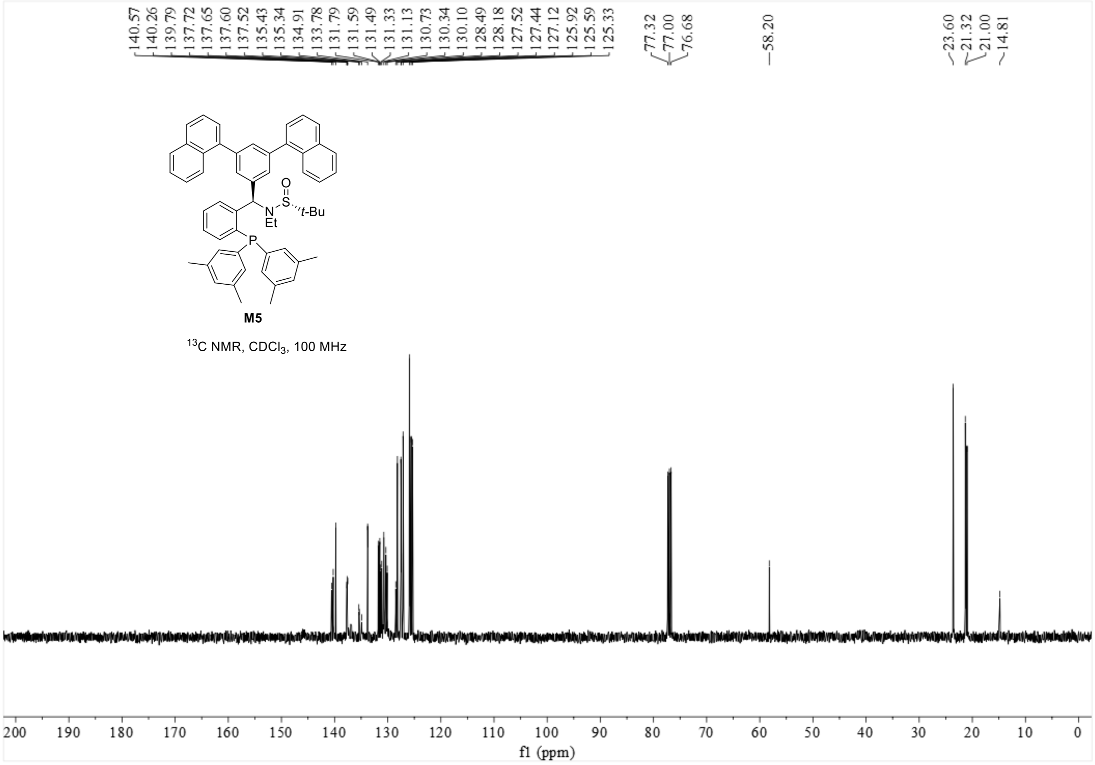
M5
¹³C NMR, CDCl₃, 100 MHz
f1 (ppm)

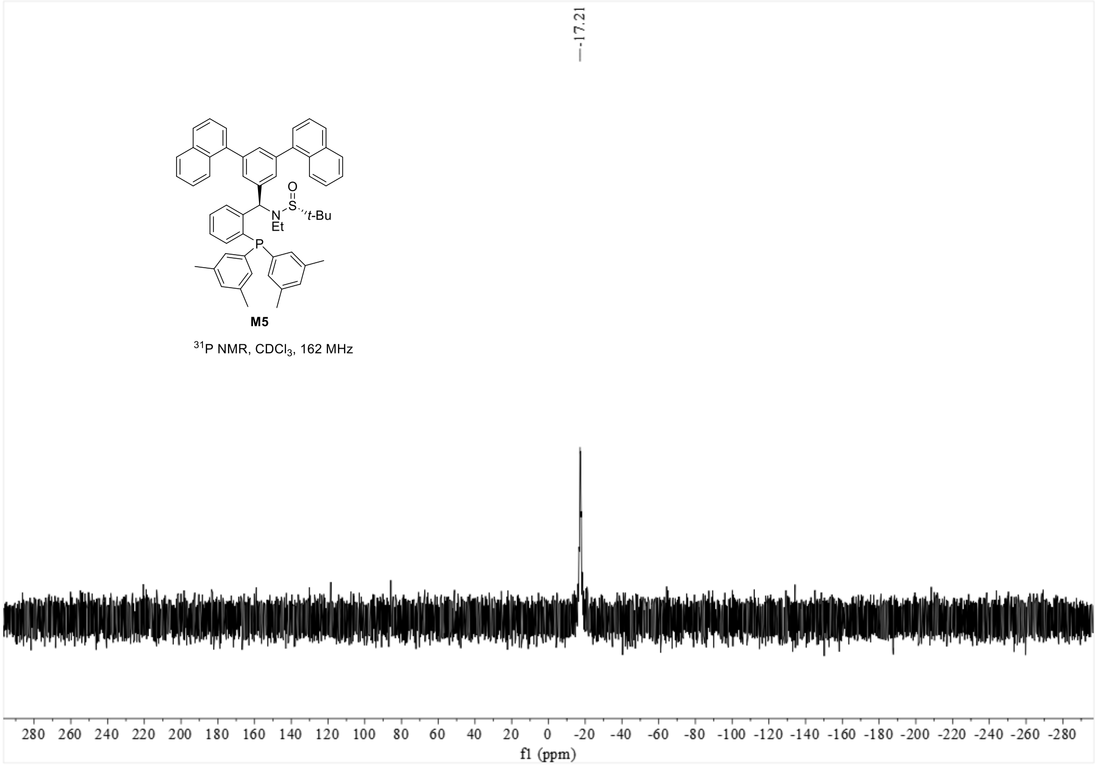

-17.21
M5
31P NMR, CDCl3, 162 MHz
f1 (ppm)

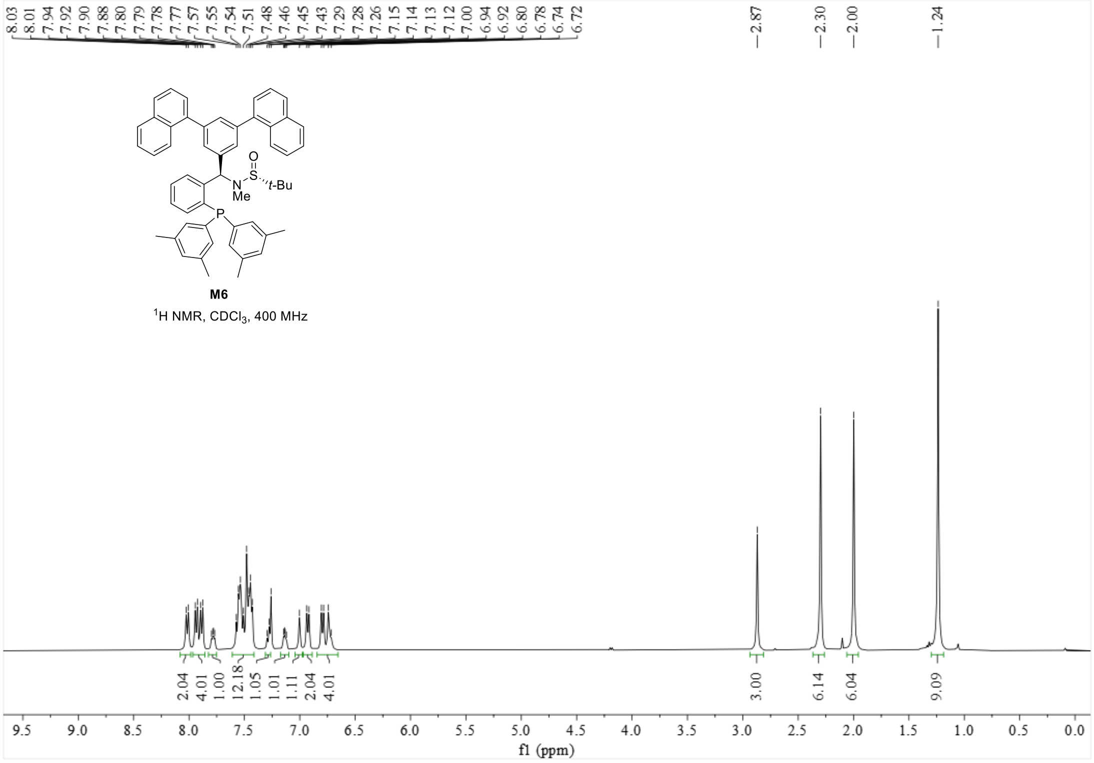

8.03
8.01
7.94
7.92
7.90
7.88
7.80
7.79
7.78
7.77
7.57
7.55
7.54
7.51
7.48
7.46
7.45
7.43
7.29
7.28
7.26
7.15
7.14
7.13
7.12
7.00
6.94
6.92
6.80
6.78
6.74
6.72
2.87
2.30
2.00
1.24
M6
1H NMR, CDCl3, 400 MHz
2.04
4.01
1.00
12.18
1.05
1.01
1.11
2.04
4.01
3.00
6.14
6.04
9.09
f1 (ppm)

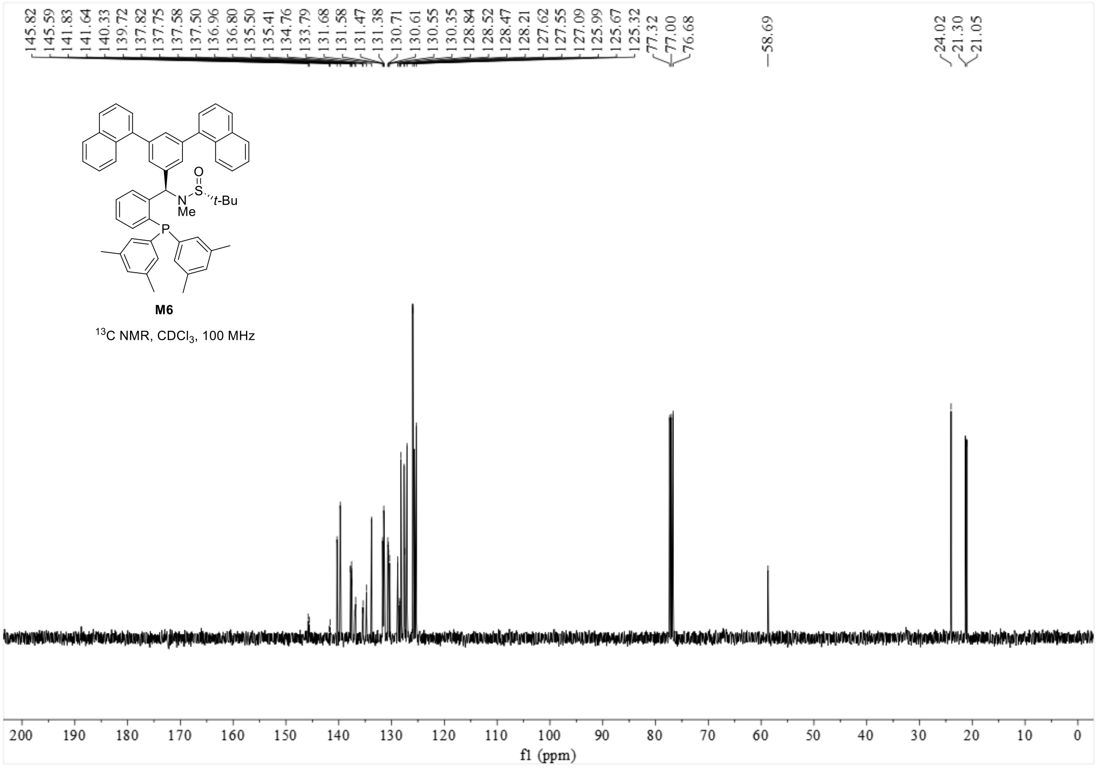
145.82
145.59
141.83
141.64
140.33
139.72
137.82
137.75
137.58
137.50
136.96
136.80
135.50
135.41
134.76
133.79
131.68
131.58
131.47
131.38
130.71
130.61
130.55
130.35
128.84
128.52
128.47
128.21
127.62
127.55
127.09
125.99
125.67
125.32
77.32
77.00
76.68
58.69
24.02
21.30
21.05
M6
^{13}C NMR, CDCl$_3$, 100 MHz
fl (ppm)

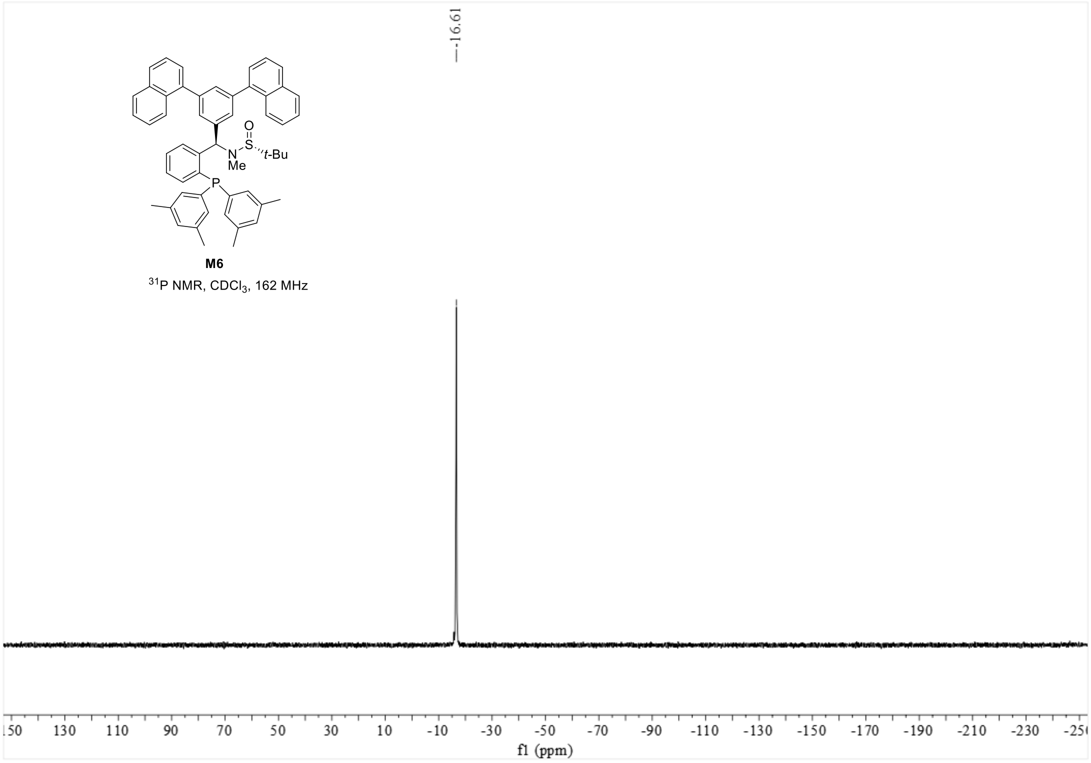
-16.61
M6
^{31}P NMR, CDCl$_3$, 162 MHz
fl (ppm)

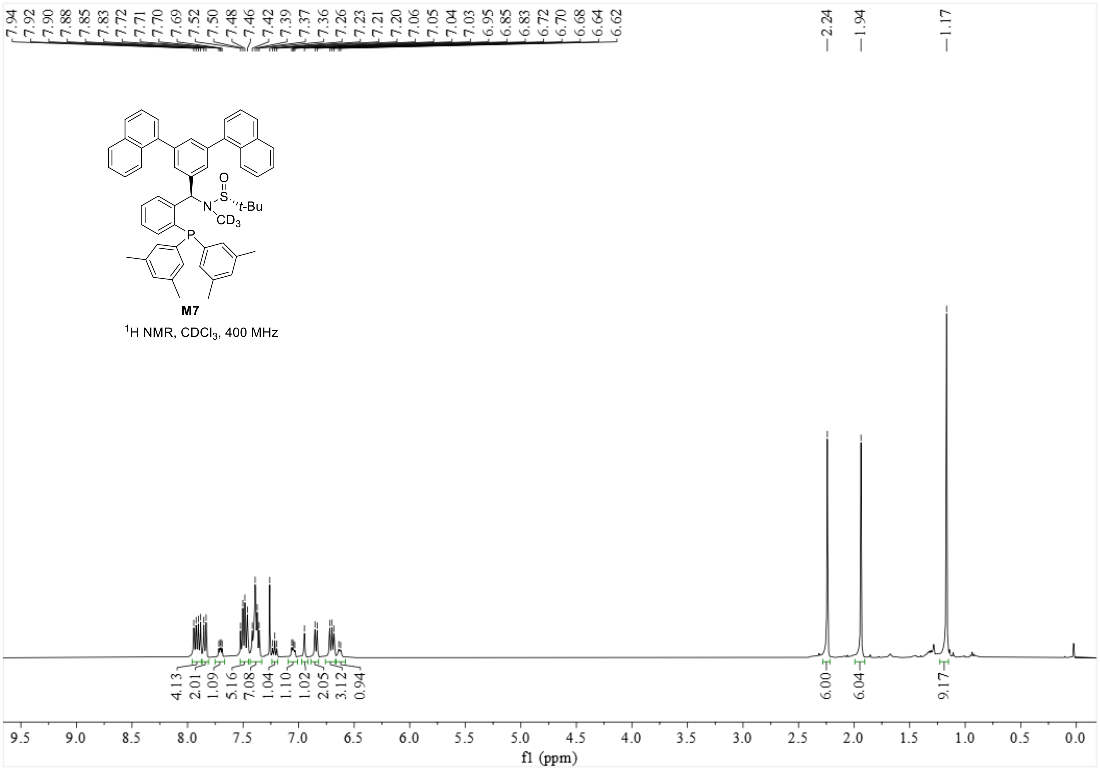

M7
^{1}H NMR, $CDCl_3$, 400 MHz
f1 (ppm)

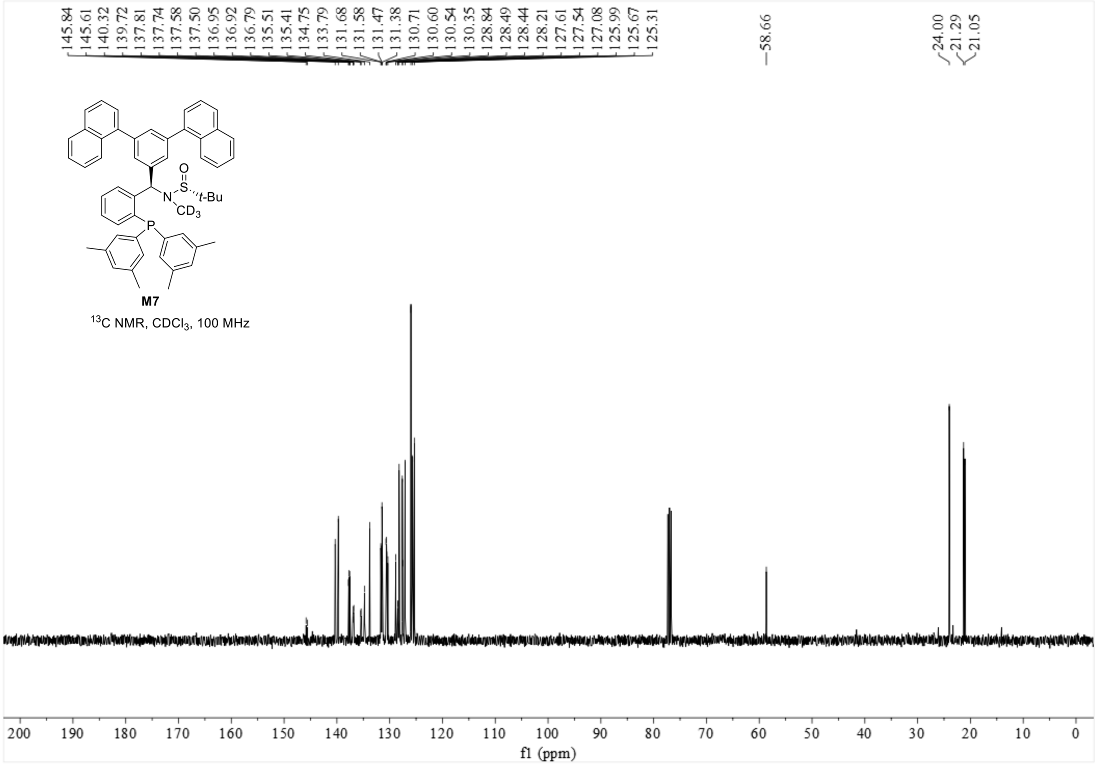

M7
^{13}C NMR, $CDCl_3$, 100 MHz
f1 (ppm)

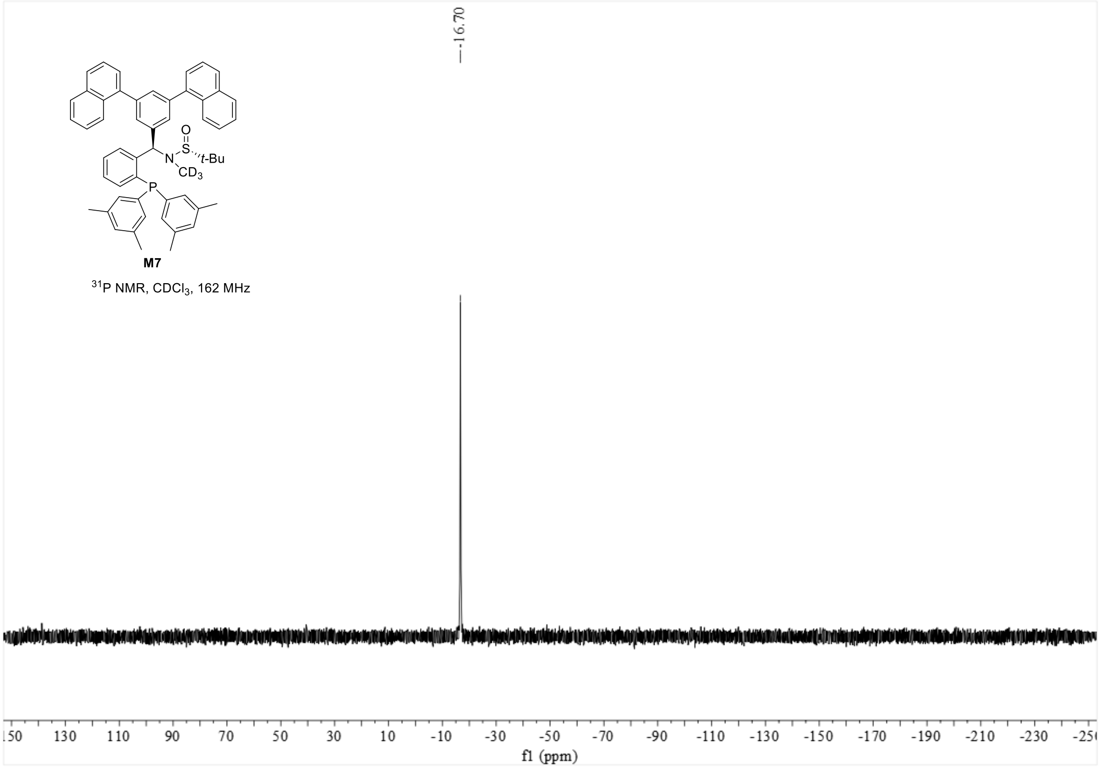

-16.70
M7
^{31}P NMR, CDCl$_3$, 162 MHz
f1 (ppm)

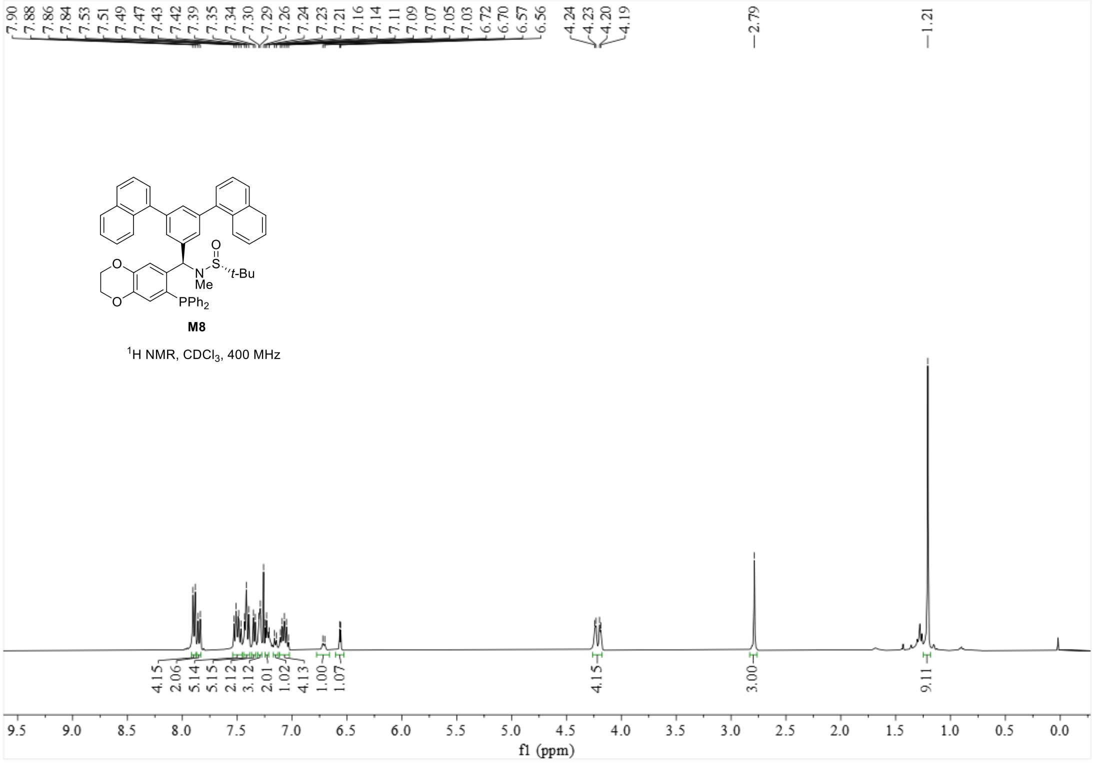

7.90 7.88 7.86 7.84 7.53 7.51 7.49 7.47 7.43 7.42 7.39 7.35 7.34 7.30 7.29 7.26 7.24 7.23 7.21 7.16 7.14 7.11 7.09 7.07 7.05 7.03 6.72 6.70 6.57 6.56
4.24 4.23 4.20 4.19
2.79
1.21
M8
^{1}H NMR, CDCl$_3$, 400 MHz
4.15 2.06 5.14 5.15 2.12 3.12 2.01 1.02 4.13 1.00 1.07
4.15
3.00
9.11
f1 (ppm)

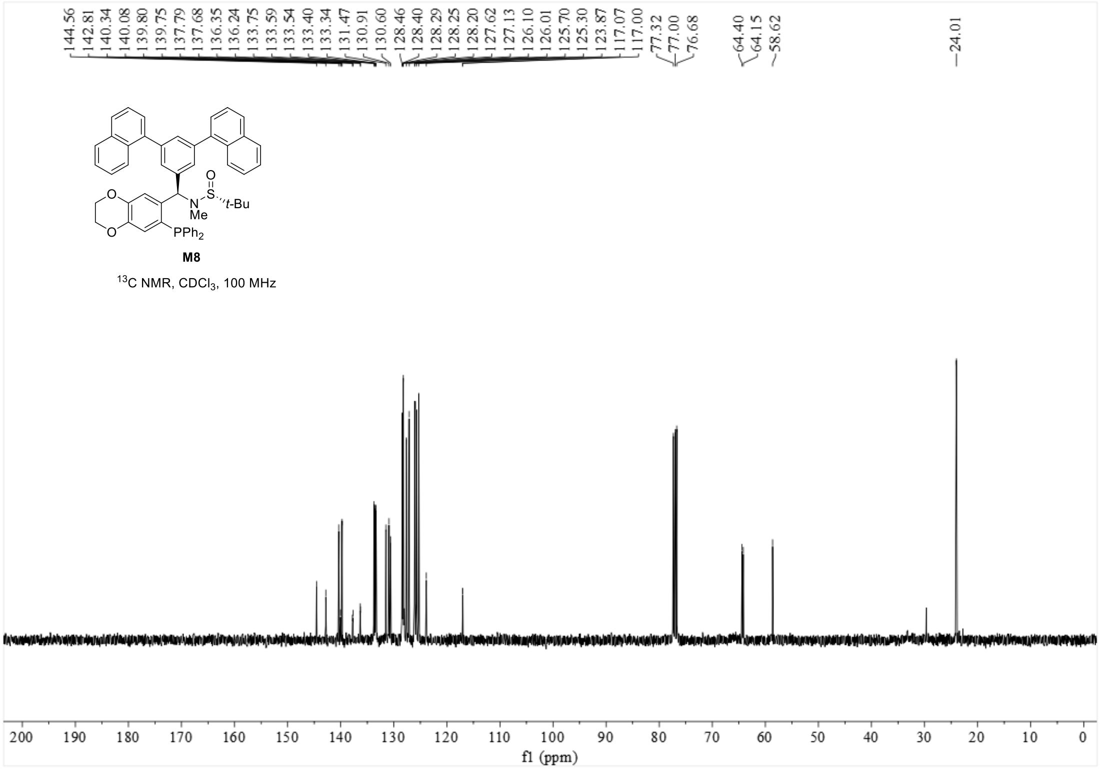
144.56
142.81
140.34
140.08
139.80
139.75
137.79
137.68
136.35
136.24
133.75
133.59
133.54
133.40
133.34
131.47
130.91
130.60
128.46
128.40
128.29
128.25
128.20
127.62
127.13
126.10
126.01
125.70
125.30
123.87
117.07
117.00
77.32
77.00
76.68
64.40
64.15
58.62
24.01
M8
13C NMR, CDCl3, 100 MHz
f1 (ppm)

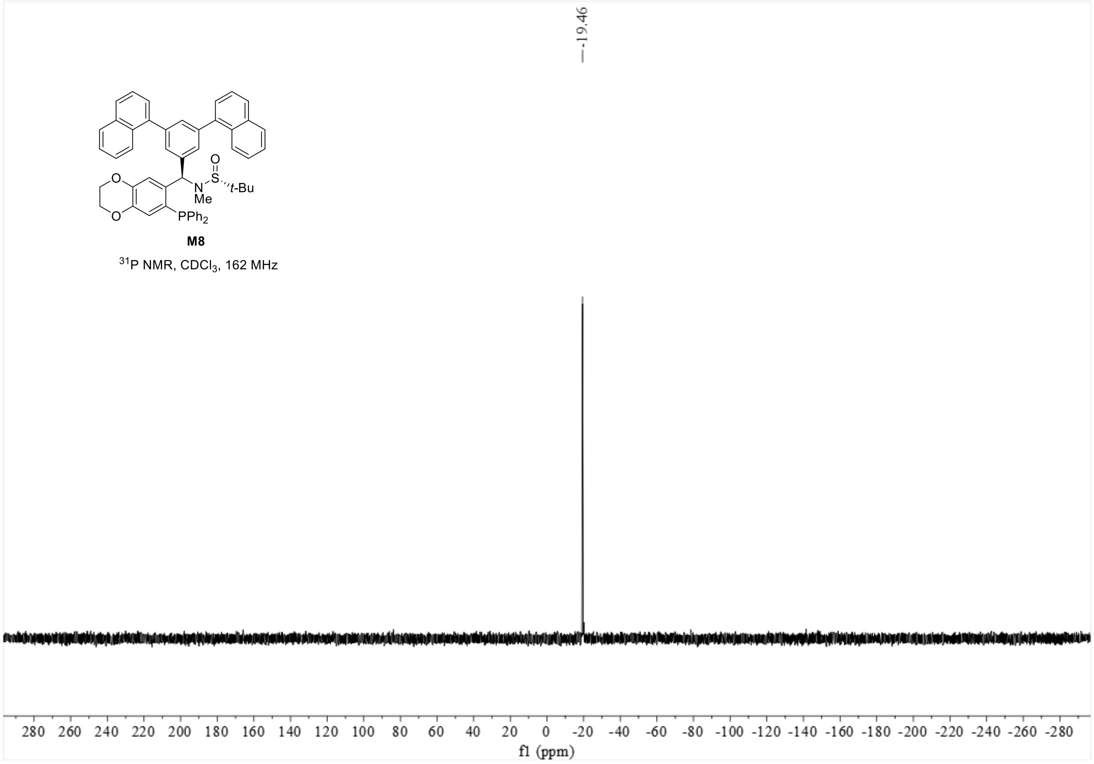
-19.46
M8
31P NMR, CDCl3, 162 MHz
f1 (ppm)

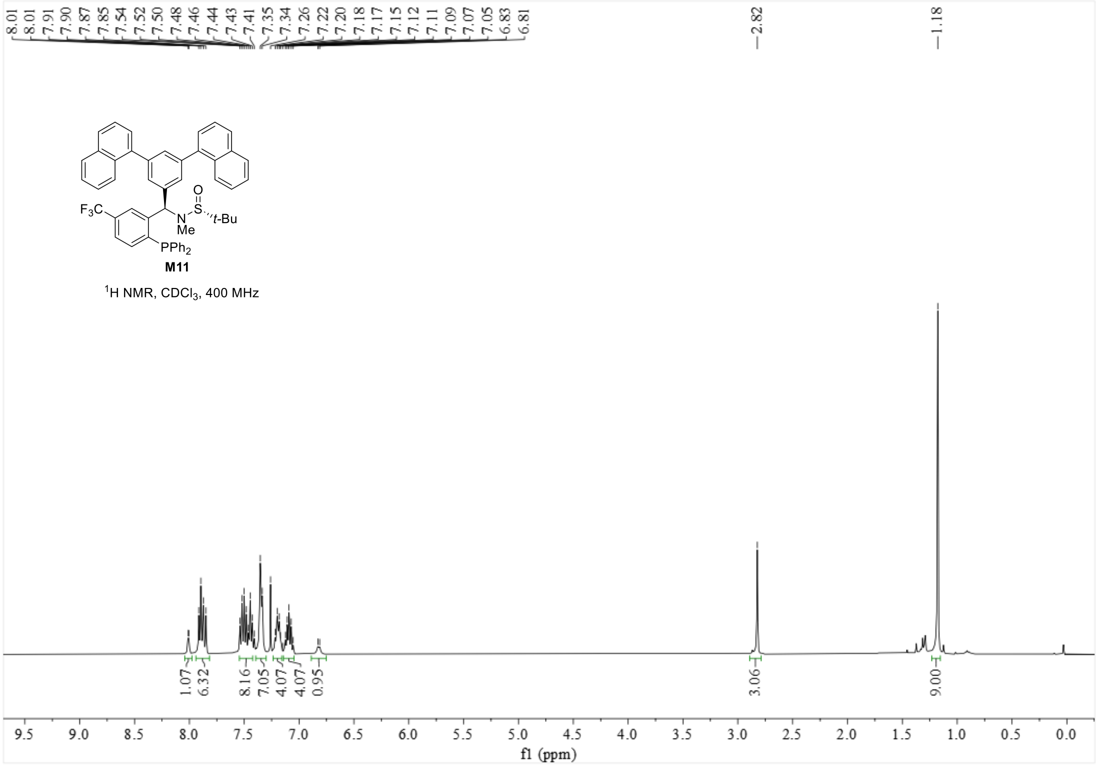

8.01 8.01 7.91 7.90 7.87 7.85 7.54 7.52 7.50 7.48 7.46 7.44 7.43 7.41 7.35 7.34 7.26 7.22 7.20 7.18 7.17 7.15 7.12 7.11 7.09 7.07 7.05 6.83 6.81
2.82
1.18
F3C
N
Me
S
O
t-Bu
PPh2
M11
1H NMR, CDCl3, 400 MHz
1.07 6.32 8.16 7.05 4.07 4.07 0.95
3.06
9.00
9.5 9.0 8.5 8.0 7.5 7.0 6.5 6.0 5.5 5.0 4.5 4.0 3.5 3.0 2.5 2.0 1.5 1.0 0.5 0.0
f1 (ppm)

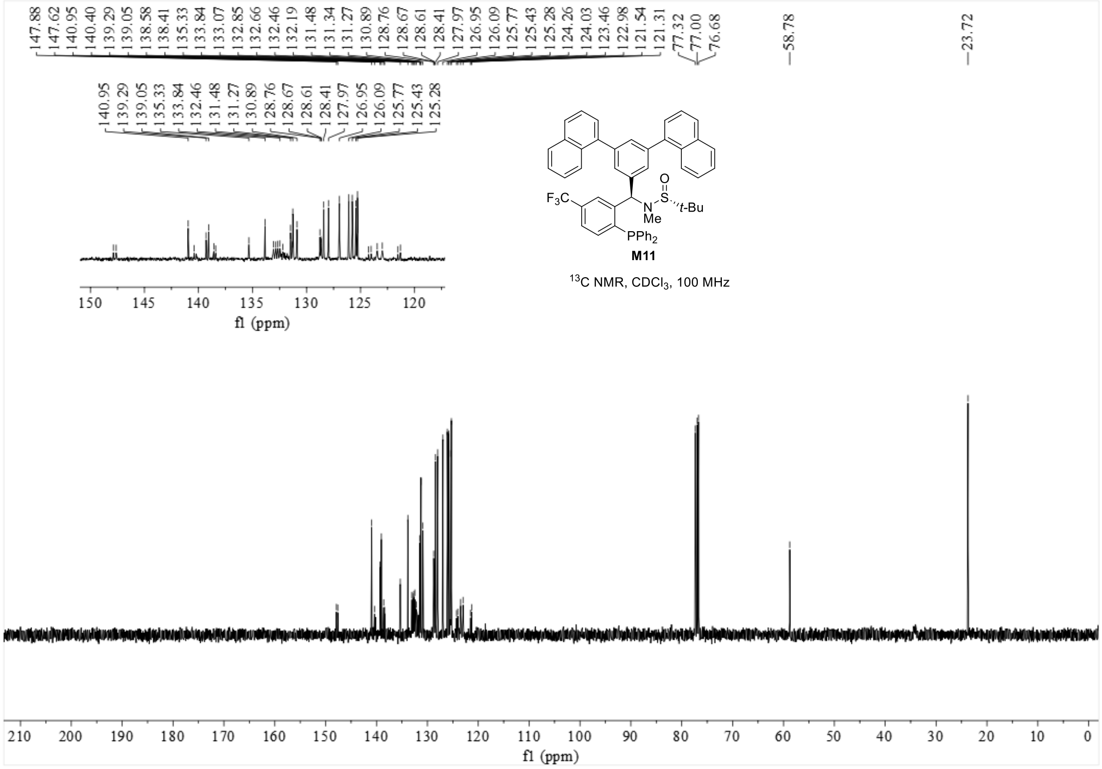

147.88 147.62 140.95 140.40 139.29 139.05 138.58 138.41 135.33 133.84 133.07 132.85 132.66 132.46 132.19 131.48 131.34 131.27 130.89 128.76 128.67 128.61 128.41 127.97 126.95 126.09 125.77 125.43 125.28 124.26 124.03 123.46 122.98 121.54 121.31
77.32 77.00 76.68
58.78
23.72
140.95 139.29 139.05 135.33 133.84 132.46 131.48 131.27 130.89 128.76 128.67 128.61 128.41 127.97 126.95 126.09 125.77 125.43 125.28
150 145 140 135 130 125 120
f1 (ppm)
F3C
N
Me
S
O
t-Bu
PPh2
M11
13C NMR, CDCl3, 100 MHz
210 200 190 180 170 160 150 140 130 120 110 100 90 80 70 60 50 40 30 20 10 0
f1 (ppm)

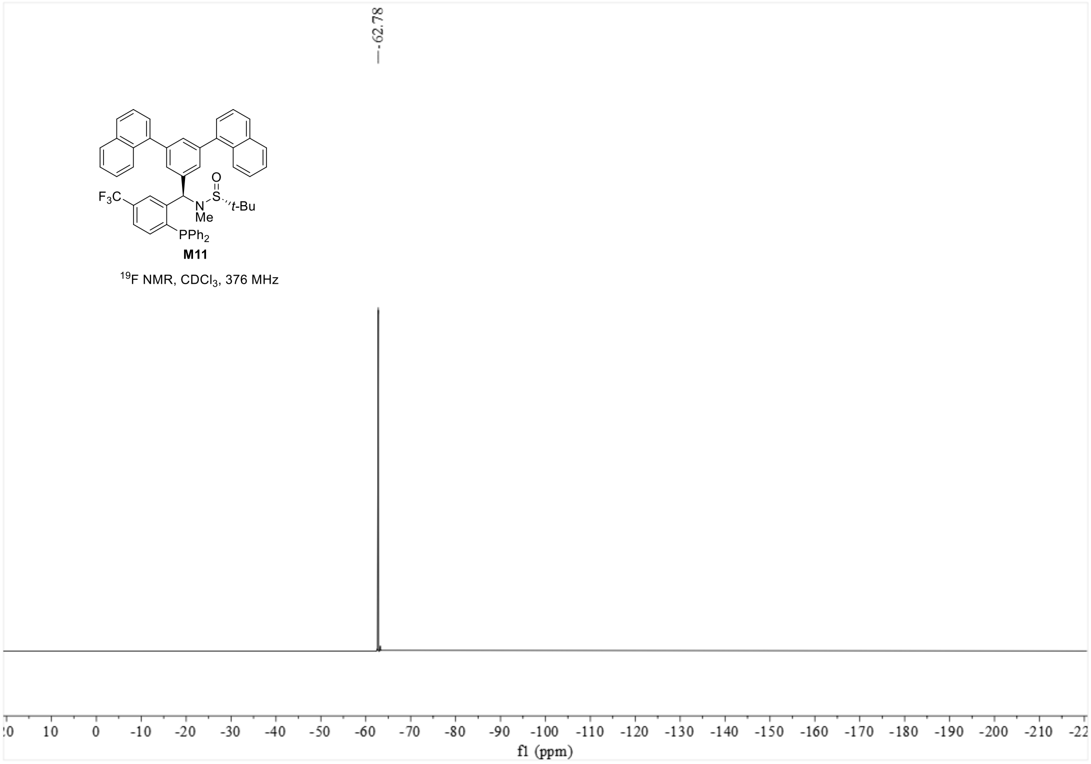
-62.78
F3C
N
S
O
t-Bu
Me
PPh2
M11
19F NMR, CDCl3, 376 MHz
f1 (ppm)

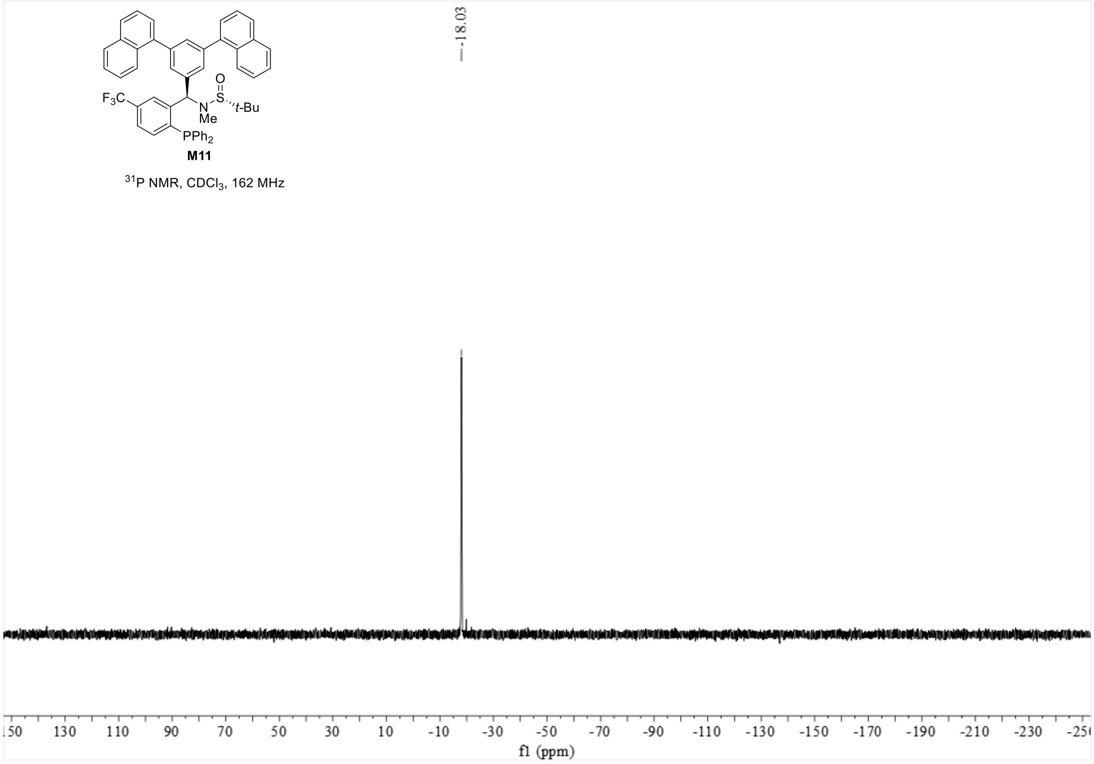
-18.03
F3C
N
S
O
t-Bu
Me
PPh2
M11
31P NMR, CDCl3, 162 MHz
f1 (ppm)

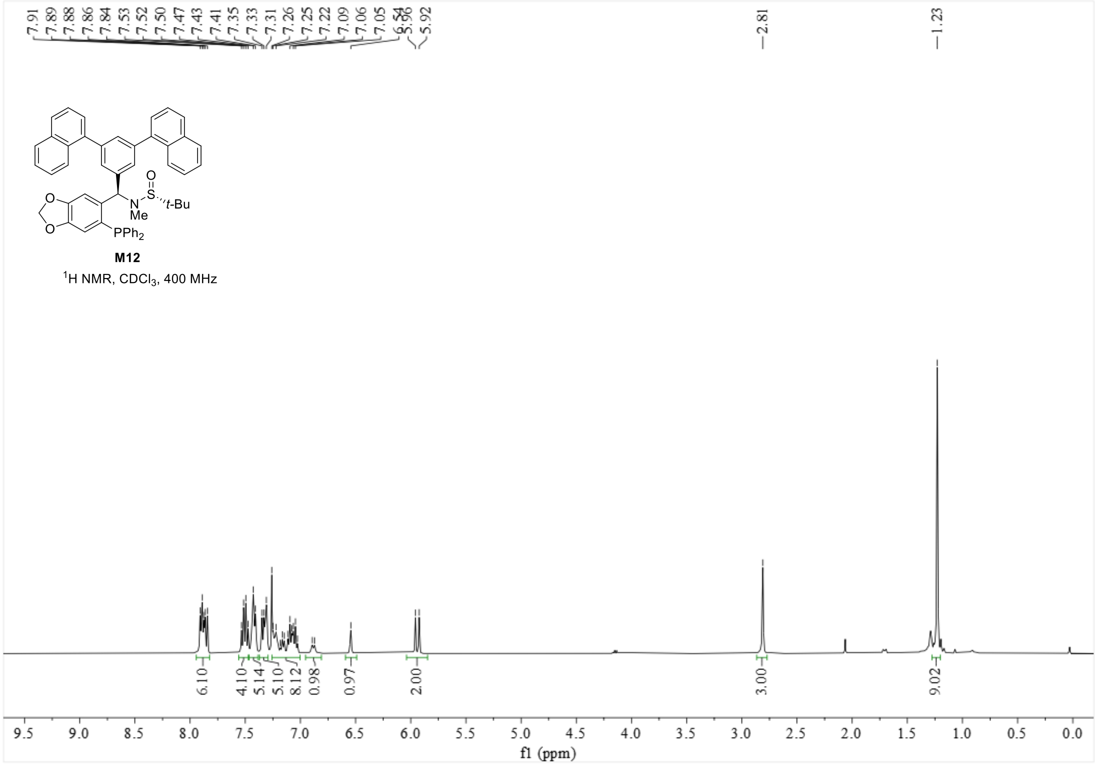
7.91
7.89
7.88
7.86
7.84
7.53
7.52
7.50
7.47
7.43
7.41
7.35
7.33
7.31
7.26
7.25
7.22
7.09
7.06
7.05
5.92
2.81
1.23
M12
1H NMR, CDCl3, 400 MHz
6.10
4.10
5.14
5.10
8.12
0.98
0.97
2.00
3.00
9.02
f1 (ppm)

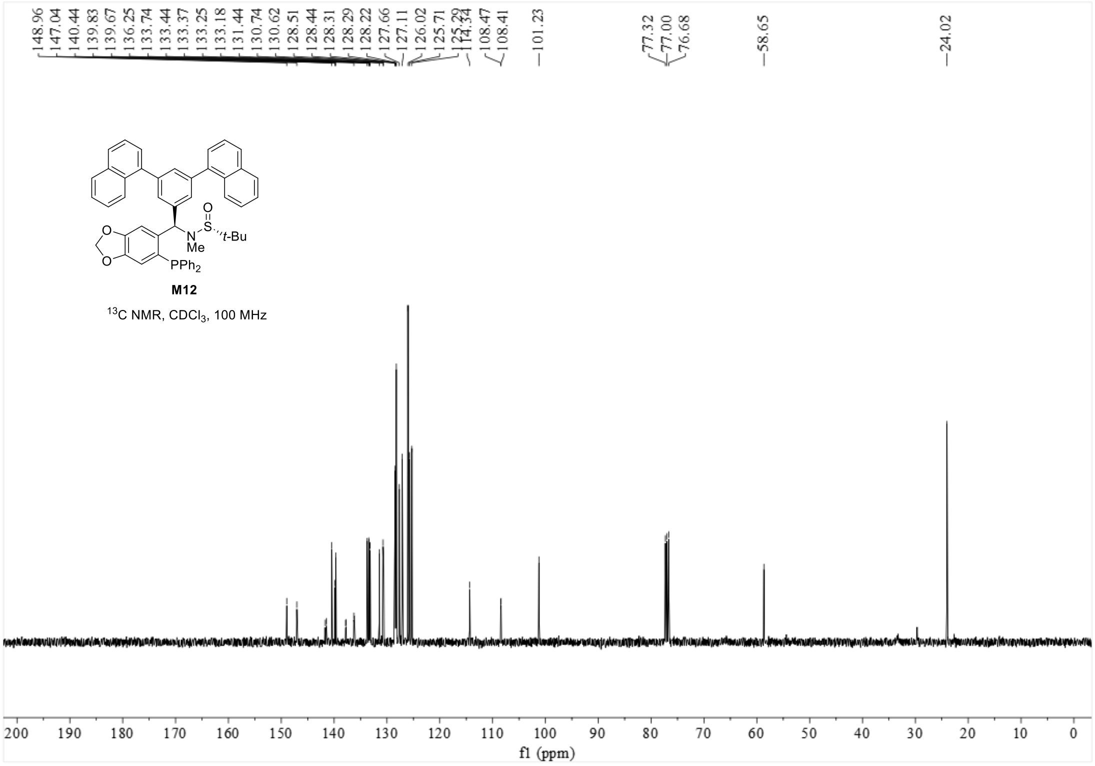
148.96
147.04
140.44
139.83
139.67
136.25
133.74
133.44
133.37
133.25
133.18
131.44
130.74
130.62
128.51
128.44
128.31
128.29
128.22
127.66
127.11
126.02
125.71
108.47
108.41
101.23
77.32
77.00
76.68
58.65
24.02
M12
13C NMR, CDCl3, 100 MHz
f1 (ppm)

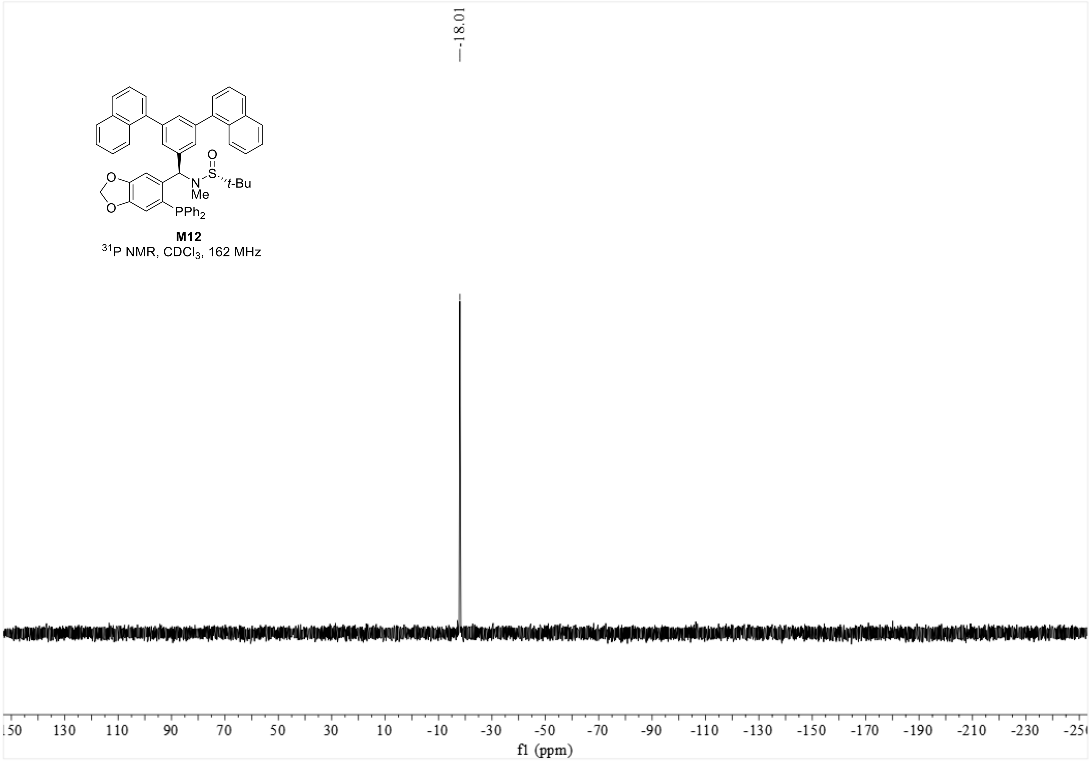

-18.01
M12
31P NMR, CDCl3, 162 MHz
f1 (ppm)

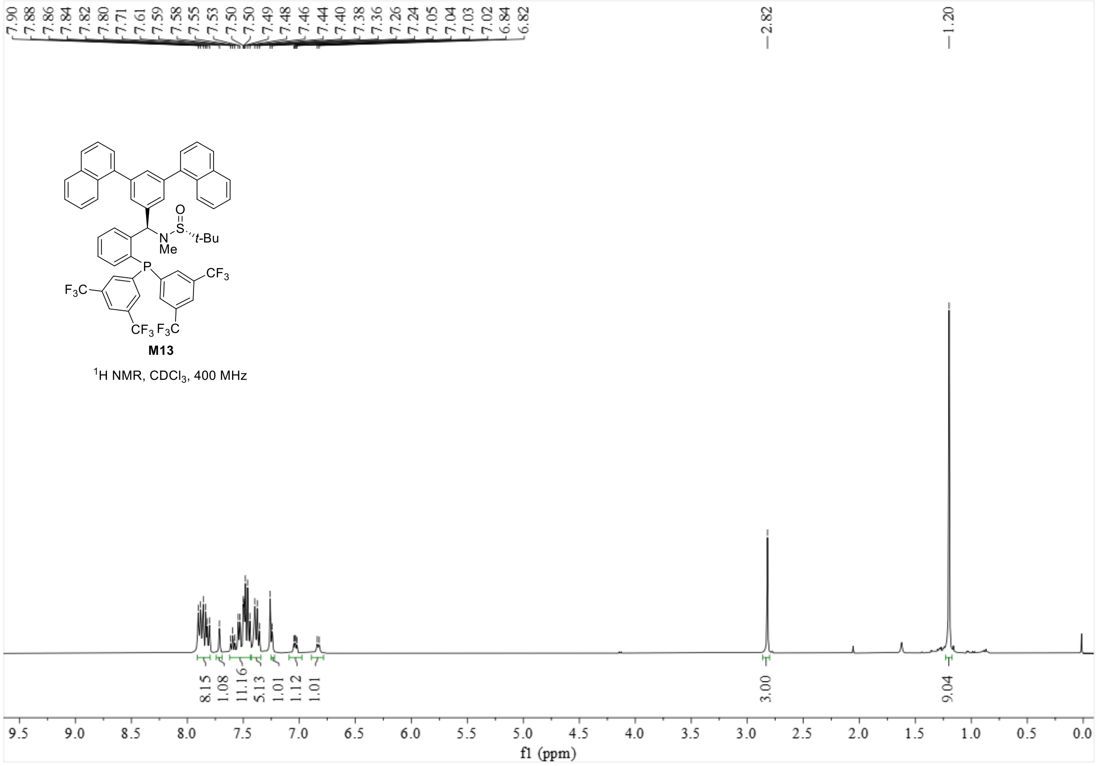

7.90 7.88 7.86 7.84 7.82 7.80 7.71 7.61 7.59 7.58 7.55 7.53 7.50 7.50 7.49 7.48 7.46 7.44 7.40 7.38 7.36 7.26 7.24 7.05 7.04 7.03 7.02 6.84 6.82
2.82
1.20
M13
1H NMR, CDCl3, 400 MHz
8.15 1.08 11.16 5.13 1.01 1.12 1.01
3.00
9.04
f1 (ppm)

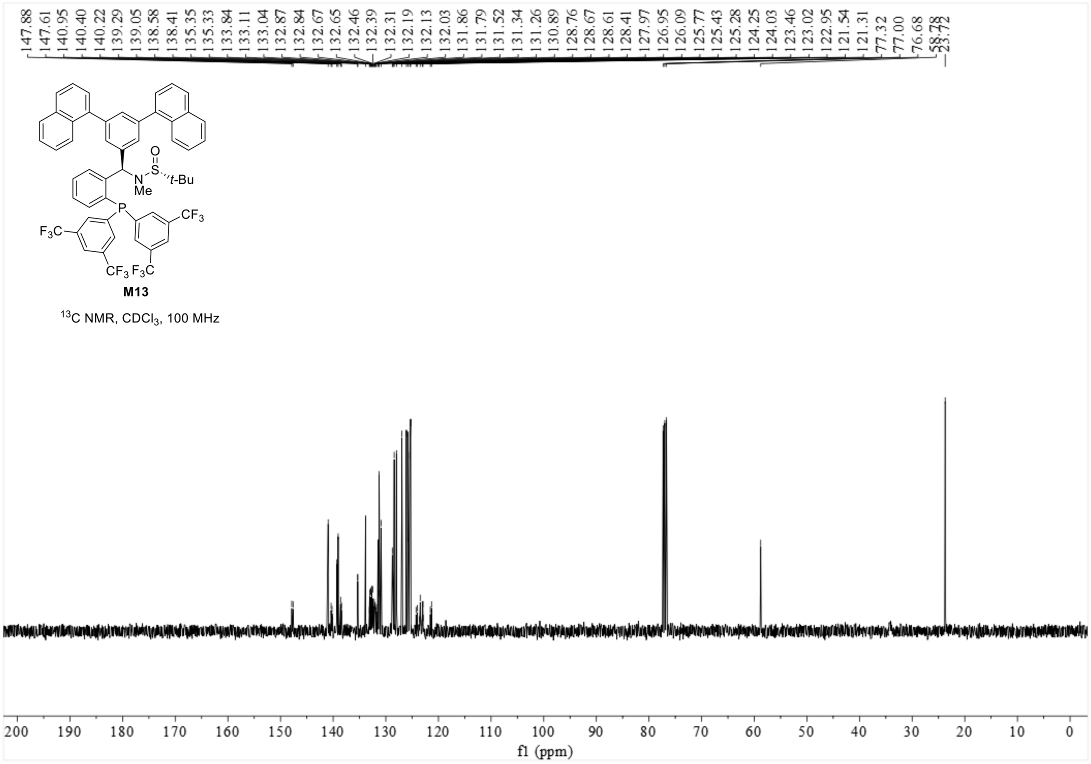
147.88
147.61
140.95
140.40
140.22
139.29
139.05
138.58
138.41
135.35
135.33
133.84
133.11
133.04
132.87
132.84
132.67
132.65
132.46
132.39
132.31
132.19
132.13
132.03
131.86
131.79
131.52
131.34
131.26
130.89
128.76
128.67
128.61
128.41
127.97
126.95
126.09
125.77
125.43
125.28
124.25
124.03
123.46
123.02
122.95
121.54
121.31
77.32
77.00
76.68
58.78
23.72
t-Bu
Me
F3C
CF3
CF3 F3C
M13
13C NMR, CDCl3, 100 MHz
f1 (ppm)

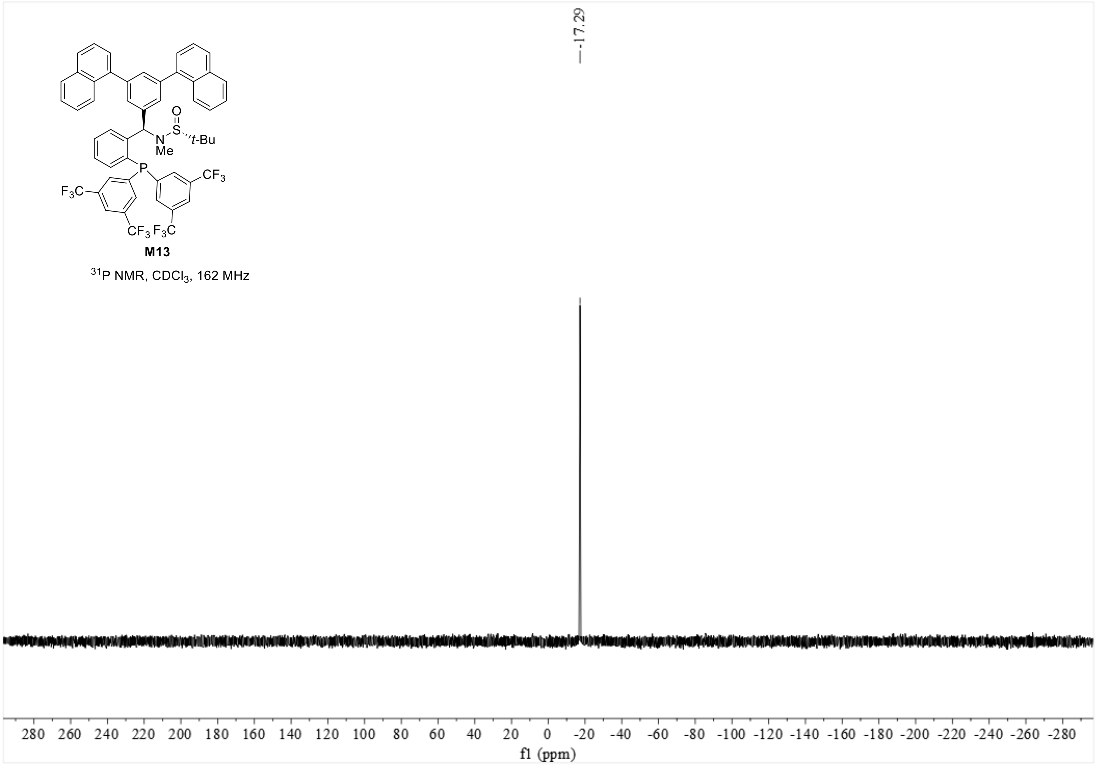
-17.29
t-Bu
Me
F3C
CF3
CF3 F3C
M13
31P NMR, CDCl3, 162 MHz
f1 (ppm)

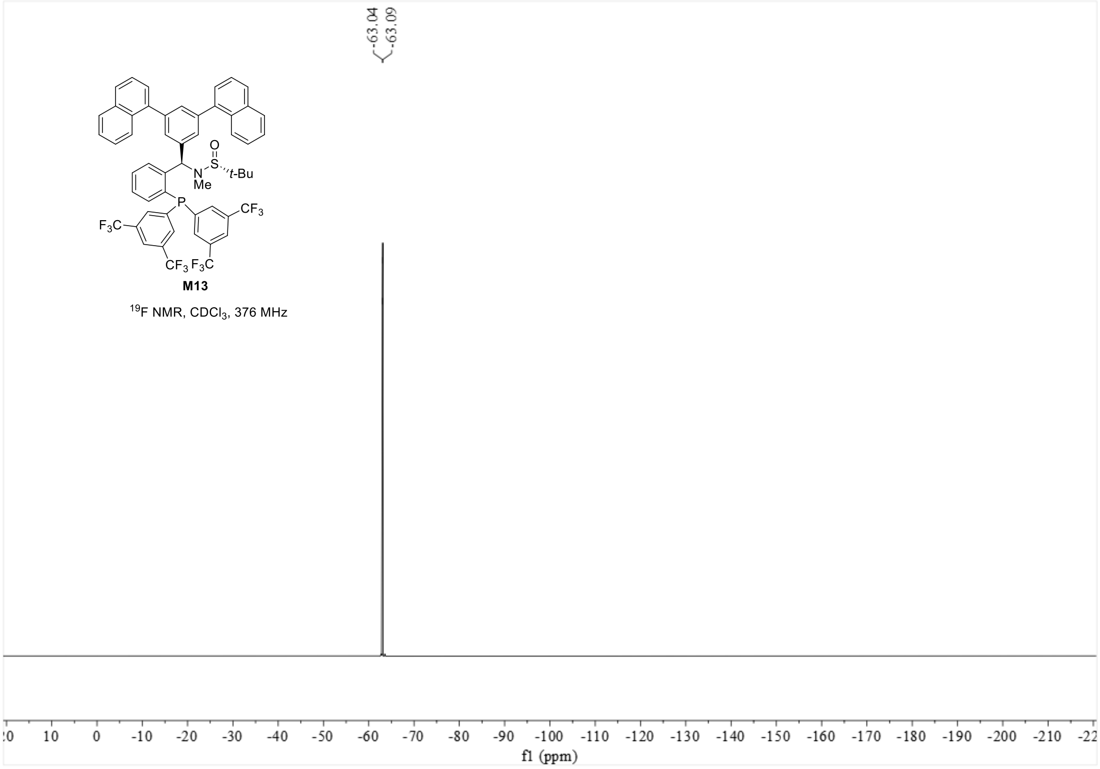
-63.04
-63.09
M13
^{19}F NMR, CDCl$_3$, 376 MHz
f1 (ppm)

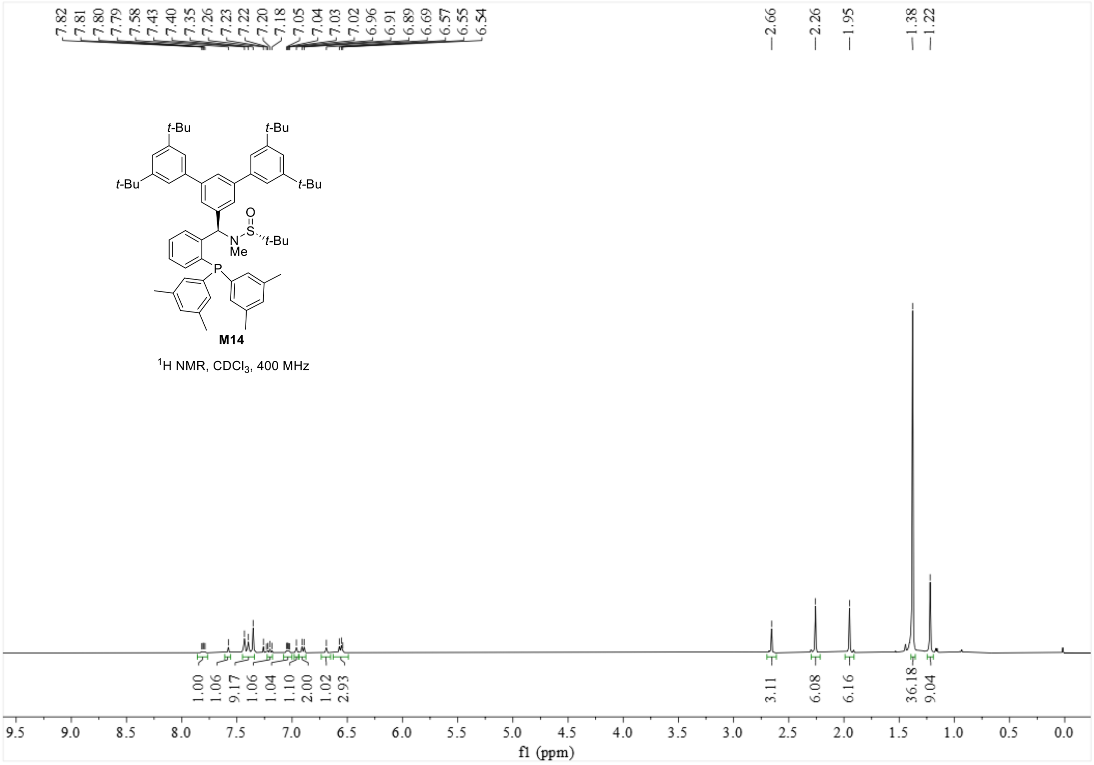
7.82
7.81
7.80
7.79
7.58
7.43
7.40
7.35
7.26
7.23
7.22
7.20
7.18
7.05
7.04
7.03
7.02
6.96
6.91
6.89
6.69
6.57
6.55
6.54
2.66
2.26
1.95
1.38
1.22
M14
^{1}H NMR, CDCl$_3$, 400 MHz
1.00
1.06
9.17
1.06
1.04
1.10
2.00
1.02
2.93
3.11
6.08
6.16
36.18
9.04
f1 (ppm)

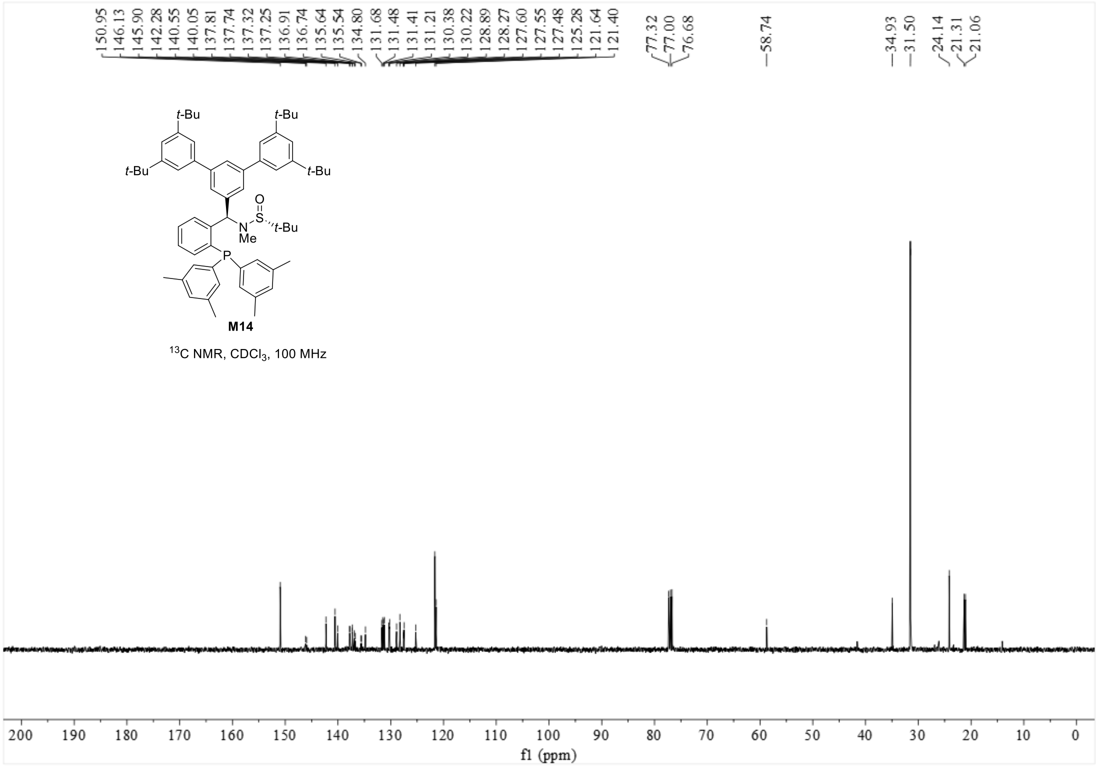
150.95
146.13
145.90
142.28
140.55
140.05
137.81
137.74
137.32
137.25
136.91
136.74
135.64
135.54
134.80
131.68
131.48
131.41
131.21
130.38
130.22
128.89
128.27
127.60
127.55
127.48
125.28
121.64
121.40
77.32
77.00
76.68
58.74
34.93
31.50
24.14
21.31
21.06
t-Bu
t-Bu
t-Bu
t-Bu
O
S
t-Bu
N
Me
P
M14
13C NMR, CDCl3, 100 MHz
200 190 180 170 160 150 140 130 120 110 100 90 80 70 60 50 40 30 20 10 0
f1 (ppm)

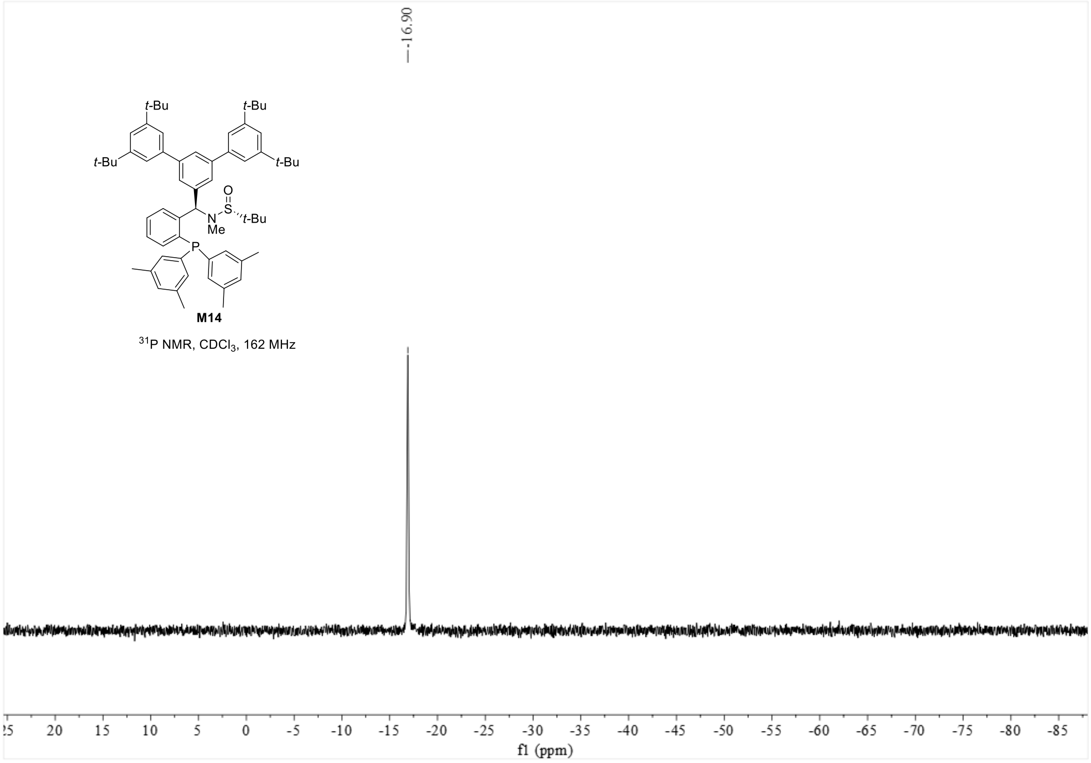
-16.90
t-Bu
t-Bu
t-Bu
t-Bu
O
S
t-Bu
N
Me
P
M14
31P NMR, CDCl3, 162 MHz
25 20 15 10 5 0 -5 -10 -15 -20 -25 -30 -35 -40 -45 -50 -55 -60 -65 -70 -75 -80 -85
f1 (ppm)

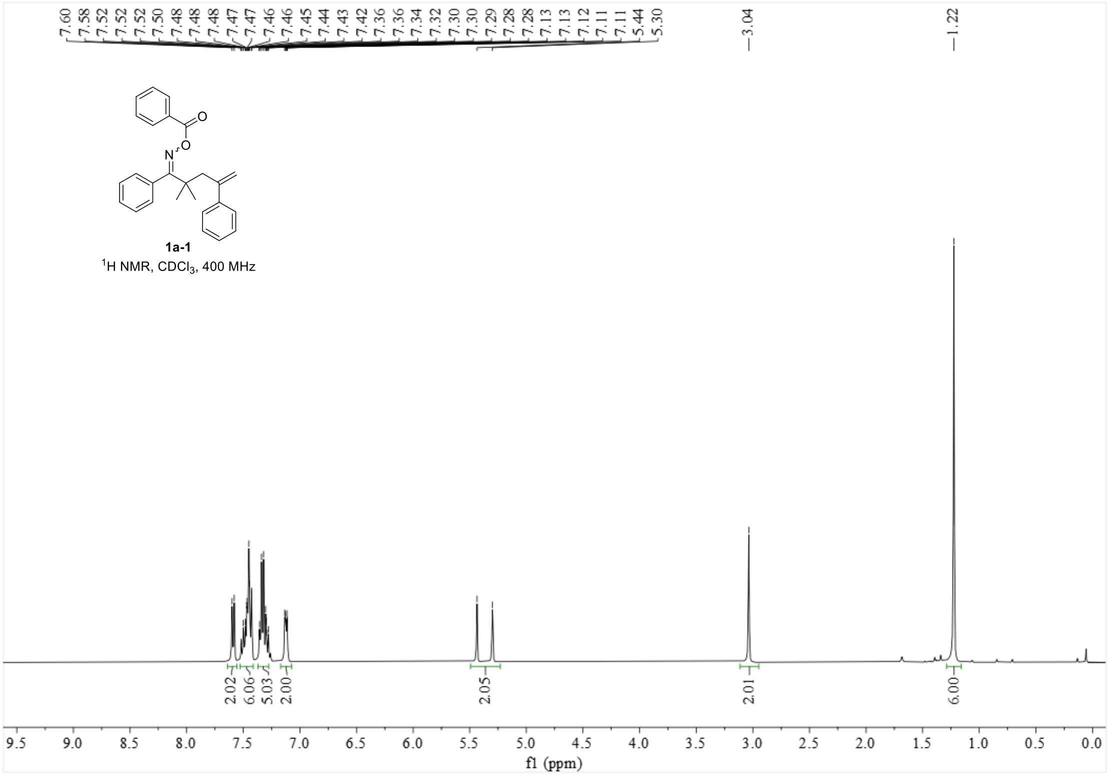
7.60 7.58 7.52 7.52 7.52 7.50 7.48 7.48 7.48 7.47 7.47 7.46 7.46 7.45 7.44 7.43 7.42 7.36 7.36 7.34 7.32 7.30 7.30 7.29 7.28 7.28 7.13 7.13 7.12 7.11 7.11 5.44 5.30
3.04
1.22
1a-1
1H NMR, CDCl3, 400 MHz
2.02 6.06 5.03 2.00
2.05
2.01
6.00
f1 (ppm)

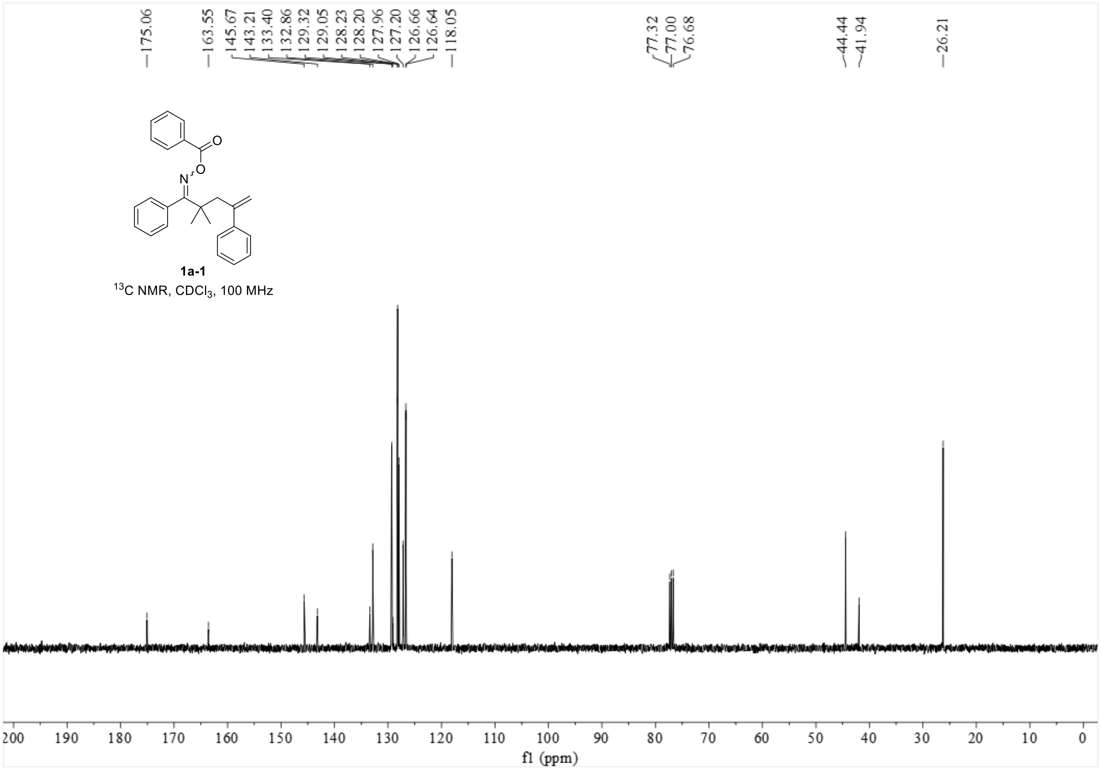
175.06
163.55
145.67 143.21 133.40 132.86 129.32 129.05 128.23 128.20 127.96 127.20 126.66 126.64 118.05
77.32 77.00 76.68
44.44 41.94
26.21
1a-1
13C NMR, CDCl3, 100 MHz
f1 (ppm)

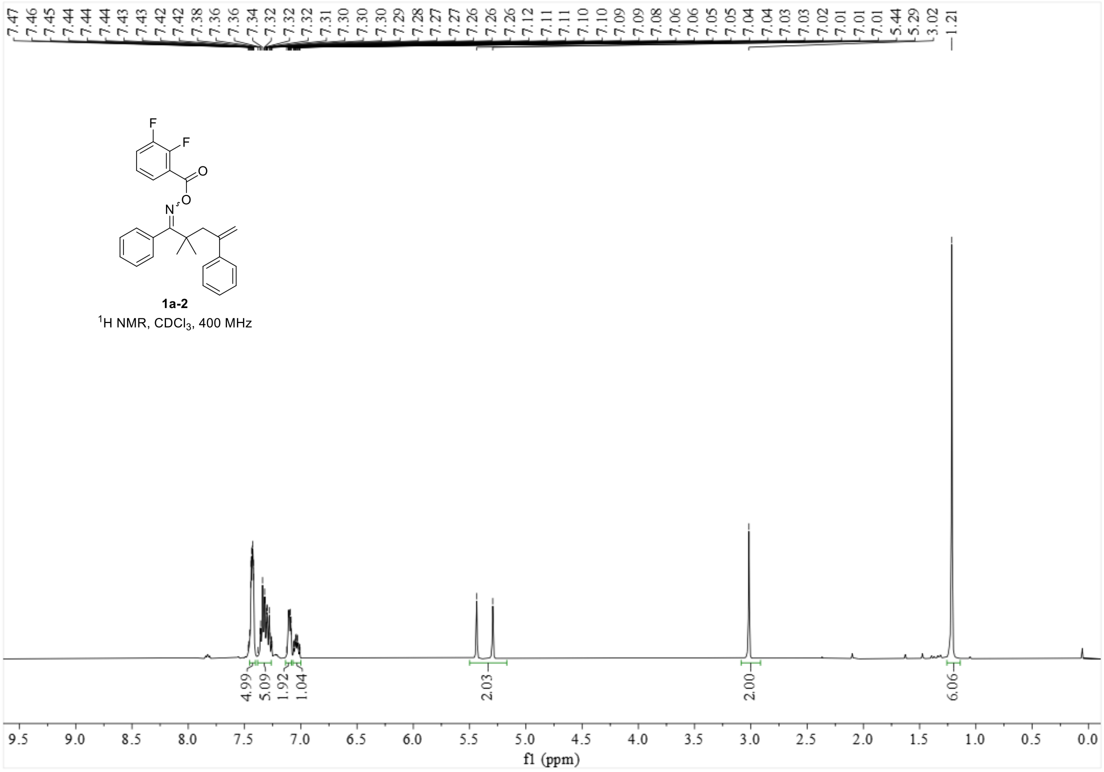

7.47 7.46 7.45 7.44 7.44 7.44 7.43 7.43 7.42 7.42 7.38 7.36 7.34 7.32 7.32 7.32 7.32 7.31 7.30 7.30 7.30 7.29 7.28 7.27 7.27 7.26 7.26 7.12 7.11 7.11 7.10 7.10 7.09 7.09 7.08 7.06 7.06 7.05 7.05 7.04 7.04 7.03 7.03 7.02 7.01 7.01 7.01 5.44 5.29 3.02 1.21
F
F
O
O
N
1a-2
^{1}H NMR, CDCl$_3$, 400 MHz
4.99 5.09 1.92 1.04 2.03 2.00 6.06
9.5 9.0 8.5 8.0 7.5 7.0 6.5 6.0 5.5 5.0 4.5 4.0 3.5 3.0 2.5 2.0 1.5 1.0 0.5 0.0
f1 (ppm)

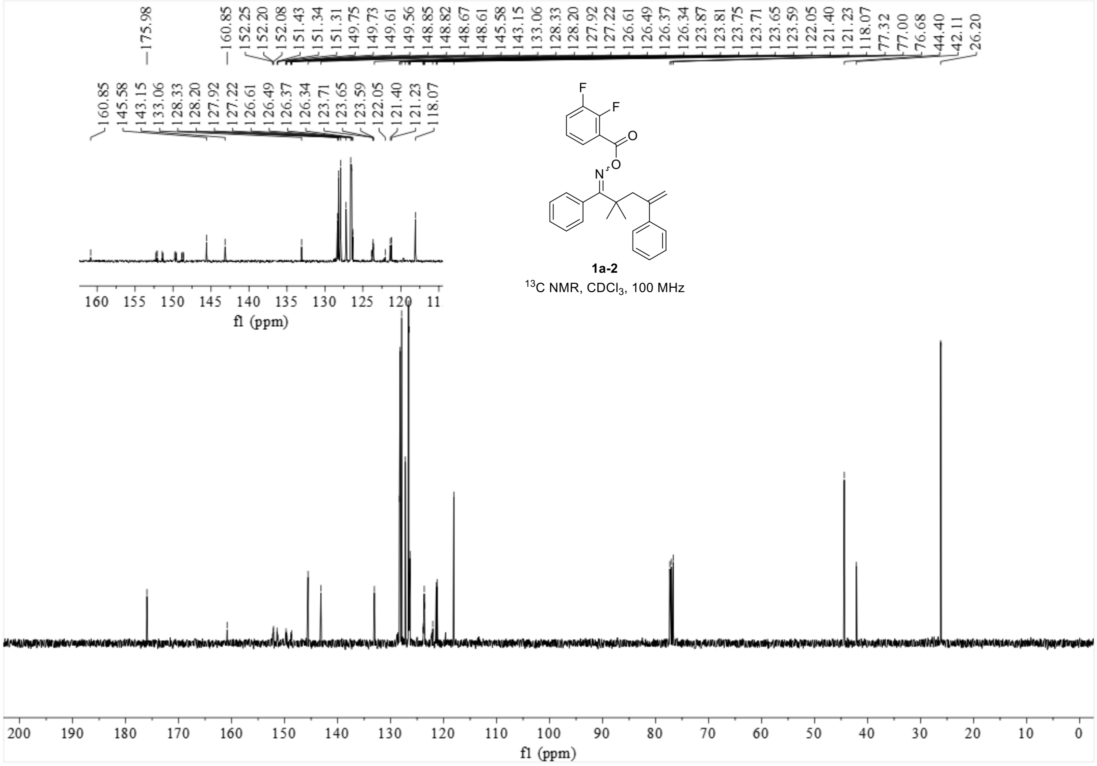

175.98 160.85 152.25 152.20 152.08 151.43 151.34 151.31 149.75 149.73 149.61 149.56 148.85 148.82 148.67 148.61 145.58 143.15 133.06 128.33 128.20 127.92 127.22 126.61 126.49 126.37 126.34 123.87 123.81 123.75 123.71 123.65 123.59 122.05 121.40 121.23 118.07 77.32 77.00 76.68 44.40 42.11 26.20
160.85 145.58 143.15 133.06 128.33 128.20 127.92 127.22 126.61 126.49 126.37 126.34 123.71 123.65 123.59 122.05 121.40 121.23 118.07
160 155 150 145 140 135 130 125 120 11
f1 (ppm)
F
F
O
O
N
1a-2
^{13}C NMR, CDCl$_3$, 100 MHz
200 190 180 170 160 150 140 130 120 110 100 90 80 70 60 50 40 30 20 10 0
f1 (ppm)

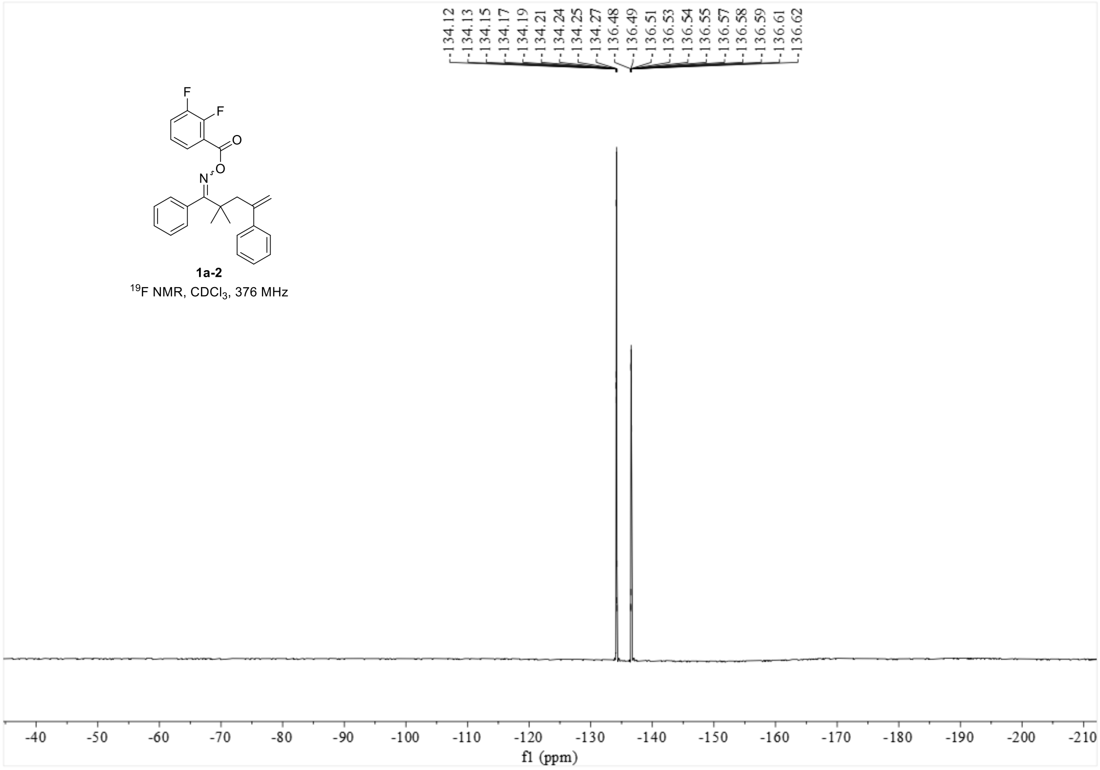
-134.12
-134.13
-134.15
-134.17
-134.19
-134.21
-134.24
-134.25
-134.27
-136.48
-136.49
-136.51
-136.53
-136.54
-136.55
-136.57
-136.58
-136.59
-136.61
-136.62
F
F
O
N
O
1a-2
19F NMR, CDCl3, 376 MHz
-40 -50 -60 -70 -80 -90 -100 -110 -120 -130 -140 -150 -160 -170 -180 -190 -200 -210
f1 (ppm)

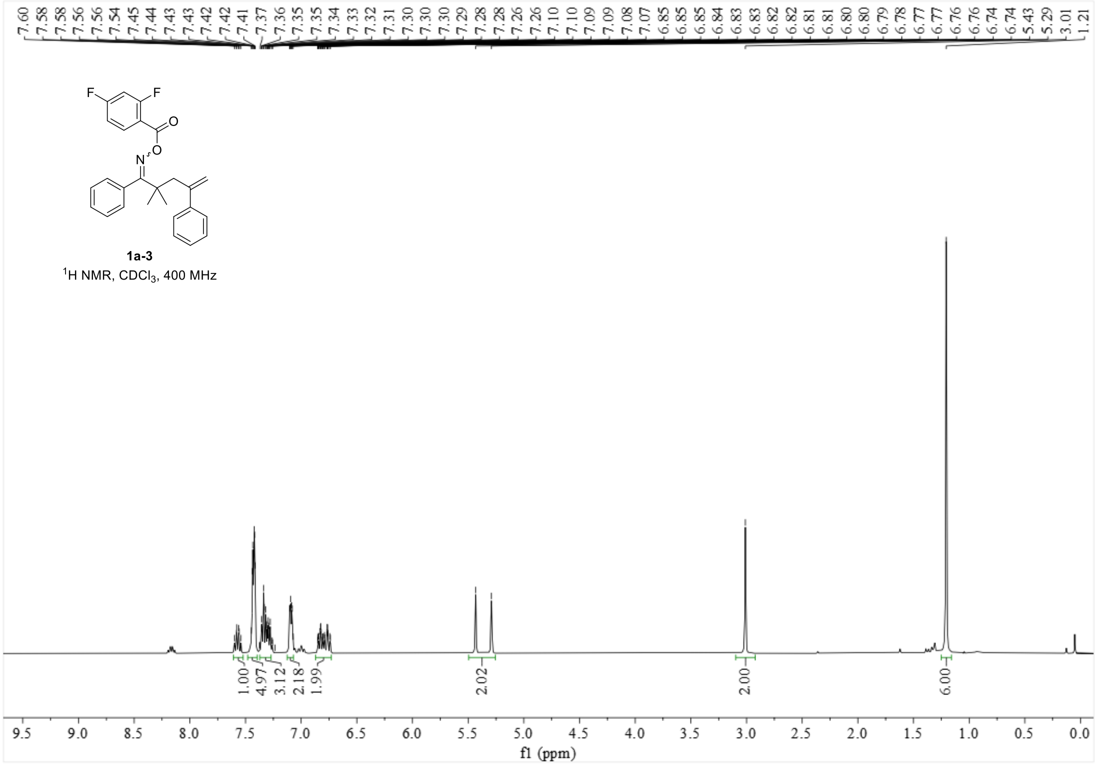
7.60
7.58
7.58
7.56
7.56
7.54
7.45
7.44
7.43
7.43
7.42
7.42
7.41
7.37
7.36
7.35
7.35
7.34
7.33
7.32
7.31
7.30
7.30
7.30
7.29
7.28
7.28
7.26
7.10
7.10
7.09
7.09
7.08
7.07
6.85
6.85
6.85
6.84
6.83
6.83
6.82
6.82
6.81
6.81
6.80
6.80
6.79
6.78
6.77
6.77
6.76
6.76
6.74
6.74
5.43
5.29
3.01
1.21
F
F
O
N
O
1a-3
1H NMR, CDCl3, 400 MHz
1.00
4.97
3.12
2.18
1.99
2.02
2.00
6.00
9.5 9.0 8.5 8.0 7.5 7.0 6.5 6.0 5.5 5.0 4.5 4.0 3.5 3.0 2.5 2.0 1.5 1.0 0.5 0.0
f1 (ppm)

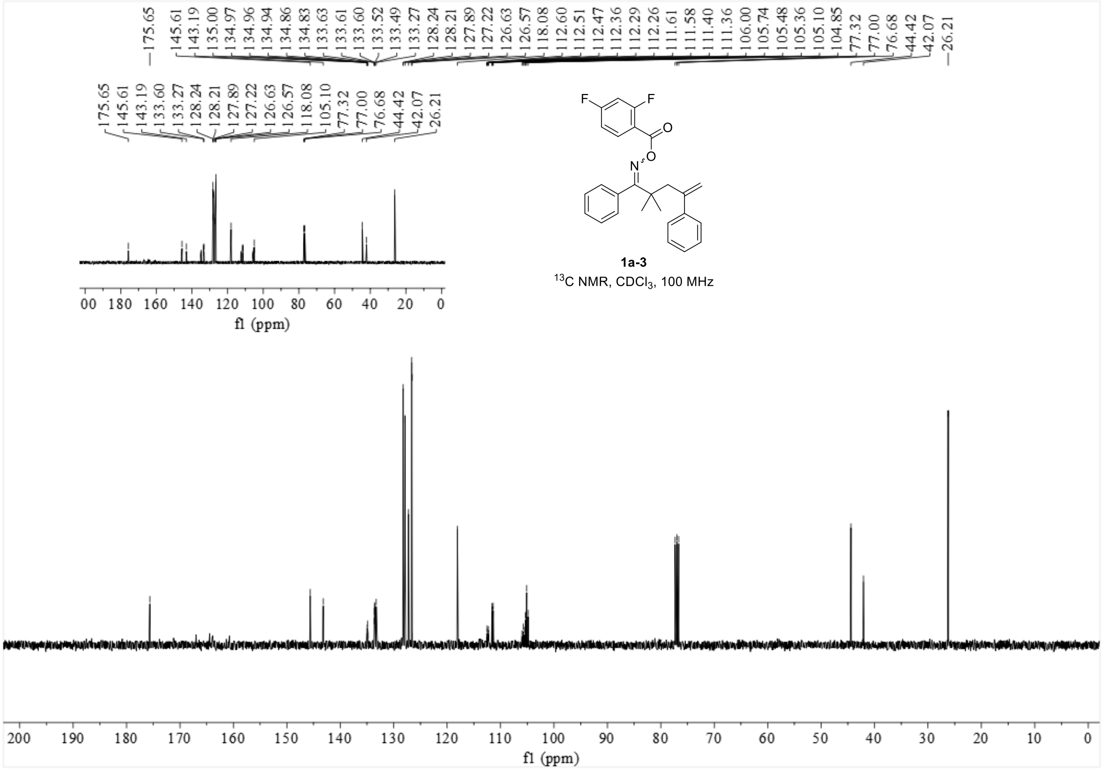
175.65
145.61
143.19
135.00
134.97
134.96
134.94
134.86
134.83
133.63
133.61
133.60
133.52
133.49
133.27
128.24
128.21
127.89
127.22
126.63
126.57
118.08
112.60
112.51
112.47
112.36
112.29
112.26
111.61
111.58
111.40
111.36
106.00
105.74
105.48
105.36
105.10
104.85
77.32
77.00
76.68
44.42
42.07
26.21
175.65
145.61
143.19
133.60
133.27
128.24
128.21
127.89
127.22
126.63
126.57
118.08
105.10
77.32
77.00
76.68
44.42
42.07
26.21
F
F
O
N
O
1a-3
13C NMR, CDCl3, 100 MHz
fl (ppm)
fl (ppm)

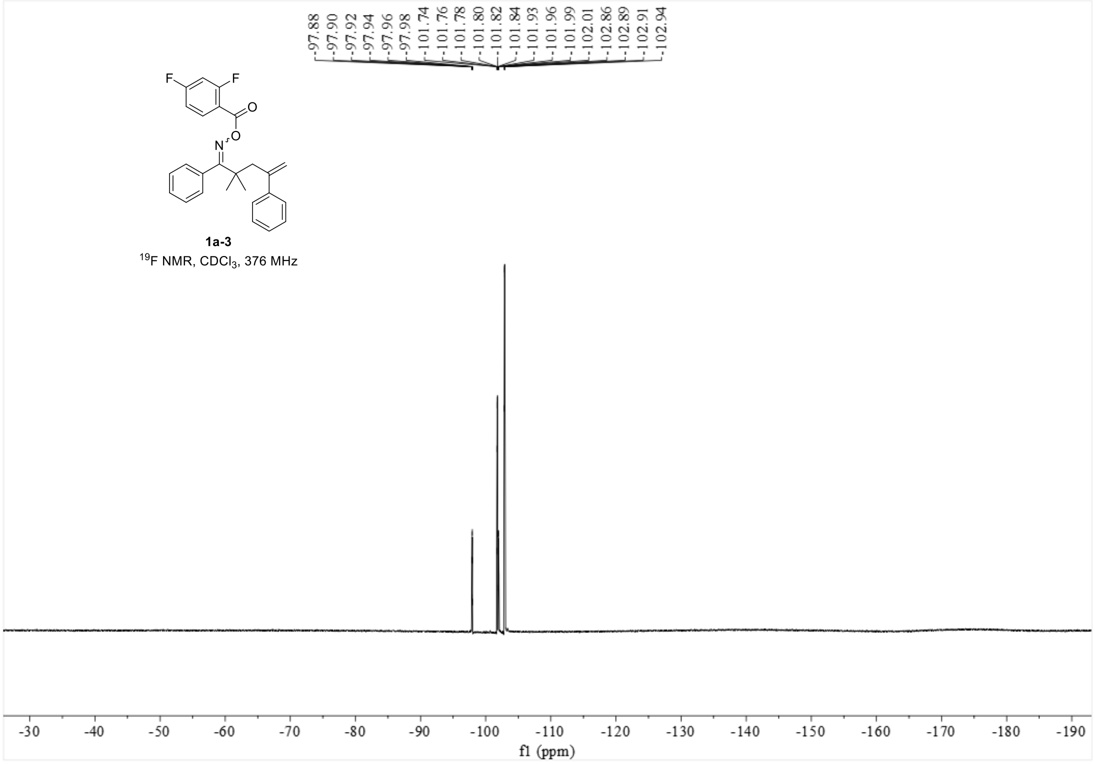
-97.88
-97.90
-97.92
-97.94
-97.96
-97.98
-101.74
-101.76
-101.78
-101.80
-101.82
-101.84
-101.93
-101.96
-101.99
-102.01
-102.86
-102.89
-102.91
-102.94
F
F
O
N
O
1a-3
19F NMR, CDCl3, 376 MHz
fl (ppm)

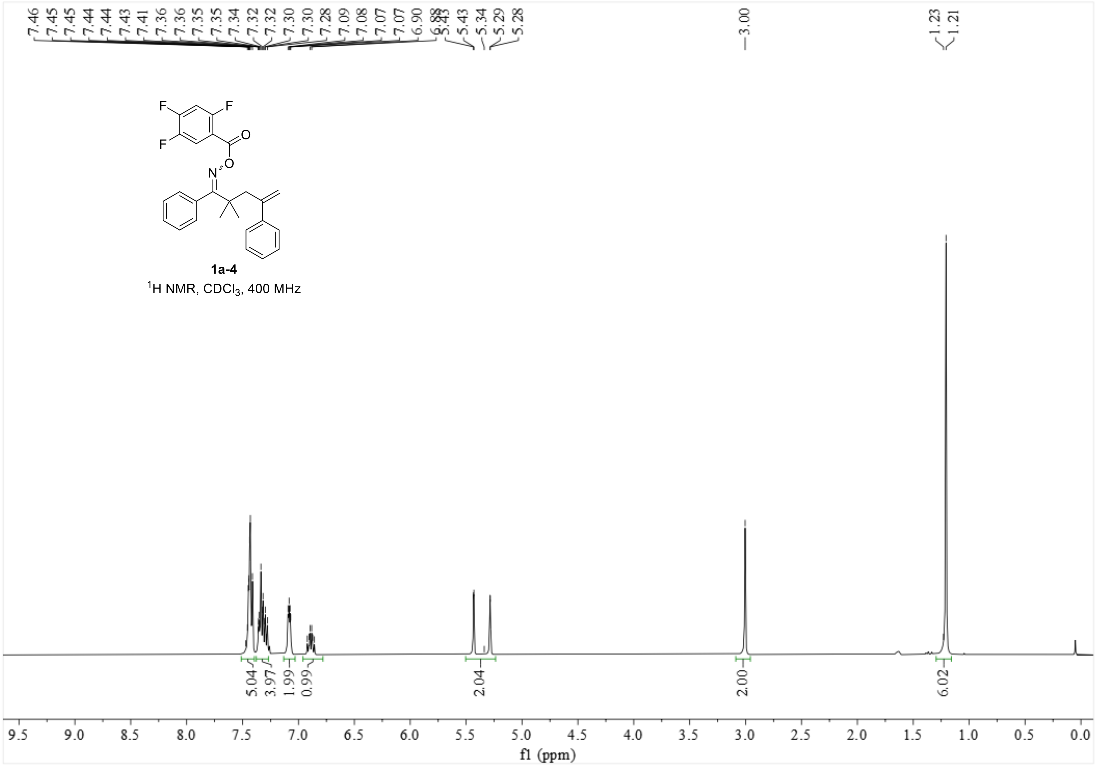
7.46
7.45
7.45
7.44
7.44
7.43
7.41
7.36
7.36
7.35
7.35
7.34
7.32
7.32
7.30
7.30
7.28
7.09
7.08
7.07
7.07
6.90
6.88
5.43
5.43
5.34
5.29
5.28
3.00
1.23
1.21
1a-4
1H NMR, CDCl3, 400 MHz
5.04
3.97
1.99
0.99
2.04
2.00
6.02
9.5 9.0 8.5 8.0 7.5 7.0 6.5 6.0 5.5 5.0 4.5 4.0 3.5 3.0 2.5 2.0 1.5 1.0 0.5 0.0
f1 (ppm)

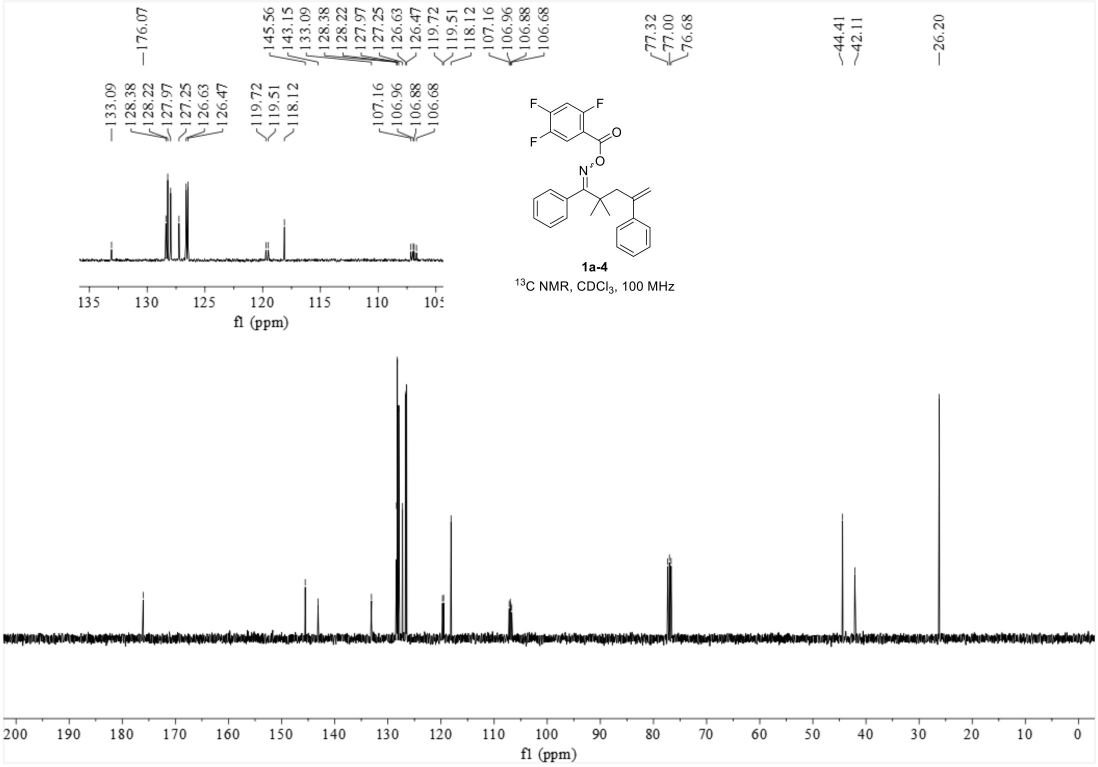
176.07
145.56
143.15
133.09
128.38
128.22
127.97
127.25
126.63
126.47
119.72
119.51
118.12
107.16
106.96
106.88
106.68
77.32
77.00
76.68
44.41
42.11
26.20
1a-4
13C NMR, CDCl3, 100 MHz
135 130 125 120 115 110 105
f1 (ppm)
200 190 180 170 160 150 140 130 120 110 100 90 80 70 60 50 40 30 20 10 0
f1 (ppm)

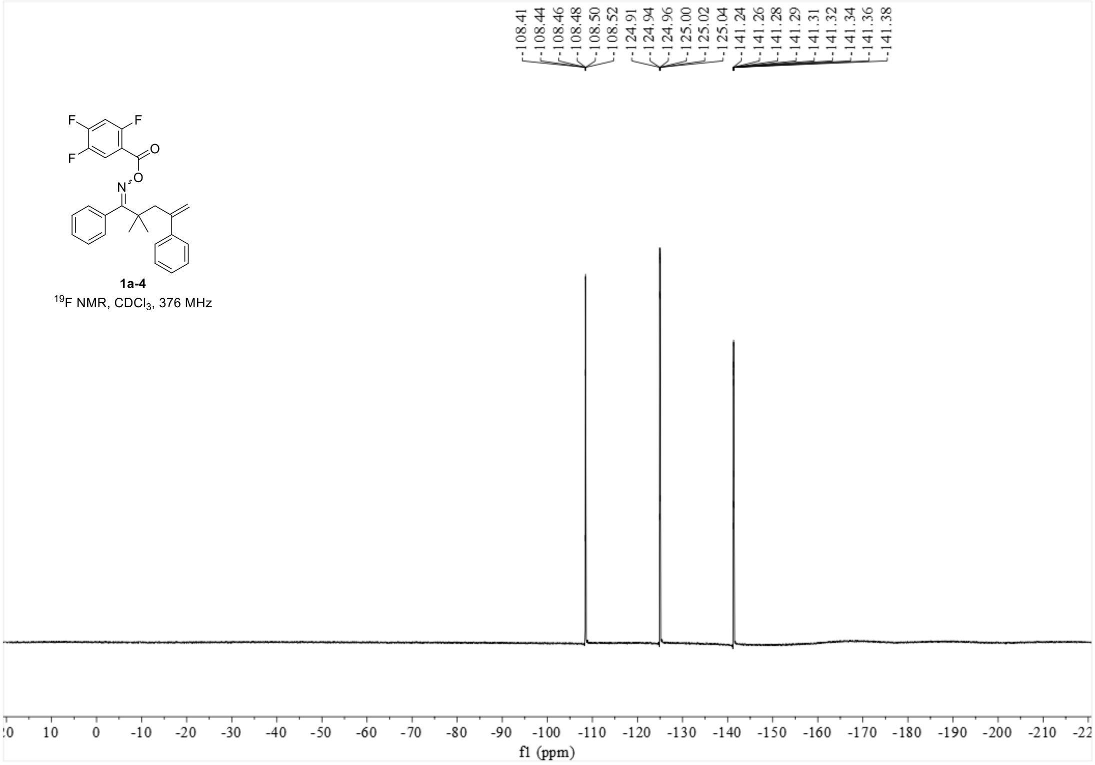
1a-4
^{19}F NMR, $CDCl_3$, 376 MHz
-108.41 -108.44 -108.46 -108.48 -108.50 -108.52 -124.91 -124.94 -124.96 -125.00 -125.02 -125.04 -141.24 -141.26 -141.28 -141.29 -141.31 -141.32 -141.34 -141.36 -141.38
f1 (ppm)

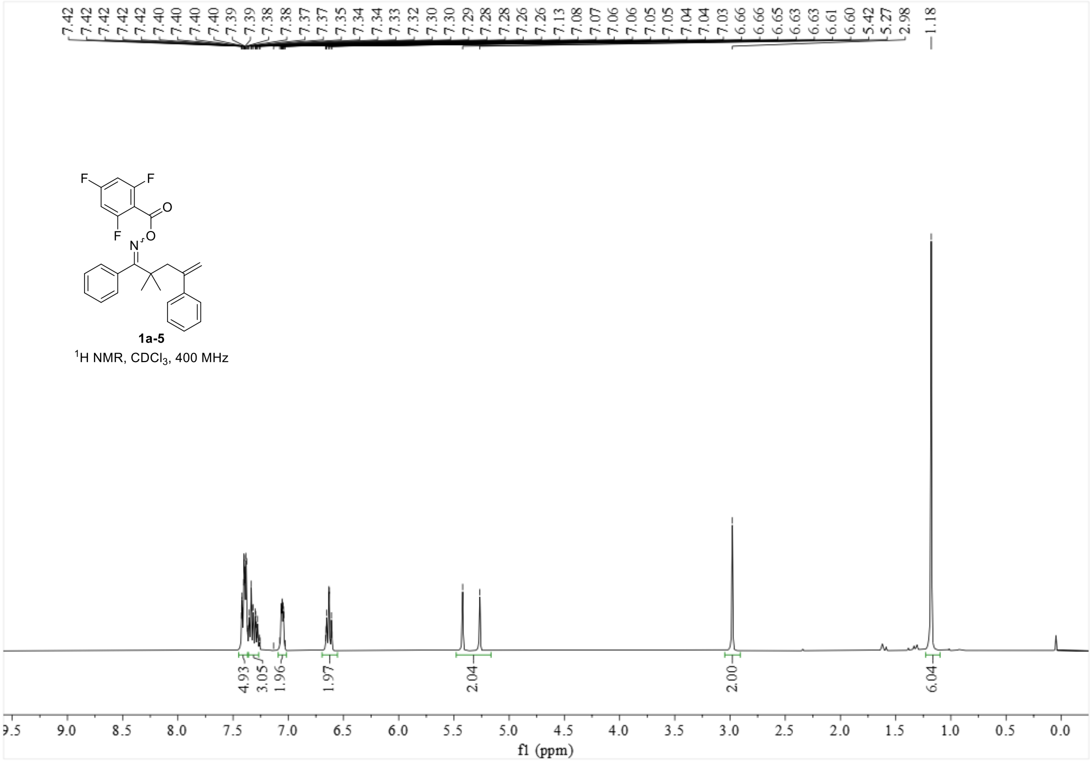
1a-5
^{1}H NMR, $CDCl_3$, 400 MHz
7.42 7.42 7.42 7.42 7.42 7.40 7.40 7.40 7.40 7.39 7.39 7.38 7.38 7.37 7.37 7.35 7.34 7.34 7.33 7.32 7.30 7.30 7.29 7.28 7.28 7.26 7.13 7.08 7.07 7.06 7.06 7.05 7.05 7.04 7.04 7.03 6.66 6.66 6.65 6.63 6.63 6.61 6.60 5.42 5.27 2.98 1.18
4.93 3.05 1.96 1.97 2.04 2.00 6.04
f1 (ppm)

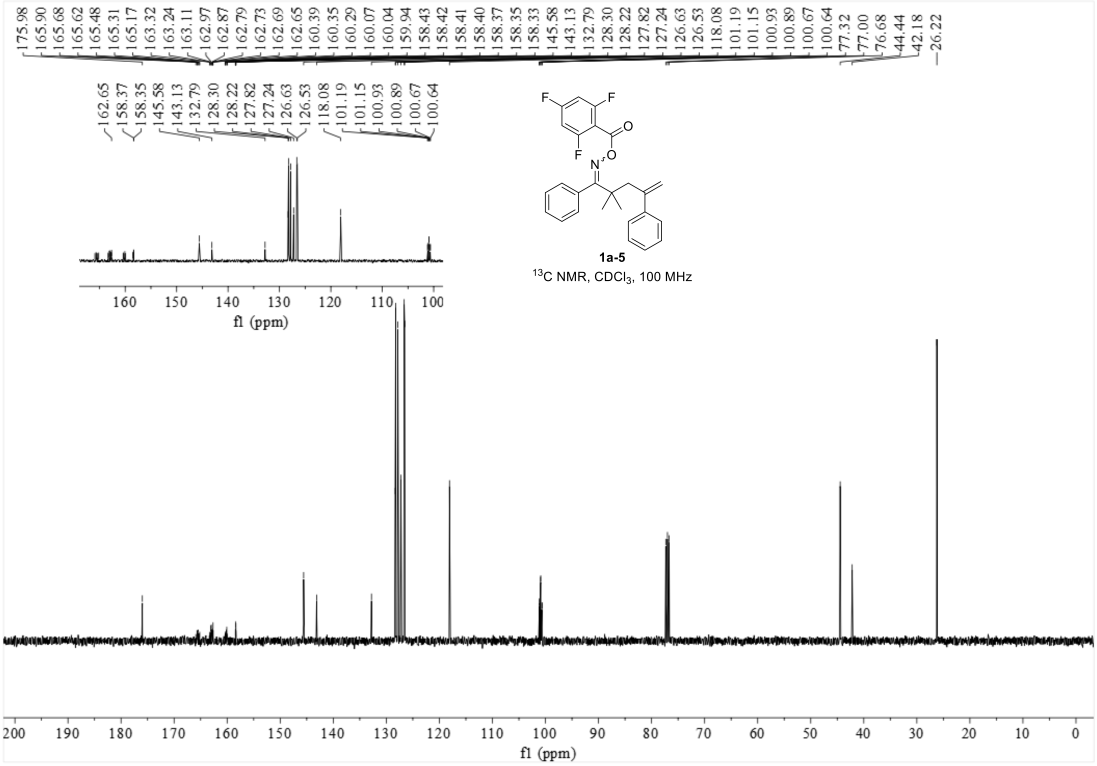
175.98
165.90
165.68
165.62
165.48
165.31
165.17
163.32
163.24
163.11
162.97
162.87
162.79
162.73
162.69
162.65
160.39
160.35
160.29
160.07
160.04
159.94
158.43
158.42
158.41
158.40
158.37
158.35
158.33
145.58
143.13
132.79
128.30
128.22
127.82
127.24
126.63
126.53
118.08
101.19
101.15
100.93
100.89
100.67
100.64
77.32
77.00
76.68
44.44
42.18
26.22
1a-5
^{13}C NMR, CDCl$_3$, 100 MHz
fl (ppm)

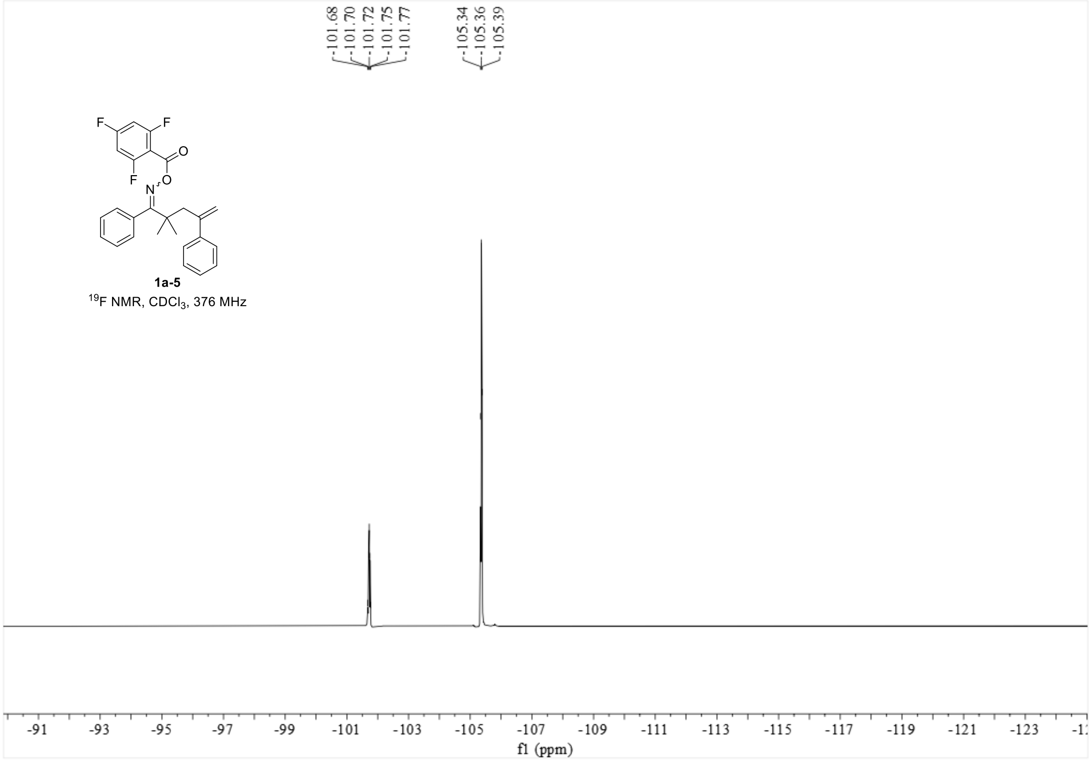
-101.68
-101.70
-101.72
-101.75
-101.77
-105.34
-105.36
-105.39
1a-5
^{19}F NMR, CDCl$_3$, 376 MHz
fl (ppm)

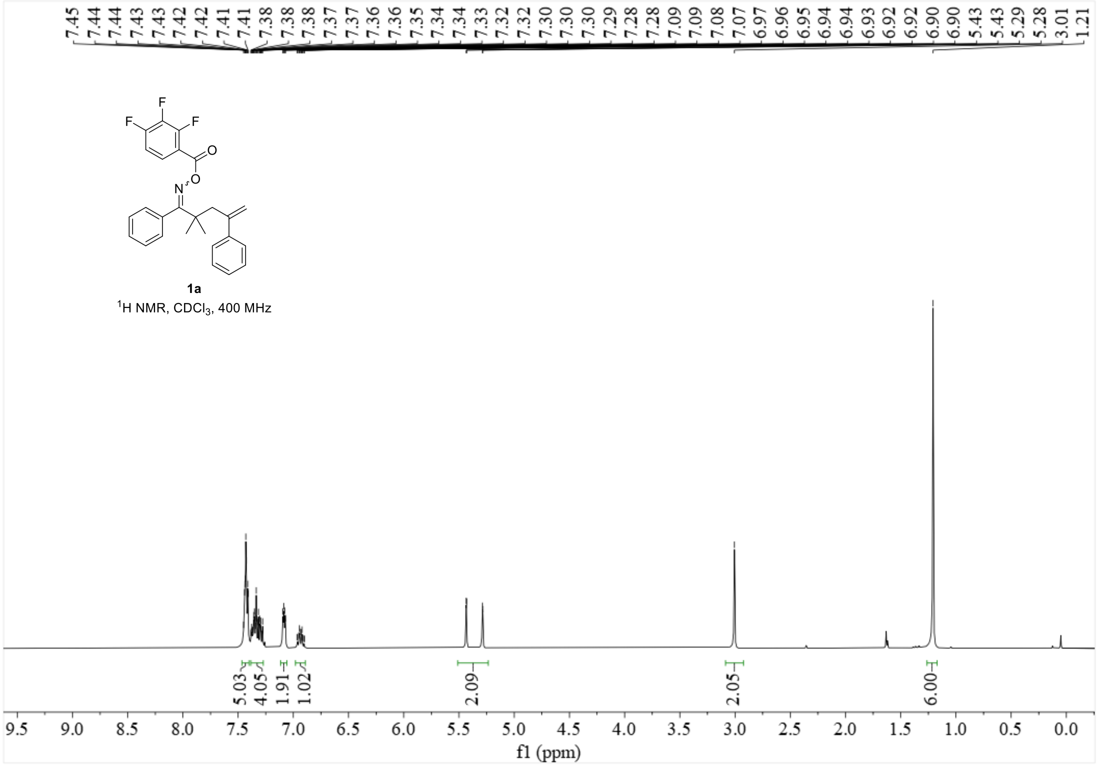
7.45 7.44 7.44 7.43 7.43 7.42 7.42 7.41 7.41 7.38 7.38 7.38 7.37 7.37 7.36 7.36 7.35 7.34 7.34 7.33 7.32 7.32 7.30 7.30 7.30 7.29 7.28 7.28 7.09 7.09 7.08 7.07 6.97 6.96 6.95 6.94 6.94 6.93 6.92 6.92 6.90 6.90 5.43 5.43 5.29 5.28 3.01 1.21
F
F
F
O
O
N
1a
1H NMR, CDCl3, 400 MHz
5.03 4.05 1.91 1.02 2.09 2.05 6.00
9.5 9.0 8.5 8.0 7.5 7.0 6.5 6.0 5.5 5.0 4.5 4.0 3.5 3.0 2.5 2.0 1.5 1.0 0.5 0.0
f1 (ppm)

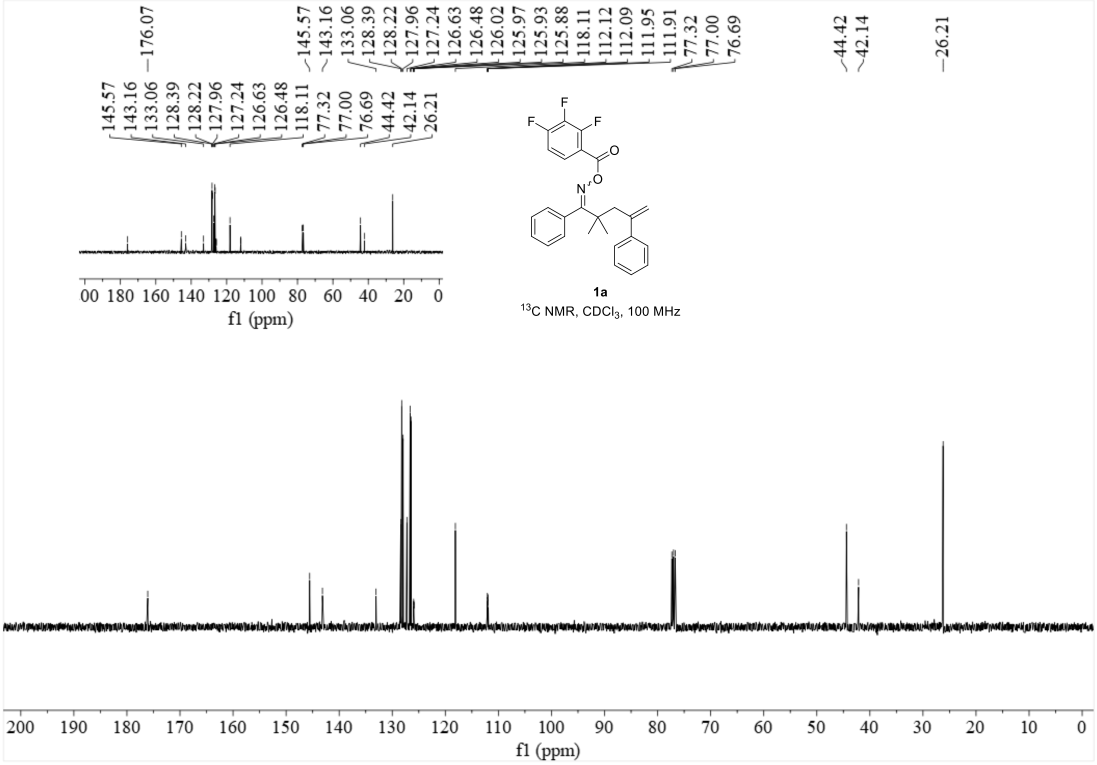
176.07 145.57 143.16 133.06 128.39 128.22 127.96 127.24 126.63 126.48 126.02 125.97 125.93 125.88 118.11 112.12 112.09 111.95 111.91 77.32 77.00 76.69 44.42 42.14 26.21
145.57 143.16 133.06 128.39 128.22 127.96 127.24 126.63 126.48 118.11 77.32 77.00 76.69 44.42 42.14 26.21
00 180 160 140 120 100 80 60 40 20 0
f1 (ppm)
F
F
F
O
O
N
1a
13C NMR, CDCl3, 100 MHz
200 190 180 170 160 150 140 130 120 110 100 90 80 70 60 50 40 30 20 10 0
f1 (ppm)

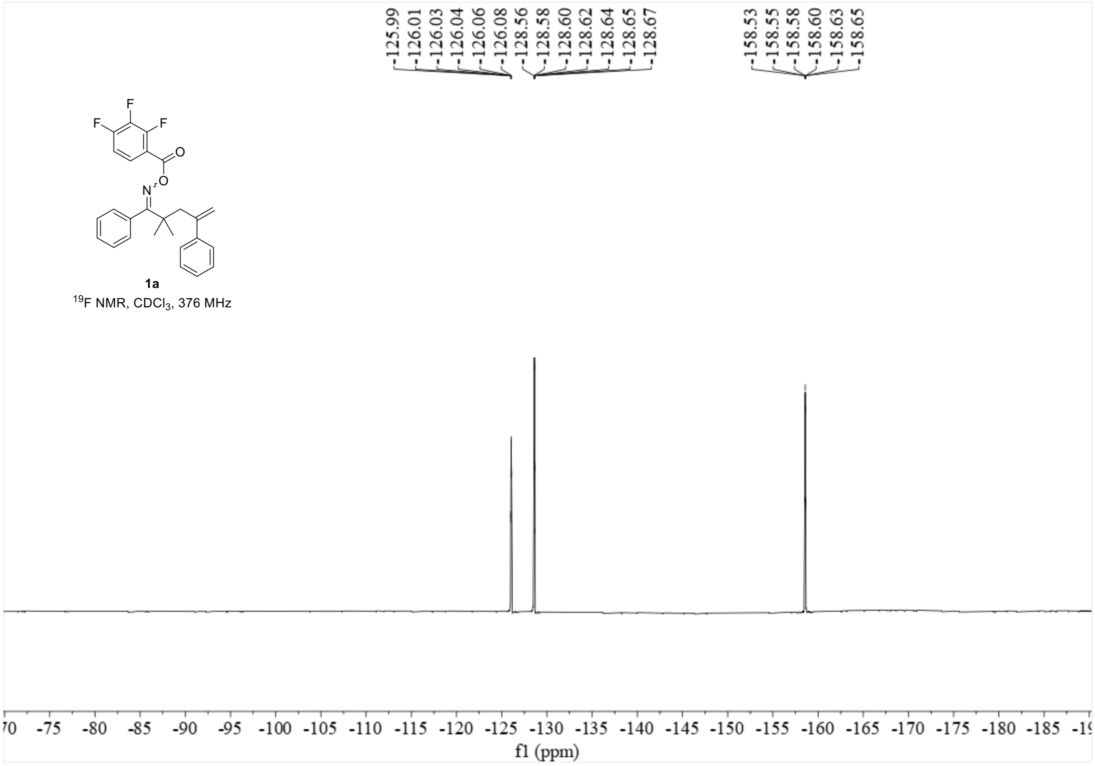
-125.99
-126.01
-126.03
-126.04
-126.06
-126.08
-128.56
-128.58
-128.60
-128.62
-128.64
-128.65
-128.67
-158.53
-158.55
-158.58
-158.60
-158.63
-158.65
1a
19F NMR, CDCl3, 376 MHz
f1 (ppm)

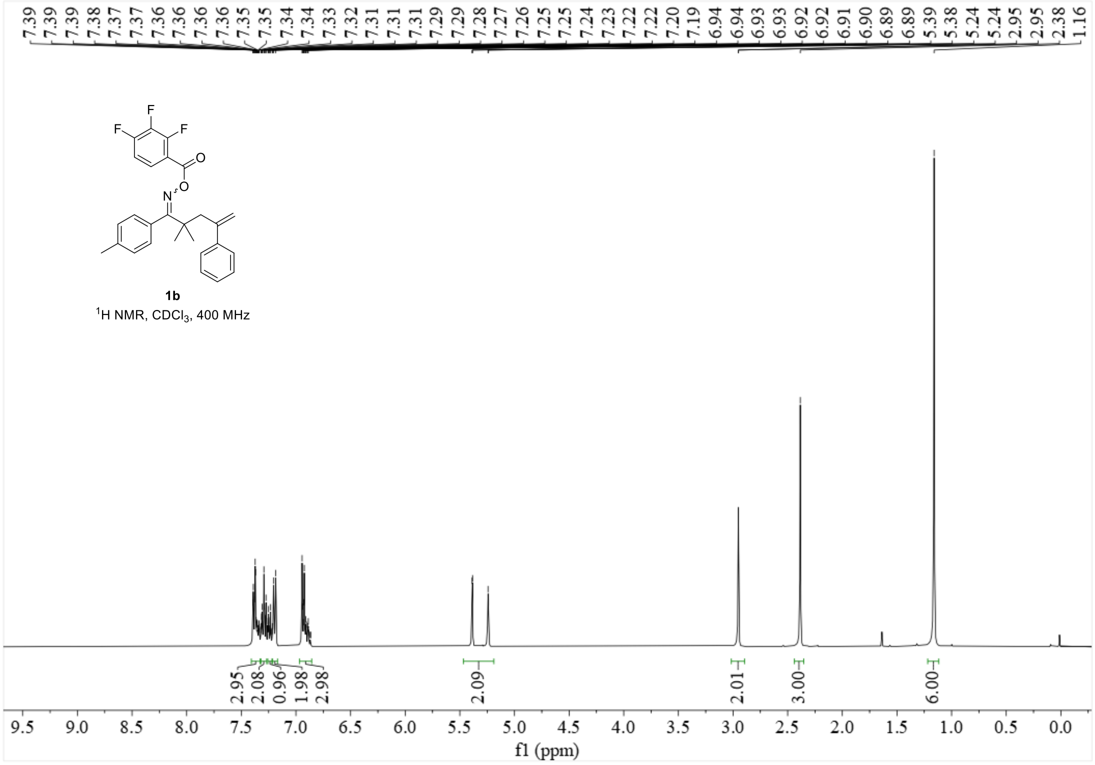
7.39
7.39
7.39
7.38
7.37
7.37
7.36
7.36
7.36
7.36
7.35
7.35
7.34
7.34
7.33
7.32
7.31
7.31
7.31
7.29
7.29
7.28
7.27
7.26
7.25
7.25
7.24
7.23
7.22
7.22
7.20
7.19
6.94
6.94
6.93
6.93
6.92
6.92
6.91
6.90
6.89
6.89
5.39
5.38
5.24
5.24
2.95
2.95
2.38
1.16
1b
1H NMR, CDCl3, 400 MHz
2.95
2.08
0.96
1.98
2.98
2.09
2.01
3.00
6.00
f1 (ppm)

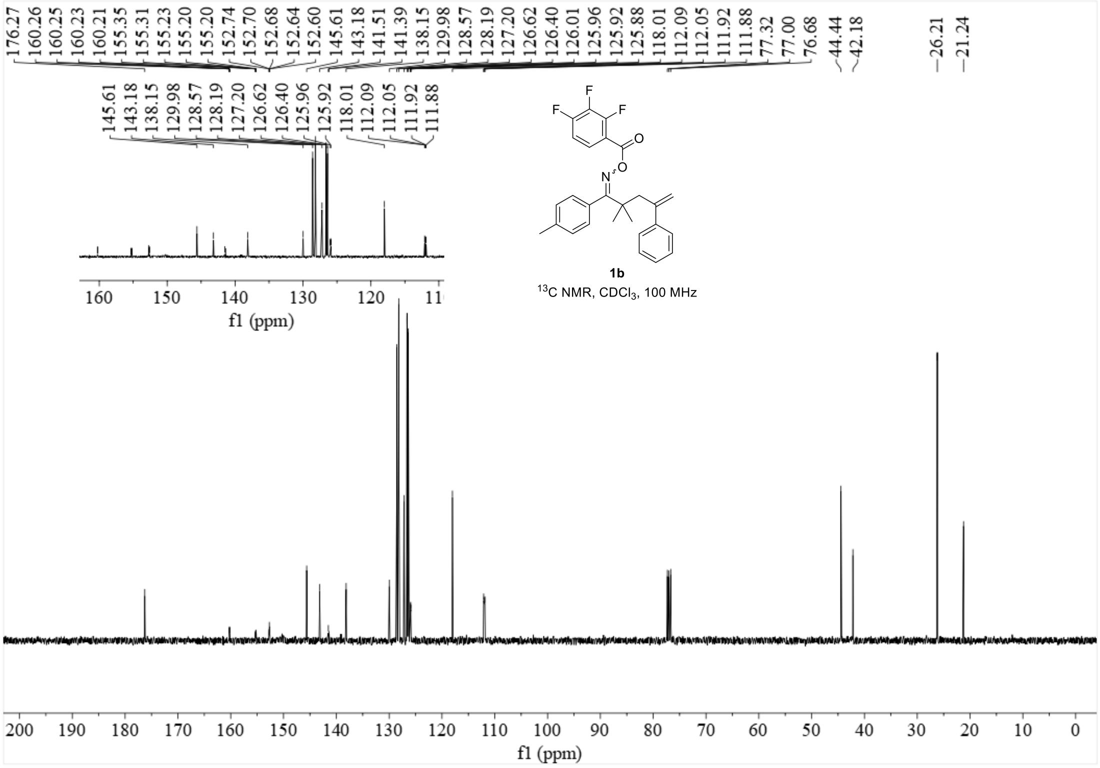
176.27
160.26
160.25
160.23
160.21
155.35
155.31
155.23
155.20
155.20
152.74
152.70
152.68
152.64
152.60
145.61
143.18
141.51
141.39
138.15
129.98
128.57
128.19
127.20
126.62
126.40
126.01
125.96
125.92
125.88
118.01
112.09
112.05
111.92
111.88
77.32
77.00
76.68
44.44
42.18
26.21
21.24
145.61
143.18
138.15
129.98
128.57
128.19
127.20
126.62
126.40
125.96
125.92
118.01
112.09
112.05
111.92
111.88
f1 (ppm)
F
F
F
O
O
N
1b
^{13}C NMR, $CDCl_3$, 100 MHz
f1 (ppm)

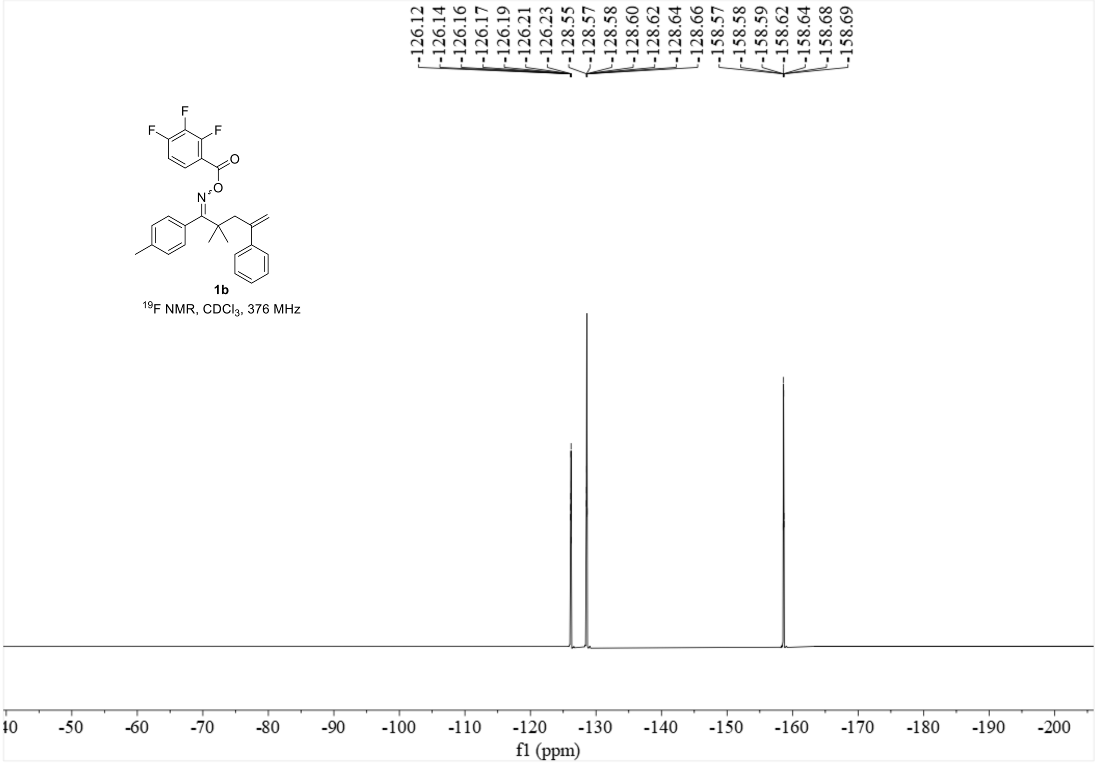
-126.12
-126.14
-126.16
-126.17
-126.19
-126.21
-126.23
-128.55
-128.57
-128.58
-128.60
-128.62
-128.64
-128.66
-158.57
-158.58
-158.59
-158.62
-158.64
-158.68
-158.69
F
F
F
O
O
N
1b
^{19}F NMR, $CDCl_3$, 376 MHz
f1 (ppm)

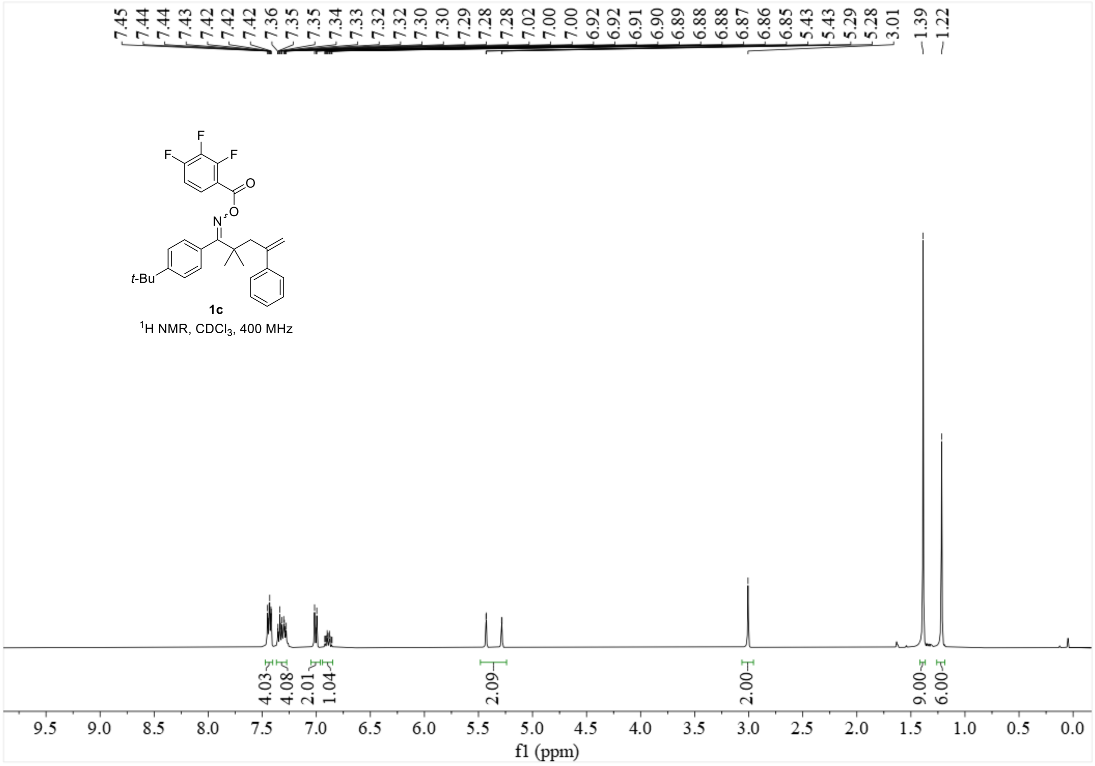

1c
^{1}H NMR, CDCl$_3$, 400 MHz
7.45
7.44
7.44
7.43
7.42
7.42
7.42
7.36
7.35
7.35
7.34
7.33
7.32
7.32
7.30
7.30
7.29
7.28
7.28
7.02
7.00
7.00
6.92
6.92
6.91
6.90
6.89
6.88
6.88
6.87
6.86
6.85
5.43
5.43
5.29
5.28
3.01
1.39
1.22
4.03
4.08
2.01
1.04
2.09
2.00
9.00
6.00
f1 (ppm)

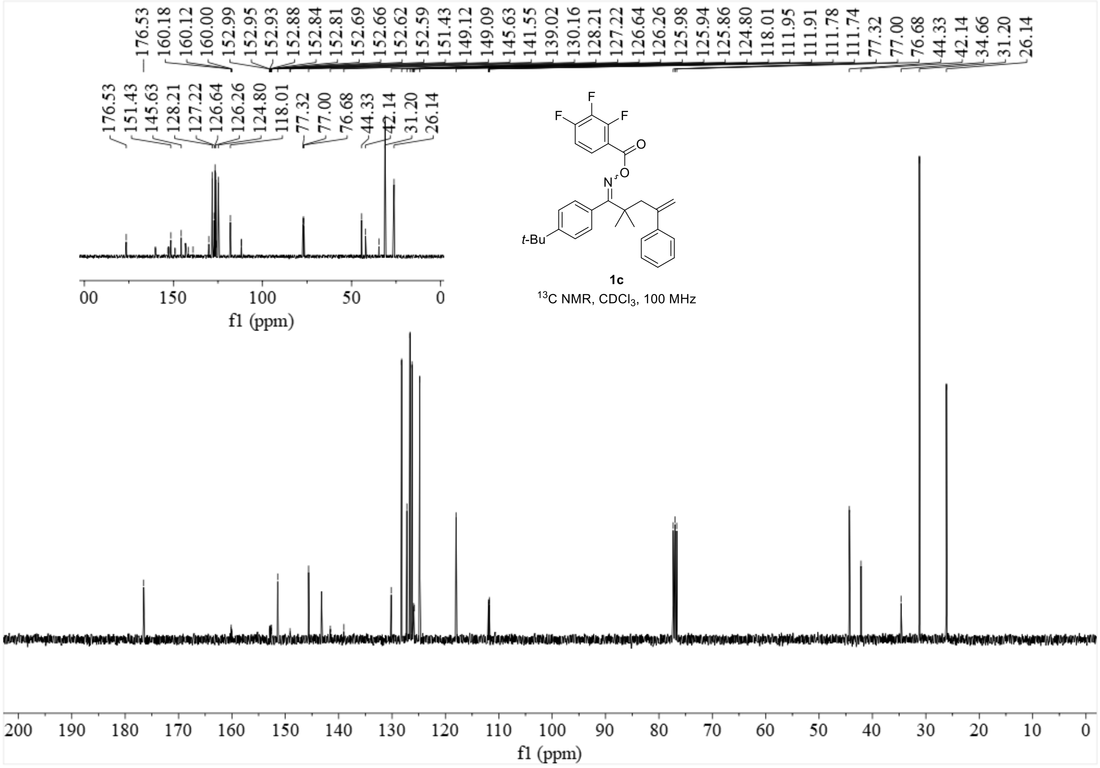

1c
^{13}C NMR, CDCl$_3$, 100 MHz
176.53
160.18
160.12
160.00
152.99
152.95
152.93
152.88
152.84
152.81
152.69
152.66
152.62
152.59
151.43
149.12
149.09
145.63
141.55
139.02
130.16
128.21
127.22
126.64
126.26
125.98
125.94
125.86
124.80
118.01
111.95
111.91
111.78
111.74
77.32
77.00
76.68
44.33
42.14
34.66
31.20
26.14
f1 (ppm)

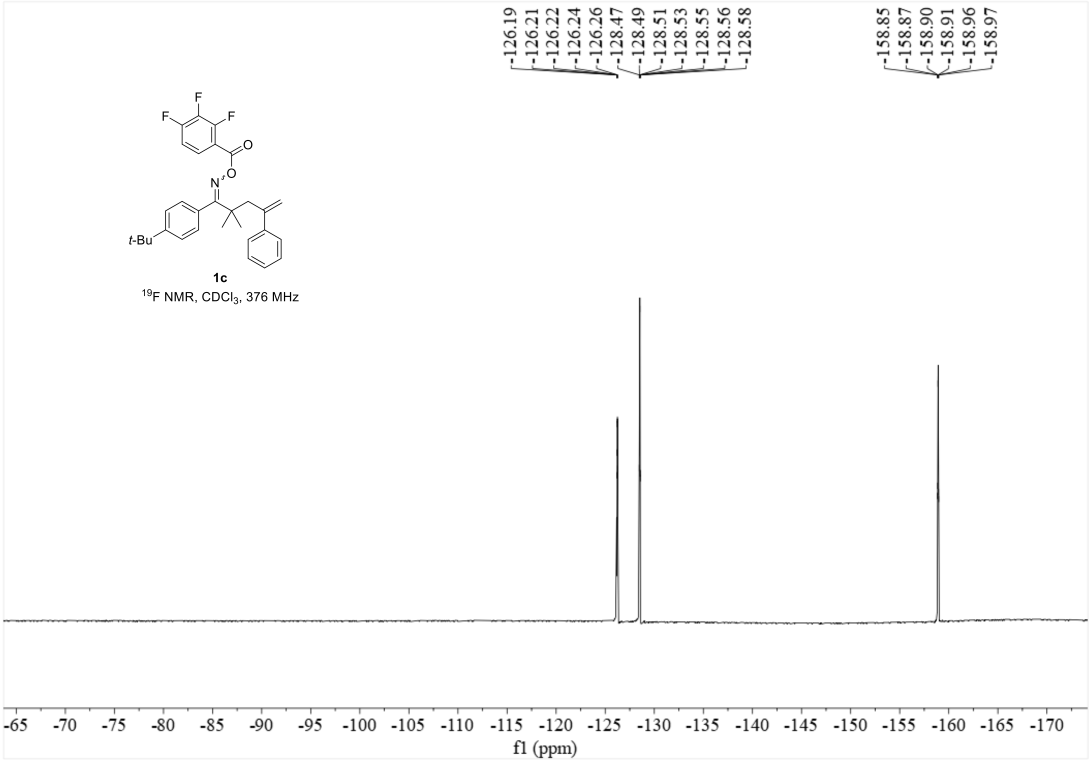
-126.19
-126.21
-126.22
-126.24
-126.26
-128.47
-128.49
-128.51
-128.53
-128.55
-128.56
-128.58
-158.85
-158.87
-158.90
-158.91
-158.96
-158.97
F
F
F
O
N
O
t-Bu
1c
19F NMR, CDCl3, 376 MHz
-65 -70 -75 -80 -85 -90 -95 -100 -105 -110 -115 -120 -125 -130 -135 -140 -145 -150 -155 -160 -165 -170
f1 (ppm)

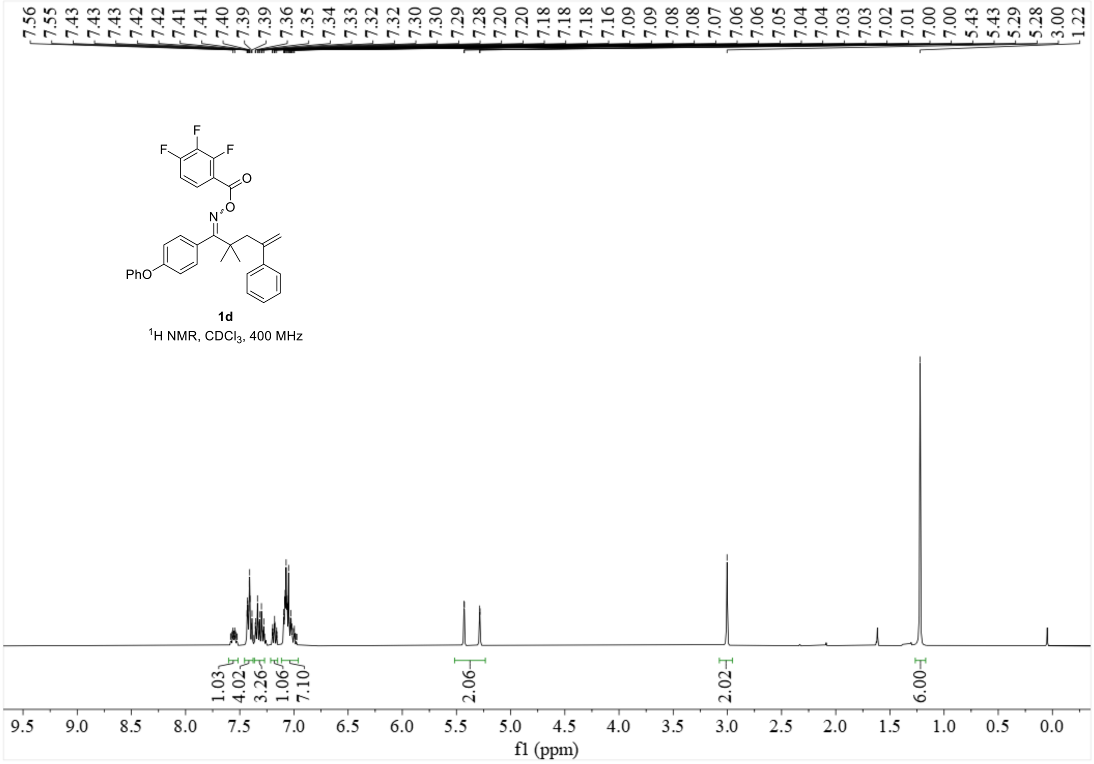
7.56
7.55
7.43
7.43
7.43
7.42
7.42
7.41
7.41
7.40
7.39
7.39
7.36
7.35
7.34
7.33
7.32
7.32
7.30
7.29
7.28
7.20
7.20
7.18
7.18
7.16
7.09
7.08
7.08
7.07
7.06
7.05
7.04
7.04
7.03
7.02
7.01
7.00
7.00
5.43
5.43
5.29
5.28
3.00
1.22
F
F
F
O
N
O
PhO
1d
1H NMR, CDCl3, 400 MHz
1.03
4.02
3.26
1.06
7.10
2.06
2.02
6.00
9.5 9.0 8.5 8.0 7.5 7.0 6.5 6.0 5.5 5.0 4.5 4.0 3.5 3.0 2.5 2.0 1.5 1.0 0.5 0.0
f1 (ppm)

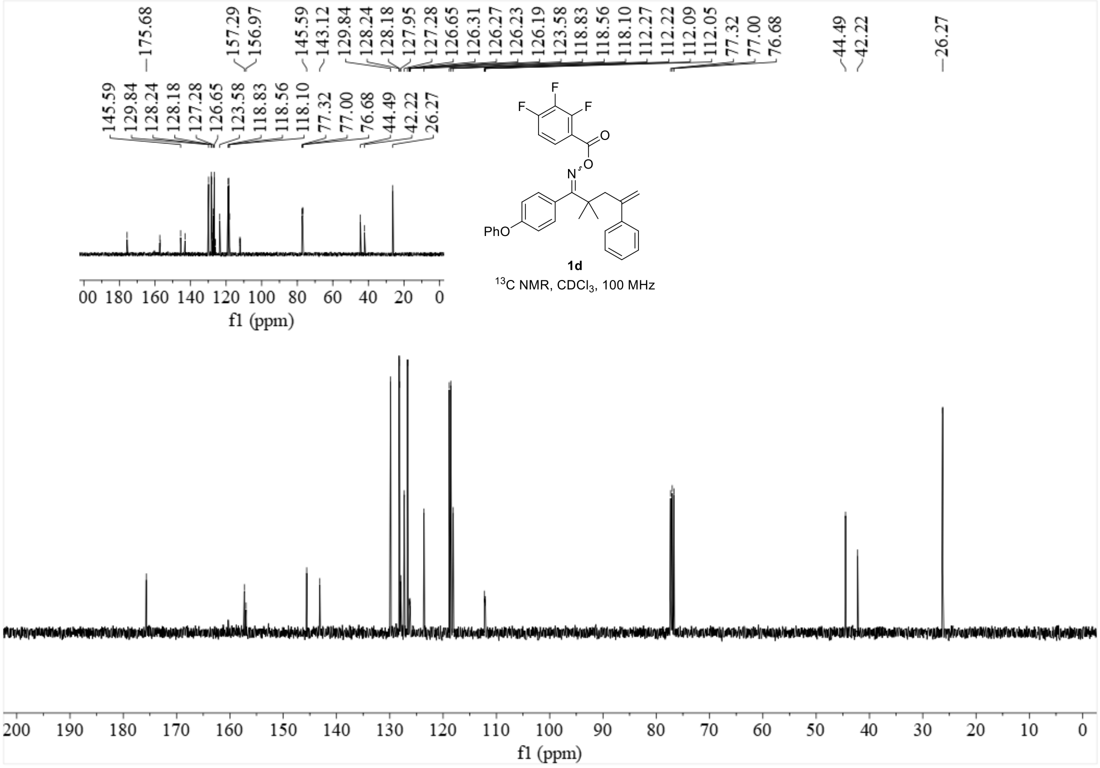
175.68
157.29
156.97
145.59
143.12
129.84
128.24
128.18
127.95
127.28
126.65
126.31
126.27
126.23
126.19
123.58
118.83
118.56
118.10
112.27
112.22
112.09
112.05
77.32
77.00
76.68
44.49
42.22
26.27
1d
^{13}C NMR, $CDCl_3$, 100 MHz
f1 (ppm)

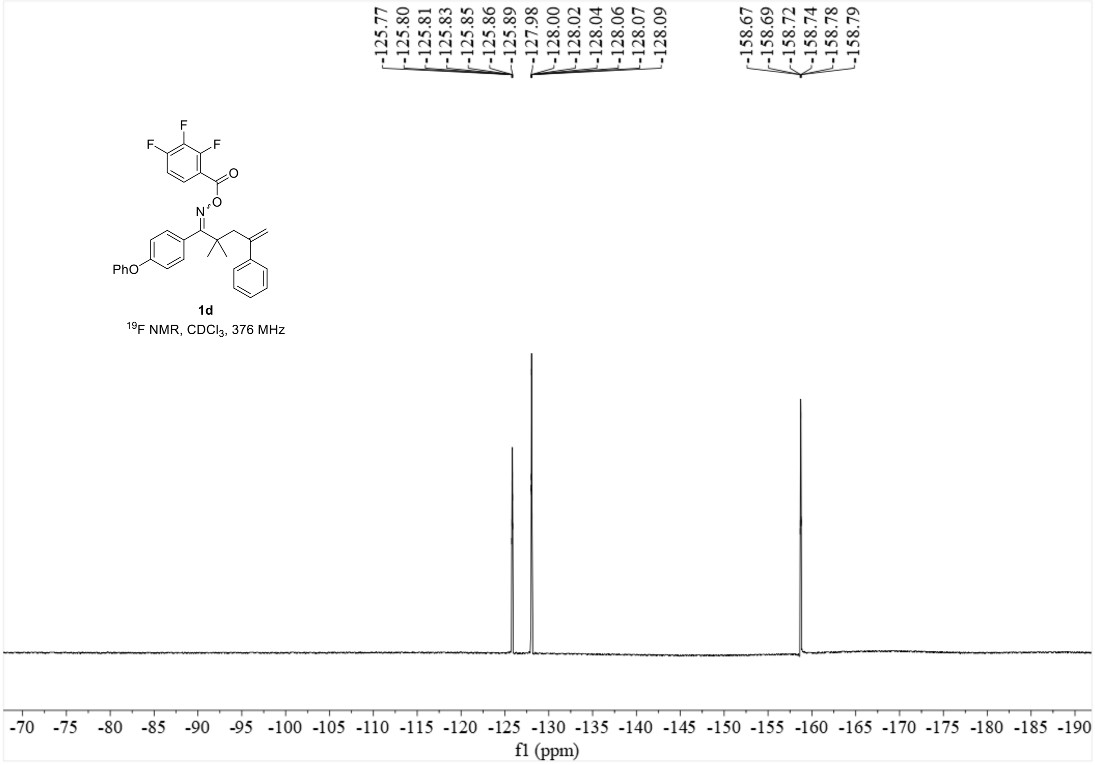
-125.77
-125.80
-125.81
-125.83
-125.85
-125.86
-125.89
-127.98
-128.00
-128.02
-128.04
-128.06
-128.07
-128.09
-158.67
-158.69
-158.72
-158.74
-158.78
-158.79
1d
^{19}F NMR, $CDCl_3$, 376 MHz
f1 (ppm)

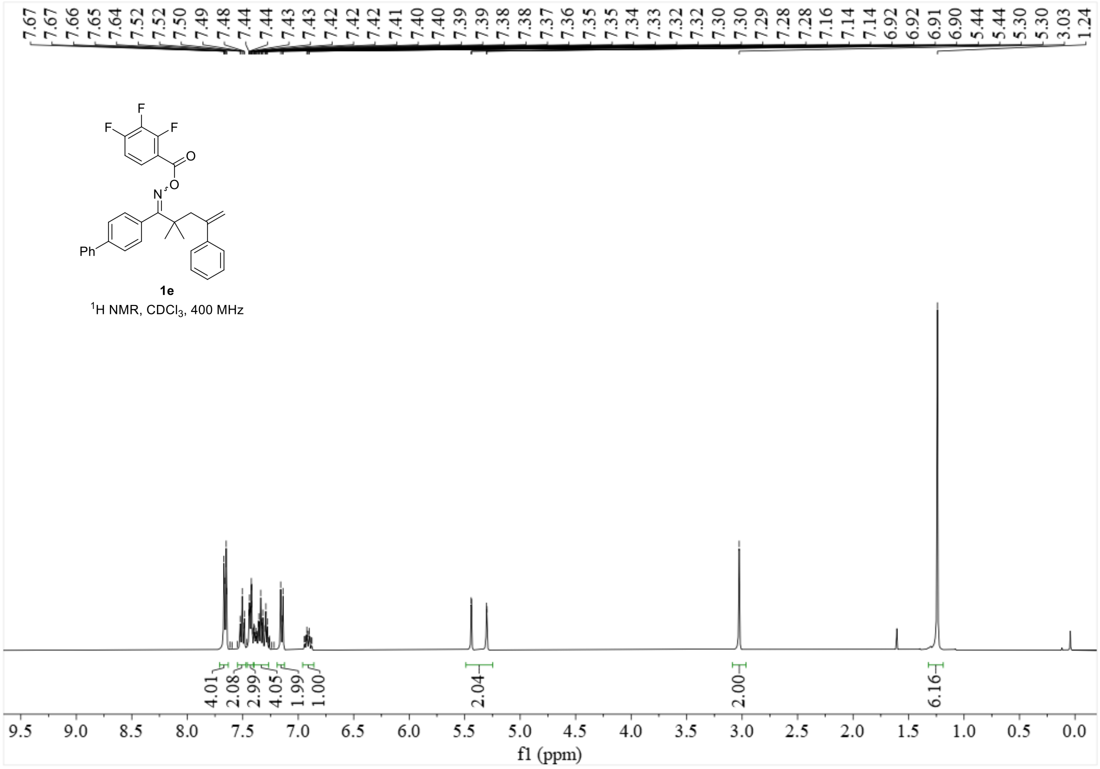
7.67 7.67 7.66 7.65 7.64 7.52 7.50 7.49 7.48 7.44 7.44 7.43 7.43 7.42 7.42 7.42 7.41 7.40 7.40 7.39 7.39 7.38 7.38 7.37 7.36 7.35 7.35 7.34 7.33 7.32 7.32 7.30 7.30 7.29 7.28 7.28 7.16 7.14 7.14 6.92 6.92 6.91 6.90 5.44 5.44 5.30 5.30 3.03 1.24
1e
^{1}H NMR, $CDCl_3$, 400 MHz
4.01 2.08 2.99 4.05 1.99 1.00 2.04 2.00 6.16
f1 (ppm)

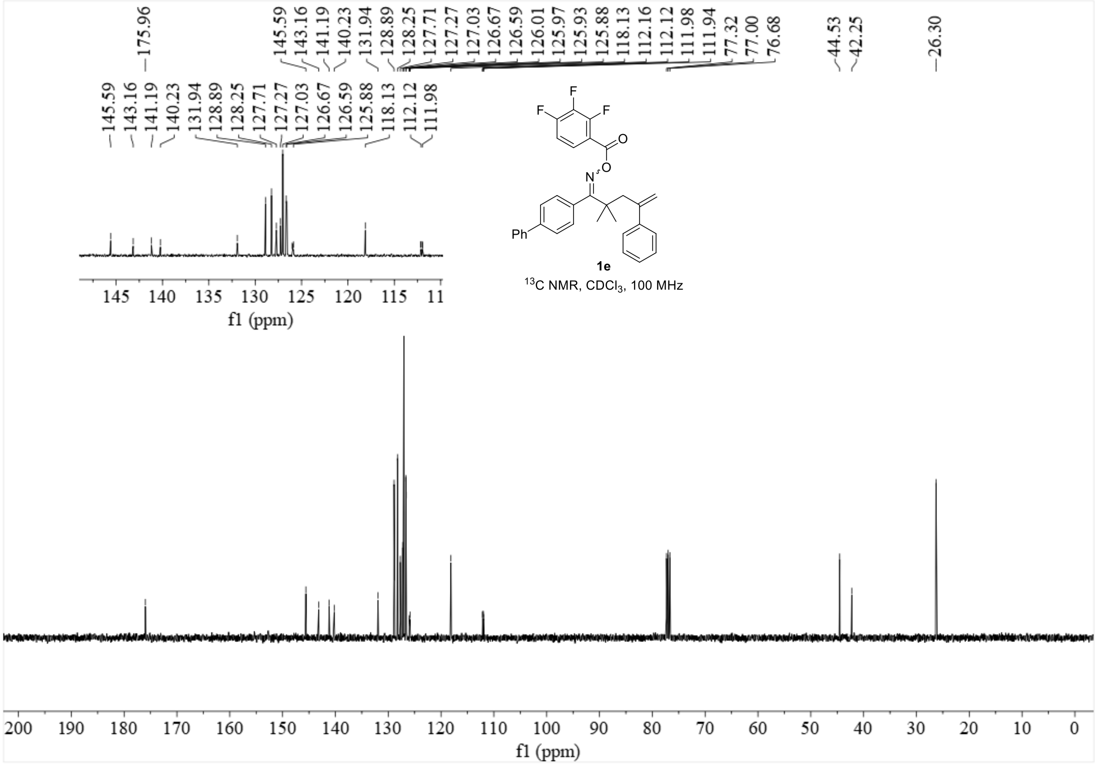
175.96 145.59 143.16 141.19 140.23 131.94 128.89 128.25 127.71 127.27 127.03 126.67 126.59 126.01 125.97 125.93 125.88 118.13 112.16 112.12 111.98 111.94 77.32 77.00 76.68 44.53 42.25 26.30
1e
^{13}C NMR, $CDCl_3$, 100 MHz
f1 (ppm)

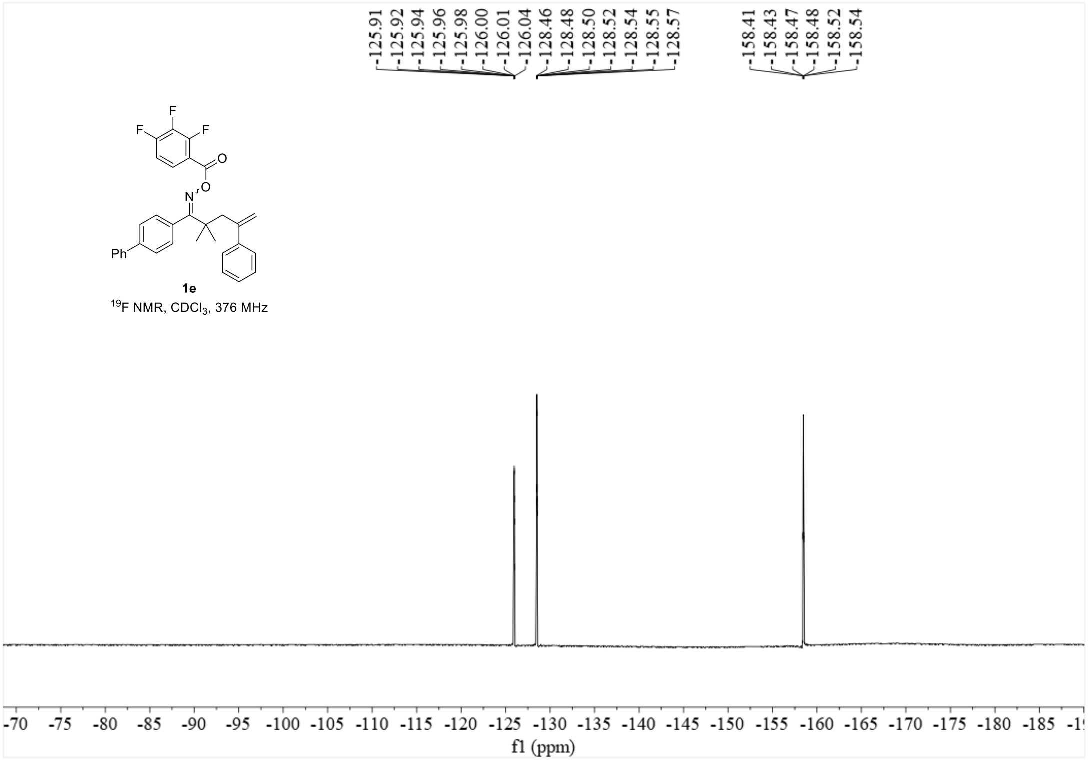
-125.91
-125.92
-125.94
-125.96
-125.98
-126.00
-126.01
-126.04
-128.46
-128.48
-128.50
-128.52
-128.54
-128.55
-128.57
-158.41
-158.43
-158.47
-158.48
-158.52
-158.54
F
F
F
O
O
N
Ph
1e
19F NMR, CDCl3, 376 MHz
-70 -75 -80 -85 -90 -95 -100 -105 -110 -115 -120 -125 -130 -135 -140 -145 -150 -155 -160 -165 -170 -175 -180 -185
f1 (ppm)

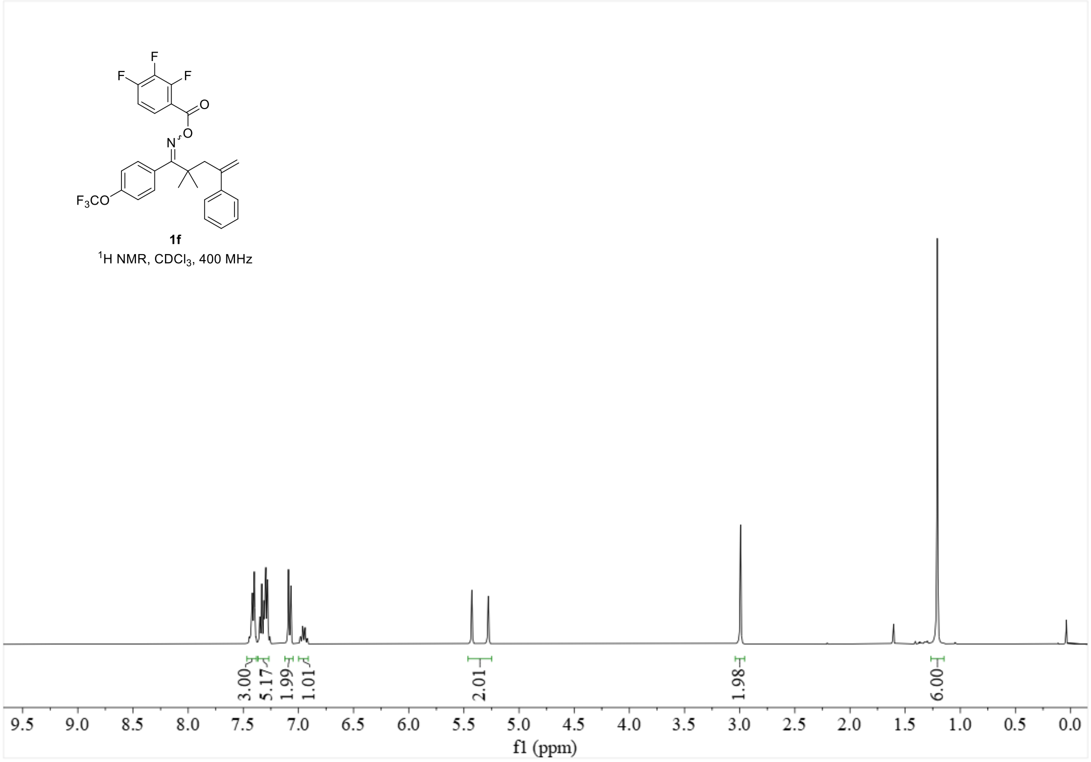
F
F
F
O
O
N
F3CO
1f
1H NMR, CDCl3, 400 MHz
3.00
5.17
1.99
1.01
2.01
1.98
6.00
9.5 9.0 8.5 8.0 7.5 7.0 6.5 6.0 5.5 5.0 4.5 4.0 3.5 3.0 2.5 2.0 1.5 1.0 0.5 0.0
f1 (ppm)

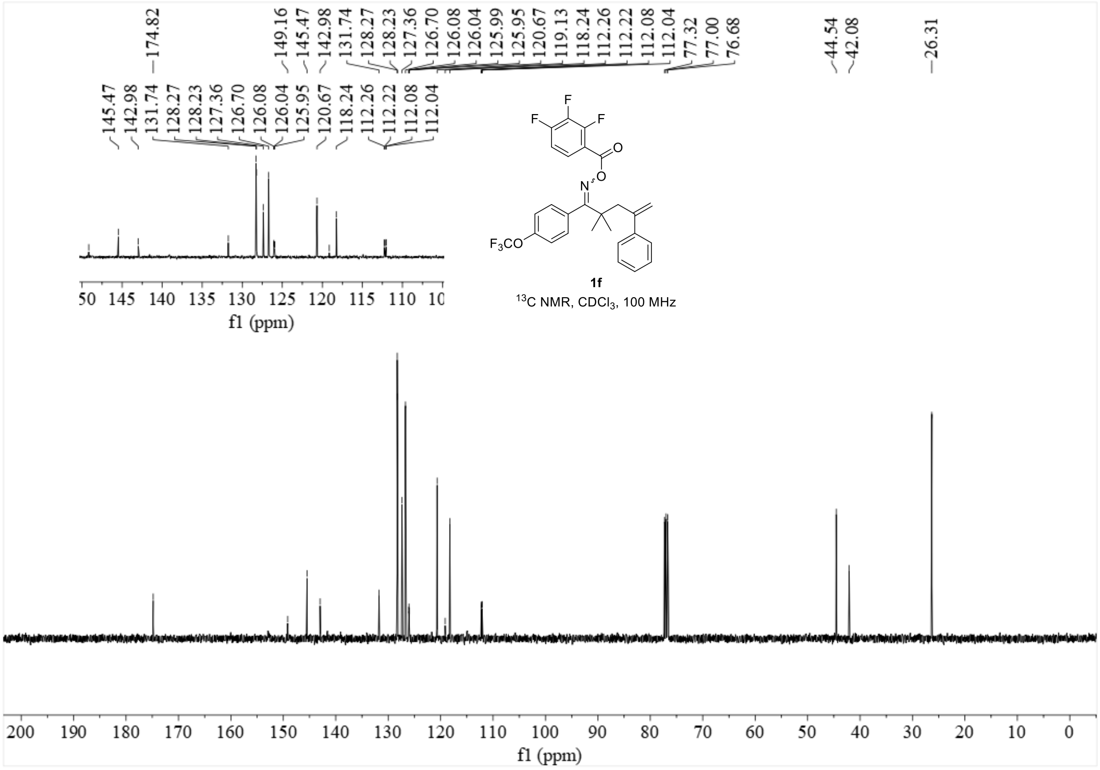
174.82
149.16
145.47
142.98
131.74
128.27
128.23
127.36
126.70
126.08
126.04
125.99
125.95
120.67
119.13
118.24
112.26
112.22
112.08
112.04
77.32
77.00
76.68
44.54
42.08
26.31
F
F
F
O
O
N
F3CO
1f
13C NMR, CDCl3, 100 MHz
f1 (ppm)
f1 (ppm)

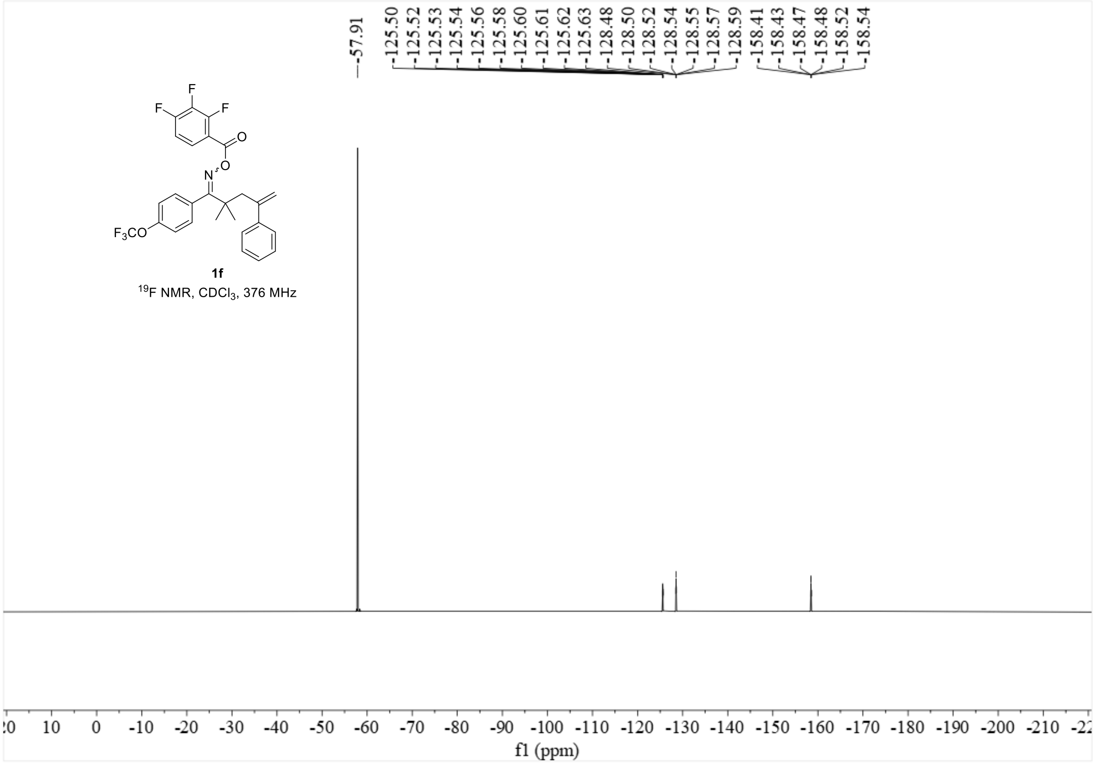
-57.91
-125.50
-125.52
-125.53
-125.54
-125.56
-125.58
-125.60
-125.61
-125.62
-125.63
-128.48
-128.50
-128.52
-128.54
-128.55
-128.57
-128.59
-158.41
-158.43
-158.47
-158.48
-158.52
-158.54
F
F
F
O
O
N
F3CO
1f
19F NMR, CDCl3, 376 MHz
f1 (ppm)

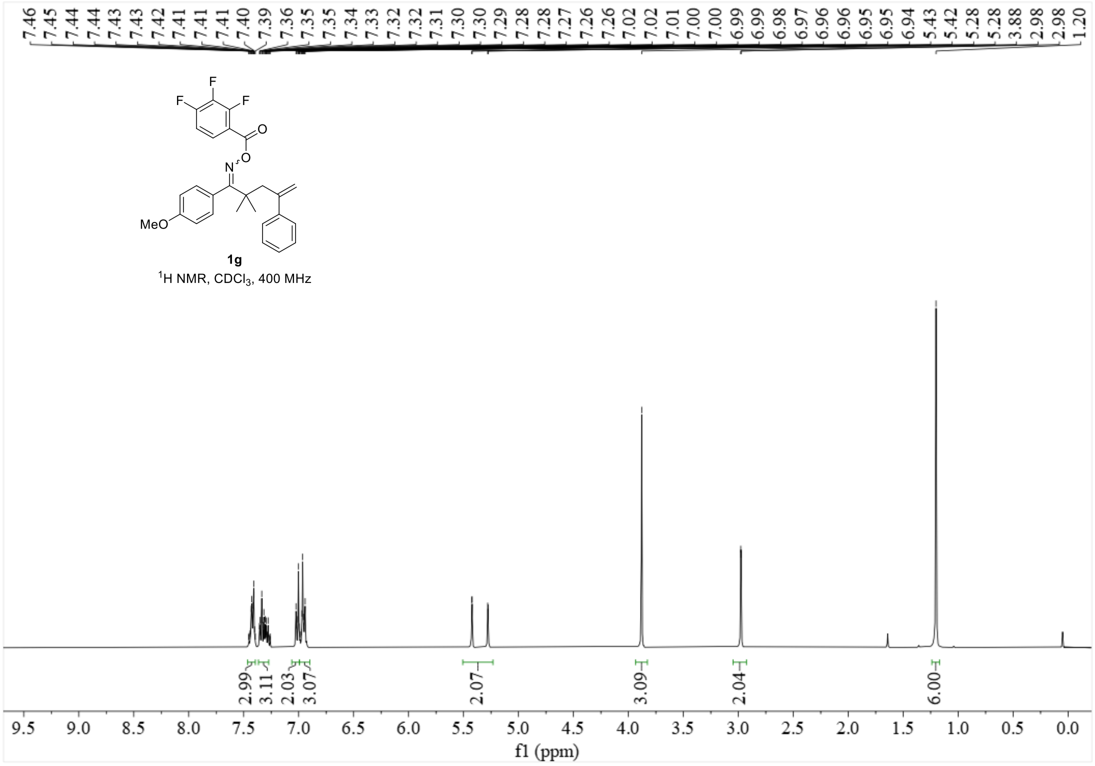
7.46 7.45 7.44 7.44 7.43 7.43 7.42 7.41 7.41 7.41 7.40 7.39 7.36 7.35 7.35 7.34 7.33 7.32 7.32 7.31 7.30 7.30 7.29 7.28 7.28 7.27 7.26 7.26 7.02 7.02 7.01 7.00 7.00 6.99 6.99 6.98 6.97 6.96 6.96 6.95 6.95 6.94 5.43 5.42 5.28 5.28 3.88 2.98 2.98 1.20
F
F
F
O
N
O
MeO
1g
1H NMR, CDCl3, 400 MHz
2.99 3.11 2.03 3.07 2.07 3.09 2.04 6.00
9.5 9.0 8.5 8.0 7.5 7.0 6.5 6.0 5.5 5.0 4.5 4.0 3.5 3.0 2.5 2.0 1.5 1.0 0.5 0.0
f1 (ppm)

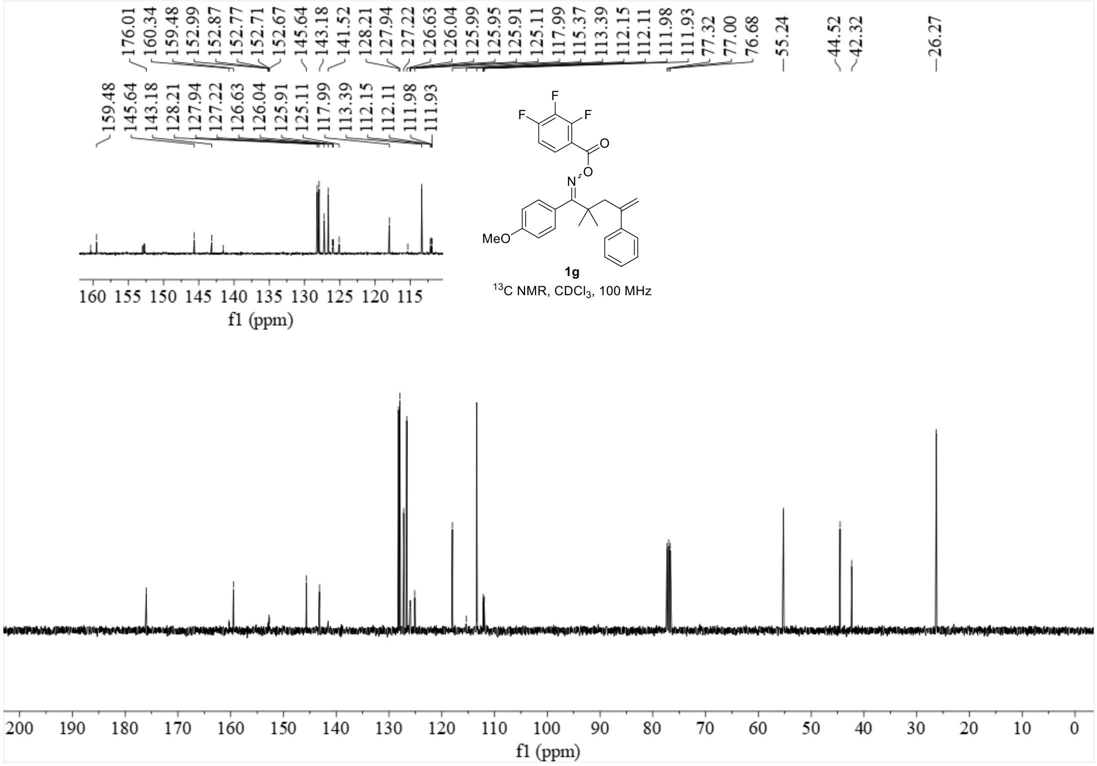
176.01 160.34 159.48 152.99 152.87 152.77 152.71 152.67 145.64 143.18 141.52 128.21 127.94 127.22 126.63 126.04 125.99 125.95 125.91 125.11 117.99 115.37 113.39 112.15 112.11 111.98 111.93 77.32 77.00 76.68 55.24 44.52 42.32 26.27
159.48 145.64 143.18 128.21 127.94 127.22 126.63 126.04 125.91 125.11 117.99 113.39 112.15 112.11 111.98 111.93
160 155 150 145 140 135 130 125 120 115
f1 (ppm)
F
F
F
O
N
O
MeO
1g
13C NMR, CDCl3, 100 MHz
200 190 180 170 160 150 140 130 120 110 100 90 80 70 60 50 40 30 20 10 0
f1 (ppm)

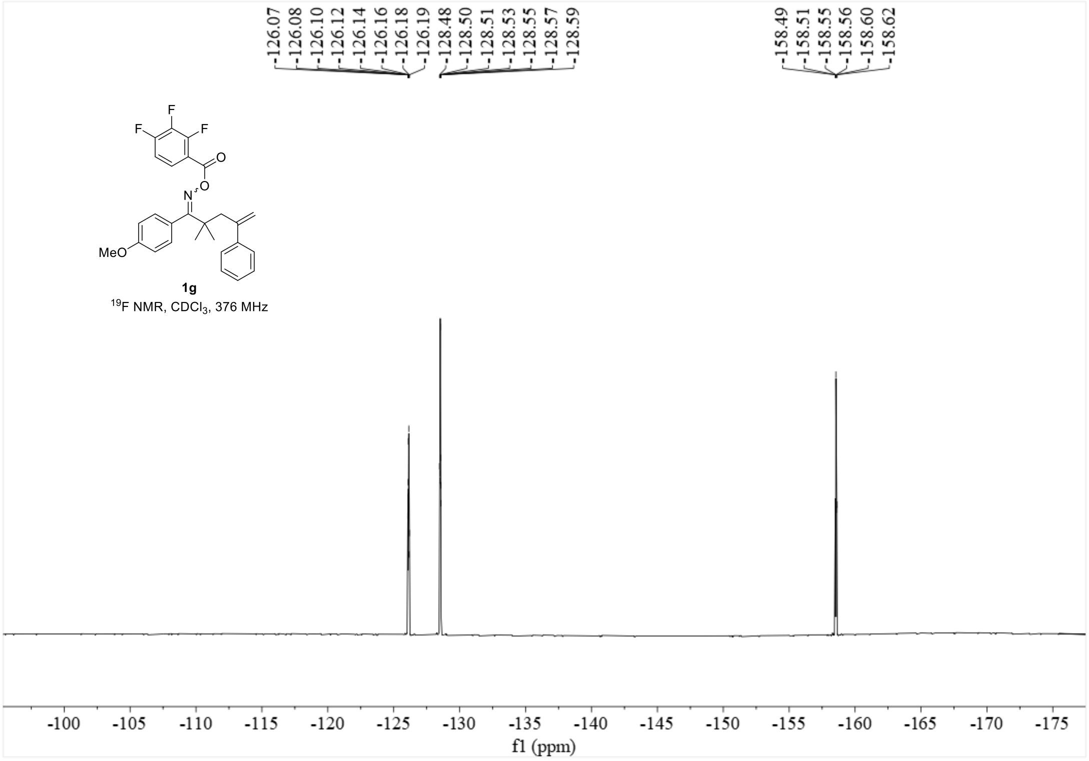
-126.07
-126.08
-126.10
-126.12
-126.14
-126.16
-126.18
-126.19
-128.48
-128.50
-128.51
-128.53
-128.55
-128.57
-128.59
-158.49
-158.51
-158.55
-158.56
-158.60
-158.62
1g
19F NMR, CDCl3, 376 MHz
f1 (ppm)

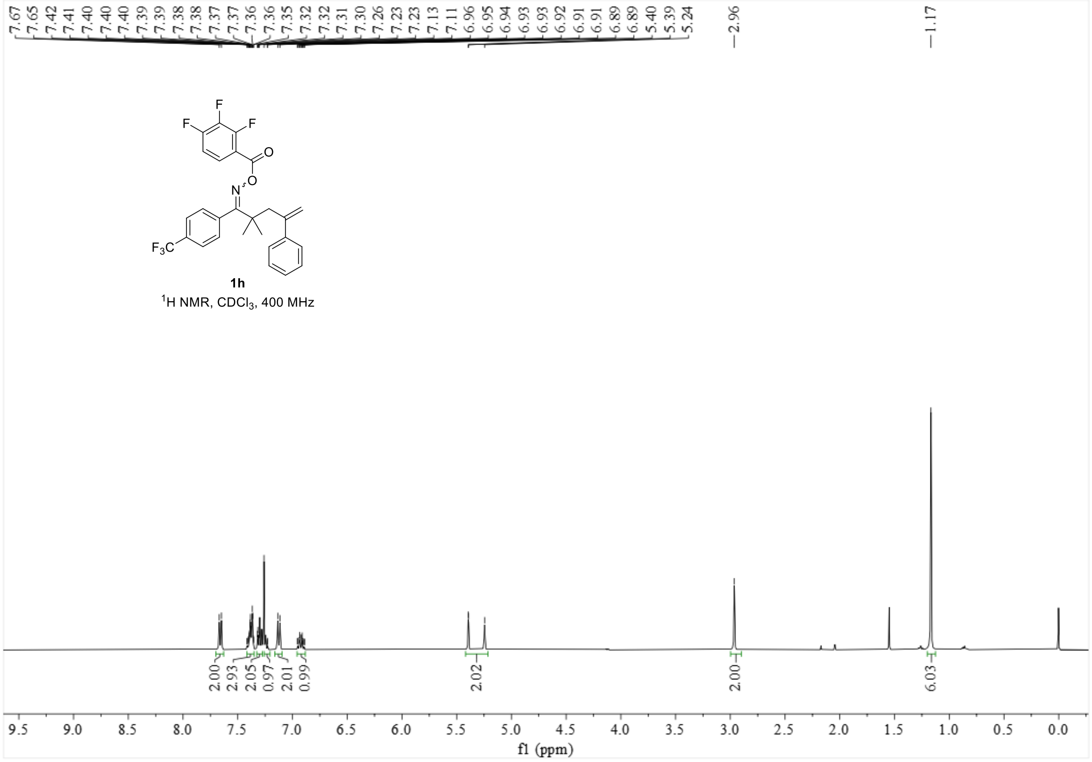
7.67
7.65
7.42
7.41
7.40
7.40
7.40
7.39
7.39
7.38
7.38
7.37
7.37
7.36
7.36
7.35
7.32
7.32
7.31
7.30
7.26
7.23
7.23
7.13
7.11
6.96
6.95
6.94
6.93
6.93
6.92
6.91
6.91
6.89
6.89
5.40
5.39
5.24
2.96
1.17
1h
1H NMR, CDCl3, 400 MHz
2.00
2.93
2.05
0.97
2.01
0.99
2.02
2.00
6.03
f1 (ppm)

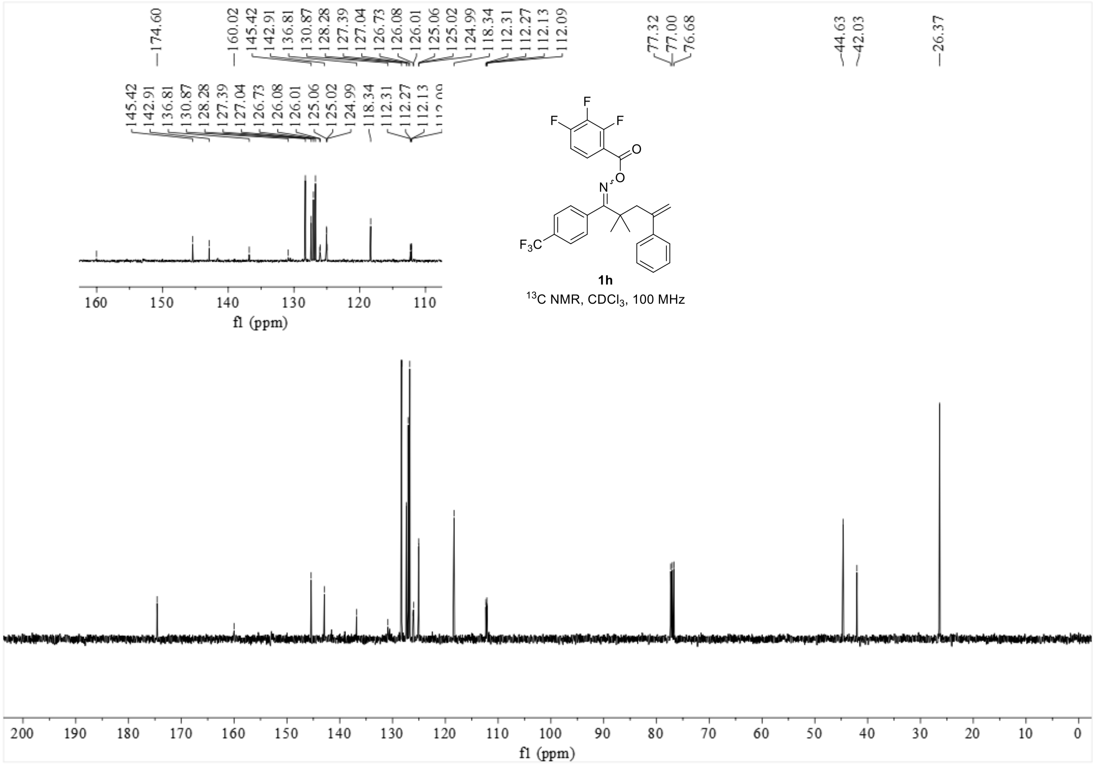

1h
^{13}C NMR, CDCl$_3$, 100 MHz
f1 (ppm)

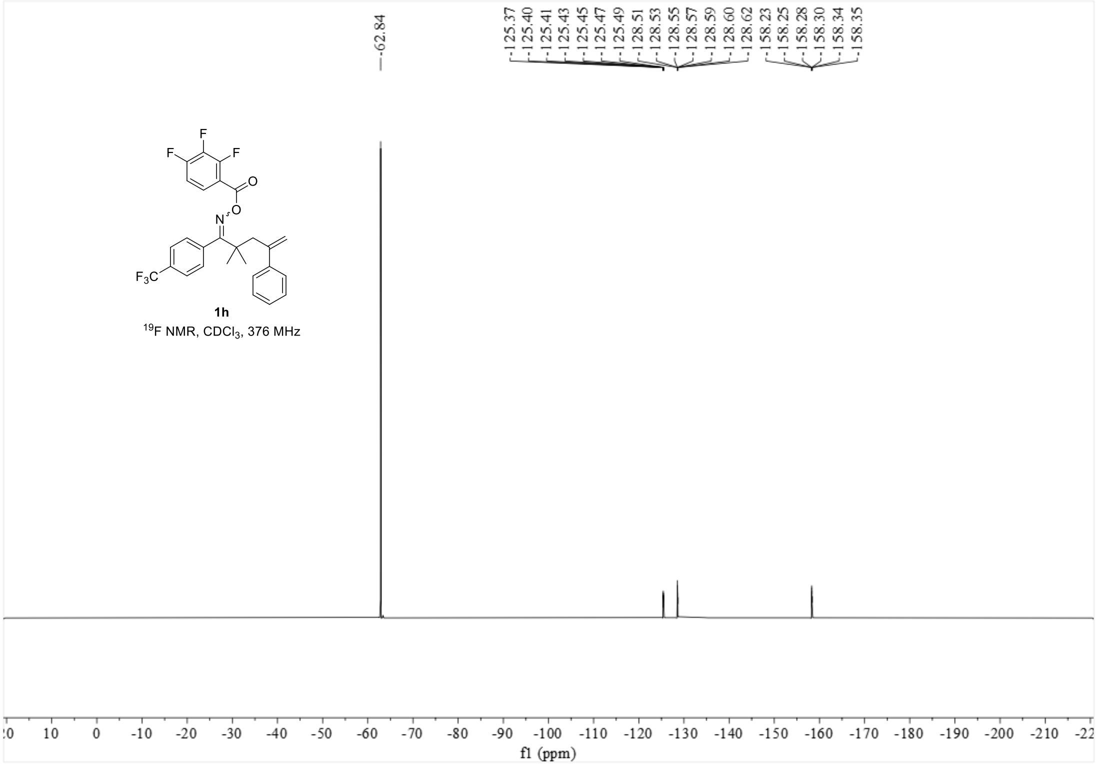

1h
^{19}F NMR, CDCl$_3$, 376 MHz
f1 (ppm)

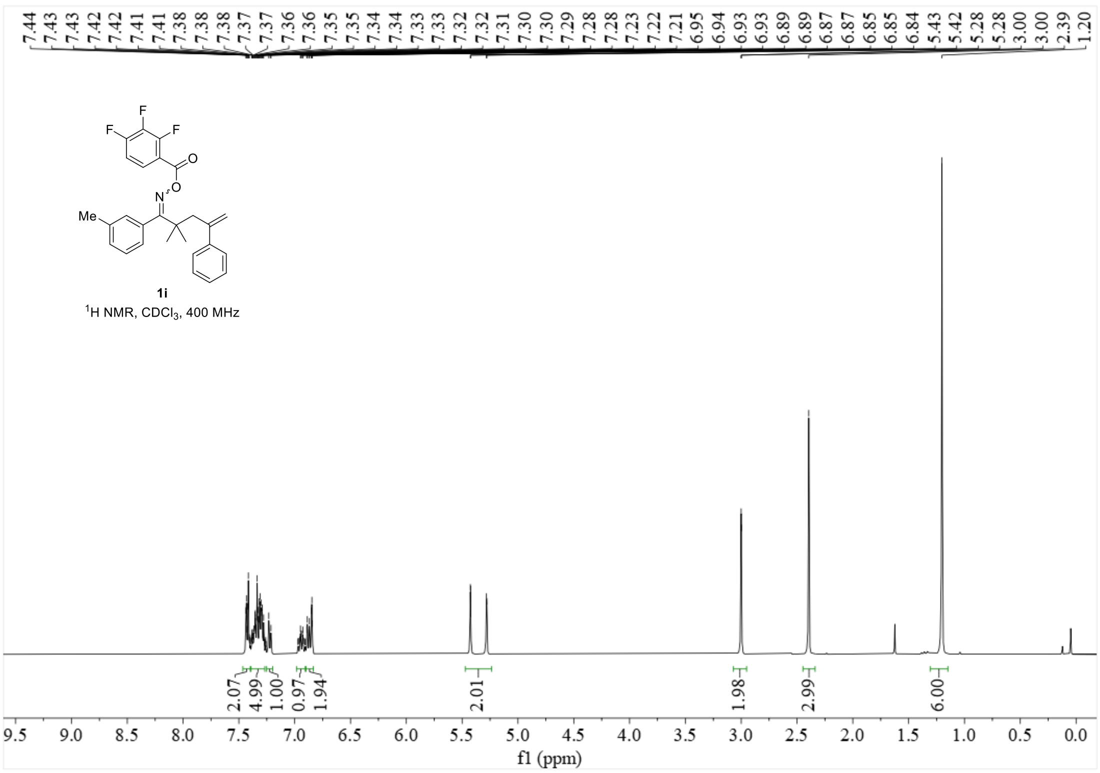
7.44 7.43 7.43 7.42 7.42 7.41 7.41 7.38 7.38 7.38 7.37 7.37 7.36 7.36 7.35 7.35 7.34 7.34 7.33 7.33 7.32 7.32 7.31 7.30 7.30 7.29 7.28 7.28 7.23 7.22 7.21 6.95 6.94 6.93 6.93 6.89 6.89 6.87 6.87 6.85 6.85 6.84 5.43 5.42 5.28 5.28 3.00 3.00 2.39 1.20
F
F
F
O
O
N
Me
1i
1H NMR, CDCl3, 400 MHz
2.07 4.99 1.00 0.97 1.94
2.01
1.98
2.99
6.00
9.5 9.0 8.5 8.0 7.5 7.0 6.5 6.0 5.5 5.0 4.5 4.0 3.5 3.0 2.5 2.0 1.5 1.0 0.5 0.0
f1 (ppm)

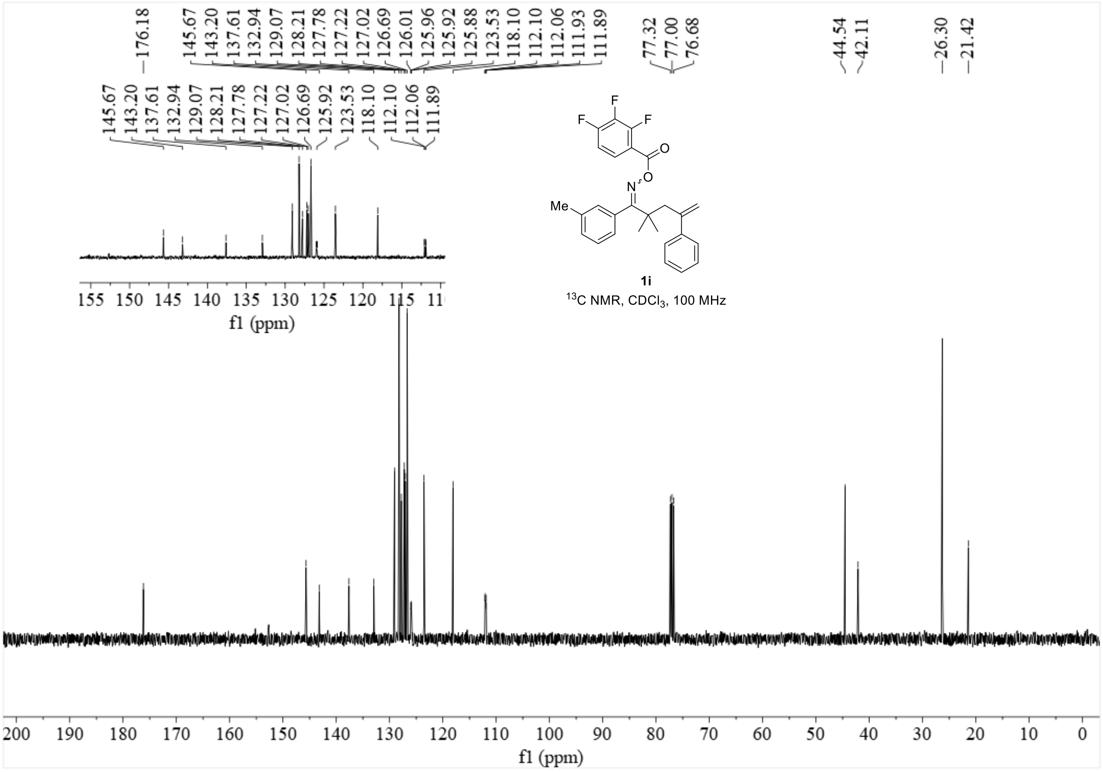
176.18
145.67 143.20 137.61 132.94 129.07 128.21 127.78 127.22 127.02 126.69 126.01 125.96 125.92 125.88 123.53 118.10 112.10 112.06 111.93 111.89
77.32 77.00 76.68
44.54 42.11
26.30 21.42
145.67 143.20 137.61 132.94 129.07 128.21 127.78 127.22 127.02 126.69 125.92 123.53 118.10 112.10 112.06 111.89
155 150 145 140 135 130 125 120 115 110
f1 (ppm)
F
F
F
O
O
N
Me
1i
13C NMR, CDCl3, 100 MHz
200 190 180 170 160 150 140 130 120 110 100 90 80 70 60 50 40 30 20 10 0
f1 (ppm)

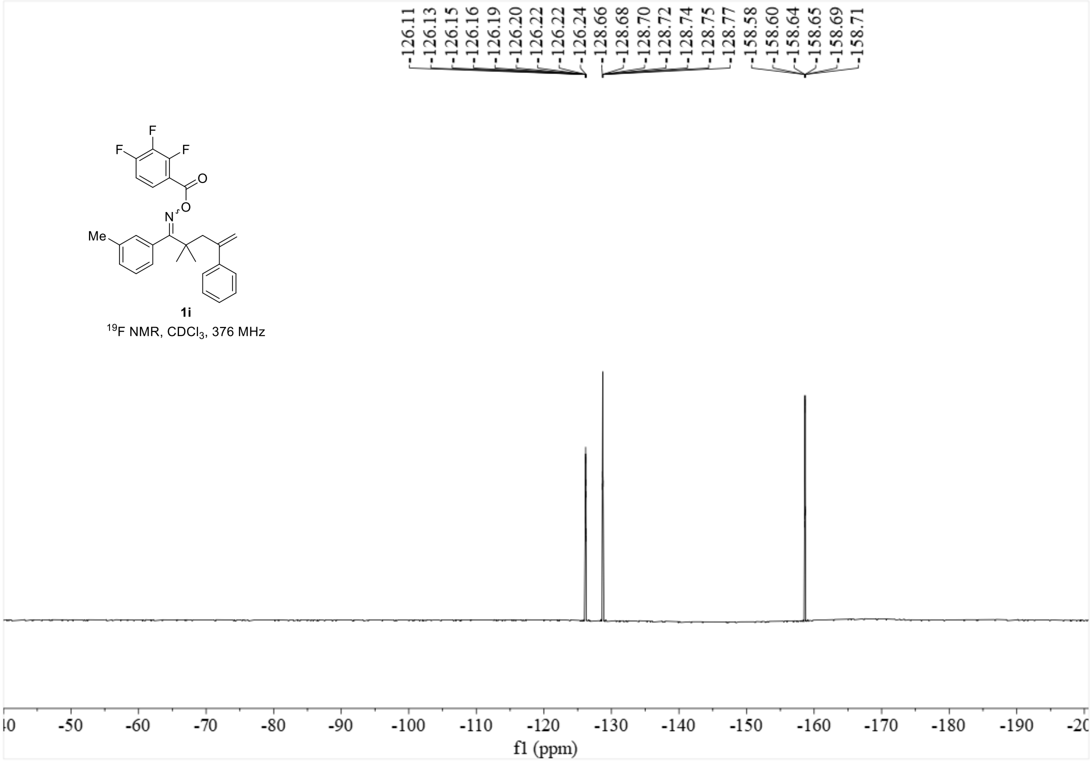

1i

^{19}F NMR, $CDCl_3$, 376 MHz

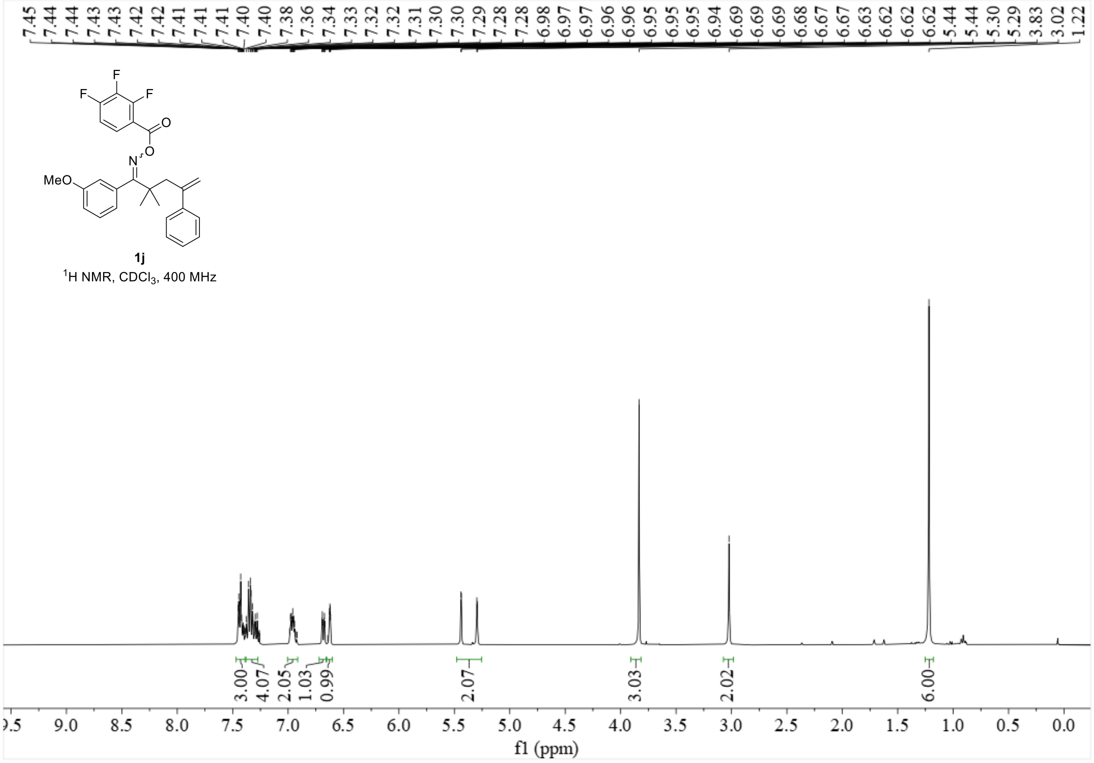

1j

^{1}H NMR, $CDCl_3$, 400 MHz

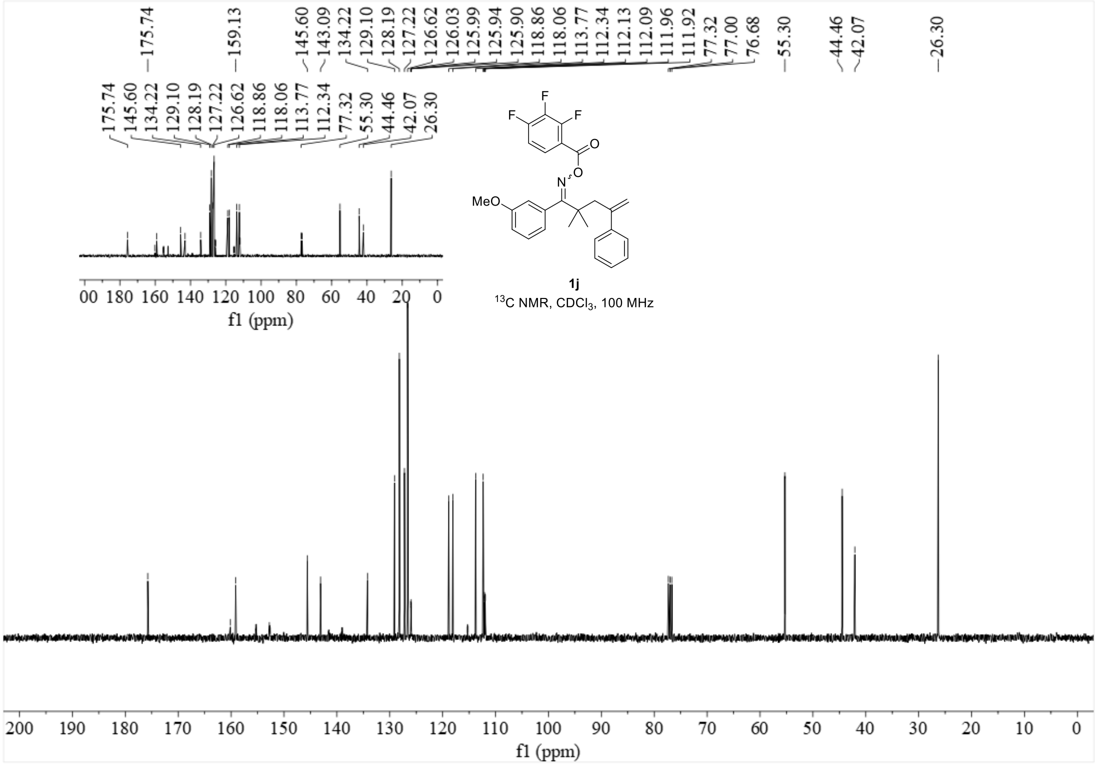
175.74
159.13
145.60
143.09
134.22
129.10
128.19
127.22
126.62
126.03
125.99
125.94
125.90
118.86
118.06
113.77
112.34
112.13
112.09
111.96
111.92
77.32
77.00
76.68
55.30
44.46
42.07
26.30
F
F
F
O
N
O
MeO
1j
^{13}C NMR, $CDCl_3$, 100 MHz
f1 (ppm)

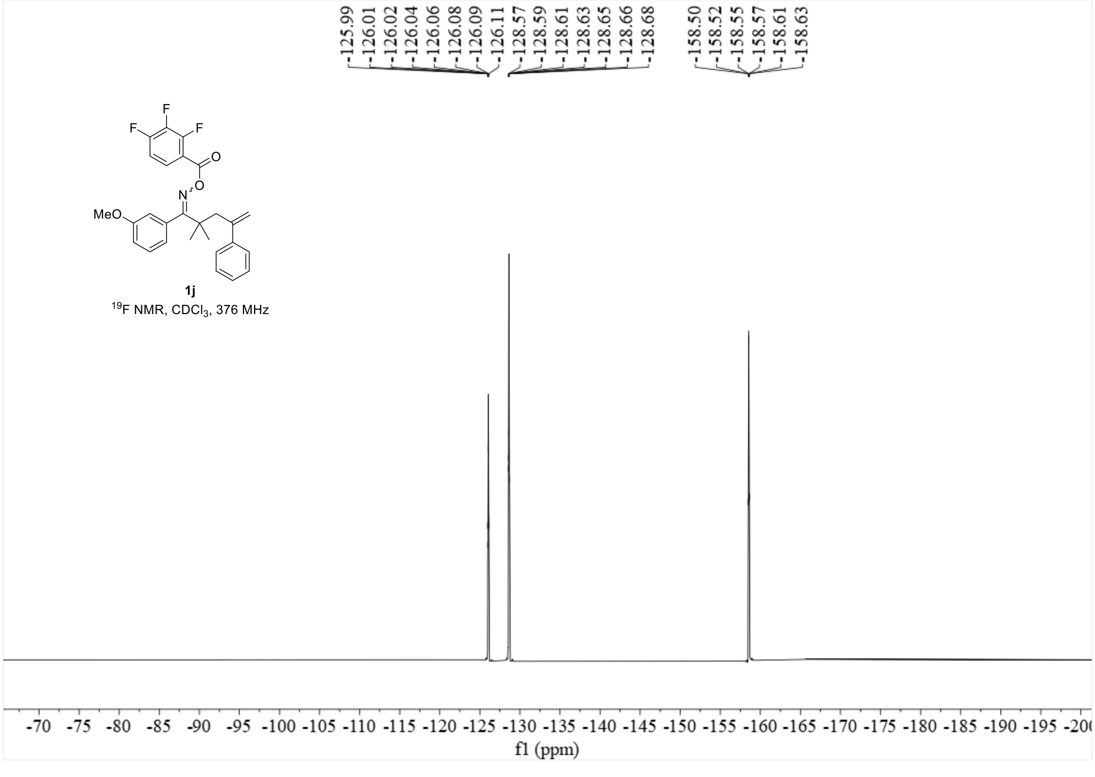
-125.99
-126.01
-126.02
-126.04
-126.06
-126.08
-126.09
-126.11
-128.57
-128.59
-128.61
-128.63
-128.65
-128.66
-128.68
-158.50
-158.52
-158.55
-158.57
-158.61
-158.63
F
F
F
O
N
O
MeO
1j
^{19}F NMR, $CDCl_3$, 376 MHz
f1 (ppm)

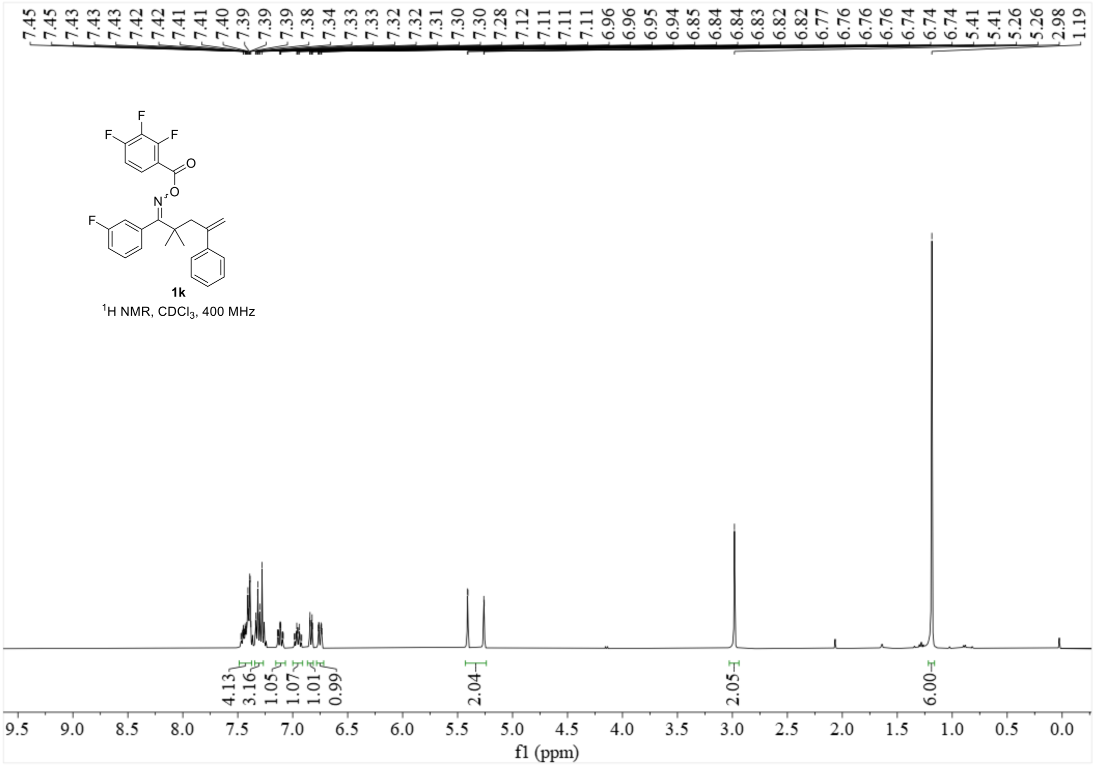
1k
^{1}H NMR, CDCl3, 400 MHz
f1 (ppm)

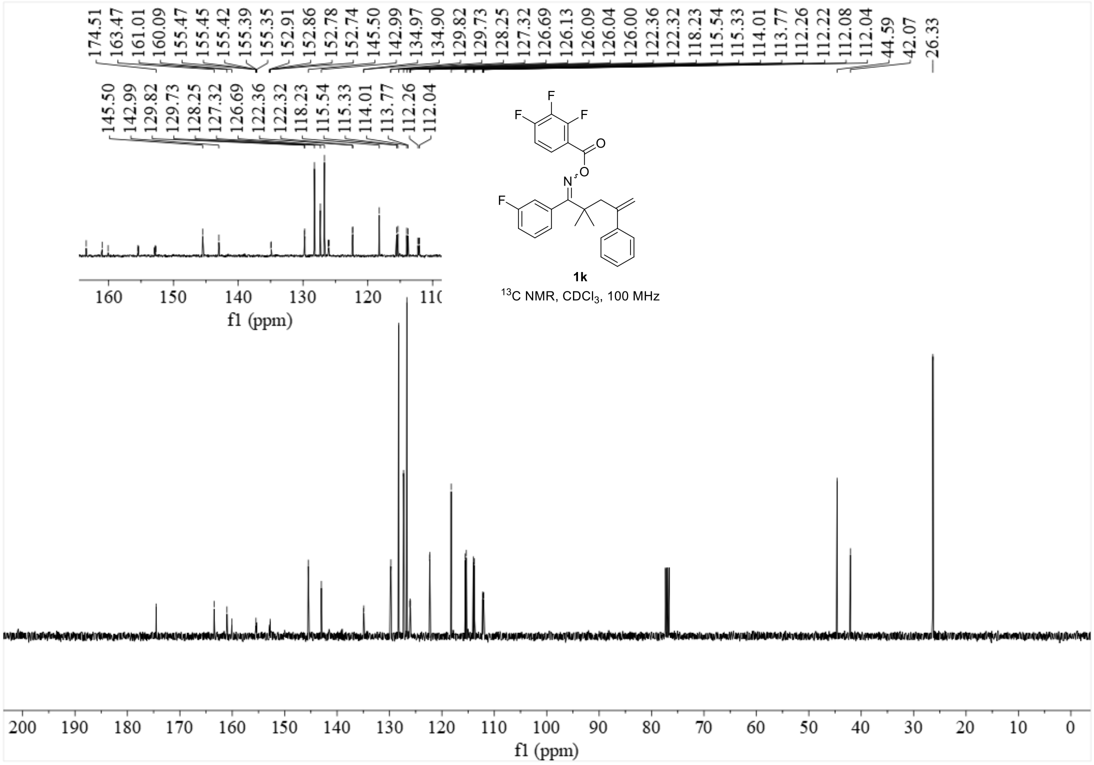
1k
^{13}C NMR, CDCl3, 100 MHz
f1 (ppm)

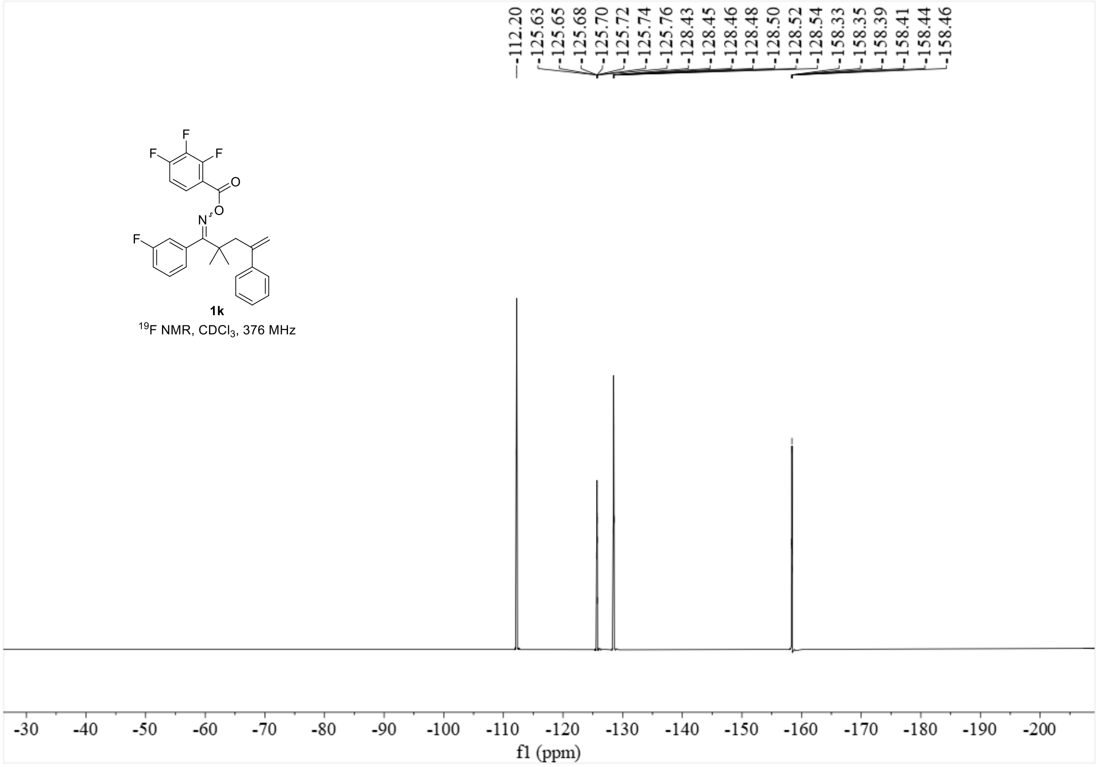
-112.20
-125.63 -125.65 -125.68 -125.70 -125.72 -125.74 -125.76
-128.43 -128.45 -128.46 -128.48 -128.50 -128.52 -128.54
-158.33 -158.35 -158.39 -158.41 -158.44 -158.46
1k
19F NMR, CDCl3, 376 MHz
-30 -40 -50 -60 -70 -80 -90 -100 -110 -120 -130 -140 -150 -160 -170 -180 -190 -200
f1 (ppm)

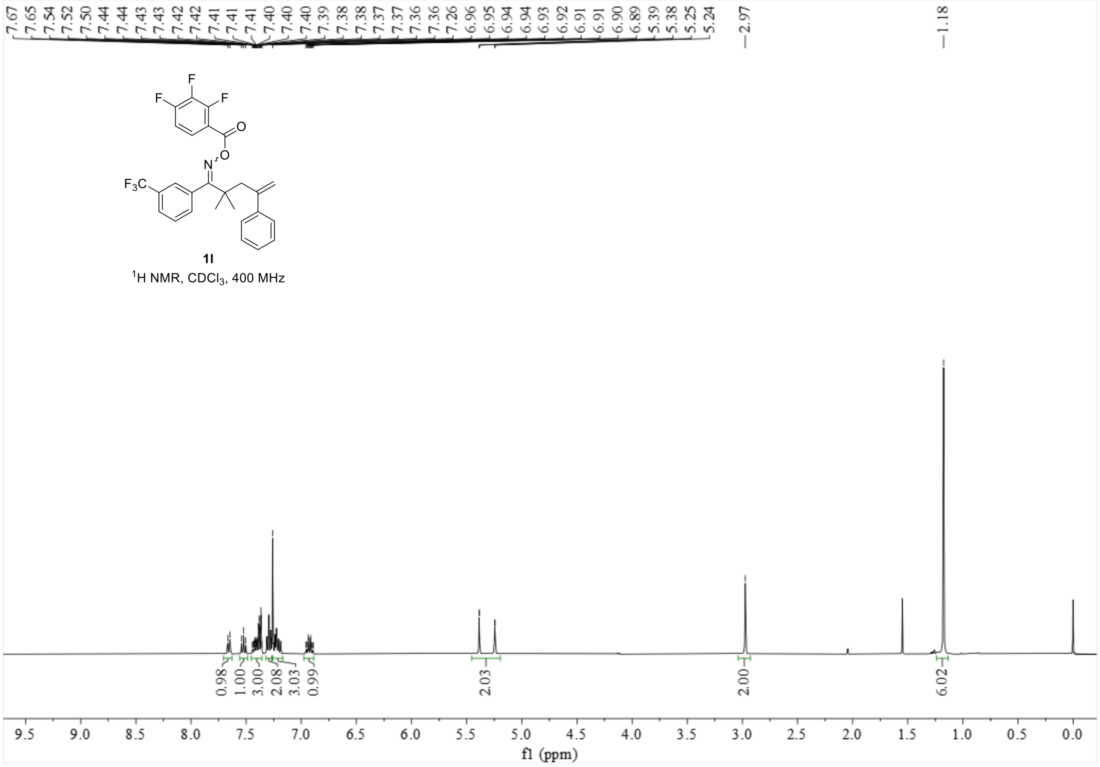
7.67 7.65 7.54 7.52 7.50 7.44 7.44 7.43 7.43 7.42 7.42 7.41 7.41 7.41 7.40 7.40 7.40 7.39 7.38 7.38 7.37 7.36 7.26 6.96 6.95 6.94 6.94 6.93 6.92 6.91 6.91 6.90 6.89 5.39 5.38 5.25 5.24
2.97
1.18
1l
1H NMR, CDCl3, 400 MHz
0.98 1.00 3.00 2.08 3.03 0.99
2.03
2.00
6.02
9.5 9.0 8.5 8.0 7.5 7.0 6.5 6.0 5.5 5.0 4.5 4.0 3.5 3.0 2.5 2.0 1.5 1.0 0.5 0.0
f1 (ppm)

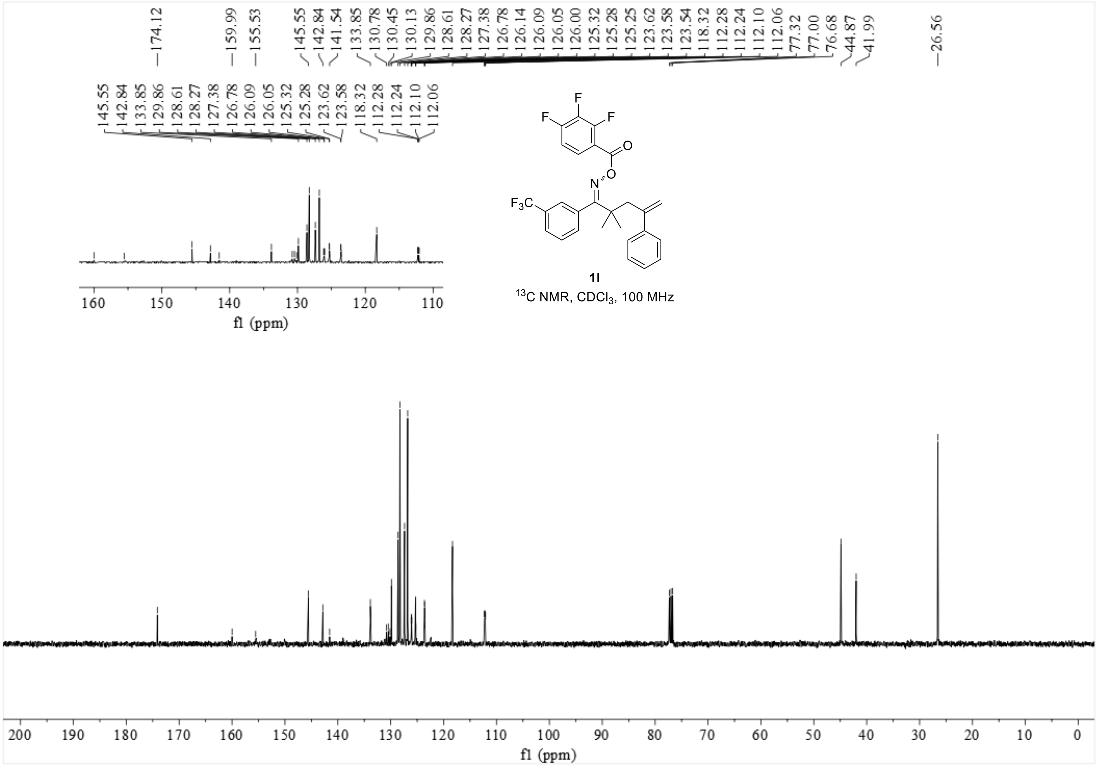
174.12
159.99
155.53
145.55
142.84
141.54
133.85
130.78
130.45
130.13
129.86
128.61
128.27
127.38
126.78
126.14
126.09
126.05
126.00
125.32
125.28
125.25
123.62
123.58
123.54
118.32
112.28
112.24
112.10
112.06
77.32
77.00
76.68
44.87
41.99
26.56
F
F
F
O
O
N
F_3C
1l
^{13}C NMR, $CDCl_3$, 100 MHz
f1 (ppm)

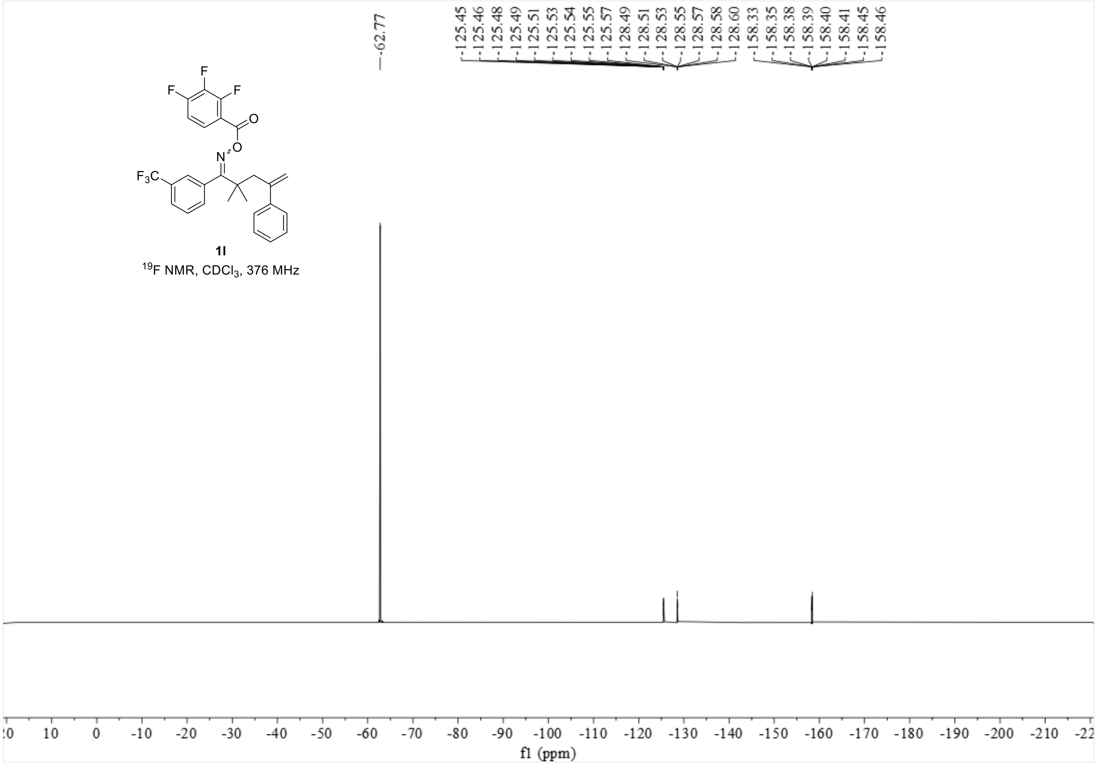
-62.77
-125.45
-125.46
-125.48
-125.49
-125.51
-125.53
-125.54
-125.55
-125.57
-128.49
-128.51
-128.53
-128.55
-128.57
-128.58
-128.60
-158.33
-158.35
-158.38
-158.39
-158.40
-158.41
-158.45
-158.46
F
F
F
O
O
N
F_3C
1l
^{19}F NMR, $CDCl_3$, 376 MHz
f1 (ppm)

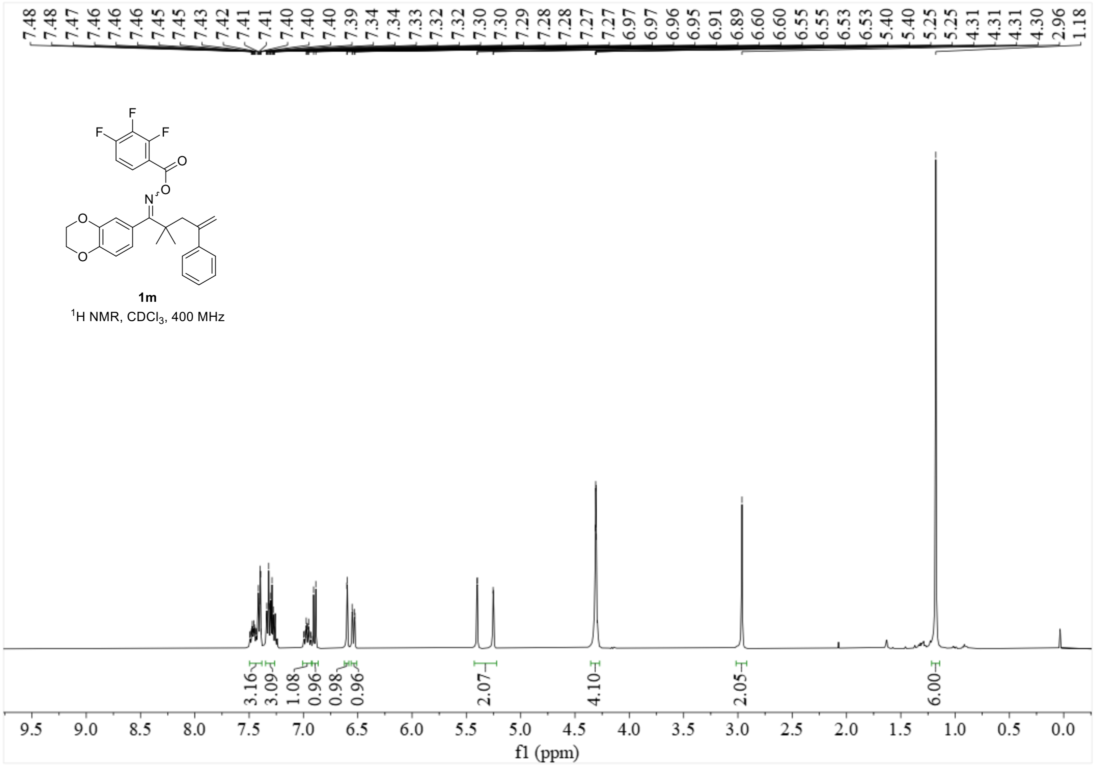
7.48 7.48 7.47 7.46 7.46 7.45 7.45 7.43 7.42 7.41 7.41 7.40 7.40 7.39 7.34 7.34 7.33 7.32 7.32 7.30 7.30 7.29 7.28 7.28 7.27 7.27 6.97 6.97 6.96 6.95 6.91 6.89 6.60 6.60 6.55 6.55 6.53 6.53 5.40 5.40 5.25 5.25 4.31 4.31 4.31 4.30 2.96 1.18
F
F
F
O
N
O
O
O
1m
1H NMR, CDCl3, 400 MHz
3.16 3.09 1.08 0.96 0.98 0.96 2.07 4.10 2.05 6.00
9.5 9.0 8.5 8.0 7.5 7.0 6.5 6.0 5.5 5.0 4.5 4.0 3.5 3.0 2.5 2.0 1.5 1.0 0.5 0.0
f1 (ppm)

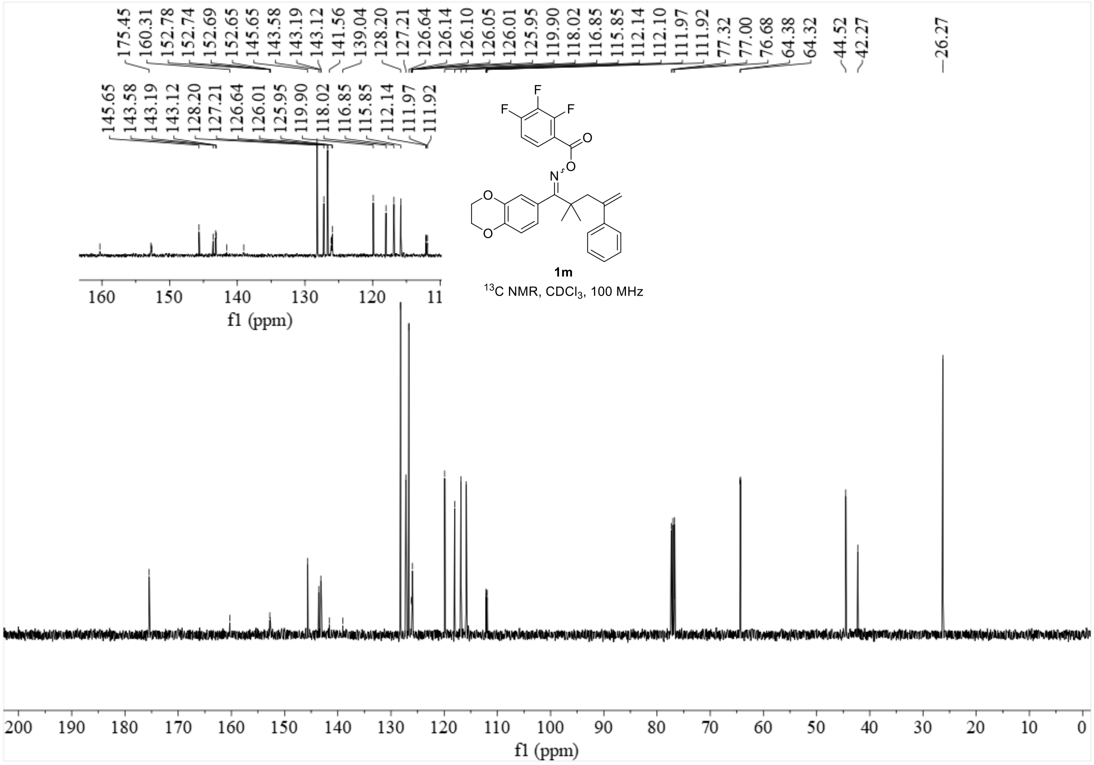
175.45 160.31 152.78 152.74 152.69 152.65 145.65 143.58 143.19 143.12 141.56 139.04 128.20 127.21 126.64 126.14 126.10 126.05 126.01 125.95 119.90 118.02 116.85 115.85 112.14 112.10 111.97 111.92 77.32 77.00 76.68 64.38 64.32 44.52 42.27 26.27
145.65 143.58 143.19 143.12 128.20 127.21 126.64 126.01 125.95 119.90 118.02 116.85 115.85 112.14 111.97 111.92
160 150 140 130 120 11
f1 (ppm)
F
F
F
O
N
O
O
O
1m
13C NMR, CDCl3, 100 MHz
200 190 180 170 160 150 140 130 120 110 100 90 80 70 60 50 40 30 20 10 0
f1 (ppm)

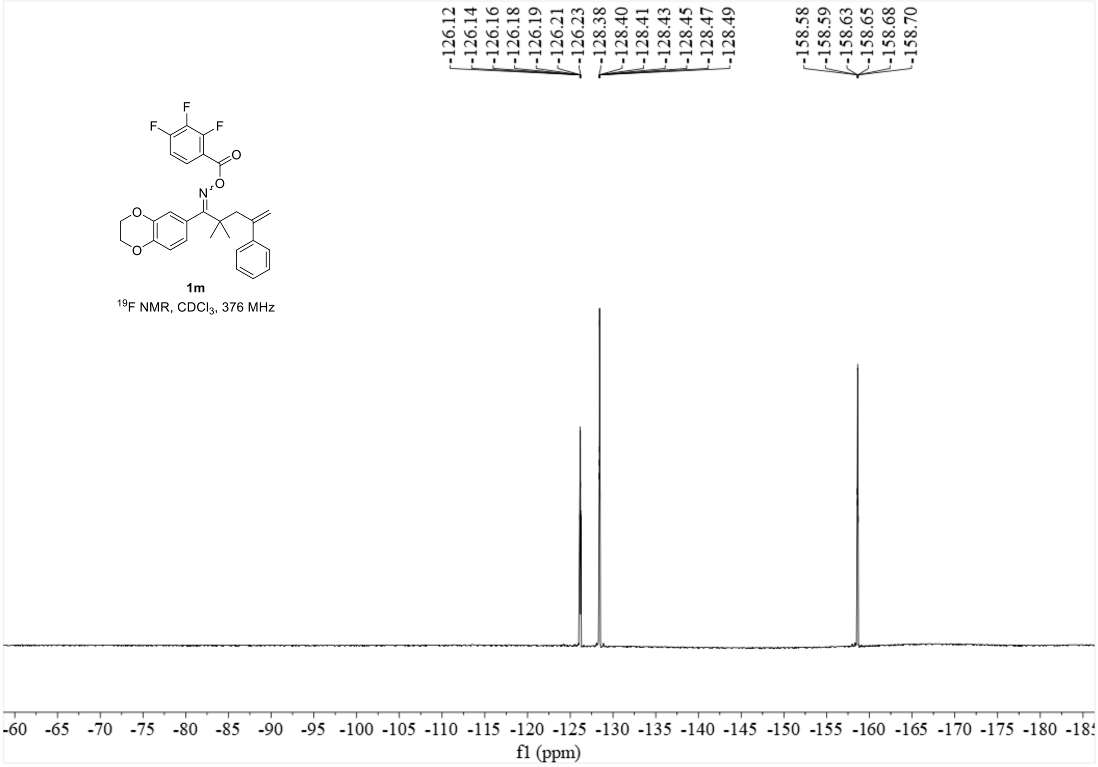

1m

^{19}F NMR, CDCl$_3$, 376 MHz

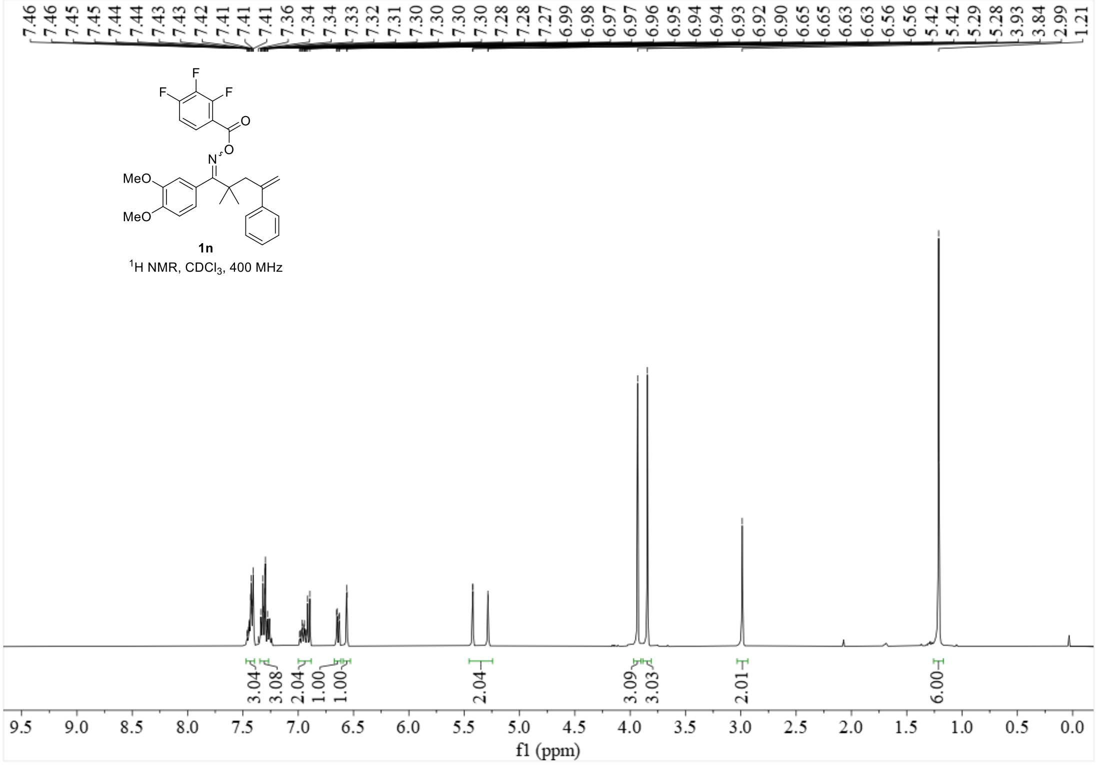

1n

^{1}H NMR, CDCl$_3$, 400 MHz

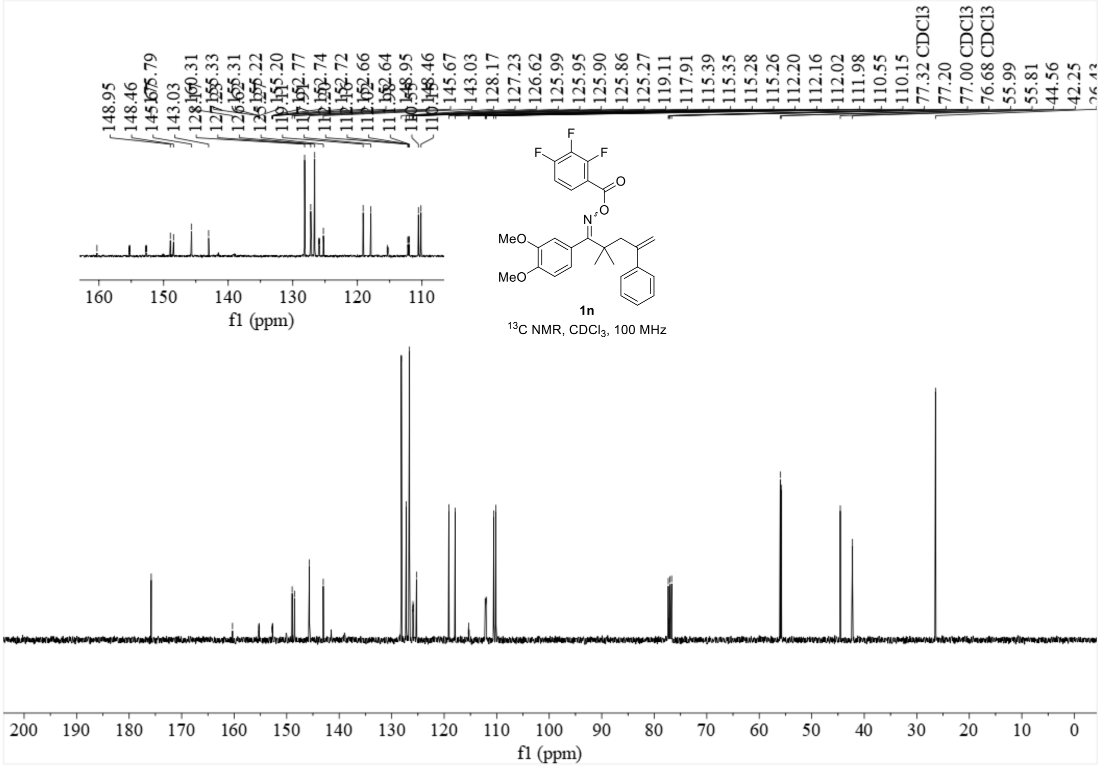
148.95
148.46
143.03
145.67
143.03
128.17
127.23
126.62
125.99
125.95
125.90
125.86
125.27
119.11
117.91
115.39
115.35
115.28
115.26
112.20
112.16
112.02
111.98
110.55
110.15
77.32 CDCl3
77.20
77.00 CDCl3
76.68 CDCl3
55.99
55.81
44.56
42.25
MeO
MeO
F
F
F
O
N
O
1n
^{13}C NMR, CDCl$_3$, 100 MHz
f1 (ppm)

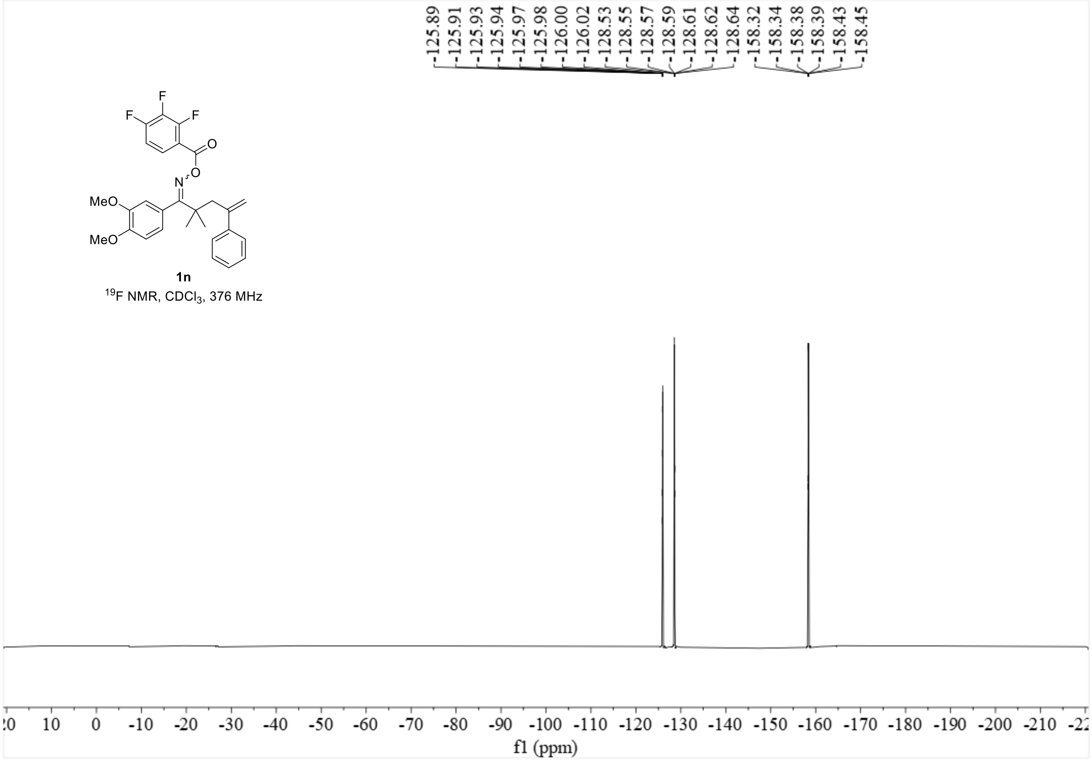
-125.89
-125.91
-125.93
-125.94
-125.97
-125.98
-126.00
-126.02
-128.53
-128.55
-128.57
-128.59
-128.61
-128.62
-128.64
-158.32
-158.34
-158.38
-158.39
-158.43
-158.45
MeO
MeO
F
F
F
O
N
O
1n
^{19}F NMR, CDCl$_3$, 376 MHz
f1 (ppm)

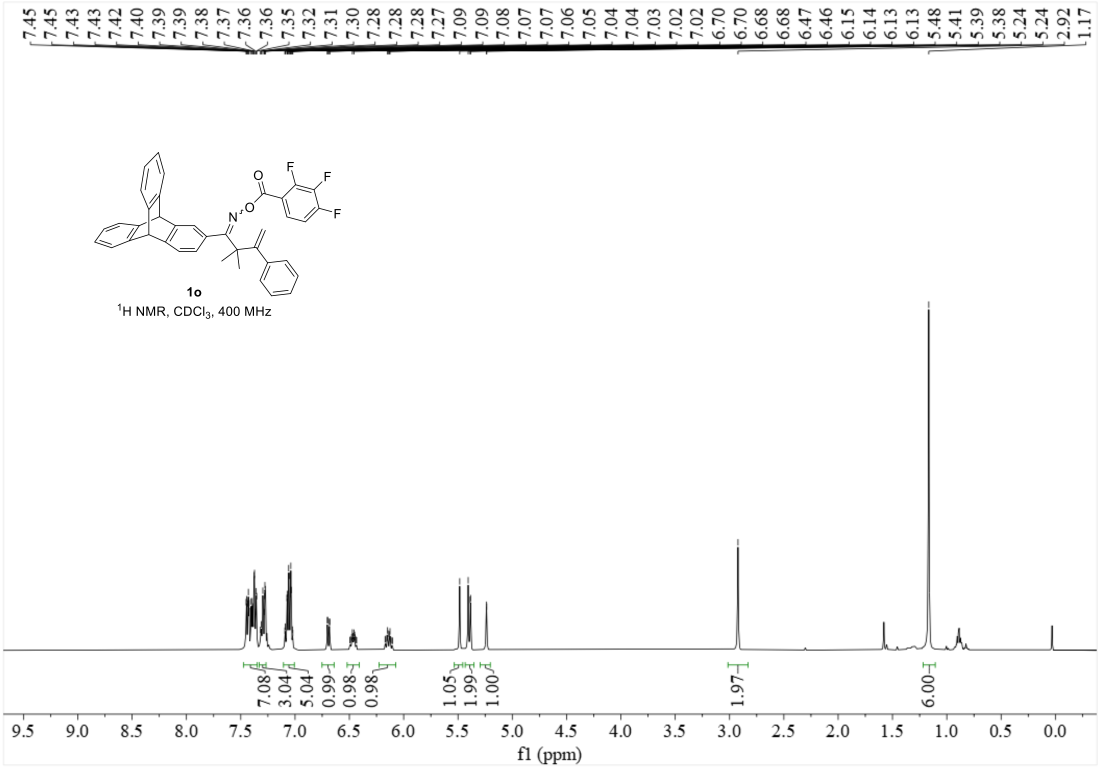
7.45 7.45 7.43 7.43 7.42 7.40 7.39 7.39 7.38 7.37 7.36 7.36 7.35 7.32 7.31 7.30 7.28 7.28 7.28 7.27 7.09 7.09 7.08 7.07 7.07 7.06 7.05 7.04 7.04 7.03 7.02 7.02 6.70 6.70 6.68 6.68 6.47 6.46 6.15 6.14 6.13 6.13 5.48 5.41 5.39 5.38 5.24 5.24 2.92 1.17
1o
^{1}H NMR, CDCl$_3$, 400 MHz
7.08 3.04 5.04 0.99 0.98 0.98 1.05 1.99 1.00 1.97 6.00
f1 (ppm)

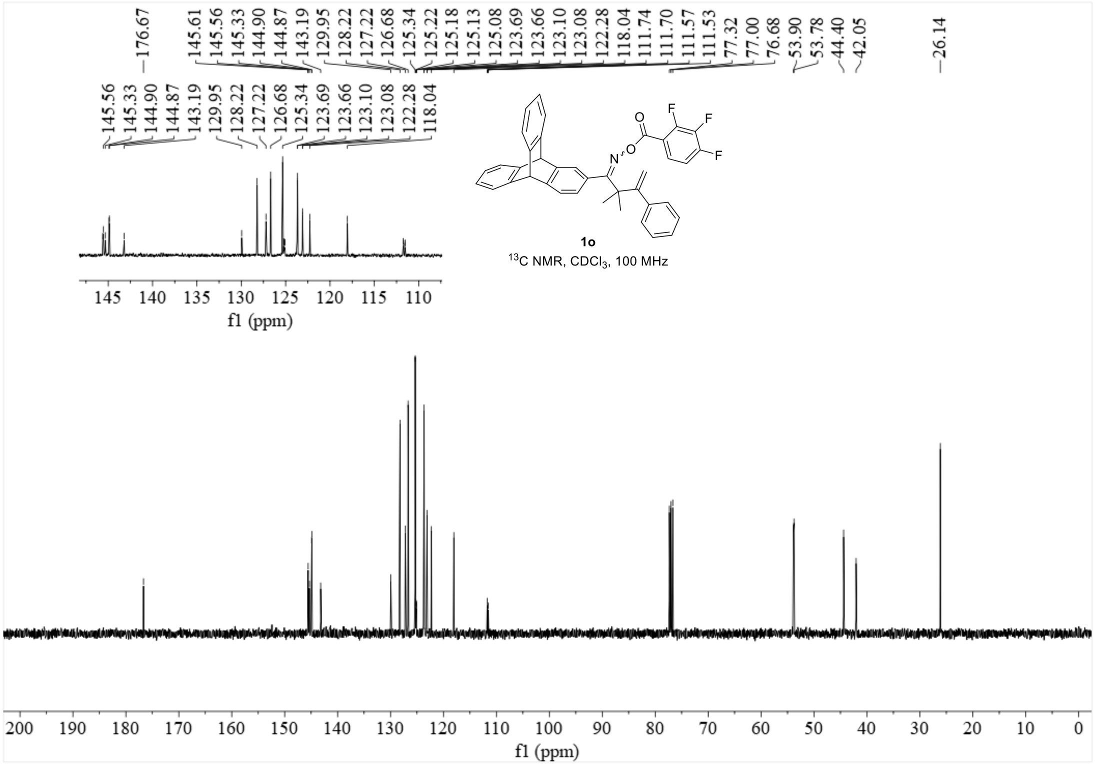
176.67 145.61 145.56 145.33 144.90 144.87 143.19 129.95 128.22 127.22 126.68 125.34 125.22 125.18 125.13 125.08 123.69 123.66 123.10 123.08 122.28 118.04 111.74 111.70 111.57 111.53 77.32 77.00 76.68 53.90 53.78 44.40 42.05 26.14
145.56 145.33 144.90 144.87 143.19 129.95 128.22 127.22 126.68 125.34 123.69 123.66 123.10 123.08 122.28 118.04
1o
^{13}C NMR, CDCl$_3$, 100 MHz
f1 (ppm)

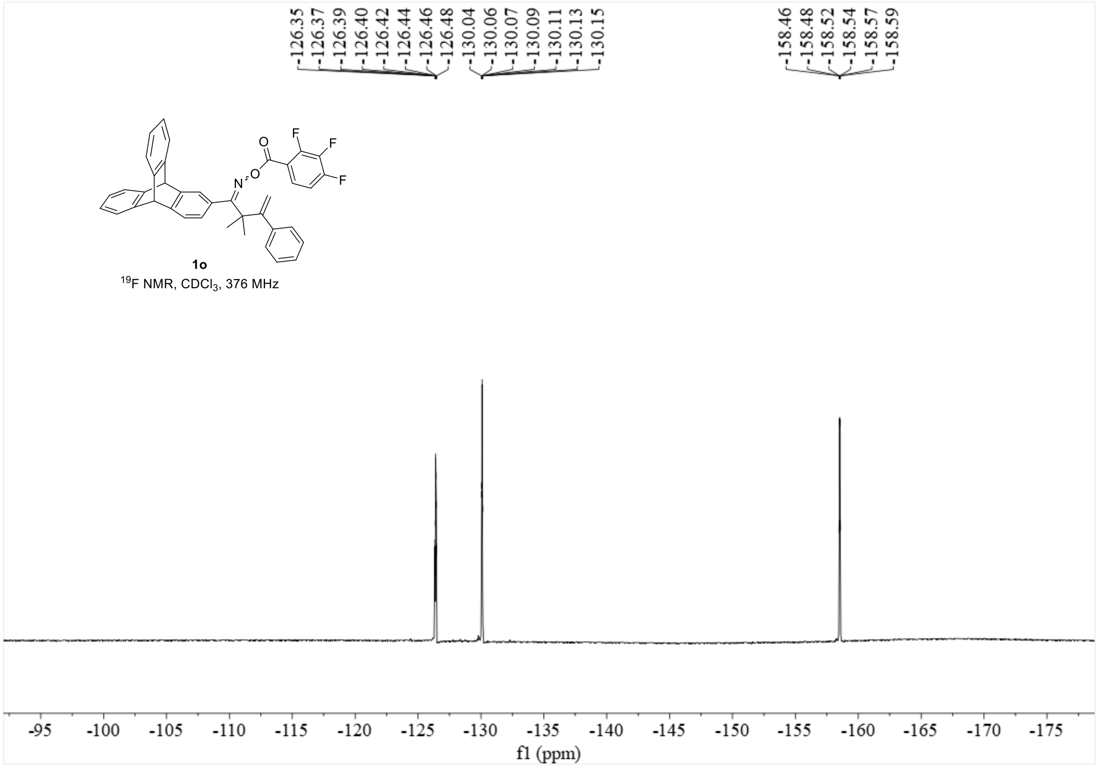
-126.35 -126.37 -126.39 -126.40 -126.42 -126.44 -126.46 -126.48 -130.04 -130.06 -130.07 -130.09 -130.11 -130.13 -130.15
-158.46 -158.48 -158.52 -158.54 -158.57 -158.59
O
F
F
F
N
O
1o
^{19}F NMR, CDCl$_3$, 376 MHz
-95 -100 -105 -110 -115 -120 -125 -130 -135 -140 -145 -150 -155 -160 -165 -170 -175
f1 (ppm)

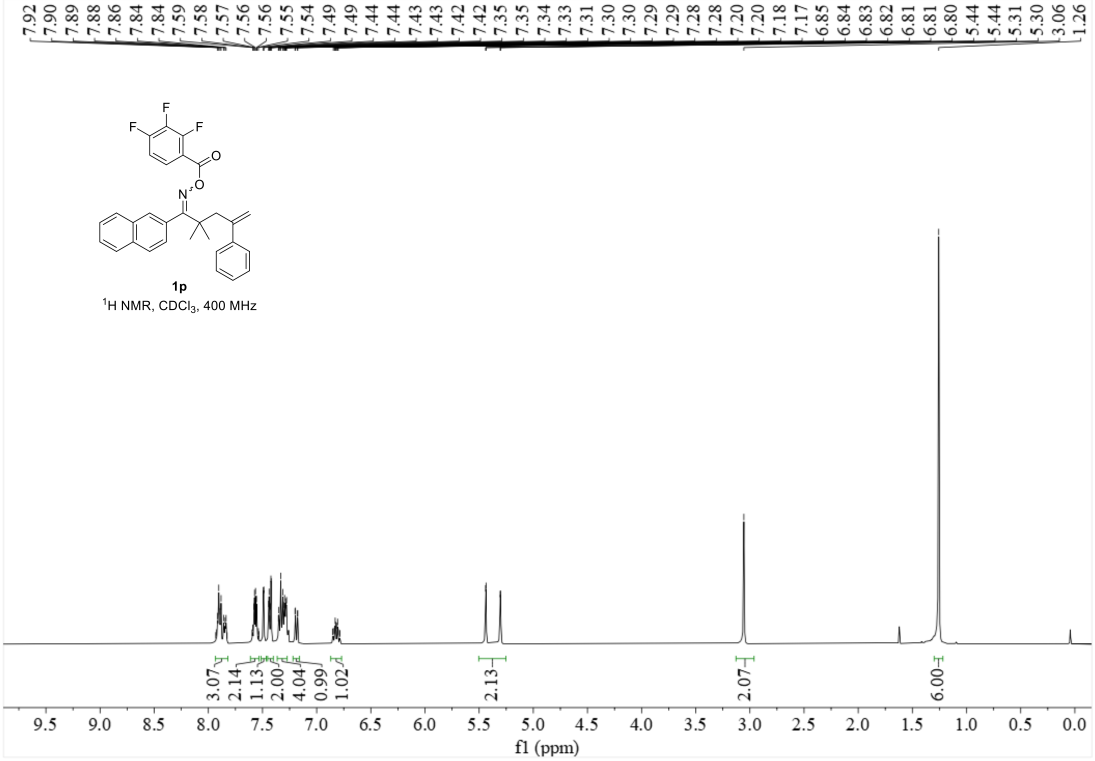
7.92 7.90 7.89 7.88 7.86 7.84 7.84 7.59 7.58 7.57 7.56 7.56 7.55 7.54 7.49 7.44 7.44 7.43 7.43 7.42 7.42 7.35 7.35 7.34 7.33 7.31 7.30 7.30 7.29 7.29 7.28 7.20 7.20 7.18 7.17 6.85 6.84 6.83 6.82 6.81 6.81 6.80 5.44 5.44 5.31 5.30 3.06 1.26
F
F
F
O
N
O
1p
^{1}H NMR, CDCl$_3$, 400 MHz
3.07 2.14 1.13 2.00 4.04 0.99 1.02 2.13 2.07 6.00
9.5 9.0 8.5 8.0 7.5 7.0 6.5 6.0 5.5 5.0 4.5 4.0 3.5 3.0 2.5 2.0 1.5 1.0 0.5 0.0
f1 (ppm)

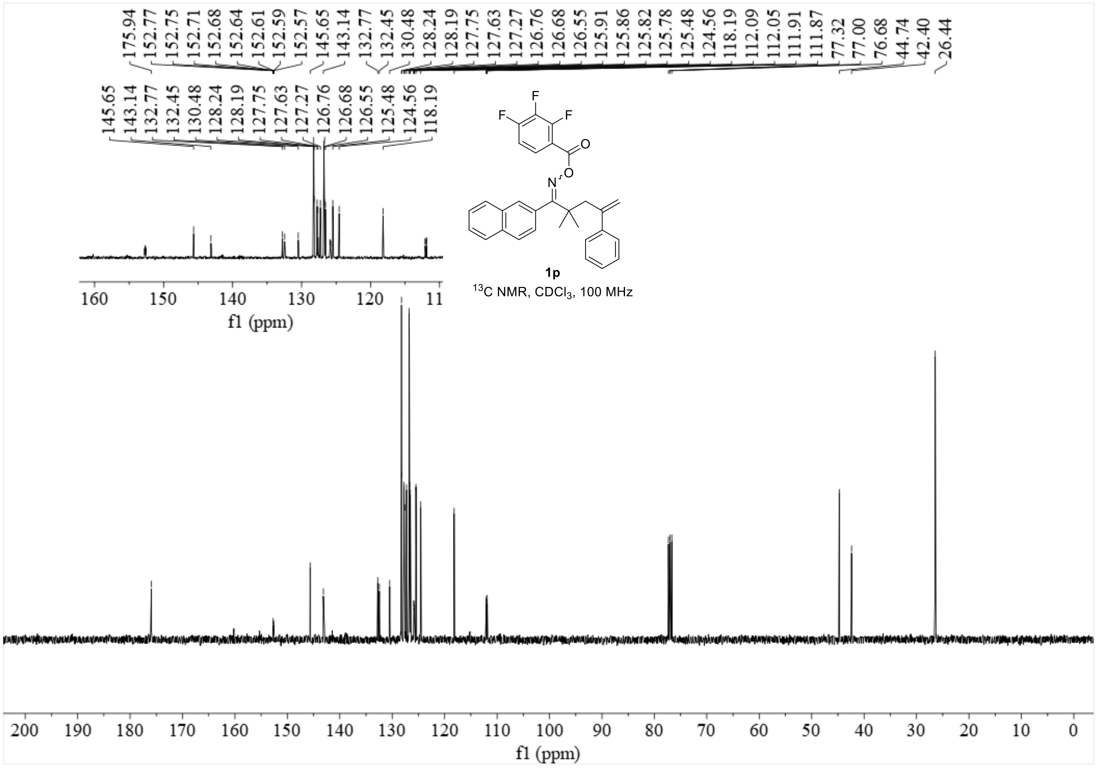
1p
13C NMR, CDCl3, 100 MHz
f1 (ppm)

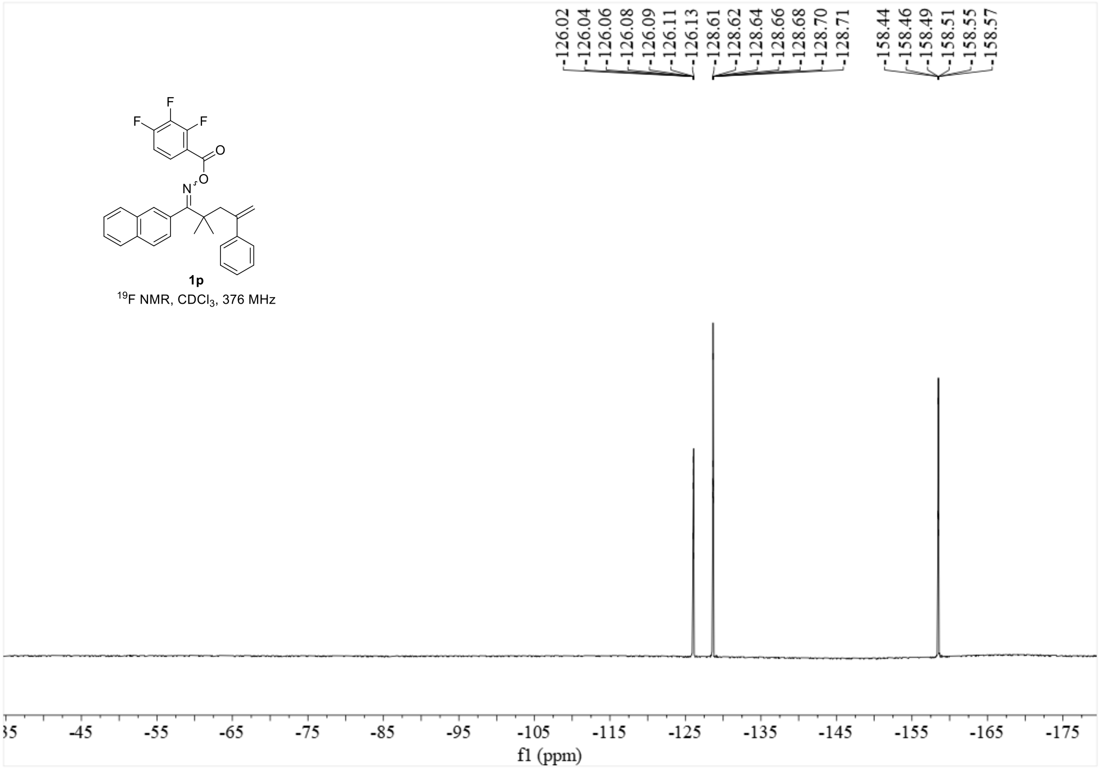
1p
19F NMR, CDCl3, 376 MHz
f1 (ppm)

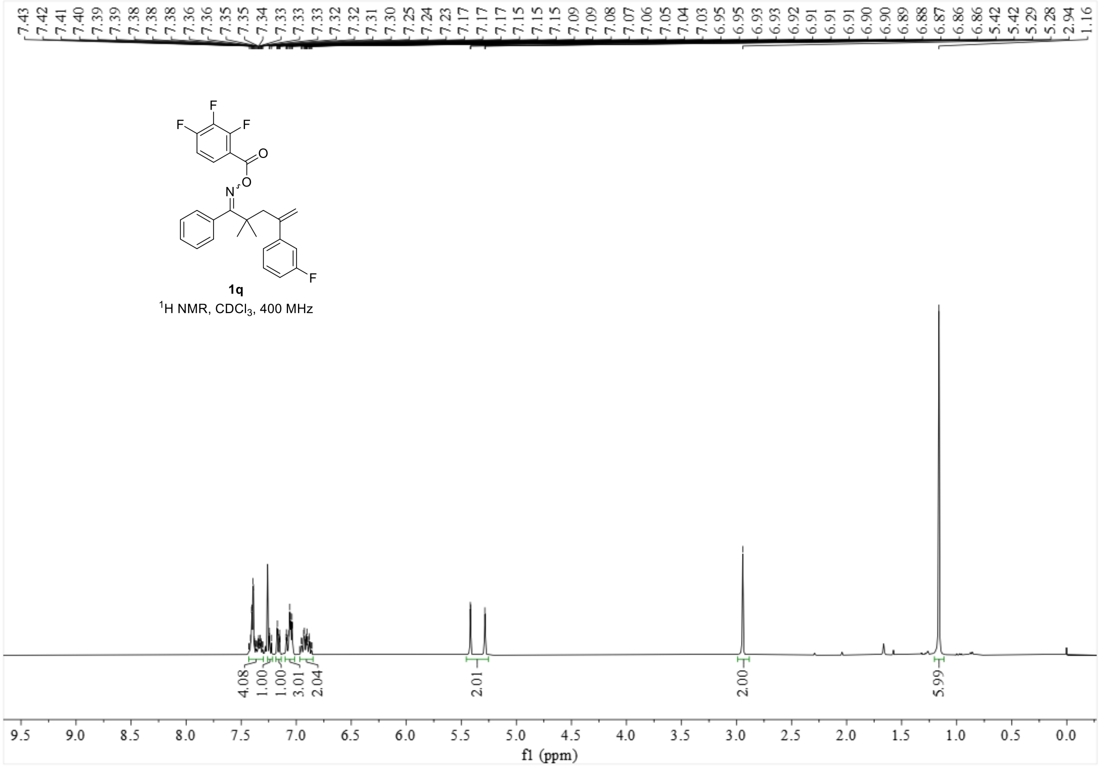
1q
^{1}H NMR, CDCl$_3$, 400 MHz
fl (ppm)

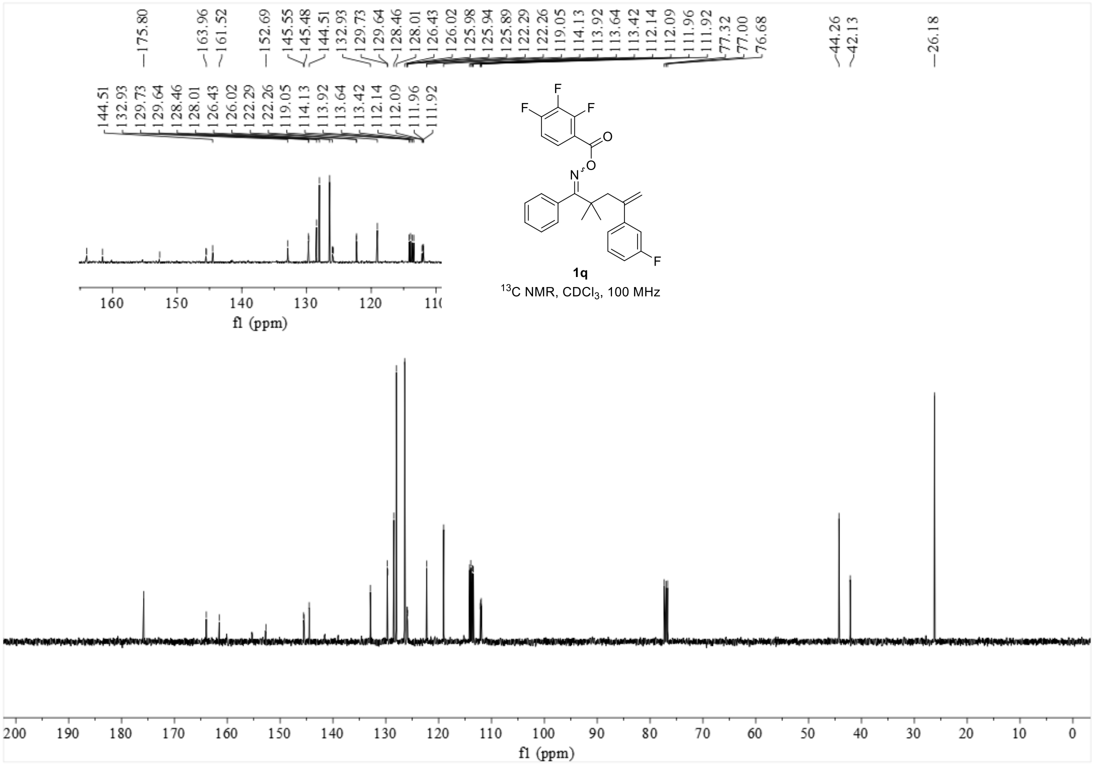
1q
^{13}C NMR, CDCl$_3$, 100 MHz
fl (ppm)

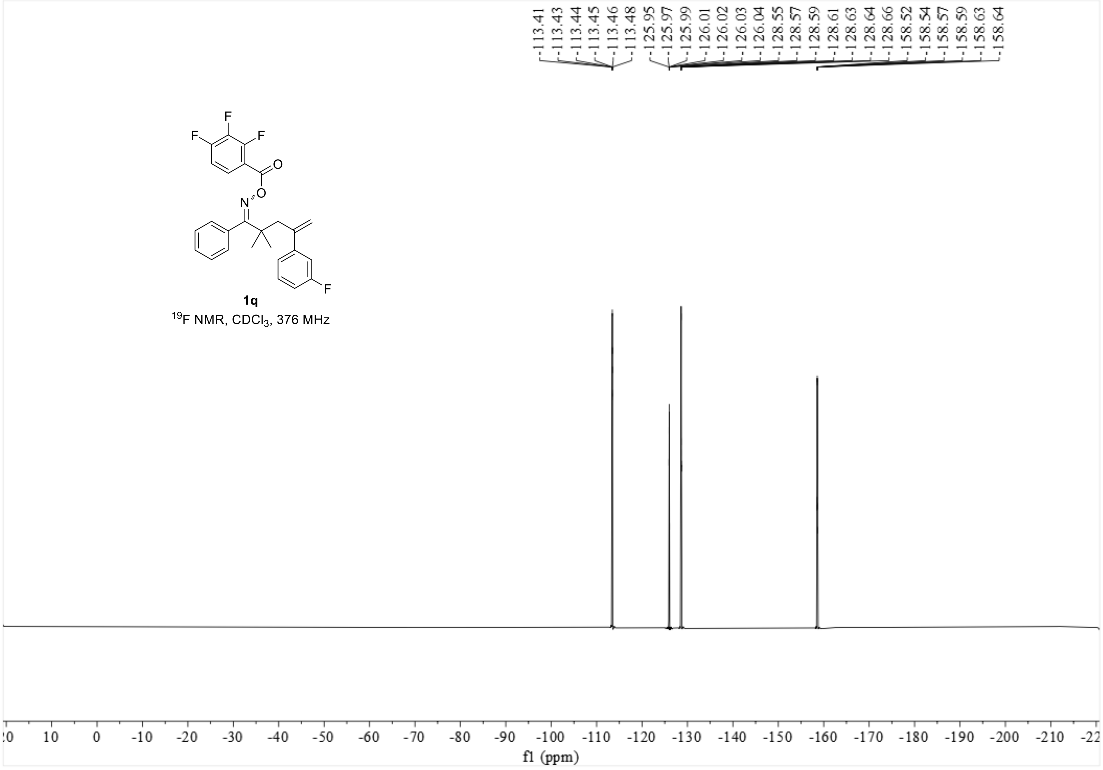
-113.41
-113.43
-113.44
-113.45
-113.46
-113.48
-125.95
-125.97
-125.99
-126.01
-126.02
-126.03
-126.04
-128.55
-128.57
-128.59
-128.61
-128.63
-128.64
-128.66
-158.52
-158.54
-158.57
-158.59
-158.63
-158.64
1q
^{19}F NMR, CDCl$_3$, 376 MHz
f1 (ppm)

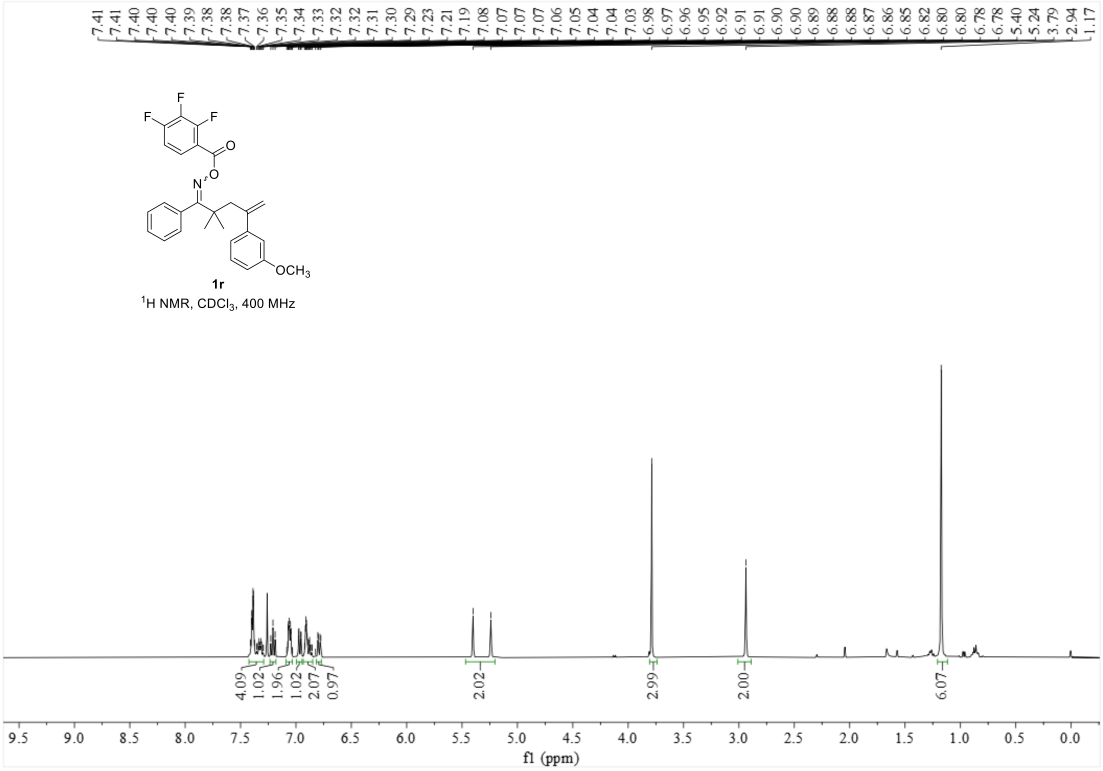
7.41
7.41
7.40
7.40
7.39
7.38
7.38
7.37
7.36
7.35
7.34
7.33
7.32
7.32
7.31
7.30
7.29
7.23
7.21
7.19
7.08
7.07
7.07
7.07
7.06
7.05
7.04
7.04
7.03
6.98
6.97
6.96
6.95
6.92
6.91
6.91
6.90
6.90
6.89
6.88
6.88
6.87
6.86
6.85
6.82
6.80
6.80
6.78
5.40
5.24
3.79
2.94
1.17
OCH$_3$
1r
^{1}H NMR, CDCl$_3$, 400 MHz
4.09
1.02
1.96
1.02
2.07
0.97
2.02
2.99
2.00
6.07
f1 (ppm)

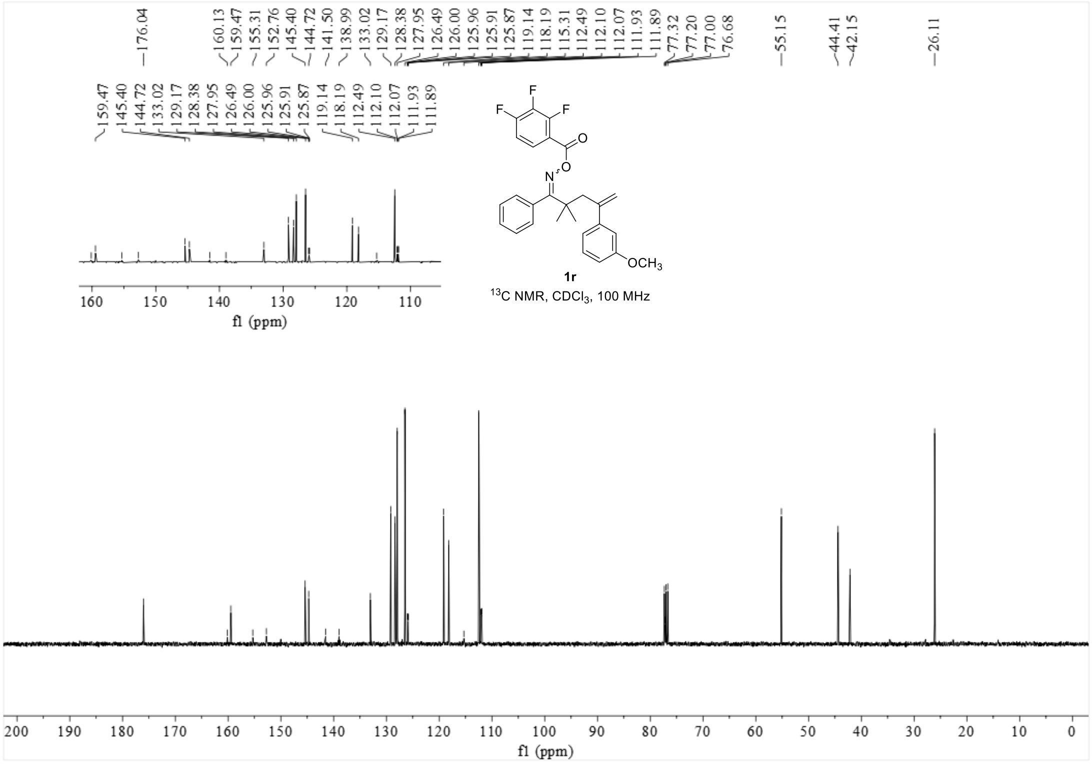
176.04
160.13
159.47
155.31
152.76
145.40
144.72
141.50
138.99
133.02
129.17
128.38
127.95
126.49
126.00
125.96
125.91
125.87
119.14
118.19
115.31
112.49
112.10
112.07
111.93
111.89
77.32
77.20
77.00
76.68
55.15
44.41
42.15
26.11
1r
^{13}C NMR, CDCl$_3$, 100 MHz
f1 (ppm)

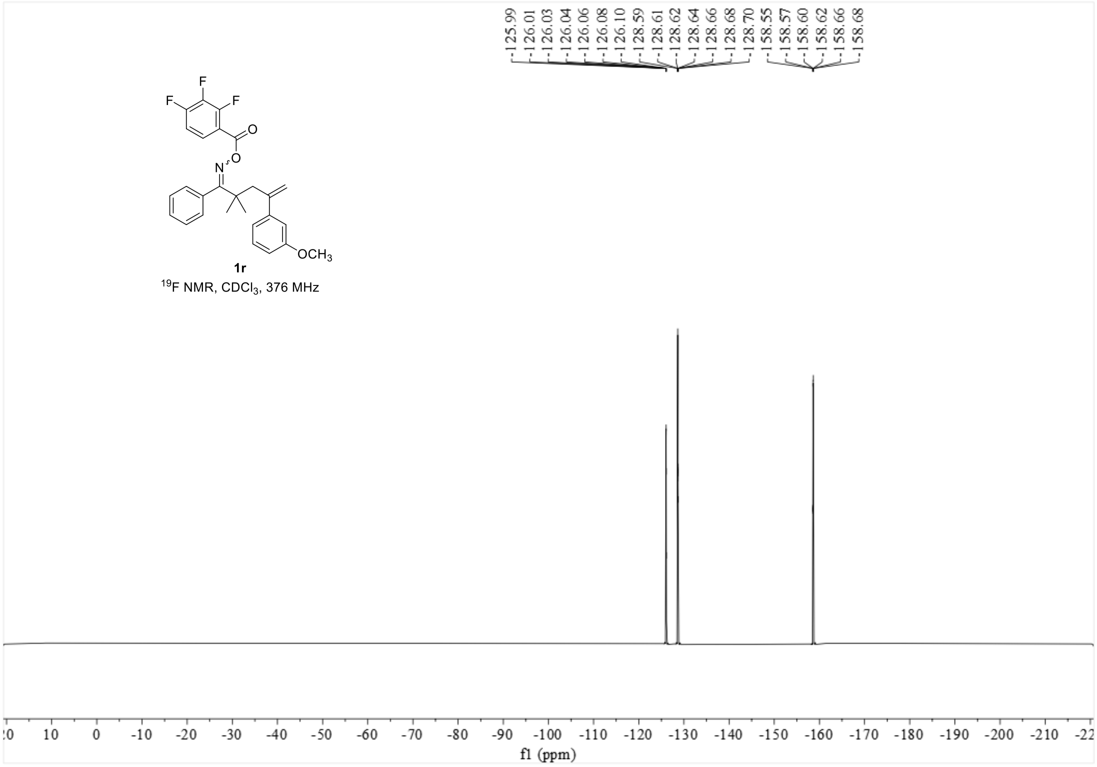
-125.99
-126.01
-126.03
-126.04
-126.06
-126.08
-126.10
-128.59
-128.61
-128.62
-128.64
-128.66
-128.68
-128.70
-158.55
-158.57
-158.60
-158.62
-158.66
-158.68
1r
^{19}F NMR, CDCl$_3$, 376 MHz
f1 (ppm)

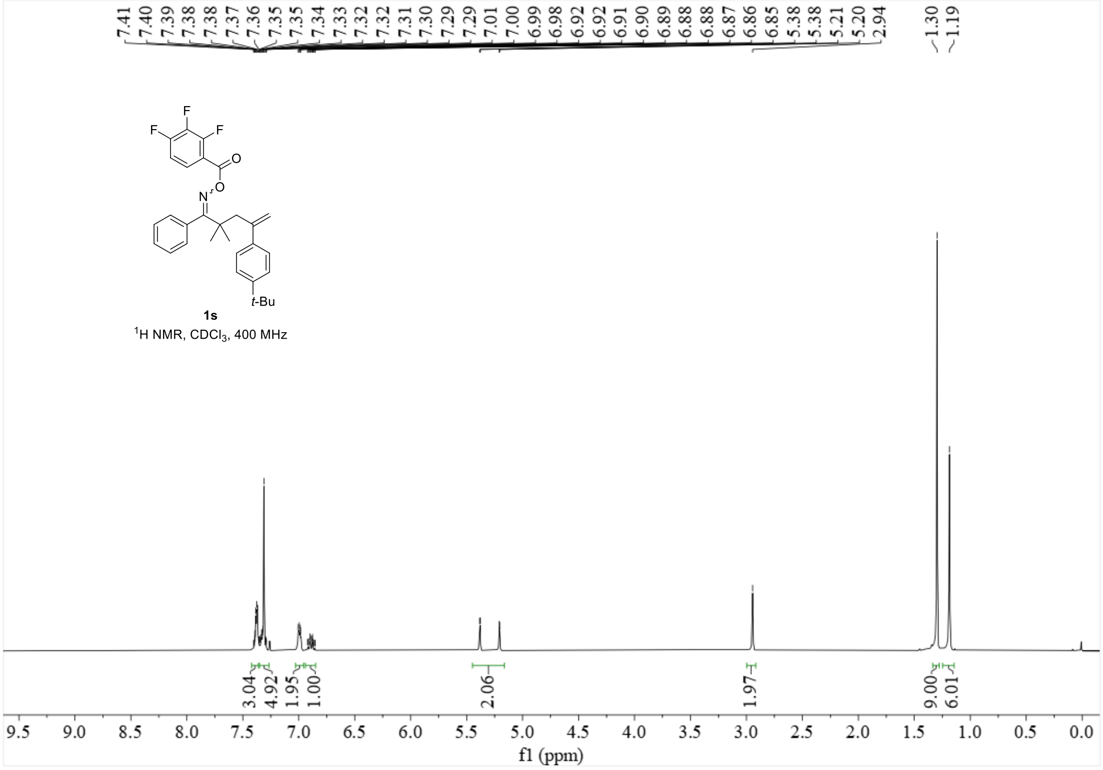
7.41 7.40 7.39 7.38 7.38 7.37 7.36 7.35 7.35 7.34 7.33 7.32 7.32 7.31 7.30 7.29 7.29 7.01 7.00 6.99 6.98 6.92 6.92 6.91 6.90 6.89 6.88 6.88 6.87 6.86 6.85 5.38 5.38 5.21 5.20 2.94
1.30 1.19
F
F
F
O
N
O
t-Bu
1s
1H NMR, CDCl3, 400 MHz
3.04 4.92 1.95 1.00
2.06
1.97
9.00 6.01
9.5 9.0 8.5 8.0 7.5 7.0 6.5 6.0 5.5 5.0 4.5 4.0 3.5 3.0 2.5 2.0 1.5 1.0 0.5 0.0
f1 (ppm)

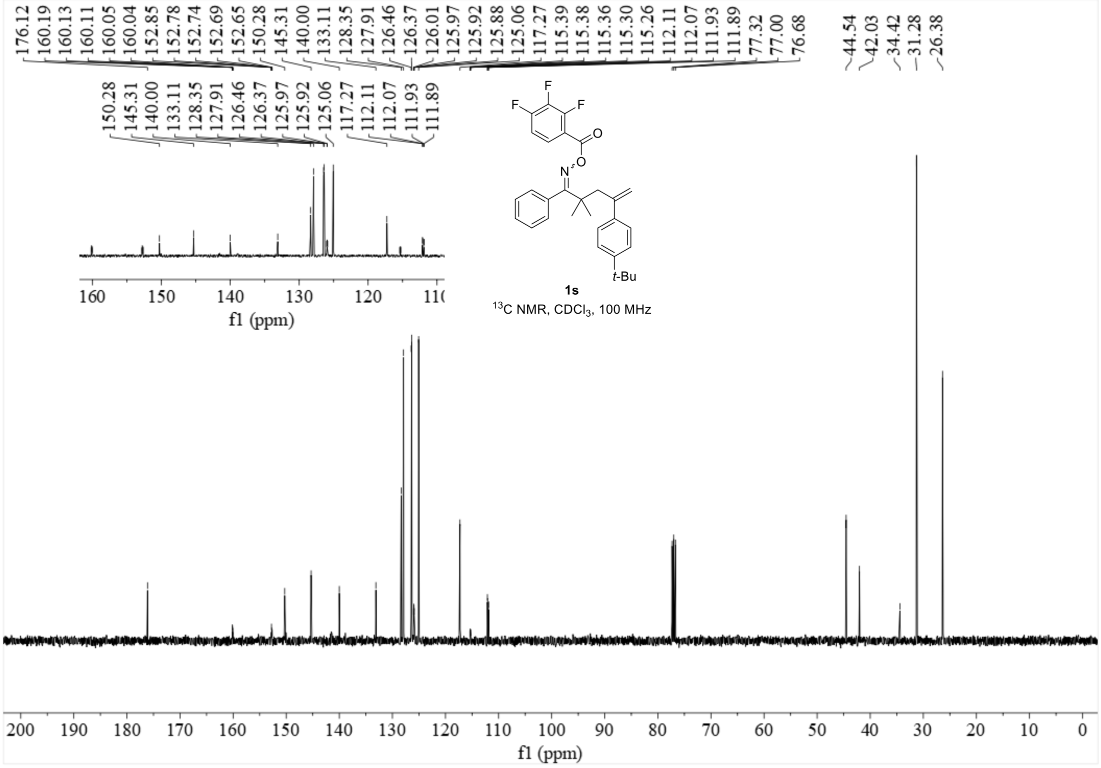
176.12 160.19 160.13 160.11 160.05 160.04 152.85 152.78 152.74 152.69 152.65 150.28 145.31 140.00 133.11 128.35 127.91 126.46 126.37 126.01 125.97 125.92 125.88 125.06 117.27 115.39 115.38 115.36 115.30 115.26 112.11 112.07 111.93 111.89 77.32 77.00 76.68
44.54 42.03 34.42 31.28 26.38
150.28 145.31 140.00 133.11 128.35 127.91 126.46 126.37 125.97 125.92 125.06 117.27 112.11 112.07 111.93 111.89
160 150 140 130 120 110
f1 (ppm)
F
F
F
O
N
O
t-Bu
1s
13C NMR, CDCl3, 100 MHz
200 190 180 170 160 150 140 130 120 110 100 90 80 70 60 50 40 30 20 10 0
f1 (ppm)

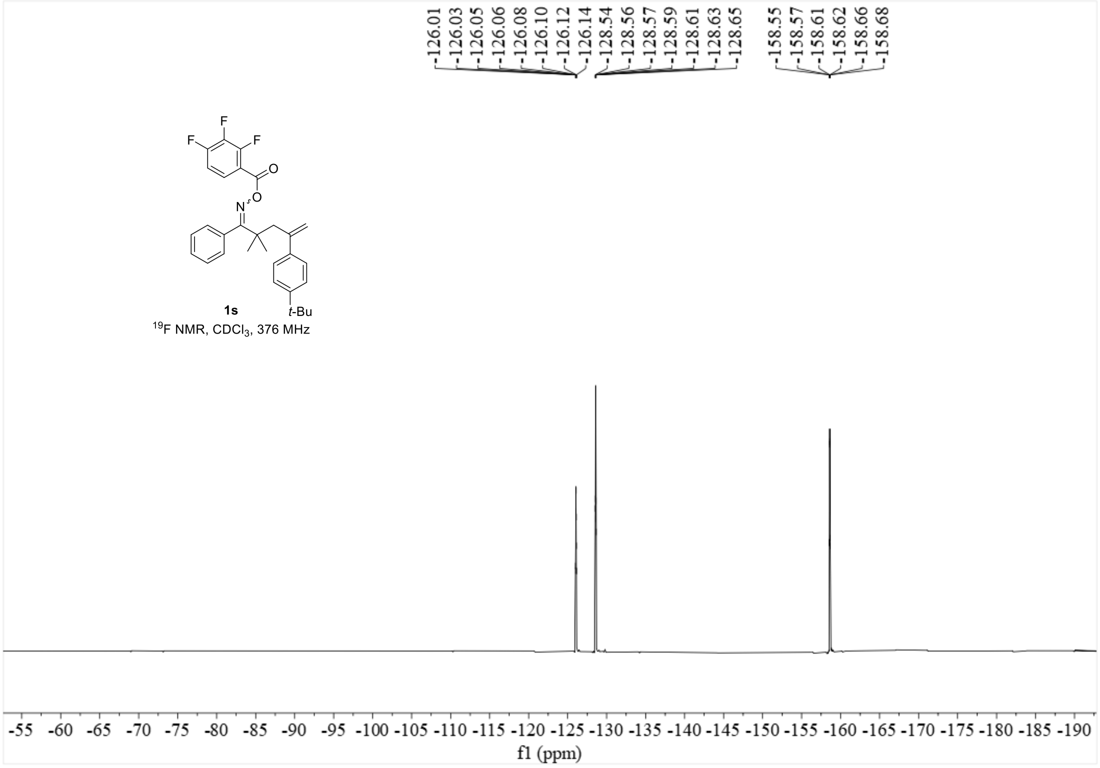
-126.01
-126.03
-126.05
-126.06
-126.08
-126.10
-126.12
-126.14
-128.54
-128.56
-128.57
-128.59
-128.61
-128.63
-128.65
-158.55
-158.57
-158.61
-158.62
-158.66
-158.68
F
F
F
O
N
O
t-Bu
1s
19F NMR, CDCl3, 376 MHz
-55 -60 -65 -70 -75 -80 -85 -90 -95 -100 -105 -110 -115 -120 -125 -130 -135 -140 -145 -150 -155 -160 -165 -170 -175 -180 -185 -190
f1 (ppm)

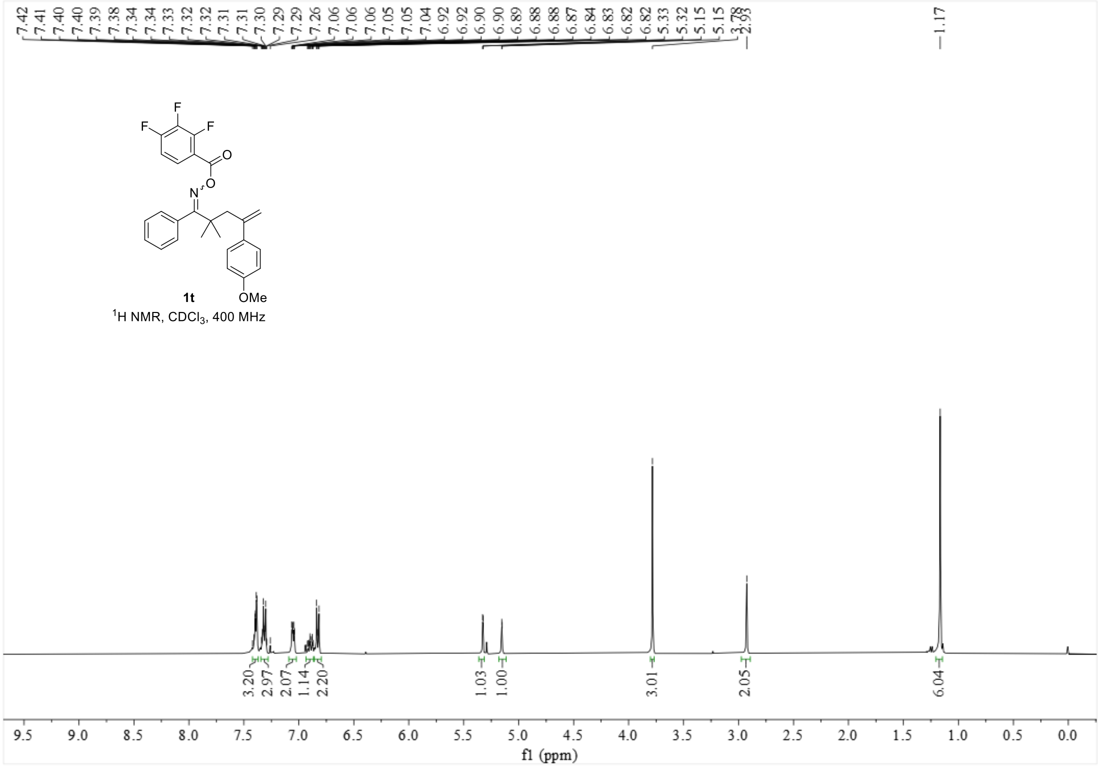
7.42
7.41
7.40
7.39
7.38
7.34
7.33
7.32
7.32
7.31
7.30
7.29
7.29
7.26
7.06
7.06
7.06
7.05
7.04
6.92
6.90
6.90
6.89
6.88
6.87
6.84
6.83
6.82
6.82
5.33
5.32
5.15
5.15
3.78
2.93
1.17
F
F
F
O
N
O
OMe
1t
1H NMR, CDCl3, 400 MHz
3.20
2.97
2.07
1.14
2.20
1.03
1.00
3.01
2.05
6.04
9.5 9.0 8.5 8.0 7.5 7.0 6.5 6.0 5.5 5.0 4.5 4.0 3.5 3.0 2.5 2.0 1.5 1.0 0.5 0.0
f1 (ppm)

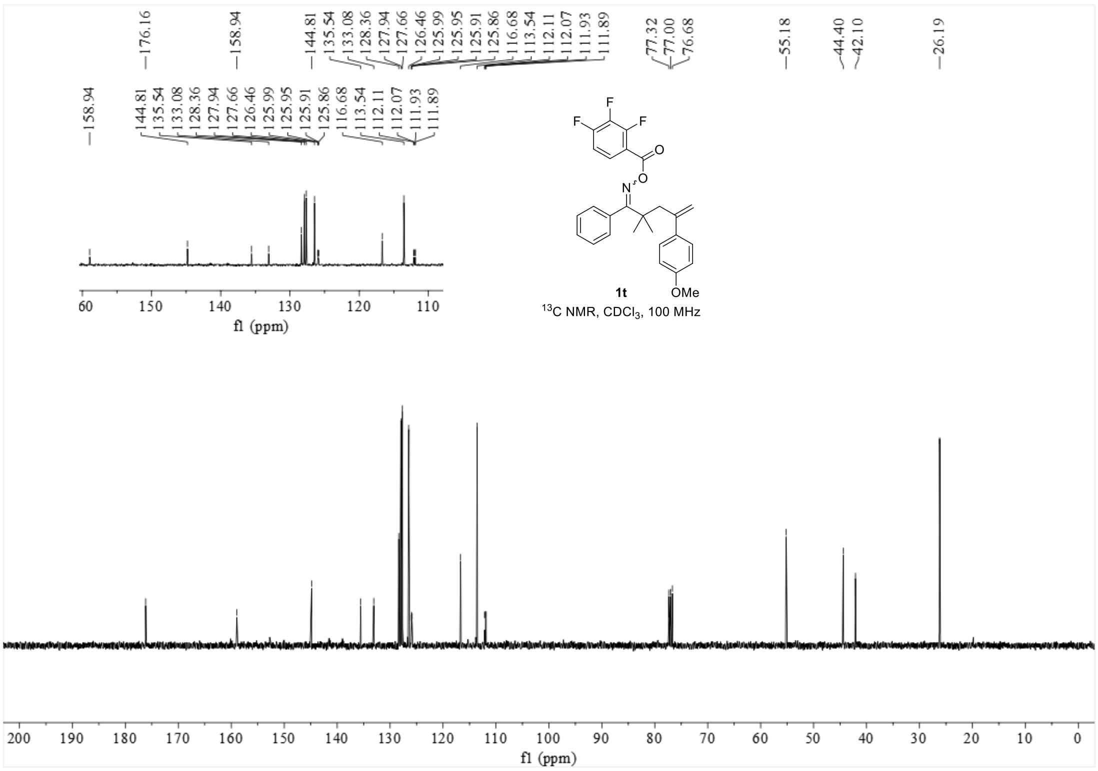
176.16
158.94
144.81
135.54
133.08
128.36
127.94
127.66
126.46
125.99
125.95
125.91
125.86
116.68
113.54
112.11
112.07
111.93
111.89
77.32
77.00
76.68
55.18
44.40
42.10
26.19
1t
OMe
^{13}C NMR, $CDCl_3$, 100 MHz
fl (ppm)

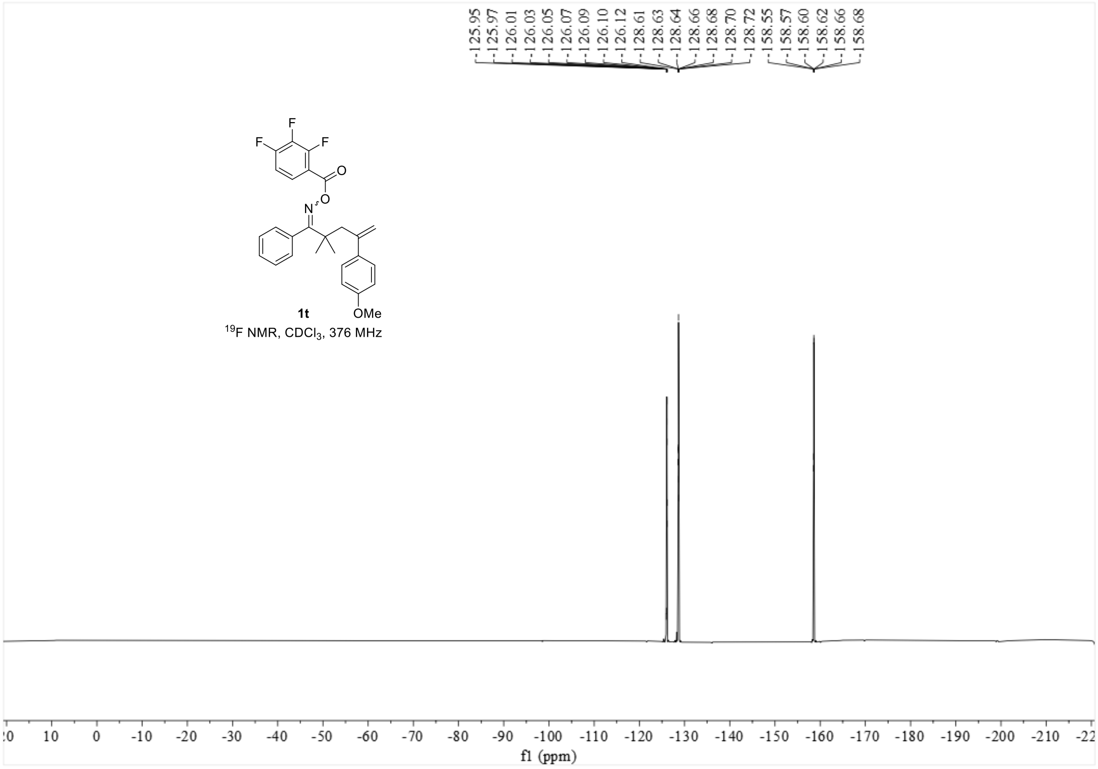
-125.95
-125.97
-126.01
-126.03
-126.05
-126.07
-126.09
-126.10
-126.12
-128.61
-128.63
-128.64
-128.66
-128.68
-128.70
-128.72
-158.55
-158.57
-158.60
-158.62
-158.66
-158.68
1t
OMe
^{19}F NMR, $CDCl_3$, 376 MHz
fl (ppm)

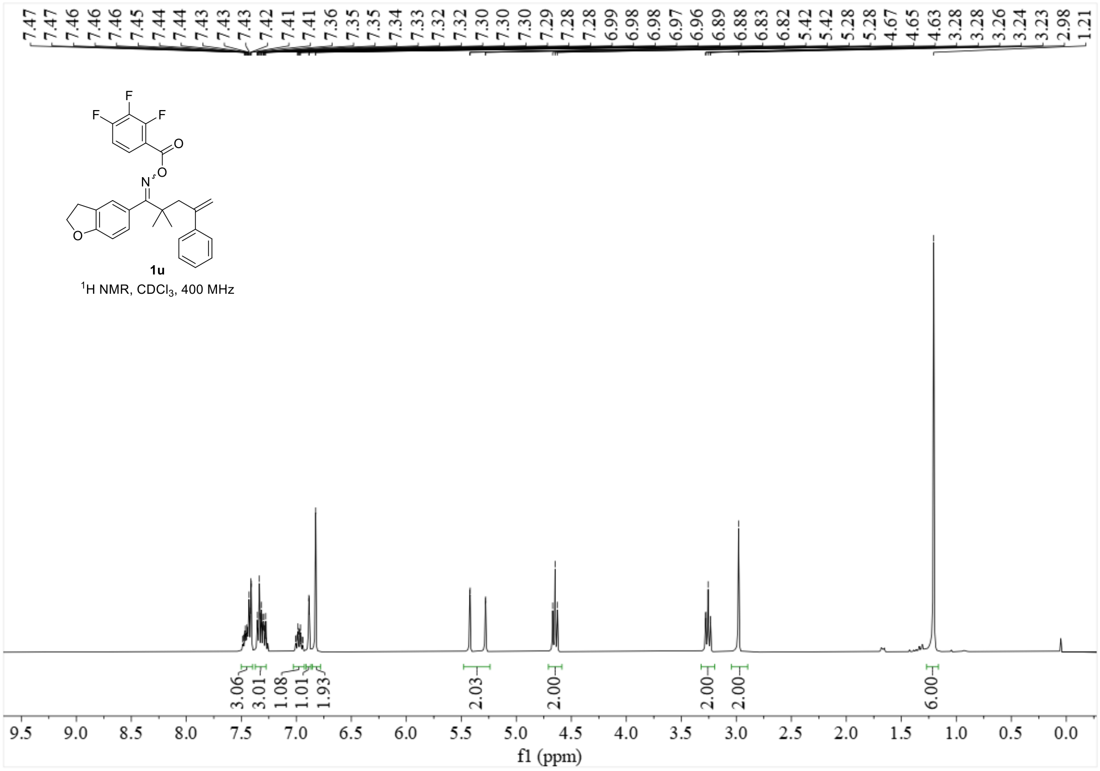
1u
¹H NMR, CDCl₃, 400 MHz
f1 (ppm)

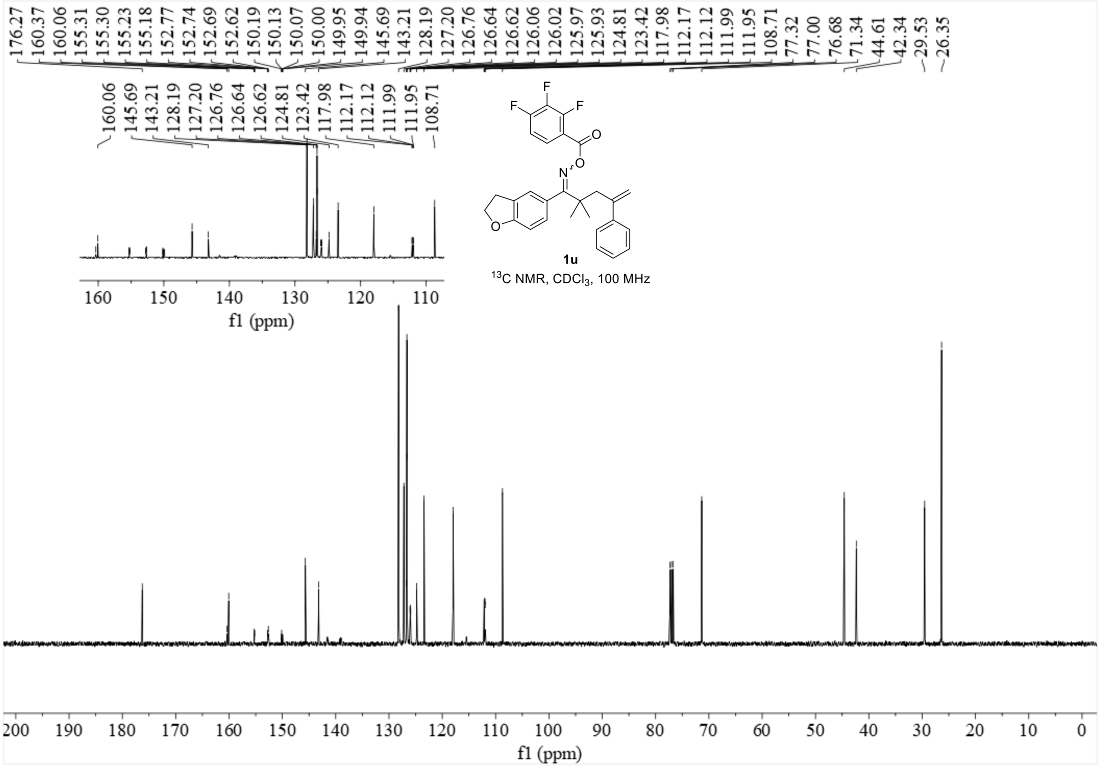
1u
¹³C NMR, CDCl₃, 100 MHz
f1 (ppm)

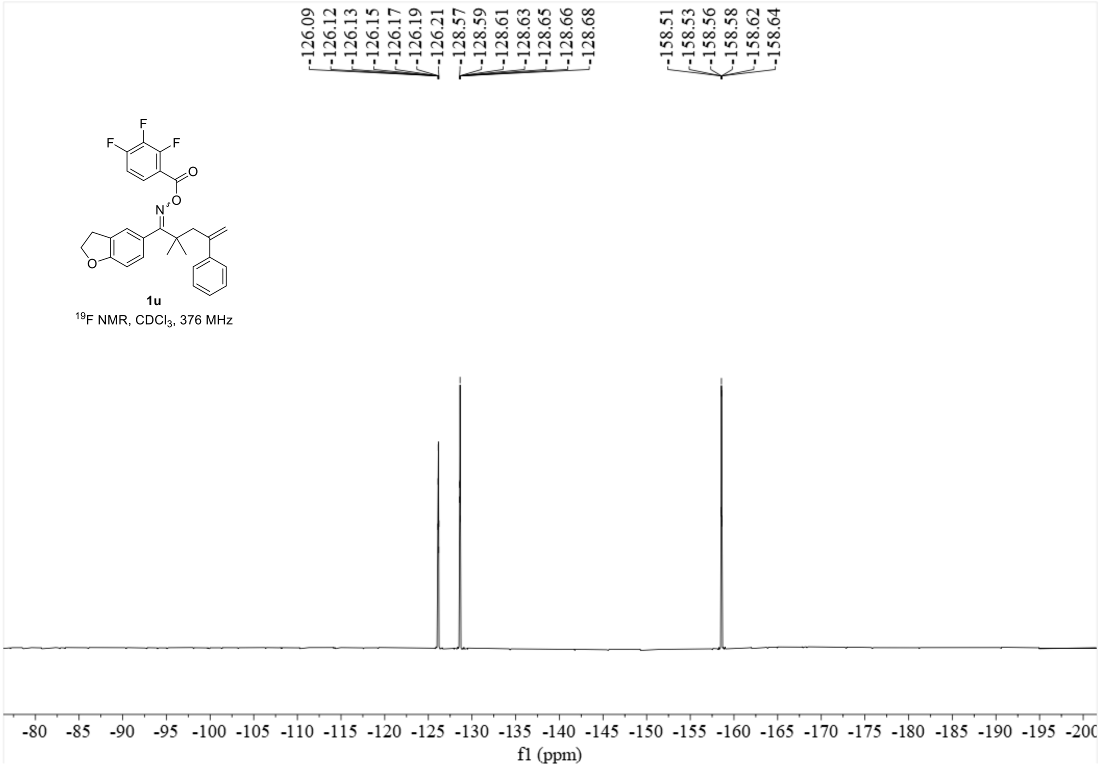

1u
^{19}F NMR, CDCl$_3$, 376 MHz

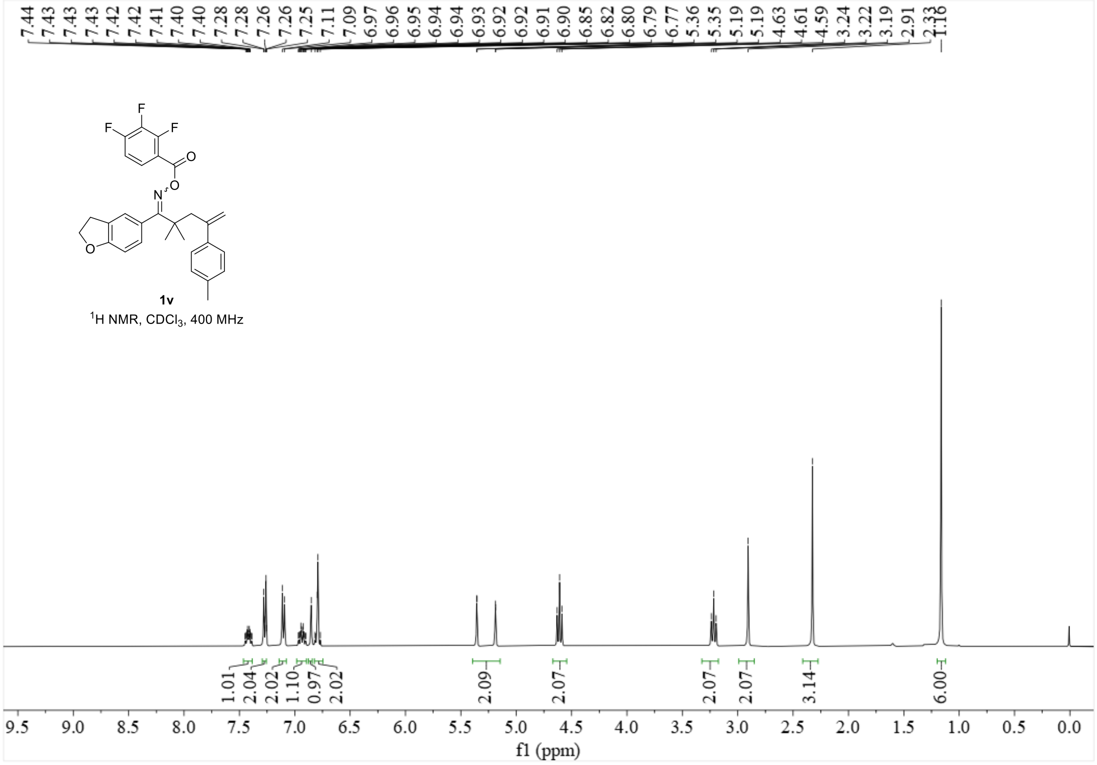

1v
^{1}H NMR, CDCl$_3$, 400 MHz

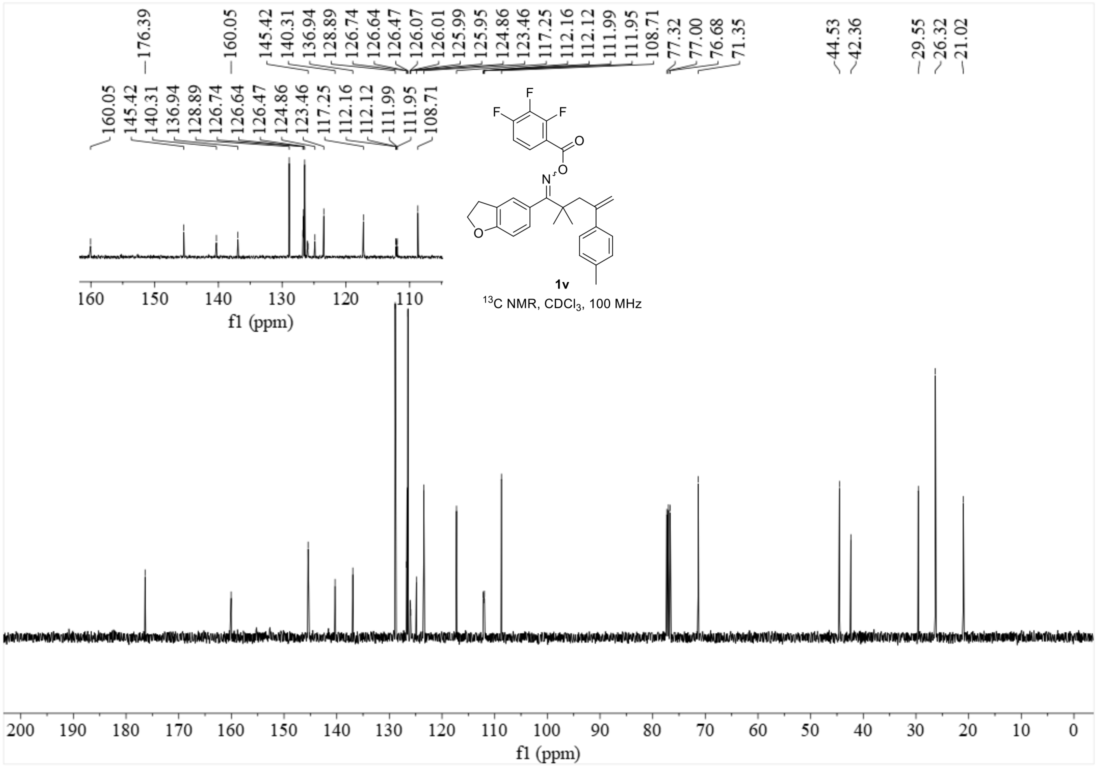
176.39
160.05
145.42
140.31
136.94
128.89
126.74
126.64
126.47
126.07
126.01
125.99
125.95
124.86
123.46
117.25
112.16
112.12
111.99
111.95
108.71
77.32
77.00
76.68
71.35
44.53
42.36
29.55
26.32
21.02
1v
^{13}C NMR, CDCl$_3$, 100 MHz
f1 (ppm)

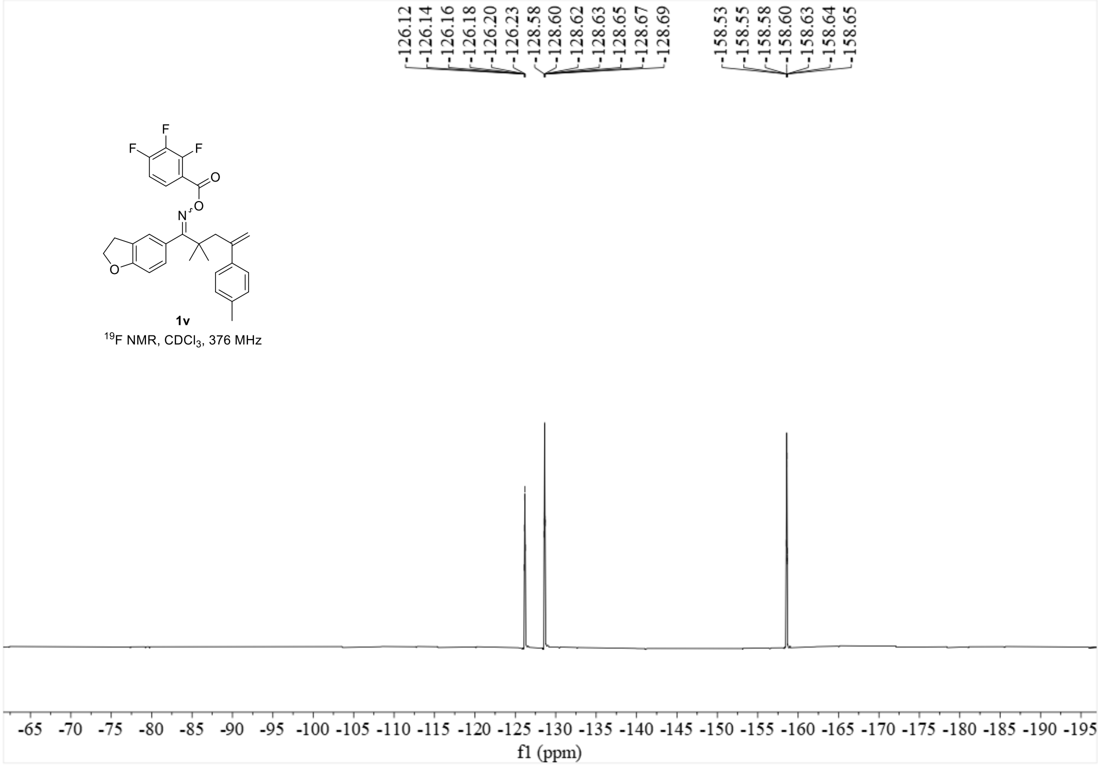
-126.12
-126.14
-126.16
-126.18
-126.20
-126.23
-128.58
-128.60
-128.62
-128.63
-128.65
-128.67
-128.69
-158.53
-158.55
-158.58
-158.60
-158.63
-158.64
-158.65
1v
^{19}F NMR, CDCl$_3$, 376 MHz
f1 (ppm)

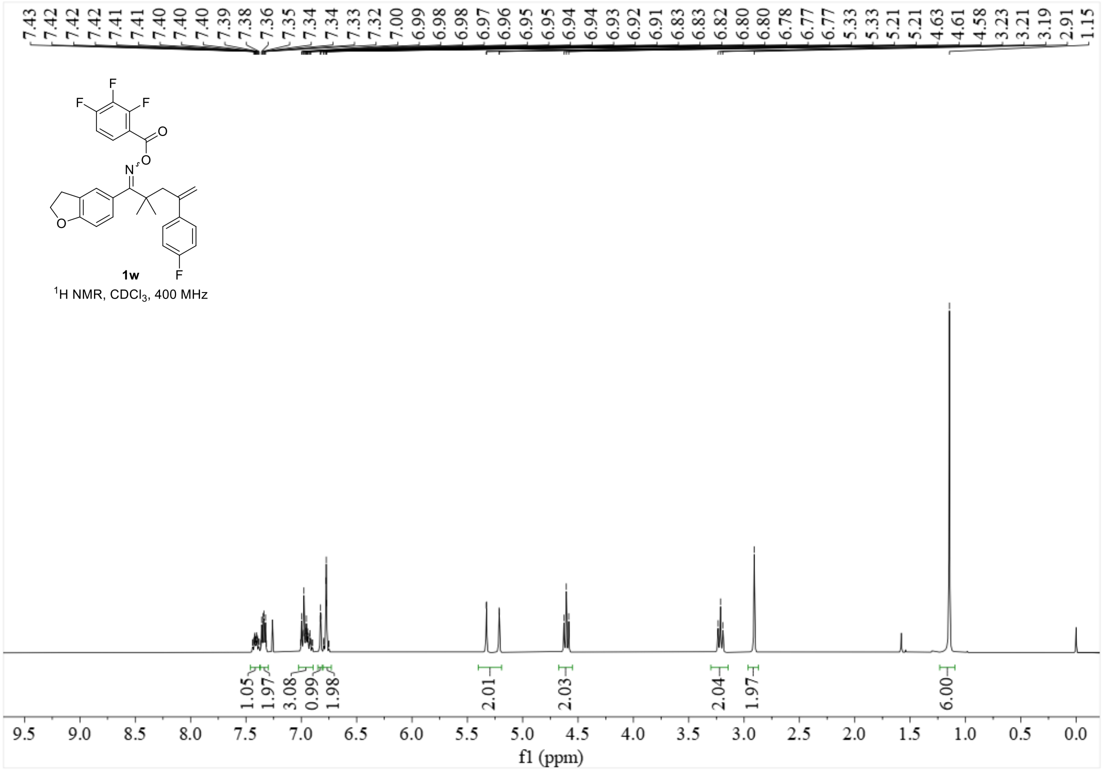
7.43 7.42 7.42 7.41 7.41 7.40 7.40 7.39 7.38 7.36 7.35 7.34 7.34 7.33 7.32 7.00 6.99 6.98 6.98 6.97 6.96 6.95 6.95 6.94 6.94 6.93 6.92 6.91 6.83 6.83 6.82 6.80 6.80 6.78 6.77 6.77 5.33 5.33 5.21 5.21 4.63 4.61 4.58 3.23 3.21 3.19 2.91 1.15
F
F
F
O
N
O
O
F
1w
^{1}H NMR, CDCl$_3$, 400 MHz
1.05 1.97 3.08 0.99 1.98 2.01 2.03 2.04 1.97 6.00
9.5 9.0 8.5 8.0 7.5 7.0 6.5 6.0 5.5 5.0 4.5 4.0 3.5 3.0 2.5 2.0 1.5 1.0 0.5 0.0
f1 (ppm)

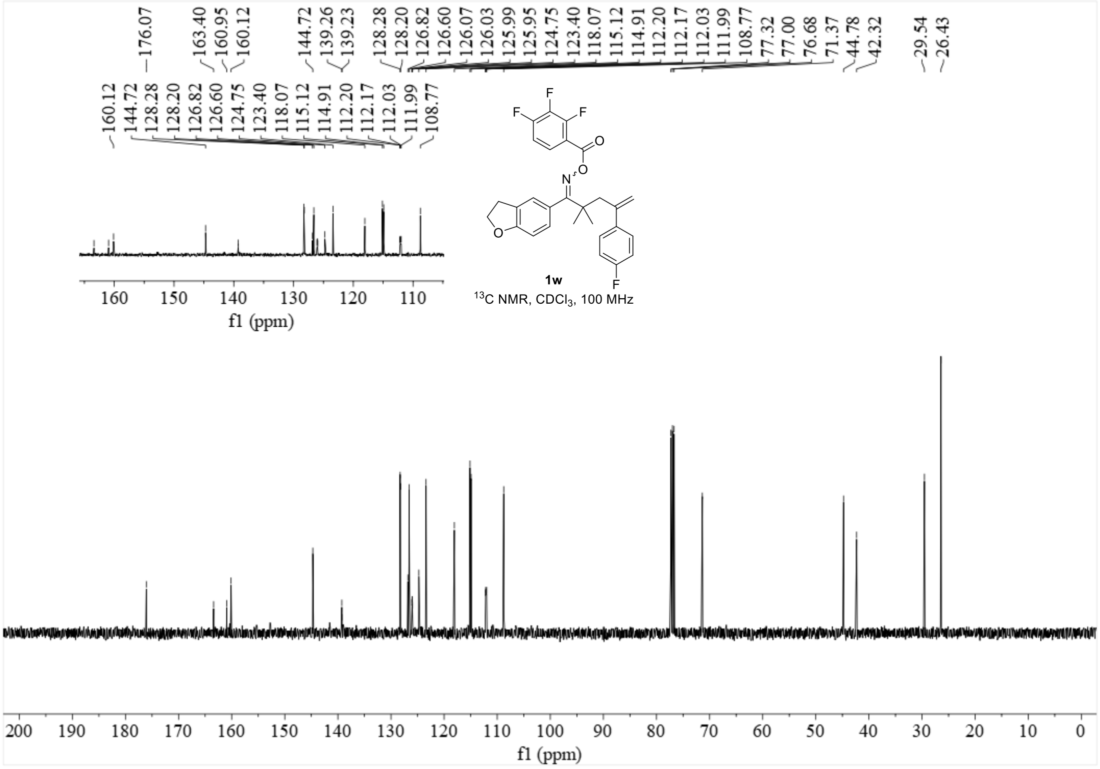
176.07 163.40 160.95 160.12 144.72 139.26 139.23 128.28 128.20 126.82 126.60 126.07 126.03 125.99 125.95 124.75 123.40 118.07 115.12 114.91 112.20 112.17 112.03 111.99 108.77 77.32 77.00 76.68 71.37 44.78 42.32 29.54 26.43
160.12 144.72 128.28 128.20 126.82 126.60 124.75 123.40 118.07 115.12 114.91 112.20 112.17 112.03 111.99 108.77
160 150 140 130 120 110
f1 (ppm)
F
F
F
O
N
O
O
F
1w
^{13}C NMR, CDCl$_3$, 100 MHz
200 190 180 170 160 150 140 130 120 110 100 90 80 70 60 50 40 30 20 10 0
f1 (ppm)

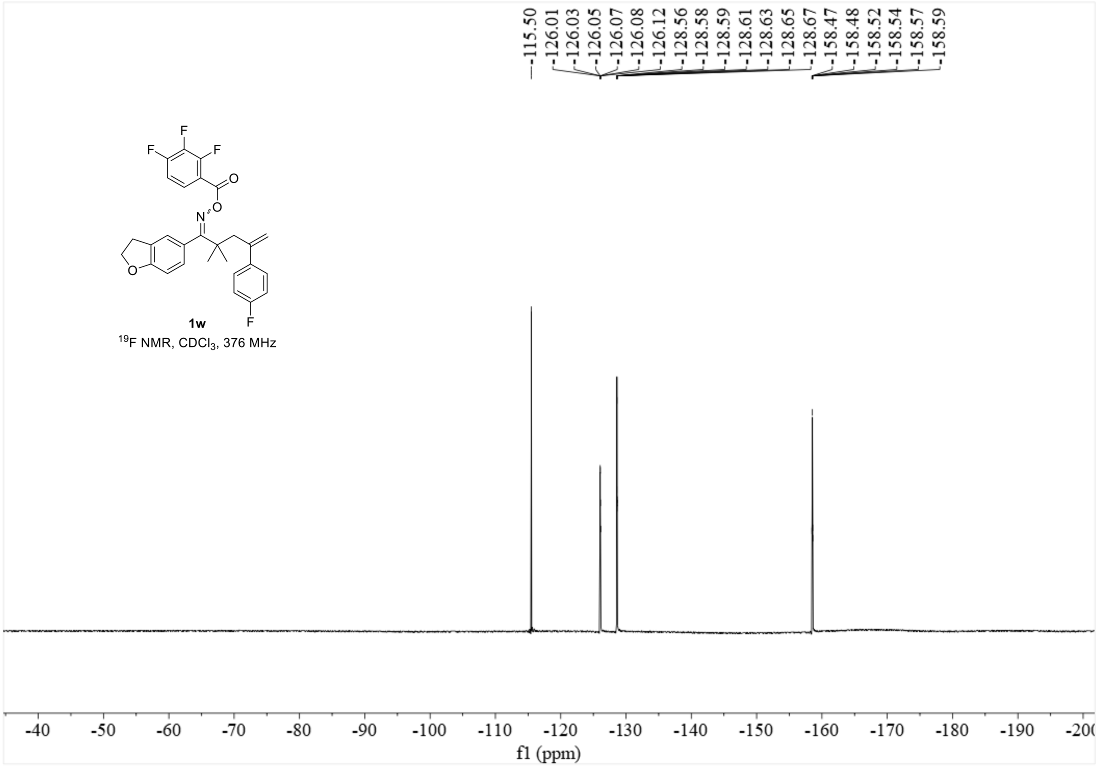
1w
19F NMR, CDCl3, 376 MHz
-115.50
-126.01
-126.03
-126.05
-126.07
-126.08
-126.12
-128.56
-128.58
-128.59
-128.61
-128.63
-128.65
-128.67
-158.47
-158.48
-158.52
-158.54
-158.57
-158.59
f1 (ppm)

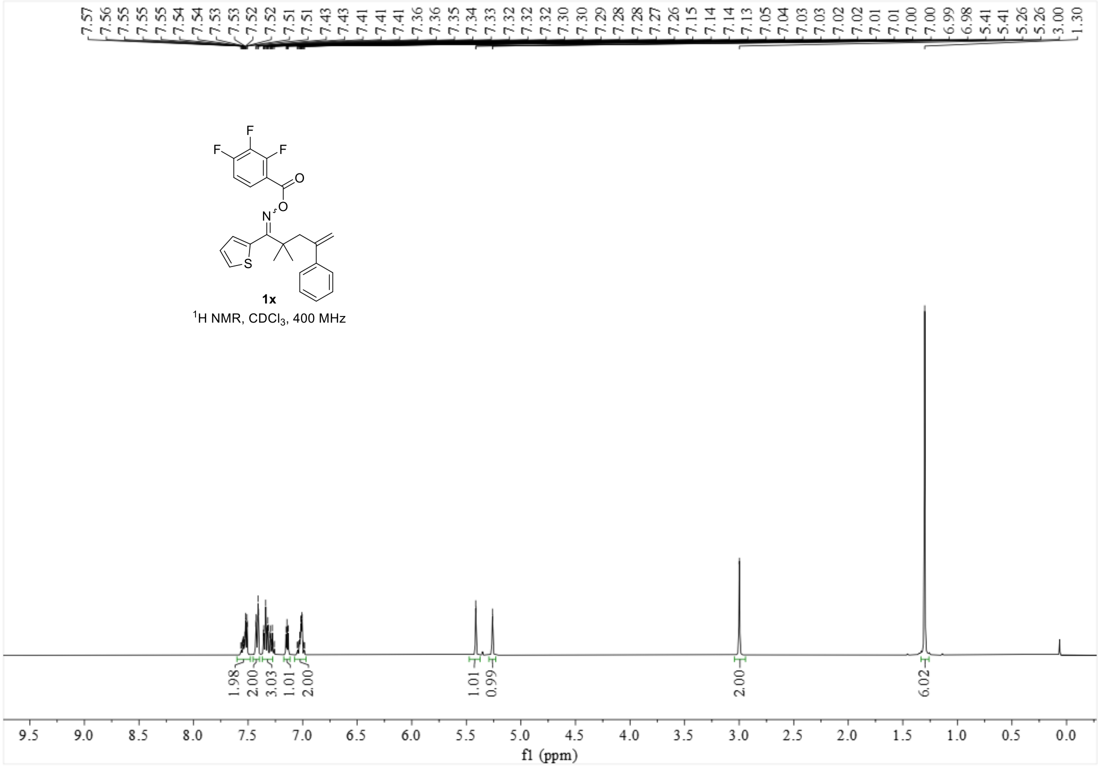
1x
1H NMR, CDCl3, 400 MHz
1.98
2.00
3.03
1.01
2.00
1.01
0.99
2.00
6.02
f1 (ppm)

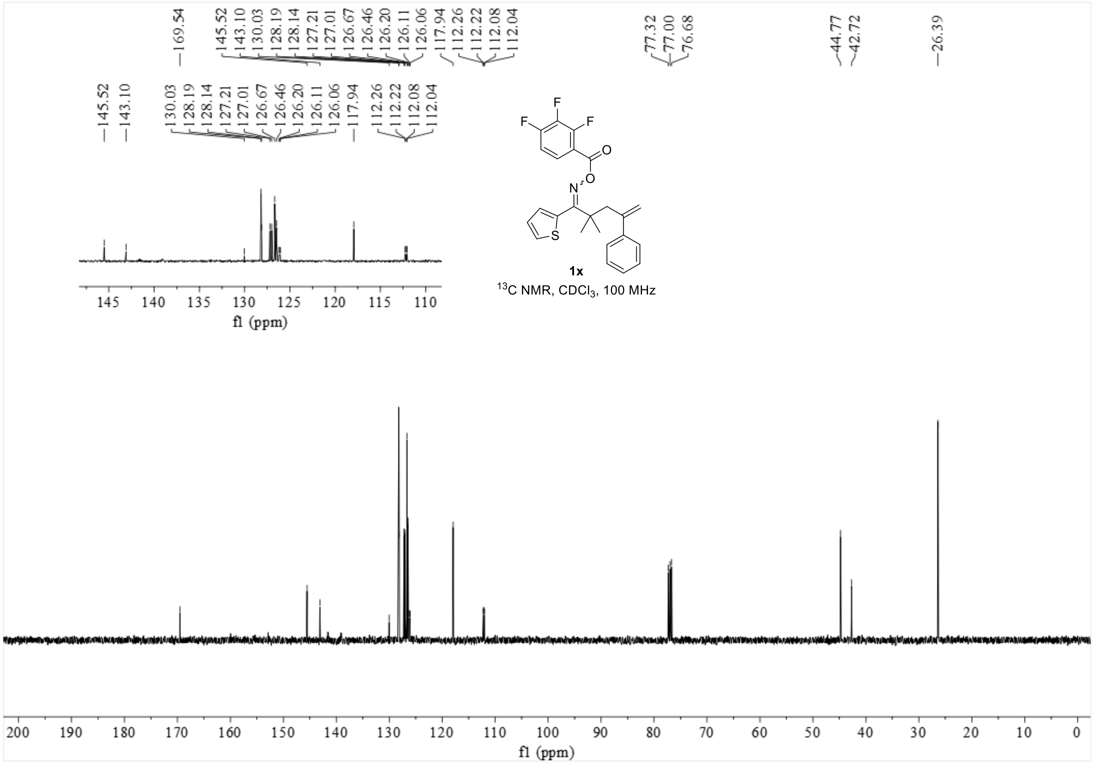
169.54
145.52
143.10
130.03
128.19
128.14
127.21
127.01
126.67
126.46
126.20
126.11
126.06
117.94
112.26
112.22
112.08
112.04
77.32
77.00
76.68
44.77
42.72
26.39
1x
^{13}C NMR, CDCl$_3$, 100 MHz
f1 (ppm)

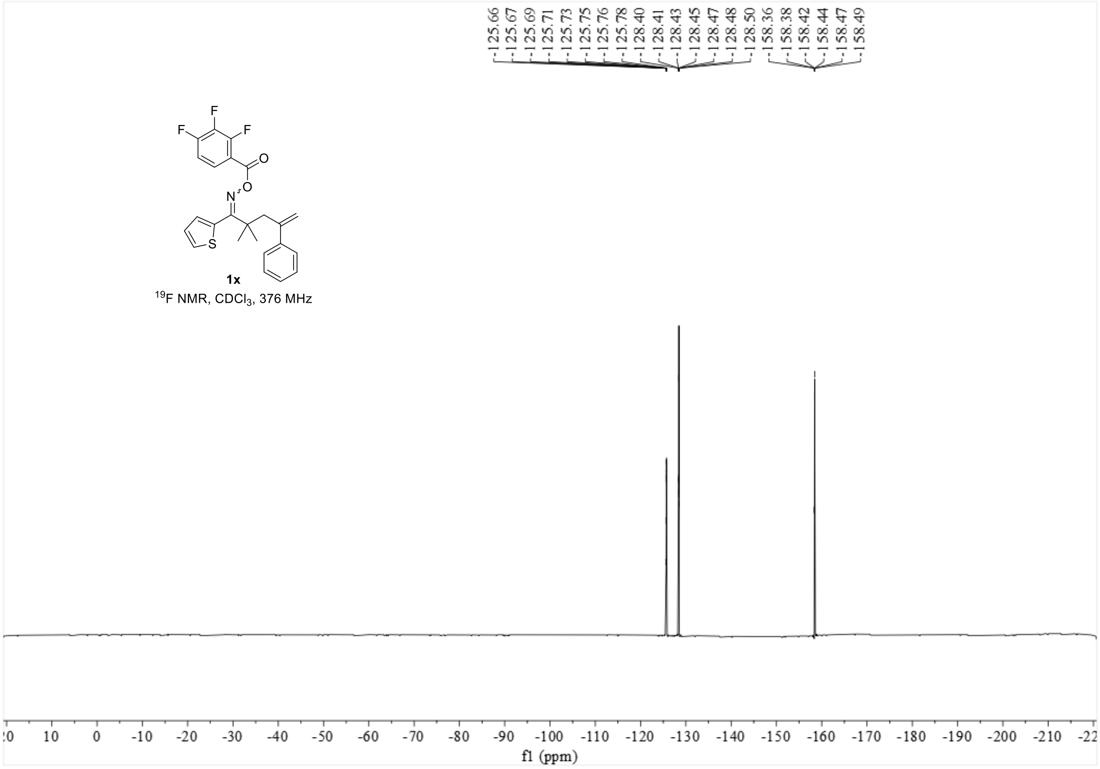
-125.66
-125.67
-125.69
-125.71
-125.73
-125.75
-125.76
-125.78
-128.40
-128.41
-128.43
-128.45
-128.47
-128.48
-128.50
-158.36
-158.38
-158.42
-158.44
-158.47
-158.49
1x
^{19}F NMR, CDCl$_3$, 376 MHz
f1 (ppm)

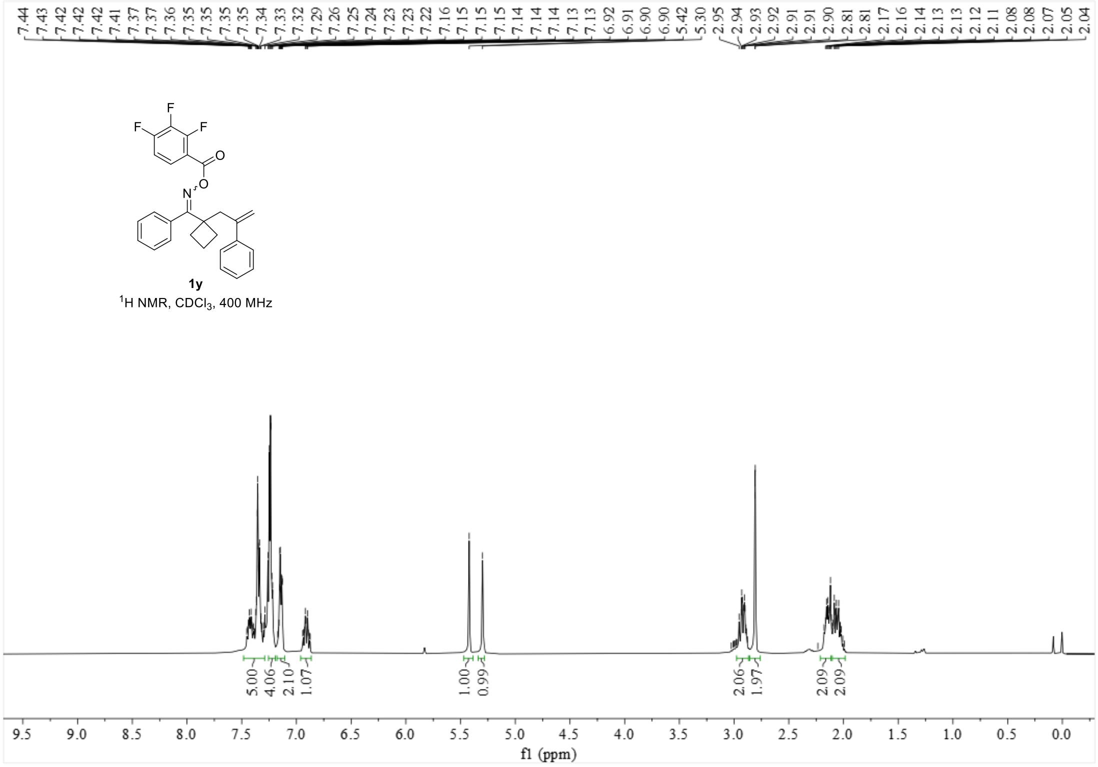
1y
^{1}H NMR, CDCl$_3$, 400 MHz
f1 (ppm)

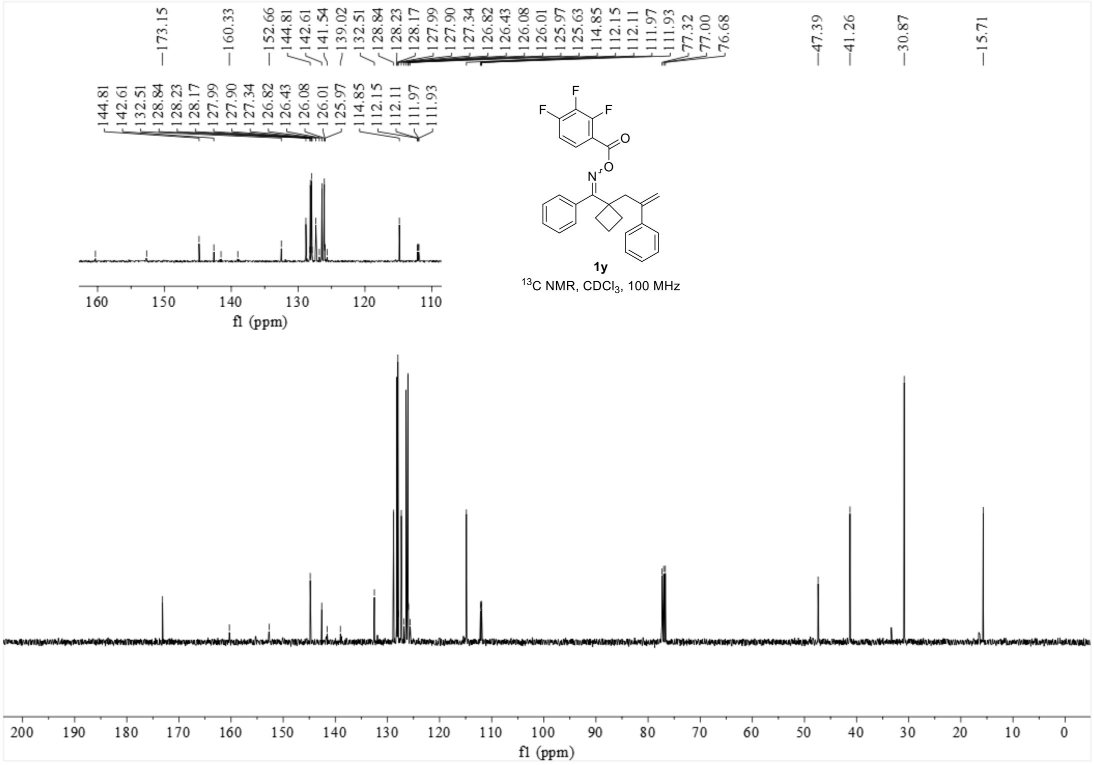
1y
^{13}C NMR, CDCl$_3$, 100 MHz
f1 (ppm)

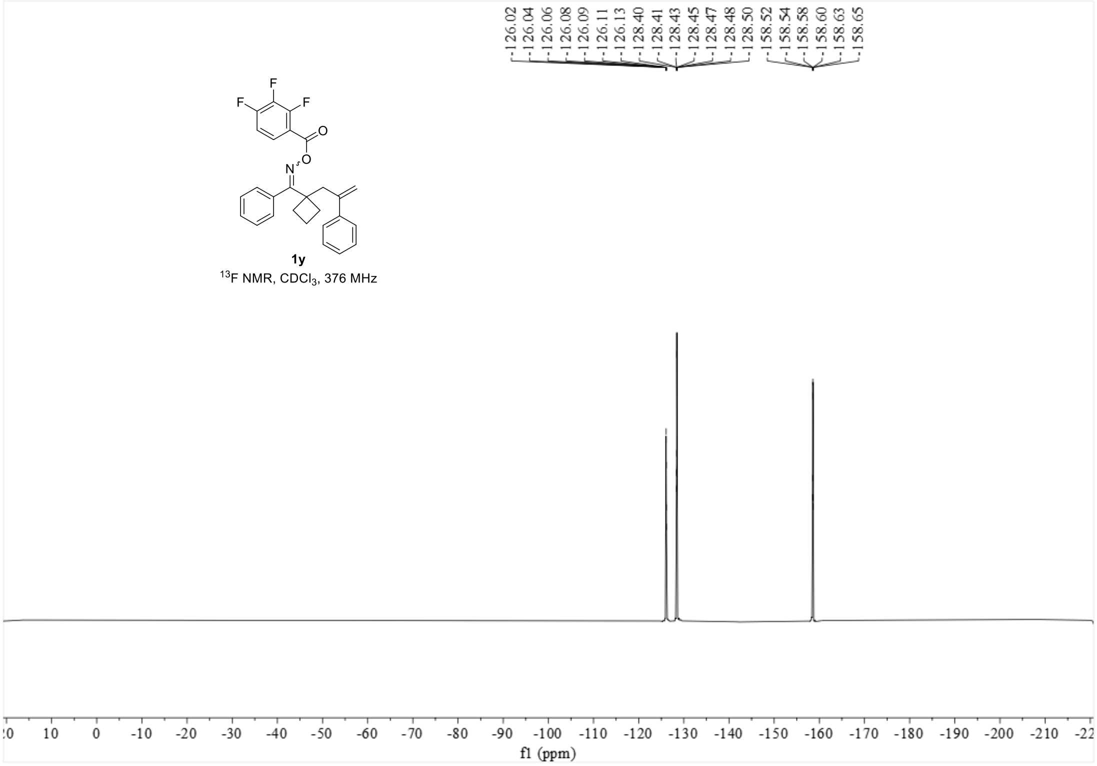
1y
13F NMR, CDCl3, 376 MHz
f1 (ppm)

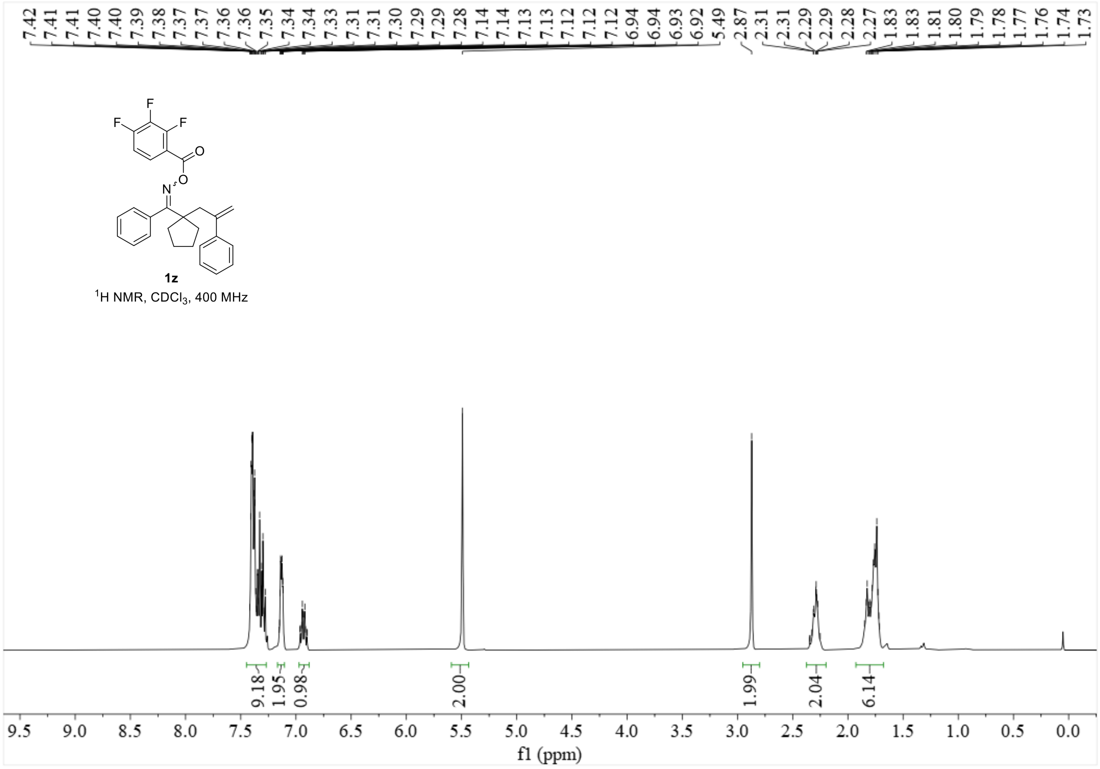
1z
1H NMR, CDCl3, 400 MHz
f1 (ppm)

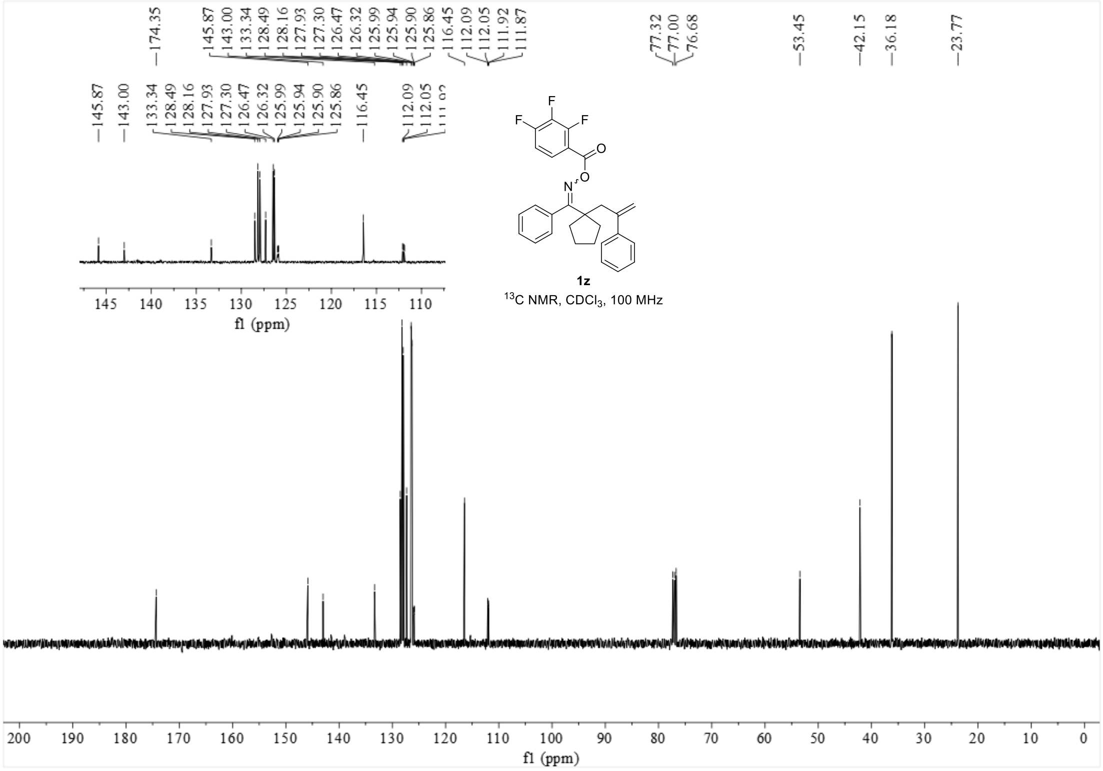
1z
^{13}C NMR, CDCl$_3$, 100 MHz
f1 (ppm)

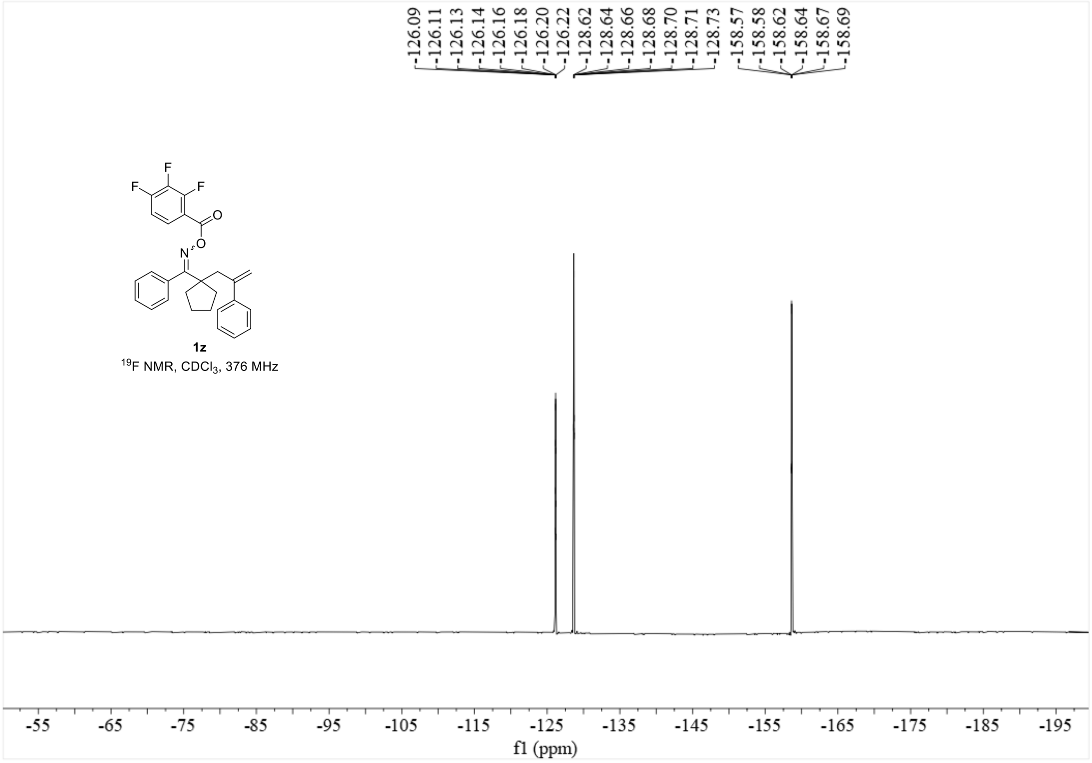
1z
^{19}F NMR, CDCl$_3$, 376 MHz
f1 (ppm)

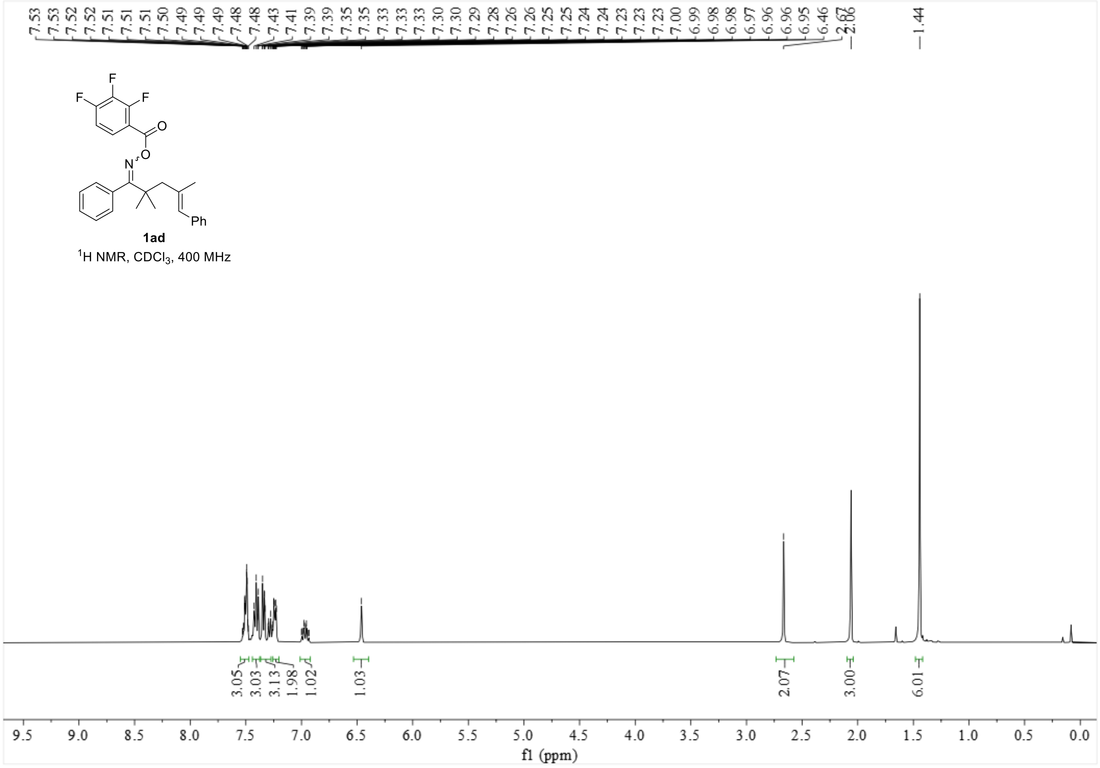
1ad
^{1}H NMR, CDCl$_3$, 400 MHz
f1 (ppm)

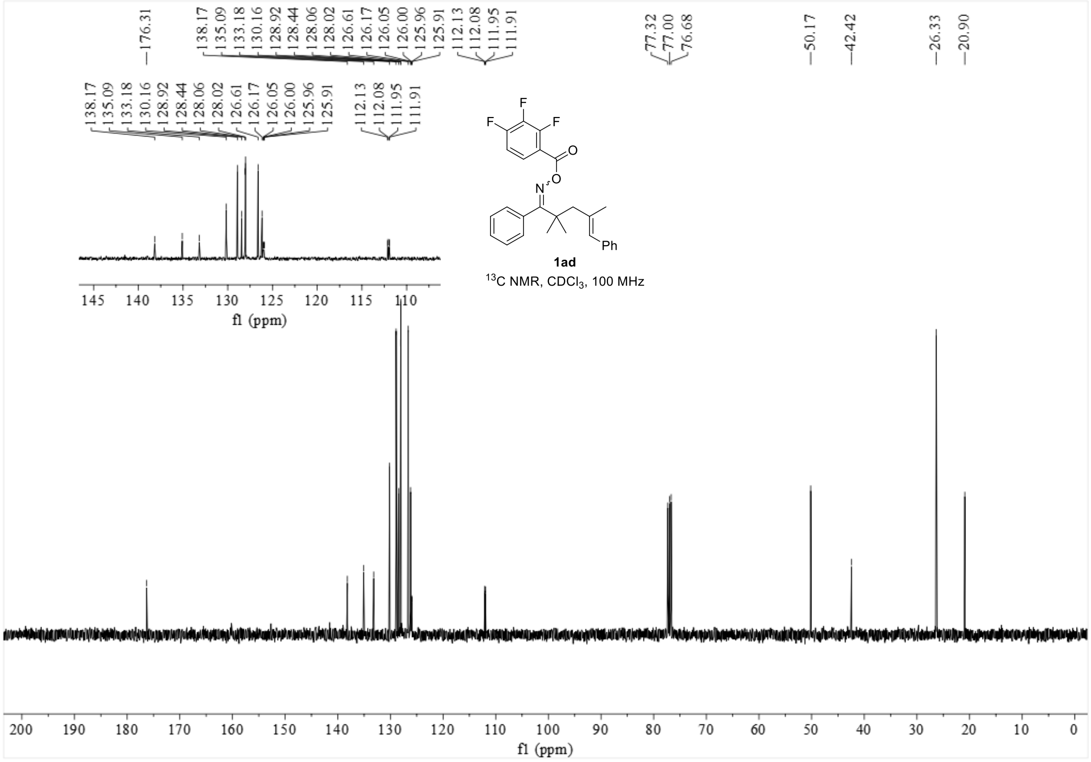
1ad
^{13}C NMR, CDCl$_3$, 100 MHz
f1 (ppm)

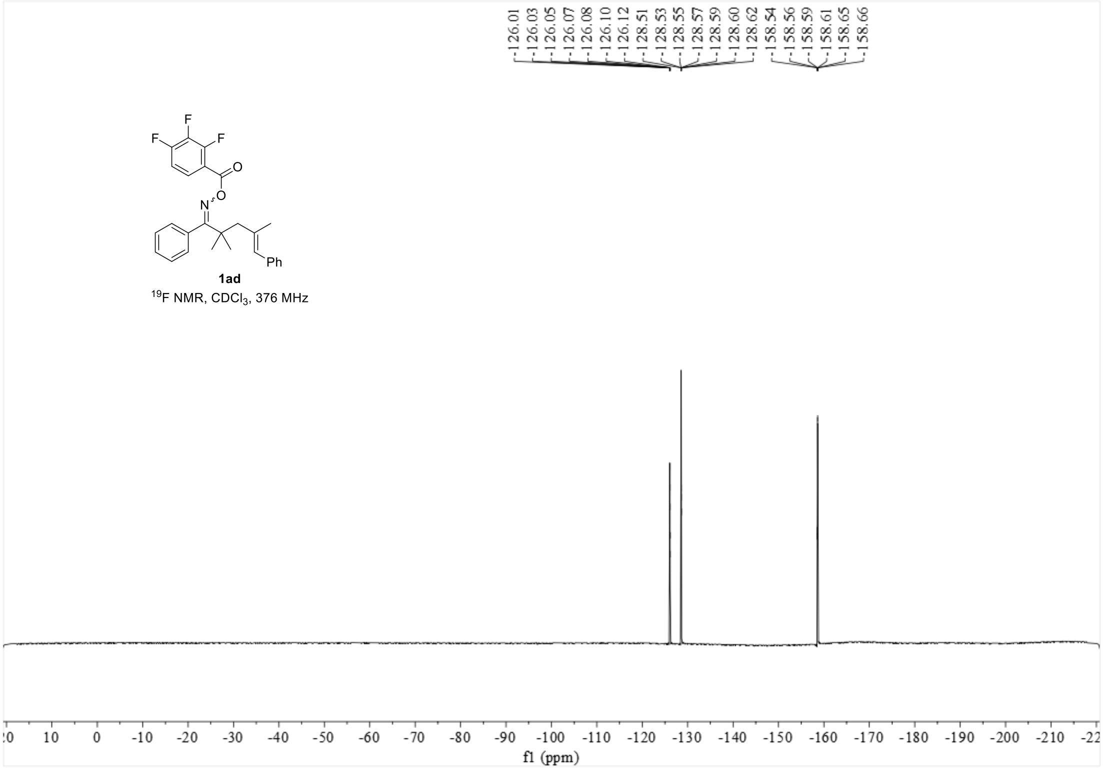

126.01
126.03
126.05
126.07
126.08
126.10
126.12
128.51
128.53
128.55
128.57
128.59
128.60
128.62
158.54
158.56
158.59
158.61
158.65
158.66
F
F
F
O
N
O
Ph
1ad
19F NMR, CDCl3, 376 MHz
f1 (ppm)

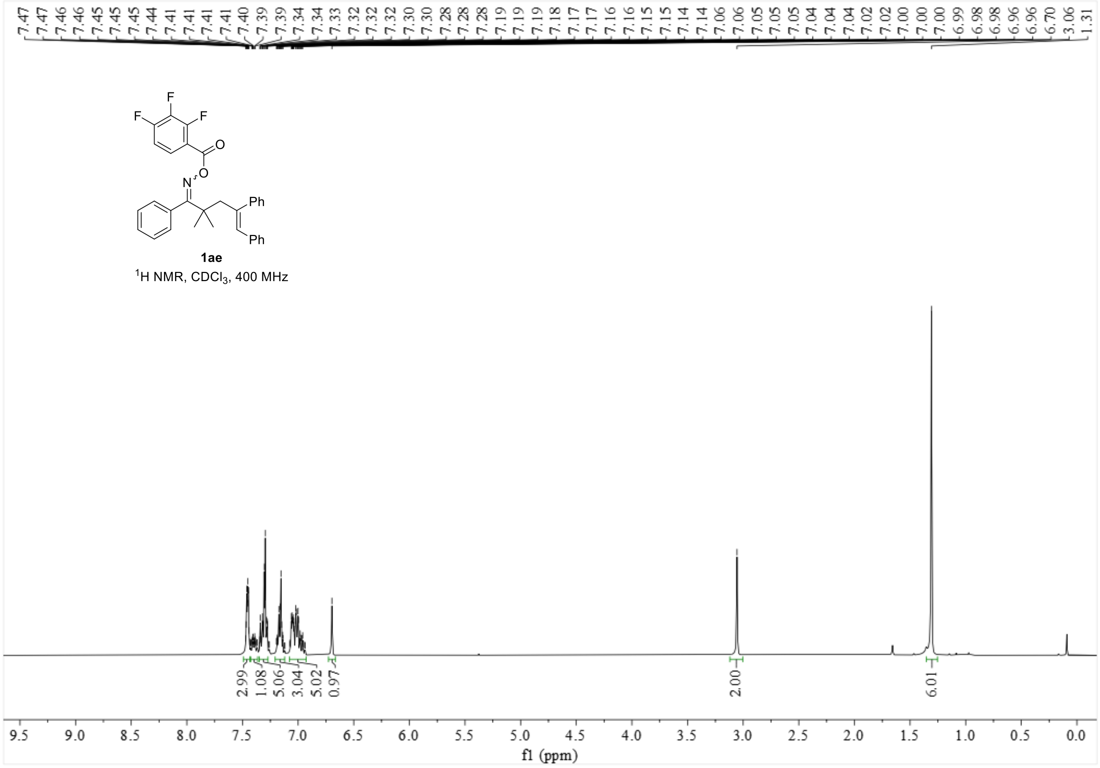

7.47
7.47
7.46
7.45
7.45
7.45
7.44
7.41
7.41
7.41
7.41
7.40
7.39
7.39
7.34
7.33
7.32
7.32
7.32
7.30
7.30
7.28
7.28
7.28
7.19
7.19
7.18
7.17
7.17
7.16
7.16
7.15
7.15
7.14
7.06
7.06
7.05
7.05
7.04
7.04
7.04
7.02
7.02
7.00
7.00
6.99
6.98
6.98
6.96
6.96
6.70
3.06
1.31
F
F
F
O
N
O
Ph
Ph
1ae
1H NMR, CDCl3, 400 MHz
2.99
1.08
5.06
3.04
5.02
0.97
2.00
6.01
f1 (ppm)

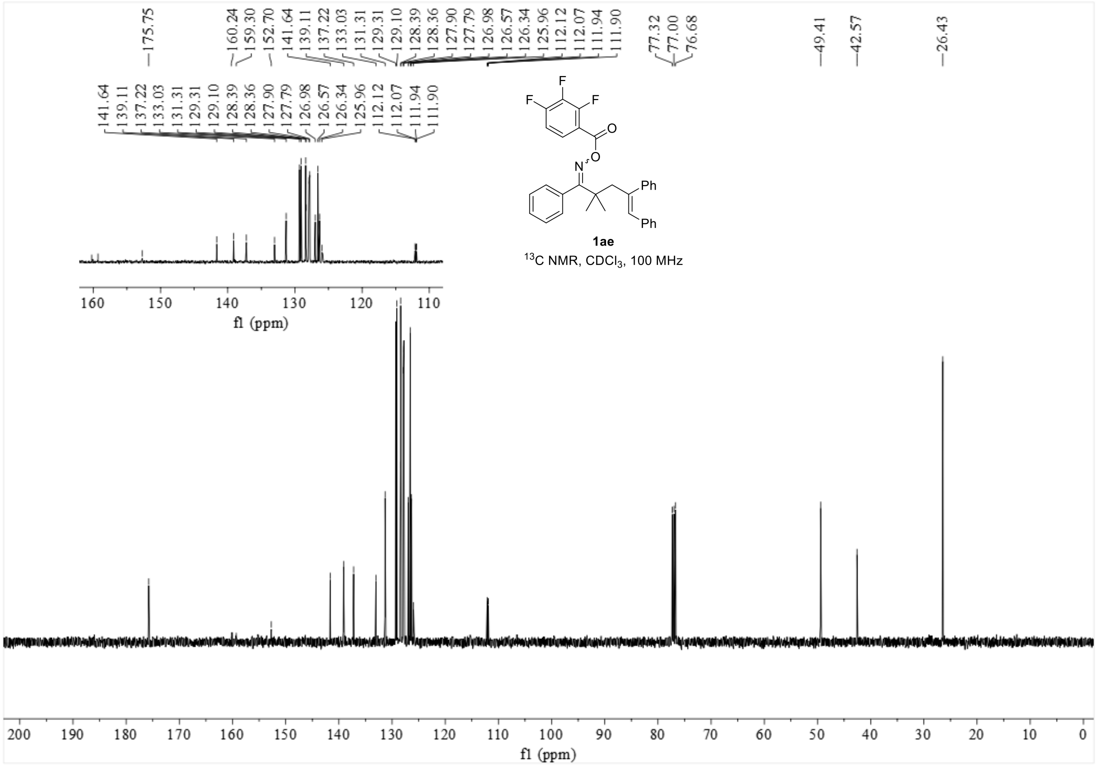
175.75
160.24
159.30
152.70
141.64
139.11
137.22
133.03
131.31
129.31
129.10
128.39
128.36
127.90
127.79
126.98
126.57
126.34
125.96
112.12
112.07
111.94
111.90
77.32
77.00
76.68
49.41
42.57
26.43
1ae
^{13}C NMR, CDCl$_3$, 100 MHz
f1 (ppm)

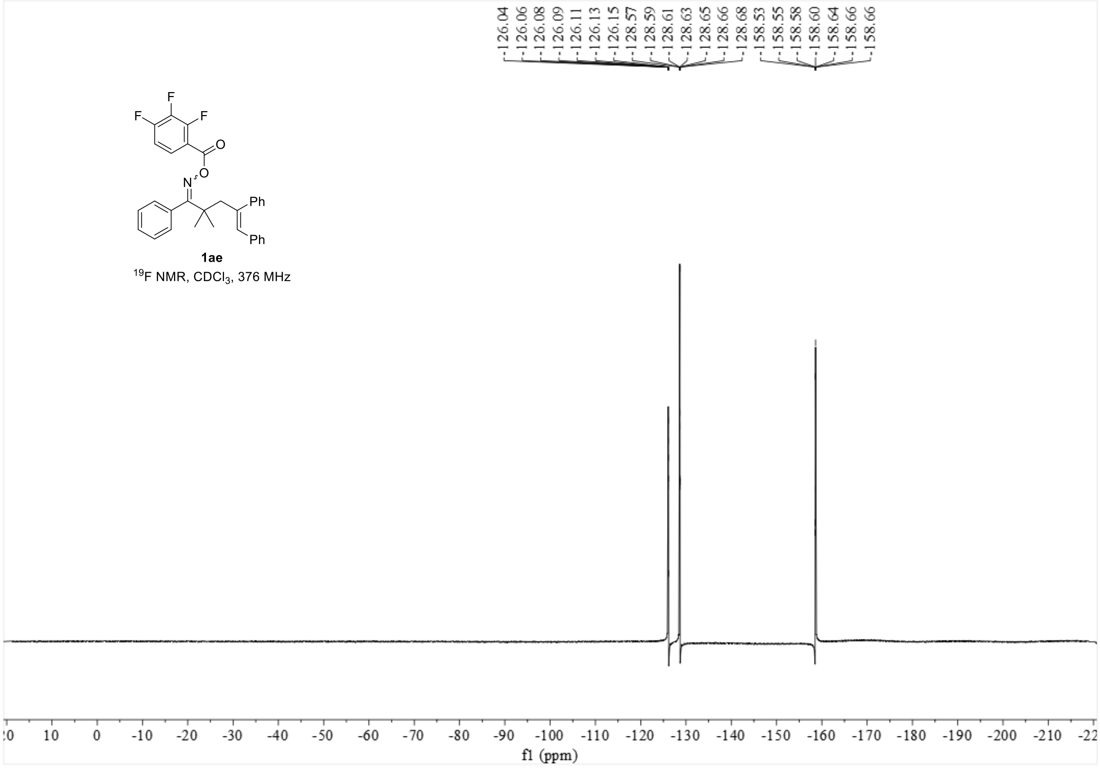
-126.04
-126.06
-126.08
-126.09
-126.11
-126.13
-126.15
-128.57
-128.59
-128.61
-128.63
-128.65
-128.66
-128.68
-158.53
-158.55
-158.58
-158.60
-158.64
-158.66
-158.66
1ae
^{19}F NMR, CDCl$_3$, 376 MHz
f1 (ppm)

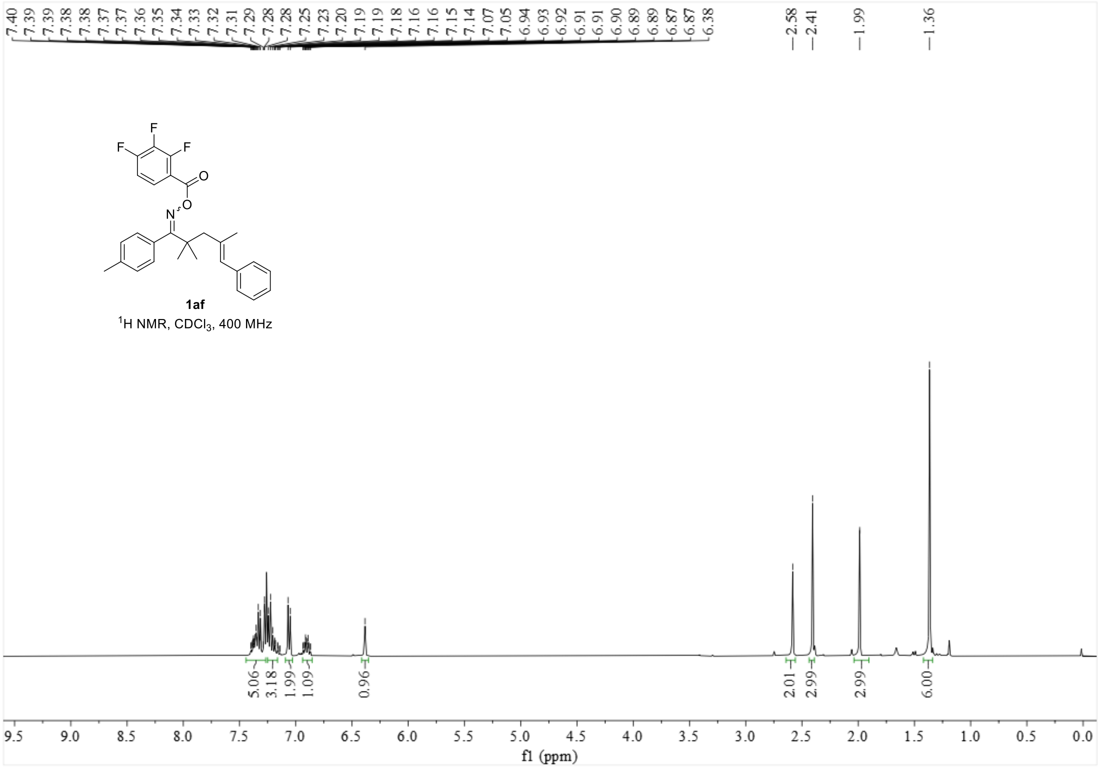

7.40 7.39 7.39 7.38 7.38 7.37 7.37 7.36 7.35 7.34 7.33 7.32 7.31 7.29 7.28 7.28 7.25 7.23 7.20 7.19 7.19 7.18 7.16 7.16 7.15 7.14 7.07 7.05 6.94 6.93 6.92 6.91 6.91 6.90 6.89 6.89 6.87 6.87 6.38
2.58 2.41 1.99 1.36
F F F O N O
1af
^{1}H NMR, CDCl$_3$, 400 MHz
5.06 3.18 1.99 1.09 0.96 2.01 2.99 2.99 6.00
9.5 9.0 8.5 8.0 7.5 7.0 6.5 6.0 5.5 5.0 4.5 4.0 3.5 3.0 2.5 2.0 1.5 1.0 0.5 0.0
f1 (ppm)

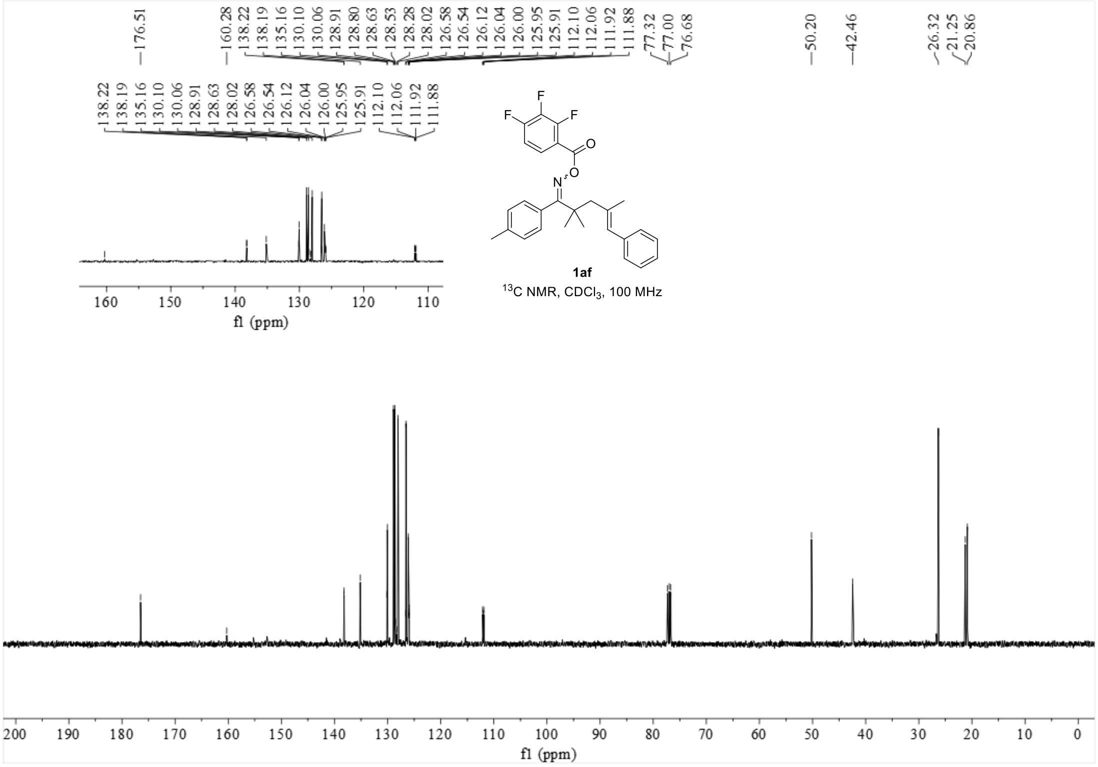

176.51
160.28 138.22 138.19 135.16 130.10 130.06 128.91 128.80 128.63 128.53 128.28 128.02 126.58 126.54 126.12 126.04 126.00 125.95 125.91 112.10 112.06 111.92 111.88
77.32 77.00 76.68
50.20 42.46 26.32 21.25 20.86
138.22 138.19 135.16 130.10 130.06 128.91 128.63 128.02 126.58 126.54 126.12 126.04 126.00 125.95 125.91 112.10 112.06 111.92 111.88
160 150 140 130 120 110
f1 (ppm)
F F F O N O
1af
^{13}C NMR, CDCl$_3$, 100 MHz
200 190 180 170 160 150 140 130 120 110 100 90 80 70 60 50 40 30 20 10 0
f1 (ppm)

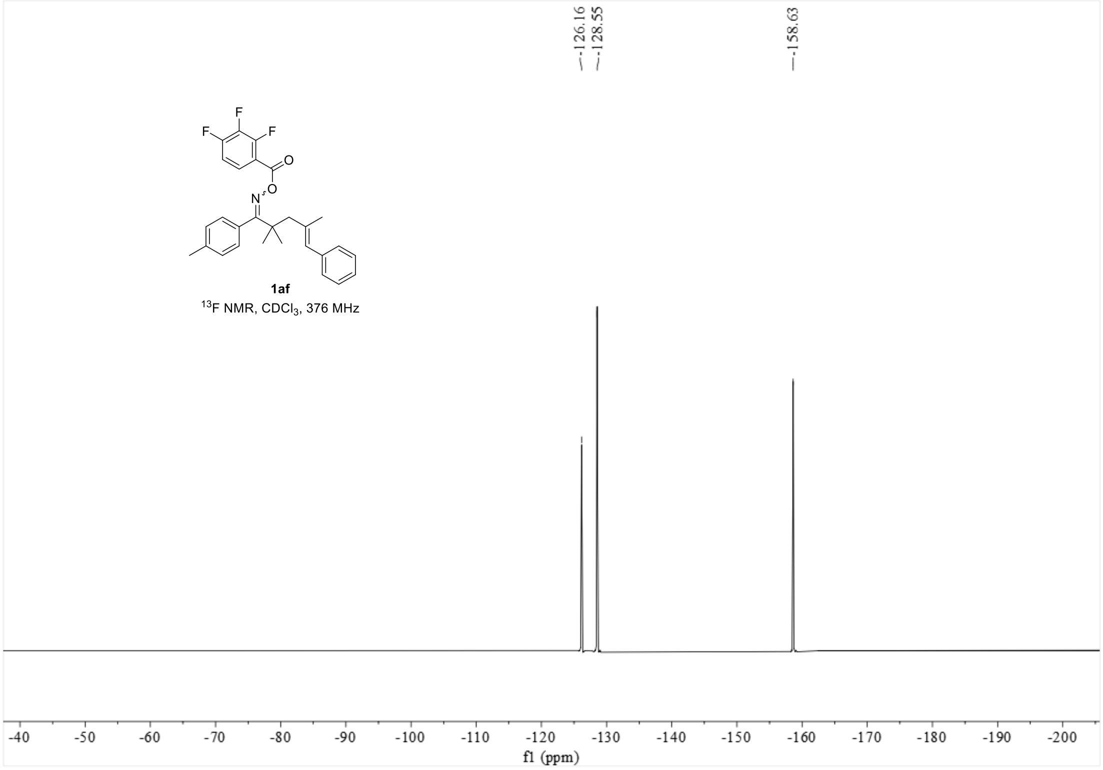
-126.16
-128.55
-158.63
1af
13F NMR, CDCl3, 376 MHz
f1 (ppm)

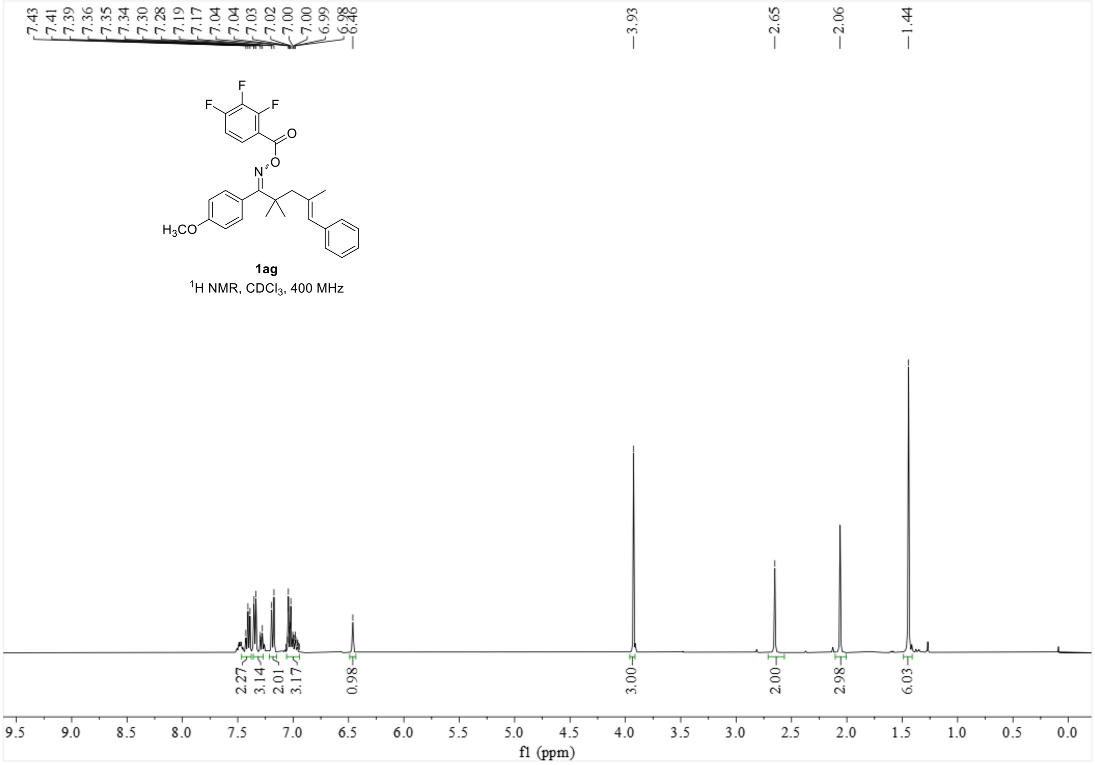
3.93
2.65
2.06
1.44
H3CO
1ag
1H NMR, CDCl3, 400 MHz
f1 (ppm)

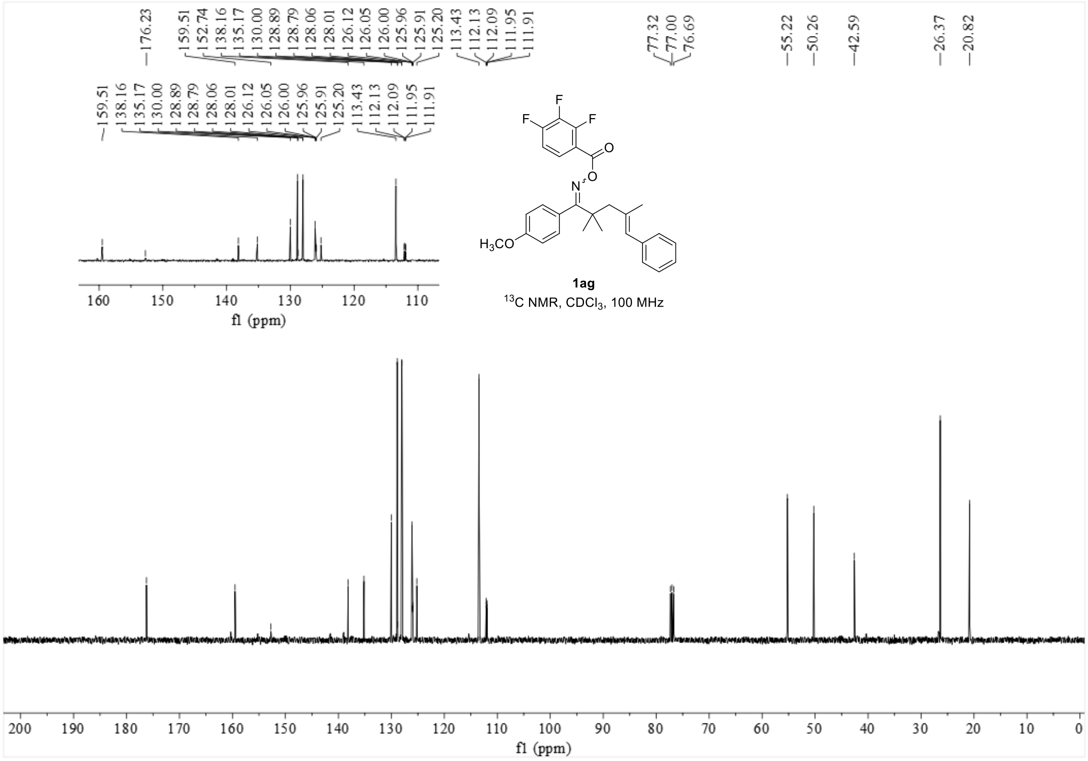
1ag
^{13}C NMR, CDCl$_3$, 100 MHz
f1 (ppm)

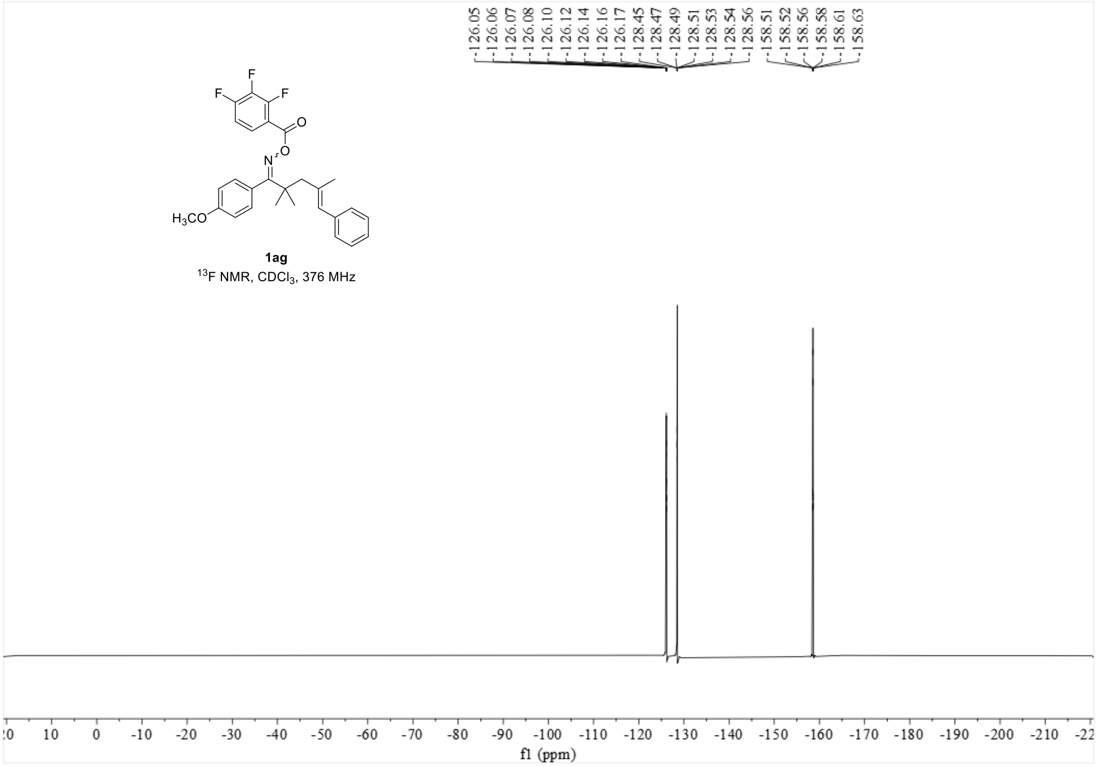
1ag
^{13}F NMR, CDCl$_3$, 376 MHz
f1 (ppm)

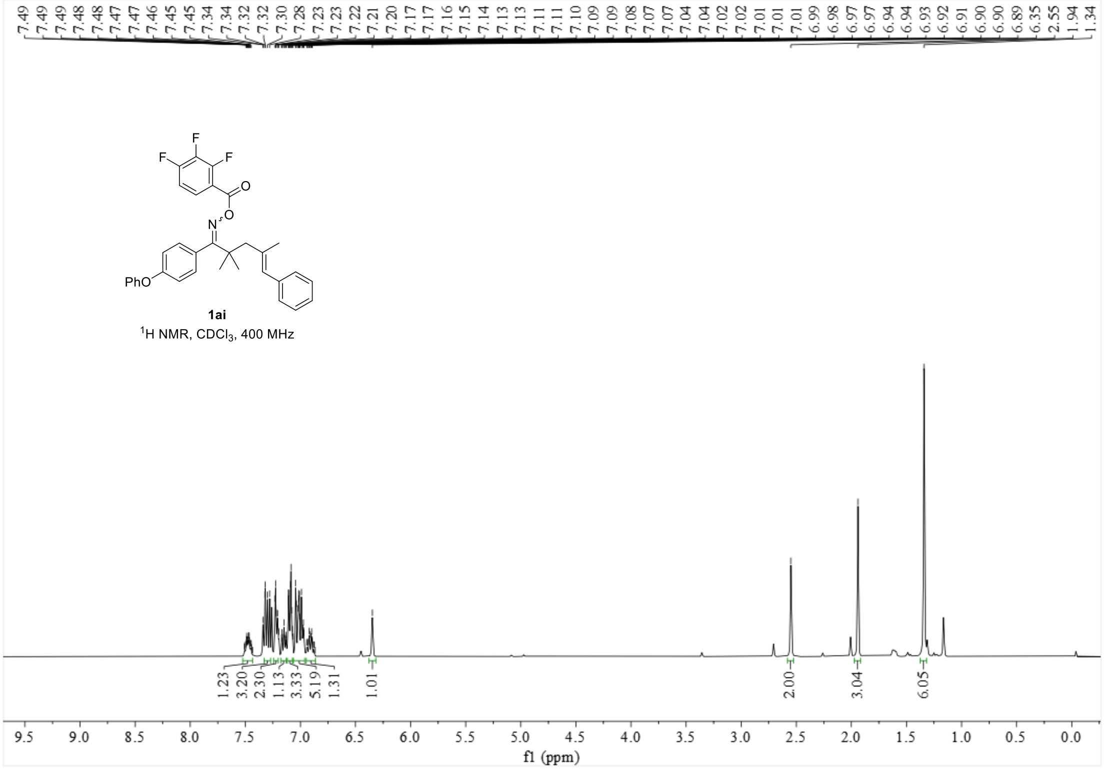
1ai
^{1}H NMR, CDCl$_3$, 400 MHz
f1 (ppm)

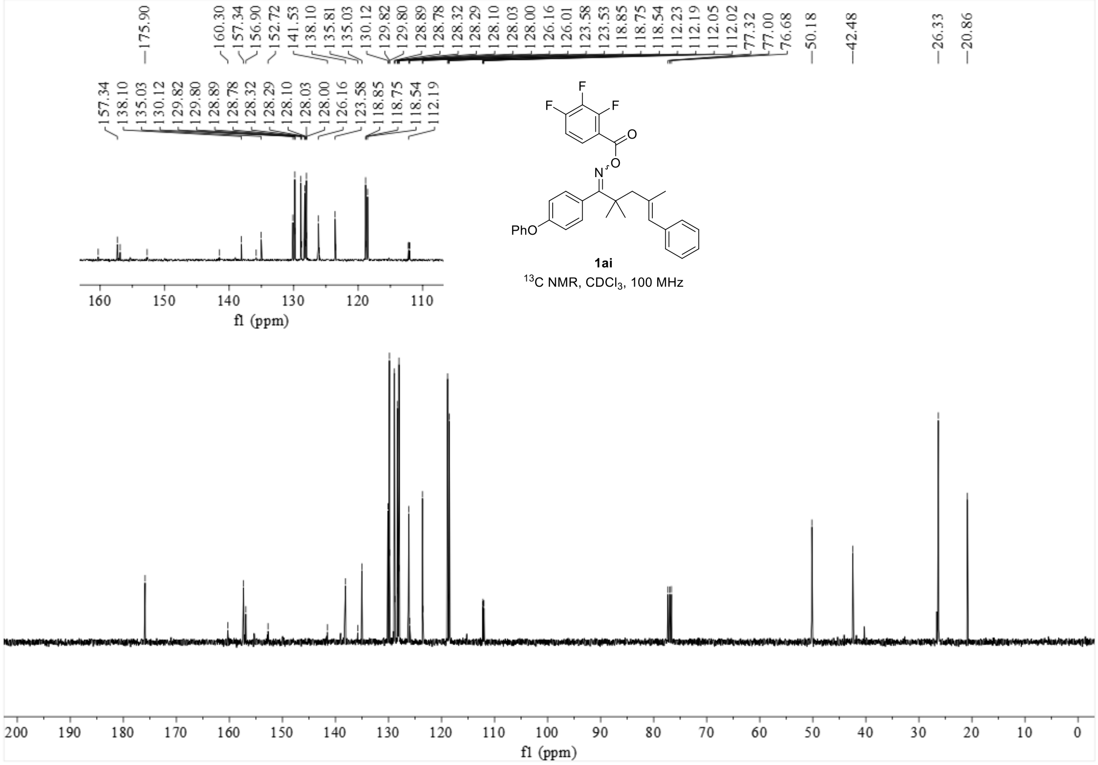
1ai
^{13}C NMR, CDCl$_3$, 100 MHz
f1 (ppm)

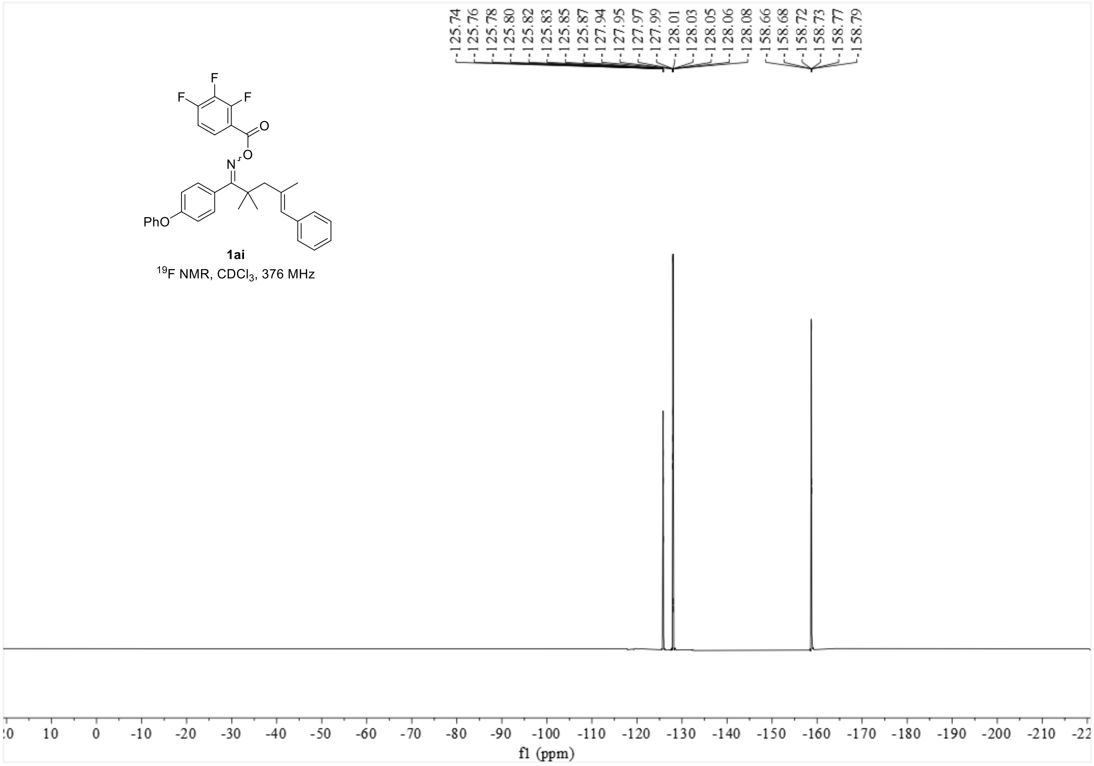
-125.74
-125.76
-125.78
-125.80
-125.82
-125.83
-125.85
-125.87
-127.94
-127.95
-127.97
-127.99
-128.01
-128.03
-128.05
-128.06
-128.08
-158.66
-158.68
-158.72
-158.73
-158.77
-158.79
F
F
F
O
N
O
PhO
1ai
19F NMR, CDCl3, 376 MHz
f1 (ppm)

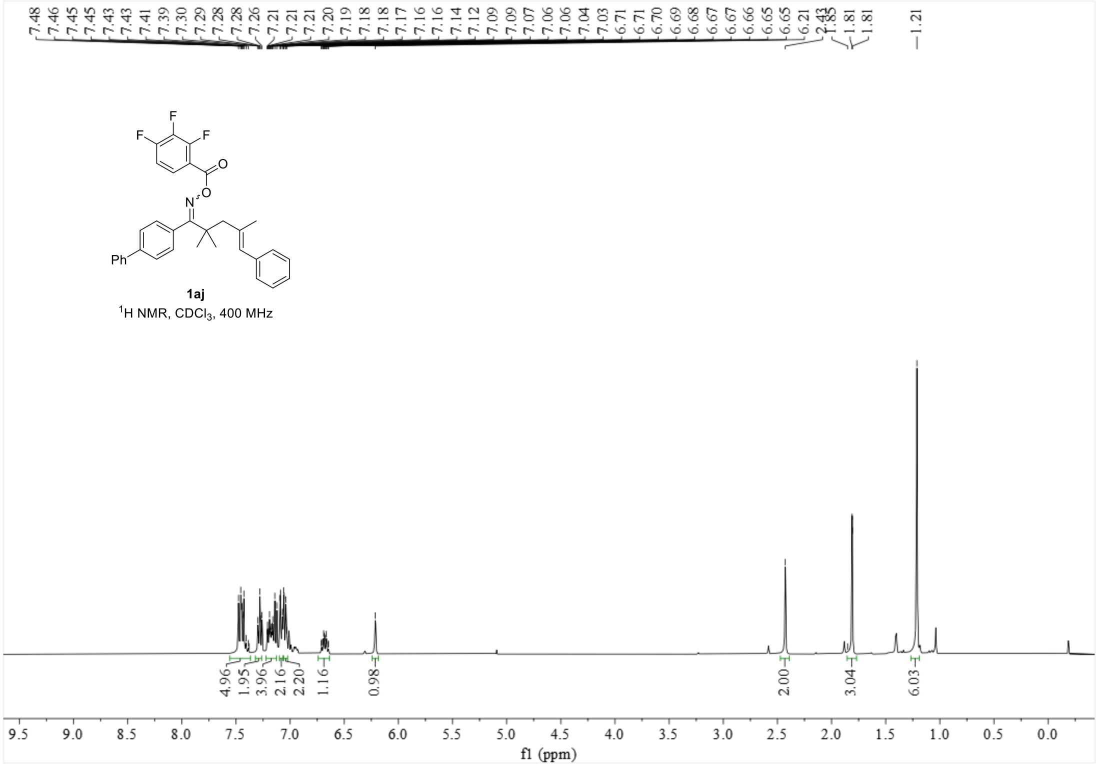
7.48
7.46
7.45
7.45
7.43
7.43
7.41
7.39
7.30
7.29
7.28
7.28
7.26
7.21
7.21
7.21
7.20
7.19
7.18
7.18
7.17
7.16
7.16
7.14
7.12
7.09
7.07
7.06
7.04
7.03
6.71
6.70
6.69
6.68
6.67
6.66
6.65
6.21
2.43
1.85
1.81
1.81
1.21
F
F
F
O
N
O
Ph
1aj
1H NMR, CDCl3, 400 MHz
4.96
1.95
3.96
2.16
2.20
1.16
0.98
2.00
3.04
6.03
f1 (ppm)

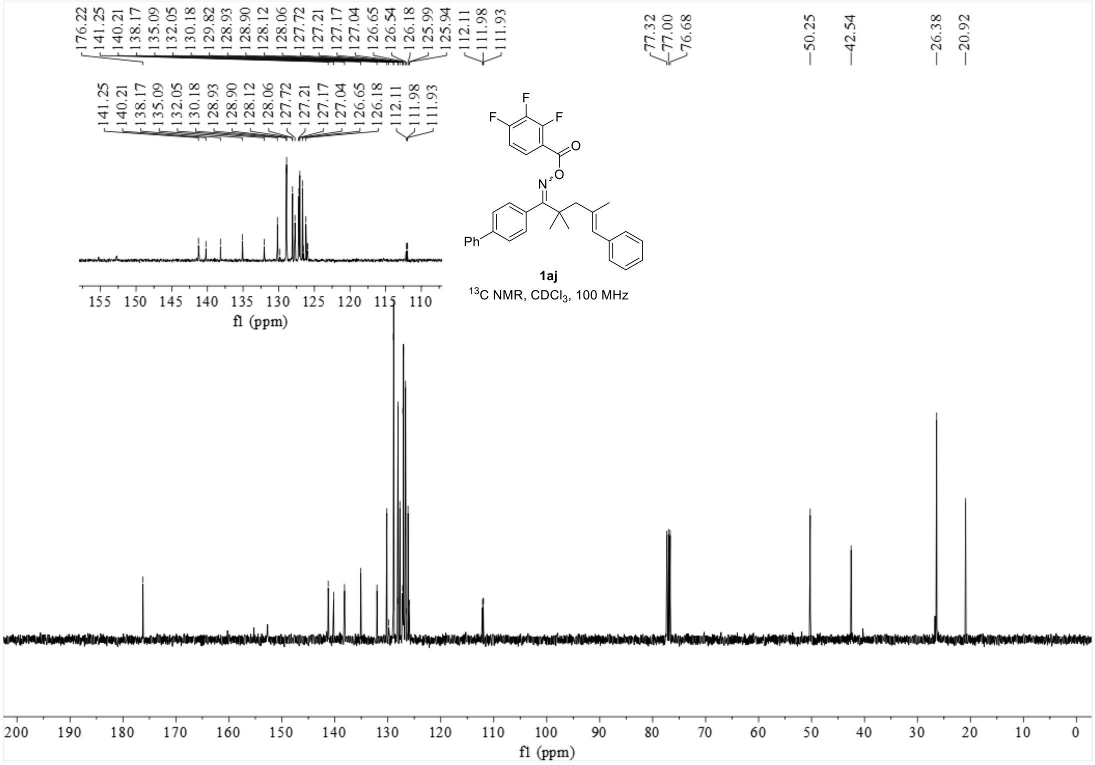
176.22
141.25
140.21
138.17
135.09
132.05
130.18
129.82
128.93
128.90
128.12
128.06
127.72
127.21
127.17
127.04
126.65
126.54
126.18
125.99
125.94
112.11
111.98
111.93
77.32
77.00
76.68
50.25
42.54
26.38
20.92
1aj
^{13}C NMR, CDCl$_3$, 100 MHz
f1 (ppm)

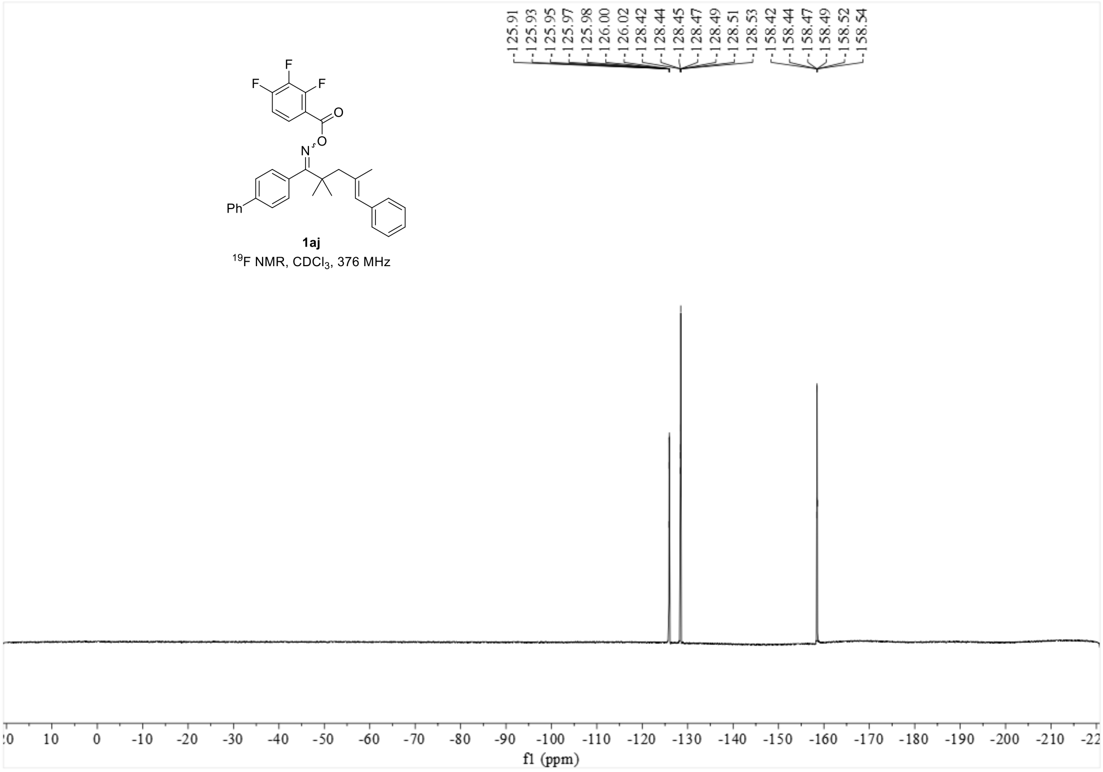
-125.91
-125.93
-125.95
-125.97
-125.98
-126.00
-126.02
-128.42
-128.44
-128.45
-128.47
-128.49
-128.51
-128.53
-158.42
-158.44
-158.47
-158.49
-158.52
-158.54
1aj
^{19}F NMR, CDCl$_3$, 376 MHz
f1 (ppm)

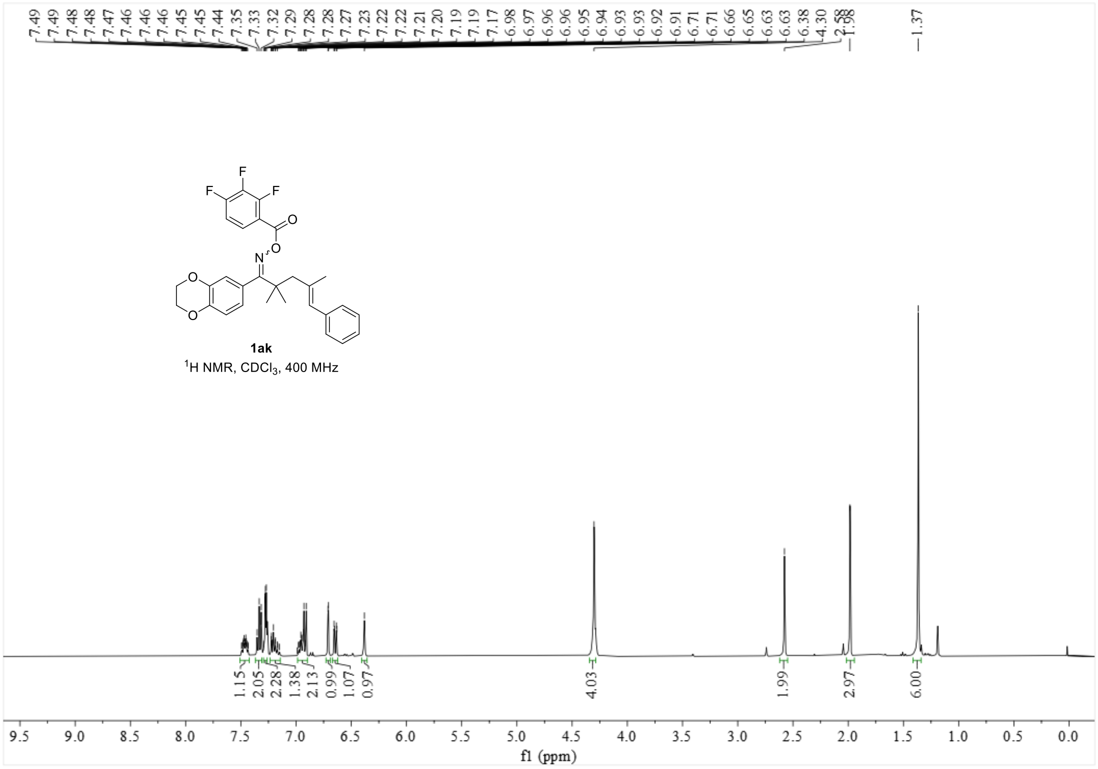
7.49 7.49 7.48 7.48 7.47 7.46 7.46 7.46 7.45 7.45 7.44 7.35 7.33 7.32 7.29 7.28 7.28 7.27 7.23 7.22 7.22 7.21 7.20 7.19 7.19 7.17 6.98 6.97 6.96 6.96 6.95 6.94 6.93 6.93 6.92 6.91 6.71 6.71 6.66 6.65 6.63 6.63 6.38 4.30 2.58 1.98
1.37
F
F
F
O
O
N
O
O
1ak
1H NMR, CDCl3, 400 MHz
1.15 2.05 2.28 1.38 2.13 0.99 1.07 0.97
4.03
1.99
2.97
6.00
9.5 9.0 8.5 8.0 7.5 7.0 6.5 6.0 5.5 5.0 4.5 4.0 3.5 3.0 2.5 2.0 1.5 1.0 0.5 0.0
f1 (ppm)

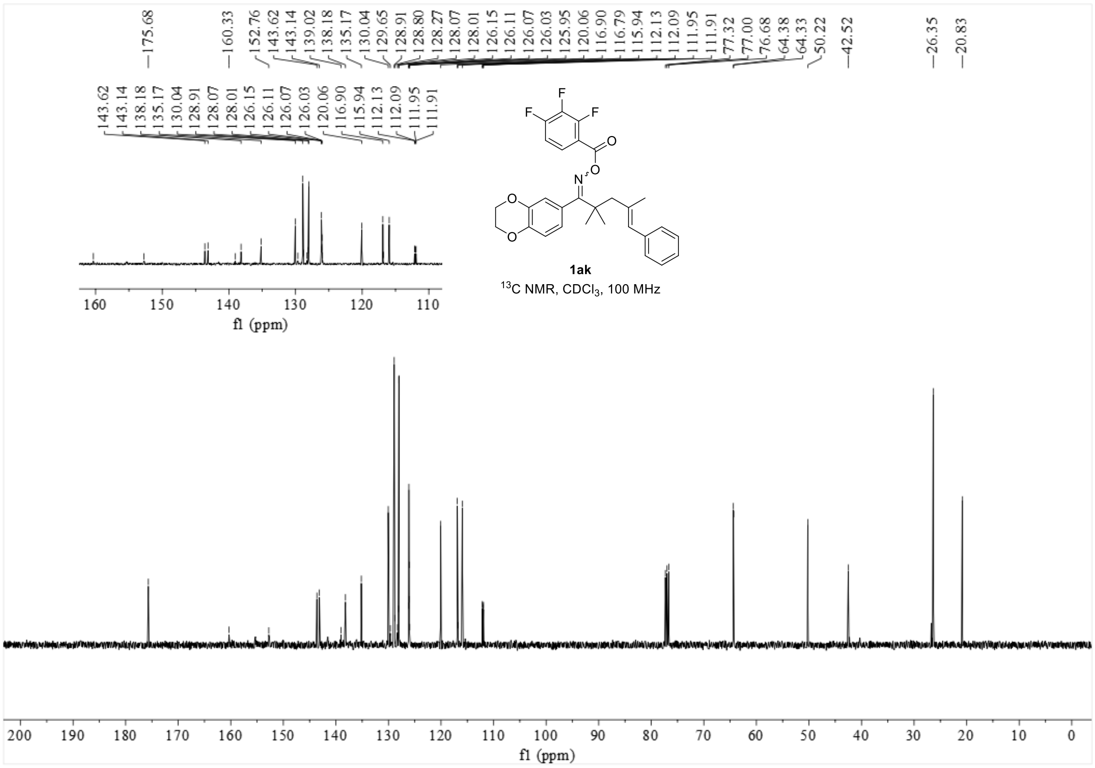
175.68
160.33 152.76 143.62 143.14 139.02 138.18 135.17 130.04 129.65 128.91 128.80 128.27 128.07 128.01 126.15 126.11 126.07 126.03 125.95 120.06 116.90 116.79 115.94 112.13 112.09 111.95 111.91 77.32 77.00 76.68 64.38 64.33 50.22
42.52
26.35
20.83
143.62 143.14 138.18 135.17 130.04 128.91 128.07 128.01 126.15 126.11 126.07 126.03 120.06 116.90 115.94 112.13 112.09 111.95 111.91
160 150 140 130 120 110
f1 (ppm)
F
F
F
O
O
N
O
O
1ak
13C NMR, CDCl3, 100 MHz
200 190 180 170 160 150 140 130 120 110 100 90 80 70 60 50 40 30 20 10 0
f1 (ppm)

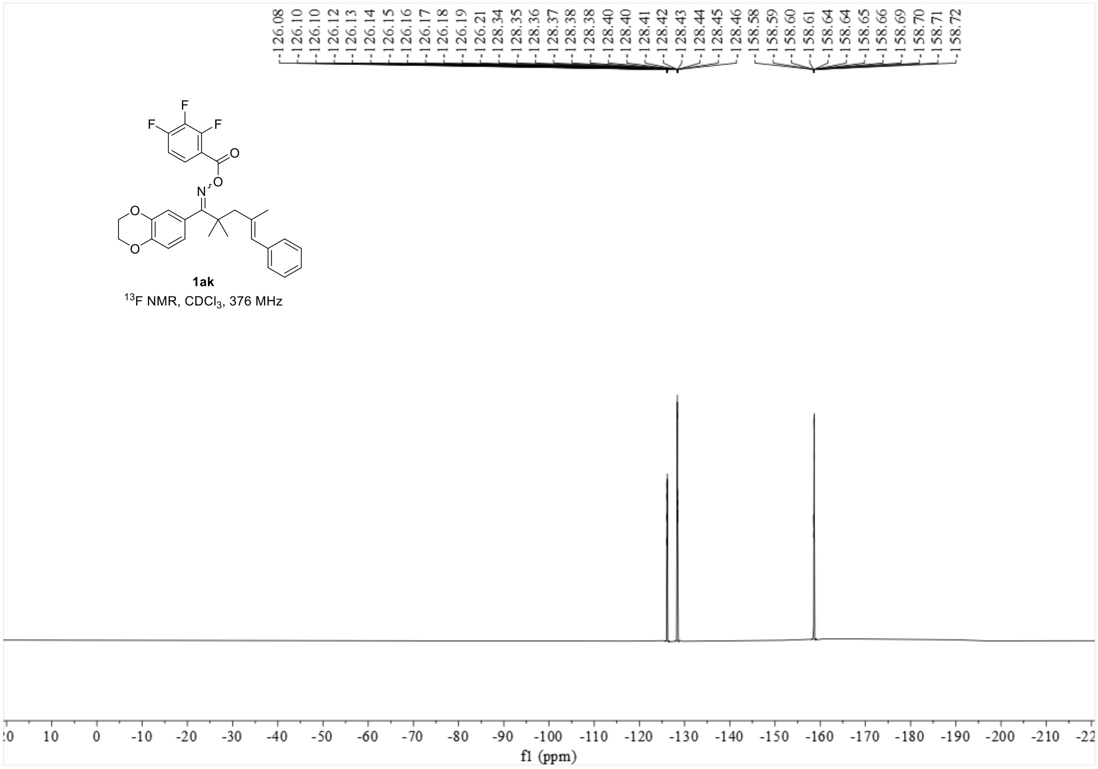
1ak
13F NMR, CDCl3, 376 MHz
f1 (ppm)

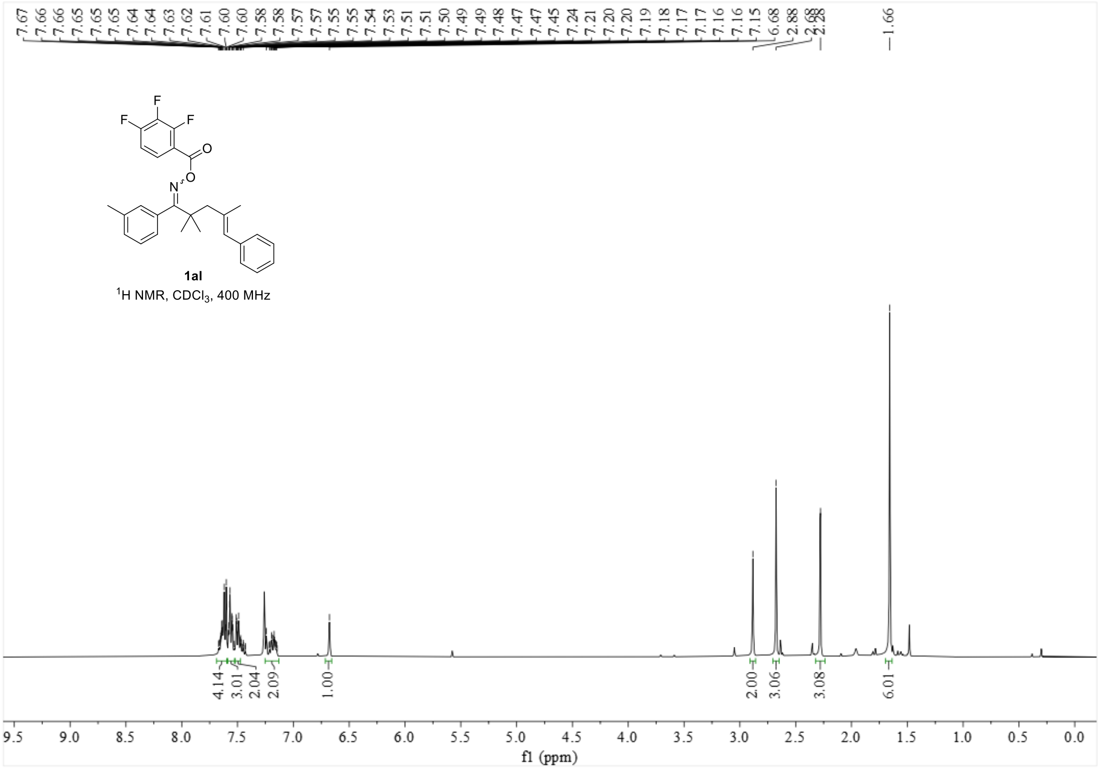
1al
1H NMR, CDCl3, 400 MHz
f1 (ppm)

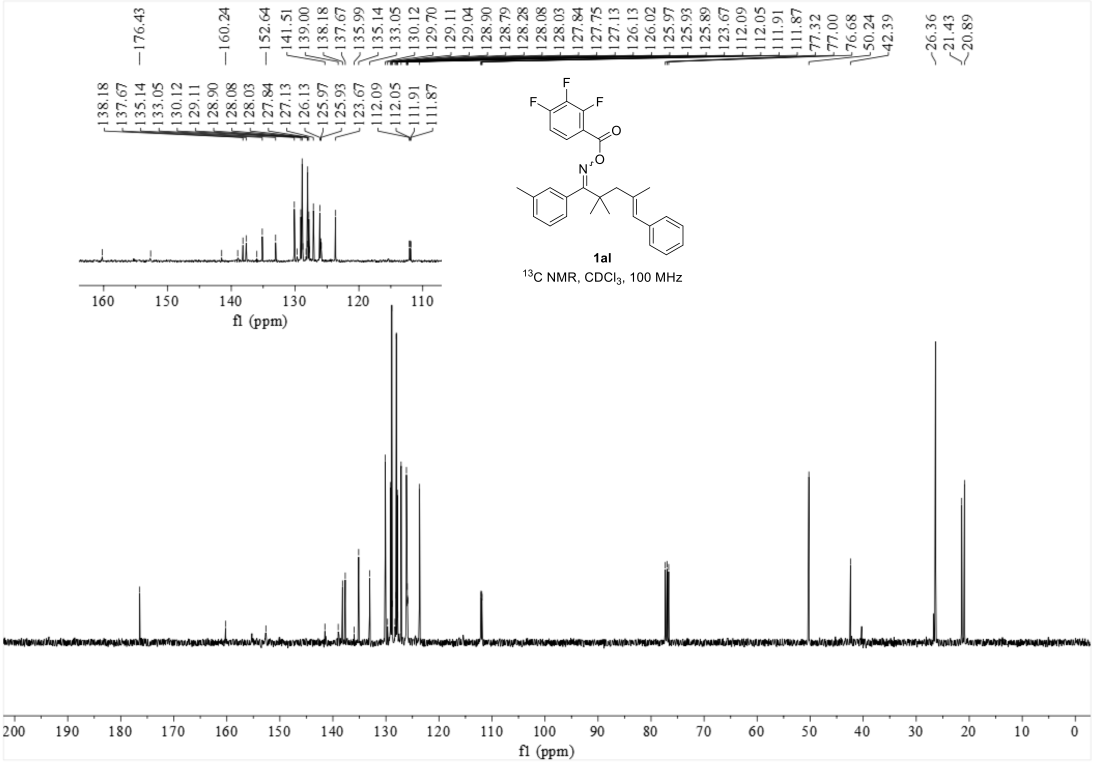
176.43
160.24
152.64
141.51
139.00
138.18
137.67
135.99
135.14
133.05
130.12
129.70
129.11
129.04
128.90
128.79
128.28
128.08
128.03
127.84
127.75
127.13
126.13
126.02
125.97
125.93
125.89
123.67
112.09
112.05
111.91
111.87
77.32
77.00
76.68
50.24
42.39
26.36
21.43
20.89
F
F
F
O
N
O
1al
^{13}C NMR, CDCl$_3$, 100 MHz
f1 (ppm)

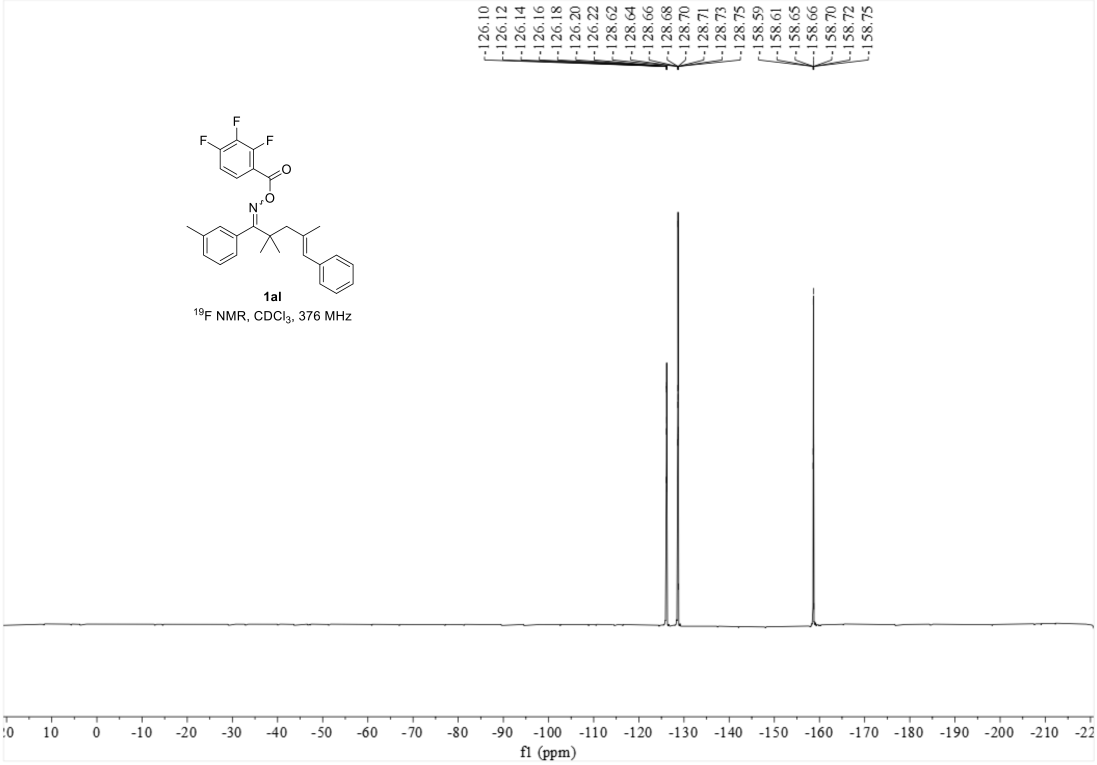
-126.10
-126.12
-126.14
-126.16
-126.18
-126.20
-126.22
-128.62
-128.64
-128.66
-128.68
-128.70
-128.71
-128.73
-128.75
-158.59
-158.61
-158.65
-158.66
-158.70
-158.72
-158.75
F
F
F
O
N
O
1al
^{19}F NMR, CDCl$_3$, 376 MHz
f1 (ppm)

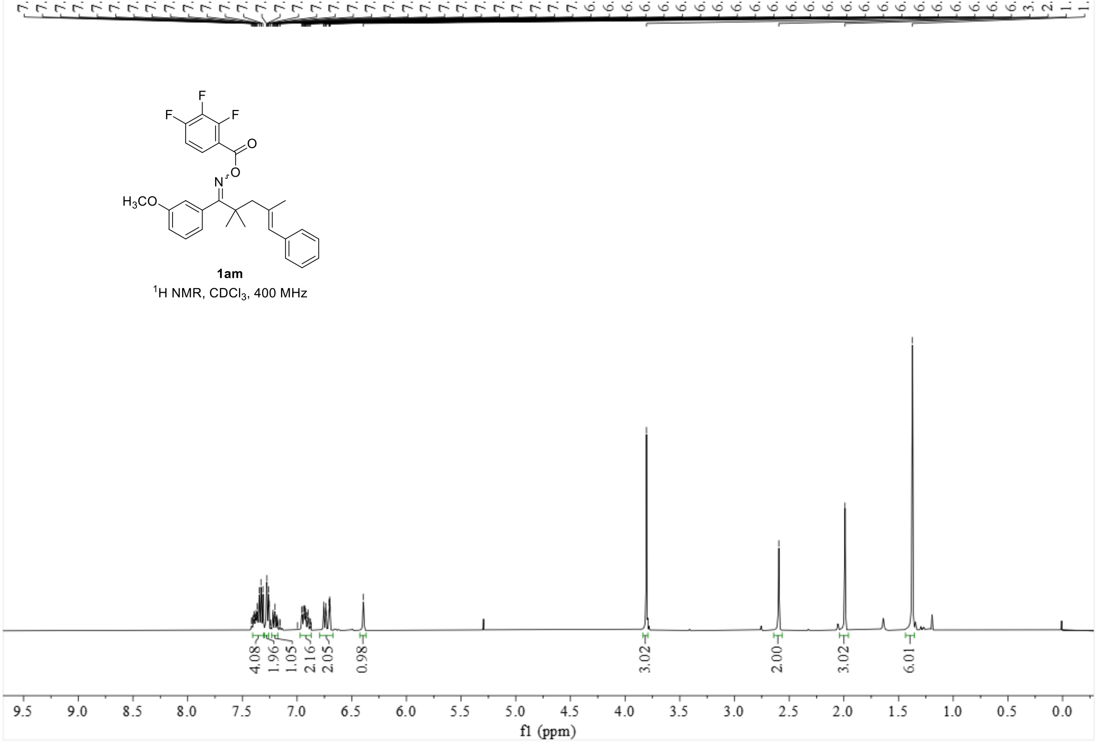
7.42 7.41 7.40 7.39 7.38 7.38 7.37 7.36 7.35 7.33 7.33 7.31 7.28 7.28 7.27 7.26 7.26 7.24 7.22 7.22 7.22 7.21 7.20 7.20 7.19 7.18 7.18 7.17 7.16 7.15 6.96 6.95 6.94 6.94 6.93 6.93 6.92 6.92 6.91 6.90 6.89 6.88 6.88 6.76 6.75 6.75 6.74 6.74 6.73 6.71 6.70 6.70 6.70 6.39 3.80 2.59 1.99 1.37
1am
1H NMR, CDCl3, 400 MHz
4.08 1.96 1.05 2.16 2.05 0.98 3.02 2.00 3.02 6.01
9.5 9.0 8.5 8.0 7.5 7.0 6.5 6.0 5.5 5.0 4.5 4.0 3.5 3.0 2.5 2.0 1.5 1.0 0.5 0.0
f1 (ppm)

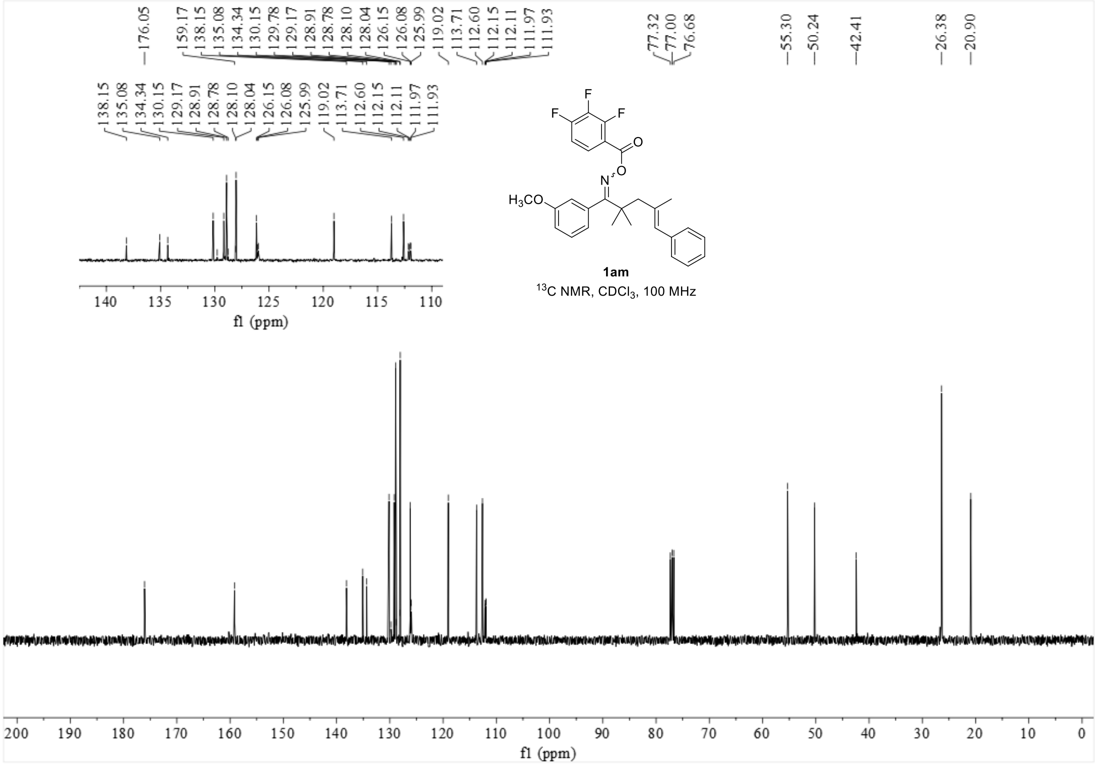
176.05 159.17 138.15 135.08 134.34 130.15 129.78 129.17 128.91 128.78 128.10 128.04 126.15 126.08 125.99 119.02 113.71 112.60 112.15 112.11 111.97 111.93 77.32 77.00 76.68 55.30 50.24 42.41 26.38 20.90
138.15 135.08 134.34 130.15 129.17 128.91 128.78 128.10 128.04 126.15 126.08 125.99 119.02 113.71 112.60 112.15 112.11 111.97 111.93
140 135 130 125 120 115 110
f1 (ppm)
1am
13C NMR, CDCl3, 100 MHz
200 190 180 170 160 150 140 130 120 110 100 90 80 70 60 50 40 30 20 10 0
f1 (ppm)

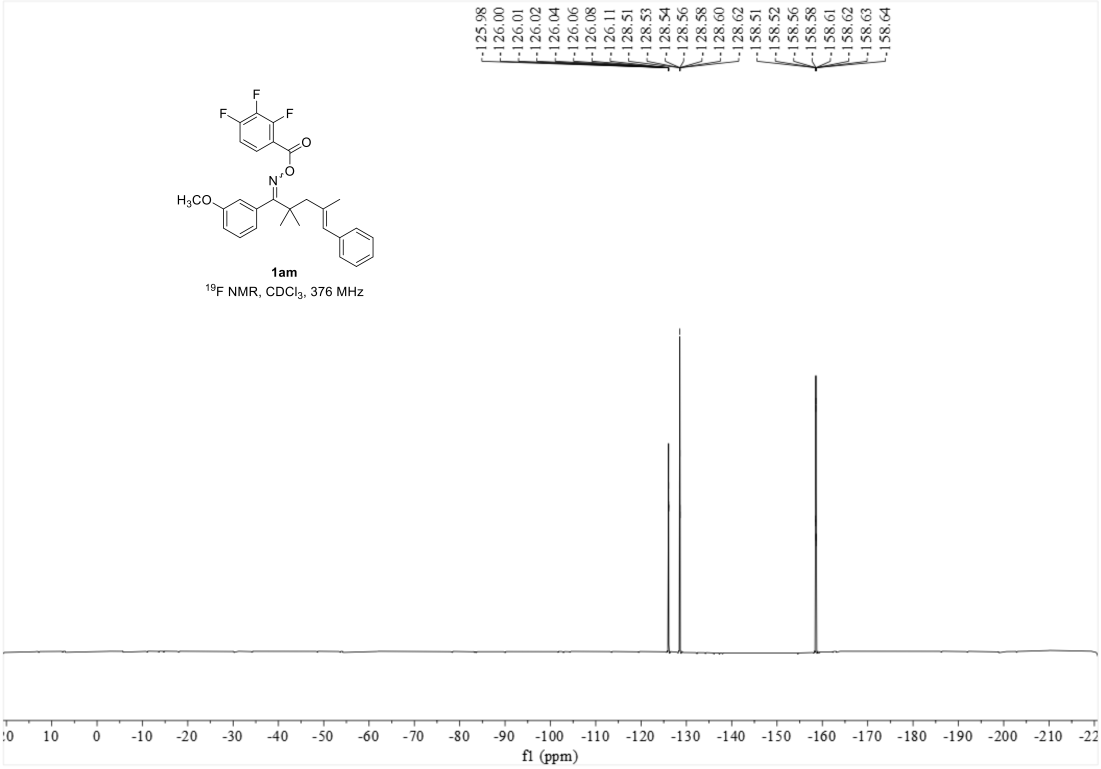

1am
^{19}F NMR, CDCl$_3$, 376 MHz
f1 (ppm)

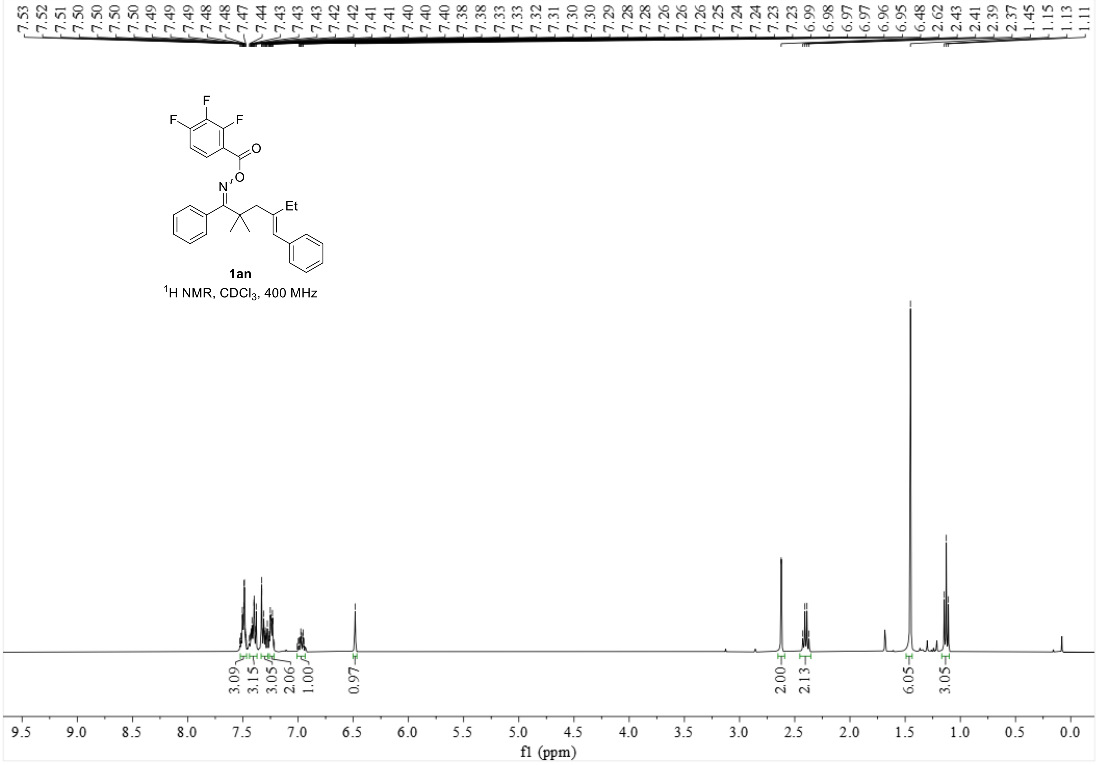

1an
^{1}H NMR, CDCl$_3$, 400 MHz
f1 (ppm)

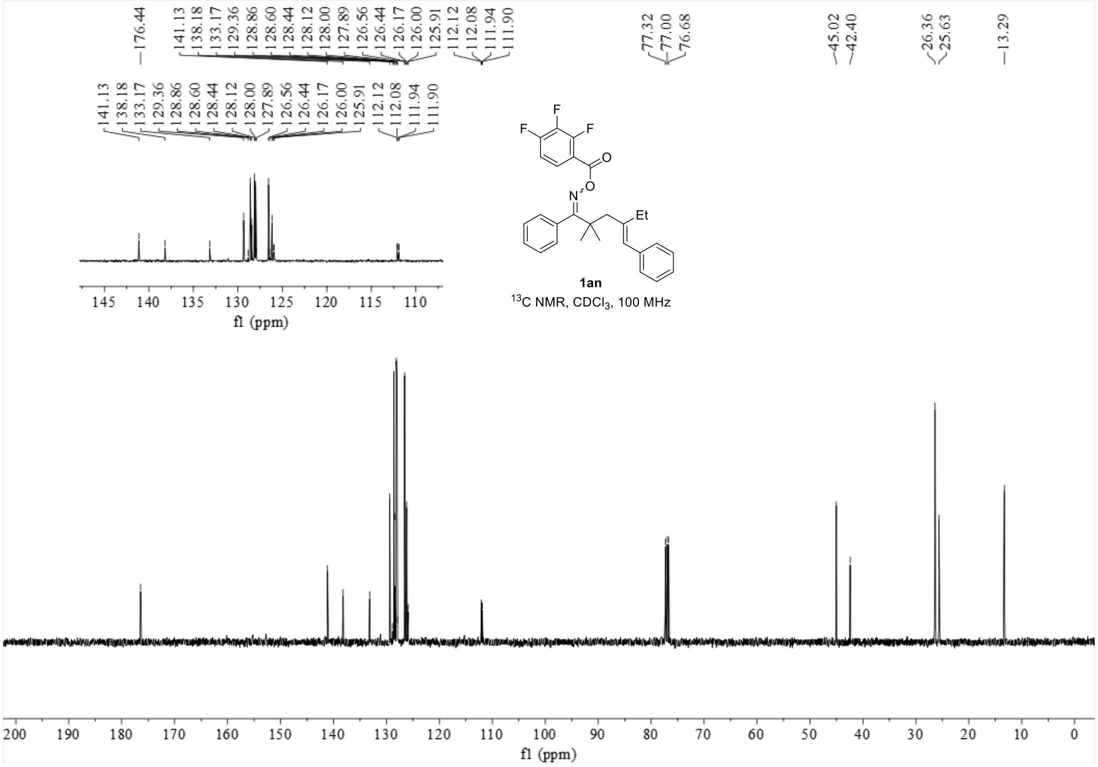
176.44
141.13 138.18 133.17 129.36 128.86 128.60 128.44 128.12 128.00 127.89 126.56 126.44 126.17 126.00 125.91 112.12 112.08 111.94 111.90
77.32 77.00 76.68
45.02 42.40
26.36 25.63
13.29
1an
^{13}C NMR, CDCl$_3$, 100 MHz
f1 (ppm)

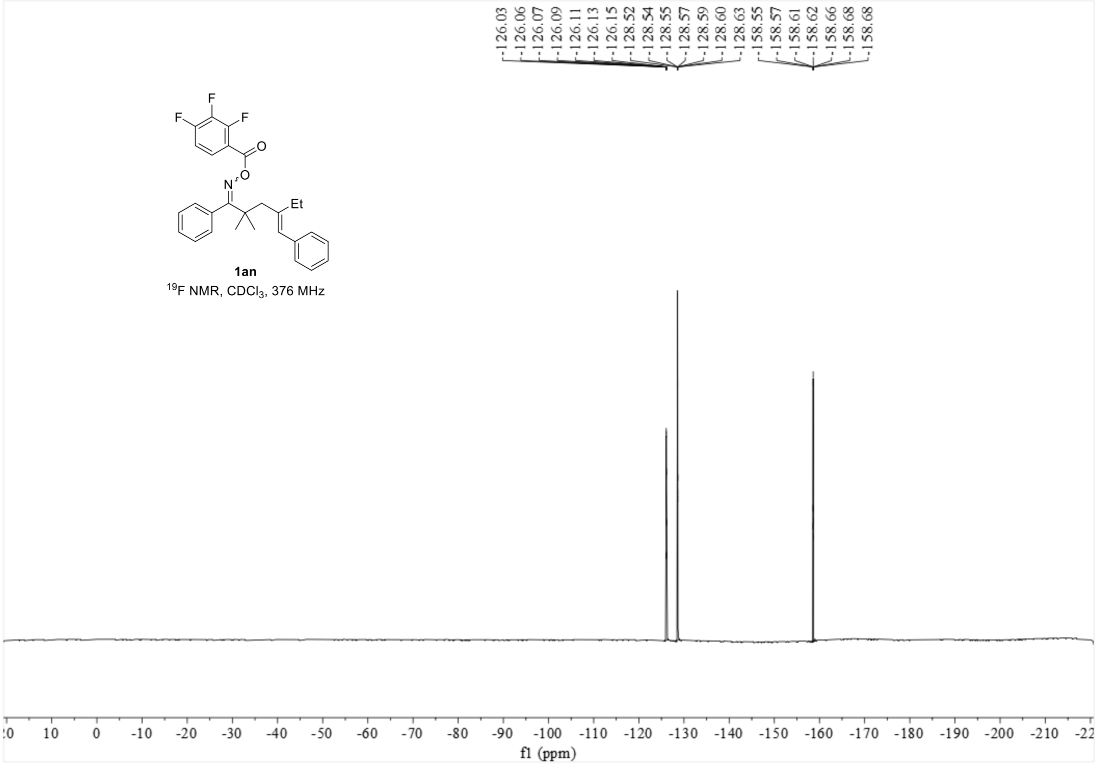
-126.03 -126.06 -126.07 -126.09 -126.11 -126.13 -126.15 -128.52 -128.54 -128.55 -128.57 -128.59 -128.60 -128.63 -158.55 -158.57 -158.61 -158.62 -158.66 -158.68 -158.68
1an
^{19}F NMR, CDCl$_3$, 376 MHz
f1 (ppm)

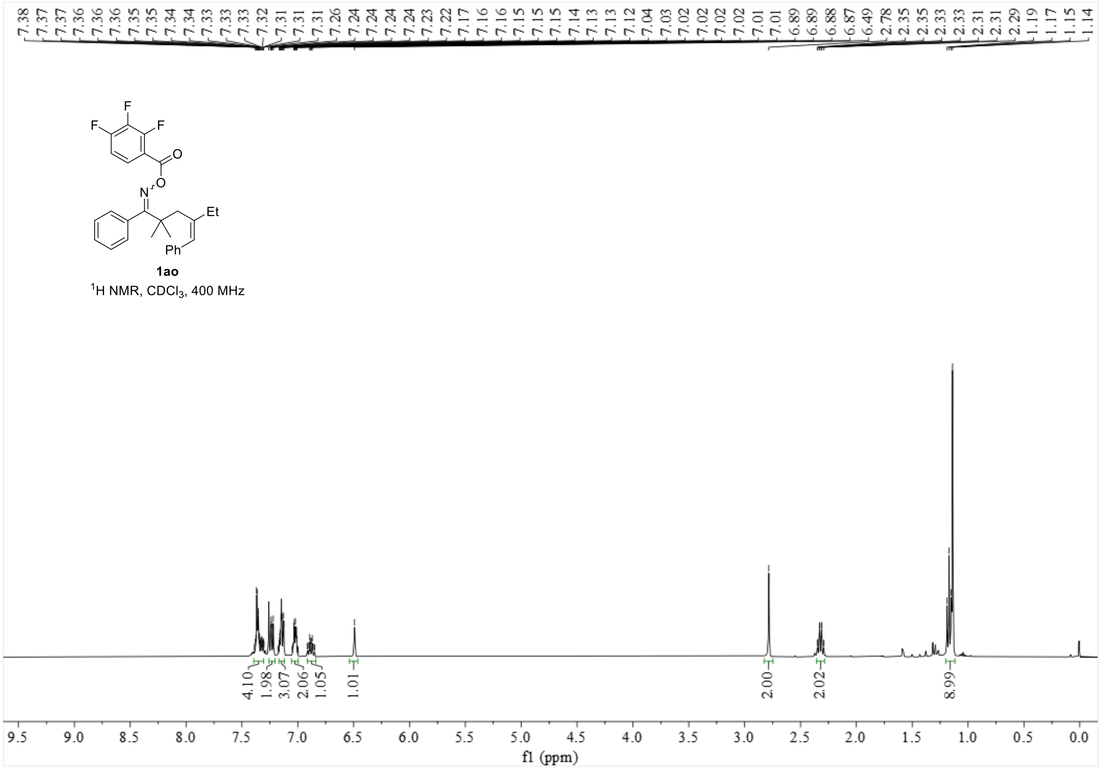
1ao
^{1}H NMR, CDCl3, 400 MHz
f1 (ppm)

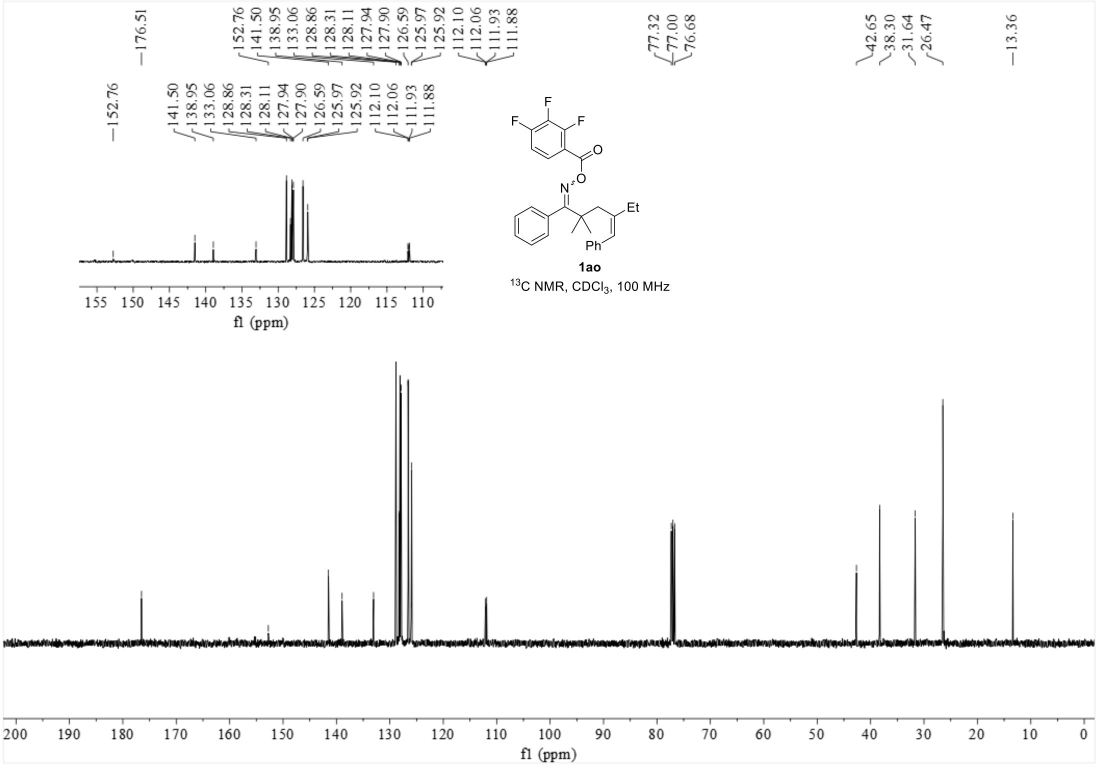
1ao
^{13}C NMR, CDCl3, 100 MHz
f1 (ppm)

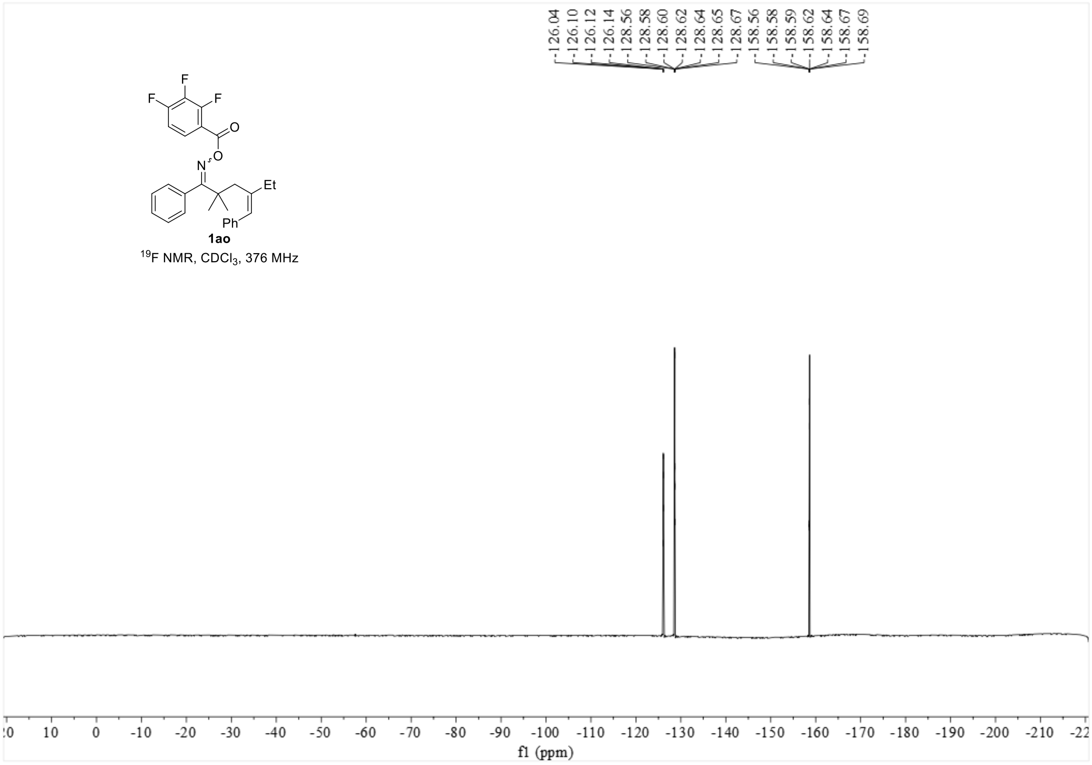
-126.04 -126.10 -126.12 -126.14 -128.56 -128.58 -128.60 -128.62 -128.64 -128.65 -128.67 -158.56 -158.58 -158.59 -158.62 -158.64 -158.67 -158.69
1ao
19F NMR, CDCl3, 376 MHz
f1 (ppm)

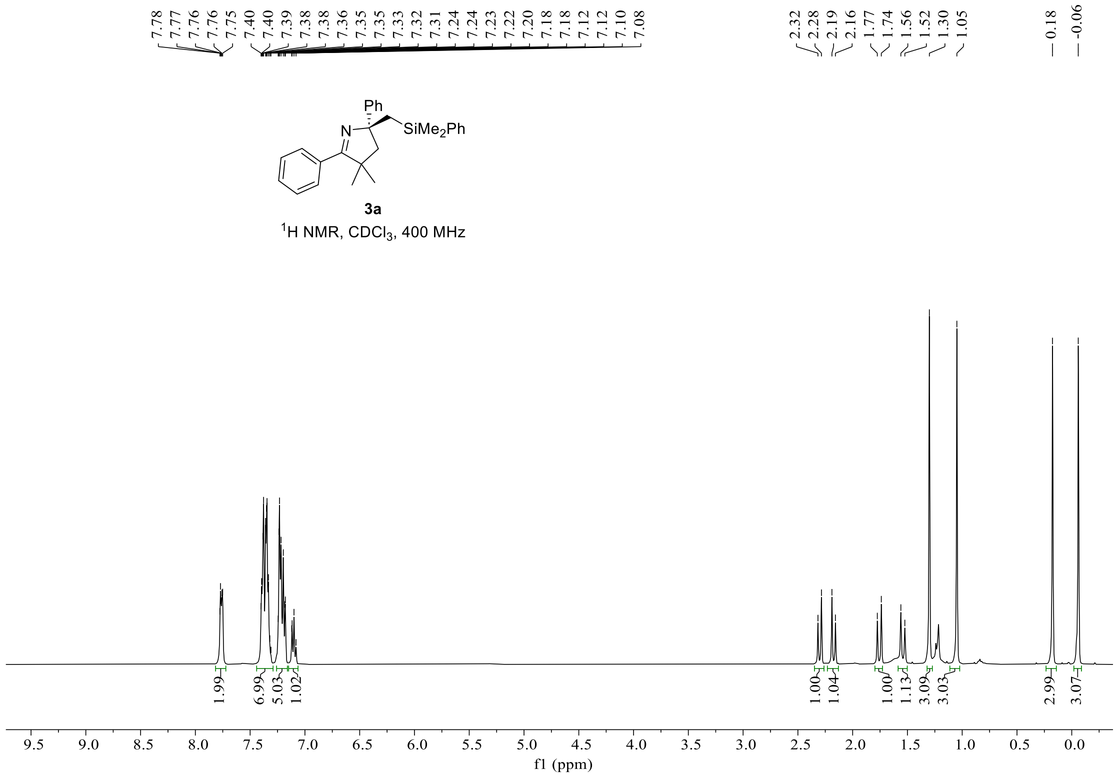
7.78 7.77 7.76 7.76 7.75 7.40 7.40 7.39 7.38 7.38 7.36 7.35 7.35 7.33 7.32 7.31 7.24 7.24 7.23 7.22 7.20 7.18 7.18 7.12 7.12 7.10 7.08
2.32 2.28 2.19 2.16 1.77 1.74 1.56 1.52 1.30 1.05
0.18 -0.06
3a
1H NMR, CDCl3, 400 MHz
1.99 6.99 5.03 1.02 1.00 1.04 1.00 1.13 3.09 3.03 2.99 3.07
f1 (ppm)

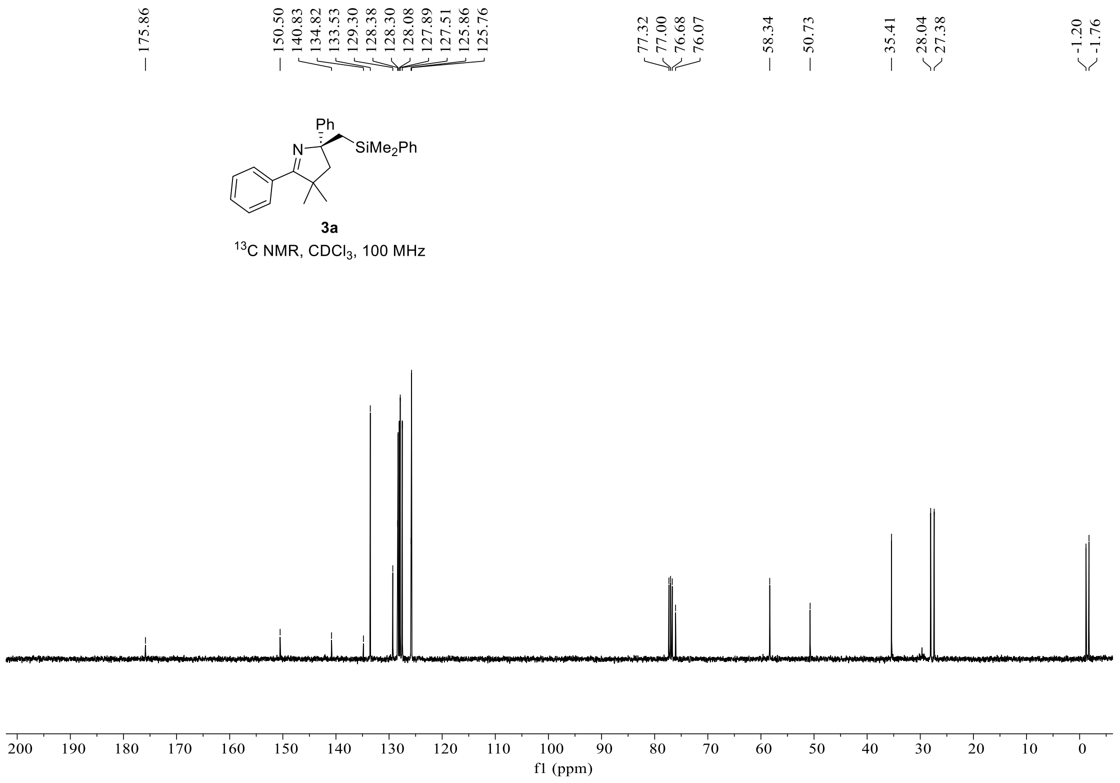

3a

^{13}C NMR, $CDCl_3$, 100 MHz

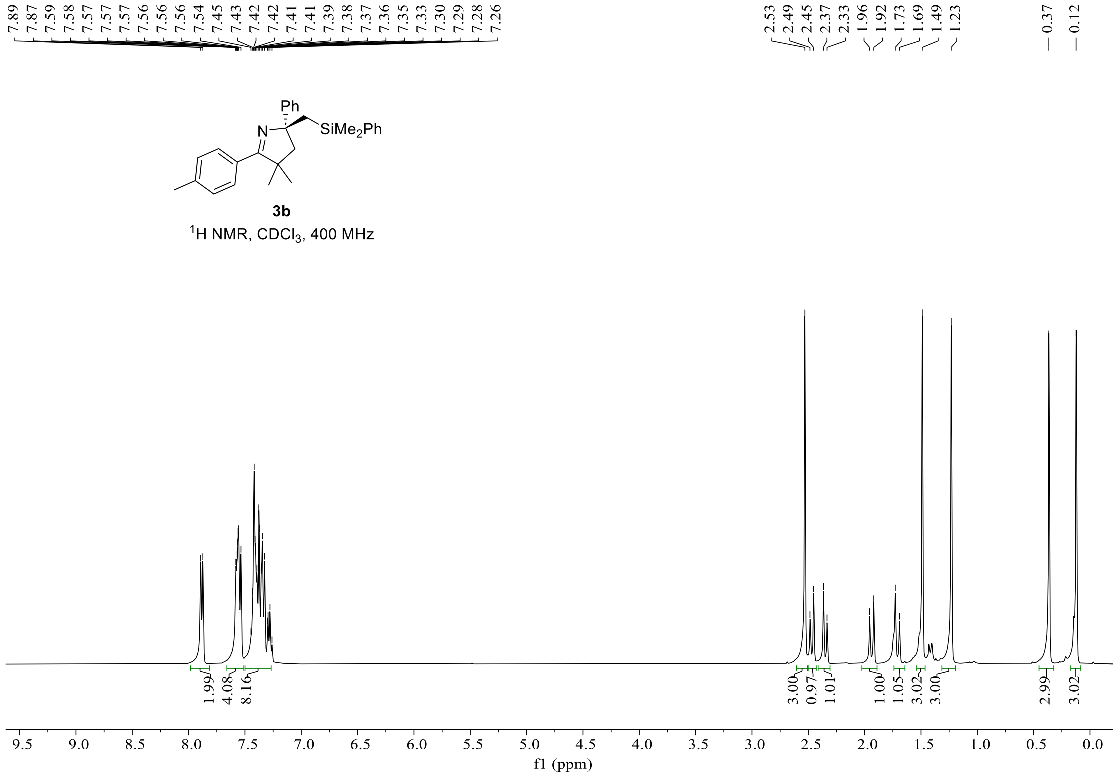

3b

^{1}H NMR, $CDCl_3$, 400 MHz

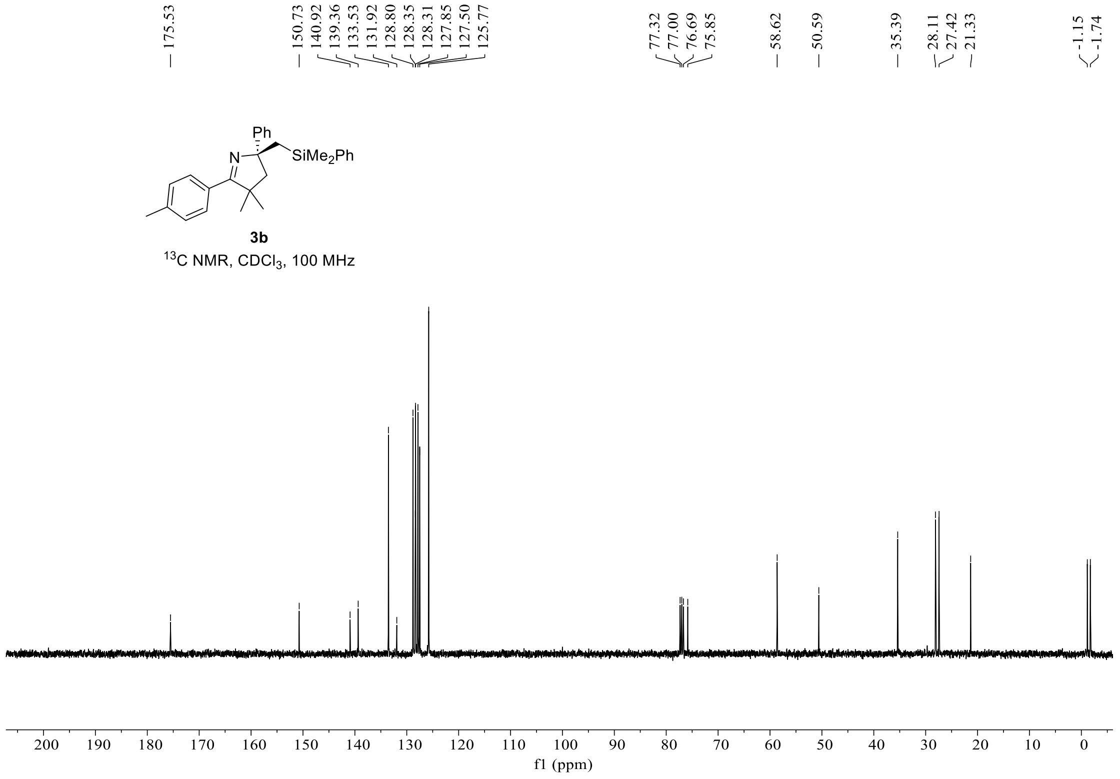

3b

^{13}C NMR, CDCl$_3$, 100 MHz

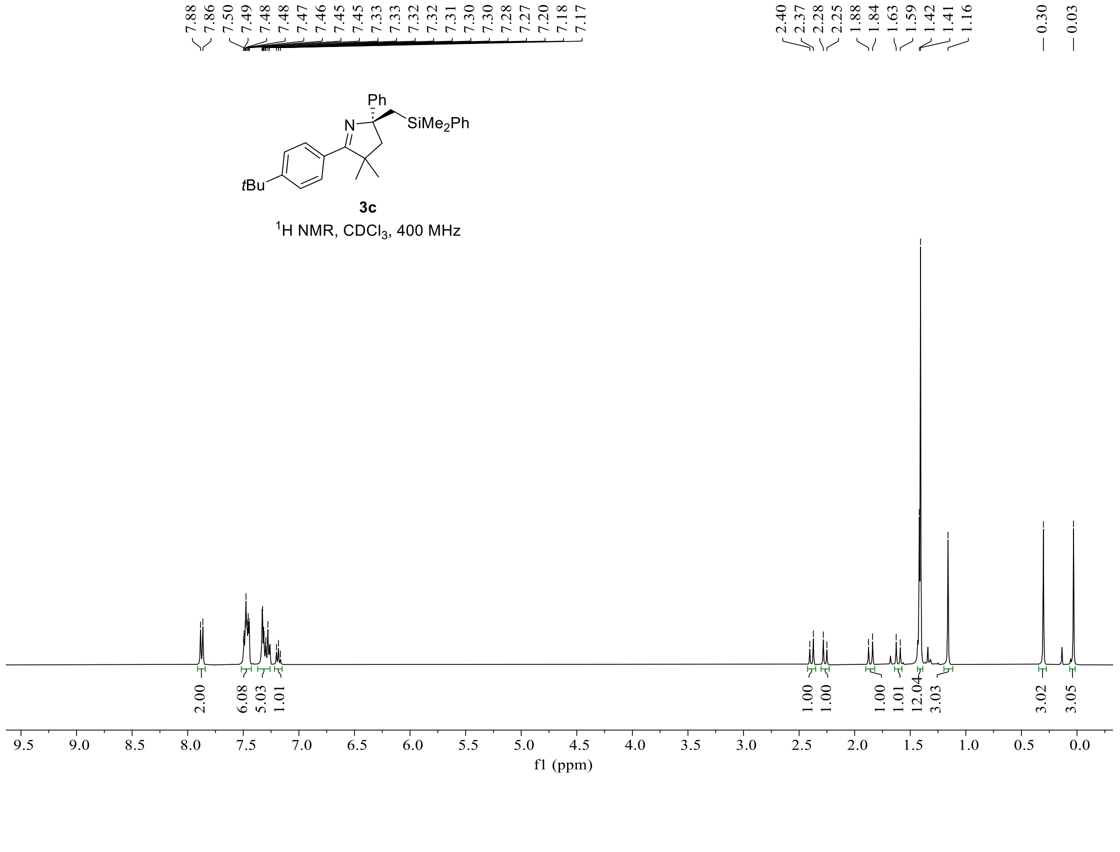

3c

^{1}H NMR, CDCl$_3$, 400 MHz

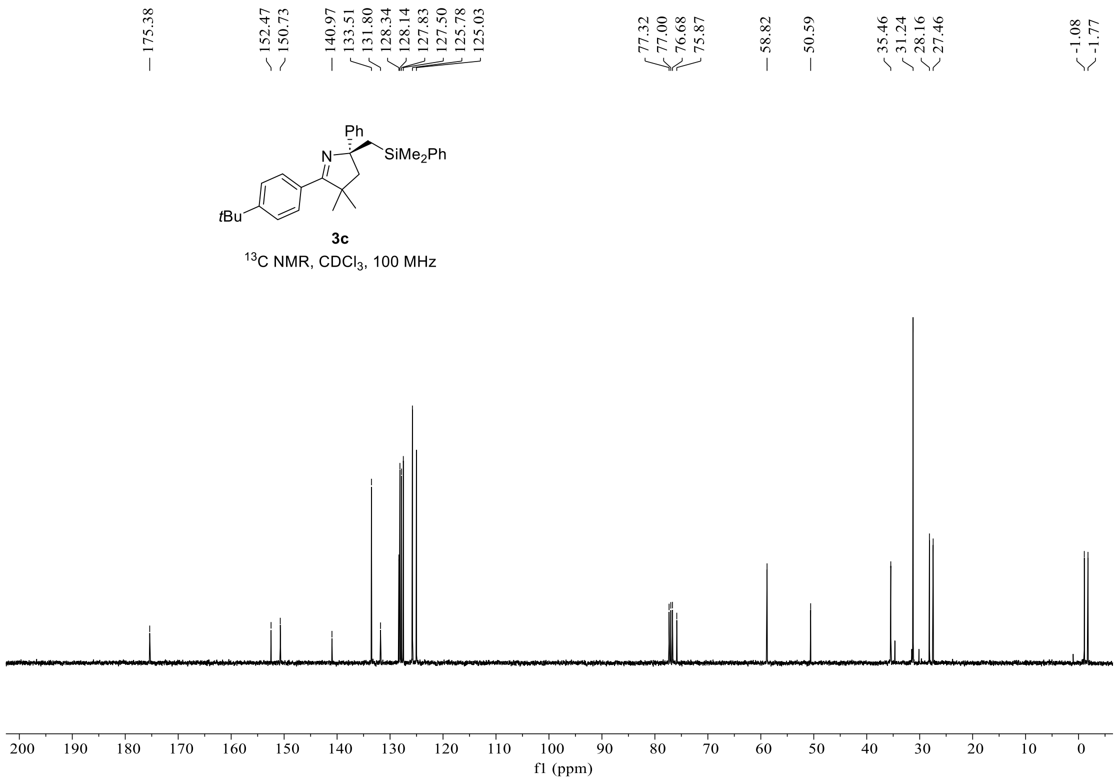
175.38
152.47
150.73
140.97
133.51
131.80
128.34
128.14
127.83
127.50
125.78
125.03
77.32
77.00
76.68
75.87
58.82
50.59
35.46
31.24
28.16
27.46
-1.08
-1.77
Ph
N
SiMe2Ph
tBu
3c
13C NMR, CDCl3, 100 MHz
f1 (ppm)

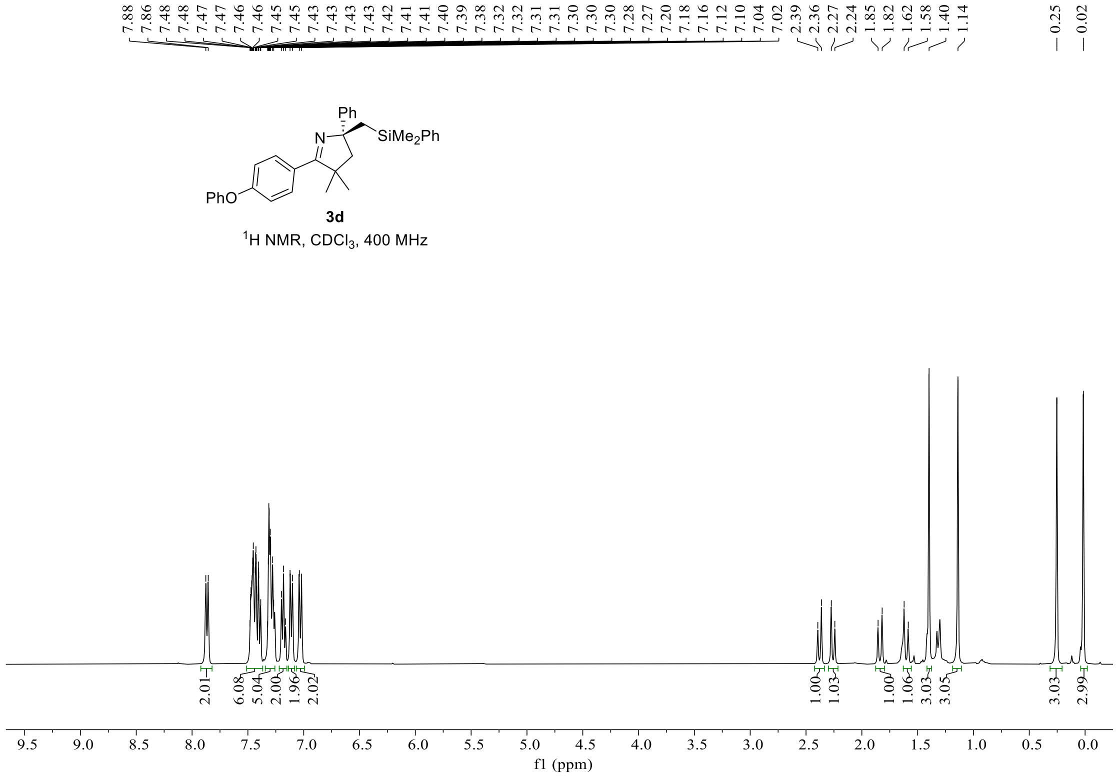
7.88
7.86
7.48
7.48
7.47
7.47
7.46
7.46
7.45
7.45
7.43
7.43
7.43
7.42
7.41
7.41
7.40
7.39
7.38
7.32
7.32
7.31
7.31
7.30
7.30
7.30
7.28
7.27
7.20
7.18
7.16
7.12
7.10
7.04
7.02
2.39
2.36
2.27
2.24
1.85
1.82
1.62
1.58
1.40
1.14
0.25
0.02
Ph
N
SiMe2Ph
PhO
3d
1H NMR, CDCl3, 400 MHz
2.01
6.08
5.04
2.00
1.96
2.02
1.00
1.03
1.00
1.06
3.03
3.05
3.03
2.99
f1 (ppm)

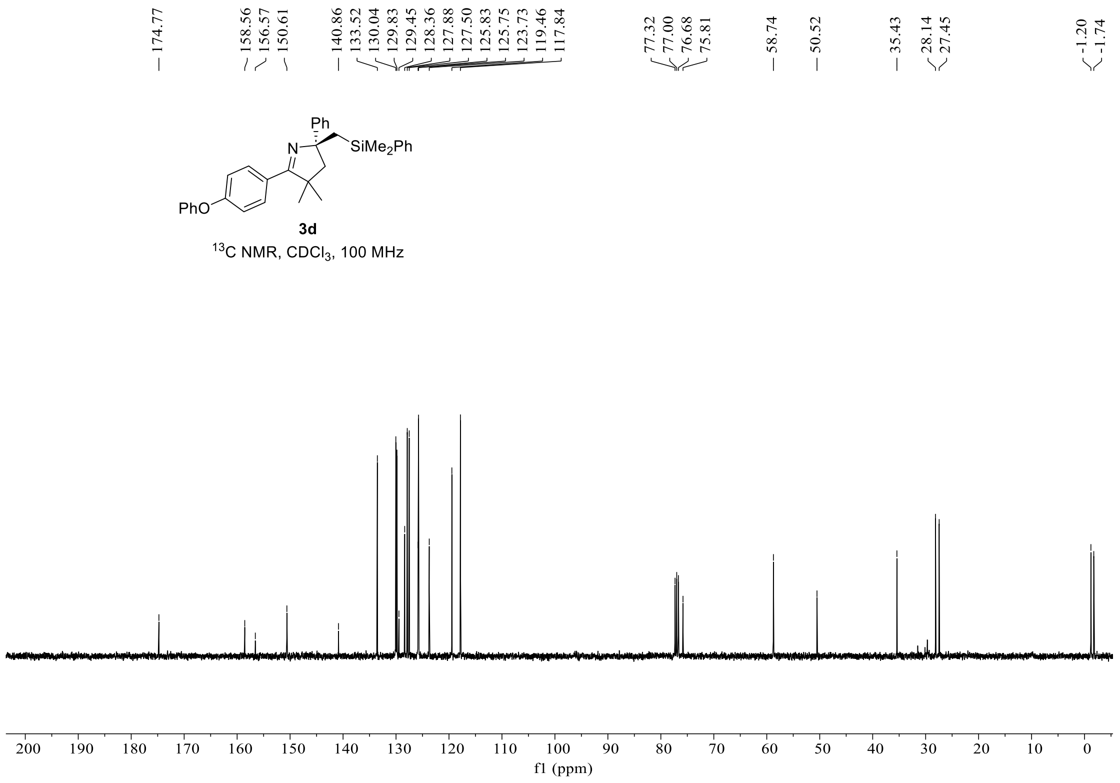
174.77
158.56
156.57
150.61
140.86
133.52
130.04
129.83
129.45
128.36
127.88
127.50
125.83
125.75
123.73
119.46
117.84
77.32
77.00
76.68
75.81
58.74
50.52
35.43
28.14
27.45
-1.20
-1.74
Ph
N
SiMe2Ph
PhO
3d
13C NMR, CDCl3, 100 MHz
200 190 180 170 160 150 140 130 120 110 100 90 80 70 60 50 40 30 20 10 0
f1 (ppm)

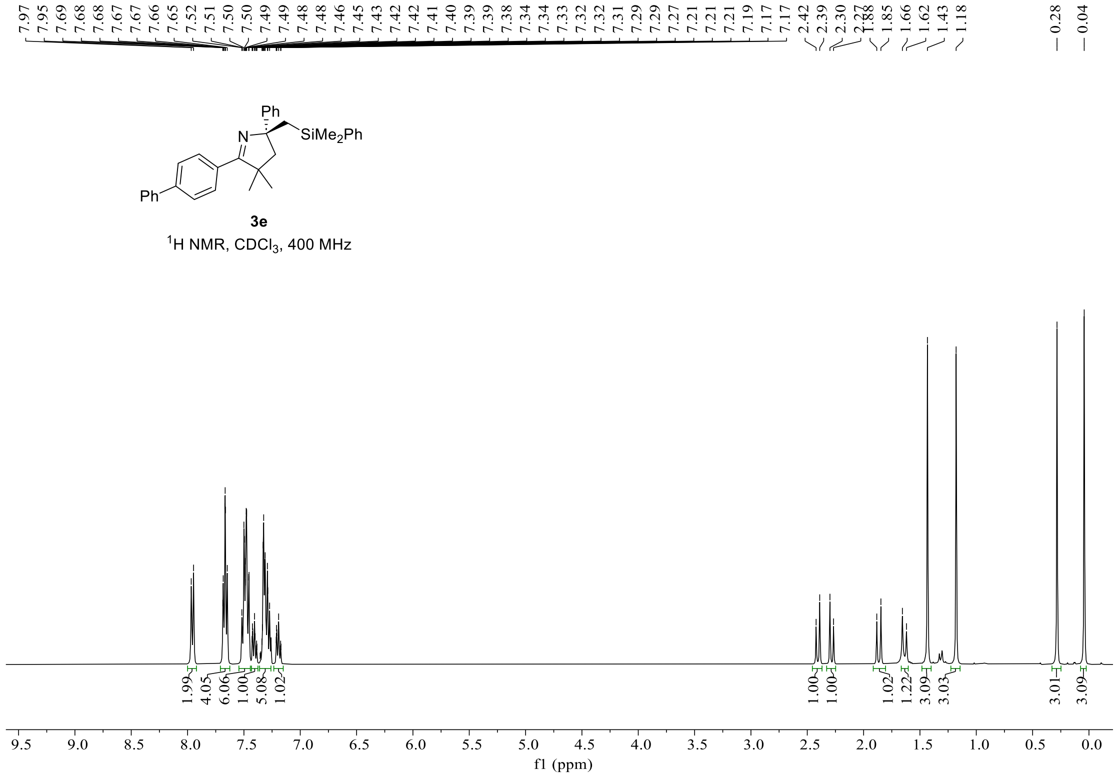
7.97
7.95
7.69
7.68
7.68
7.67
7.67
7.66
7.65
7.52
7.51
7.50
7.50
7.49
7.49
7.48
7.46
7.45
7.43
7.42
7.42
7.41
7.40
7.39
7.39
7.38
7.34
7.34
7.33
7.32
7.32
7.31
7.29
7.29
7.27
7.21
7.21
7.21
7.19
7.17
7.17
2.42
2.39
2.30
2.27
1.88
1.85
1.66
1.62
1.43
1.18
0.28
0.04
Ph
N
SiMe2Ph
Ph
3e
1H NMR, CDCl3, 400 MHz
1.99
4.05
6.06
1.00
5.08
1.02
1.00
1.00
1.02
1.22
3.09
3.03
3.01
3.09
9.5 9.0 8.5 8.0 7.5 7.0 6.5 6.0 5.5 5.0 4.5 4.0 3.5 3.0 2.5 2.0 1.5 1.0 0.5 0.0
f1 (ppm)

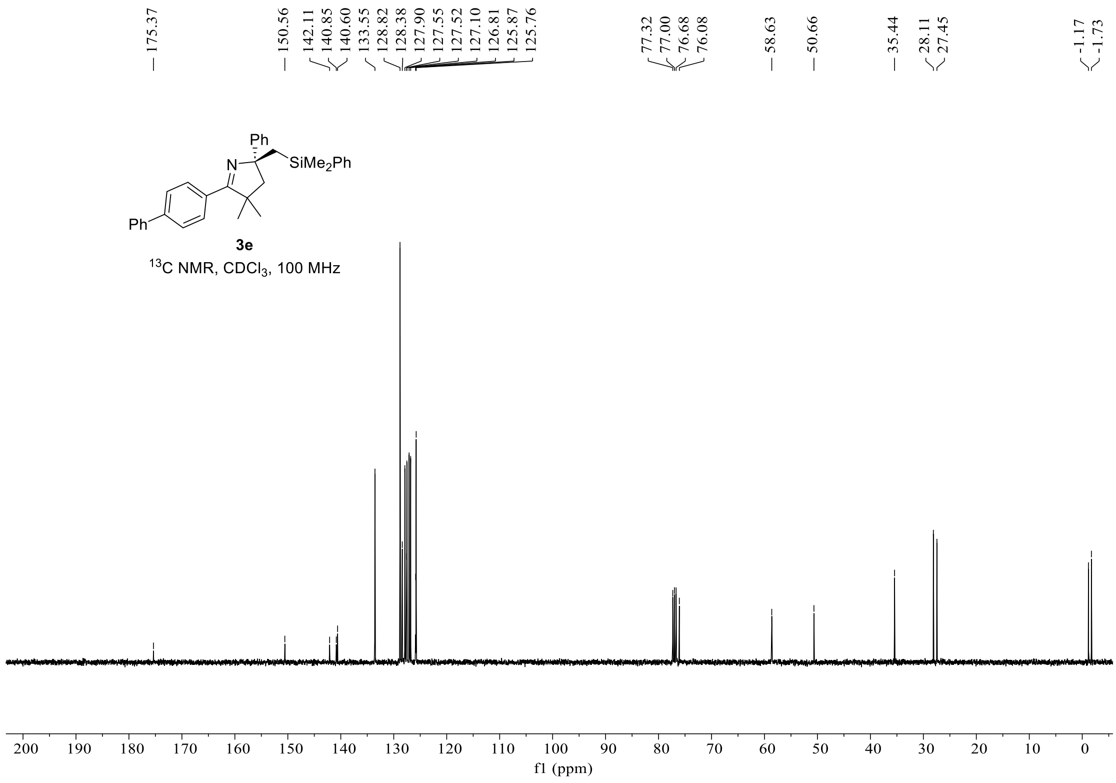

3e

^{13}C NMR, $CDCl_3$, 100 MHz

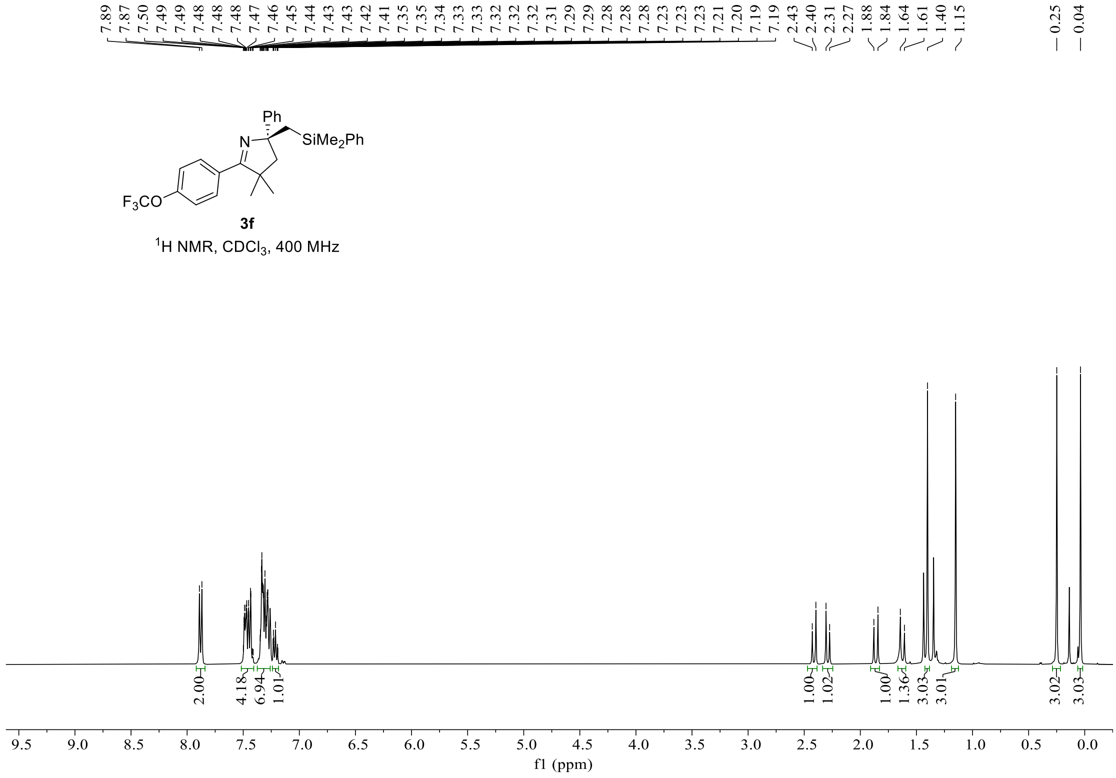

3f

^{1}H NMR, $CDCl_3$, 400 MHz

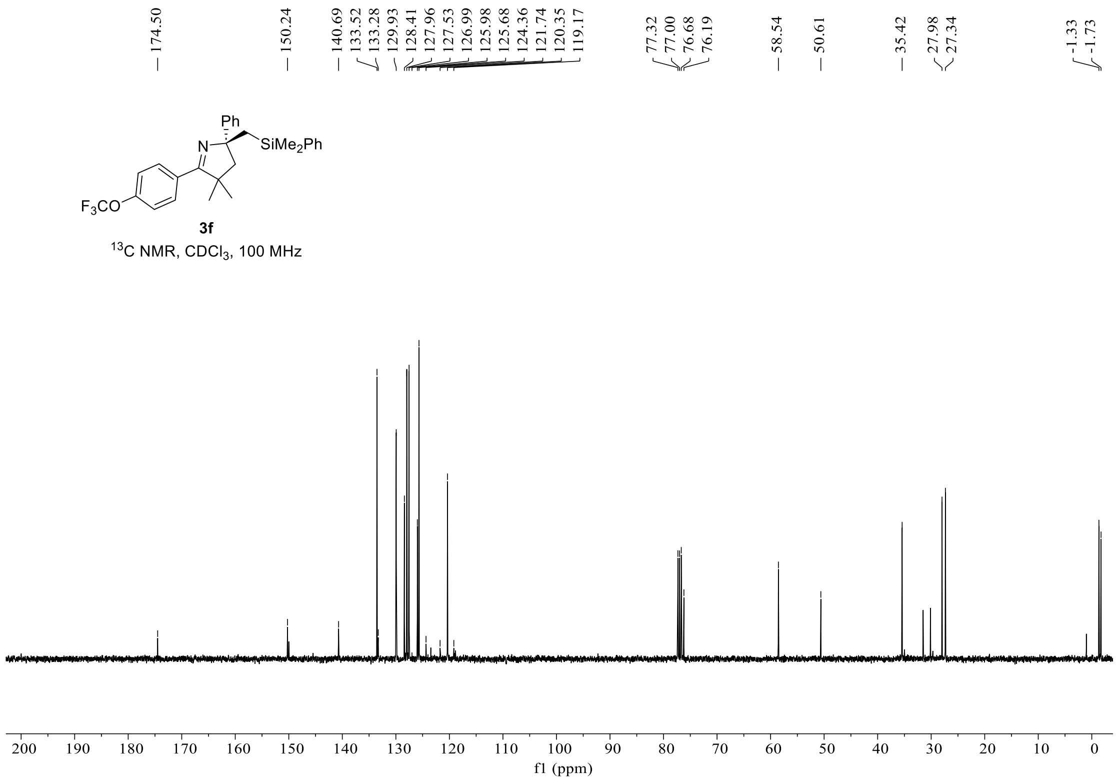
174.50
150.24
140.69
133.52
133.28
129.93
128.41
127.96
127.53
126.99
125.98
125.68
124.36
121.74
120.35
119.17
77.32
77.00
76.68
76.19
58.54
50.61
35.42
27.98
27.34
-1.33
-1.73
Ph
N
SiMe2Ph
F3CO
3f
13C NMR, CDCl3, 100 MHz
200 190 180 170 160 150 140 130 120 110 100 90 80 70 60 50 40 30 20 10 0
f1 (ppm)

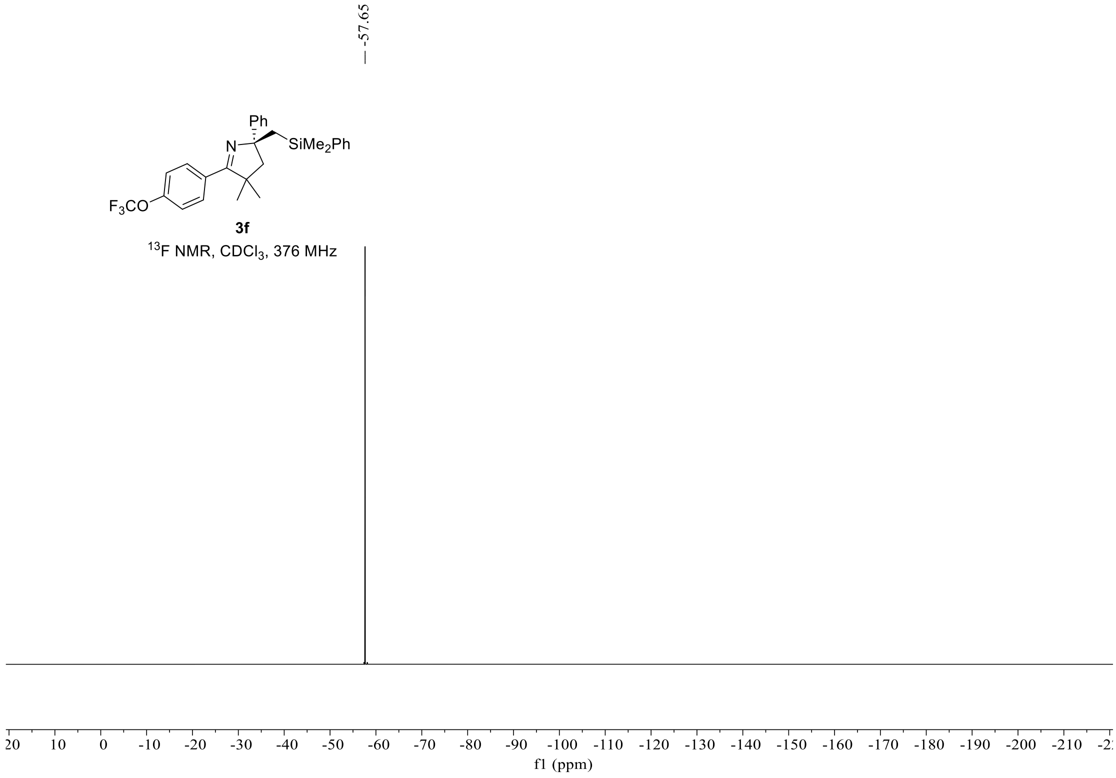
-57.65
Ph
N
SiMe2Ph
F3CO
3f
13F NMR, CDCl3, 376 MHz
20 10 0 -10 -20 -30 -40 -50 -60 -70 -80 -90 -100 -110 -120 -130 -140 -150 -160 -170 -180 -190 -200 -210
f1 (ppm)

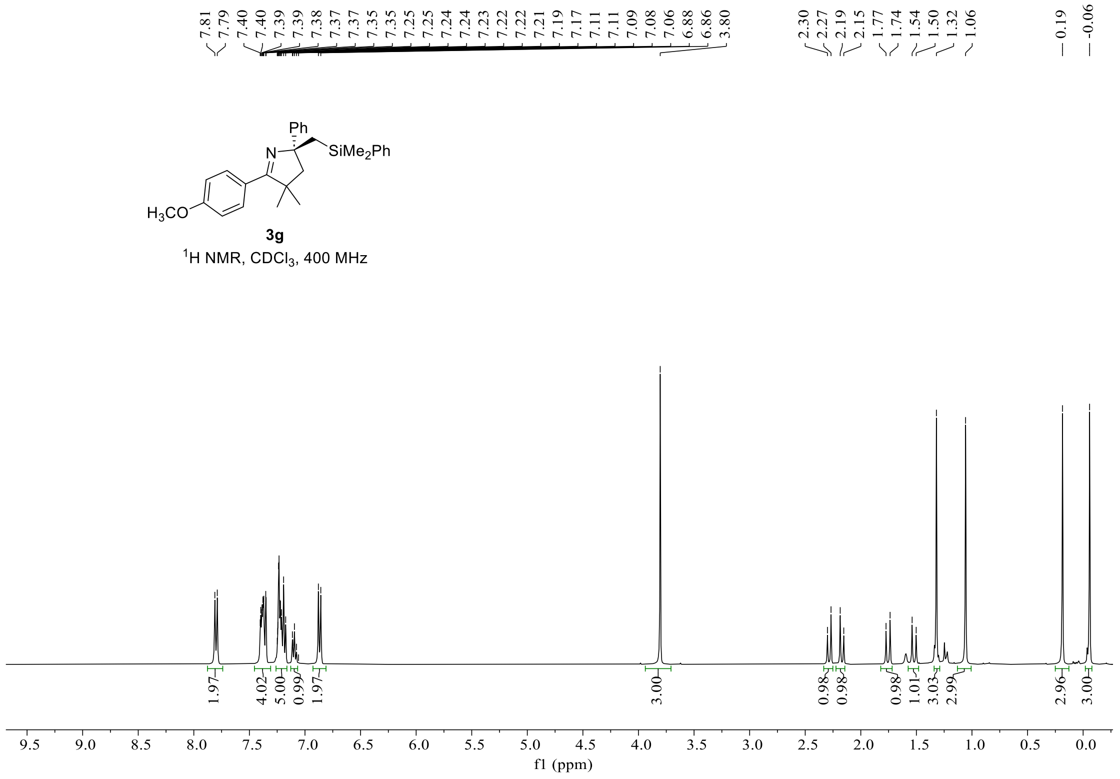

3g
^{1}H NMR, $CDCl_3$, 400 MHz

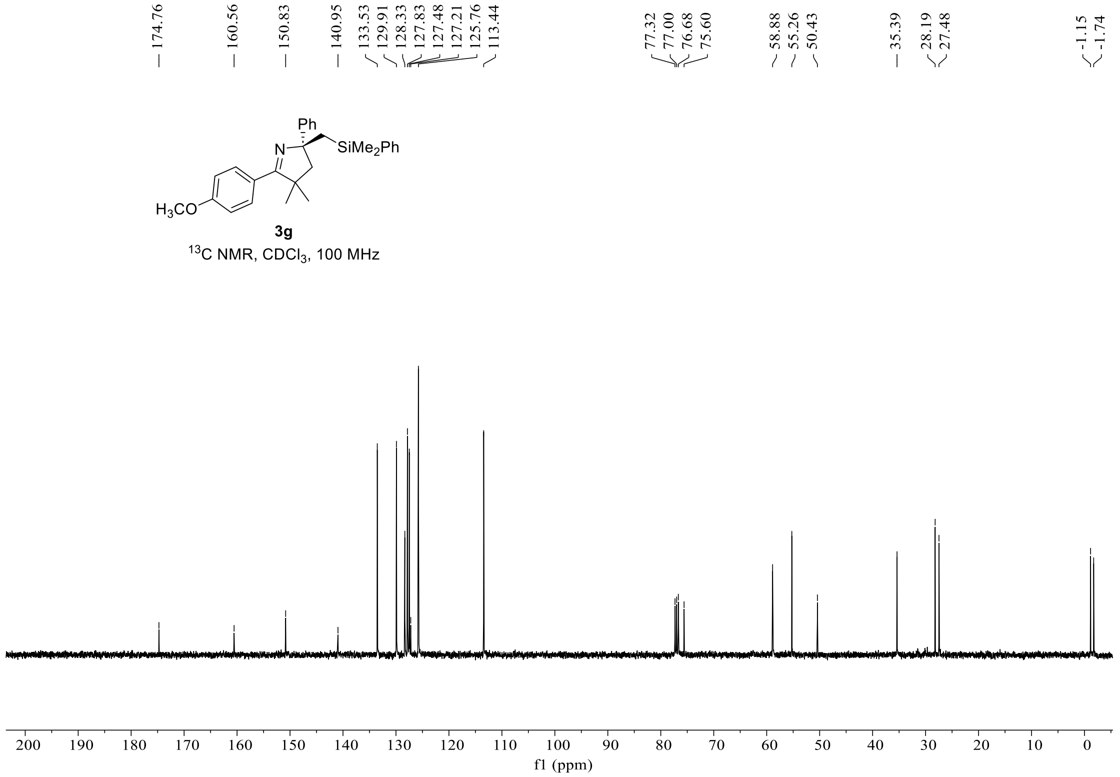

3g
^{13}C NMR, $CDCl_3$, 100 MHz

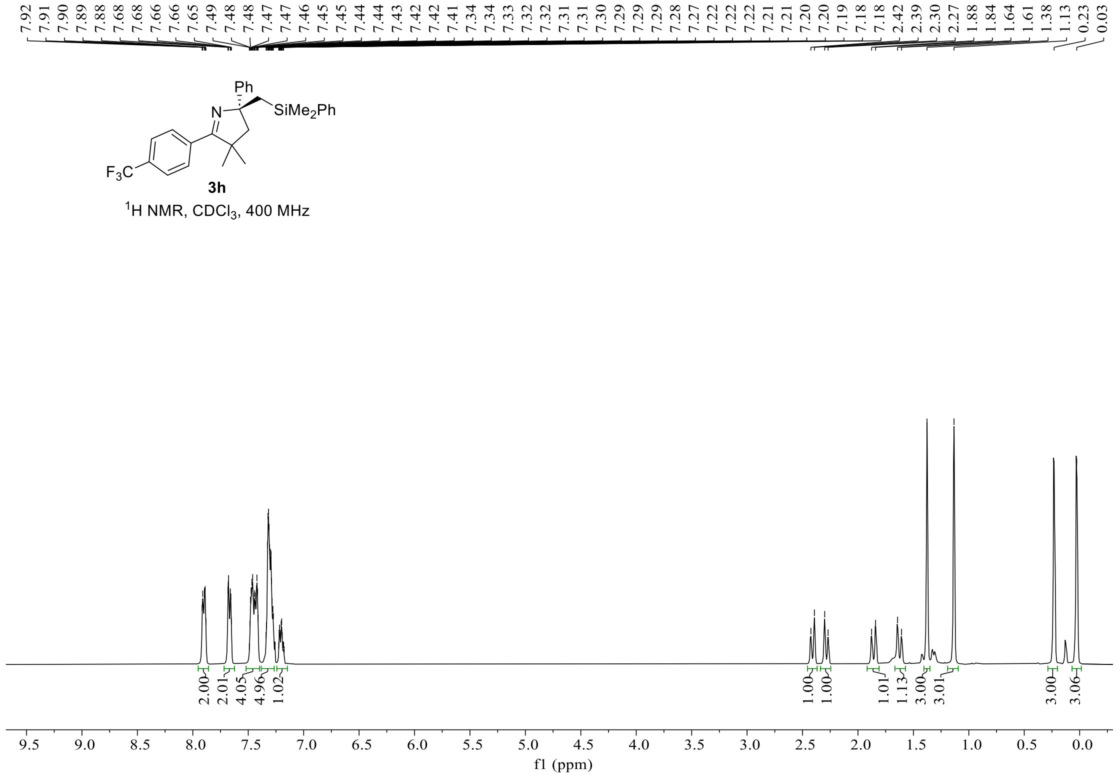
7.92 7.91 7.90 7.89 7.88 7.68 7.68 7.66 7.66 7.65 7.49 7.48 7.48 7.47 7.47 7.46 7.45 7.45 7.44 7.44 7.43 7.42 7.42 7.41 7.34 7.34 7.33 7.32 7.32 7.31 7.31 7.30 7.29 7.29 7.29 7.28 7.27 7.22 7.22 7.22 7.21 7.21 7.20 7.20 7.19 7.18 7.18 2.42 2.39 2.30 2.27 1.88 1.84 1.64 1.61 1.38 1.13 0.23 0.03
Ph
N
SiMe2Ph
F3C
3h
1H NMR, CDCl3, 400 MHz
2.00 2.01 4.05 4.96 1.02 1.00 1.00 1.01 1.13 3.00 3.01 3.00 3.06
9.5 9.0 8.5 8.0 7.5 7.0 6.5 6.0 5.5 5.0 4.5 4.0 3.5 3.0 2.5 2.0 1.5 1.0 0.5 0.0
f1 (ppm)

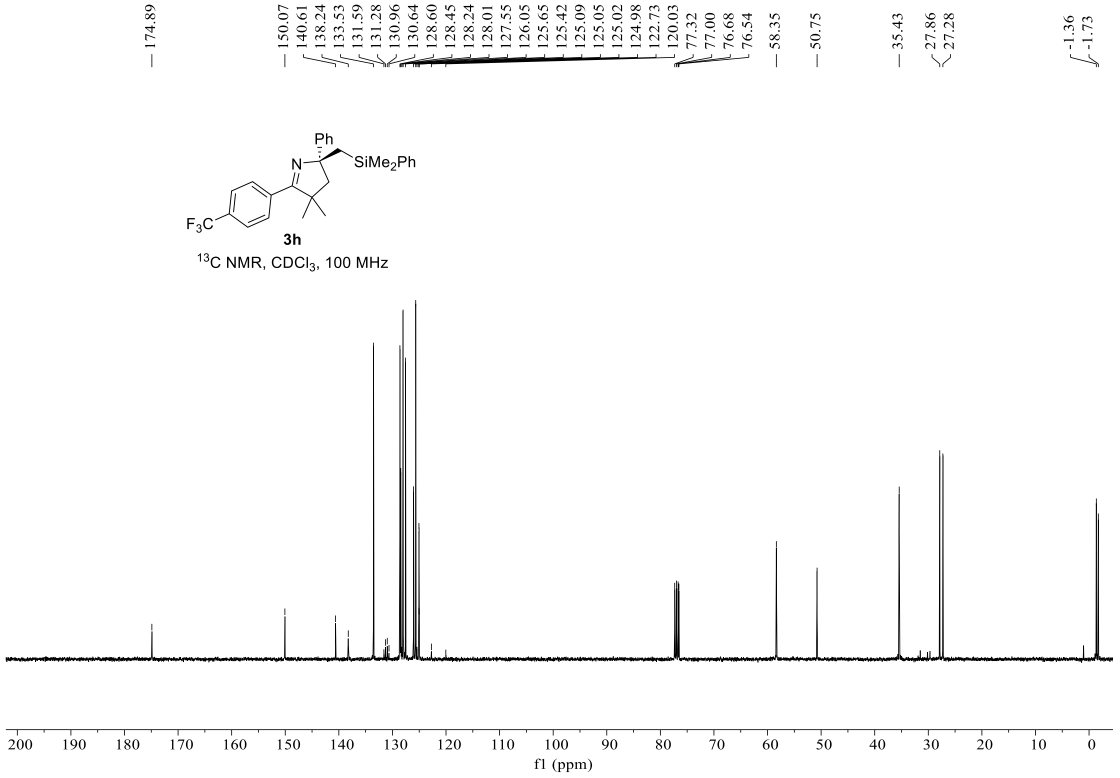
174.89 150.07 140.61 138.24 133.53 131.59 131.28 130.96 130.64 128.60 128.45 128.24 128.01 127.55 126.05 125.65 125.42 125.09 125.05 125.02 124.98 122.73 120.03 77.32 77.00 76.68 76.54 58.35 50.75 35.43 27.86 27.28 -1.36 -1.73
Ph
N
SiMe2Ph
F3C
3h
13C NMR, CDCl3, 100 MHz
200 190 180 170 160 150 140 130 120 110 100 90 80 70 60 50 40 30 20 10 0
f1 (ppm)

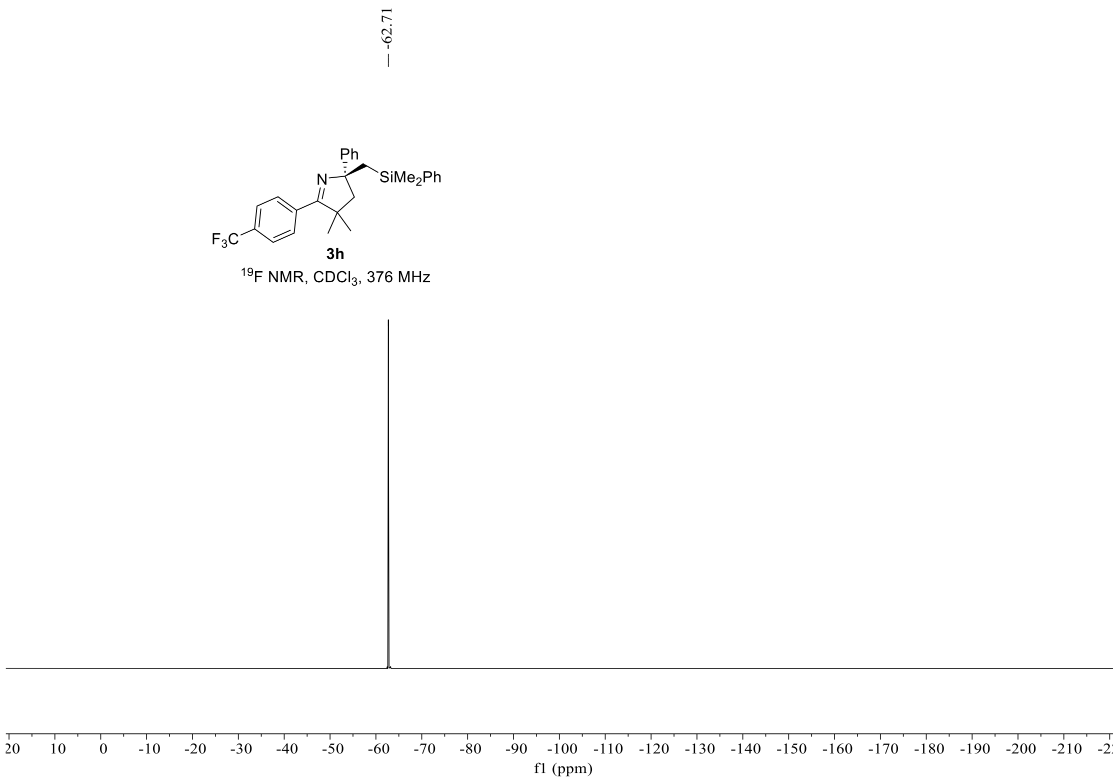

3h

^{19}F NMR, $CDCl_3$, 376 MHz

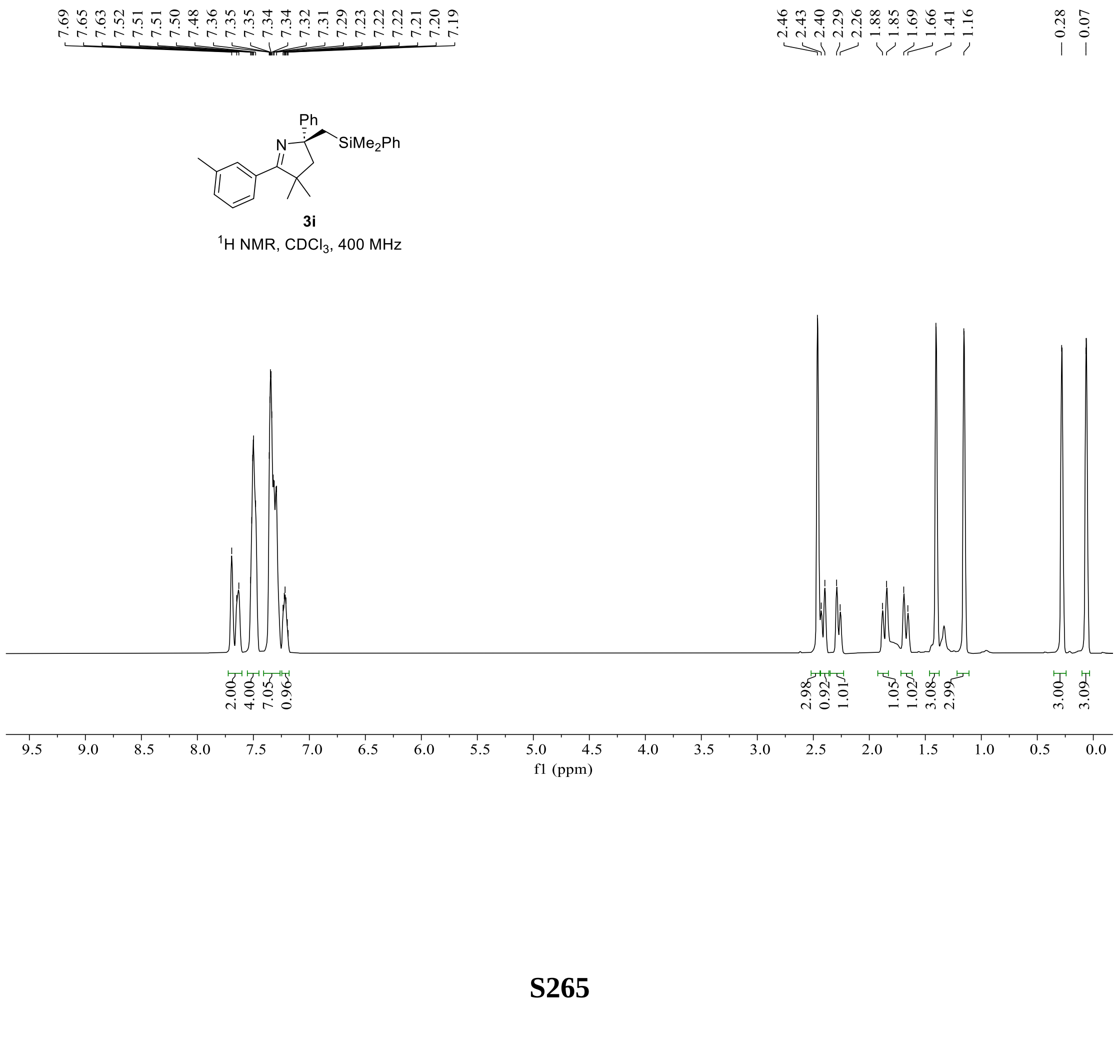

3i

^{1}H NMR, $CDCl_3$, 400 MHz

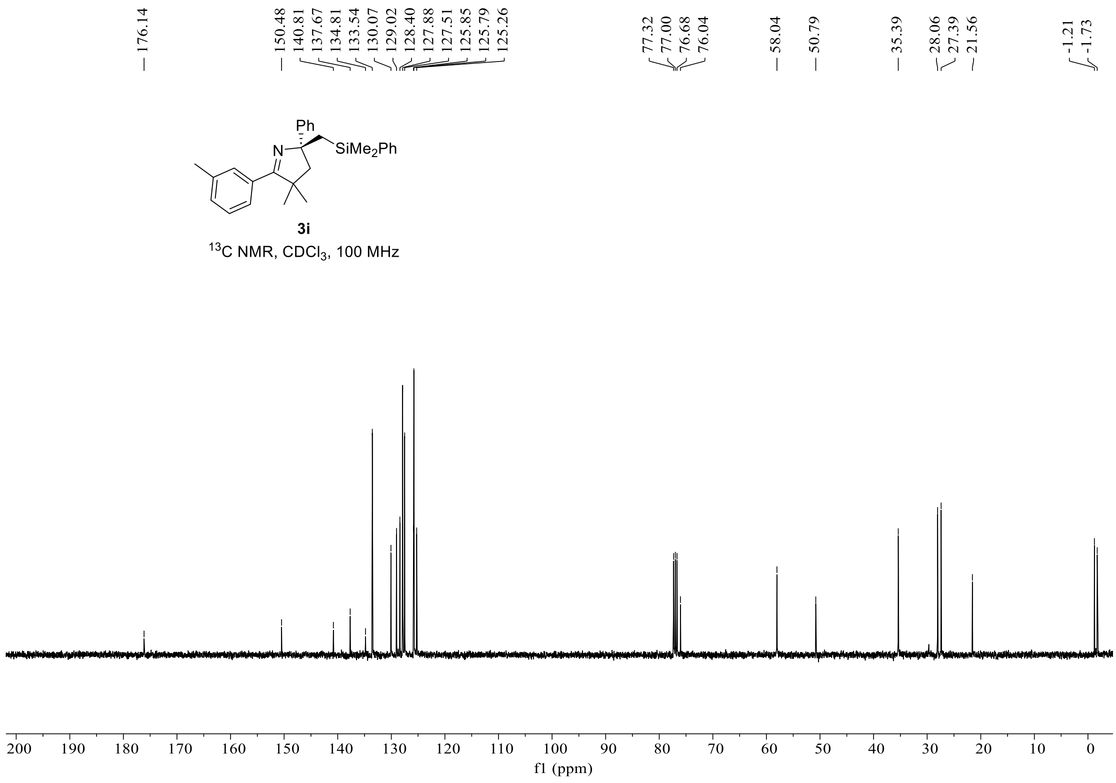
176.14
150.48
140.81
137.67
134.81
133.54
130.07
129.02
128.40
127.88
127.51
125.85
125.79
125.26
77.32
77.00
76.68
76.04
58.04
50.79
35.39
28.06
27.39
21.56
-1.21
-1.73
Ph
N
SiMe2Ph
3i
13C NMR, CDCl3, 100 MHz
f1 (ppm)

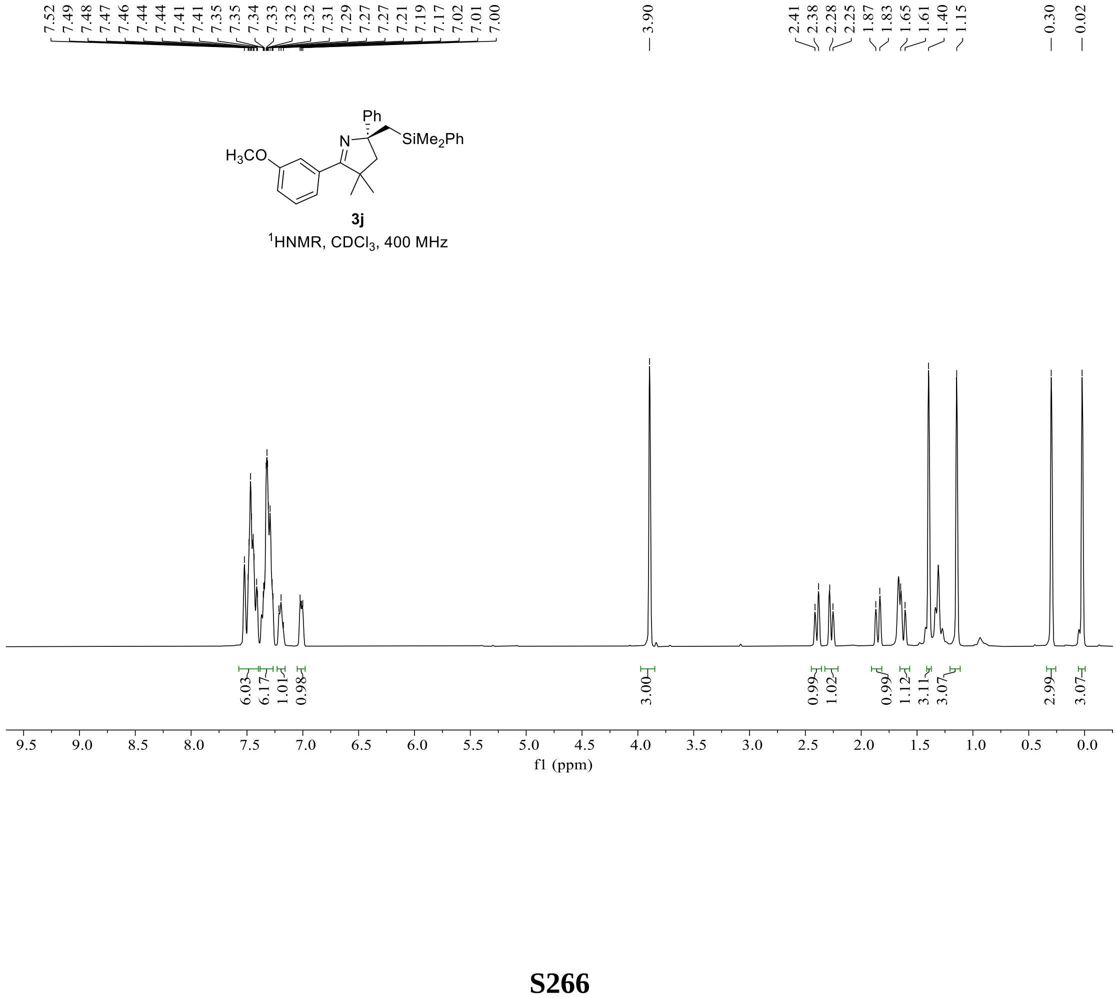
7.52
7.49
7.48
7.47
7.46
7.44
7.44
7.41
7.41
7.35
7.35
7.34
7.33
7.32
7.32
7.31
7.29
7.27
7.27
7.21
7.19
7.17
7.02
7.01
7.00
3.90
2.41
2.38
2.28
2.25
1.87
1.83
1.65
1.61
1.40
1.15
0.30
0.02
Ph
N
SiMe2Ph
H3CO
3j
1HNMR, CDCl3, 400 MHz
6.03
6.17
1.01
0.98
3.00
0.99
1.02
0.99
1.12
3.11
3.07
2.99
3.07
f1 (ppm)

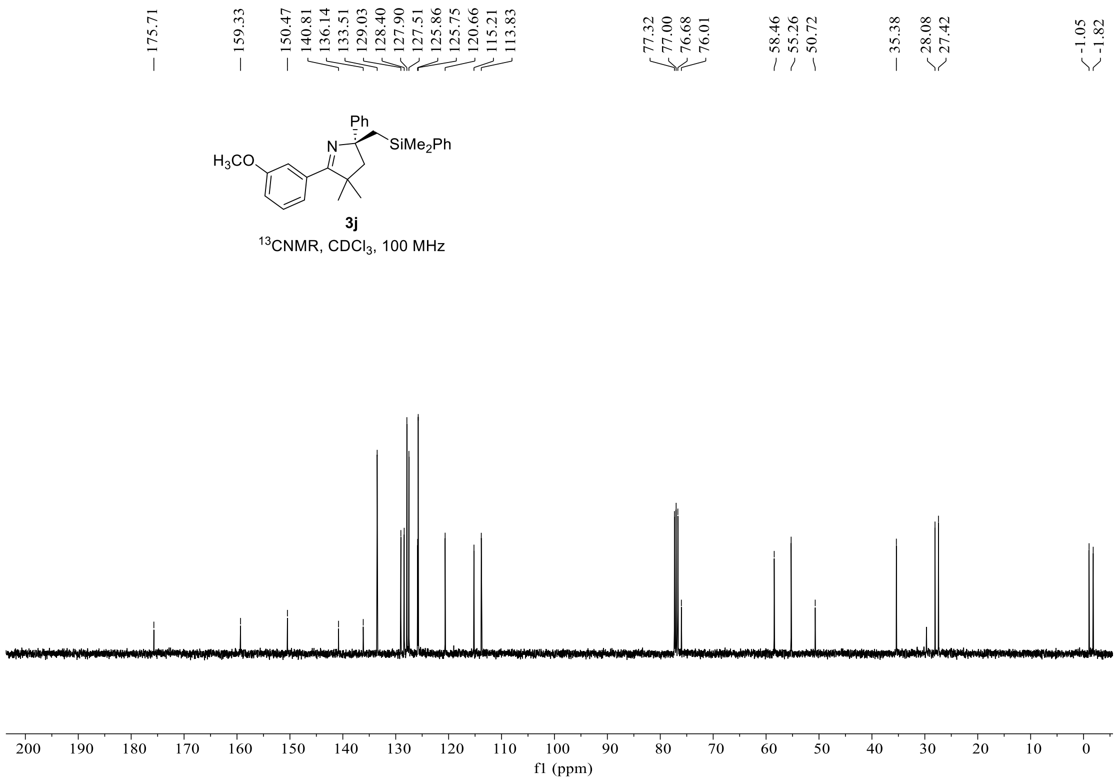
175.71
159.33
150.47
140.81
136.14
133.51
129.03
128.40
127.90
127.51
125.86
125.75
120.66
115.21
113.83
77.32
77.00
76.68
76.01
58.46
55.26
50.72
35.38
28.08
27.42
-1.05
-1.82
Ph
N
SiMe2Ph
H3CO
3j
13CNMR, CDCl3, 100 MHz
200 190 180 170 160 150 140 130 120 110 100 90 80 70 60 50 40 30 20 10 0
f1 (ppm)

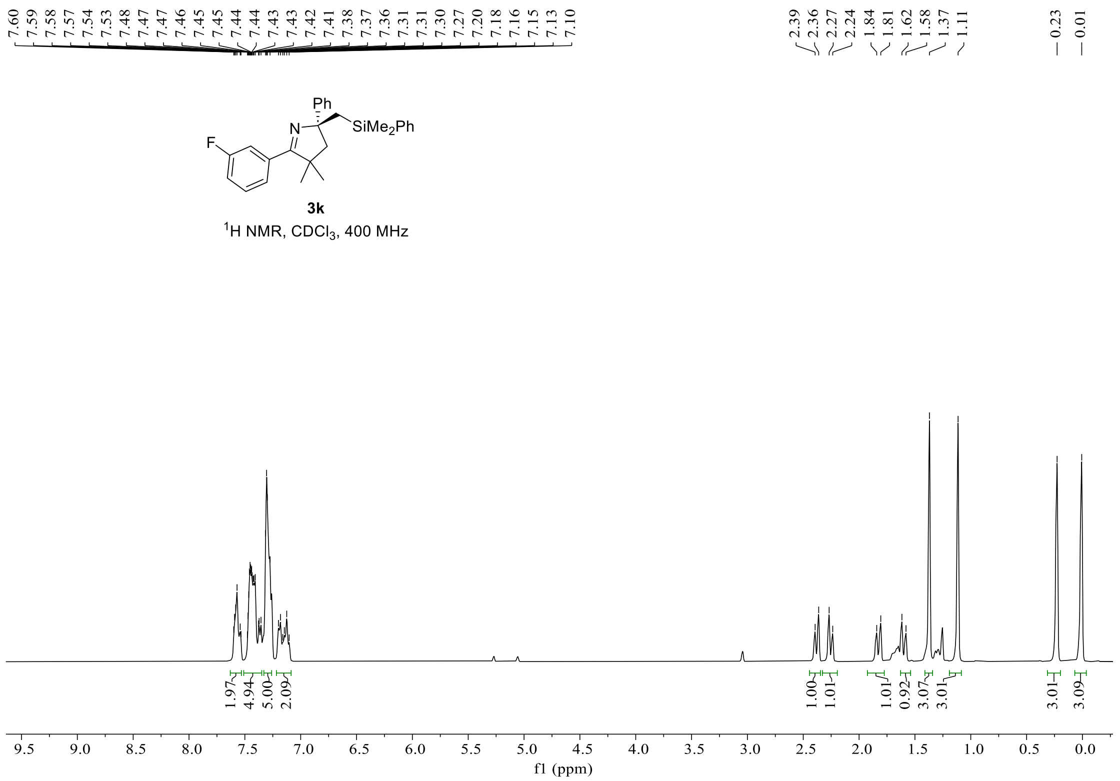
7.60
7.59
7.58
7.57
7.54
7.53
7.48
7.47
7.47
7.46
7.45
7.45
7.44
7.44
7.43
7.43
7.42
7.41
7.38
7.37
7.36
7.31
7.31
7.30
7.27
7.20
7.18
7.16
7.15
7.13
7.10
2.39
2.36
2.27
2.24
1.84
1.81
1.62
1.58
1.37
1.11
0.23
0.01
Ph
N
SiMe2Ph
F
3k
1H NMR, CDCl3, 400 MHz
1.97
4.94
5.00
2.09
1.00
1.01
1.01
0.92
3.07
3.01
3.01
3.09
9.5 9.0 8.5 8.0 7.5 7.0 6.5 6.0 5.5 5.0 4.5 4.0 3.5 3.0 2.5 2.0 1.5 1.0 0.5 0.0
f1 (ppm)

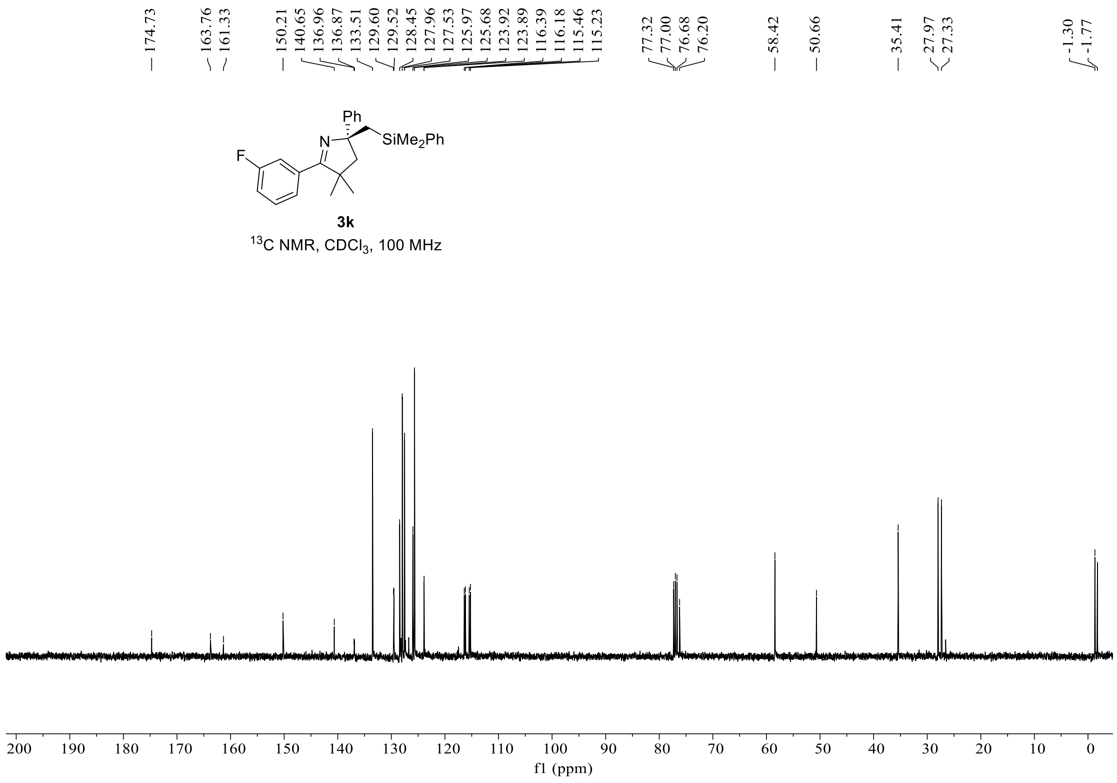

3k

^{13}C NMR, $CDCl_3$, 100 MHz

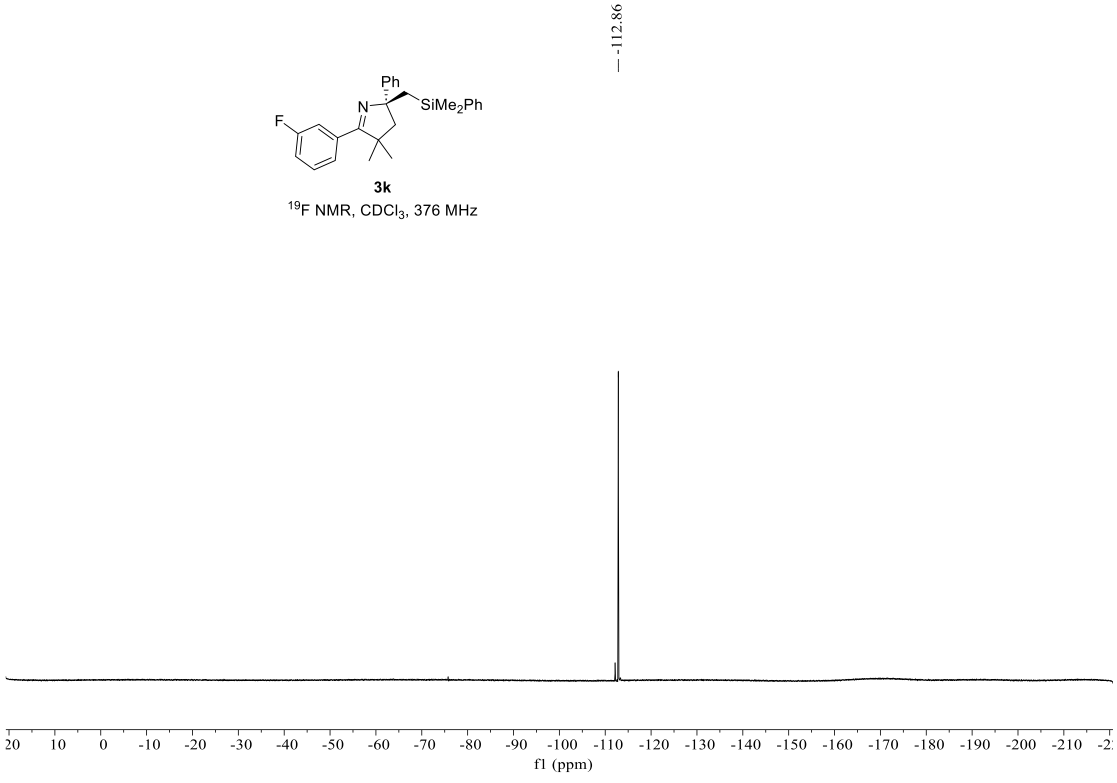

3k

^{19}F NMR, $CDCl_3$, 376 MHz

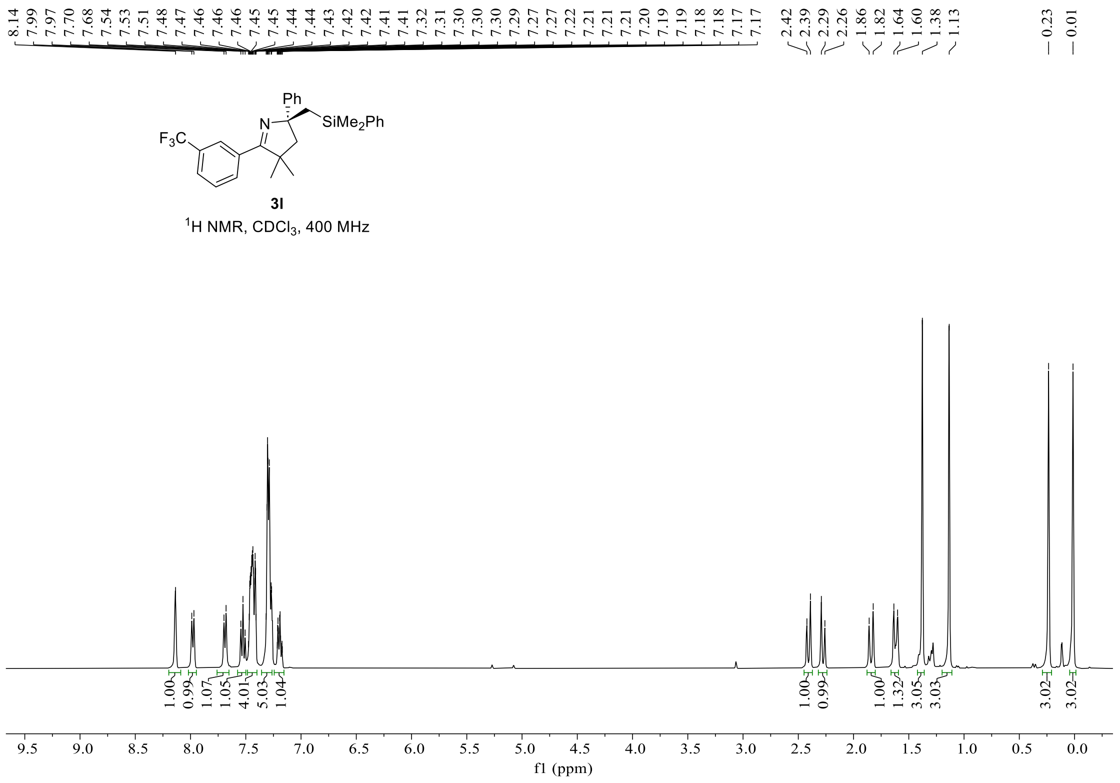
Ph
N
SiMe2Ph
F3C
3l
1H NMR, CDCl3, 400 MHz
8.14 7.99 7.97 7.70 7.68 7.54 7.53 7.51 7.48 7.47 7.46 7.46 7.46 7.45 7.45 7.44 7.44 7.43 7.42 7.42 7.41 7.41 7.32 7.31 7.30 7.30 7.30 7.29 7.27 7.27 7.22 7.21 7.21 7.21 7.20 7.19 7.19 7.18 7.18 7.17 7.17
2.42 2.39 2.29 2.26 1.86 1.82 1.64 1.60 1.38 1.13
0.23 0.01
1.00 0.99 1.07 1.05 4.01 5.03 1.04
1.00 0.99 1.00 1.32 3.05 3.03 3.02 3.02
9.5 9.0 8.5 8.0 7.5 7.0 6.5 6.0 5.5 5.0 4.5 4.0 3.5 3.0 2.5 2.0 1.5 1.0 0.5 0.0
f1 (ppm)

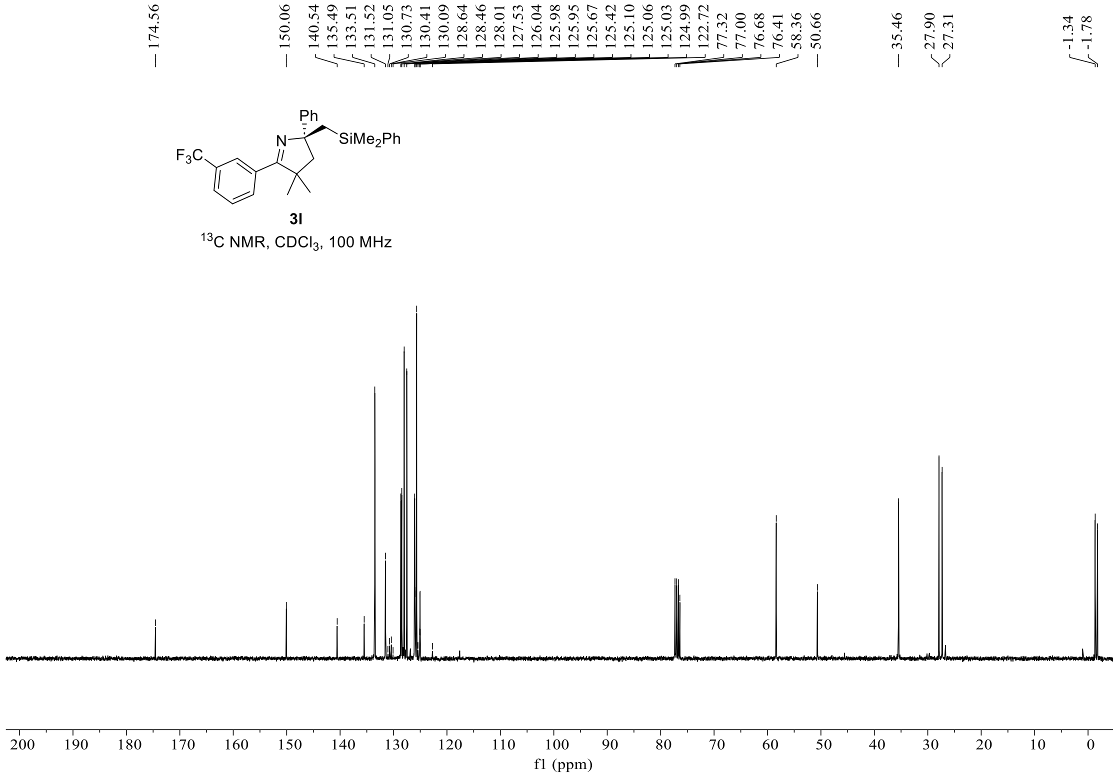
Ph
N
SiMe2Ph
F3C
3l
13C NMR, CDCl3, 100 MHz
174.56
150.06 140.54 135.49 133.51 131.52 131.05 130.73 130.41 130.09 128.64 128.46 128.01 127.53 126.04 125.98 125.95 125.67 125.42 125.10 125.06 125.03 124.99 122.72 77.32 77.00 76.68 76.41 58.36 50.66
35.46 27.90 27.31
-1.34 -1.78
200 190 180 170 160 150 140 130 120 110 100 90 80 70 60 50 40 30 20 10 0
f1 (ppm)

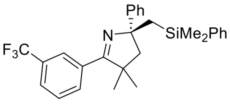

3l

^{19}F NMR, $CDCl_3$, 376 MHz

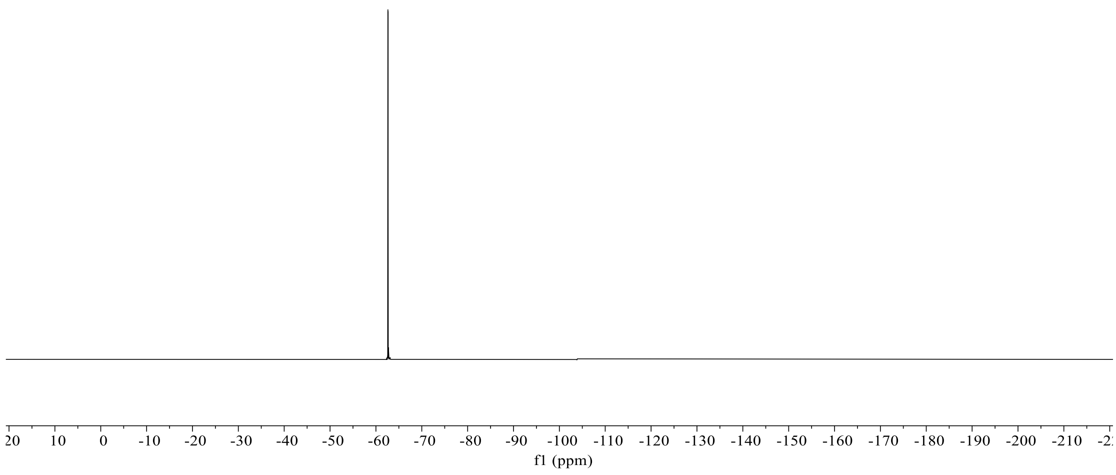

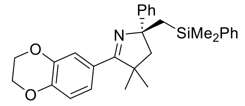

3m

^{1}H NMR, $CDCl_3$, 400 MHz

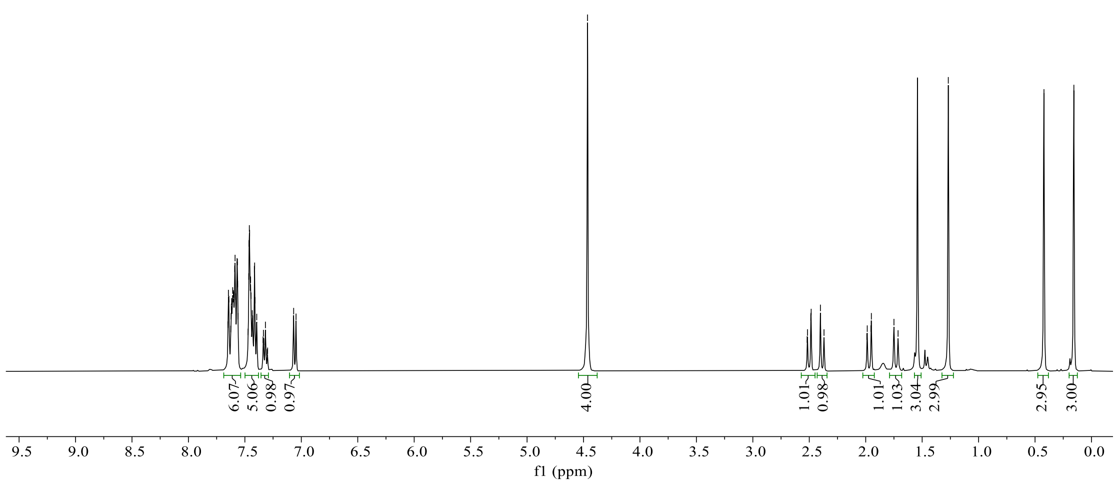

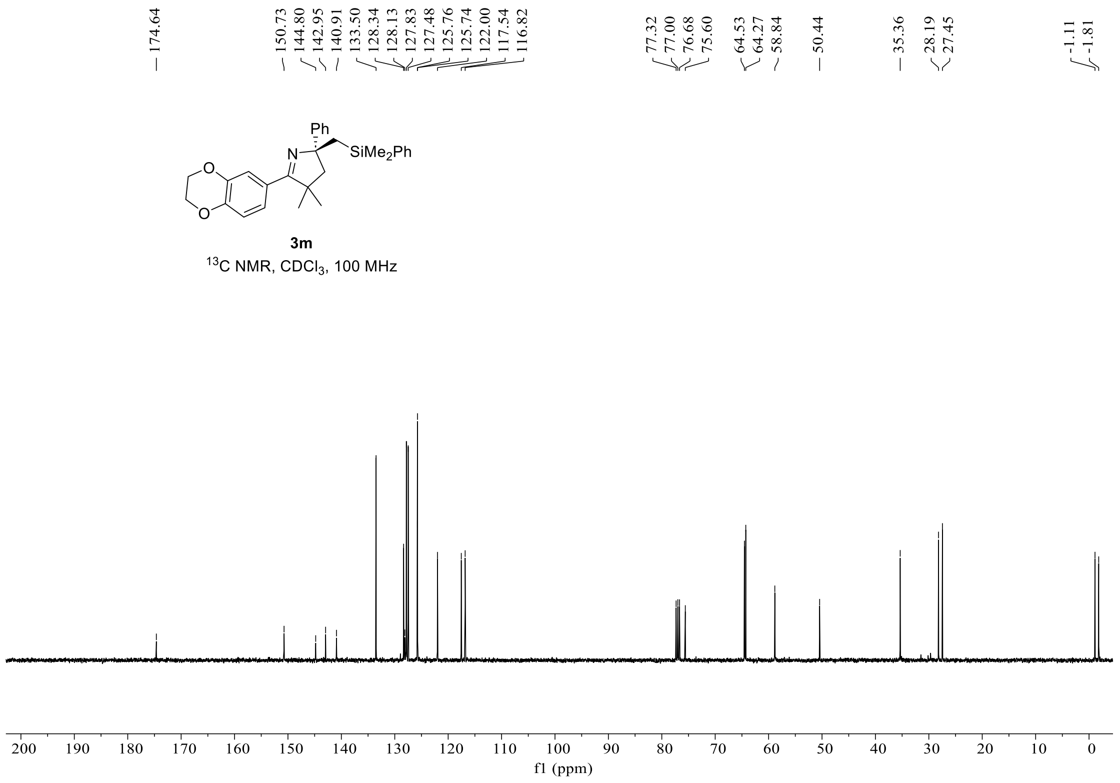
174.64
150.73 144.80 142.95 140.91 133.50 128.34 128.13 127.83 127.48 125.76 125.74 122.00 117.54 116.82
77.32 77.00 76.68 75.60 64.53 64.27 58.84 50.44
35.36 28.19 27.45
-1.11 -1.81
Ph
N
SiMe2Ph
O
O
3m
13C NMR, CDCl3, 100 MHz
f1 (ppm)

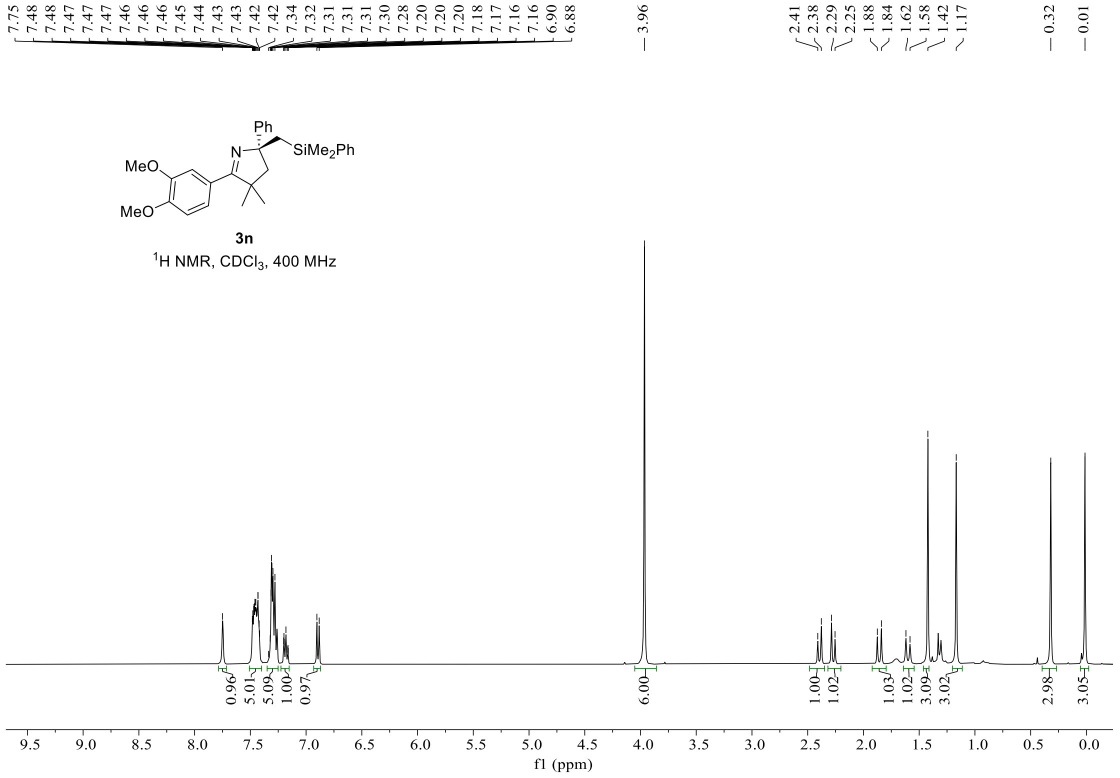
7.75 7.48 7.48 7.47 7.47 7.47 7.46 7.46 7.46 7.45 7.44 7.43 7.43 7.42 7.42 7.34 7.32 7.31 7.31 7.30 7.28 7.20 7.20 7.20 7.18 7.17 7.16 7.16 6.90 6.88
3.96
2.41 2.38 2.29 2.25 1.88 1.84 1.62 1.58 1.42 1.17
0.32 0.01
Ph
N
SiMe2Ph
MeO
MeO
3n
1H NMR, CDCl3, 400 MHz
0.96 5.01 5.09 1.00 0.97 6.00 1.00 1.02 1.03 1.02 3.09 3.02 2.98 3.05
f1 (ppm)

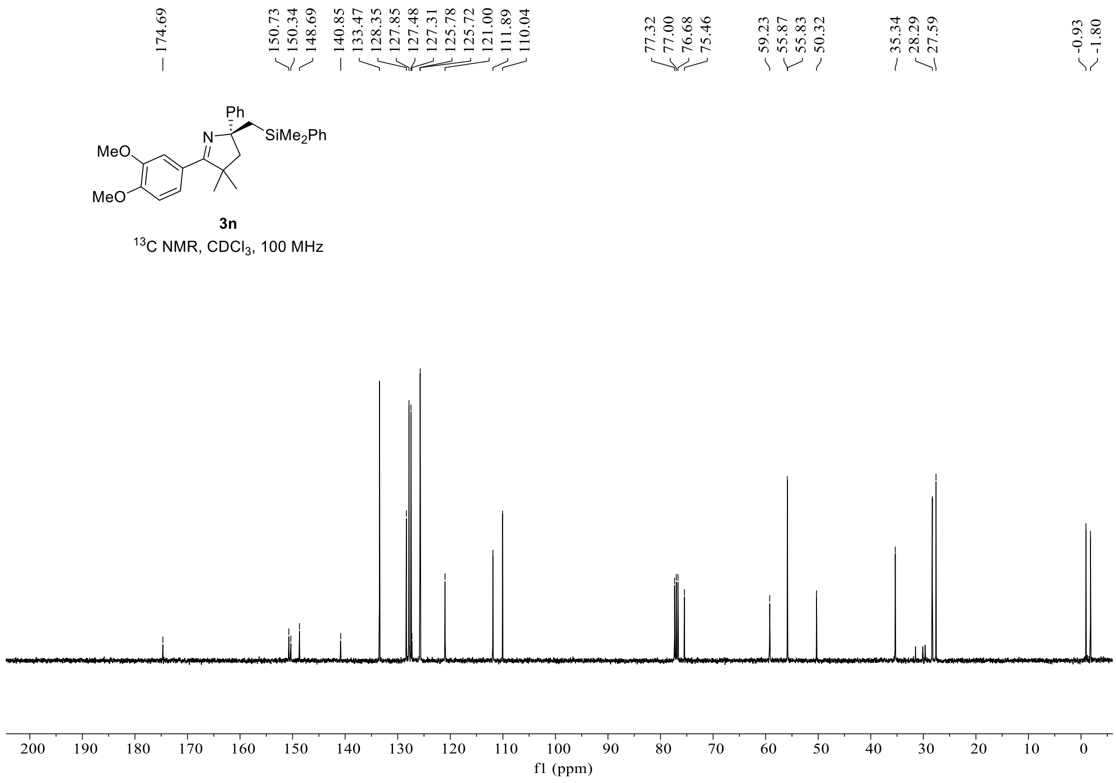

3n
^{13}C NMR, $CDCl_3$, 100 MHz

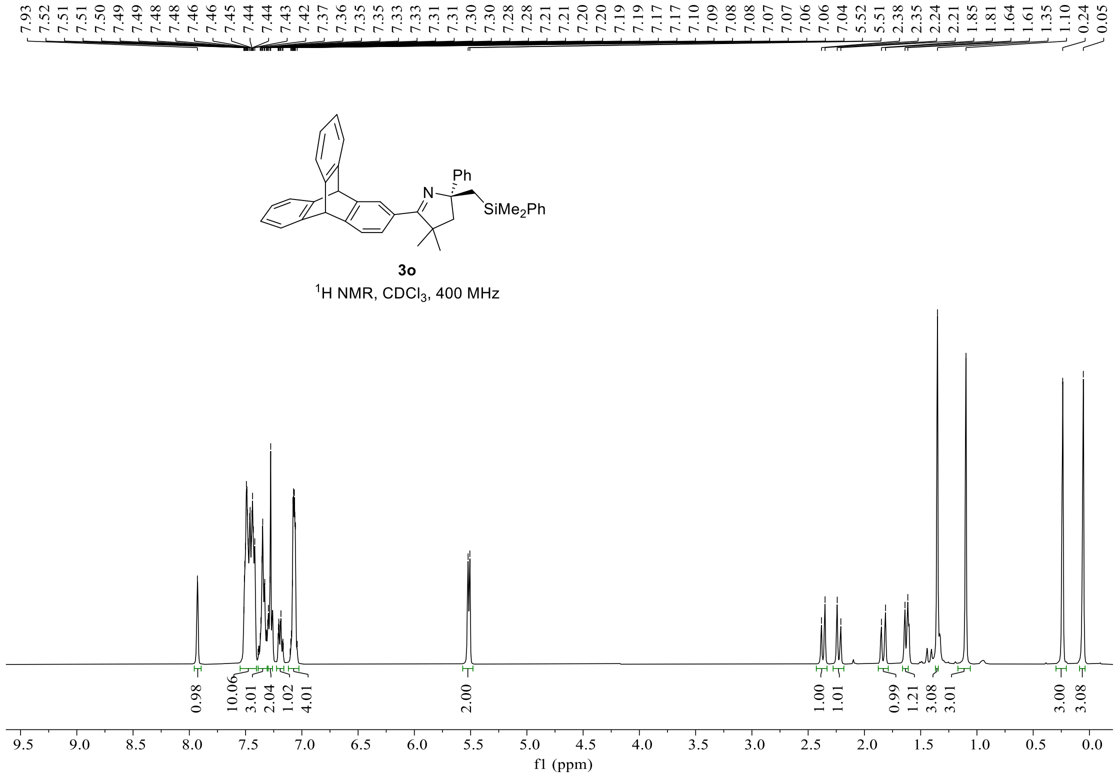

3o
^{1}H NMR, $CDCl_3$, 400 MHz

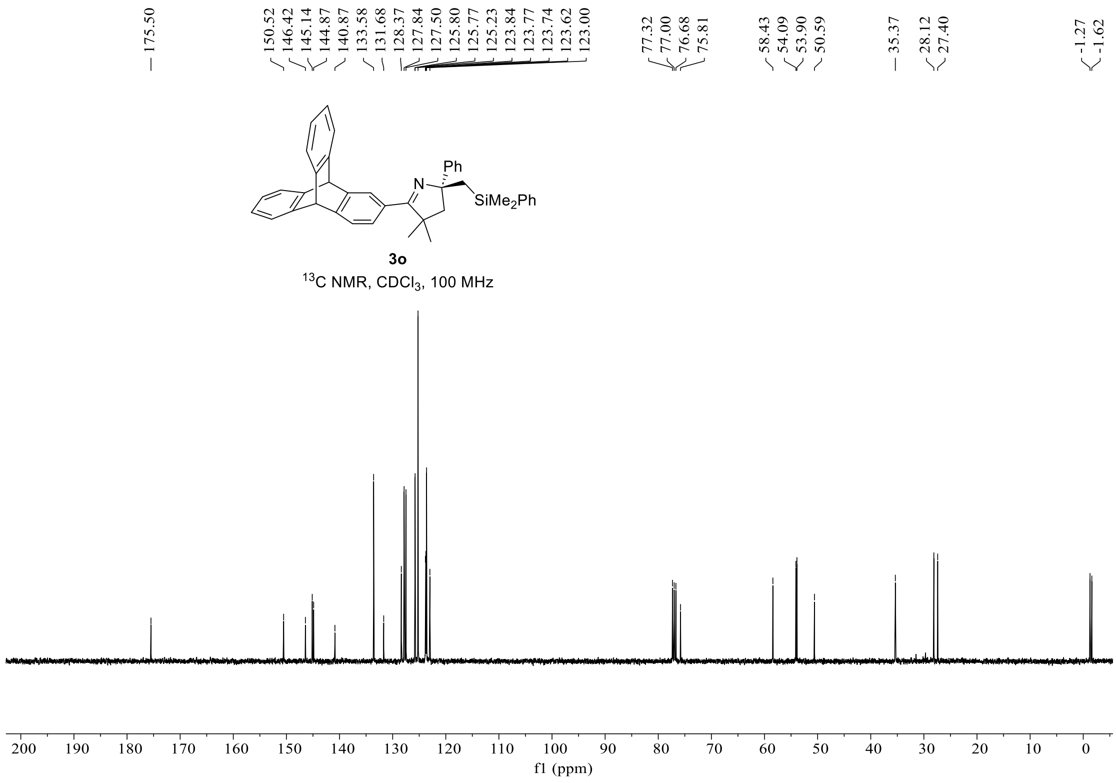
175.50
150.52
146.42
145.14
144.87
140.87
133.58
131.68
128.37
127.84
127.50
125.80
125.77
125.23
123.84
123.77
123.74
123.62
123.00
77.32
77.00
76.68
75.81
58.43
54.09
53.90
50.59
35.37
28.12
27.40
-1.27
-1.62
Ph
N
SiMe2Ph
3o
13C NMR, CDCl3, 100 MHz
200 190 180 170 160 150 140 130 120 110 100 90 80 70 60 50 40 30 20 10 0
f1 (ppm)

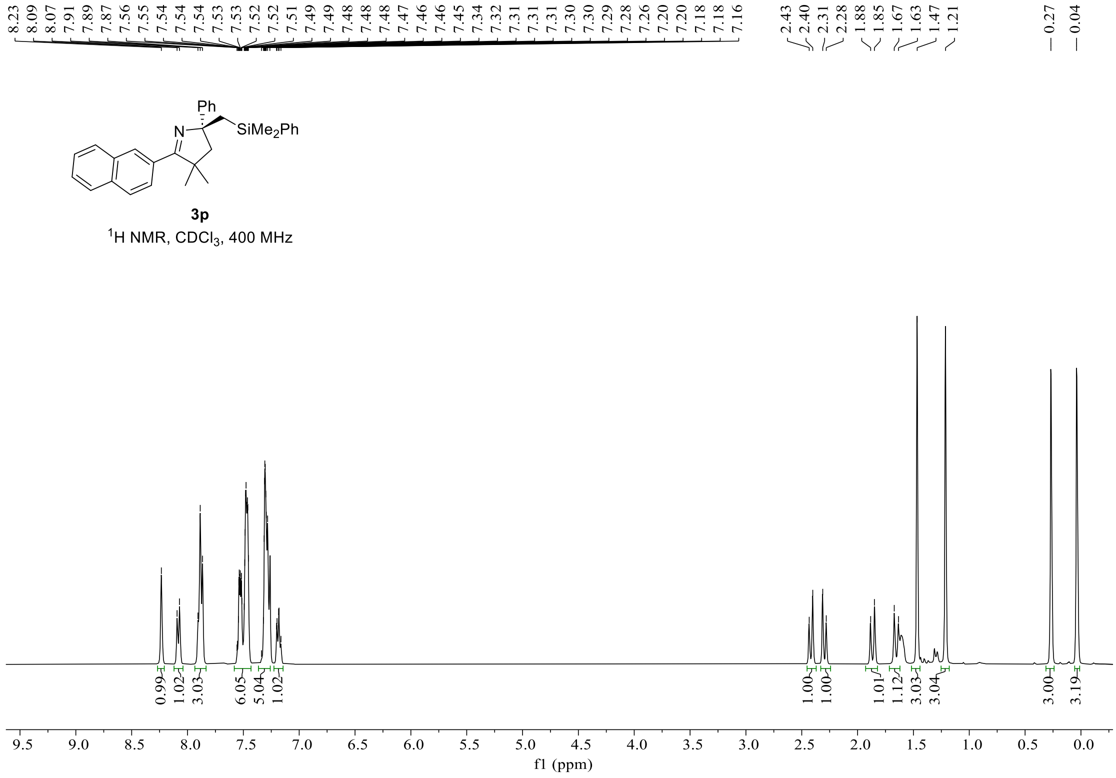
8.23
8.09
8.07
7.91
7.89
7.87
7.56
7.55
7.54
7.54
7.54
7.53
7.53
7.52
7.52
7.51
7.49
7.49
7.48
7.48
7.48
7.47
7.46
7.46
7.45
7.34
7.32
7.31
7.31
7.31
7.30
7.30
7.29
7.28
7.26
7.20
7.20
7.18
7.16
2.43
2.40
2.31
2.28
1.88
1.85
1.67
1.63
1.47
1.21
0.27
0.04
Ph
N
SiMe2Ph
3p
1H NMR, CDCl3, 400 MHz
0.99
1.02
3.03
6.05
5.04
1.02
1.00
1.00
1.01
1.12
3.03
3.04
3.00
3.19
9.5 9.0 8.5 8.0 7.5 7.0 6.5 6.0 5.5 5.0 4.5 4.0 3.5 3.0 2.5 2.0 1.5 1.0 0.5 0.0
f1 (ppm)

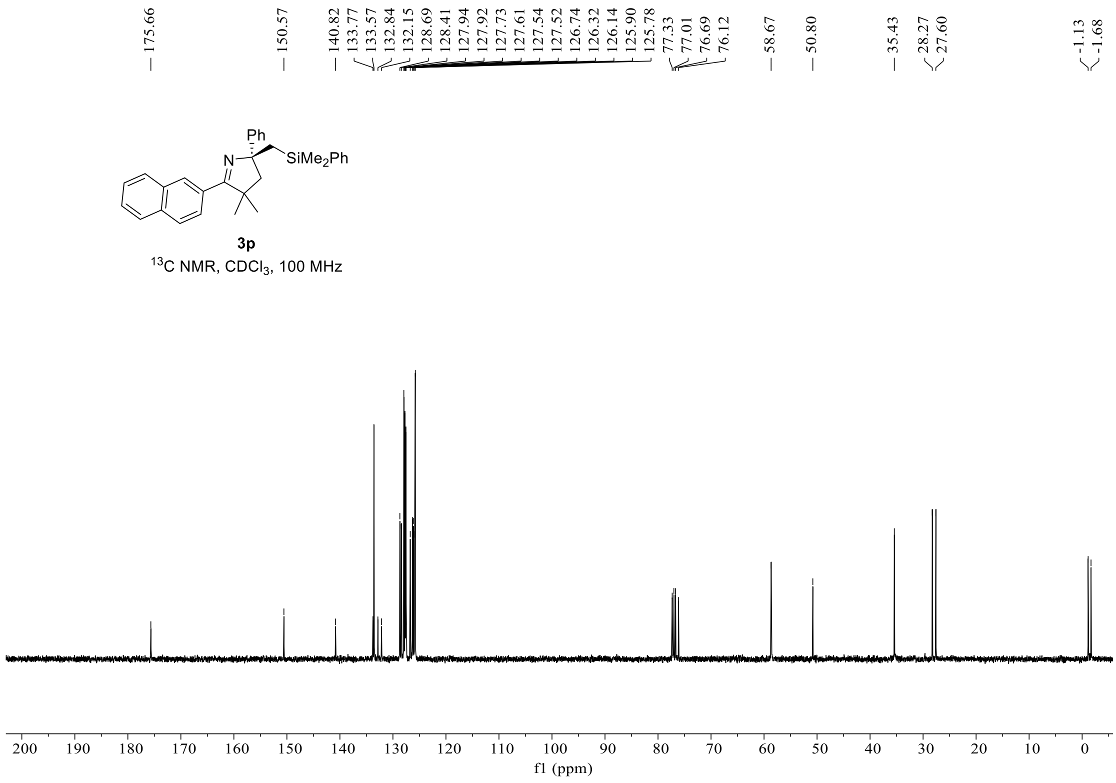

3p

^{13}C NMR, CDCl$_3$, 100 MHz

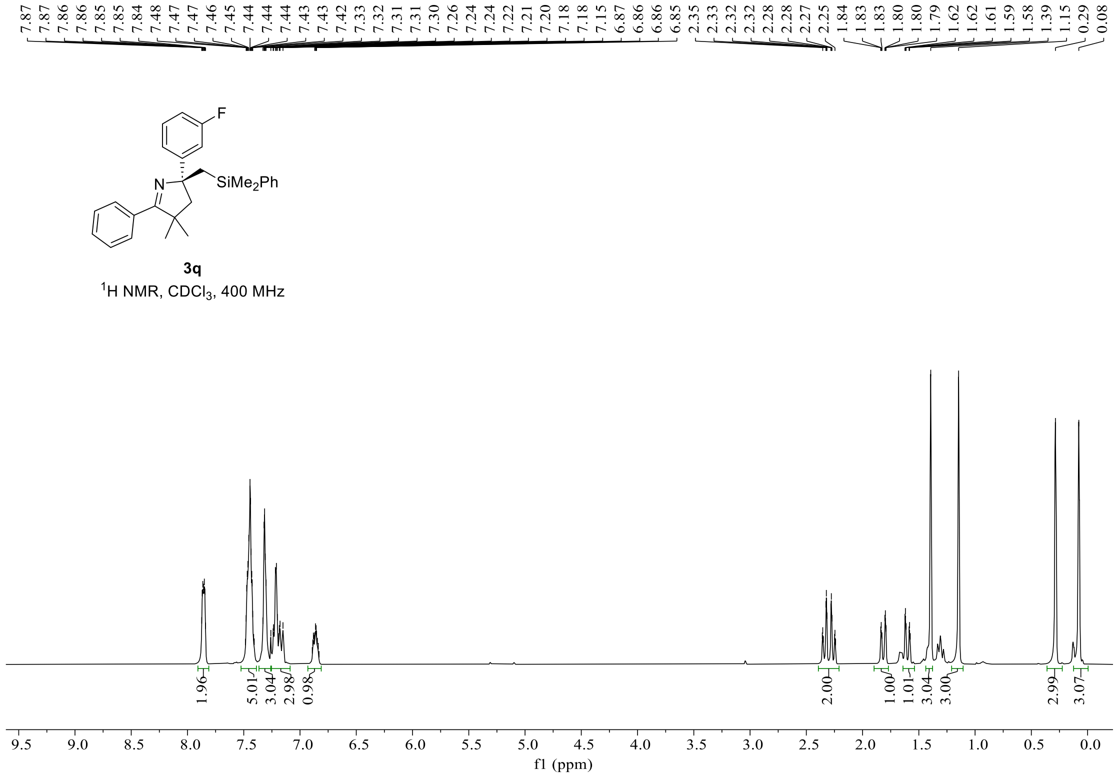

3q

^{1}H NMR, CDCl$_3$, 400 MHz

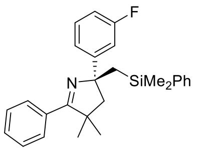

3q
^{13}C NMR, $CDCl_3$, 100 MHz

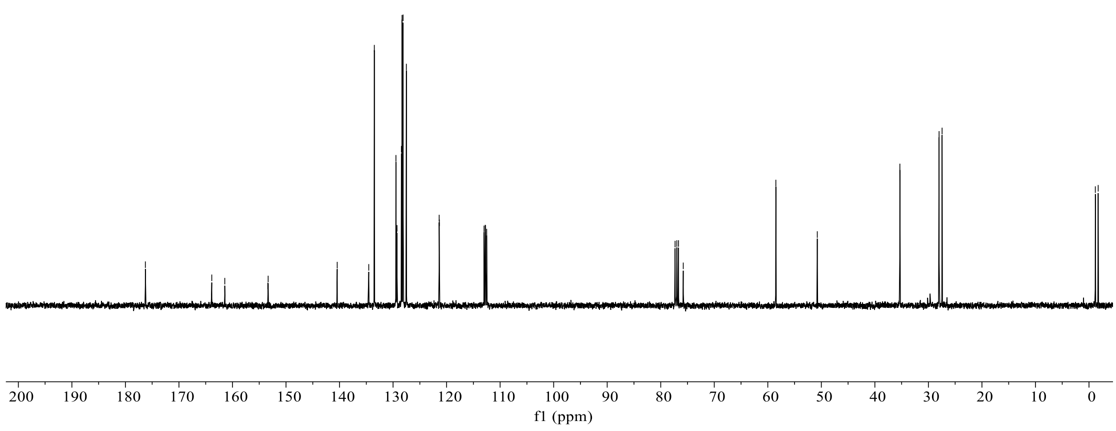

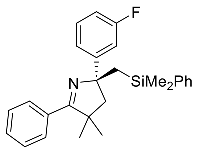

3q
^{19}F NMR, $CDCl_3$, 376 MHz

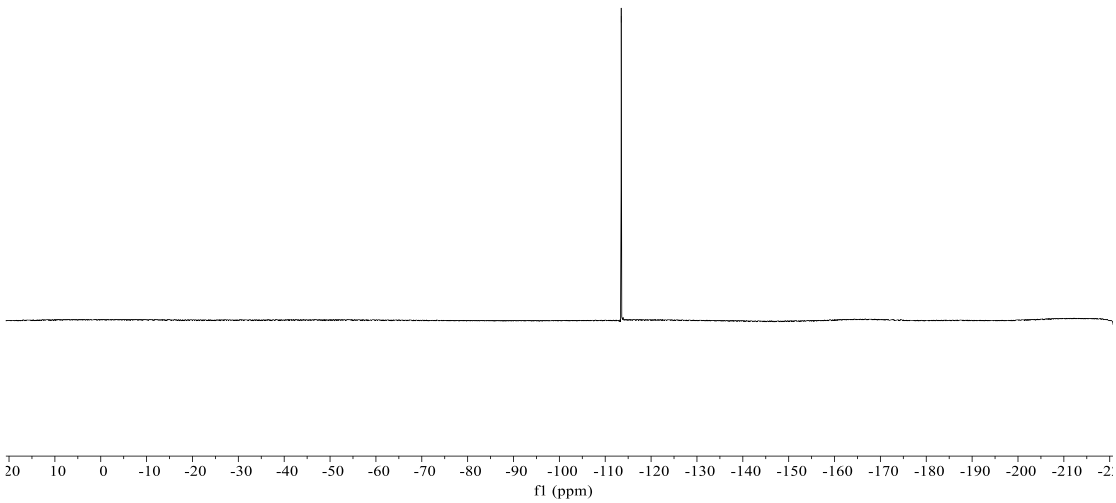

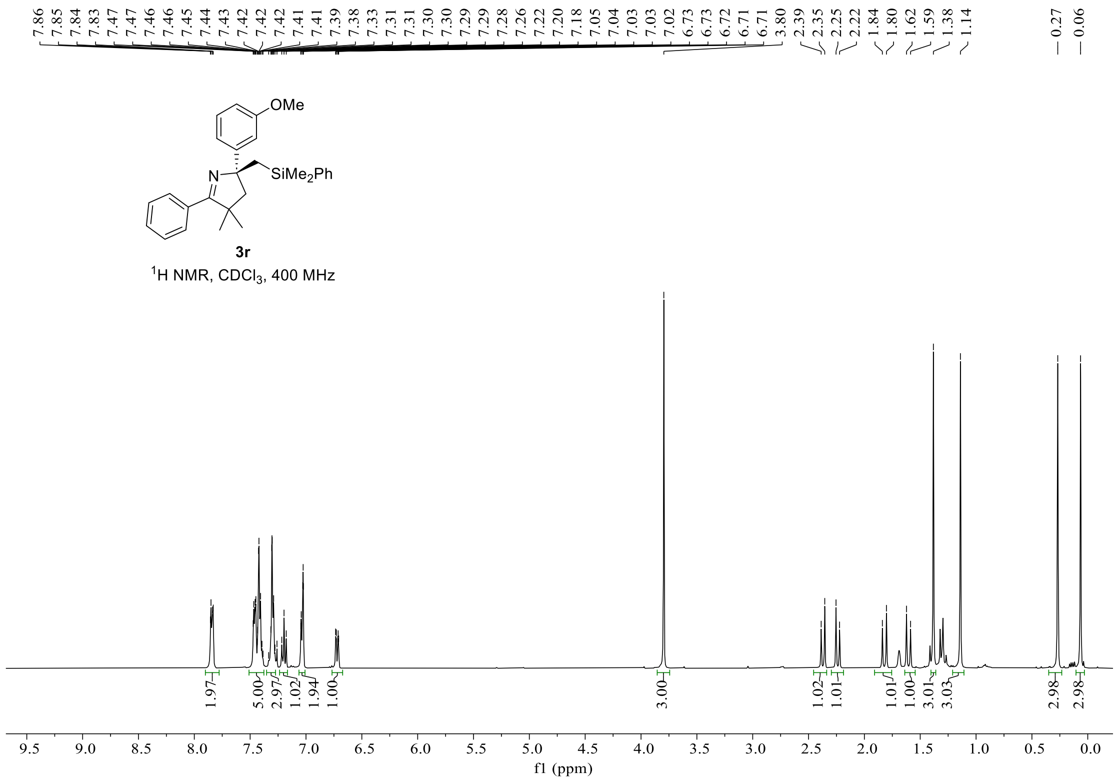
7.86 7.85 7.84 7.83 7.47 7.47 7.46 7.45 7.44 7.43 7.42 7.42 7.42 7.41 7.41 7.39 7.38 7.33 7.31 7.30 7.30 7.29 7.29 7.28 7.26 7.22 7.20 7.18 7.05 7.04 7.03 7.03 7.02 6.73 6.73 6.72 6.71 3.80 2.39 2.35 2.25 2.22 1.84 1.80 1.62 1.59 1.38 1.14 0.27 0.06
OMe
N
SiMe2Ph
3r
1H NMR, CDCl3, 400 MHz
1.97 5.00 2.97 1.02 1.94 1.00 3.00 1.02 1.01 1.01 1.00 3.01 3.03 2.98 2.98
9.5 9.0 8.5 8.0 7.5 7.0 6.5 6.0 5.5 5.0 4.5 4.0 3.5 3.0 2.5 2.0 1.5 1.0 0.5 0.0
f1 (ppm)

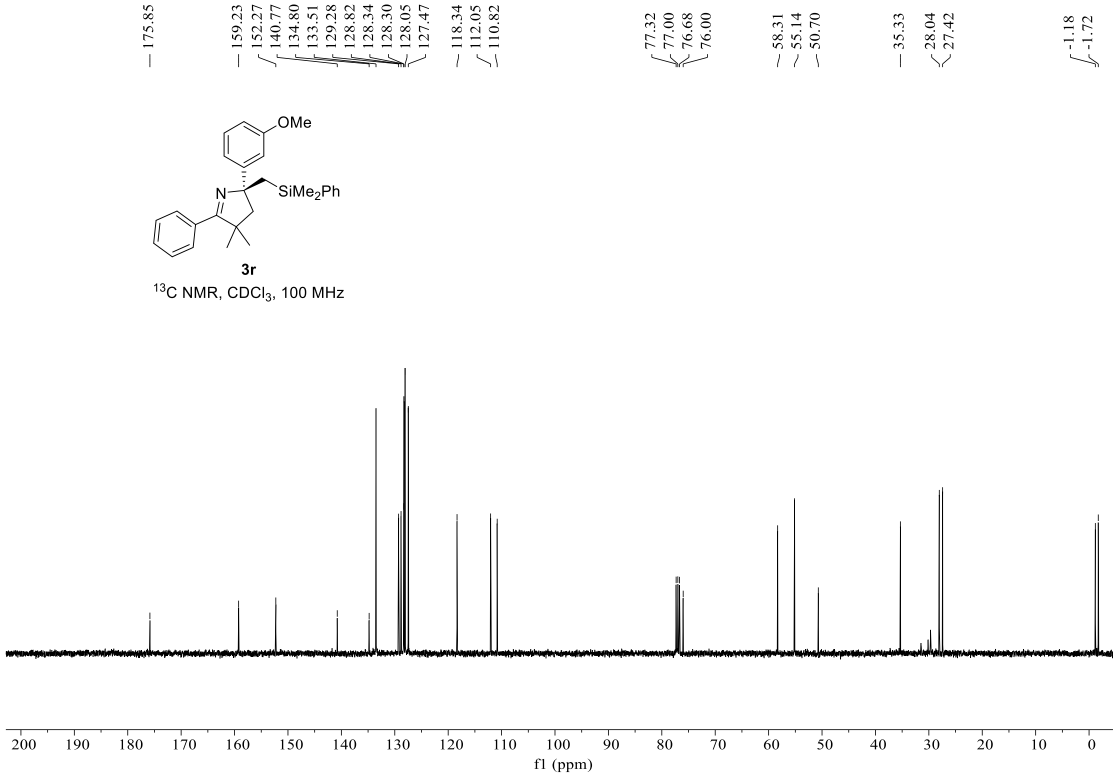
175.85 159.23 152.27 140.77 134.80 133.51 129.28 128.82 128.34 128.30 128.05 127.47 118.34 112.05 110.82 77.32 77.00 76.68 76.00 58.31 55.14 50.70 35.33 28.04 27.42 -1.18 -1.72
OMe
N
SiMe2Ph
3r
13C NMR, CDCl3, 100 MHz
200 190 180 170 160 150 140 130 120 110 100 90 80 70 60 50 40 30 20 10 0
f1 (ppm)

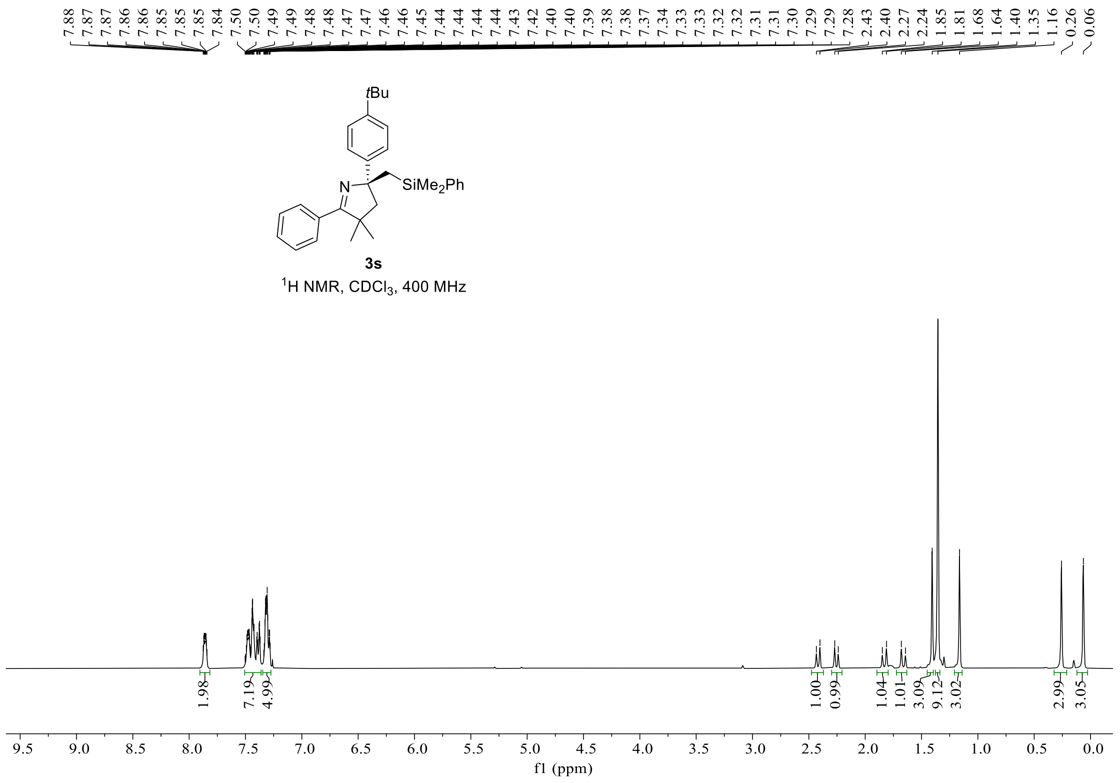
7.88 7.87 7.87 7.86 7.86 7.85 7.85 7.84 7.50 7.50 7.49 7.49 7.48 7.48 7.47 7.46 7.46 7.45 7.44 7.44 7.44 7.44 7.43 7.42 7.40 7.40 7.39 7.38 7.38 7.37 7.34 7.33 7.33 7.32 7.32 7.31 7.31 7.30 7.29 7.29 7.28 2.43 2.40 2.27 2.24 1.85 1.81 1.68 1.64 1.40 1.35 1.16 0.26 0.06
tBu
N
SiMe2Ph
3s
1H NMR, CDCl3, 400 MHz
1.98 7.19 4.99 1.00 0.99 1.04 1.01 3.09 9.12 3.02 2.99 3.05
9.5 9.0 8.5 8.0 7.5 7.0 6.5 6.0 5.5 5.0 4.5 4.0 3.5 3.0 2.5 2.0 1.5 1.0 0.5 0.0
f1 (ppm)

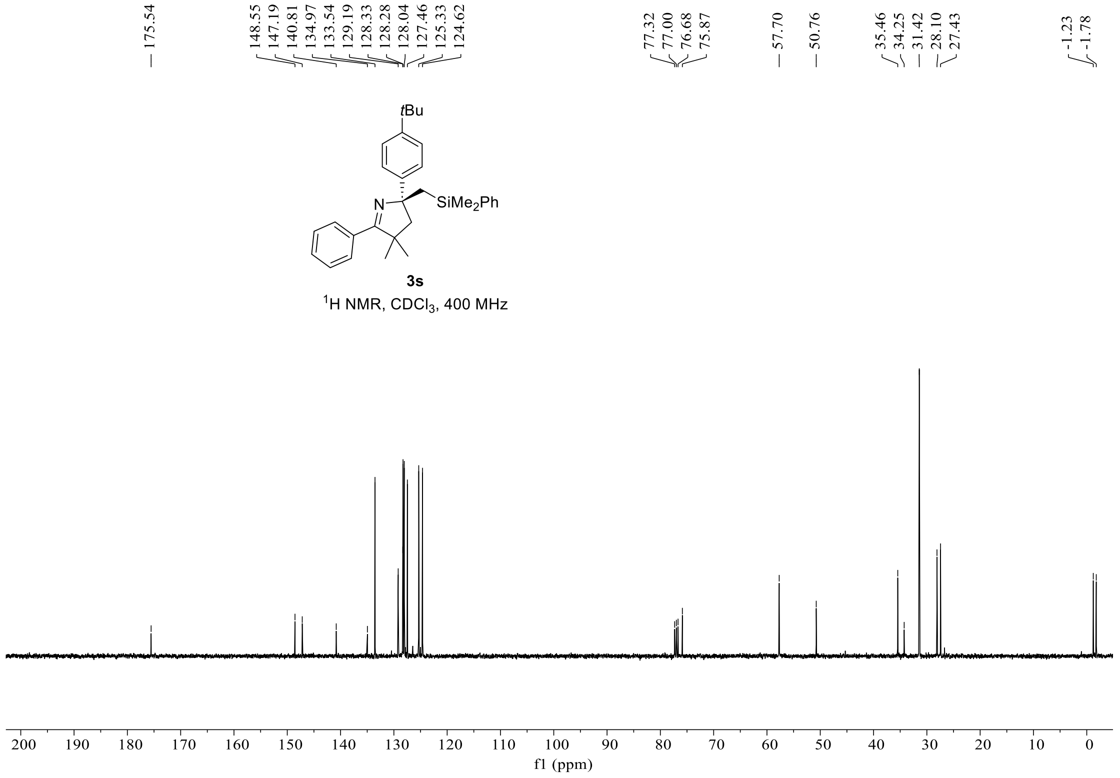
175.54
148.55 147.19 140.81 134.97 133.54 129.19 128.33 128.28 128.04 127.46 125.33 124.62
77.32 77.00 76.68 75.87
57.70
50.76
35.46 34.25 31.42 28.10 27.43
-1.23 -1.78
tBu
N
SiMe2Ph
3s
1H NMR, CDCl3, 400 MHz
200 190 180 170 160 150 140 130 120 110 100 90 80 70 60 50 40 30 20 10 0
f1 (ppm)

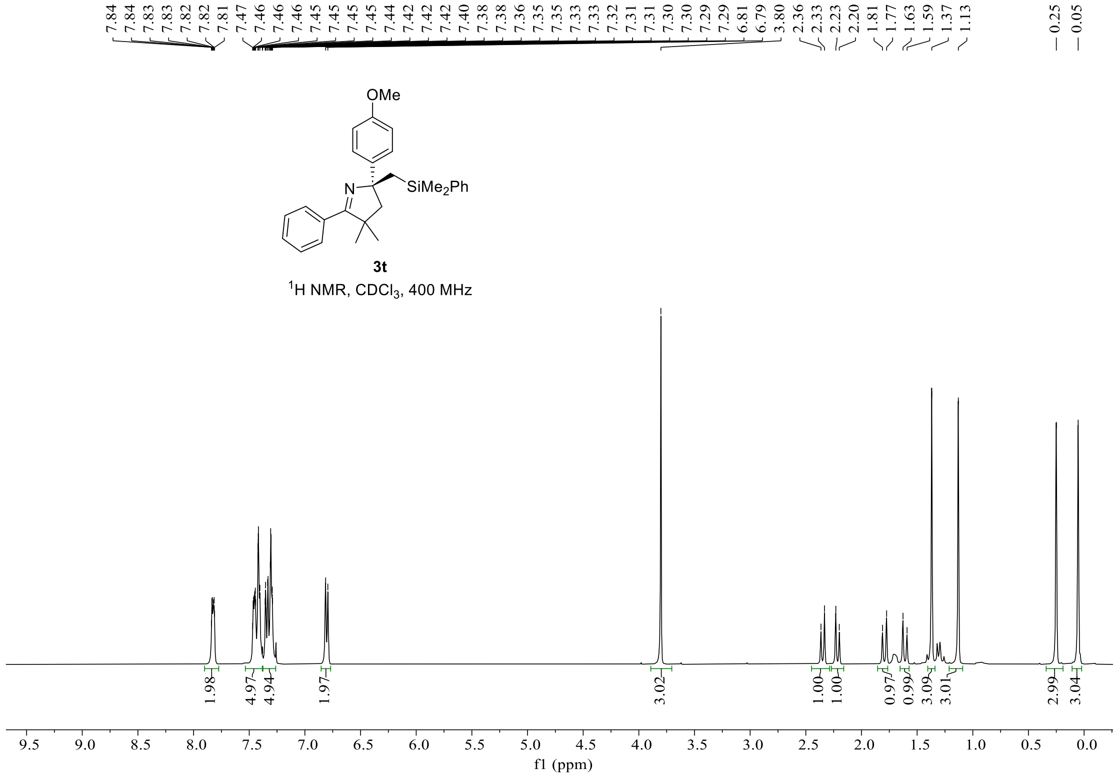
7.84 7.84 7.83 7.82 7.82 7.81 7.47 7.46 7.46 7.45 7.45 7.45 7.45 7.44 7.42 7.42 7.42 7.40 7.38 7.38 7.36 7.35 7.35 7.33 7.33 7.32 7.31 7.31 7.30 7.30 7.29 7.29 6.81 6.79 3.80 2.36 2.33 2.23 2.20 1.81 1.77 1.63 1.59 1.37 1.13 0.25 0.05
OMe
N
SiMe2Ph
3t
1H NMR, CDCl3, 400 MHz
1.98 4.97 4.94 1.97 3.02 1.00 1.00 0.97 0.99 3.09 3.01 2.99 3.04
9.5 9.0 8.5 8.0 7.5 7.0 6.5 6.0 5.5 5.0 4.5 4.0 3.5 3.0 2.5 2.0 1.5 1.0 0.5 0.0
f1 (ppm)

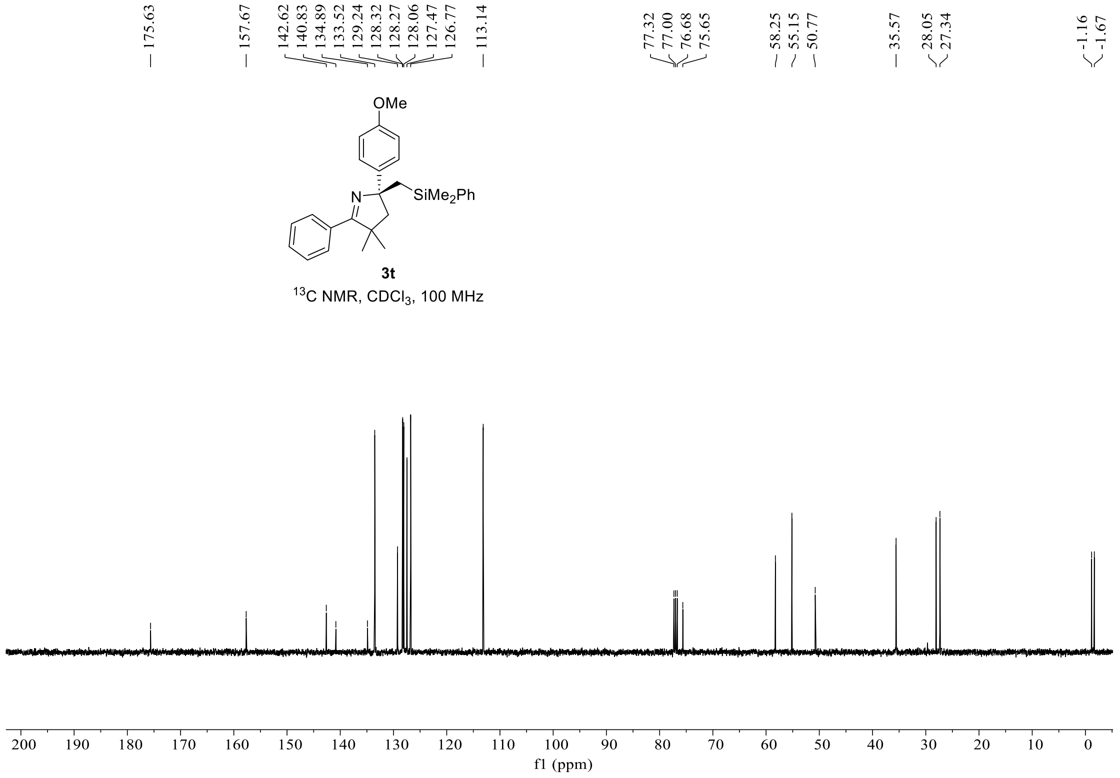
175.63 157.67 142.62 140.83 134.89 133.52 129.24 128.32 128.27 128.06 127.47 126.77 113.14 77.32 77.00 76.68 75.65 58.25 55.15 50.77 35.57 28.05 27.34 -1.16 -1.67
OMe
N
SiMe2Ph
3t
13C NMR, CDCl3, 100 MHz
200 190 180 170 160 150 140 130 120 110 100 90 80 70 60 50 40 30 20 10 0
f1 (ppm)

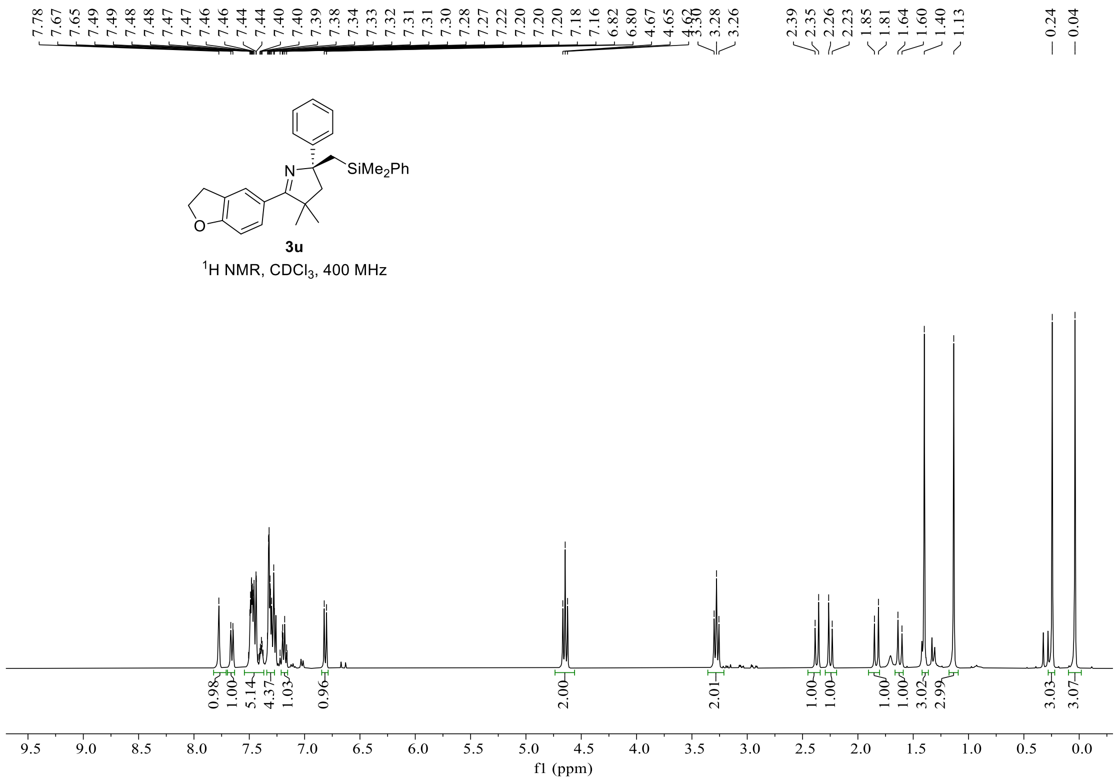
7.78 7.67 7.65 7.49 7.48 7.48 7.47 7.46 7.44 7.44 7.40 7.40 7.39 7.38 7.34 7.33 7.32 7.31 7.31 7.30 7.28 7.27 7.22 7.20 7.20 7.20 7.18 7.16 6.82 6.80 4.67 4.65 4.62 3.30 3.28 3.26
2.39 2.35 2.26 2.23 1.85 1.81 1.64 1.60 1.40 1.13
0.24 0.04
N
SiMe2Ph
O
3u
1H NMR, CDCl3, 400 MHz
0.98 1.00 5.14 4.37 1.03 0.96 2.00 2.01 1.00 1.00 1.00 1.00 3.02 2.99 3.03 3.07
f1 (ppm)

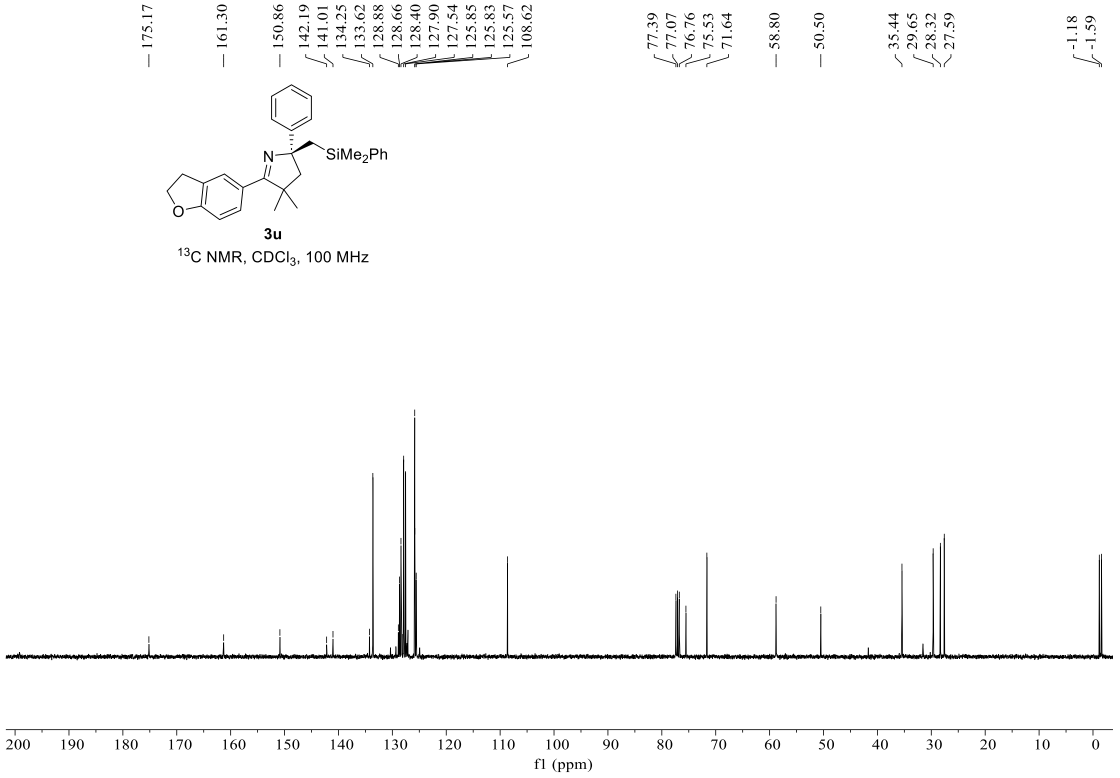
175.17 161.30 150.86 142.19 141.01 134.25 133.62 128.88 128.66 128.40 127.90 127.54 125.85 125.83 125.57 108.62
77.39 77.07 76.76 75.53 71.64 58.80 50.50 35.44 29.65 28.32 27.59 -1.18 -1.59
N
SiMe2Ph
O
3u
13C NMR, CDCl3, 100 MHz
f1 (ppm)

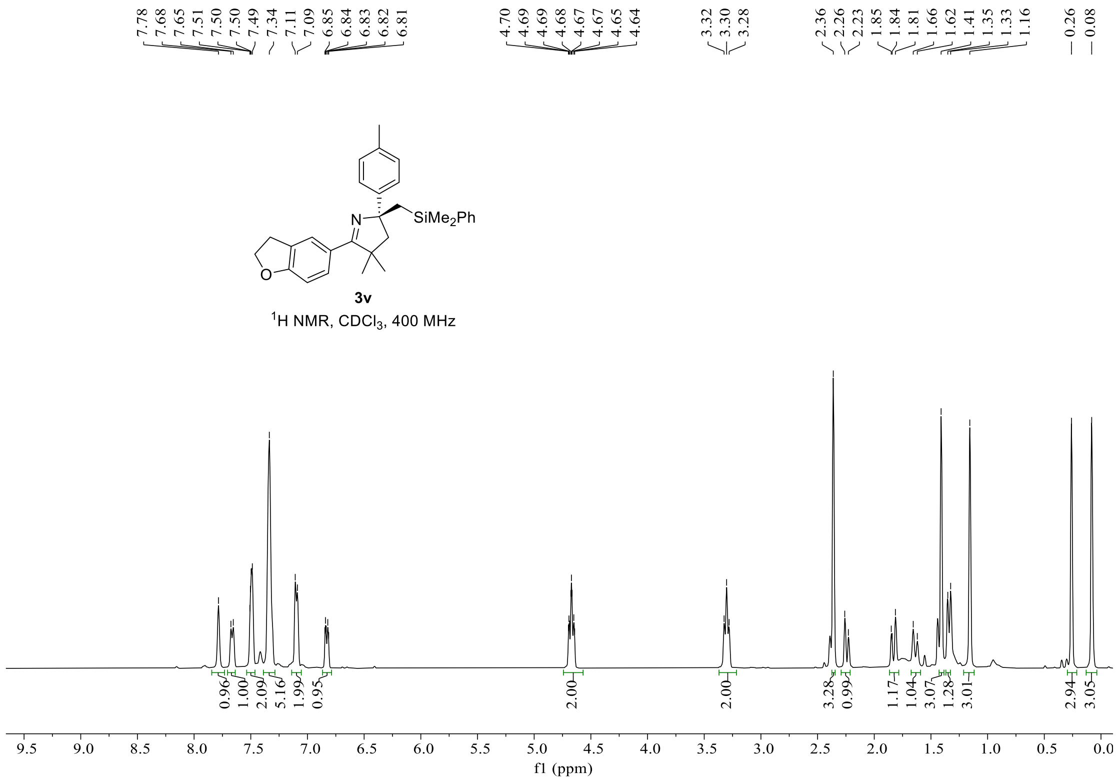

3v

^{1}H NMR, $CDCl_3$, 400 MHz

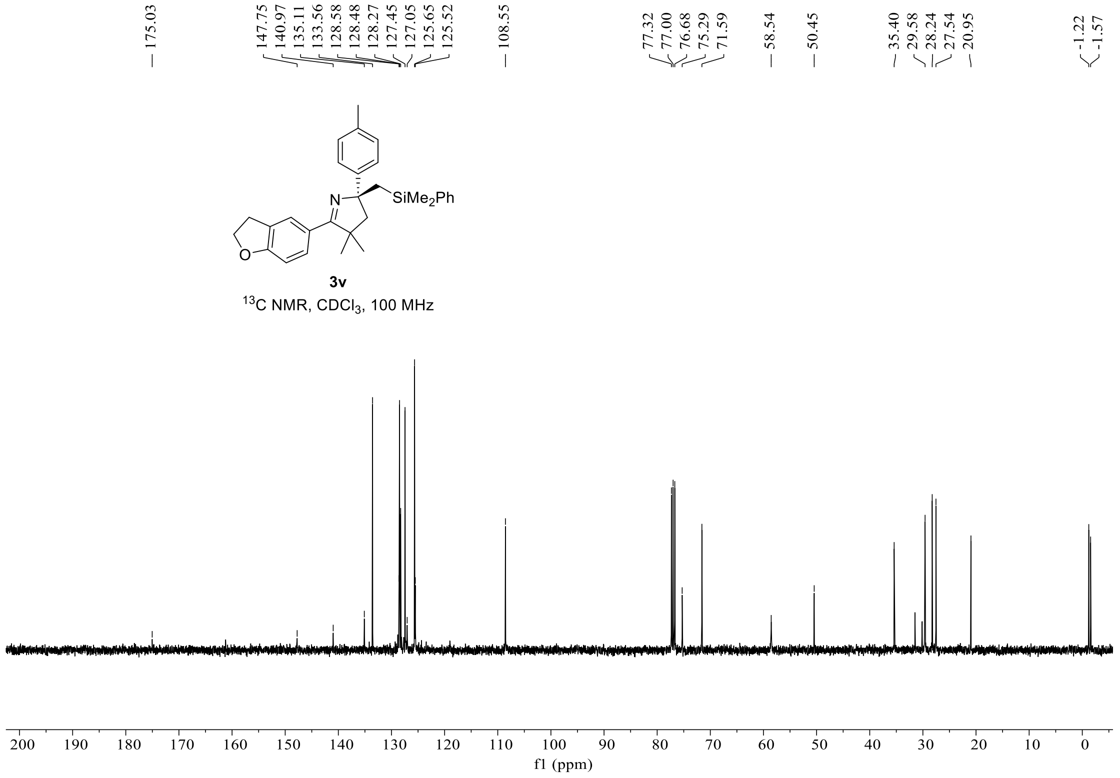

3v

^{13}C NMR, $CDCl_3$, 100 MHz

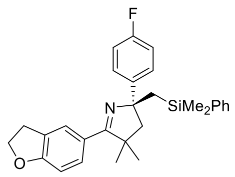

3w

^{1}H NMR, $CDCl_3$, 400 MHz

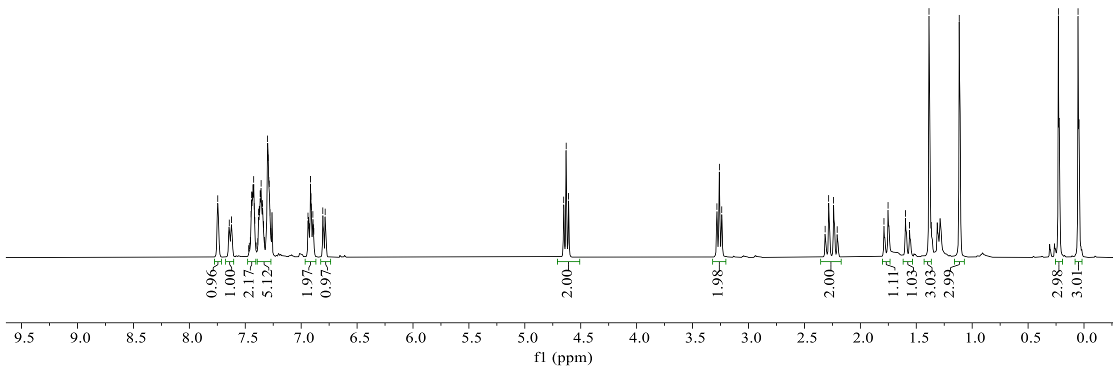

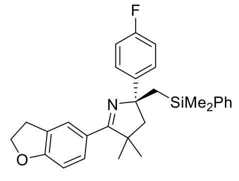

3w

^{13}C NMR, $CDCl_3$, 100 MHz

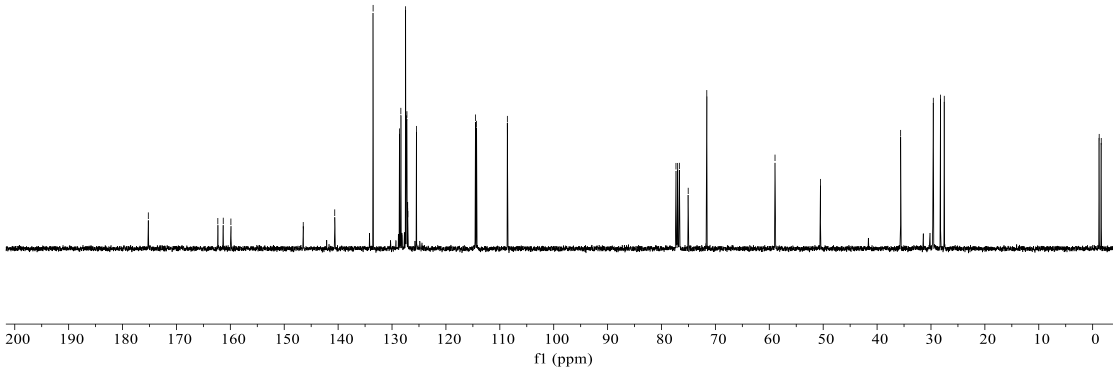

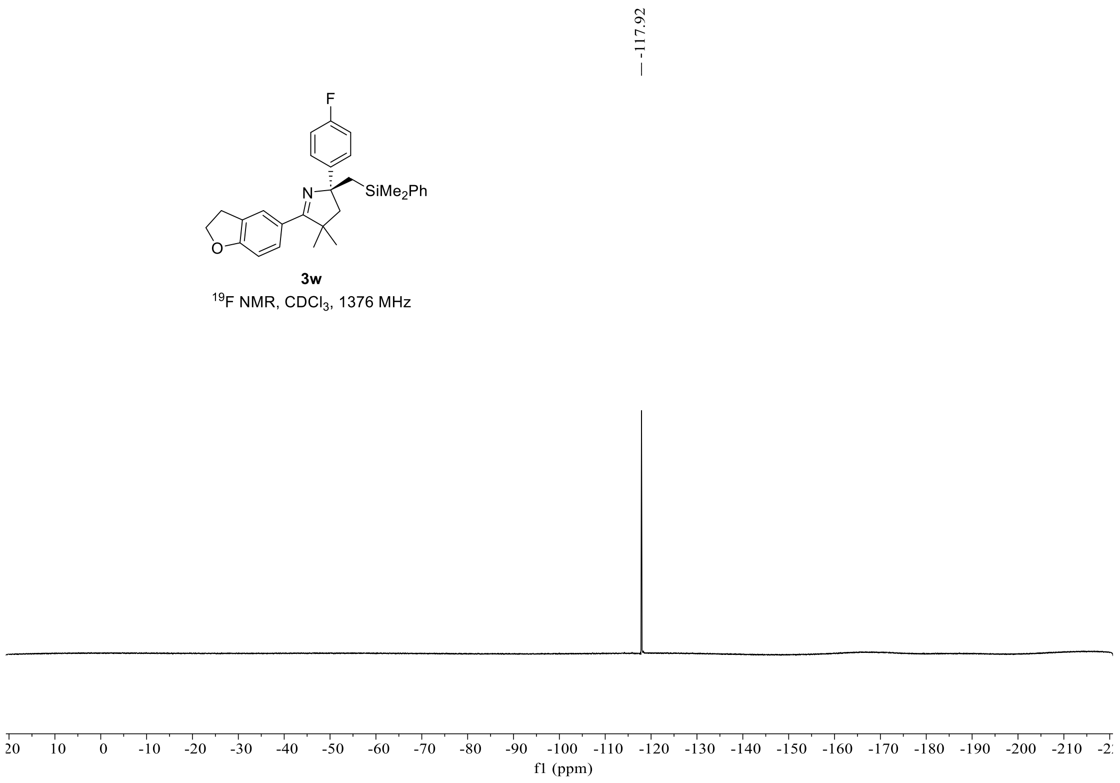
-117.92
F
N
SiMe2Ph
O
3w
19F NMR, CDCl3, 1376 MHz
20 10 0 -10 -20 -30 -40 -50 -60 -70 -80 -90 -100 -110 -120 -130 -140 -150 -160 -170 -180 -190 -200 -210
f1 (ppm)

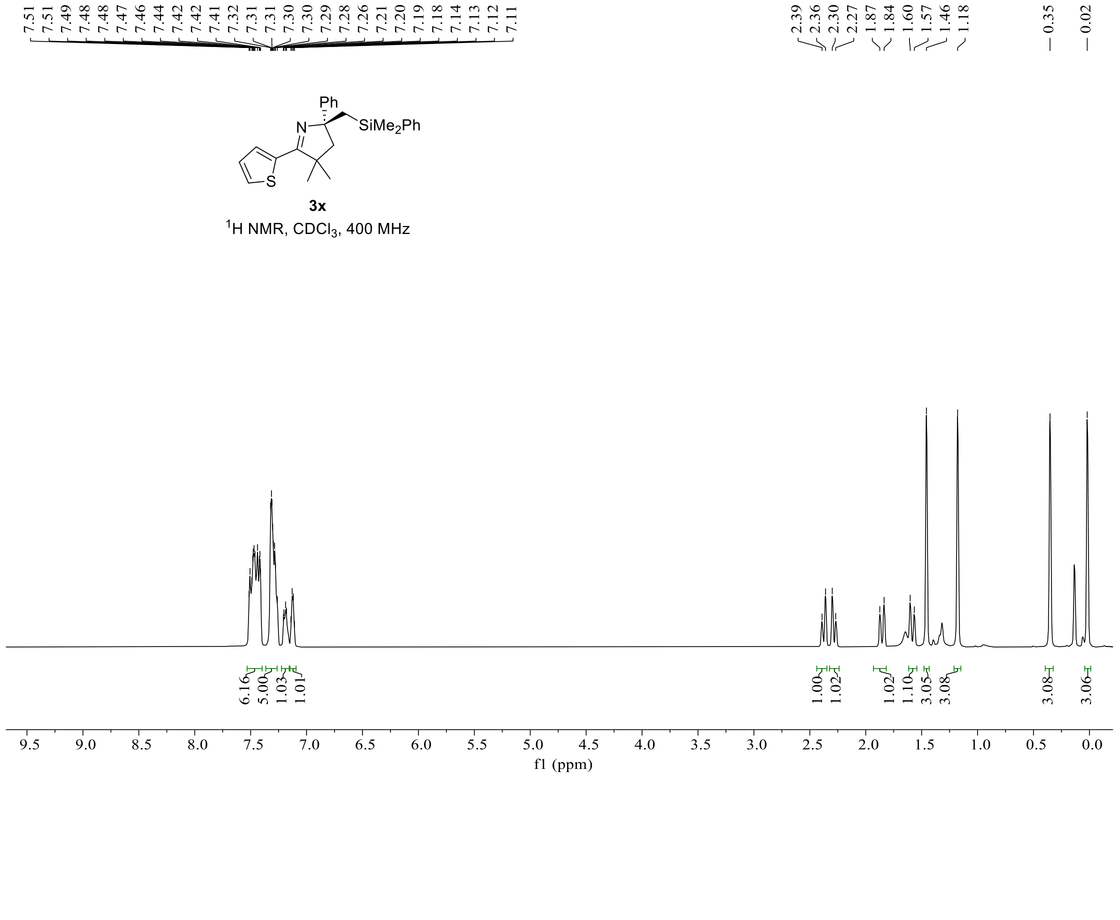
7.51 7.51 7.49 7.48 7.48 7.47 7.46 7.44 7.42 7.42 7.41 7.32 7.31 7.31 7.30 7.30 7.29 7.28 7.26 7.21 7.20 7.19 7.18 7.14 7.13 7.12 7.11
2.39 2.36 2.30 2.27 1.87 1.84 1.60 1.57 1.46 1.18
0.35
0.02
Ph
N
SiMe2Ph
S
3x
1H NMR, CDCl3, 400 MHz
6.16 5.00 1.03 1.01
1.00 1.02
1.02 1.10 3.05 3.08
3.08
3.06
9.5 9.0 8.5 8.0 7.5 7.0 6.5 6.0 5.5 5.0 4.5 4.0 3.5 3.0 2.5 2.0 1.5 1.0 0.5 0.0
f1 (ppm)

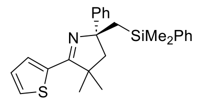

3x

^{13}C NMR, $CDCl_3$, 100 MHz

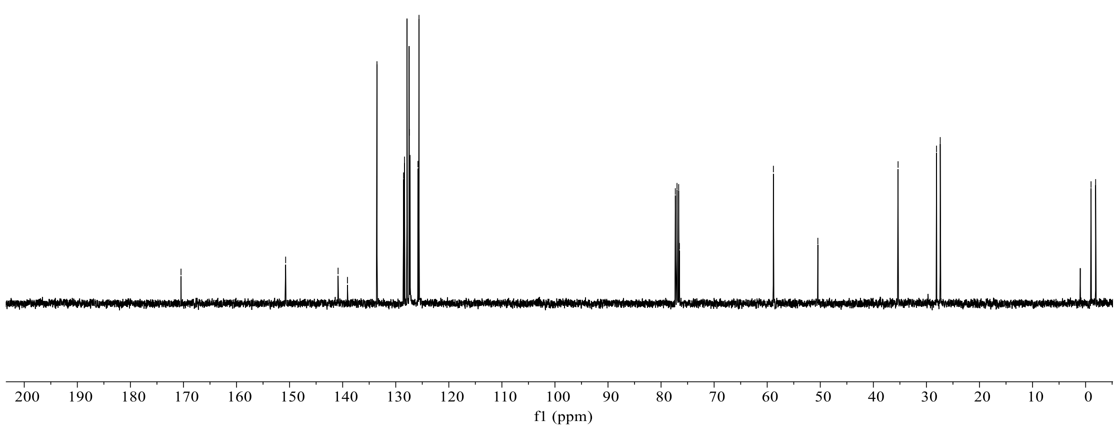

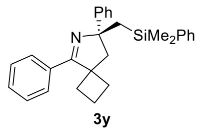

3y

^{1}H NMR, $CDCl_3$, 400 MHz

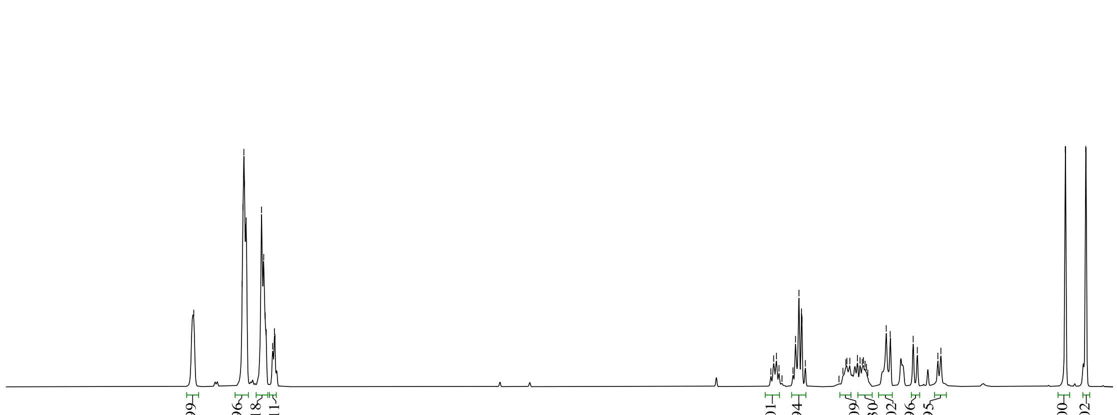

174.52, 149.61, 140.77, 135.13, 133.52, 129.27, 128.38, 128.20, 128.03, 127.87, 127.51, 125.96, 125.77, 77.32, 77.00, 76.68, 76.55, 57.70, 54.98, 33.29, 32.98, 32.27, 16.39, -1.23, -1.76

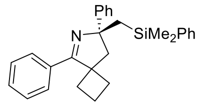

3y

^{13}C NMR, $CDCl_3$, 100 MHz

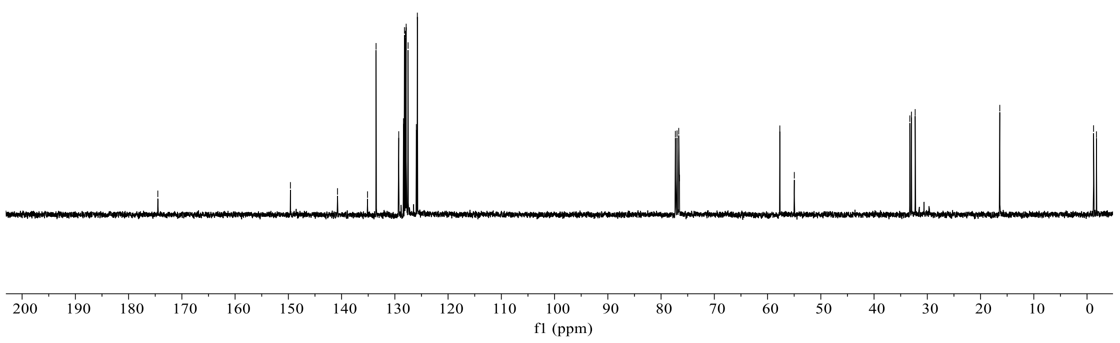

7.82, 7.81, 7.80, 7.79, 7.50, 7.49, 7.48, 7.48, 7.47, 7.45, 7.44, 7.36, 7.33, 7.33, 7.31, 7.31, 7.29, 7.28, 7.27, 7.27, 7.22, 7.20, 7.18, 2.38, 2.35, 2.26, 2.23, 1.96, 1.94, 1.92, 1.91, 1.89, 1.86, 1.83, 1.79, 1.78, 1.77, 1.76, 1.74, 1.72, 1.70, 1.68, 1.68, 1.67, 1.66, 1.65, 1.64, 1.58, 1.56, 1.54, 1.34, 1.31, 1.28, 1.27, 1.25, 0.27, 0.04

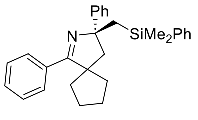

3z

^{1}H NMR, $CDCl_3$, 400 MHz

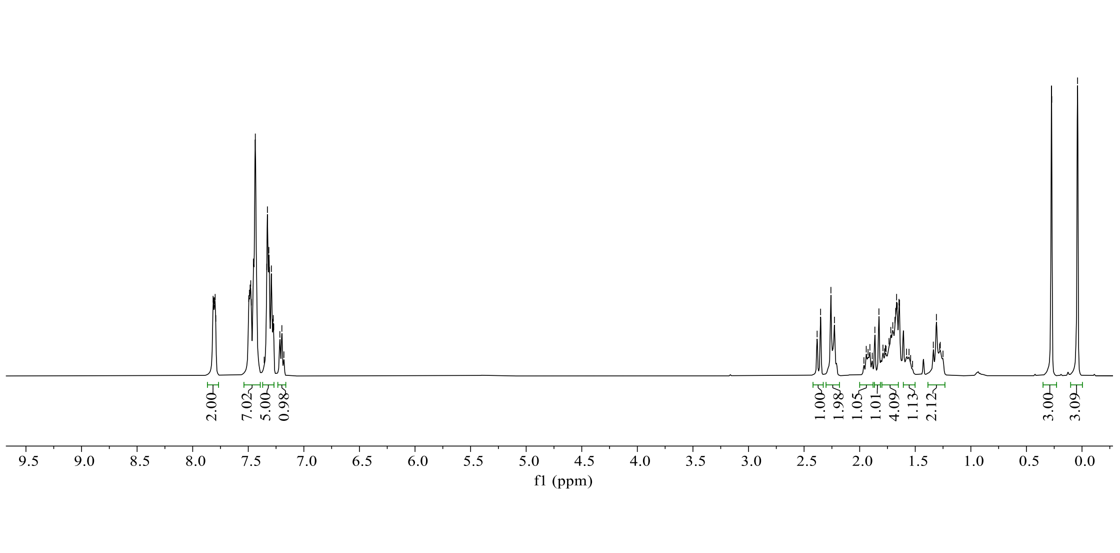

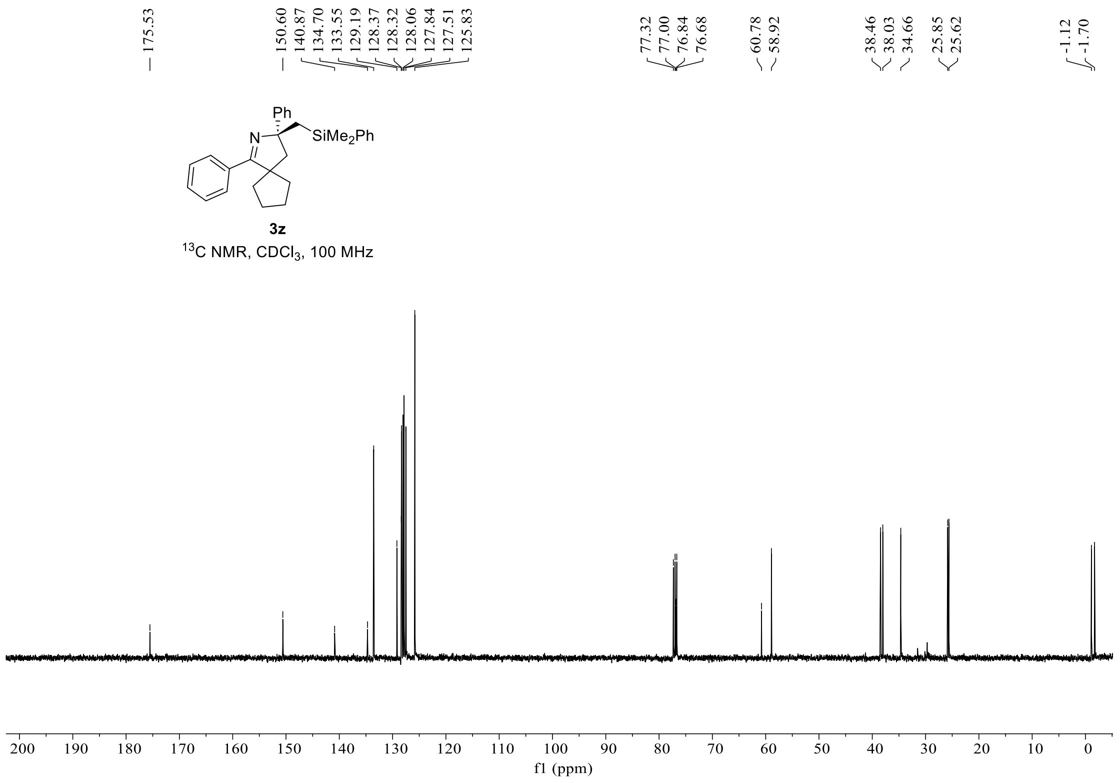
175.53
150.60 140.87 134.70 133.55 129.19 128.37 128.32 128.06 127.84 127.51 125.83
77.32 77.00 76.84 76.68
60.78 58.92
38.46 38.03 34.66 25.85 25.62
-1.12 -1.70
Ph
N
SiMe2Ph
3z
13C NMR, CDCl3, 100 MHz
f1 (ppm)

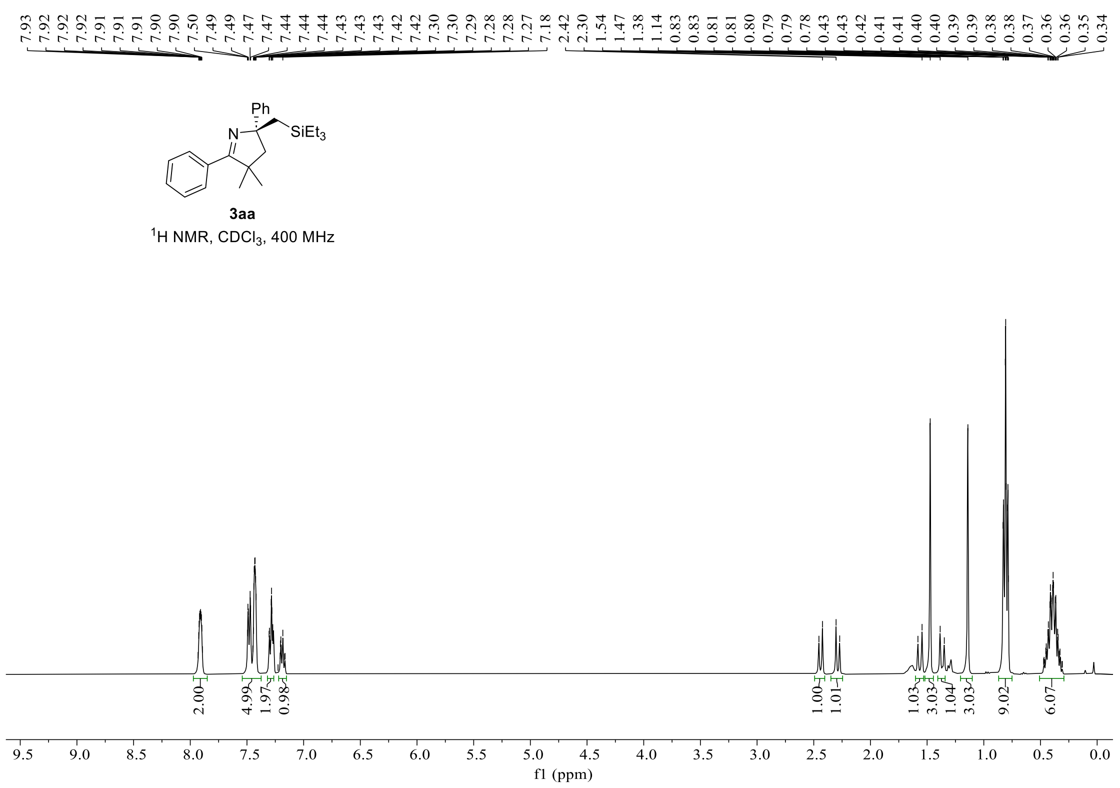
7.93 7.92 7.92 7.92 7.91 7.91 7.91 7.90 7.90 7.50 7.49 7.49 7.47 7.47 7.44 7.44 7.44 7.43 7.43 7.42 7.42 7.30 7.30 7.29 7.28 7.28 7.27 7.18
2.42 2.30 1.54 1.47 1.38 1.14 0.83 0.83 0.81 0.81 0.80 0.79 0.79 0.78 0.43 0.43 0.42 0.41 0.41 0.40 0.40 0.39 0.39 0.38 0.38 0.37 0.36 0.36 0.35 0.34
Ph
N
SiEt3
3aa
1H NMR, CDCl3, 400 MHz
2.00 4.99 1.97 0.98 1.00 1.01 1.03 3.03 1.04 3.03 9.02 6.07
f1 (ppm)

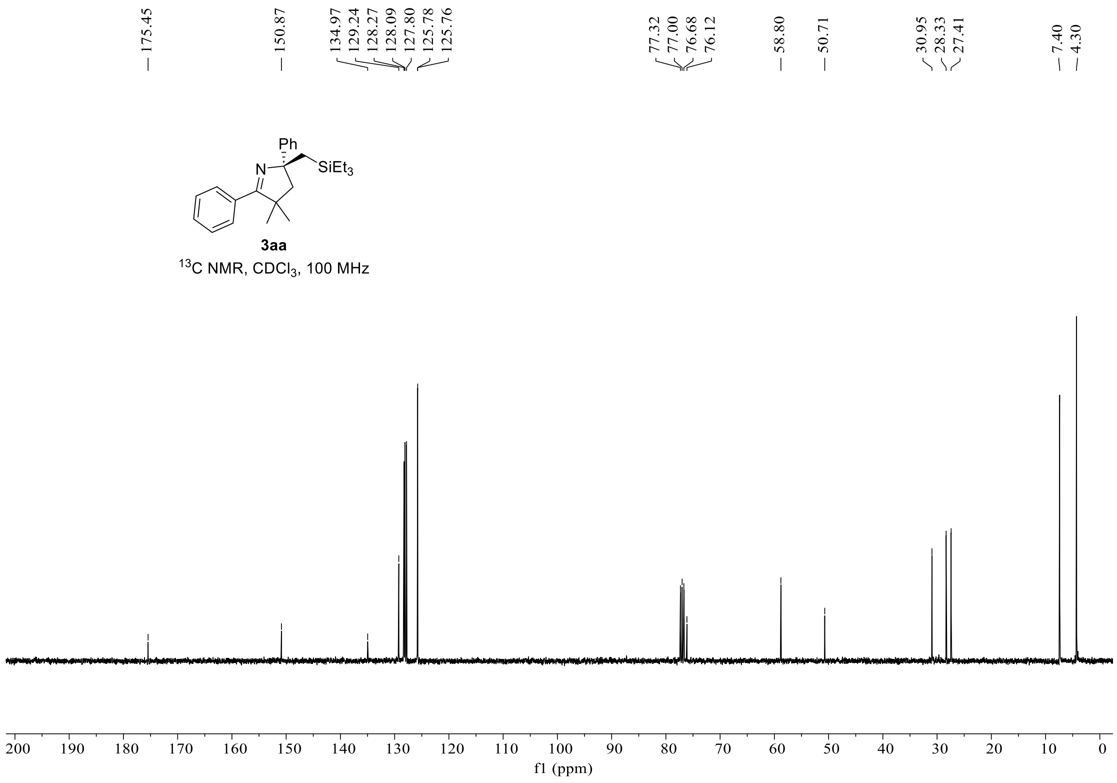
175.45
150.87
134.97
129.24
128.27
128.09
127.80
125.78
125.76
77.32
77.00
76.68
76.12
58.80
50.71
30.95
28.33
27.41
7.40
4.30
Ph
N
SiEt3
3aa
13C NMR, CDCl3, 100 MHz
f1 (ppm)

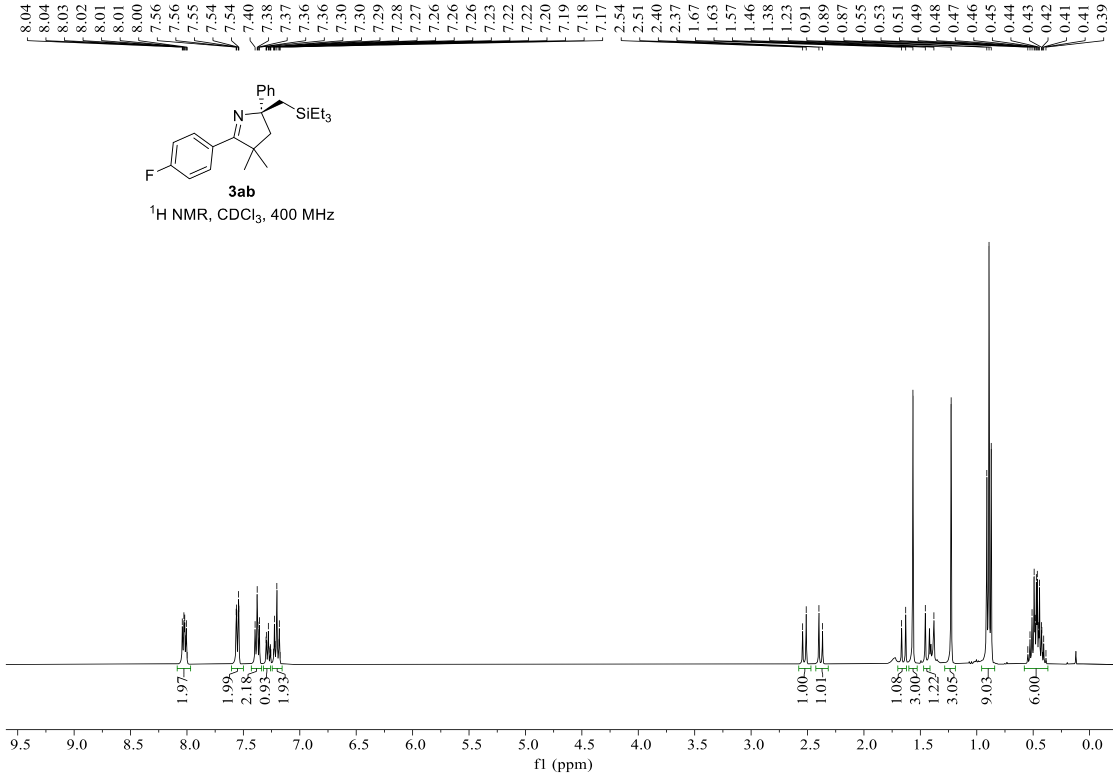
8.04
8.04
8.03
8.02
8.01
8.01
8.00
7.56
7.56
7.55
7.54
7.54
7.40
7.38
7.37
7.36
7.36
7.30
7.30
7.29
7.28
7.27
7.26
7.26
7.26
7.23
7.22
7.22
7.20
7.19
7.18
7.17
2.54
2.51
2.40
2.37
1.67
1.63
1.57
1.46
1.38
1.23
0.91
0.89
0.87
0.55
0.53
0.51
0.49
0.48
0.47
0.46
0.45
0.44
0.43
0.42
0.41
0.41
0.39
Ph
N
SiEt3
F
3ab
1H NMR, CDCl3, 400 MHz
1.97
1.99
2.18
0.93
1.93
1.00
1.01
1.08
3.00
1.22
3.05
9.03
6.00
f1 (ppm)

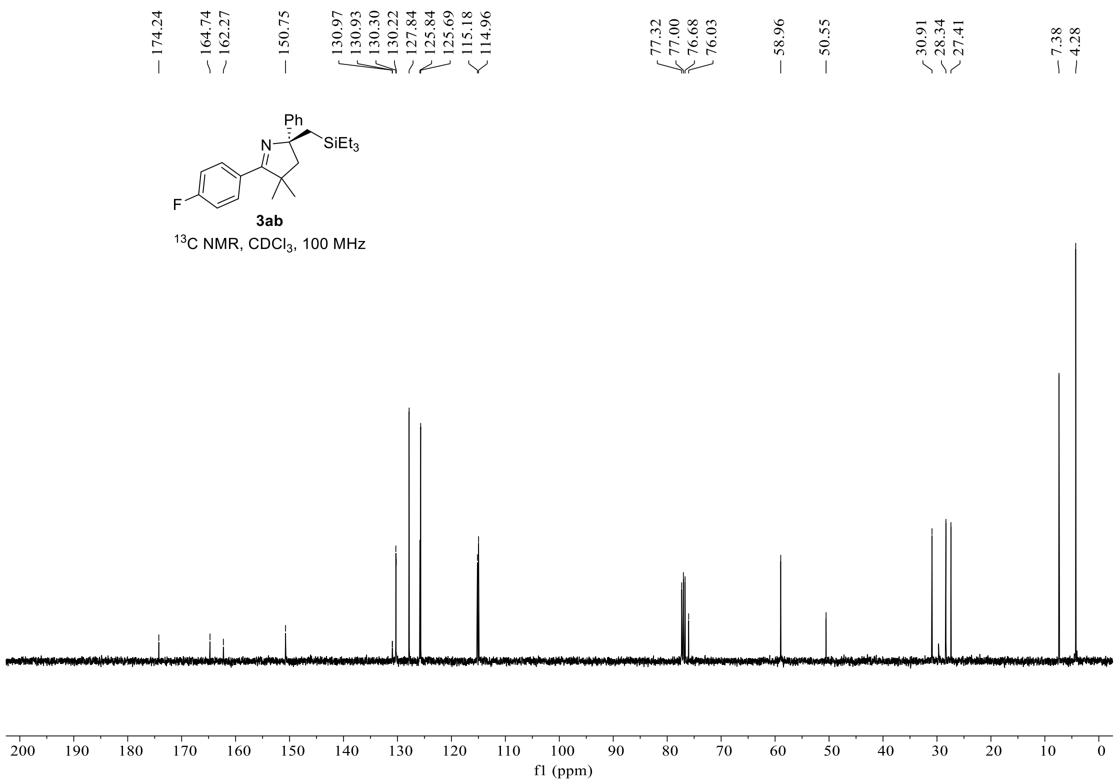
174.24
164.74
162.27
150.75
130.97
130.93
130.30
130.22
127.84
125.84
125.69
115.18
114.96
77.32
77.00
76.68
76.03
58.96
50.55
30.91
28.34
27.41
7.38
4.28
Ph
N
SiEt3
F
3ab
13C NMR, CDCl3, 100 MHz
f1 (ppm)

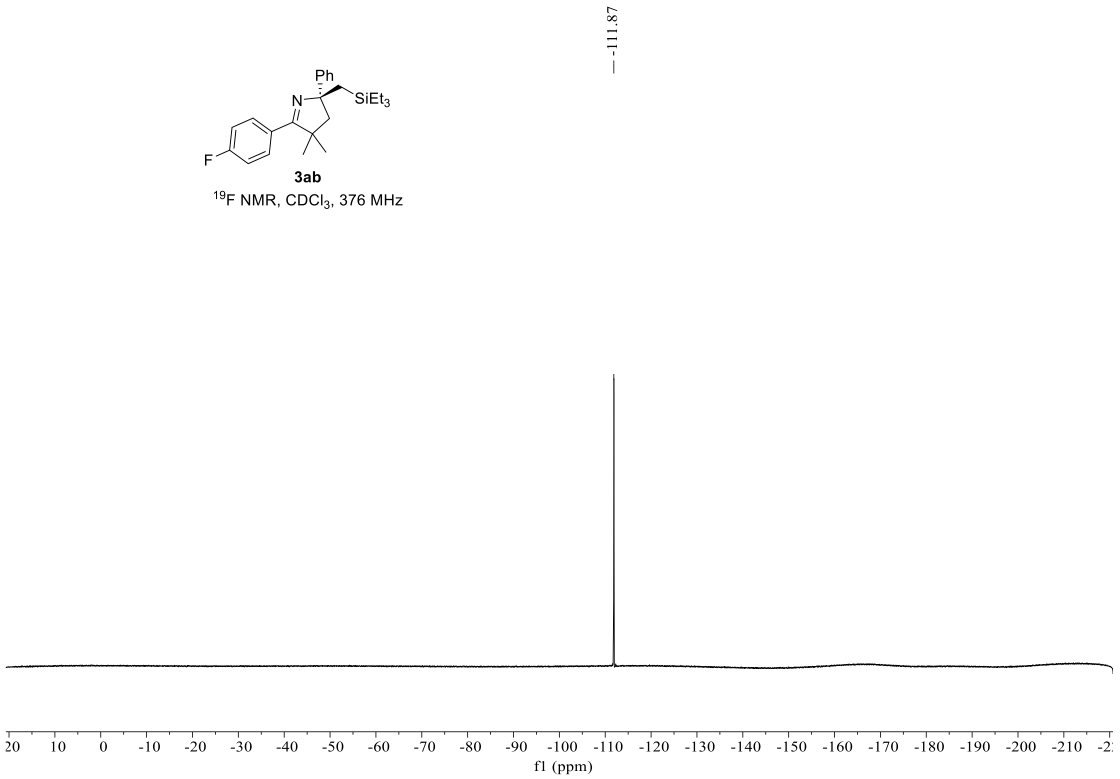
-111.87
Ph
N
SiEt3
F
3ab
19F NMR, CDCl3, 376 MHz
f1 (ppm)

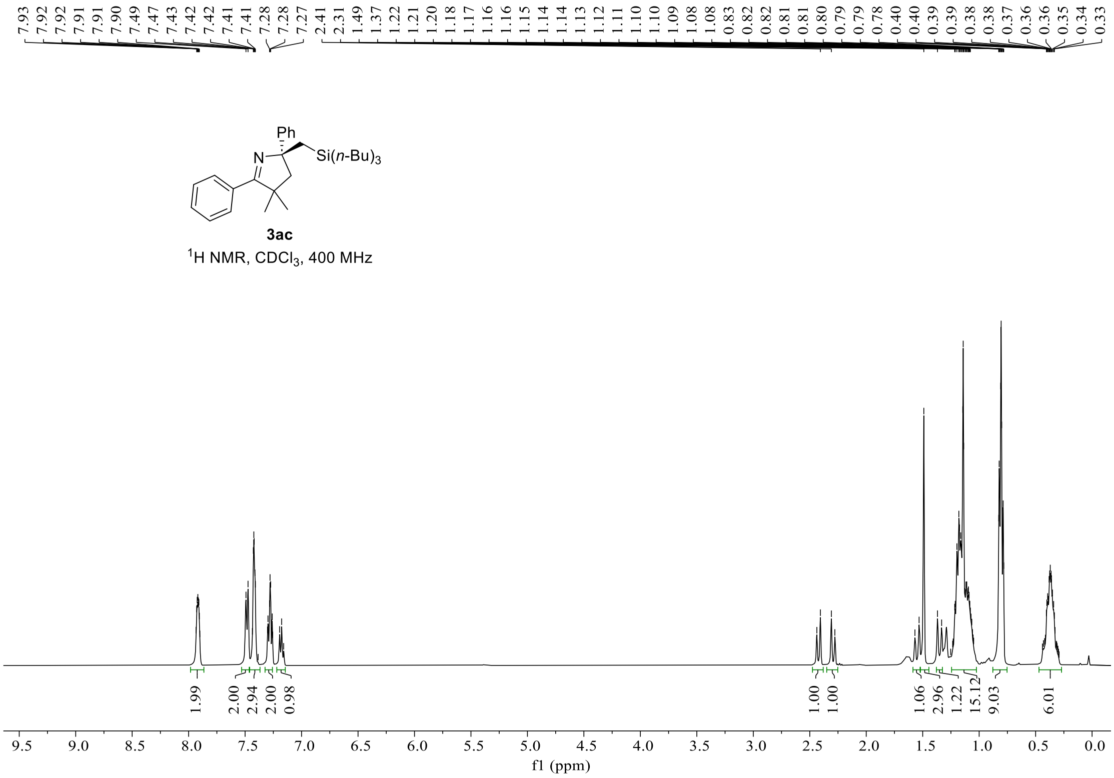
7.93 7.92 7.92 7.91 7.91 7.90 7.49 7.47 7.43 7.42 7.42 7.41 7.41 7.28 7.28 7.27 2.41 2.31 1.49 1.37 1.22 1.21 1.20 1.18 1.17 1.16 1.16 1.15 1.14 1.14 1.13 1.12 1.11 1.10 1.10 1.09 1.08 1.08 0.83 0.82 0.82 0.81 0.81 0.80 0.79 0.79 0.78 0.40 0.40 0.39 0.39 0.38 0.38 0.37 0.36 0.36 0.35 0.34 0.33
Ph
N
Si(n-Bu)3
3ac
1H NMR, CDCl3, 400 MHz
1.99 2.00 2.94 2.00 0.98 1.00 1.00 1.06 2.96 1.22 15.12 9.03 6.01
f1 (ppm)

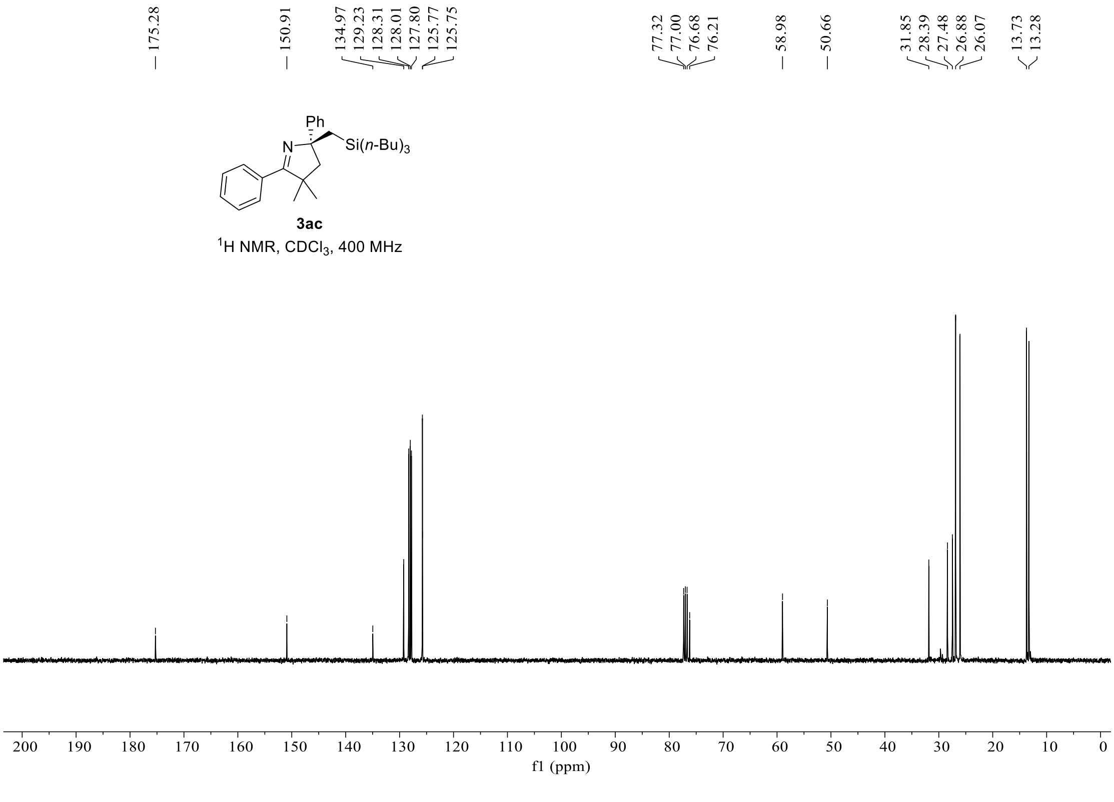
175.28 150.91 134.97 129.23 128.31 128.01 127.80 125.77 125.75 77.32 77.00 76.68 76.21 58.98 50.66 31.85 28.39 27.48 26.88 26.07 13.73 13.28
Ph
N
Si(n-Bu)3
3ac
1H NMR, CDCl3, 400 MHz
f1 (ppm)

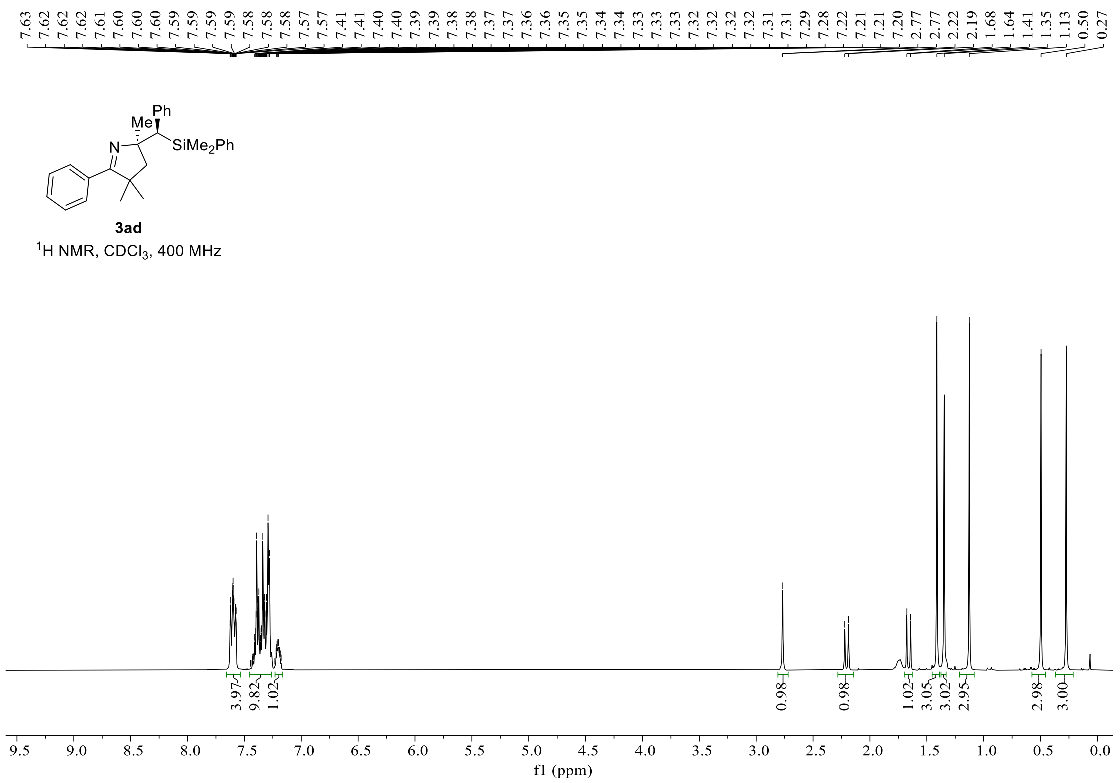

3ad

^{1}H NMR, $CDCl_3$, 400 MHz

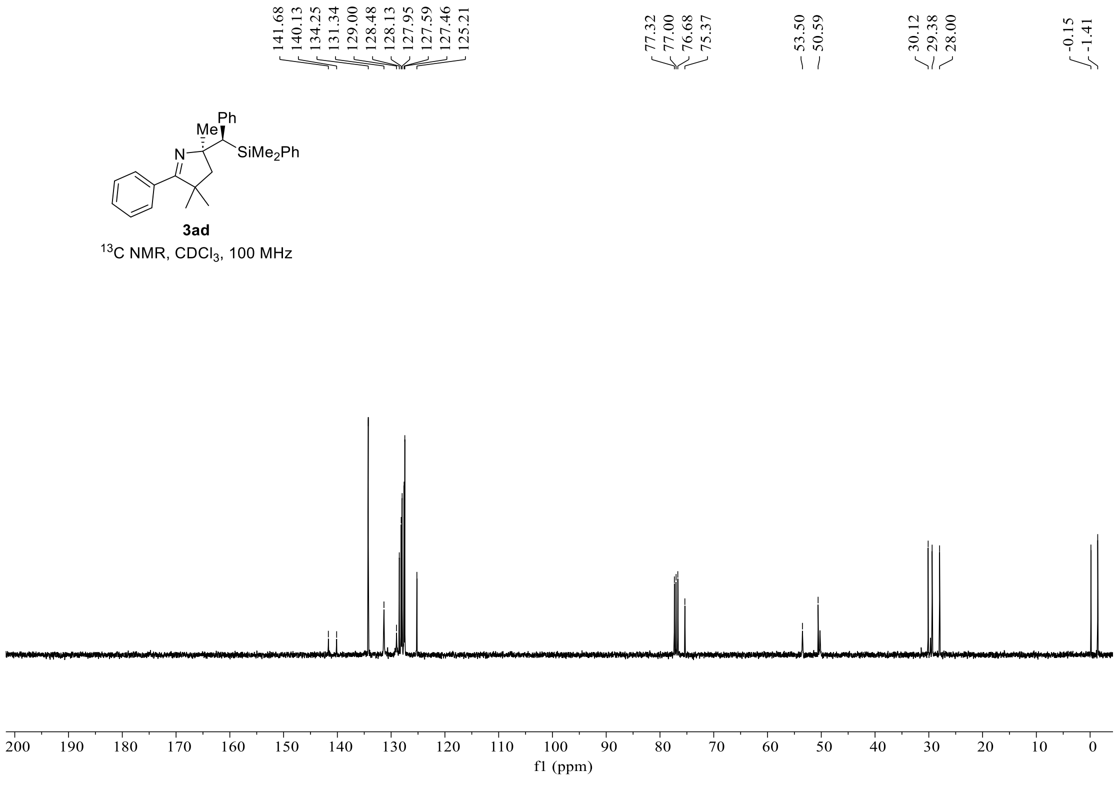

3ad

^{13}C NMR, $CDCl_3$, 100 MHz

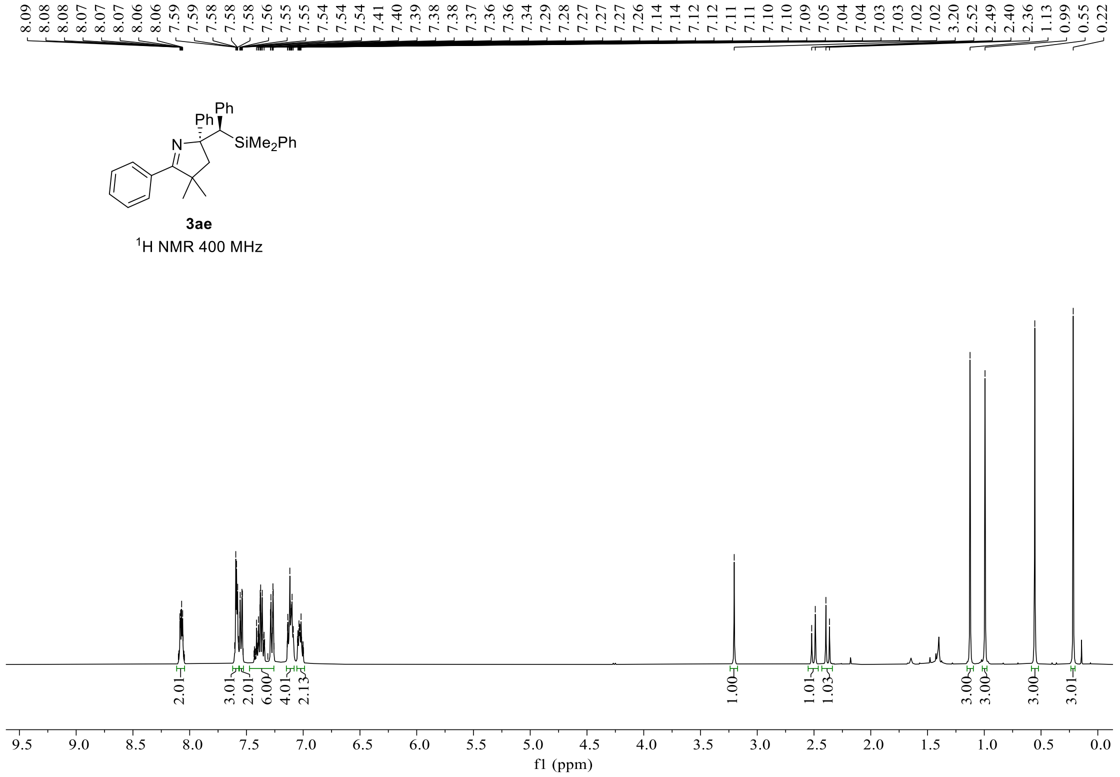
8.09 8.08 8.08 8.07 8.07 8.07 8.06 8.06 7.59 7.59 7.58 7.58 7.58 7.56 7.55 7.55 7.54 7.54 7.54 7.41 7.40 7.39 7.38 7.38 7.37 7.36 7.36 7.34 7.29 7.28 7.27 7.27 7.27 7.26 7.14 7.14 7.12 7.12 7.11 7.11 7.10 7.09 7.05 7.04 7.04 7.03 7.03 7.02 7.02 3.20 2.52 2.49 2.40 2.36 1.13 0.99 0.55 0.22
Ph
Ph
N
SiMe2Ph
3ae
1H NMR 400 MHz
2.01 3.01 2.01 6.00 4.01 2.13 1.00 1.01 1.03 3.00 3.00 3.00 3.01
9.5 9.0 8.5 8.0 7.5 7.0 6.5 6.0 5.5 5.0 4.5 4.0 3.5 3.0 2.5 2.0 1.5 1.0 0.5 0.0
f1 (ppm)

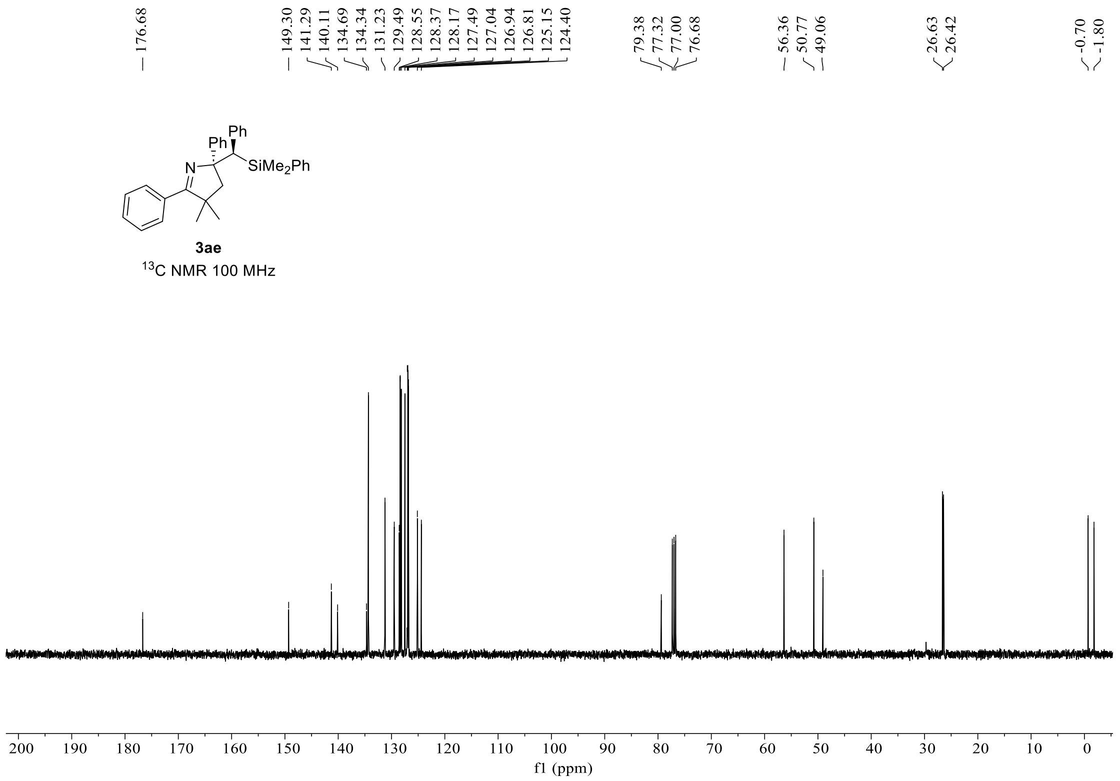
176.68 149.30 141.29 140.11 134.69 134.34 131.23 129.49 128.55 128.37 128.17 127.49 127.04 126.94 126.81 125.15 124.40 79.38 77.32 77.00 76.68 56.36 50.77 49.06 26.63 26.42 -0.70 -1.80
Ph
Ph
N
SiMe2Ph
3ae
13C NMR 100 MHz
200 190 180 170 160 150 140 130 120 110 100 90 80 70 60 50 40 30 20 10 0
f1 (ppm)

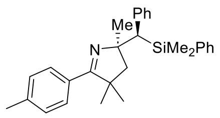

3af

^{1}H NMR, $CDCl_3$, 400 MHz

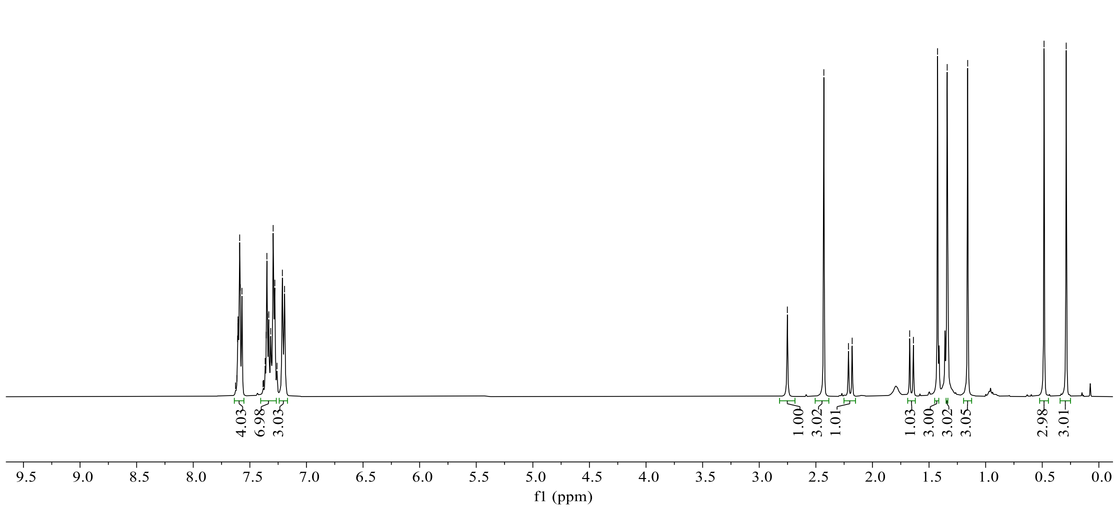

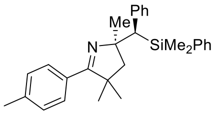

3af

^{13}C NMR, $CDCl_3$, 100 MHz

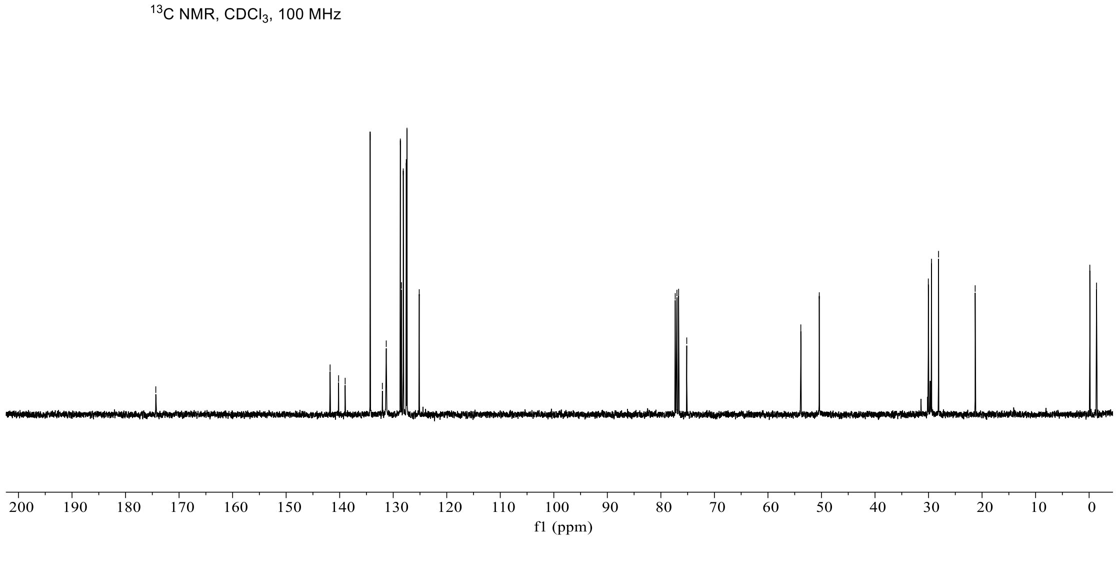

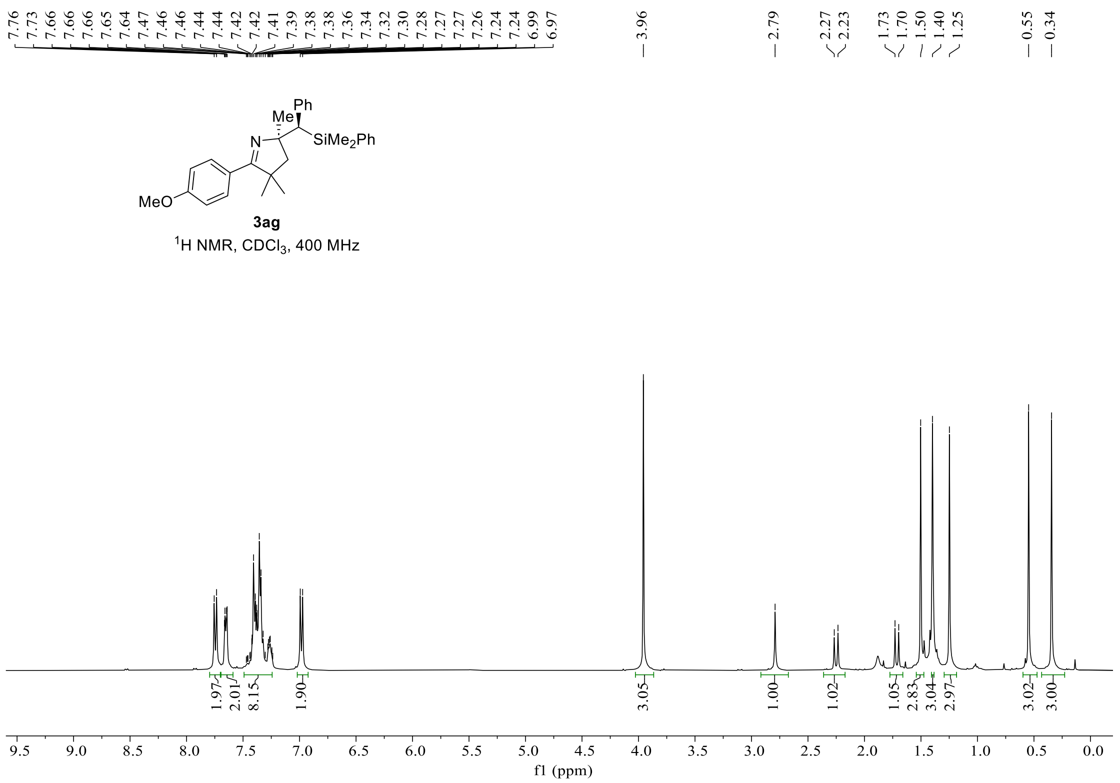
7.76 7.73 7.66 7.66 7.66 7.65 7.64 7.47 7.46 7.46 7.44 7.44 7.42 7.42 7.41 7.39 7.38 7.38 7.36 7.34 7.32 7.30 7.28 7.27 7.27 7.26 7.24 7.24 6.99 6.97
3.96
2.79
2.27 2.23
1.73 1.70 1.50 1.40 1.25
0.55 0.34
Me
Ph
N
SiMe2Ph
MeO
3ag
1H NMR, CDCl3, 400 MHz
1.97 2.01 8.15 1.90 3.05 1.00 1.02 1.05 2.83 3.04 2.97 3.02 3.00
9.5 9.0 8.5 8.0 7.5 7.0 6.5 6.0 5.5 5.0 4.5 4.0 3.5 3.0 2.5 2.0 1.5 1.0 0.5 0.0
f1 (ppm)

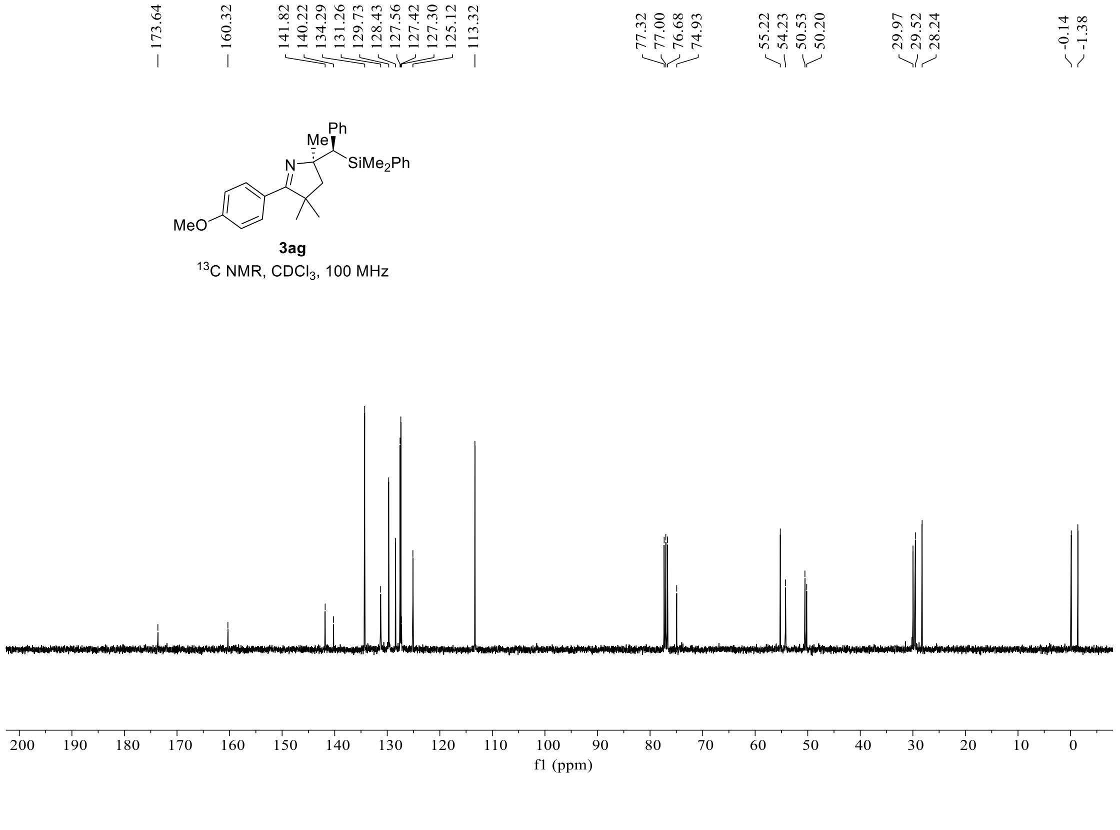
173.64
160.32
141.82 140.22 134.29 131.26 129.73 128.43 127.56 127.42 127.30 125.12 113.32
77.32 77.00 76.68 74.93
55.22 54.23 50.53 50.20
29.97 29.52 28.24
-0.14 -1.38
Me
Ph
N
SiMe2Ph
MeO
3ag
13C NMR, CDCl3, 100 MHz
200 190 180 170 160 150 140 130 120 110 100 90 80 70 60 50 40 30 20 10 0
f1 (ppm)

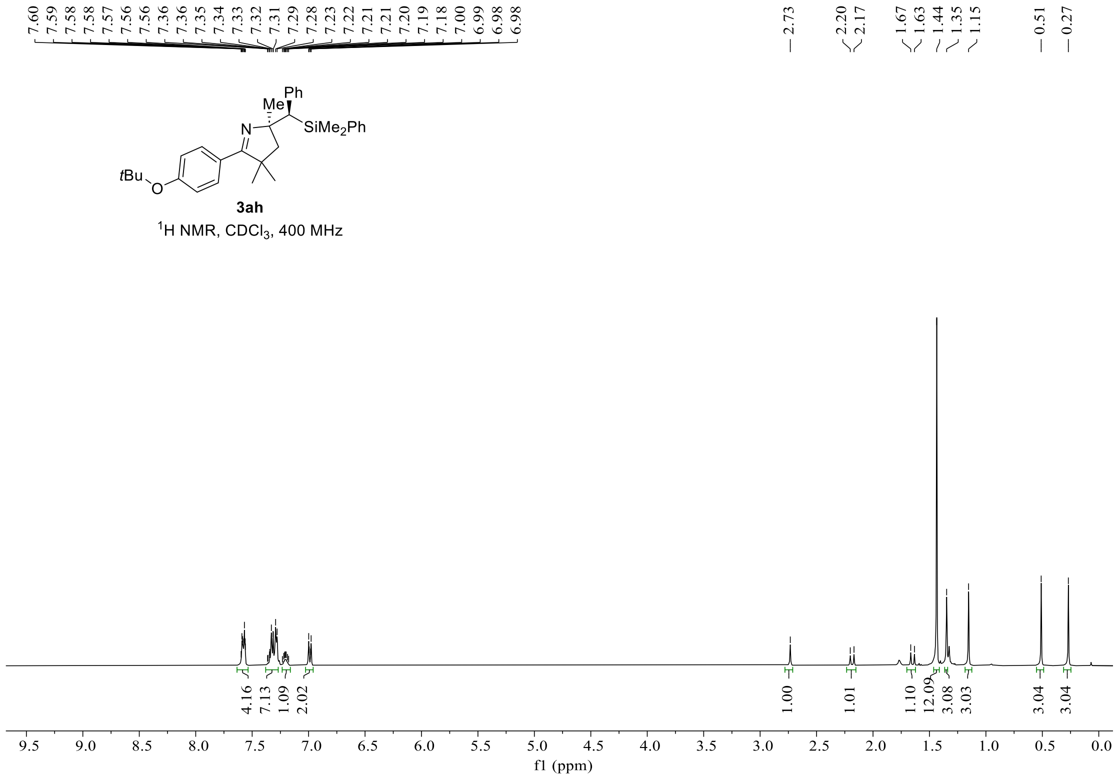
7.60 7.59 7.58 7.58 7.57 7.56 7.56 7.36 7.36 7.35 7.34 7.33 7.32 7.31 7.29 7.28 7.23 7.22 7.21 7.21 7.20 7.19 7.18 7.00 6.99 6.98 6.98
2.73 2.20 2.17 1.67 1.63 1.44 1.35 1.15 0.51 0.27
Ph
Me
N
SiMe2Ph
tBu
O
3ah
1H NMR, CDCl3, 400 MHz
4.16 7.13 1.09 2.02 1.00 1.01 1.10 12.09 3.08 3.03 3.04 3.04
9.5 9.0 8.5 8.0 7.5 7.0 6.5 6.0 5.5 5.0 4.5 4.0 3.5 3.0 2.5 2.0 1.5 1.0 0.5 0.0
f1 (ppm)

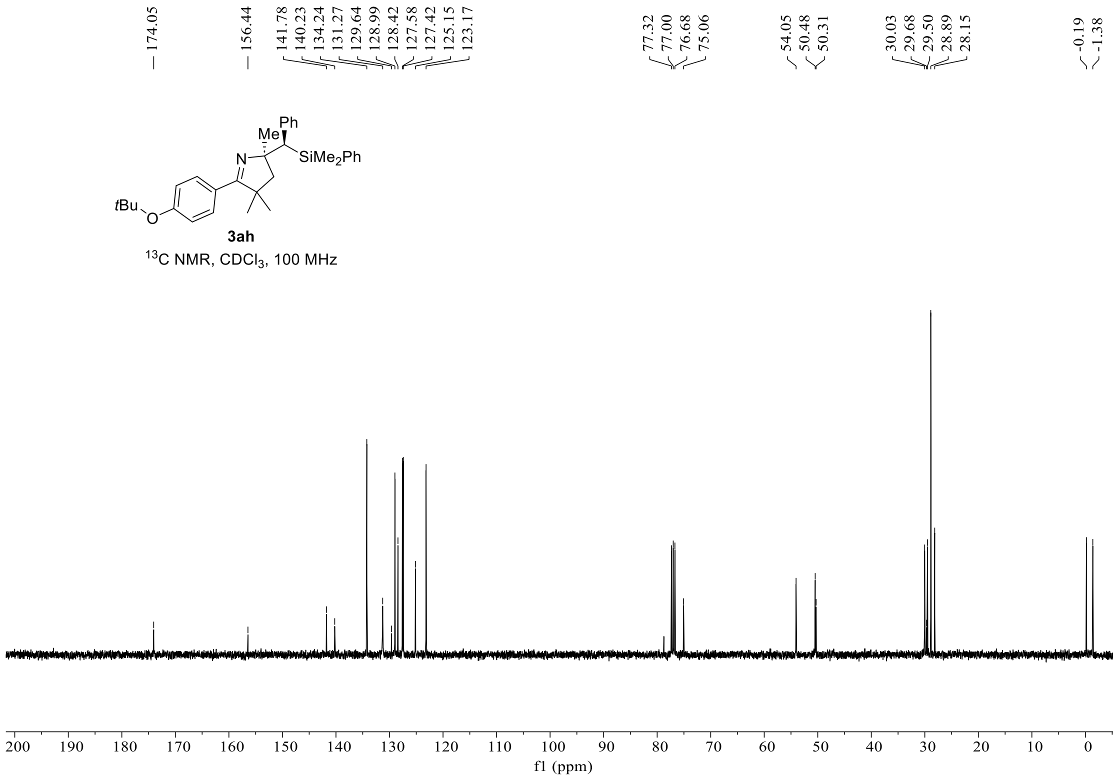
174.05 156.44 141.78 140.23 134.24 131.27 129.64 128.99 128.42 127.58 127.42 125.15 123.17
77.32 77.00 76.68 75.06 54.05 50.48 50.31 30.03 29.68 29.50 28.89 28.15 -0.19 -1.38
Ph
Me
N
SiMe2Ph
tBu
O
3ah
13C NMR, CDCl3, 100 MHz
200 190 180 170 160 150 140 130 120 110 100 90 80 70 60 50 40 30 20 10 0
f1 (ppm)

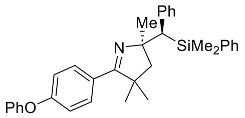

3ai

^{1}H NMR, $CDCl_3$, 400 MHz

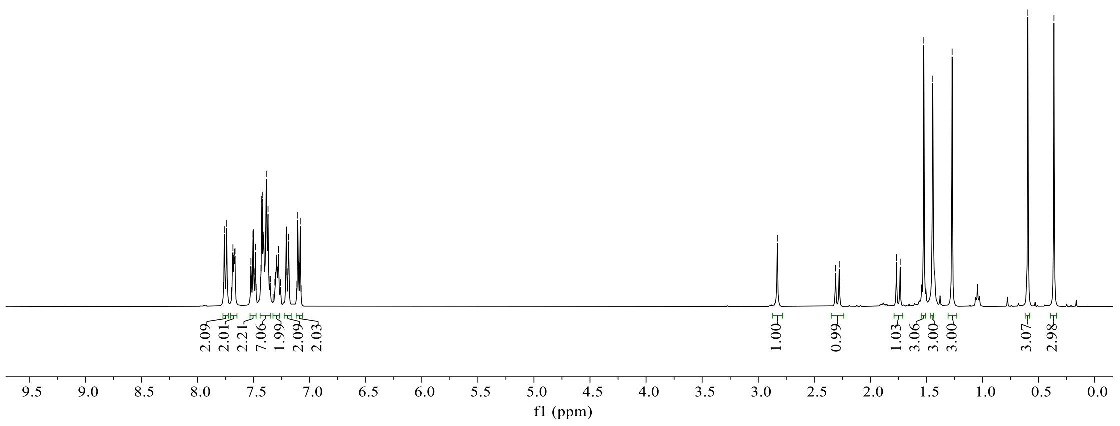

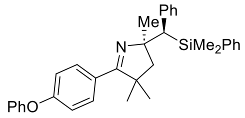

3ai

^{13}C NMR, $CDCl_3$, 100 MHz

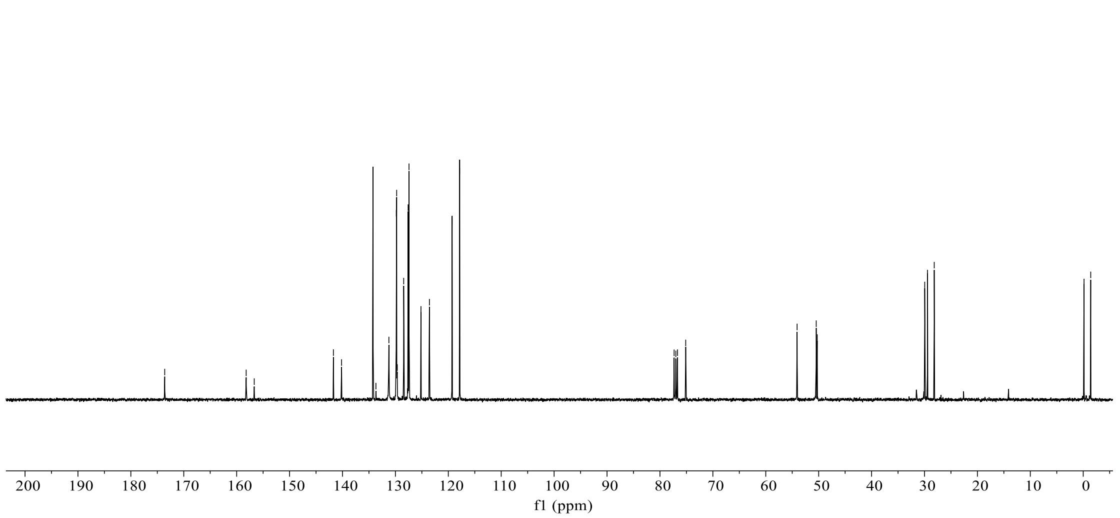

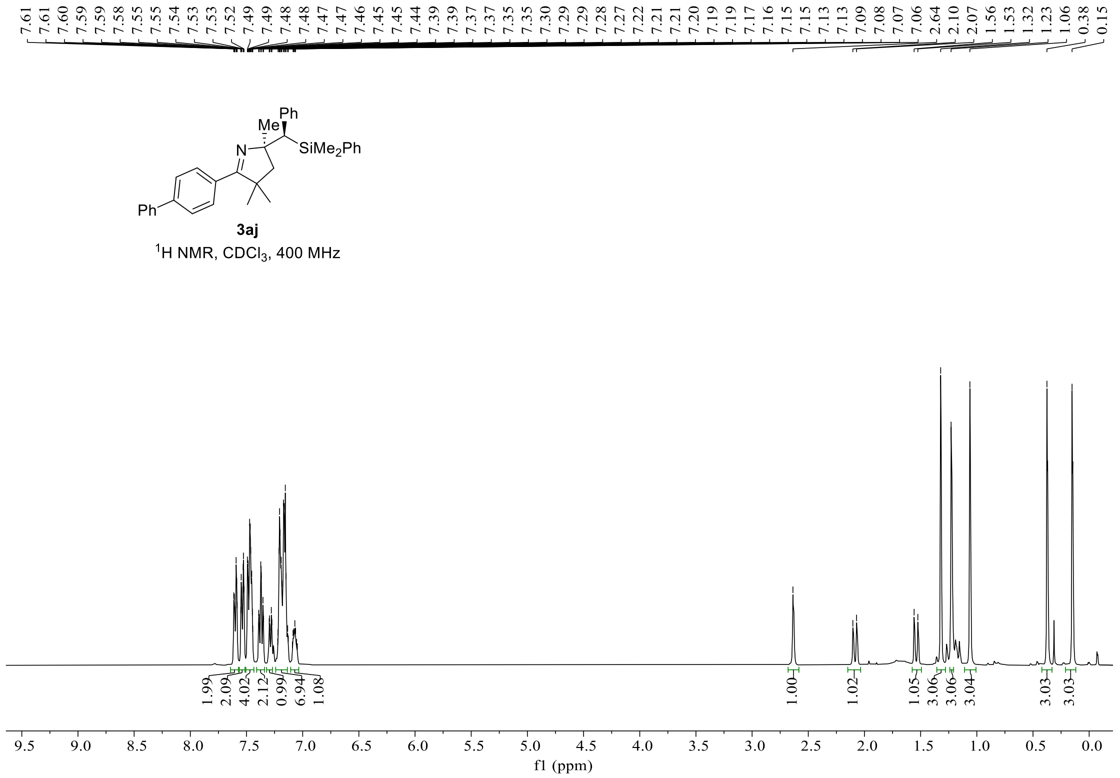
7.61 7.61 7.60 7.59 7.59 7.58 7.55 7.55 7.54 7.53 7.53 7.52 7.49 7.49 7.48 7.48 7.47 7.47 7.46 7.45 7.45 7.44 7.39 7.39 7.37 7.37 7.35 7.35 7.30 7.29 7.29 7.28 7.27 7.22 7.21 7.21 7.20 7.19 7.19 7.17 7.16 7.15 7.15 7.13 7.13 7.09 7.08 7.07 7.06 2.64 2.10 2.07 1.56 1.53 1.32 1.23 1.06 0.38 0.15
Ph
Me
N
SiMe2Ph
Ph
3aj
1H NMR, CDCl3, 400 MHz
1.99 2.09 4.02 2.12 0.99 6.94 1.08
1.00
1.02
1.05 3.06 3.06 3.04
3.03 3.03
9.5 9.0 8.5 8.0 7.5 7.0 6.5 6.0 5.5 5.0 4.5 4.0 3.5 3.0 2.5 2.0 1.5 1.0 0.5 0.0
f1 (ppm)

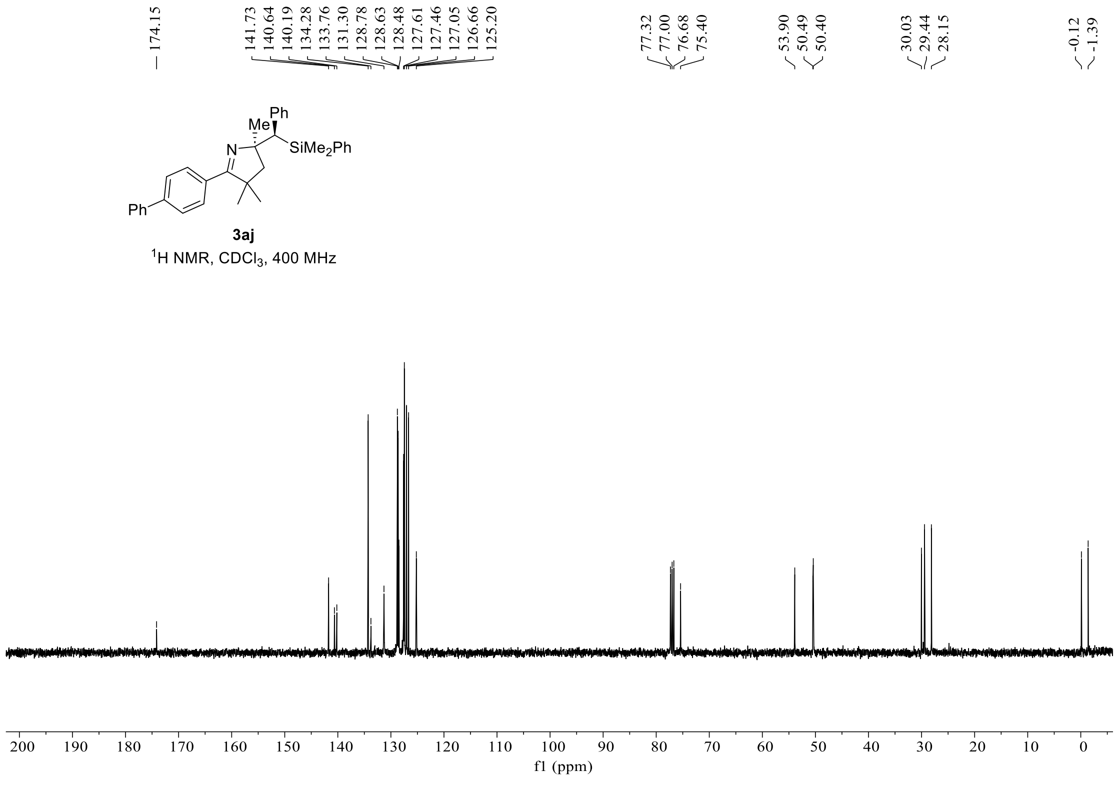
174.15
141.73 140.64 140.19 134.28 133.76 131.30 128.78 128.63 128.48 127.61 127.46 127.05 126.66 125.20
77.32 77.00 76.68 75.40
53.90 50.49 50.40
30.03 29.44 28.15
-0.12 -1.39
Ph
Me
N
SiMe2Ph
Ph
3aj
1H NMR, CDCl3, 400 MHz
200 190 180 170 160 150 140 130 120 110 100 90 80 70 60 50 40 30 20 10 0
f1 (ppm)

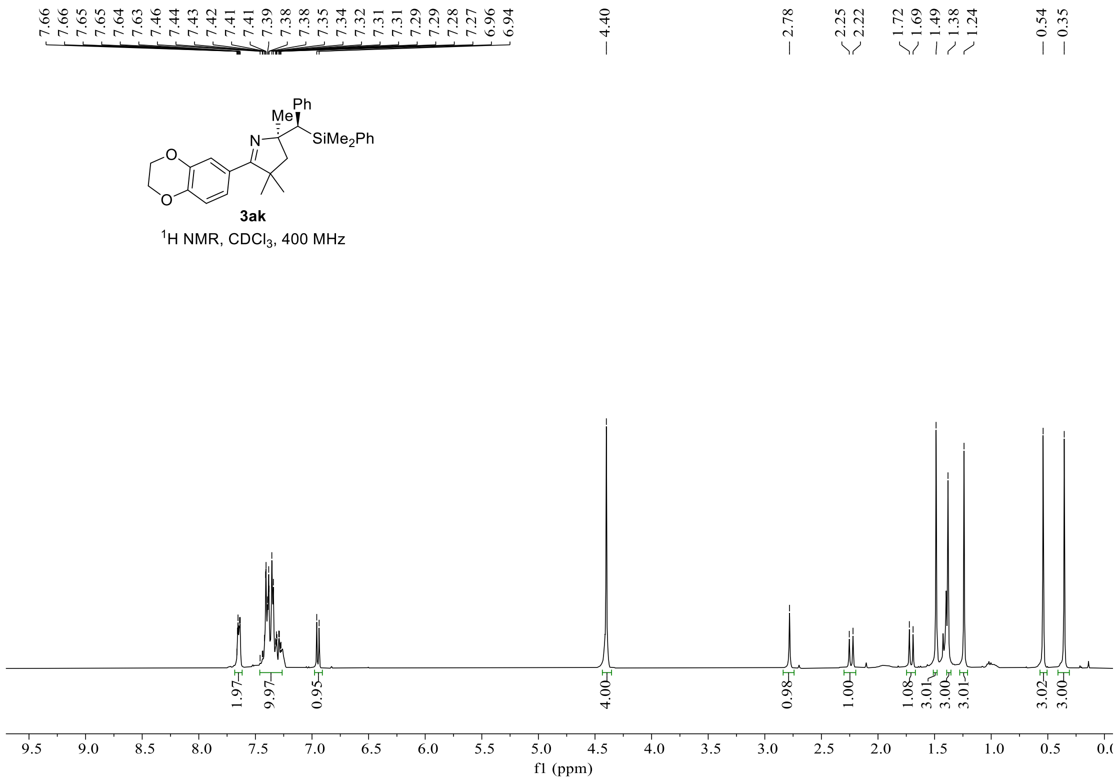
7.66 7.66 7.65 7.65 7.64 7.63 7.46 7.44 7.43 7.42 7.41 7.39 7.38 7.38 7.35 7.34 7.32 7.31 7.31 7.29 7.28 7.27 6.96 6.94
4.40
2.78
2.25 2.22
1.72 1.69 1.49 1.38 1.24
0.54 0.35
Ph
Me
N
SiMe2Ph
O
O
3ak
1H NMR, CDCl3, 400 MHz
1.97 9.97 0.95 4.00 0.98 1.00 1.08 3.01 3.00 3.01 3.02 3.00
f1 (ppm)

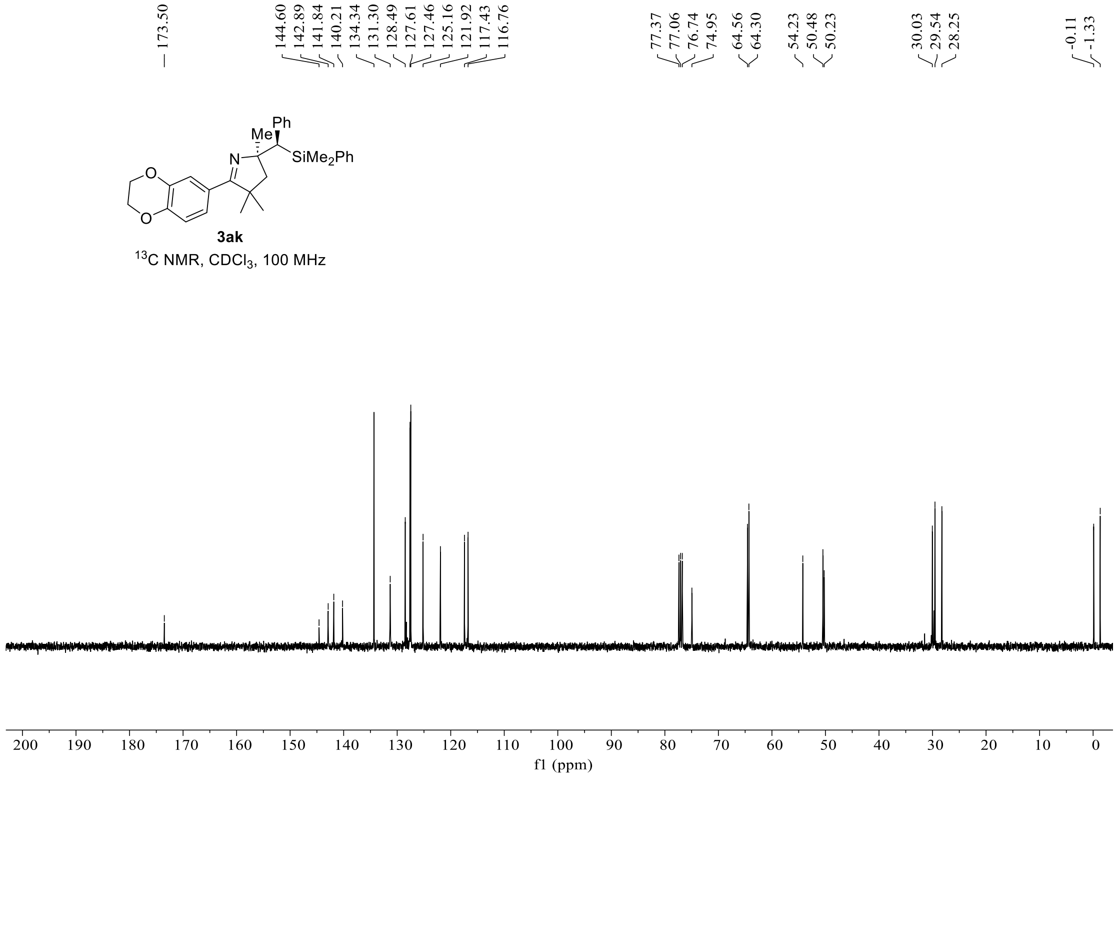
173.50
144.60 142.89 141.84 140.21 134.34 131.30 128.49 127.61 127.46 125.16 121.92 117.43 116.76
77.37 77.06 76.74 74.95 64.56 64.30 54.23 50.48 50.23
30.03 29.54 28.25
-0.11 -1.33
Ph
Me
N
SiMe2Ph
O
O
3ak
13C NMR, CDCl3, 100 MHz
f1 (ppm)

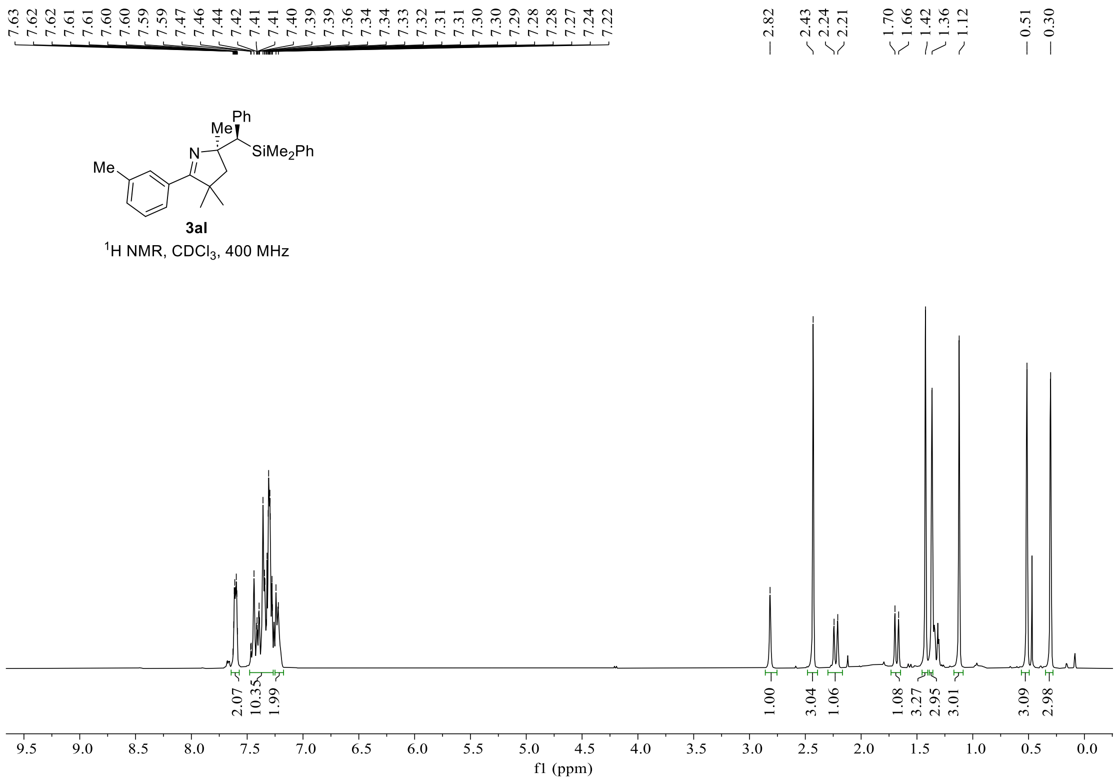

3al

^{1}H NMR, $CDCl_3$, 400 MHz

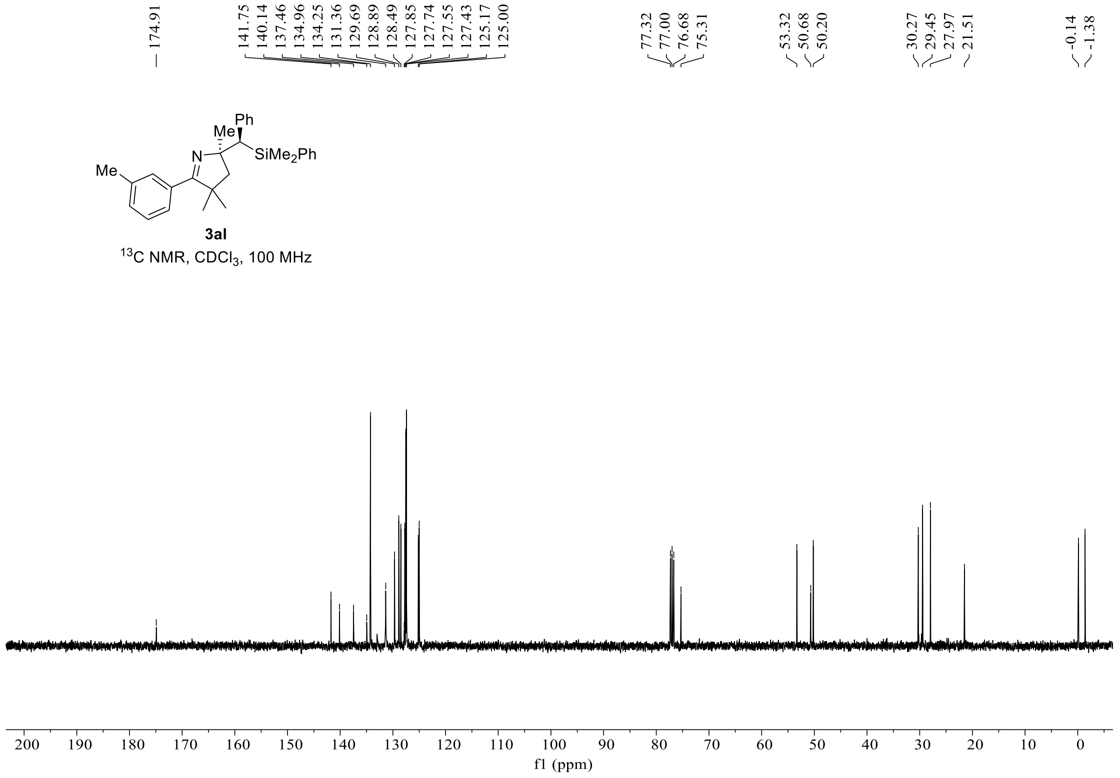

3al

^{13}C NMR, $CDCl_3$, 100 MHz

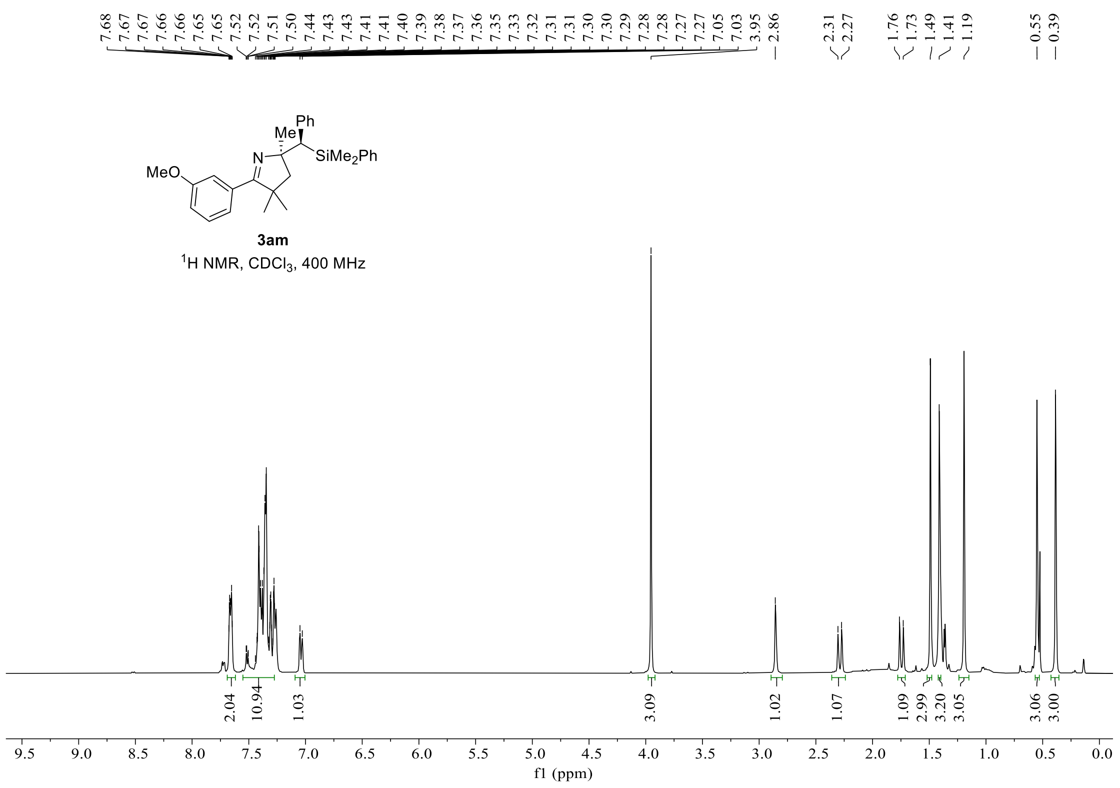
7.68 7.67 7.67 7.66 7.66 7.65 7.65 7.52 7.52 7.51 7.50 7.44 7.43 7.43 7.41 7.41 7.40 7.39 7.38 7.37 7.36 7.35 7.33 7.32 7.31 7.31 7.30 7.30 7.29 7.28 7.28 7.27 7.27 7.05 7.03 3.95 2.86 2.31 2.27 1.76 1.73 1.49 1.41 1.19 0.55 0.39
Ph
Me
SiMe$_2$Ph
N
MeO
3am
^{1}H NMR, CDCl$_3$, 400 MHz
2.04 10.94 1.03 3.09 1.02 1.07 1.09 2.99 3.20 3.05 3.06 3.00
f1 (ppm)

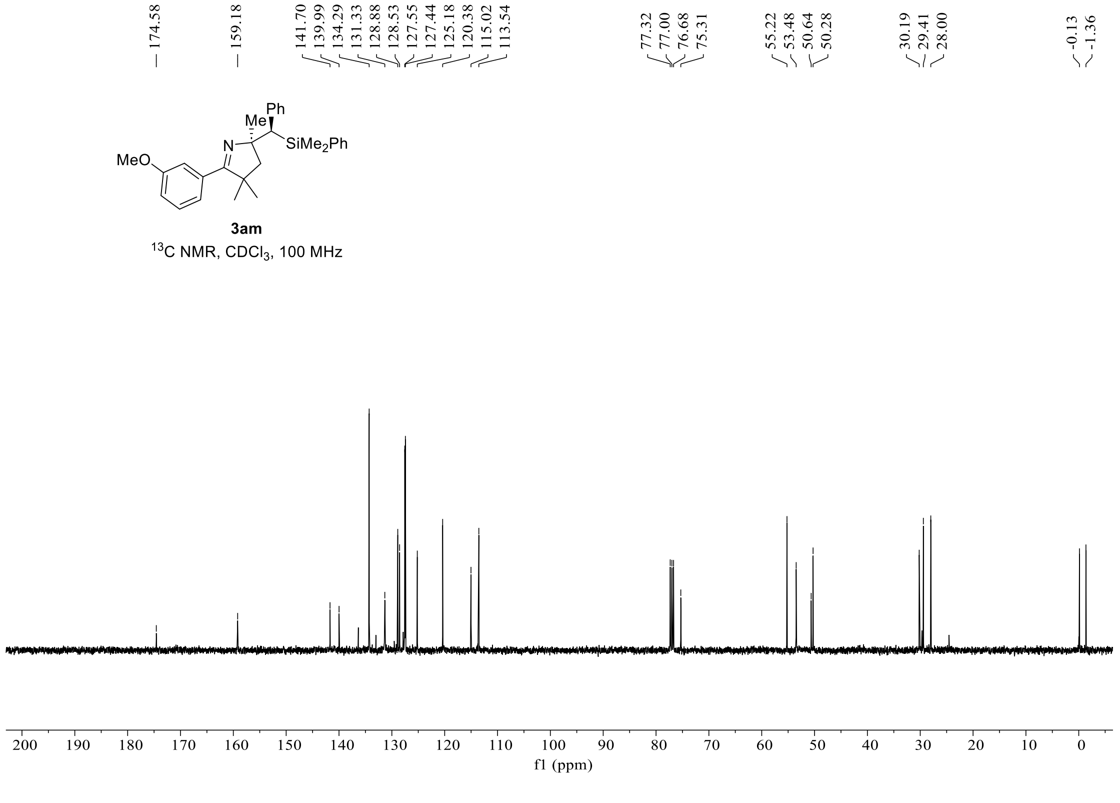
174.58 159.18 141.70 139.99 134.29 131.33 128.88 128.53 127.55 127.44 125.18 120.38 115.02 113.54 77.32 77.00 76.68 75.31 55.22 53.48 50.64 50.28 30.19 29.41 28.00 -0.13 -1.36
Ph
Me
SiMe$_2$Ph
N
MeO
3am
^{13}C NMR, CDCl$_3$, 100 MHz
f1 (ppm)

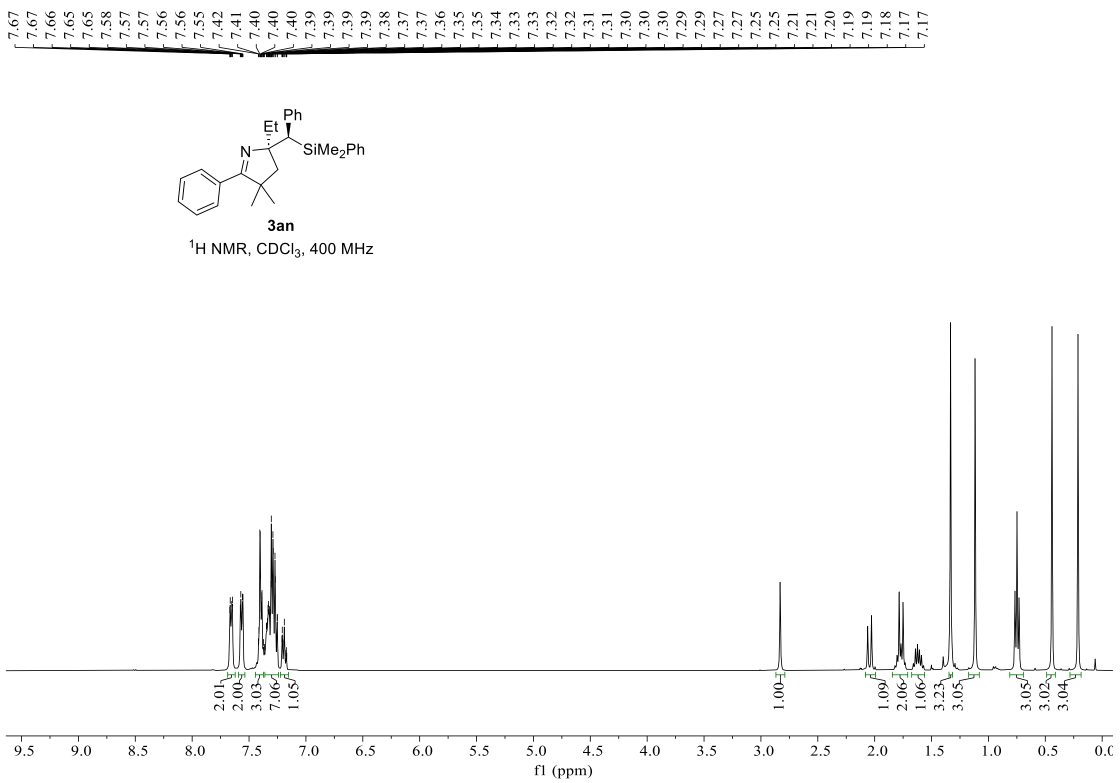
7.67 7.67 7.66 7.65 7.65 7.58 7.57 7.57 7.56 7.56 7.55 7.42 7.41 7.40 7.40 7.40 7.39 7.39 7.39 7.39 7.38 7.37 7.37 7.36 7.35 7.35 7.34 7.33 7.33 7.32 7.32 7.32 7.31 7.31 7.30 7.30 7.29 7.29 7.27 7.27 7.25 7.25 7.21 7.21 7.20 7.19 7.19 7.18 7.17 7.17
Ph
Et
N
SiMe2Ph
3an
1H NMR, CDCl3, 400 MHz
2.01 2.00 3.03 7.06 1.05 1.00 1.09 2.06 1.06 3.23 3.05 3.05 3.02 3.04
f1 (ppm)

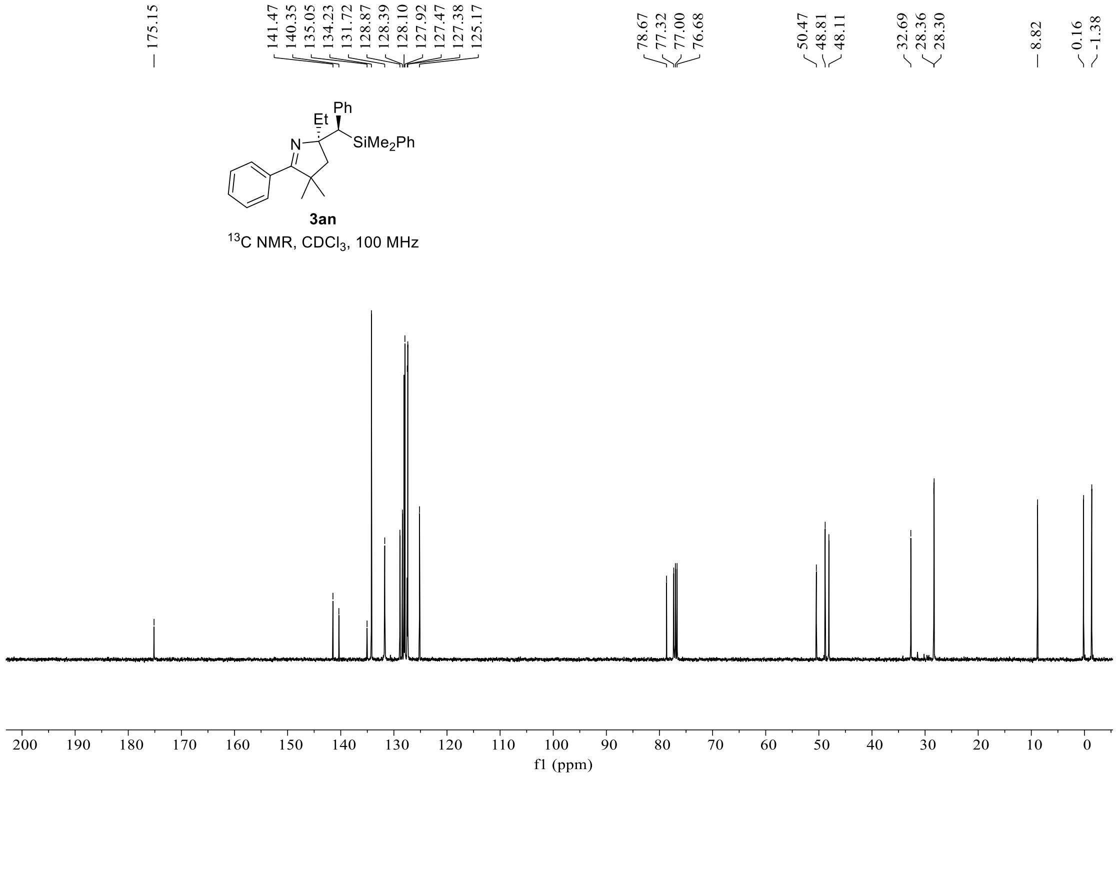
175.15
141.47 140.35 135.05 134.23 131.72 128.87 128.39 128.10 127.92 127.47 127.38 125.17
78.67 77.32 77.00 76.68
50.47 48.81 48.11
32.69 28.36 28.30
8.82
0.16 -1.38
Ph
Et
N
SiMe2Ph
3an
13C NMR, CDCl3, 100 MHz
f1 (ppm)

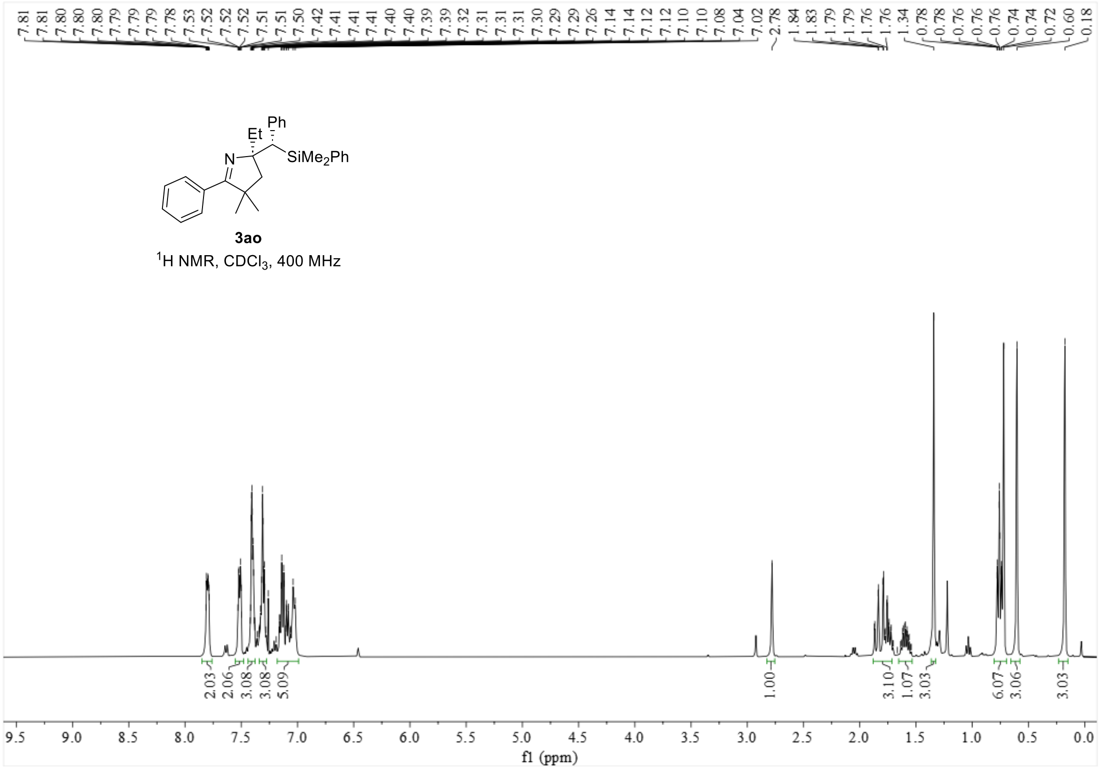
3ao
^{1}H NMR, CDCl$_3$, 400 MHz
f1 (ppm)

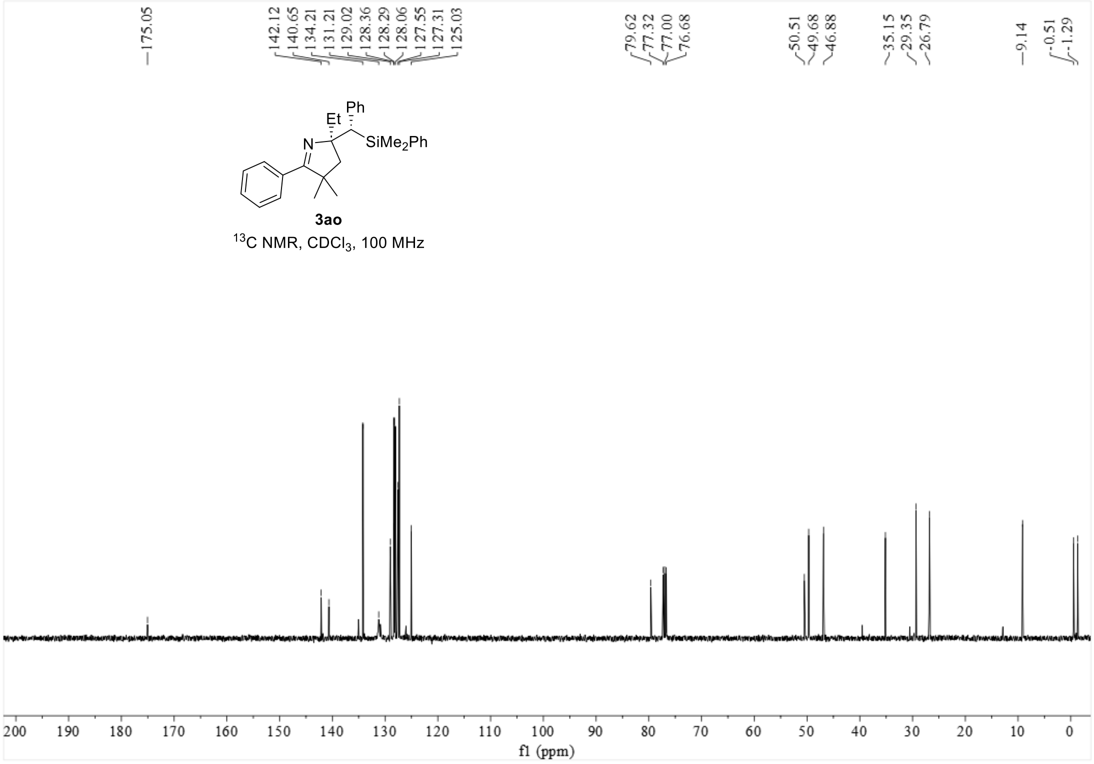
3ao
^{13}C NMR, CDCl$_3$, 100 MHz
f1 (ppm)

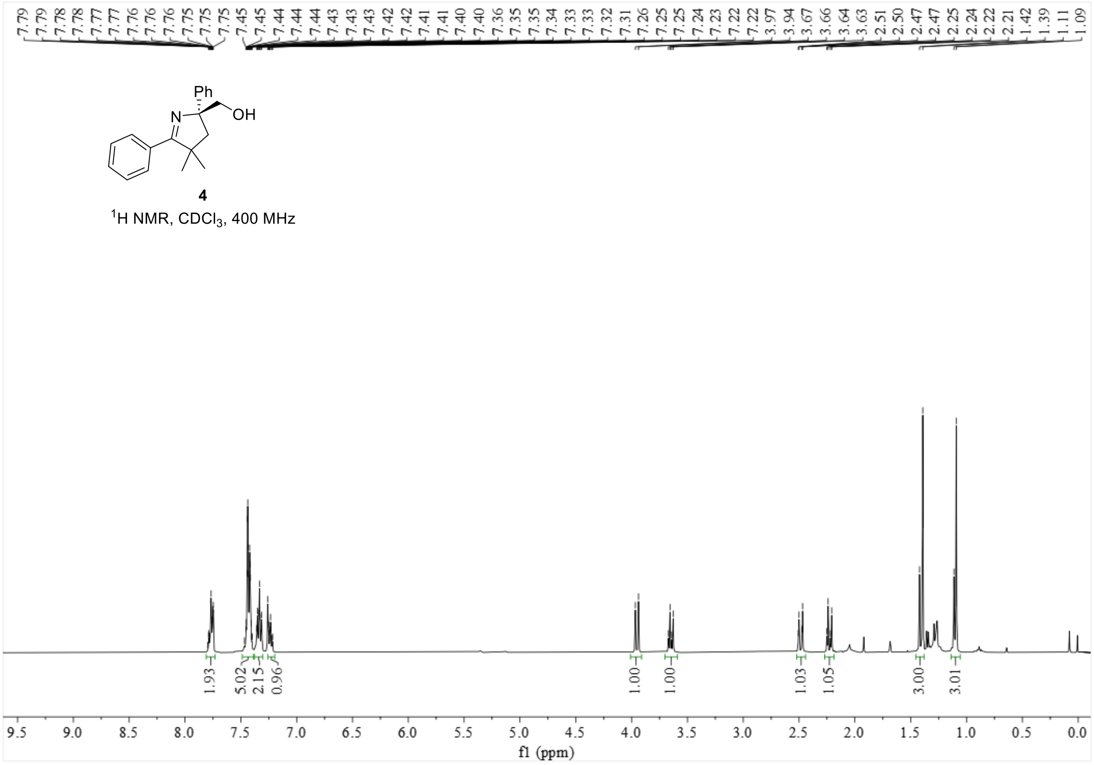

7.79 7.79 7.78 7.78 7.77 7.77 7.76 7.76 7.75 7.75 7.75 7.45 7.45 7.44 7.44 7.44 7.43 7.43 7.43 7.42 7.42 7.41 7.41 7.40 7.40 7.36 7.35 7.35 7.34 7.33 7.33 7.32 7.31 7.26 7.25 7.25 7.24 7.23 7.22 7.22 3.97 3.94 3.67 3.66 3.64 3.63 2.51 2.50 2.47 2.47 2.25 2.24 2.22 2.21 1.42 1.39 1.11 1.09
Ph
N
OH
4
^{1}H NMR, CDCl$_3$, 400 MHz
1.93 5.02 2.15 0.96 1.00 1.00 1.03 1.05 3.00 3.01
9.5 9.0 8.5 8.0 7.5 7.0 6.5 6.0 5.5 5.0 4.5 4.0 3.5 3.0 2.5 2.0 1.5 1.0 0.5 0.0
f1 (ppm)

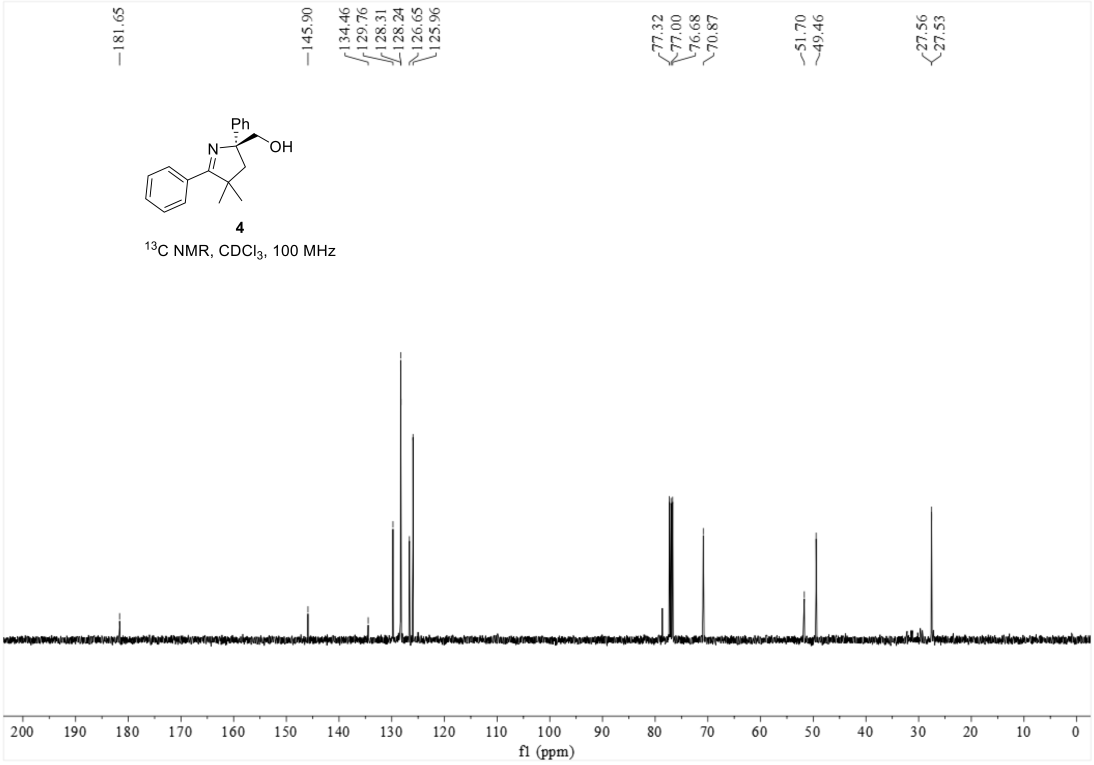

181.65 145.90 134.46 129.76 128.31 128.24 126.65 125.96 77.32 77.00 76.68 70.87 51.70 49.46 27.56 27.53
Ph
N
OH
4
^{13}C NMR, CDCl$_3$, 100 MHz
200 190 180 170 160 150 140 130 120 110 100 90 80 70 60 50 40 30 20 10 0
f1 (ppm)

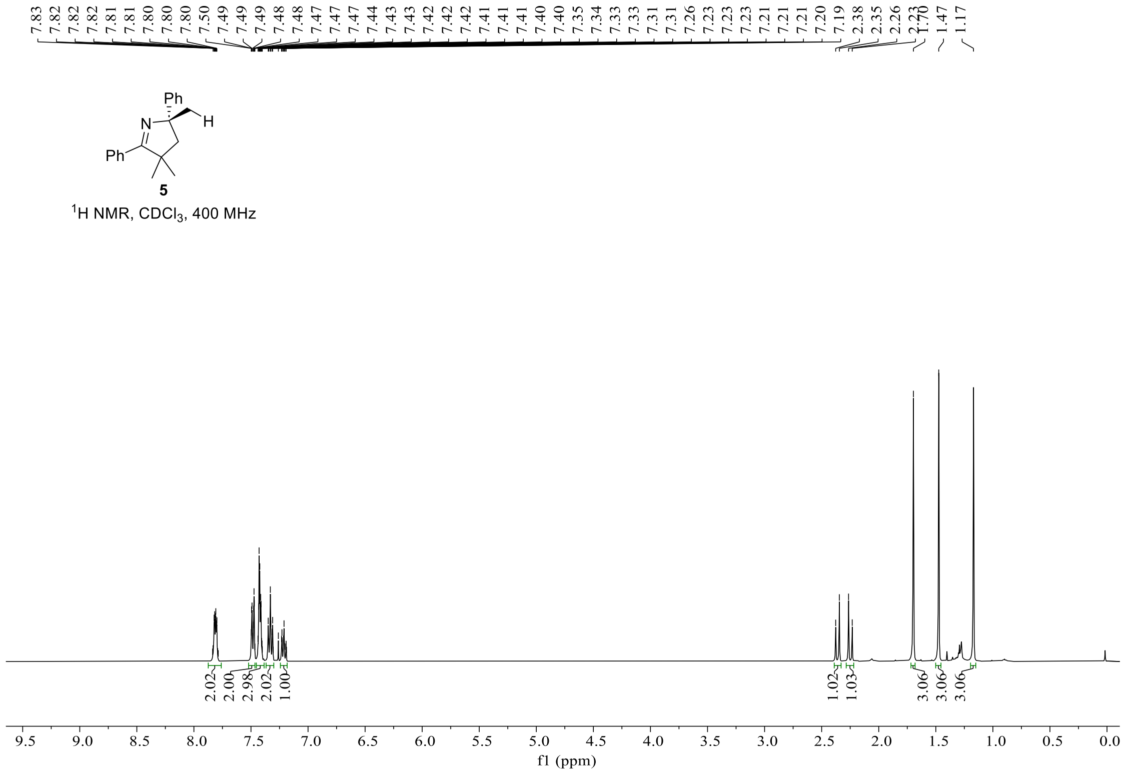
7.83 7.82 7.82 7.82 7.81 7.81 7.80 7.80 7.80 7.50 7.49 7.49 7.49 7.48 7.47 7.47 7.47 7.44 7.43 7.43 7.42 7.42 7.42 7.41 7.41 7.41 7.40 7.40 7.35 7.34 7.33 7.33 7.31 7.31 7.26 7.23 7.23 7.23 7.21 7.21 7.21 7.20 7.19 2.38 2.35 2.26 2.23 1.70 1.47 1.17
Ph
N
H
Ph
5
^{1}H NMR, $CDCl_3$, 400 MHz
2.02 2.00 2.98 2.02 1.00 1.02 1.03 3.06 3.06 3.06
9.5 9.0 8.5 8.0 7.5 7.0 6.5 6.0 5.5 5.0 4.5 4.0 3.5 3.0 2.5 2.0 1.5 1.0 0.5 0.0
f1 (ppm)

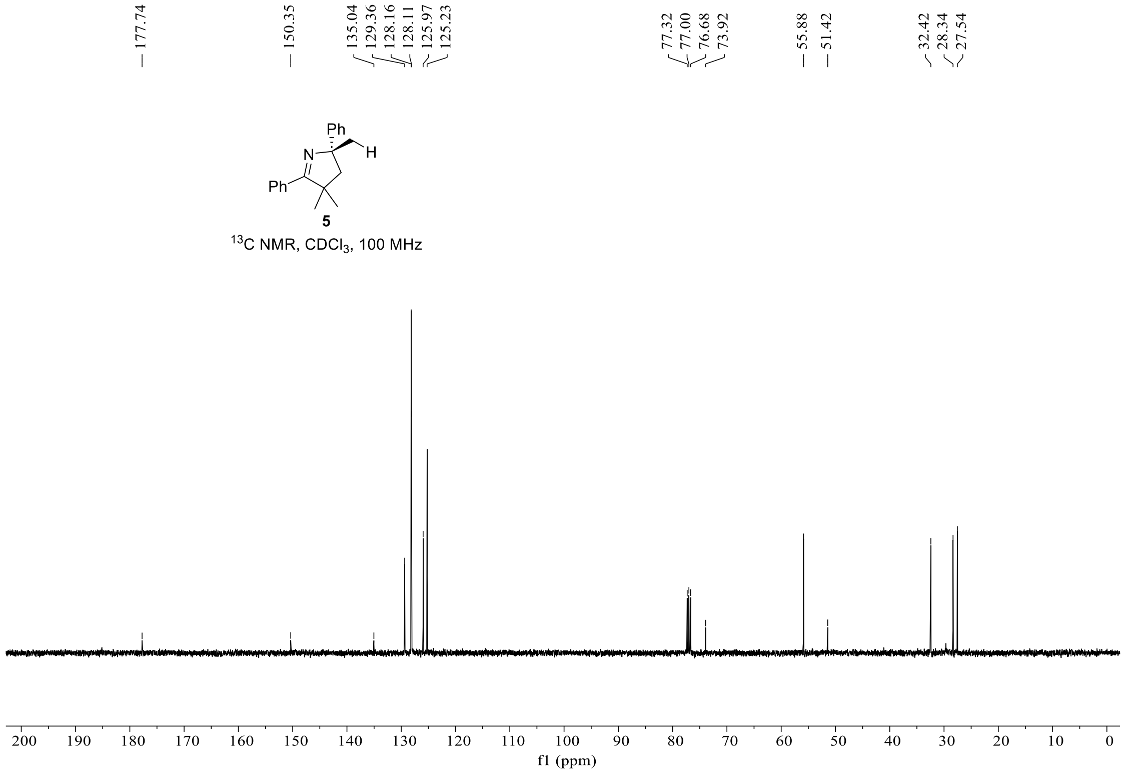
177.74 150.35 135.04 129.36 128.16 128.11 125.97 125.23 77.32 77.00 76.68 73.92 55.88 51.42 32.42 28.34 27.54
Ph
N
H
Ph
5
^{13}C NMR, $CDCl_3$, 100 MHz
200 190 180 170 160 150 140 130 120 110 100 90 80 70 60 50 40 30 20 10 0
f1 (ppm)

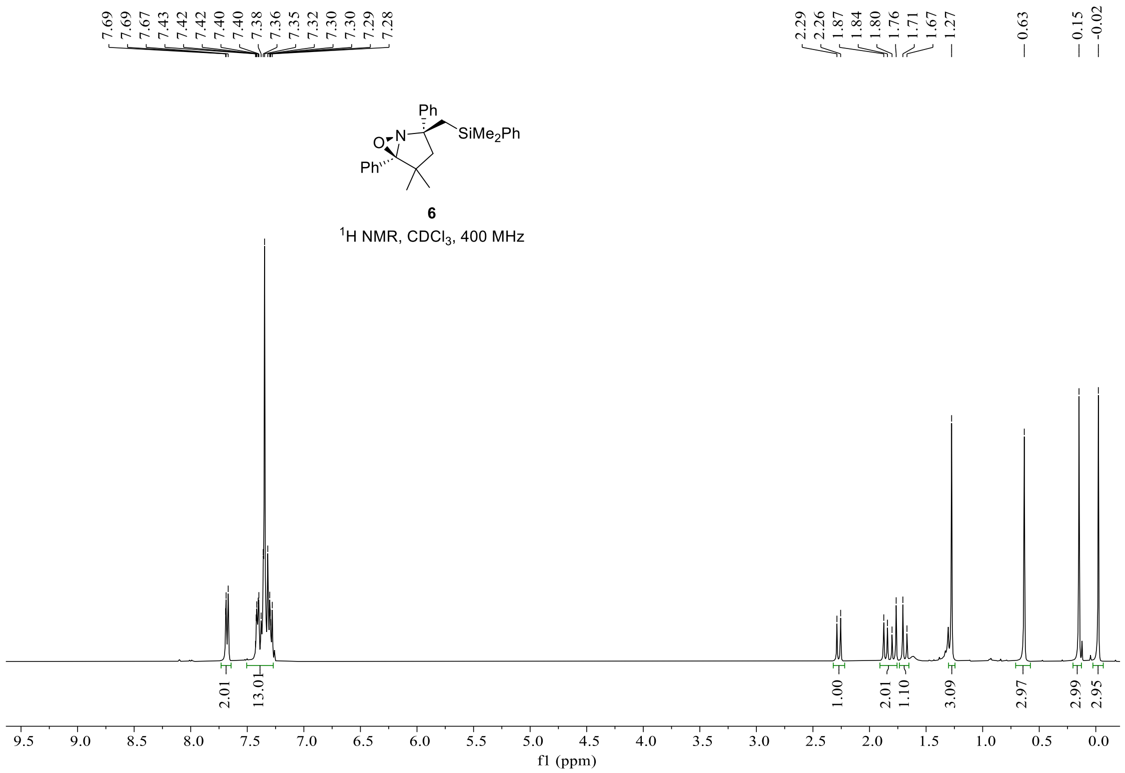

6

^{1}H NMR, $CDCl_3$, 400 MHz

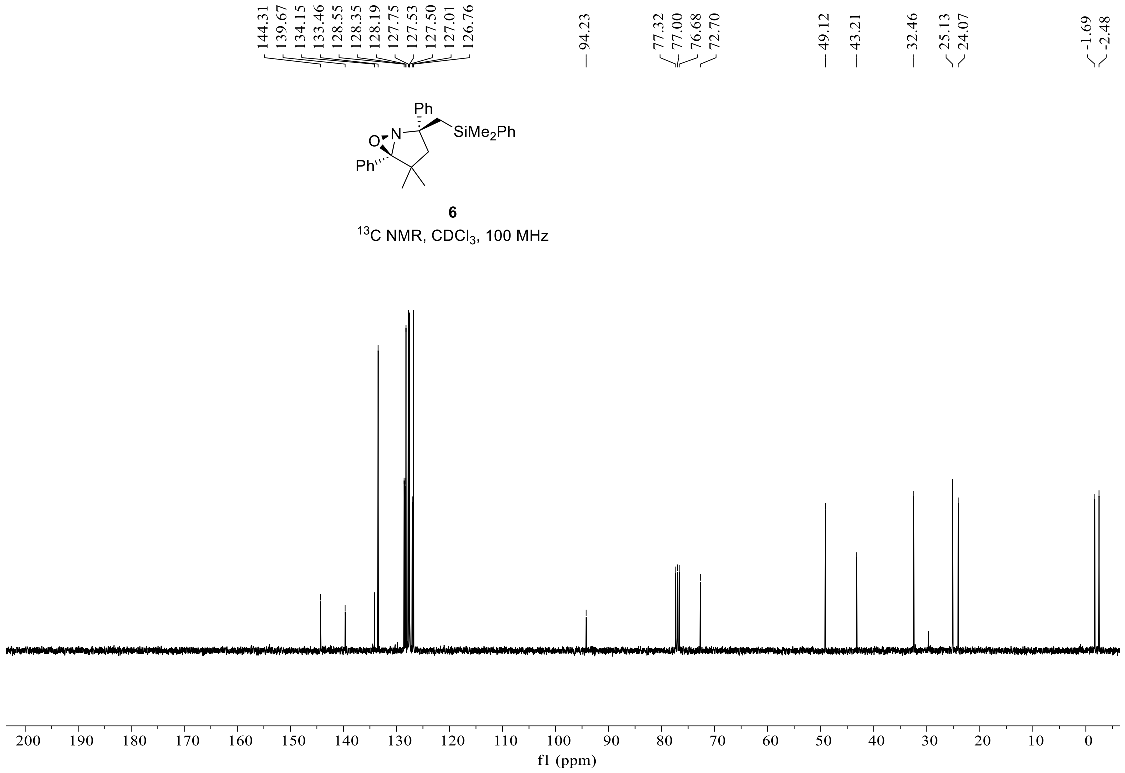

6

^{13}C NMR, $CDCl_3$, 100 MHz

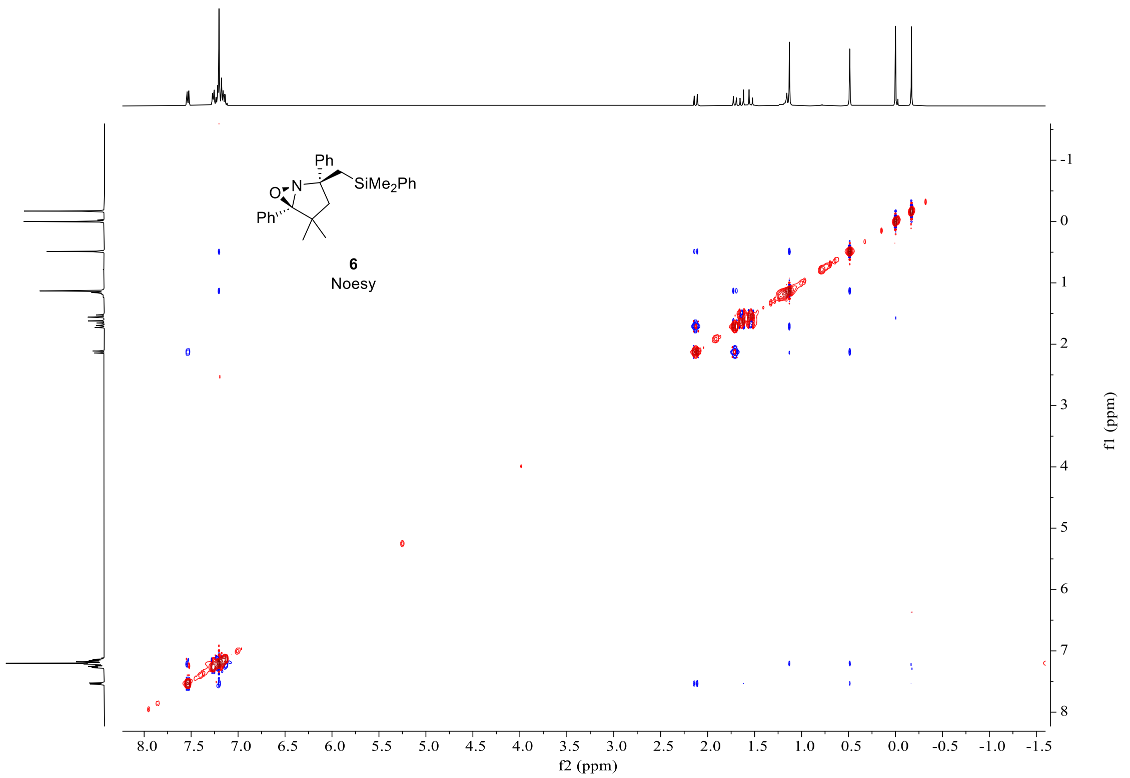

Ph
SiMe2Ph
O
N
Ph
6
Noesy
f2 (ppm)
f1 (ppm)

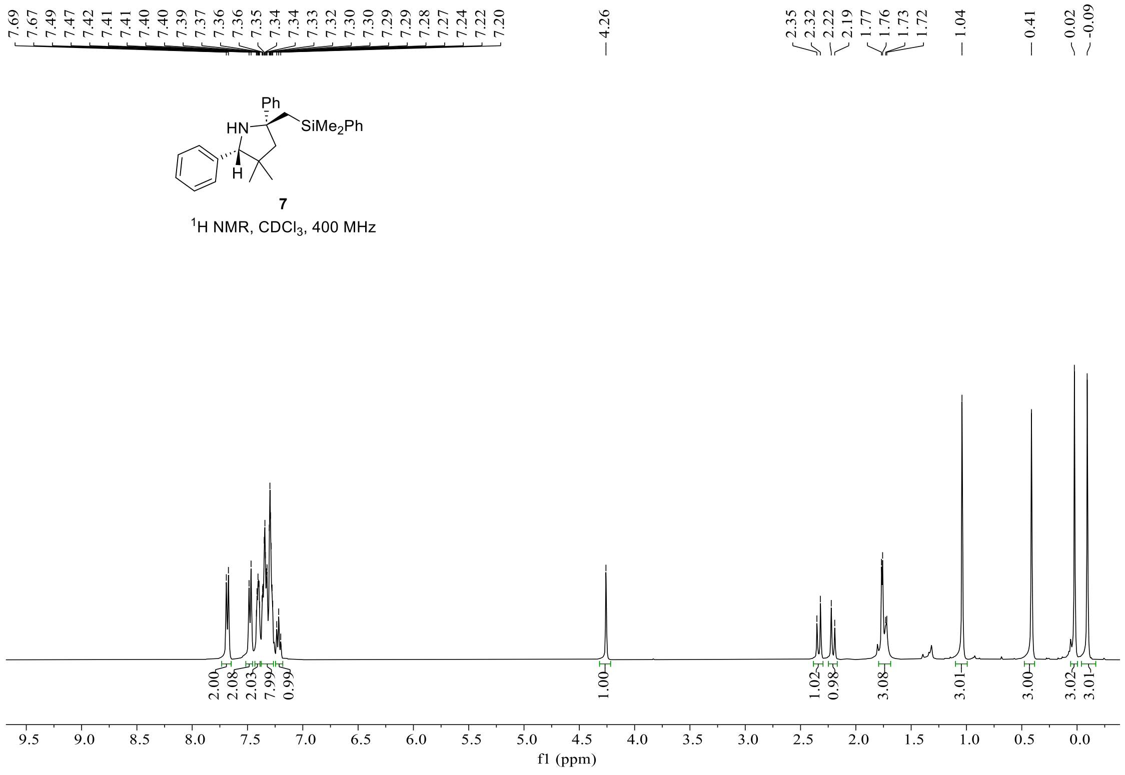

7.69 7.67 7.49 7.47 7.42 7.41 7.41 7.40 7.40 7.39 7.37 7.36 7.36 7.35 7.34 7.34 7.33 7.32 7.30 7.30 7.29 7.29 7.28 7.27 7.24 7.22 7.20
4.26
2.35 2.32 2.22 2.19 1.77 1.76 1.73 1.72
1.04
0.41
0.02 -0.09
Ph
HN
SiMe2Ph
H
7
1H NMR, CDCl3, 400 MHz
2.00 2.08 2.03 7.99 0.99
1.00
1.02 0.98
3.08
3.01
3.00
3.02 3.01
f1 (ppm)

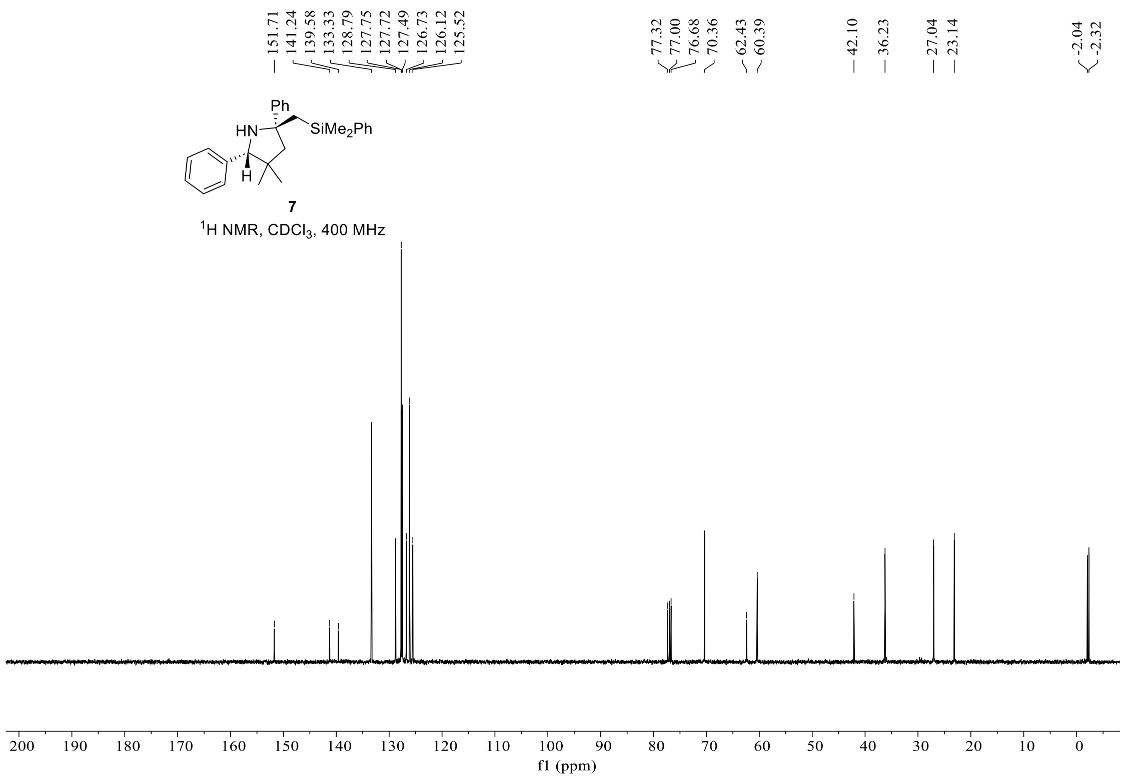

7

^{1}H NMR, $CDCl_3$, 400 MHz

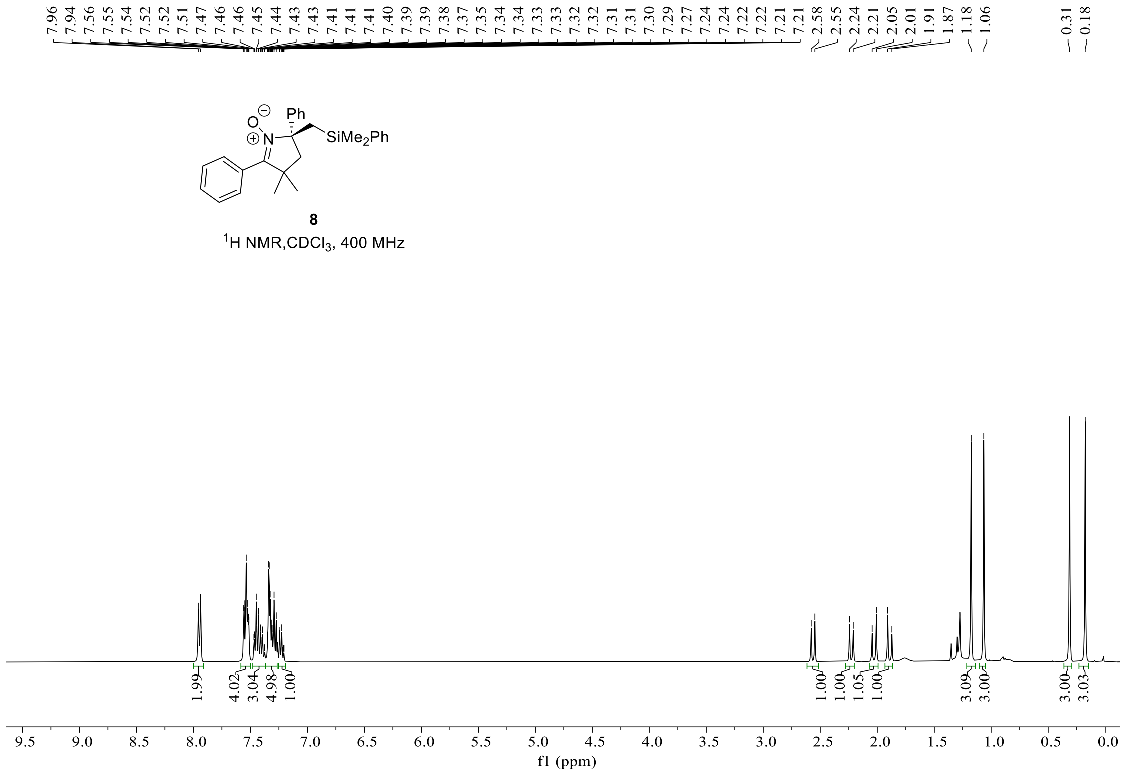

8

^{1}H NMR, $CDCl_3$, 400 MHz

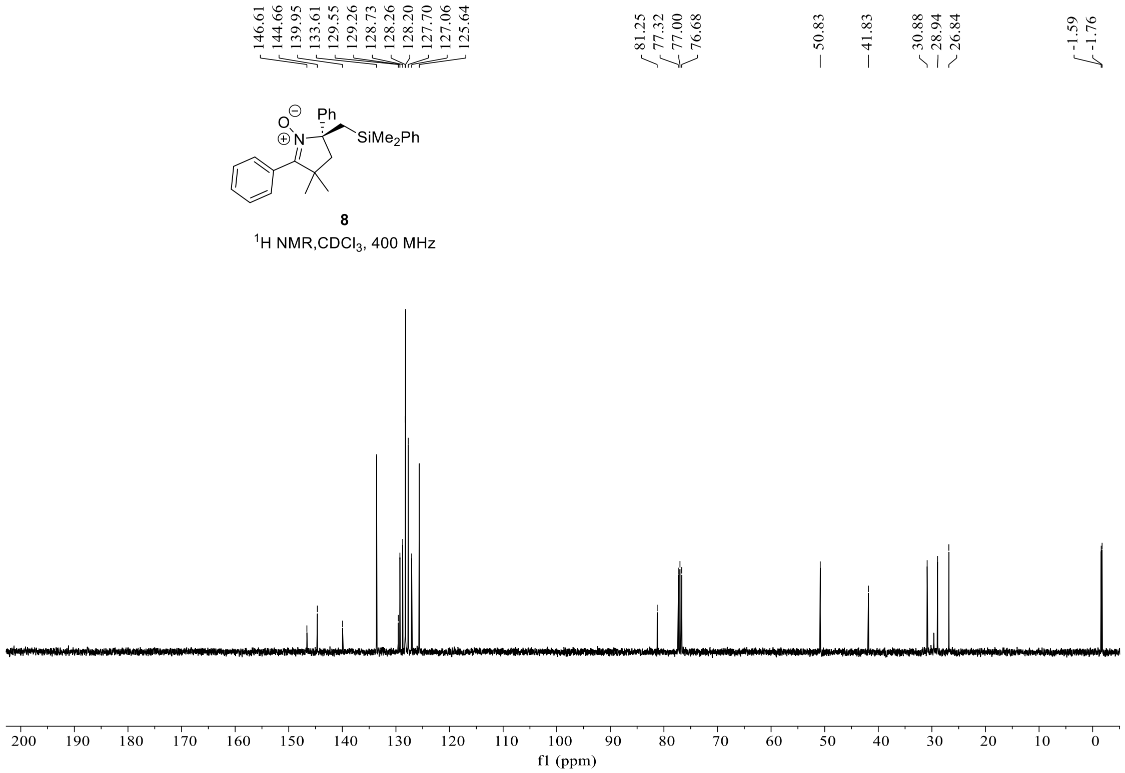

8

^{1}H NMR,CDCl$_3$, 400 MHz

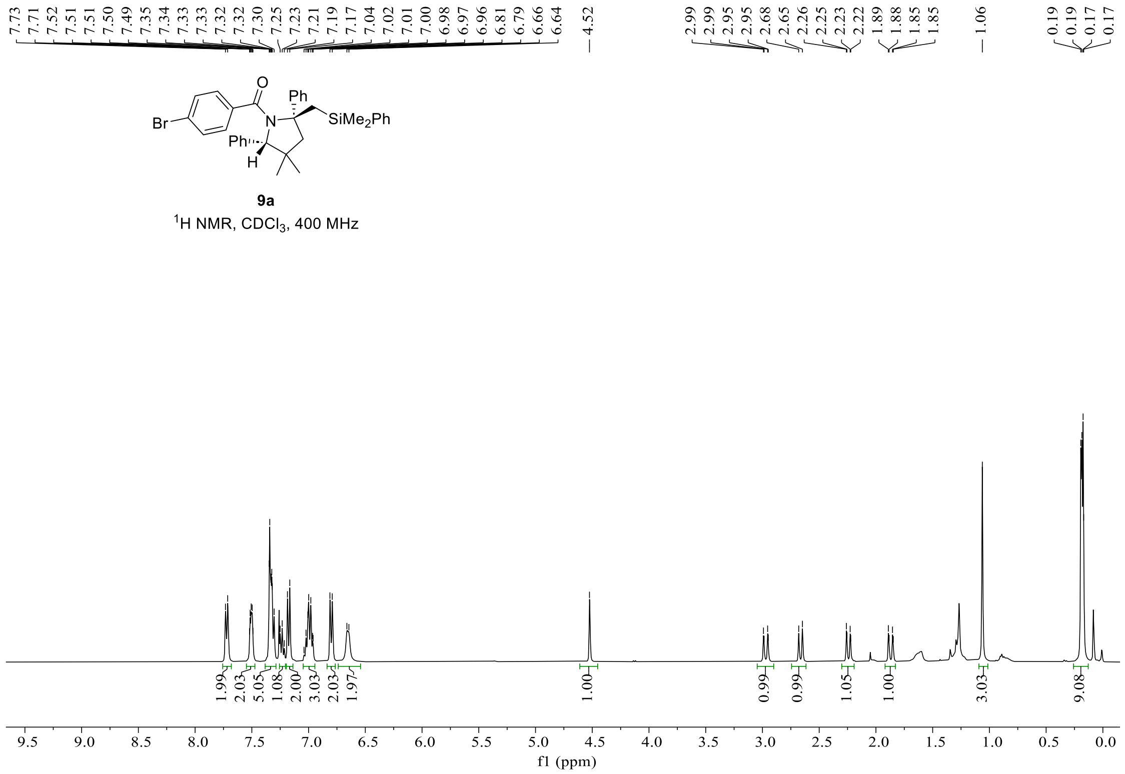

9a

^{1}H NMR, CDCl$_3$, 400 MHz

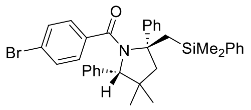

9a

^{13}C NMR, $CDCl_3$, 100 MHz

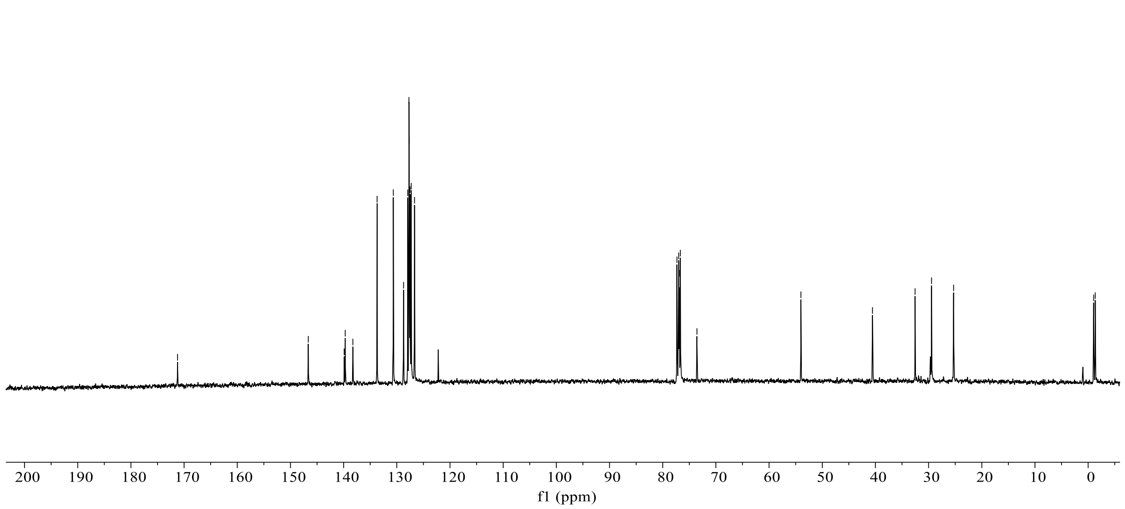

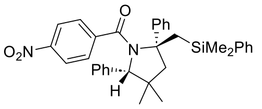

9b

^{1}H NMR, $CDCl_3$, 400 MHz

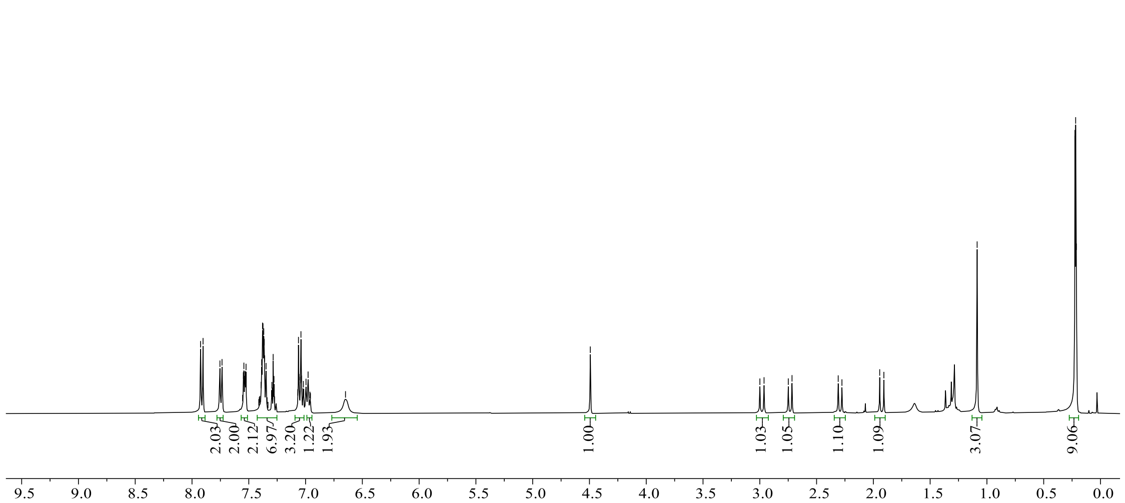

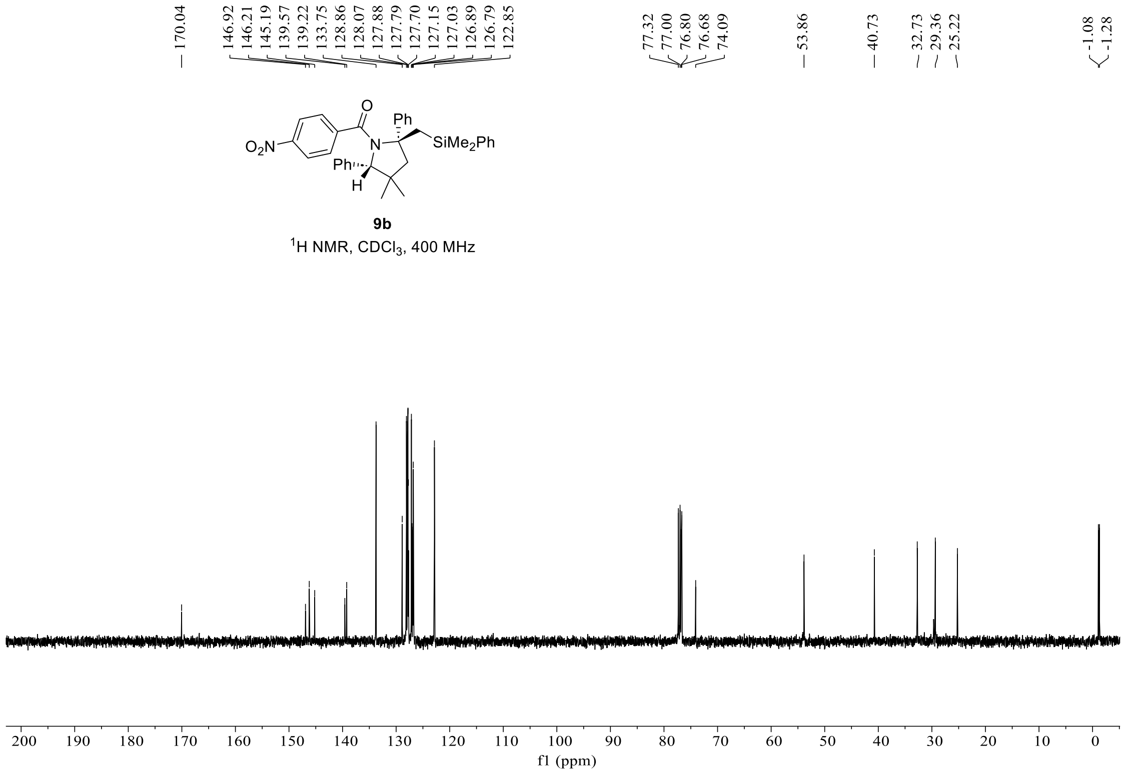
170.04
146.92
146.21
145.19
139.57
139.22
133.75
128.86
128.07
127.88
127.79
127.70
127.15
127.03
126.89
126.79
122.85
77.32
77.00
76.80
76.68
74.09
53.86
40.73
32.73
29.36
25.22
-1.08
-1.28
O_2N
O
N
Ph
$SiMe_2Ph$
Ph
H
9b
^1H NMR, $CDCl_3$, 400 MHz
200 190 180 170 160 150 140 130 120 110 100 90 80 70 60 50 40 30 20 10 0
f1 (ppm)

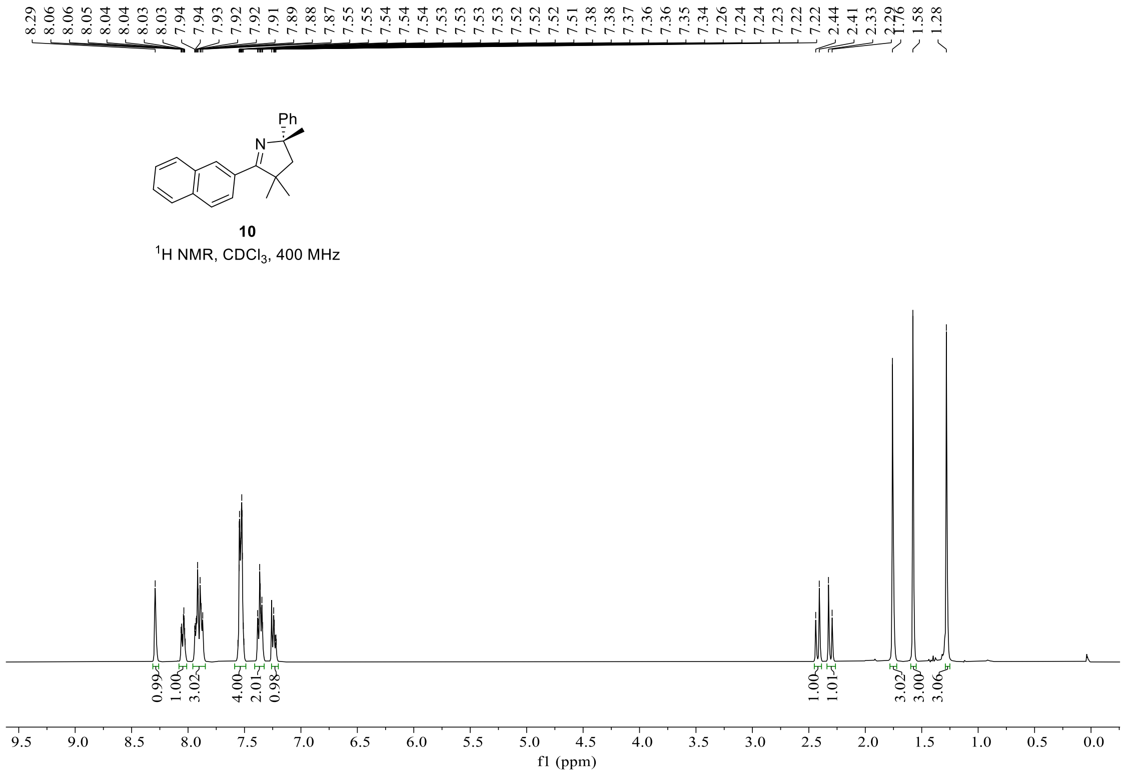
8.29
8.06
8.06
8.05
8.04
8.04
8.03
7.94
7.94
7.93
7.92
7.92
7.91
7.89
7.88
7.87
7.55
7.55
7.54
7.54
7.54
7.53
7.53
7.53
7.53
7.52
7.52
7.51
7.38
7.38
7.37
7.36
7.36
7.35
7.34
7.26
7.24
7.24
7.23
7.22
7.22
2.44
2.41
2.33
2.29
1.76
1.58
1.28
Ph
N
10
^1H NMR, $CDCl_3$, 400 MHz
0.99
1.00
3.02
4.00
2.01
0.98
1.00
1.01
3.02
3.00
3.06
9.5 9.0 8.5 8.0 7.5 7.0 6.5 6.0 5.5 5.0 4.5 4.0 3.5 3.0 2.5 2.0 1.5 1.0 0.5 0.0
f1 (ppm)

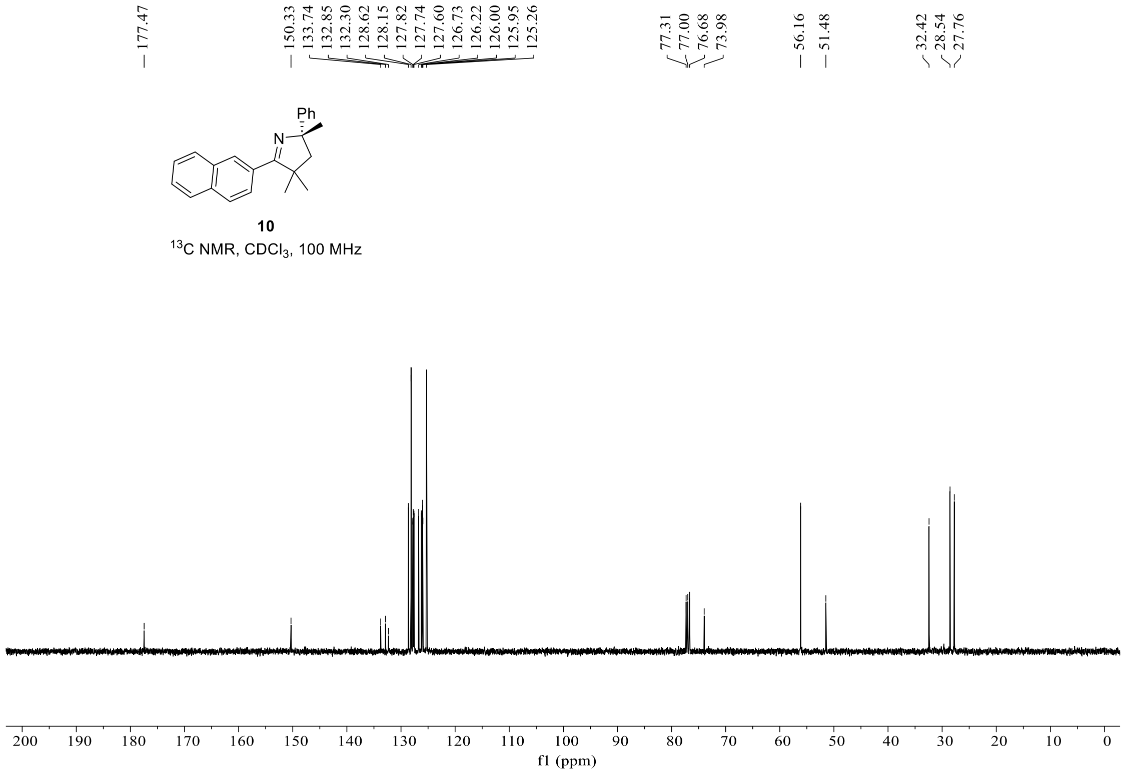
177.47
150.33 133.74 132.85 132.30 128.62 128.15 127.82 127.74 127.60 126.73 126.22 126.00 125.95 125.26
77.31 77.00 76.68 73.98
56.16
51.48
32.42 28.54 27.76
Ph
N
10
13C NMR, CDCl3, 100 MHz
200 190 180 170 160 150 140 130 120 110 100 90 80 70 60 50 40 30 20 10 0
f1 (ppm)

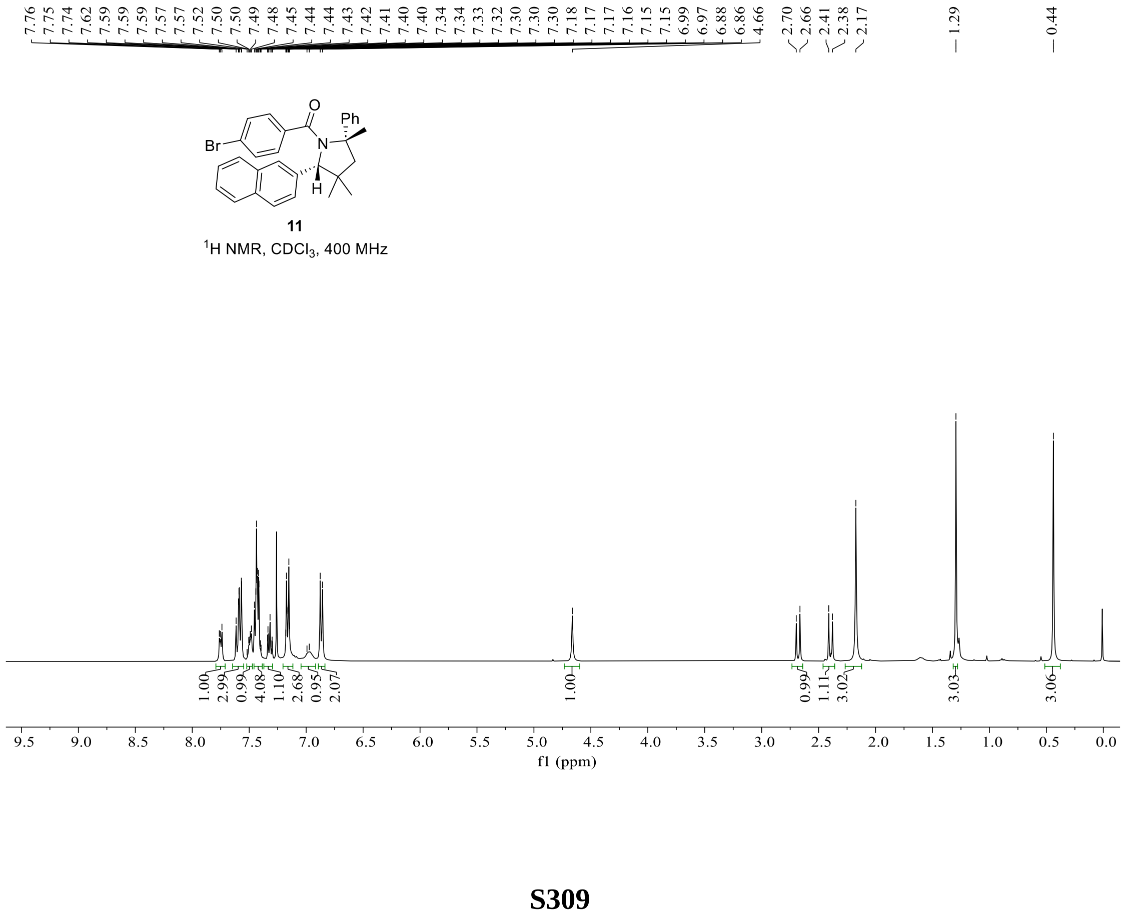
7.76 7.75 7.74 7.62 7.59 7.59 7.59 7.57 7.57 7.52 7.50 7.50 7.49 7.48 7.45 7.44 7.44 7.43 7.42 7.41 7.40 7.40 7.34 7.34 7.33 7.32 7.30 7.30 7.30 7.18 7.17 7.17 7.16 7.15 7.15 6.99 6.97 6.88 6.86 4.66
2.70 2.66 2.41 2.38 2.17
1.29
0.44
O
Ph
Br
N
H
11
1H NMR, CDCl3, 400 MHz
1.00 2.99 0.99 4.08 1.10 2.68 0.95 2.07
1.00
0.99 1.11 3.02
3.03
3.06
9.5 9.0 8.5 8.0 7.5 7.0 6.5 6.0 5.5 5.0 4.5 4.0 3.5 3.0 2.5 2.0 1.5 1.0 0.5 0.0
f1 (ppm)

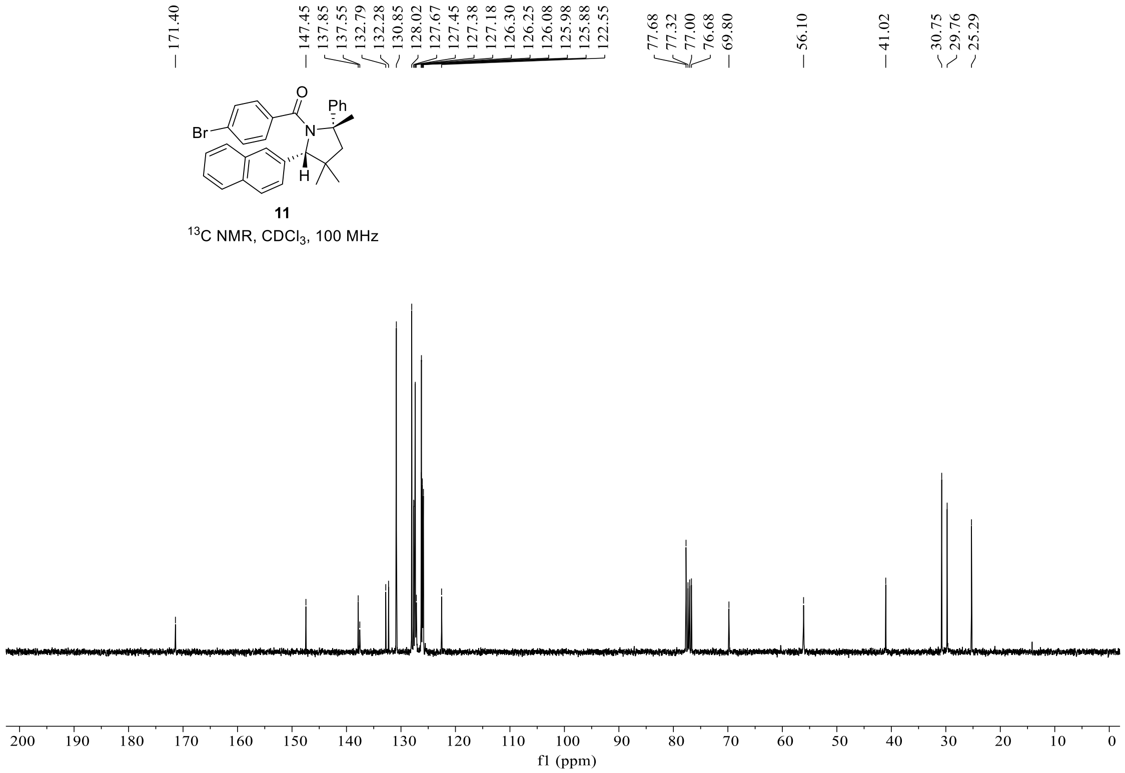
171.40
147.45
137.85
137.55
132.79
132.28
130.85
128.02
127.67
127.45
127.38
127.18
126.30
126.25
126.08
125.98
125.88
122.55
77.68
77.32
77.00
76.68
69.80
56.10
41.02
30.75
29.76
25.29
O
Ph
Br
N
H
11
13C NMR, CDCl3, 100 MHz
200 190 180 170 160 150 140 130 120 110 100 90 80 70 60 50 40 30 20 10 0
f1 (ppm)

10. References

1. Zhang, Z.; Chen, P.; Li, W.; Niu, Y.; Zhao, X.; Zhang, J. A New Type of Chiral Sulfinamide Monophosphine Ligands: Stereodivergent Synthesis and Application in Enantioselective Gold(I)-Catalyzed Cycloaddition Reactions. *Angew. Chem., Int. Ed.* **2014**, *53*, 4350-4354.

2. Zhou, W.; Su, X.; Tao, M.; Zhu, C.; Zhao, Q.; Zhang, J. Chiral Sulfinamide BisphosphineCatalysts: Design, Synthesis, and Application in Highly Enantiose-lective Intermolecular Cross-Rauhut−Currier Reactions. *Angew. Chem., Int. Ed.* **2015**, *54*, 14853−14857.

3. Wei, W.-X.; Li, Y.; Wen, Y.-T.; Li, M.; Li, X.-S.; Wang, C.-T.; Liu, H.-C.; Xia, Y.; Zhang, B.-S.; Jiao, R.-Q.; Liang, Y.-M. Experimental and Computational Studies of Palladium-Catalyzed Spirocyclization via a Narasaka−Heck/C(sp^3 or sp^2)−H Activation Cascade Reaction. *J. Am. Chem. Soc.* **2021**, *143*, 7868.

4. (a) He, Y.-X.; Hu, K.; Ran, Y.; Lei, Z.-Y.; Geng, S.; Chen, L.-N.; Pan, L.; Zhong, J.-B.; Huang, F. Intramolecular cascade cyclization via photogenerated N-amidyl radicals toward isoindolin-1-one/3,4-dihydroisoquinolin-1(2H)-one fused oxazinane. *Org. Chem. Front.*, **2023**, *10*, 4871–4877; (b) Nguyen, T. N. Thanh; Thiel, Niklas O.; Pape, Felix; Teichert, Johannes F. Copper(I)-Catalyzed Allylic Substitutions with a Hydride Nucleophile. *Org. Lett.* **2016**, *18*, 2455−245.

5. (a) Fleming, I.; Sanderson, P. E. J. *Tetrahedron Lett.* **1987**, *28*, 4229; (b) Lee, K.-S.; Hoveyda, A. H. Enantioselective Conjugate Silyl Additions to Cyclic and Acyclic Unsaturated Carbonyls Catalyzed by Cu Complexes of Chiral *N*-Heterocyclic Carbenes. *J. Am. Chem. Soc.* **2010**, *132*, 2898-2900.

6. Smith, J.; Kamath, A.; Greene, A. E.; Delair, P.; Total Synthesis of (+)-Hyacinthacine A_6 and (+)-Hyacinthacine A_7. *Synlett.*, **2014**, *25*, 0209-0212.

7. Wang, R.; Wang, C. Asymmetric imino-acylation of alkenes enabled by HAT-photo/nickel cocatalysis. *Chem. Sci.*, **2023**, *14*, 6449–6456.

8. Zhang, X.; Qi, D.; Jiao, C.; Zhang, Z.; Liu, X.; Zhang, G. Ni-Catalyzed Direct Iminoalkynylation of Unactivated Olefins with Terminal Alkynes: Facile Access to Alkyne-Labelled Pyrrolines. *Org. Chem. Front.* **2021**, *8*, 6522-6529.

9. Zhu, G.; Liu, S.; Wu, S.; Peng, L.; Qu, J.; Wang, B. Assembly of Indolenines, 3-Amino Oxindoles, and Aldehydes into Indolenine-Substituted Spiro-[pyrroli-din-2,3′-oxindoles] via 1,3-Dipolar Cycloaddition with Divergent Diastereoselectivities. *J. Org. Chem.* 2**017**, *82*, 4317-4327.

10. Ma, C.; Zhang, T.; Zhou, J.-Y.; Mei, G.-J.; Shi, F. Catalytic asymmetric chemodivergent arylative dearomatization of tryptophols. *Chem. Commun.*, **2017**, *53*, 12124-12127.

11. Frisch, M. J.; Trucks, G. W.; Schlegel, H. B.; Scuseria, G. E.; Robb, M. A.; Cheeseman, J.; R. Cooke, S.; Scalmani, G.; Barone, V.; Mennucci, B.; Petersson,

G. A.; Nakatsuji, H.; aricato, M.; Li, X.; Hratchian, H. P.; Izmaylov, A. F.; Bloino, J.; Zheng, G.; Sonnenberg, J. L.; Hada, M.; Ehara, M.; Toyota, K.; Fukuda, R.; Hasegawa, J.; Ishida, M.; Nakajima, T.; Honda, Y.; Kitao, O.; Nakai, H.; Vreven, T.; Montgomery, J.; J. A.; Peralta, J. E.; Ogliaro, F.; Bearpark, M.; Heyd, J. J.; Brothers, E.; Kudin, K. N.; Staroverov, V. N.; Kobayashi, R.; Normand, J.; Raghavachari, K.; Rendell, A.; Burant, J. C.; Iyengar, S. S.; Tomasi, J.; Cossi, M.; Rega, N.; Millam, N. J.; Klene, M.; Knox, J. E.; Cross, J. B.; Bakken, V.; Adamo, C.; Jaramillo, J.; Gomperts, R.; Stratmann, R. E.; Yazyev, O.; Austin, A. J.; Cammi, R.; Pomelli, C.; Ochterski, J. W.; Martin, R. L.; Morokuma, K.; Zakrzewski, V. G.; Voth, G. A.; Salvador, P.; Dannenberg, J. J.; Dapprich, S.; Daniels, A. D.; Farkas, Ö.; Foresman, J. B.; Ortiz, J. V.; Cioslowski, J.; Fox, D. J. G., Revision D.01, Gaussian, Inc., Wallingford CT, **2010**.

12. (a) Grimme, S.; Antony, J.; Ehrlich, S.; Krieg, H., *J. Chem. Phys.* **2010**, *132*, 154104-154108. (b) Grimme, S.; Ehrlich, S.; Goerigk, L., *J. Comput. Chem.* **2011**, *32*, 1456-1465. (c) Zhao, Q.; Jin, J.-K.; Wang, J.; Zhang, F.-L.; Wang, Y.-F., *Chem. Sci.* **2020**, *11*, 3909-3913.

13. Hay, P. J.; Wadt, W. R., *J. Chem. Phys.* **1985**, *82*, 270-283.

14. (a) Dolg, M.; Wedig, U.; Stoll, H.; Preuss, H., *J. Chem. Phys.* **1987**, *86*, 866-872. (b) Andrae, D.; Häußermann, U.; Dolg, M.; Stoll, H.; Preuß, H., *Theoretica chimica acta* **1990**, *77*, 123-141.

15. (a) Krishnan, R.; Binkley, J. S.; Seeger, R.; Pople, J. A., *J. Chem. Phys.* **1980**, *72*, 650-654. (b) Francl, M. M.; Pietro, W. J.; Hehre, W. J.; Binkley, J. S.; Gordon, M. S.; DeFrees, D. J.; Pople, J. A., *J. Chem. Phys.* **1982**, *77*, 3654-3665. (c) Clark, T.; Chandrasekhar, J.; Spitznagel, G. W.; Schleyer, P. V. R., *J. Comput. Chem.* **1983**, *4*, 294-301. (d) McLean, A. D.; Chandler, G. S., *J. Chem. Phys.* **2008**, *72*, 5639-5648.

16. Tomasi, J.; Mennucci, B.; Cammi, R., *Chem. Rev.* **2005**, *105*, 2999-3094.

17. (a) Johnson, E. R.; Keinan, S.; Mori-Sánchez, P.; Contreras-García, J.; Cohen, A. J.; Yang, W., *J. Am. Chem. Soc.* **2010**, *132*, 6498-6506. (b) Lu, T.; Chen, F., *J. Comput. Chem.* **2012**, *33*, 580-592. (c) Humphrey, W.; Dalke, A.; Schulten, K., *J. Mol. Graphics.* **1996**, *14*, 33-38.

18. Legault, C. Y. C., 1.0b; Universitéde Sherbrooke: Québec, Montreal, Canada; , **2009**, http://www.cylview.org.
